# A mega-cryptic species complex hidden among one of the most common annelids in the North East Atlantic

**DOI:** 10.1371/journal.pone.0198356

**Published:** 2018-06-20

**Authors:** Arne Nygren, Julio Parapar, Joan Pons, Karin Meißner, Torkild Bakken, Jon Anders Kongsrud, Eivind Oug, Daria Gaeva, Andrey Sikorski, Robert André Johansen, Pat Ann Hutchings, Nicolas Lavesque, Maria Capa

**Affiliations:** 1 Sjöfartmuseet Akvariet, Göteborg, Sweden; 2 Institutionen för marina vetenskaper, Göteborgs Universitet, Göteborg, Sweden; 3 Departamento de Bioloxía, Facultade de Ciencias, Universidade da Coruña, A Coruña, Spain; 4 Department of Biodiversity and Conservation, Mediterranean Institute for Advanced Studies, IMEDEA, Balearic Islands, Spain; 5 Senckenberg Forschungsinstitute und Naturmuseun, German Centre for Marine Biodiversity Research, Hamburg, Germany; 6 Norwegian University of Science and Technology, NTNU University Museum, Trondheim, Norway; 7 Department of Natural History, University Museum of Bergen, Bergen, Norway; 8 Norwegian Institute for Water Research, Region South, Grimstad, Norway; 9 Shirshov Institute of Oceanology, Russian Academy of Sciences, Moscow, Russia; 10 Akvaplan-niva AS, Fram Centre, Tromsø, Norway; 11 Institute of Marine Research, Tromsø, Norway; 12 Australian Museum Research Institute, Australian Museum, Sydney, New South Wales, Australia; 13 Centre National de la Recherche Scientifique & Université de Bordeaux, Environnements et Paléoenvironnements Océaniques et Continentaux, Station Marine d’Arcachon, Arcachon, France; 14 University of the Balearic Island, Department of Biology, Ctra. Valldemossa, Balearic Islands, Spain; National Cheng Kung University, TAIWAN

## Abstract

We investigate mitochondrial (*COI*, *16S rDNA*) and nuclear (*ITS2*, *28S rDNA*) genetic structure of North East Atlantic lineages of *Terebellides*, a genus of sedentary annelids mainly inhabiting continental shelf and slope sediments. We demonstrate the presence of more than 25 species of which only seven are formally described. Species boundaries are determined with molecular data using a broad range of analytical methods. Many of the new species are common and wide spread, and the majority of the species are found in sympatry with several other species in the complex. Being one of the most regularly encountered annelid taxa in the North East Atlantic, it is more likely to find an undescribed species of *Terebellides* than a described one.

## Introduction

The revelation of cryptic species has increased exponentially since the use of molecular data in taxonomic studies became common practise, but our understanding of the magnitude and importance of this neglected biodiversity is still at an early stage [1–3]. To unravel, describe and explain this hidden and unexplored dimension of life on earth is one of the major challenges to practising taxonomists [1].

This paper is a case study on the genus *Terebellides* Sars, 1835 (Annelida) based on specimens collected from North East Atlantic waters, ranging from the British Isles in the south, to the Polar Basin in the north. The genus and its first member, *Terebellides stroemii* Sars, 1835, was described from the west coast of Norway near Bergen. Even though a few other species of *Terebellides* were described during the 19th and 20th century, *T*. *stroemii* has, as many of the early described polychaetes, been considered to be a cosmopolitan species reported from all over the world and from a wide variety of habitats [4–5]. About 150 years after its description, Williams [6] revealed the existence of different morphotypes among members traditionally considered as *T*. *stroemii*, and described a few of them as new species, and since then, the number of descriptions of new species of *Terebellides* has increased [7–13]. Recently, Parapar and Hutchings [14] redescribed *T*. *stroemii*. The material used in the original description has been lost, but they designated a neotype from museum specimens collected by Michael Sars from a nearby locality [4, 14]. Today *T*. *stroemii* is considered to be restricted to the North East Atlantic where it coexists with other species of *Terebellides* [11, 15].

**Fig 1:**
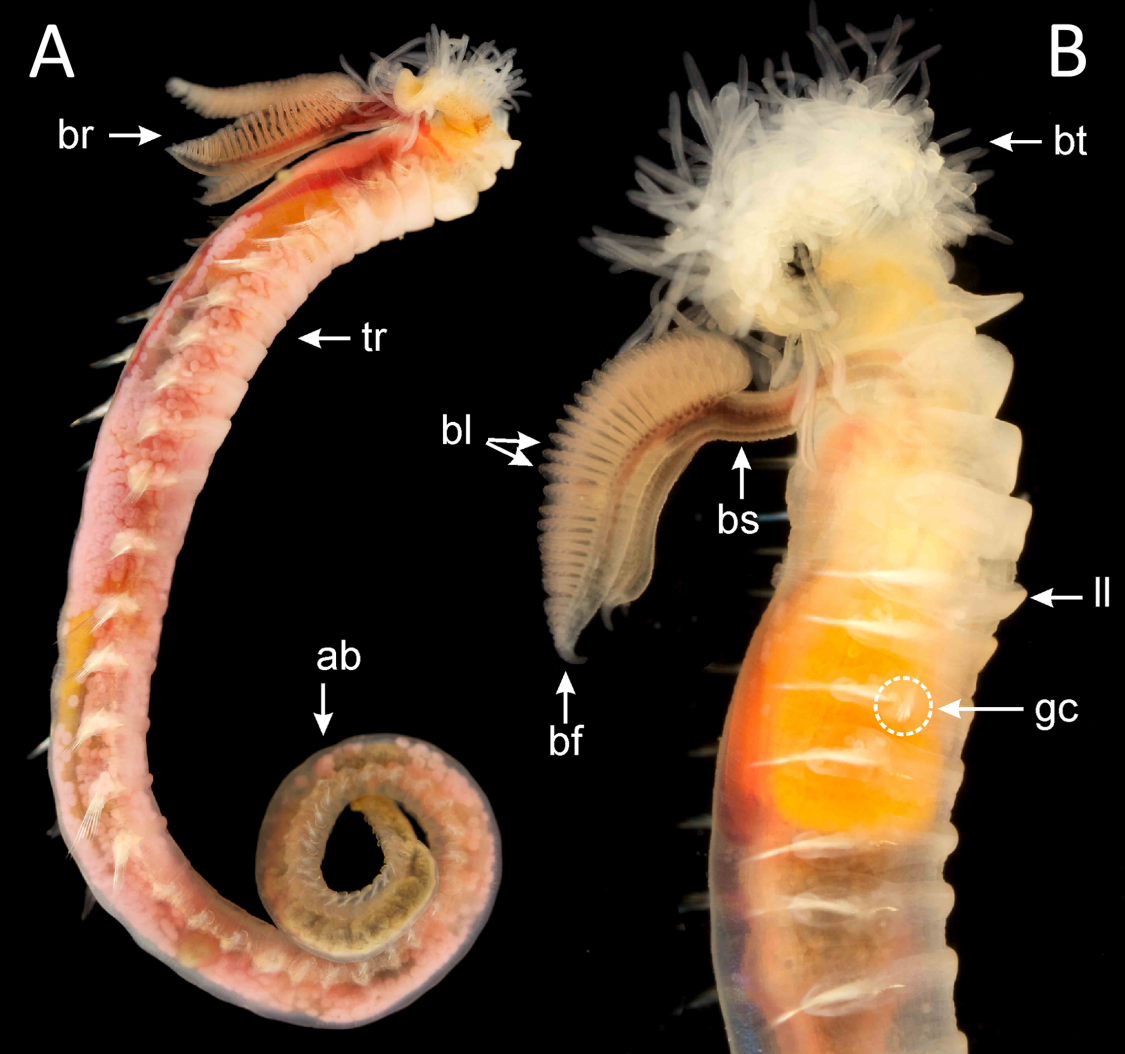
Live specimens of A) *Terebellides williamsae* (specimen 2181_2), in lateral view, with oocytes in the coelomic cavity and B) species 7 (specimen 2448_7), in lateral view. *Abbreviations*: ab (abdomen), bl (branchial lamellae), br (branchiae), bs (branchial stalk), bt (buccal tentacles), gc (geniculate chaetae), ll (lateral lappets), tr (thorax).

**Fig 2:**
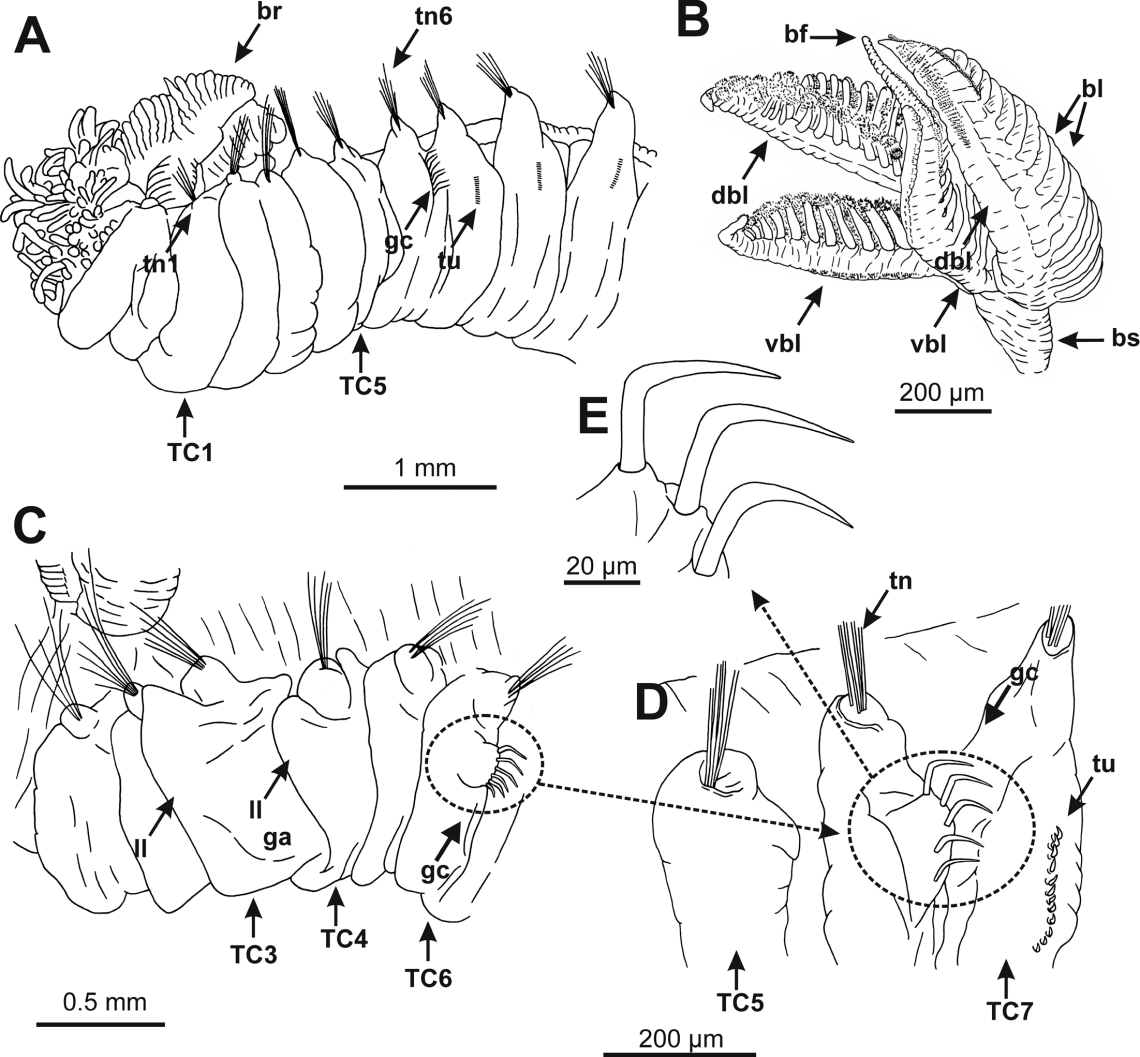
Line drawings made from different *Terebellides* species showing main macroscopic body characters with taxonomic relevance. A. Ventro-lateral view of *T*. *gracilis* or *T*. *williamsae* from Iceland showing most relevant taxonomic characters (e.g. position of anterior 1–5 thoracic chaetigers with whitish ventral colouration). B. Ventral view of branchiae in *T*. *shetlandica* from the Shetland Islands showing branchial stalk, size and shape of dorsal and ventral lobes, branchial lamellae, and branchial filaments. C. Left lateral view of anterior thoracic region of *T*. *cf stroemii* from Iceland showing lateral lappets in TC3 and TC4, position of geniculate chaetae in TC6 and enlarged glandular area in TC3. D. Detail of thoracic chaetigers TC5 to TC7 of *T*. *atlantis* from Iceland showing position of geniculate chaetae in TC6 and normal thoracic uncini in TC7. E. Detail of three geniculate chaetae. A, C, D, E redrawn from [11], B redrawn from [18]. *Abbreviations*: bf (branchial filament), bl (branchial lamellae), br (branchiae), bs (branchial stalk), dbl (dorsal branchial lobe), ga (glandular area), gc (geniculate chaetae), ll (lateral lappets), TC (thoracic chaetiger), tn (thoracic notopodium), tr (thorax), tu (thoracic uncini), vbl (ventral branchial lobe).

*Terebellides* is the most species-rich of three genera in Trichobranchidae, with 52 species considered valid [16]. Trichobranchidae is closely related to the more commonly known spaghetti worms (Terebellidae), ice-cone worms (Pectinariidae) and Pompeii worms (Alvinellidae) [17]. The genus *Terebellides* is morphologically a homogenous group characterized by its unique branchiae with a single mid-dorsal stalk on segment 3. Differences between species are mainly based on detailed branchial morphology, shape and size of anterior lobes, and on details of chaetae [14, 18, 19] (Figs 1 and 2).

Members of *Terebellides* are tube-dwelling surface deposit feeders, and they occur predominantly in soft bottoms on continental shelfs and slopes. The information on reproductive biology of the species is referred to *T*. *stroemii* exclusively. *Terebellides stroemii* spawns annually from the age of one or two years for the rest of their life (until the age of three to five years). Breeding season is reported to be in October–November in Greenland waters [20], in May in the Kiel Bay [21], and in March–April in the Mediterranean [22]. Further, *Terebellides stroemii* has been described to deposit their eggs in a compact, slimy mass, attached to pieces of decaying seagrass, or at the entrance to their tube. Fertilization probably occurs before the eggs are deposited, larvae emerge as trochophores, and the free-swimming larval stage is thought to be very short and supposedly spent in near-bottom layers [21].

**Fig 3:**
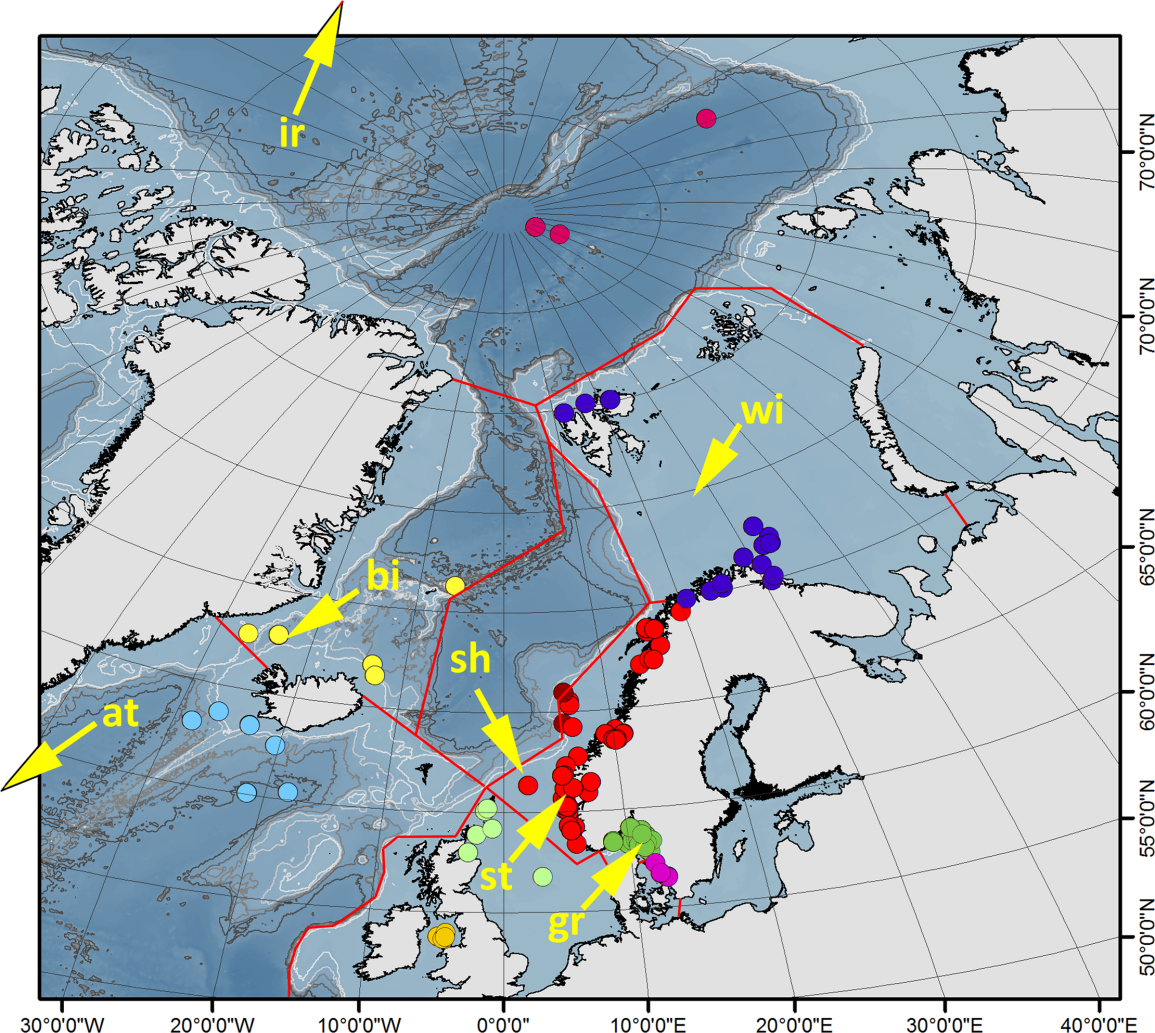
Collecting sites, biogeographic regions, and type localities for *Terebellides irinae* (ir), *T*. *atlantis* (at), *T*. *bigeniculatus* (bi), *T*. *shetlandica* (sh), *T*. *williamsae* (wi), *T*. *stroemii* (st), and *T*. *gracilis* (gr) indicated with an arrow. Type localities for *T*. *irinae* and *T*. *atlantis* are located outside the map's area. Biogeographic regions given by colours of samples (collecting sites) (see text for definitions): *Kattegat* (magenta); *Skagerrak* (dark green); *North Sea* (light green); *Irish Sea*, *Celtic Sea* (orange); *Norwegian coast and shelf* (red); *Norwegian Sea* (brown); *Barents Sea* (dark blue); *Arctic Ocean* (rose red); *Greenland Sea* (yellow); *South of Iceland* (light blue).

In the North East Atlantic, including the Arctic region but excluding the Mediterranean, seven species have been described or reported to date based on morphology alone, and these are *T*. *stroemii* with type locality in south-west Norway in 55–110 m, *T*. *gracilis* Malm, 1874 with type locality in Skagerrak in 65–230 m, *T*. *atlantis* Williams, 1984 with type locality on the New England slope in 400 m, *T*. *williamsae* Jirkov, 1989 with type locality in the Barents Sea between northern Norway and Svalbard in 385–390 m, *T*. *irinae* Gagaev, 2009 with type locality in the Canada Basin in Beaufort Sea off Alaska in 2570–2678 m, *T*. *bigeniculatus* Parapar, Moreira & Helgason, 2011 with type locality north-west of Iceland in 333 m, and *T*. *shetlandica* Parapar, Moreira & O'Reilly, 2016 with type locality between Shetland and the Norwegian coast in 160 m (Fig 3). Among these, *T*. *williamsae* is considered a junior synonym to *T*. *gracilis* [15].

In this paper, we report on a series of molecular genetic analyses of *Terebellides* from North East Atlantic waters using both mitochondrial (*COI*, *16S rDNA*) and nuclear genes (*ITS2*, *28S rDNA*). The main aim of the study is to answer how many species of *Terebellides* that are actually inhabiting the North East Atlantic. With species we mean separately evolving metapopulation lineages sensu de Quieroz 2007 [23], identifiable as such using a combination of mitochondrial and nuclear markers, see also [2] for a discussion on the species concept we use in this paper. Further, the study examines if the currently recognized species are to be considered valid, and if there are additional species not yet reported in the area. We also want to investigate the geographic and bathymetric distribution for the different *Terebellides* species, in order to answer whether the species are predominantly sympatric or allopatric, and whether there are any biogeographical and/or bathymetrical patterns. Finally, we also intend to explore the population structure within the different species.

## Material and methods

### Specimens, and study area

Specimens were collected between 2005 and 2014 on collecting trips, or by the following scientific expeditions, monitoring programs or institutes: Survey of Utsjöbankarna, SAMARIN (Marine surveys done by the Swedish Taxonomy Initiative), BIOICE (Benthic Invertebrates of Icelandic waters), MAREANO (Marine Area database for Norwegian waters), POLYSKAG (Marine bristle worms (Polychaeta) in coastal waters of Skagerrak), BIOSKAG 2 (Deep Skagerrak), IceAGE (Icelandic marine Animals: Genetics and Ecology), UNIS 2009 (University Centre in Svalbard), ACCESS (Arctic Climate Change, Economy and Society) expedition Polarstern in 2012, UM/BIO (University Museum and Department of Biology, Bergen) surveys, and Marbank (Biobank of Arctic Marine Organisms), Institute of Marine Research, Tromsø. All samples were collected prior to that the Nagoya protocol entered into force, thus there was no need for specific permissions. Sampling did not include endangered or protected species.

**Fig 4:**
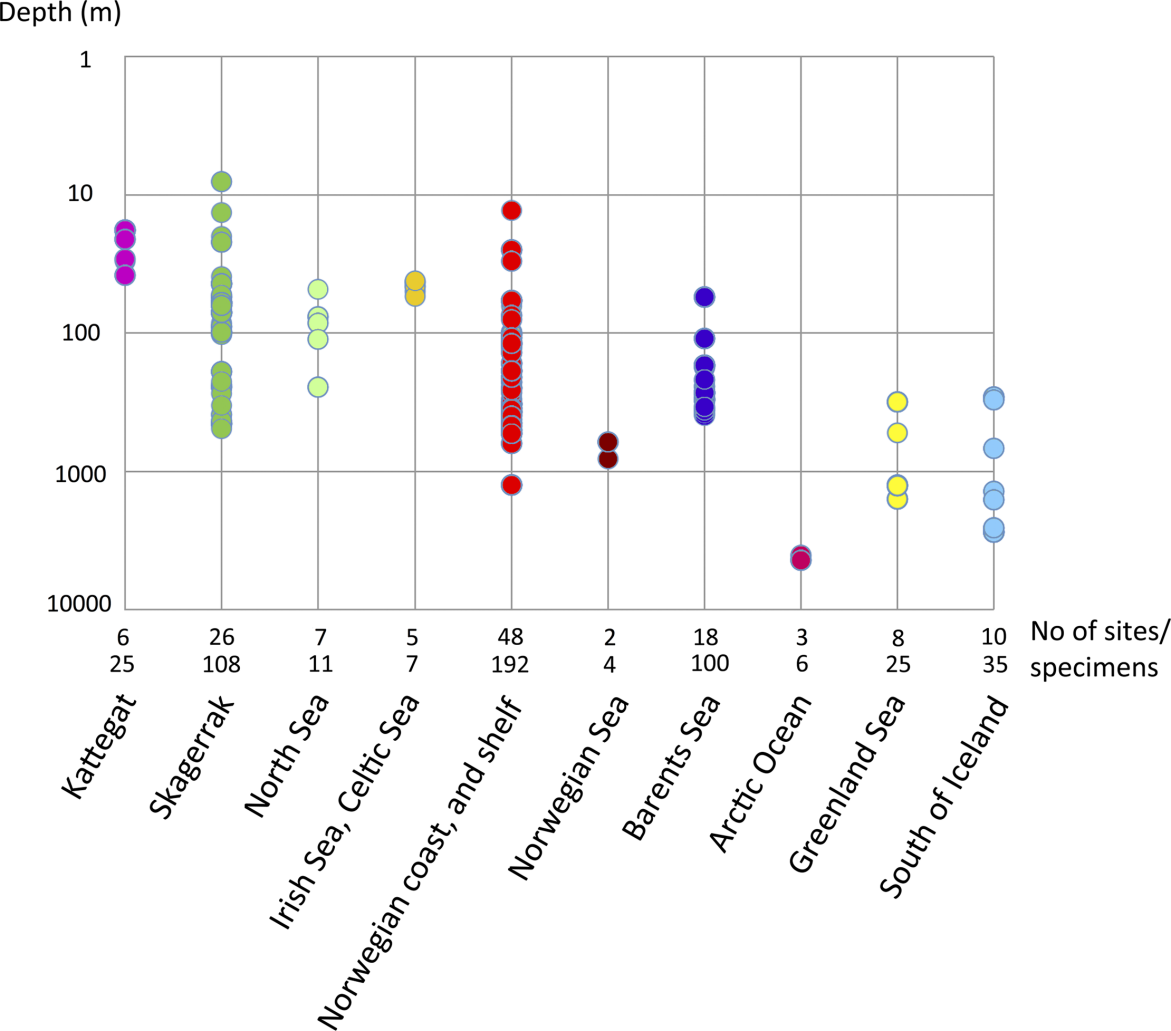
Depth distribution for collecting sites, including number of sites and specimens for each biogeographic region. Scale is logarithmic.

We analyzed 513 specimens from 133 collecting sites, in the depth range 8–4380 m (Figs 3 and 4), with the majority of the samples and specimens coming from the continental shelf along the Swedish and Norwegian coasts.

The study area was divided into the following biogeographic regions according to topographic and oceanographic features [24–26] (Fig 3). *Kattegat* (magenta dots in Fig 3), is a rather shallow area dominated by water masses from the North Sea, and heavily influenced by the Baltic Stream; *Skagerrak* (dark green), also a shallow shelf area, technically a part of the eastern part of the North Sea; *North Sea* (light green), shallow shelf area dominated by warm North Atlantic water masses; *Irish Sea*, *Celtic Sea* (orange), shelf areas, western UK and Ireland; *Norwegian coast and shelf* (red), north of Egersund to Loppa, areas <600 m except in the fjords, dominated by North Atlantic water with a mix of the less saline Norwegian coastal current; *Norwegian Sea* (brown), off the shelf break at approximately 600 m and deeper waters. Deeper areas below 800 m with permanent sub zero temperatures with Norwegian Sea deep water; *Barents Sea* (dark blue), separated from the Norwegian Sea by the shelf break between Norway and Svalbard, shelf sea dominated by cold water areas, but with a strong influence of North Atlantic water in the western areas and along the Troms and Finnmark coast [27]; *Arctic Ocean* (rose red), proper Polar Basin with permanent sub zero temperatures; *Greenland Sea* (yellow), with cold water areas with inflow of water from the Arctic Ocean by the East Greenland current; *South of Iceland* (light blue), area south of the Scotland-Faroe-Greenland ridge. Collecting data for specimens, together with voucher and GenBank accession numbers can be found in S36 Appendix and Table 1. Specimens are deposited in one of the following museums: Department of Natural History, University Museum of Bergen (ZMBN 116171–116514, 344 specimens), The Gothenburg Museum of Natural History (GNM 14625–15137, 74 specimens), Norwegian University of Science and Technology, NTNU University Museum, Trondheim (NTNU-VM 59990–72567, 36 specimens), and Senckenberg Museum Frankfurt (SMF 24368–24693, 59 specimens). All specimens are publicly deposited and accessible in a permanent repository.

**Table 1:** Locality and collecting data, including sample size, and species sampled.

SiteID	Geograhic area	Locality	Sample size	Clades sampled	Latitud, longitud (DD)	Depth (m)	Collecting date	Habitat	Gear
KA1	Kattegat	NE Hallands Väderö	11	4	56.44998, 12.60042	18–20	2007-05-25	Sand, fine gravel	Warén sledge
KA2	Kattegat	NE Hallands Väderö	2	4	56.451, 12.59828	18–20	2007-05-25	Sand, fine gravel	Rectangular dredge
KA3	Kattegat	W Laholmsbukten	5	12	56.49483, 12.64515	21–22	2007-05-25	Fine mud, shells	Rectangular dredge
KA4	Kattegat	E Anholt	1	1	56.68285, 12.107	30–33	2007-05-23	Clay, sand	Rectangular dredge
KA5	Kattegat	E Anholt	2	1	56.68452, 12.1096	29–32	2007-05-23	Clay, sand	Rectangular dredge
KA6	Kattegat	Fladen	4	6	57.19717, 11.82517	38	2005-06-17	Silt, sand	Van Veen grab
SK1	Skagerrak	W Kungälv	1	6	57.80798, 11.56585	20–28	2008-06-09	Shell, gravel,	Rectangular dredge
SK2	Skagerrak	W Kungälv	1	6	57.81822, 11.40038	39–67	2008-06-09	Shell, gravel	Rectangular dredge
SK3	Skagerrak		1	1	58.0081, 11.20107	85–98	2006-08-23	Sand, mud, gravel	Warén sledge
SK4	Skagerrak		4	1, 2, 5	58.14457, 10.71923	245–297	2008-06-12	Mud	Warén sledge
SK5	Skagerrak		2	2, 3	58.19173, 10.6648	237–277	2008-06-12	Mud, silt	Warén sledge
SK6	Skagerrak	Bonden	2	6	58.21947, 11.38658	8–18	2006-04-26	Mud, shells	Circular dredge
SK7	Skagerrak		7	8, 13	58.2237, 9.9267	453–477	2009-05-13	Mud	Sneli sledge
SK8	Skagerrak	Gullmarsfjorden	1	12	58.29163, 11.51393	53–105	2006-04-27	Mixed bottom	Agassiz trawl
SK9	Skagerrak	Gullmarsfjorden	9	12	58.29293, 11.51555	44–101	2006-04-27	Mixed bottom	Warén sledge
SK10	Skagerrak	Byfjorden	1	4	58.3255, 11.86183	13,5	2012-09-18	Sandy silty clay	Grab
SK11	Skagerrak		2	3, 13	58.3532, 10.3300	390–406	2009-05-13	Fine mud	Agassiz trawl
SK12	Skagerrak		2	8, 13	58.36037, 10.24012	429–445	2006-05-29	Soft bottom	Agassiz trawl
SK13	Skagerrak	Aust-Agder, Ryvingdypet	4	1, 8	58.36978, 8.72617	190	2011-05-28	Mud	RP sledge
SK14	Skagerrak		1	13	58.40322, 10.51548	273–365	2006-08-21	Mixed bottom	Rectangular dredge
SK15	Skagerrak	Aust-Agder, Ærøydypet	4	1	58.4066, 8.77758	90–100	2011-05-26	Mud	RP sledge
SK16	Skagerrak	Aust-Agder, Utnes	3	6	58.41023, 8.74602	22–32	2011-06-25	Algae, ascidians	Triangular dredge
SK17	Skagerrak		1	2	58.43017, 10.5800	248–335	2006-08-22	Soft clay	Agassiz trawl
SK18	Skagerrak		1	2	58.45702, 10.54635	224–286	2008-06-14	Hard bottom, mud	Rectangular dredge
SK19	Skagerrak		1	8	58.48285, 10.13443	491–531	2006-06-06	Soft bottom	Agassiz trawl
SK20	Skagerrak	E Väderöarna	4	6	58.58353, 11.08332	55–121	2008-06-15	Mixed bottom	Rectangular dredge
SK21	Skagerrak	W Grebbestad	1	1	58.68122, 11.11432	53–54	2008-06-16	Mixed bottom	Rectangular dredge
SK22	Skagerrak	W Tanum	2	6	58.73875, 10.73752	102–173	2008-06-15	Clay, mud	Rectangular dredge
SK23	Skagerrak	W Tanum	8	6, 12	58.7398, 10.73842	98–148	2008-06-15	Mixed bottom	Rectangular dredge
SK24	Skagerrak	Koster Area	25	1, 6	58.86667, 11.1	60–80	2005–04	Mud	Warén sledge
SK25	Skagerrak	SW Yttre Vattenholmen	13	1, 7	58.87417, 11.09472	62–71	2008-04-08	Mud	Rectangular dredge
SK26	Skagerrak	Vestfold, Sandefjord	7	1	59.05485, 10.25047	63–75	2011-05-29	Mud	RP sledge
NS1	North Sea		1	1	56.75, 3	111	2008-02-07	Soft bottom	Van Veen grab
NS2	North Sea		3	1	57.98075, -2.83516	76	2008–07	Sand, fine gravel	Grab
NS3	North Sea	E Orkney Island	1	9	58.87267, -2.19	85	2008–07	Sandy clay, gravel	Grab
NS4	North Sea	E Orkney Island	1	6	59.18933, -1.91867	85	2008–07	Sand, shell gravel	Grab
NS5	North Sea	W Shetland Islands	1	9	60.0675, -1.54467	111	2008–07	Silty clay, gravel	Grab
NS6	North Sea	S Shetland Islands	1	9	60.17983, -1.38883	48	2008–07	Sandy clay, gravel	Grab
NS7	North Sea		3	1	61.34553, 2.06935	246	2014-05-31	-	Grab
ISCS1	Irish Sea, Celtic Sea	S Isle of Man	1	6	53.60867, -4.38783	50	2010–07	Sand, gravel	Grab
ISCS2	Irish Sea, Celtic Sea	S Isle of Man	2	6	53.626, -4.46967	43	2010–07	Sand, gravel	Grab
ISCS3	Irish Sea, Celtic Sea	S Isle of Man	2	6	53.72067, -4.28283	46	2010–07	Sand, gravel	Grab
ISCS4	Irish Sea, Celtic Sea	S Isle of Man	1	6	53.73567, -4.83767	54	2010–07	Sand, gravel	Grab
ISCS5	Irish Sea, Celtic Sea	S Isle of Man	1	6	53.952, -4.27867	42	2010–07	Gravel	Grab
NCS1	Norwegian coast, shelf	Rogaland, S Kvitsøy	1	1	59.02712, 5.45419	64	2014-06-10	Sand, mud	Grab
NCS2	Norwegian coast, shelf	Rogaland, S Kvitsøy	11	1	59.02985, 5.44881	58–60	2014-06-10	Stones, gravel,sand	Triangular dredge
NCS3	Norwegian coast, shelf	Rogaland	4	8, 13	59.20548, 5.78051	226–242	2014-06-11	-	-
NCS4	Norwegian coast, shelf	Rogaland, Karmøysundet	3	1	59.28789, 5.32506	74–79	2014-06-08	Mud	RP sledge
NCS5	Norwegian coast, shelf	Hordaland, Langenuen	7	3, 5, 8	59.99, 5.35	250	2007-06-26	-	Warén sledge
NCS6	Norwegian coast, shelf	Hordaland, St Kalsøy	8	5	60.12, 5.07	119	2005-04-15	-	-
NCS7	Norwegian coast, shelf	Hordaland, Lysefjord	5	1, 7	60.21465, 5.3472	25–47	2007-06-28	-	-
NCS8	Norwegian coast, shelf	Hordaland, Fanafjord	1	1	60.2333, 5.28042	103	2014-05-19	Clay	Grab
NCS9	Norwegian coast, shelf	Hordaland, Skogsvåg	3	1	60.2691, 5.1157	98	2006-05-02	-	-
NCS10	Norwegian coast, shelf	Hordaland, Skogsvåg	3	1	60.26915, 5.11583	102	2008-03-17	-	-
NCS11	Norwegian coast, shelf	Hordaland, Herdlafjord	2	5, 28	60.51018, 5.19228	375	2007-04-20	-	-
NCS12	Norwegian coast, shelf	Hordaland, Mangerfjord	1	11	60.62360, 4.94120	325	2006-02-07	-	-
NCS13	Norwegian coast, shelf	Hordaland, Toskasundet	1	6	60.65862, 4.94718	13	2014-06-04	-	-
NCS14	Norwegian coast, shelf	Sogn & Fjordane, Aurlandsfjord	2	5, 11	60.90389, 7.16813	115	12-11-17	-	-
NCS15	Norwegian coast, shelf	Sogn & Fjordane, slope S Nesholmen	2	3, 13	61.08952, 5.21063	300–619	2012-11-15	-	Rectangular dredge
NCS16	Norwegian coast, shelf	Sogn & Fjordane—Møre & Romsdal	4	3	61.13339, 5.16632	631–644	2012-07-22	-	RP sledge
NCS17	Norwegian coast, shelf	Sogn & Fjordane, Sognefjorden	10	3, 8	61.14484, 5.91575	1259–1268	2012-11-16	-	RP sledge
NCS18	Norwegian coast, shelf	Sogn & Fjordane, Lustra-Nattropefjorden	20	3, 28	61.43212, 7.47763	327–337	2012-11-18	-	RP sledge
NCS19	Norwegian coast, shelf	Sogn & Fjordane—Møre & Romsdal	12	1, 3, 5, 8	61.80178, 5.08135	370–375	2012-07-20	-	RP sledge
NCS20	Norwegian coast, shelf	Sogn & Fjordane—Møre & Romsdal	5	3, 8, 13	61.82371, 5.21031	446–453	2012-07-20	-	RP sledge
NCS21	Norwegian coast, shelf	Sogn & Fjordane—Møre & Romsdal	1	7	62.27842, 5.45413	169–188	2012-07-21	-	-
NCS22	Norwegian coast, shelf	Møre & Romsdal, Harøyfjord	1	13	62.71988, 6.58989	126	2012-05-20	-	-
NCS23	Norwegian coast, shelf	Sør-Trøndelag, Trondheimsfjord	2	1	63.44500, 10.17010	30–51	2013-01-17	Sand, clay	Triangular dredge
NCS24	Norwegian coast, shelf	Sør-Trøndelag, Trondheimsfjord	8	2, 3, 5, 8, 13	63.47672, 9.92872	534	2013-01-17	Mud	Sneli sledge
NCS25	Norwegian coast, shelf	Sør-Trøndelag, Trondheimsfjord	6	5, 8, 13	63.47903, 10.21283	502–505	2013-01-17	Mud	Sneli sledge
NCS26	Norwegian coast, shelf	Sør-Trøndelag, Trondheimsfjord	2	8, 11	63.48733, 10.37383	271–334	2002-01-15	Mud	Triangular dredge
NCS27	Norwegian coast, shelf	Sør-Trøndelag, Trondheimsfjord	1	8	63.71208, 10.89915	420	2012-05-27	-	-
NCS28	Norwegian coast, shelf	Sør-Trøndelag, Trondheimsfjord	2	8	63.73615, 10.97631	419	2012-05-27	-	-
NCS29	Norwegian coast, shelf	Sør-Trøndelag, Frohavet	7	8, 13	63.75767, 9.20882	350–357	2010-05-10	Mud	Agassiz trawl
NCS30	Norwegian coast, shelf	Sør-Trøndelag, Åfjord	2	10	63.99012, 10.04445	102–110	2007-07-11	-	-
NCS31	Norwegian coast, shelf	Storegga	2	11, 28	64.19888, 6.06965	387–388	2013-06-26	Muddy sand	RP sledge
NCS32	Norwegian coast, shelf	Skjoldryggen	1	2	65.28217, 6.28326	357–369	2013-06-24	Sandy mud	RP sledge
NCS33	Norwegian coast, shelf	Skjoldryggen	3	11, 20, 28	65.50056, 6.26848	397–420	2013-06-23	Sandy mud	RP sledge
NCS34	Norwegian coast, shelf	Nordland, Holmsund	1	13	67.039251, 13.85357	259	2012-05-13	-	-
NCS35	Norwegian coast, shelf	Nordland, Skjærstadfjord	2	8	67.21783, 15.27833	476	2010-10-14	-	-
NCS36	Norwegian coast, shelf	Nordland, Skjærstadfjord	1	8	67.26417, 14.86983	513	2010-10-13	-	-
NCS37	Norwegian coast, shelf	Nordland, Hellemofjord	1	8	67.86733, 16.37033	461	2008-03-04	-	-
NCS38	Norwegian coast, shelf	Nordland, Hellemofjord	1	8	67.87383, 16.353	466	2008-03-04	-	-
NCS39	Norwegian coast, shelf	Sør-Trøndelag, Trondheimsfjord	1	13	68.47672, 9.92872	534	2013-01-17	Mud	Sneli sledge
NCS40	Norwegian coast, shelf	Nordland, Gullesfjord	1	15	68.59100, 15.80474	131	2008-11-05	-	-
NCS41	Norwegian coast, shelf	Nordland, Sortlandssundet	1	10	68.62817, 15.34959	128	2008-11-07	-	-
NCS42	Norwegian coast, shelf	Nordland, Sortlandssundet	2	10, 15	68.62856, 15.35318	122	2008-11-07	-	-
NCS43	Norwegian coast, shelf	Nordland, Gullesfjord	6	15	68.63708, 15.82157	165	2008-11-05	-	-
NCS44	Norwegian coast, shelf	Nordland, Gullesfjord	3	15	68.64117, 15.83652	139	2008-11-05	-	-
NCS45	Norwegian coast, shelf	Nordland, Gullesfjord	7	8, 15	68.71076, 16.01100	209	2008-11-06	-	-
NCS46	Norwegian coast, shelf	Nordland, Sortlandssundet	4	10, 13, 15	68.79015, 15.41222	108	2008-11-08	-	-
NCS47	Norwegian coast, shelf	Nordland, Sortlandssundet	4	10	68.79663, 15.41033	119	2008-11-08	-	-
NCS48	Norwegian coast, shelf	Troms, Balsfjord	14	14, 15	69.37333, 19.06167	187	2014-10-27	-	Sledge
NWS1	Norwegian Sea	Storegga	1	16	64.39374, 5.57426	814–819	2013-06-26	Sandy mud	RP sledge
NW2	Norwegian Sea	Skjoldryggen	3	2, 3	65.94317, 5.83320	610–612	2013-06-17	Sandy mud	RP sledge
BS1	Barents Sea	Finnmark, Varangerfjord	3	2	69.91217, 30.888	351	2014-04-15	Mud	RP sledge
BS2	Barents Sea	Troms, Ullsfjorden, S Karlsøya	3	8, 10	69.95333, 20.07183	243	2009-12-07	-	-
BS3	Barents Sea	Finnmark, Altafjord	2	8	70.1165, 23.07533	392	2009-12-09	-	-
BS4	Barents Sea	Finnmark	1	2	70.11767, 31.35033	303–304	2013-08-19	Mud	RP sledge
BS5	Barents Sea	Finnmark, Porsangerfjord	7	14, 15	70.12002, 25.18625	109	2011-10-08	Mud	Van Veen grab
BS6	Barents Sea	Finnmark, Porsangerfjord	2	2, 13	70.35324, 25.26369	178	2009-05-30	-	-
BS7	Barents Sea	Finnmark	2	2, 10	70.77383, 30.78117	377–378	2013-08-17	Mud	Beam traw
BS8	Barents Sea	Finnmark	1	13	71.056, 29.65567	337	2014-04-21	Muddy sand	Large Van Veen grab
BS9	Barents Sea	Finnmark	3	2, 13	71.321, 29.1965	362	2014-04-24	Mud	Beam traw
BS10	Barents Sea	Finnmark, TOO	6	2, 16, 21	71.61416, 33.0041	305	2013-08-09	Mud, clay	Beam traw
BS11	Barents Sea	Finnmark, TOO	8	2, 13, 16, 21	71.61527, 32.99719	305–306	2013-08-09	Mud, clay	RP sledge
BS12	Barents Sea	Finnmark, TOO	4	2, 16, 21	71.61817, 32.23133	297–298	2013-08-08	Sandy mud	RP sledge
BS13	Barents Sea	Finnmark, TOO	2	2, 16	71.9085, 33.44717	219–220	2013-08-06	Muddy sand, gravel	RP sledge
BS14	Barents Sea	Finnmark, TOO	26	2, 16, 28	72.57905, 32.38726	271–272	2013-08-03	Sandy mud	RP sledge
BS15	Barents Sea	Svalbard	10	12, 14, 25, 26, 27	79.8195, 12.0876	55	2009-09-01	-	RP sledge
BS16	Barents Sea	Svalbard	18	12, 21	80.1010, 22.2006	171	2009-09-01	-	RP sledge
BS17	Barents Sea	Svalbard	1	21	80.1086, 22.1414	216	2009-09-01	-	RP sledge
BS18	Barents Sea	Svalbard	1	21	80.1524, 16.9354	340	2009-09-01	-	RP sledge
AO1	Arctic Ocean		2	24	81.927, 130.91666	4038	2012-09-04	-	Multi grab
AO2	Arctic Ocean		1	24	87.92683, 61.01217	4380	2012-09-19	-	Multi grab
AO3	Arctic Ocean		3	24	88.7865, 56.372	4373	2012-09-23	-	Multi grab
GS1	Greenland Sea	NE Iceland	2	16	66.53817, -12.86483	316–317	2011-09-22	Silty mud	RP sledge
GS2	Greenland Sea	NE Iceland	2	2	66.54383, -12.87467	315–317	2011-09-22	Silty mud	RP sledge
GS3	Greenland Sea	NE Iceland	1	13	66.55483, -12.86483	316–317	2011-09-22	Silty mud	RP sledge
GS4	Greenland Sea	NE Iceland	5	16	67.07867, -13.06383	1575–1581	2011-09-21	Silty mud	RP sledge
GS5	Greenland Sea	Denmark Strait	1	16	67.63583, -26.7665	315–316	2011-09-14	Silty mud	RP sledge
GS6	Greenland Sea	Denmark Strait	4	16	67.8465, -23.696	1249–1250	2011-09-15	Silty mud	RP sledge
GS7	Greenland Sea	Denmark Strait	9	10, 16	67.86783, -23.69633	1267–2181	2011-09-15	Silty mud	RP sledge
GS8	Greenland Sea	Jan Mayen	1	16	71.29733, -5.77350	528	2011-06-15	-	-
SI1	South of Iceland	Iceland Basin	1	16	60.0455, -21.46767	2747–2749	2011-08-28	Silty mud	RP sledge
SI2	South of Iceland	Iceland Basin	9	16	60.04617, -21.47567	2747–2750	2011-08-29	Silty mud	RP sledge
SI3	South of Iceland	Iceland Basin	2	16	60.35733, -18.13567	2568–2569	2011-08-30	Silty mud	RP sledge
SI4	South of Iceland	Iceland Basin	3	16	60.35733, -18.13567	2568–2572	2011-08-30	Silty mud	RP sledge
SI5	South of Iceland	Iceland Basin	3	18	62.55167, -20.39517	1385–1389	2011-09-02	Silty mud	RP sledge
SI6	South of Iceland	Irminger Basin	4	16, 19, 23	63.00767, -28.06817	1569–1594	2011-09-08	Silty mud	RP sledge
SI7	South of Iceland	Reykjanes Ridge	3	3, 17, 22	63.3085, -23.15767	285–289	2011-09-04	Silty mud	RP sledge
SI8	South of Iceland	Reykjanes Ridge	3	3	63.31467, -23.16017	288–294	2011-09-04	Silty mud	RP sledge
SI9	South of Iceland	Reykjanes Ridge	3	3	63.33333, -23.16667	305	2011-09-04	Silty mud	RP sledge
SI10	South of Iceland	Irminger Basin	4	3, 16, 20	63.70883, -26.38417	678–698	2011-09-09	Silty mud	RP sledge

### Data retrieval

We extracted DNA with QuickExtract DNA Extraction (Epicentre). A small piece, usually one or two parapodia, were put in 50–100 μl QuickExtract, and treated with 65°C for 45 min followed by 2 min in 95°C in a dry block thermostat. We used the primers 16SANNF (GCGGTATCCTGACCGTRCWAAGGTA) [28] or 16SARL (CGCCTGTTTATCAAAAACAT), together with 16SBRH (CCGGTCTGAACTCAGATCACGT) [29]) for *16S rDNA*; LCO1490 (GGTCAACAAATCATAAAGATATTGG) and HCO2198 (TAAACTTCAGGGTGACCAAAAAATCA) [30], or COIE (TATACTTCTGGGTGTCCGAAGAATCA) [31] for *COI*; 28SC1 (ACCCGCTGAATTTAAGCAT) and 28SD2 (TCCGTGTTTCAAGACGG) [32] for *28SrDNA* (D1-D2 region); and ITS58SF (GAATTGCAGGACACATTGAAC) and ITS28SR (ATGCTTAAATTCAGCGGGT) [33] for *ITS2*.

PCR mixtures contained 0.33 μl of each primer (10μM), 1 μl of DNA template, and 10 μl of RedTaq 1.1x MasterMix 2.0 mM MgCl_2_ (VWR). Temperature profile was as follows: a denaturation step at 96°C for 1 minute, 29 cycles (95°C for 30 seconds– 52°C (for COI and 16S rDNA) or 62°C (for ITS2 and 28S rDNA) for 30 seconds– 72°C for 60 seconds), and a final step at 72°C for 7 minutes. PCR products were run for c. 15 minutes on a 1% agarose gel electrophoresis, containing GelRed Nuclear Acid Stain (Bioticum), and then visualized under UV-light. PCR products were purified using ExoSAP-IT PCR Product Cleanup protocol (ThermoScientific). Sanger sequencing was performed on both strands at Eurofins Genomics, DNA Sequencing Department in Ebersberg, Germany. Overlapping complementary strands were merged into consensus sequences using Geneious version 7.0.6 [34].

### Sequence data

In total, we amplified and sequenced the mitochondrial *COI* (up to 658bp) and *16S rDNA* (c. 440 bp), and the nuclear *ITS2* (290–419 bp) and *28S rDNA* (c. 760 bp) from 513 specimens of *Terebellides* spp from the North East Atlantic. Final data coverage was as follows: *COI*, 462 spms (90%) (GenBank accession numbers: MG024894–MG025355), *16S rDNA*, 75 spms (15%) (GenBank accession numbers: MG025443–MG025517), *ITS2*, 402 spms (90%) (GenBank accession numbers: MG024492–MG024893), and *28S rDNA*, 86 spms (17%) (GenBank accession numbers: MG025356–MG025441) (S36 Appendix and Table 2).

**Table 2:** Overview of sequence coverage for each genetic marker (*COI*, *ITS2*, *16S rDNA*, *28S rDNA*) and respective clade, as well as the combination of *COI* and *ITS2* (used in the STACEY analysis), and the combination including specimens with at least three out of the four genetic markers (CONCAT).

Clade number	Number of specimens	COI	ITS2	COI and ITS2	16S rDNA	28S rDNA	CONCAT
**1**	82	63	63	44	3	5	5
**2**	36	32	28	24	3	4	4
**3**	57	50	55	48	4	5	5
**4**	14	14	13	13	4	4	4
**5**	19	19	18	18	4	4	4
**6**	36	33	25	22	2	4	4
**7**	12	12	6	6	4	5	5
**8**	41	40	29	28	3	3	3
**9**	3	2	2	1	2	2	2
**10**	12	12	7	7	3	3	3
**11**	5	5	3	3	3	3	3
**12**	23	23	17	17	3	6	6
**13**	27	26	25	24	3	5	5
**14**	20	18	19	17	3	4	4
**15**	18	15	16	13	3	4	4
**16**	62	55	50	43	6	6	8
**17**	1	1	1	1	1	1	1
**18**	3	3	2	2	2	2	2
**19**	1	1	1	1	1	1	1
**20**	2	2	2	2	2	2	2
**21**	18	18	2	2	1	1	1
**22**	1	1	1	1	1	1	1
**23**	1	1	1	1	1	1	1
**24**	6	5	4	3	4	3	4
**25**	4	4	3	3	2	2	2
**26**	3	1	3	1	2	2	2
**27**	1	1	1	1	1	1	1
**28**	5	5	5	5	4	2	4
	**513**	**462**	**402**	**351**	**75**	**86**	**91**

Sequences from individual specimens can be identified by the extraction number and an appended clade-number (S36 Appendix), preliminary circumscribed from statistical parsimony haplotype networks [35], also known as TCS-analyses, of *COI*-data (see below). One other member of Trichobranchidae, *Trichobranchus roseus* (Malm, 1874), and two representatives of Terebellidae, *Polycirrus* Grube, 1850 and *Pista cristata* (Müller, 1776) were selected to root the tree [17]. Outgroups were used when assessing the general phylogeny of the *Terebellides* lineages, but not in the species delimitation analyses. Molecular data for outgroups were either retrieved as above (*Trichobranchus roseus*: *COI* (GenBank accession number MH113923), and *16S rDNA* (GenBank accession number MG025442), specimen voucher ZMBN 120609), or downloaded from GenBank (*Polycirrus*: *COI* = JX423769, *16S rDNA* = JX423681, *28S rDNA* = JN936481, and *Pista cristata*: *COI* = EU239688, *16S rDNA* = NC011011, *28S rDNA* = DQ790057).

### Alignments

We used MAFFT version 7.017 [36] within Geneious version 7.0.6 with the following settings: algorithm = E-INS-i, scoring matrix = 200PAM / k = 2, gap open penalty = 1.53, to align *16S rDNA* and *28S rDNA*. Aligning was unproblematic since the sequences were of similar length and resulting alignments had a moderate number of indels. The *ITS2*-region was challenging to align due to a high number of indels, and we proceeded with aligning using two approaches. In the first approach, we removed identical haplotypes with the uniqhaplo.pl script (S35 Appendix) leaving a data set with 136 unique *ITS2*-sequences. As we experienced problems with two sequences that were shorter due to incomplete 3'-end, these sequences were first removed (1999_13 and 2865_24), and the remaining 134 complete, or nearly complete, sequences were aligned with the X-INS-i algorithm in MAFFT that takes into account the secondary structure of the sequence. Subsequently the short excluded sequences were reincluded with the mafft-add command. The resulting alignment is referred to as *ITS2*x-unique. In the second approach, the sequences in the *ITS2*x-unique alignment were realigned using the software RNAsalsa [37], using the secondary structure of *ITS2* modeled for *Eumida ockelmanni* Eibye-Jacobsen, 1987 (GenBank accession number HM358782) [38] as a constraint, and implementing default parameters. The resulting alignment is referred to as *ITS2*s-unique. Identical sequences removed in the first step with the uniqhaplo.pl script were then added back to the two alignments by hand in Geneious version 7.0.6 mimicking the gaps present in those identical sequences aligned. The two resulting alignments with all 402 *ITS2*-sequences are referred to as *ITS2*x-all, and *ITS2*s-all. Finally, we used the MUSCLE alignment option in Geneious version 7.0.6 to align all 462 *COI*-sequences (*COI*-all) which was trivial due to the absence of indels. Identical *COI*-sequences were removed using uniqhaplo.pl script creating an alignment with 271 unique *COI*-sequences (*COI*-unique). Where relevant, aligned gene partitions were concatenated using Mesquite v. 2.75 (Maddison and Maddison 2008) [39]. For the statistical parsimony haplotype analyses, we used *COI*-all, and the two *ITS2*-all alignments as a starting point. Sequences of each haplotype network were extracted separately, and subsequently these clade data sets were pruned to remove gaps in flanking positions that was caused by incomplete sequencing. The purpose of this was to obtain the same data coverage for all included specimens in each haplotype network, and allowing for an unambiguous assessment of haplotypes. In a few instances, one, or a few of the shortest sequences were removed prior to pruning the sequence ends (Tables 3 and 4). In the choice between removing short sequences or pruning we chose the method that kept the maximum number of haplotypes. As there were a few ambiguities assessing number of haplotypes between the two *ITS2*-alignments, although based on the same data, we decided to realign the *ITS2*-data from each network separately, using the E-INS-i algorithm in MAFFT, with scoring matrix = 200PAM / k = 2, and gap open penalty = 1.53. The rational behind this is that aligning more similar sequences will result in a more accurate alignment. For the distance calculations we used *COI*-all, and *ITS2*s-all alignments. All different alignments, and data set combinations described above are available as S1–S9 Appendixes.

**Table 3:** Summary of haplotype and distance analyses for COI, with specification of excluded sequences, alignment length, number of haplotypes, and uncorrected intra- and interspecific distances. Species number to which the species is compared with, for the minimum and maximum interspecific distances, in parentheses.

Species number	Number of specimens	Removed sequences in haplotype analysis	Original alignment length	Pruned alignment length	Number of haplotypes	Uncorrected intraspecific distance	Minimum uncorrected interspecific distance (%)	Maximum uncorrected interspecific distance (%)
**1**	63		658	555	12	0–1.9	15.6–17.7 (7)	17.4–20.3 (8)
**2**	32		658	569	25	0–2.4	13.9–16.0 (3)	19.6–21.5 (21)
**3**	50		658	615	44	0–2.3	13.9–16.0 (2)	20.1–22.4 (21)
**4**	14		658	615	7	0–1.0	9.9–10,7 (26)	20.9–22.7 (10)
**5**	19		658	600	10	0–1.1	12.3–14.0 (16)	19.6–21.6 (15)
**6**	33	1314_6	658	609	10	0–0.8	8.8–10.8 (7)	19.2–20.4 (27)
**7**	12		658	627	8	0–0.6	8.8–10.8 (6)	19.2–20.9 (4)
**8**	40	1203_8	658	612	33	0–3.1	10.5–12.8 (7)	19.1–21.5 (15)
**9**	2		649	603	2	0.2	11.2–12.1 (7)	20.5–21.9 (4)
**10**	12		658	593	4	0–1.9	11.5–12.9 (11)	20.9–22.7 (4)
**11**	5		630	615	4	0–1.1	11.5–12.9 (10)	19.5–19.7 (26)
**12**	23		658	606	16	0–1.3	8.2–9.7 (13)	19.1–20.5 (2)
**13**	26	1959_13	658	597	14	0–1.9	8.2–9.7 (12)	19.5–21.3 (15)
**14**	18		658	615	5	0–0.3	16.0–17.4 (1)	20.1–21.1 (24)
**15**	15		658	567	4	0–0.5	17.2–18.6 (6)	19.5–21.8 (16)
**16**	55	2325_16	658	579	48	0–2.4	12.3–14.0 (5)	19.5–21.8 (15)
**17**	1		NA	NA	1	NA	14.6–15.6 (6)	20.6–21.4 (20)
**18**	3		627	624	3	0.5–0.6	13.0–14.3 (10)	20.7–21.4 (4)
**19**	1		NA	NA	1	NA	12.1–12.5 (10)	19.6–20.8 (3)
**20/28**	7		630	621	2	0–3.4	12.1–13.2 (21)	20.4–22.0 (22)
**21**	18		658	585	2	0–0.3	12.0–13.2 (20)	20.1–22.4 (3)
**22**	1		NA	NA	1	NA	13.1–13.6 (25)	20.4–22.0 (20)
**23**	1		NA	NA	1	NA	17.4–18.9 (16)	22.9 (24)
**24**	5		618	510	2	0–0.02	16.0–17.1 (25)	22.9 (23)
**25**	4		624	567	2	0–0.8	13.1–13.6 (22)	20.7–21.7 (23)
**26**	1		NA	NA	1	NA	9.9–10.7 (4)	22.1 (23)
**27**	1		NA	NA	1	NA	11.1–12.3 (4)	20.7–21.8 (10)

**Table 4:** Summary of haplotype and distance analyses for ITS2, with specification of excluded sequences, alignment length, number of haplotypes, and uncorrected intra- and interspecific distances. Species number to which the species is compared with, for the minimum and maximum interspecific distances, in parentheses.

Species number	Number of specimens	Removed sequences in haplotype analysis	Original alignment length	Pruned alignment length	Number of haplotypes	Uncorrected intraspecific distance (%)	Minimum uncorrected interspecific distance (%)	Maximum uncorrected interspecific distance (%)
**1**	63	856, 858, 1941, 1955, 2860, 2789, 2909	316	274	18	0–2.6	13.2–19.9 (26)	24.7–28.9 (15)
**2**	28		291	257	8	0–1.7	3.9–6.7 (3)	26.9–31.2 (25)
**3**	55		303		8	0–3.4	3.9–6.7 (2)	30.7–31.8 (23)
**4**	13		369		1	0	0.56–0.85 (26)	32.3–33.7 (15)
**5**	18		343		4	0–1.5	1.8–3.2 (16)	28.5–31.9 (21)
**6**	25		335	268	8	0–2.8	4.4–9.2 (10)	23.6–30.3 (14)
**7**	6		322		4	0–2.2	6.2–10.5 (8)	25.0–29.7 (14)
**8**	29	2896	327	292	5	0–1.2	6.2–10.5 (7)	29.6–33.0 (25)
**9**	2		317		1	0	6.7–10.7 (8)	26.3–30.4 (14)
**10**	7		326	295	1	0–0.33	4.9–6.6 (12)	26.2–28.5 (27)
**11**	3		350	323	2	0–0.31	9.8–12.4 (12)	30.2–32.9 (4)
**12**	17	2818	368	347	10	0–1.7	2.6–4.2 (13)	26.7–30.6 (14)
**13**	25		357	288	3	0–0.64	2.6–4.2 (12)	28.1–31.6 (14)
**14**	19	2477, 2479, 2852	361	332	6	0–1.5	9.4–13.9 (5)	30.6–35.3 (15)
**15**	16		305	273	1	0	16.9–18.4 (2)	30.6–35.3 (14)
**16**	50		348		4	0–0.87	1.8–3.2 (5)	28.9–32.2 (21)
**17**	1		315		1	NA	14.4–17.1 (1)	27.2–29.4 (21)
**18**	2		344		1	0	8.5–8.9 (10)	24.2–26.9 (14)
**19**	1		312		1	NA	6.4–11.9 (8)	23.5–27.5 (14)
**20/28**	7		410		1	0	3.0–3.3 (21)	30.2–31.9 (15)
**21**	2		419	391	1	0	3.0–3.3 (20)	32.1–33.4 (15)
**22**	1		303		1	NA	19.7–22.0 (24)	30.0–31.1 (21)
**23**	1		305		1	NA	8.8–9.7 (10)	24.3–28.0 (14)
**24**	4		324	223	1	0	9.9 (25)	30.2–33.4 (21)
**25**	3		309		1	0	9.9 (24)	32.6–34.4 (14)
**26**	3		365	184	1	0	0.56–0.85 (4)	22.3–33.9 (15)
**27**	1		375		1	NA	1.6 (4)	32.3–33.8 (15)

### Data set combinations

For a robust assessment of the evolutionary relationships of the *Terebellides* lineages, specimens for which three or four of the genetic markers were present (i.e. *COI*, *16S rDNA*, *ITS2*, *28S rDNA*), were combined into a data set comprising 91 *Terebellides* specimens (S36 Appendix and Table 2, last column) plus three outgroups. This was done by combining *COI*-all with either *ITS2*x-all or *ITS2*s-all, concatenating *16S rDNA* and *28S rDNA*, but excluding specimens that did not meet the criteria having three or four genetic markers. This resulted in two data set combinations, referred to as concatenated-xinsi-alignment (CONCATx) and concatenated-salsa-alignment (CONCATs).

For the three types of species delimitation analyses, we used the following data sets: *COI*-all, *ITS2*x-all, and *ITS2*s-all for TCS; *COI*-unique, *ITS2*s-unique, and *ITS2*x-unique for GMYC [40, 41]; the concatenated alignment of *COI*-all and *ITS2*s-all, keeping all specimens with both *COI* and *ITS2* data present, resulting in a data set with 351 *Terebellides* specimens (Table 2, 5th column) for STACEY [42].

### Model selection

Best-fit models for phylogenetic analyses were selected using the Akaike information criterion in JModel [43]. The protein coding gene *COI* was divided into two partitions, one with the first and second codon positions, and one with the third codon positions. In the general phylogeny of *Terebellides*, *ITS2* and the neighboring *28S rDNA* were combined into a single partition.

### Phylogenetic analyses

Mitochondrial (*COI* and *16S rDNA*) and nuclear data sets (*ITS2* and *28S rDNA*) were analyzed separately and combined using Bayesian inference (BI), and Maximum Likelihood (ML). This means five different analyses per method; 1) mitochondrial data alone, 2) nuclear data alone with *28S rDNA* combined with xinsi-, or 3) salsa-aligned *ITS2* sequences, and 4) mitochondrial data combined with nuclear data with *28S rDNA* combined with xinsi-, or 5) salsa-aligned *ITS2* sequences (S8 and S9 Appendixes). Bayesian analyses of separate and combined data sets were run in MrBayes version 3.2 [44]. Partitions were unlinked for the parameters statefreq, revmat, shape and pinvar. Rateprior for the partition rate multiplier was set to be variable. Two independent analyses were run for 10 million generations, with four parallel chains (three hot, one cold), that were sampled every 1000th generation. One fourth of the samples was discarded as burn-in. Maximum likelihood analyses were performed in raxmlGUI [45]. In RAxML, we used the same partitioning as in MrBayes, and node support was assessed with 1000 bootstrap replicates.

### Species delimitation analyses

Minimum spanning haplotype networks were constructed with the software program TCS 1.2.1, using a 95% connection limit with gaps = missing. The General Mixed Yule Coalescent model (GMYC) uses a likelihood ratio test to compare a null model assuming a single coalescent branching rate across a clock-like tree (i.e. intraspecific population events) with a complex model including both coalescent and Yule (interspecific diversification events) branching rate models. The later also estimates the threshold time that maximizes the transition between coalescent and Yule branching models, and hence delimiting species boundaries. Species delimitation with the GMYC algorithm was performed with the R library splits v.1.0–19 [46] using a single threshold and the required R packages ape, paran, and MASS. Ultrametric trees for species delimitation using GMYC algorithm were built in BEAST v1.8.2 [47] setting a nucleotide substitution rate for COI with a prior with log-normal distribution (log mean -4.466, standard deviation 0.075). This rate of 2.2% per my (95% interval 2.0–2.6%) is close the rate of 2.3% estimated by Brower [48] and widely implemented by many studies. Alternation of the GMYC algorithm permit to assess whether the branch leading to a node contains a threshold from coalescence to speciation under different coalescent models [41]. A node support value of 1 means that all coalescent models tested support the existence of a speciation event on that branch, and lower supports indicate that fewer coalescent models support such a speciation event. The number of species and so species limits would be influenced by the support cut-off selected. With lower cut-off value, the number of species will be more similar to the raw species delimitation estimated by GMYC algorithm without taking into account the support. On the other hand, higher cut-off values would reduce the number of species, generally merging closely related GMYC entities (species). We selected an arbitrary, but high, GMYC support value cut-off (0.9) to ensure that remaining species are discovered by GMYC algorithms (i.e. supported) under most of the different coalescent models tested (90%). The optimal cut-off value should be validated by simulation studies and with several empirical datasets but this is beyond the scope of our study. STACEY is a phylogenetic and a species delimitation method under a multispecies coalescent method (i.e. find the species tree and delimit species but allowing different coalescent gene trees and coalescent times). STACEY v. 1.2.0 analyses were run in BEAST2 v2.4.3 [49].

### Haplotype analyses, genetic distances, maps and distribution analysis

Haplotype networks were constructed using the TCS network inference method with a 95% connection limit, and gaps treated as uninformative. Each individual network was plotted in PopART [50] including distribution information according to the geographic areas designated. Uncorrected p-distances, with gaps treated as uninformative, were calculated in PAUP*4.0b10 [51], and Microsoft Excel v. 14.7.3. Distribution maps were compiled using ArcGIS 10.4.1 software package [52]. The geographic coordinate system GCS Sphere with Azimuthal Equidistant projection is used. Seafloor topography is accounted by the layer Etopo2. This is based on a global two minute gridded relief of ocean areas (ETOPO2v2, 2006) and provided by the National Oceanic and Atmospheric Administration (NOAA) of the U.S. Department of Commerce [53]. Bathymetric range, and clade composition for each biogeographic area, were analyzed and visualized using Microsoft Excel and Powerpoint for Mac 2011, version 14.7.3. Final design was completed in Adobe Photoshops Elements 12.0.

### Morphological analysis

The aim of the morphological work in the present study was primarily to identify our species to available species names, and to allocate these available names to the correct clade circumscribed by the molecular analysis. The detailed morphological analyses of new species derived from this study will appear in forthcoming papers.

## Results

### Model selection

The selected best-fit models were a general time reversible model with a proportion of invariable sites and gamma distributed rate across sites (GTR+I+G) for the partitions *16S rDNA*, *ITS2*, and *ITS2* combined with *28S rDNA*, and *COI*-partition with third codon sites only, while a general time reversible model with a proportion of the sites invariable (GTR+I) was selected for the *COI*-partition including first and second codon positions. In RAxML, the analyses were run with an independent GTRGAMMAI model for each partition, as the program do not allow the assignment of more than one model to different partitions.

### Phylogenetic analyses

**Fig 5:**
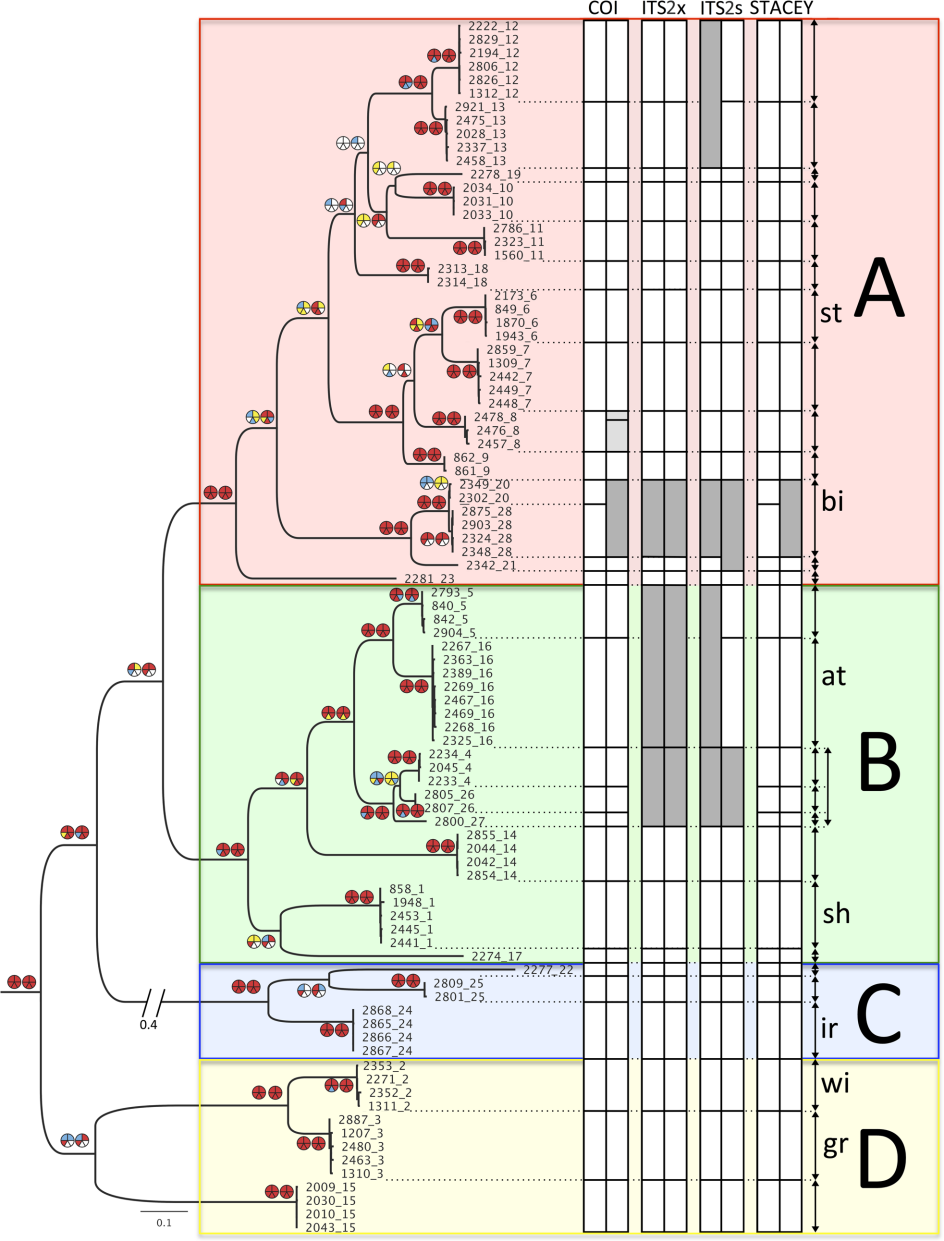
Results from the phylogenetic analyses, summarized on the ML estimate of the combined data set with xinsi-aligned ITS2-sequences including 91 terminals. Specimens are named according to the extraction-number and the appended clade-number. The phylogenetic tree is arbitrarily divided into four colour-coded groups, A–D. These colours are used as background colour in the distribution and haplotype network figures (Figs 6–8). Specimens with at least three of the genetic markers were included in the phylogenetic analyses, outgroups are not shown. Pie diagrams indicate support values for the node, left pie shows results from ML analyses, and right pie diagram results from Bayesian analyses. Upper two slices of a pie illustrate results from the combined data sets' two different alignments, with xinsi-aligned ITS2-sequences to the left, and salsa-aligned ITS2-sequences to the right. The three remaining slices illustrate results from the combined mitochondrial data (lower left slice), and the combined nuclear data sets' two different alignments, where lower median slice has xinsi-aligned ITS2-sequences, and lower right slice has salsa-aligned ITS2-sequences. Yellow, blue and red colour indicate low, moderate and strong support, which equals ML support in the intervals 50–74, 75–89, and 90–100, or BI posterior probabilities in the intervals 0.50–0.84, 0.85–0.94 and 0.95–1.0 respectively. White means support <50/0.50 for the node. Columns show clustering of terminals according to different methodologies performed on more inclusive data sets where all specimens with COI or ITS2 data, or specimens with both COI and ITS2 data, were included. The first columns under the headings COI, ITSx and ITSs represent the results from TCS, and the second columns represent the results from GMYC. The columns under the heading STACEY show the two different outcomes from this analysis. White means that the network or species recovered is identical to the initial haplotype network found in COI including all COI-sequences, light grey means that less inclusive networks or putative species were recovered, and dark grey means that a more inclusive network or putative species was recovered. Double-headed arrows to the right of the columns show our final judgement of species delimitation. The two small letters to the right indicate our designation of described species, st = *T*. *stroemii*, bi = *T*. *bigeniculatus*, at = *T*. *atlantis*, sh = *T*. *shetlandica*, ir = *T*. *irinae*, wi = *T*. *williamsae*, and gr = *T*. *gracilis*.

The combined data set of the two different combinations of *COI*, *16S rDNA*, *ITS2* and *28S rDNA* (CONCATx and CONCATs) consisted of 2574/2474 aligned positions, of which 993/1023 were parsimony-informative, and 172/171 were variable but not parsimony-informative. The results from the separate and combined analyses are summarized on the ML-tree from CONCATx (Fig 5). The phylogenetic tree is arbitrarily divided into four major groups, A–D, to make the presentation of the results more perspicuous. The results from each analysis (S10–S19 Appendixes), are presented in pie diagrams next to each node (Fig 5). The different analyses show high level of congruence between methods (ML or BI), alignment treatment (CONCATx or CONCATs), and data set combinations (mitochondrial, nuclear or combined). Out of the 49 nodes in Fig 5, 35 are identical among all five different analyses. There are few conflicting nodes between the topologies, most of them are related to the arrangement within group A, and most of them have low node support and therefore cannot be interpreted as incongruences. However, the analyses have recovered four well supported clades different to the topology illustrated in Fig 5: 1) clades 11 and 19 (group A) are sister taxa with BI-support of 0.97, in the separate nuclear data set with salsa-aligned ITS2 sequences (S13 Appendix); 2) clade 18 (group A) is sister taxa to a clade with 11, 12, 13, 19, 20, and 21 with BI-support of 0.95 in the separate nuclear data set with salsa-aligned ITS2 sequences (S13 Appendix); 3) clade 17 (group B) is sister taxa to a clade with 1, 4, 5, 14, 16, 26, and 27, with 0.98 in BI-support and 78 in ML-support, in the separate nuclear data set with xinsi-aligned ITS2 sequences (S14 and S15 Appendixes); 4) clades 24 and 25 (group C) are sister taxa, with 0.93/1.0 in BI-support, and 70/95 in ML-support in both separate nuclear data sets (with xinsi- or salsa-aligned *ITS2* sequences) (S12–S15 Appendixes).

### Species delimitation analyses: TCS, GMYC and STACEY

The statistical parsimony analysis of the *COI* data set, rendered 28 separate haplotype networks, while TCS analyses of *ITS2*x and *ITS2*s resulted in 24 and 23 networks respectively (S20–S22 Appendixes). GMYC analysis of the *COI* data set rendered 28 putative species, and GMYC of *ITS2*x and *ITS2*s resulted both in 24 putative species (S23–S31 Appendixes). In STACEY we treated the 28 haplotype networks from the *COI* data as the species to be tested, and in 98.8% of the resulting trees, all of these 28 clades were recovered and in 1.2% of the trees, clades 20 and 28 were lumped together (S32 Appendix) (see Fig 5). We used the most inclusive data sets for each species delimitation analyses, and in TCS all sequences of *COI* (n = 462) and *ITS2* (n = 402) were included, in GMYC all unique sequences of *COI* (n = 271) and *ITS2* (n = 136) were included, while all terminals with both *COI* and *ITS2*-data (n = 351) were included in STACEY.

The outcomes from the TCS, GMYC and STACEY analyses are identical for 17 of the 28 putative species, namely clades 1, 2, 3, 6, 7, 9, 10, 11, 14, 15, 17, 18, 19, 22, 23, 24, and 25. Looking at the instances where there is disagreement among methods, and starting with group A, clades 12 and 13 are separate in all analyses except for TCS on ITSs, where the haplotypes are connected into a single haplotype network, with the closest haplotypes for clades 12 and 13 separated by eight mutations (connection limit = nine). Clade 8 is further divided in the GMYC-analysis of COI where a group with six haplotypes (1197_8, 1198_8, 1999_5, 2013_8, 2014_8, 2214_8) is found as a separate putative species. The closest haplotype of this group is seven mutations from the closest haplotype in the main group of clade 8 in the minimum-spanning haplotype network from the TCS-analysis on the same data. Clades 20 and 28 are connected in the GMYC-analysis of COI. The closest haplotypes for these clades are separated by 16 mutations in the minimum-spanning haplotype network from the TCS-analysis (using a fixed connection limit) on the same data. Clades 20 and 28 share the same haplotype in *ITS2*, and are thus connected in all analyses on *ITS2*; this haplotype is also connected to clade 21 in the GMYC-analysis of *ITS2*s. Haplotypes of clades 21 and 20/28 are separated by 11 mutations in the minimum-spanning haplotype network from the TCS-analysis (using a fixed connection limit) on the same data. Continuing with group B, clades 5 and 16 are connected in the TCS-analyses of *ITS2*x and *ITS2*s (where the closest haplotypes of clades 5 and 16 are separated by 6 and 5 mutations; connection limit = 9), as well as in the GMYC-analysis of ITSx. Clades 4, 26 and 27, all represented by single haplotypes in *ITS2*, are connected in all four analyses of the *ITS2*-data. The haplotypes are separated by between three and eight mutations in the minimum-spanning haplotype network in the two TCS-analyses.

**Fig 6:**
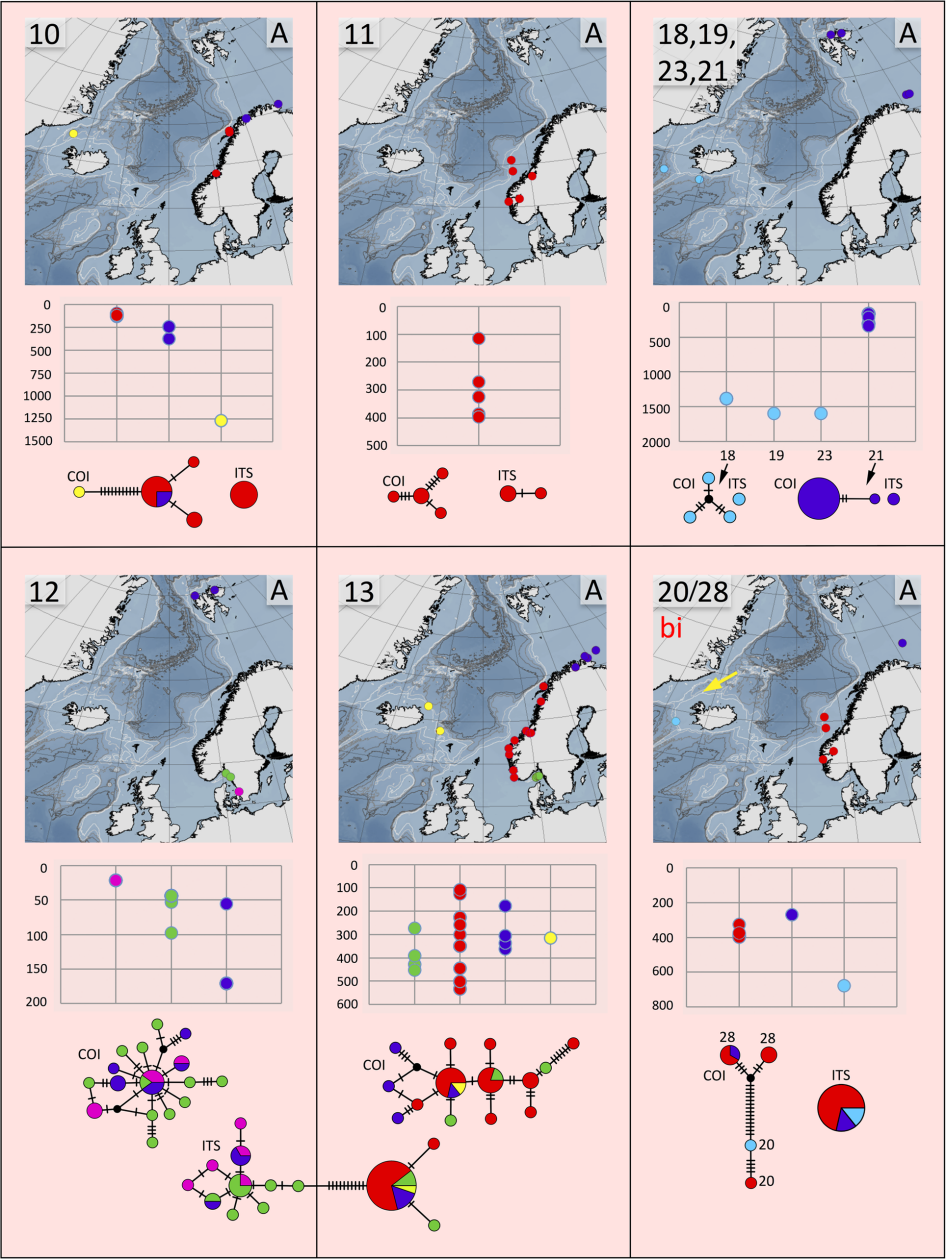
Distribution maps, depth distribution in meters, and haplotype networks for group A, species 10, 11, 18, 19, 23, 21, 12, 13, and 20/28. All species except for species 20/28 that we refer to *T*. *bigeniculatus* (bi) are undescribed. Sites are colour coded as in Fig 3. Type locality for *T*. *bigeniculatus* indicated with yellow arrow.

**Fig 7:**
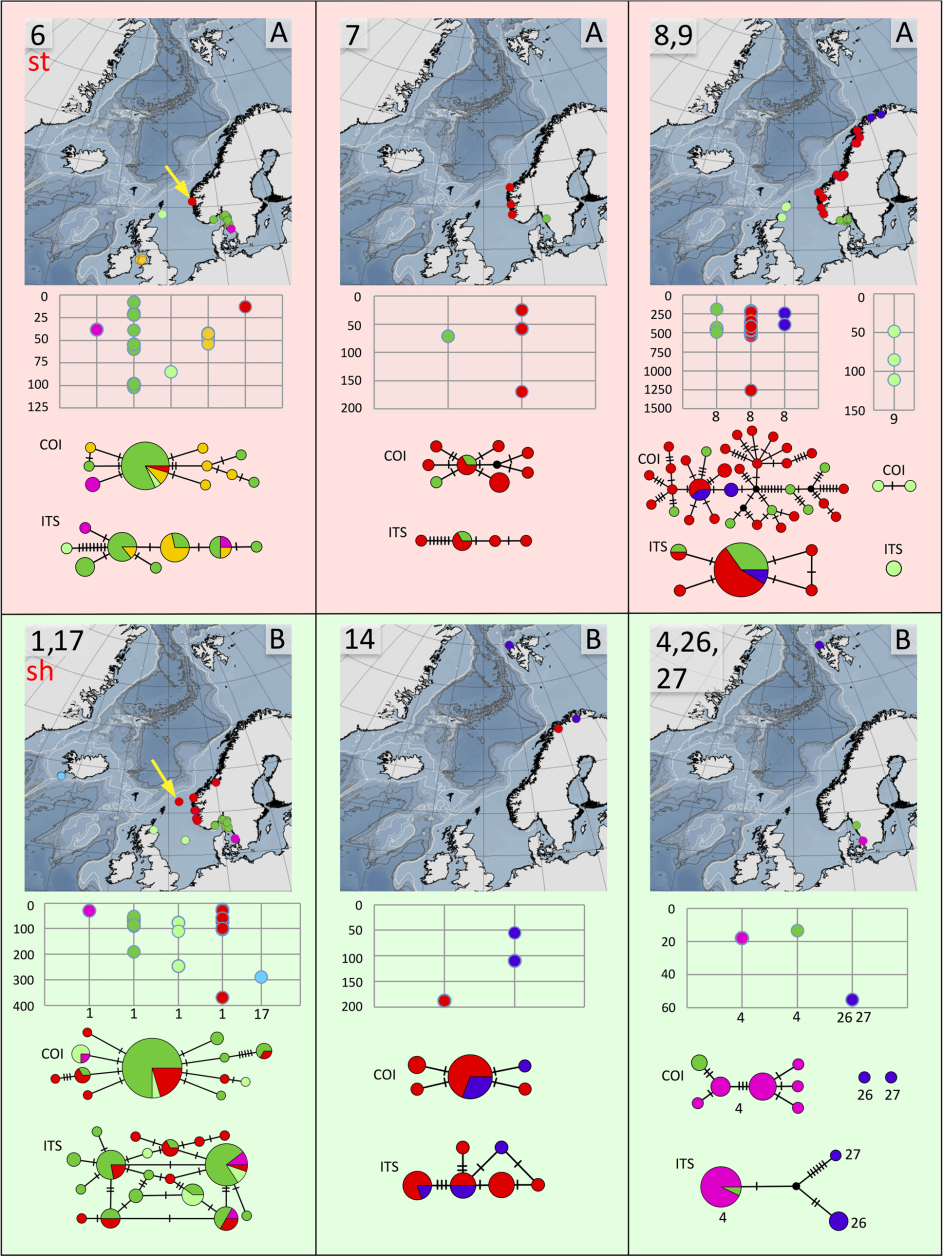
Distribution maps, depth distribution in meters, and haplotype networks for group A, species 6, 7, 8, and 9, and for group B, species 1, 17, 14, 4, 26, and 27. All species except for species 6 that we refer to as *T*. *stroemii* (st), and clade 1 that we refer to as *T*. *shetlandica* (sh) are undescribed. Sites are colour coded as in Fig 3. Type localities for *T*. *stroemii*, and *T*. *shetlandica* indicated with yellow arrows.

**Fig 8:**
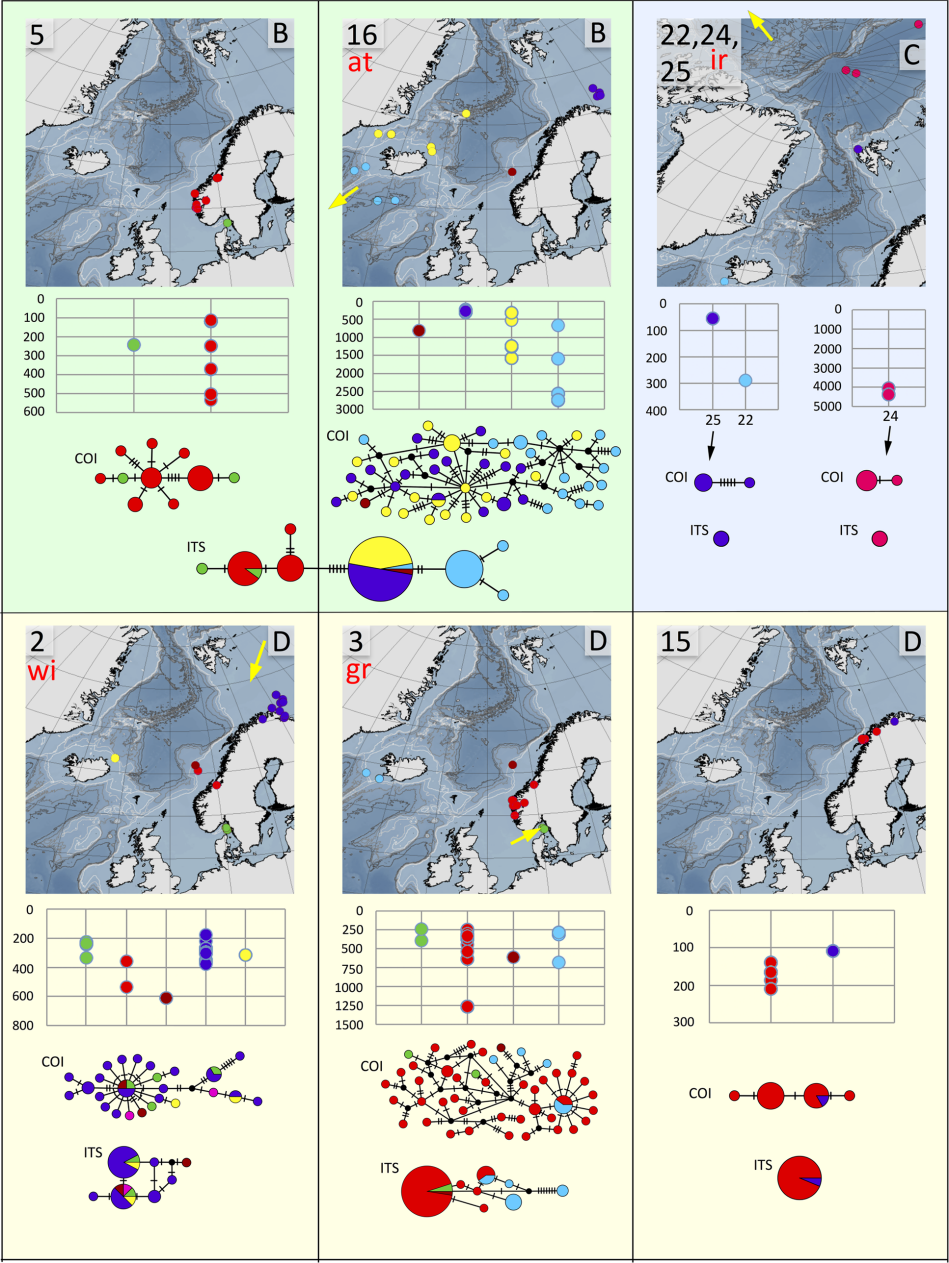
Distribution maps, depth distribution in meters, and haplotype networks for group B, species 5 and 16, group C, species 22, 24, and 25, and group D, species 2, 3, and 15. Species 5, 22, 25, and 15 are undescribed, while we refer species 16 to *T*. *atlantis* (at), species 24 to *T*. *irinae* (ir), species 2 to *T*. *williamsae* (wi), and species 3 to *T*. *gracilis* (gr). Sites are colour coded as in Fig 3. Type localities for *T*. *atlantis*, *T*. *irinae*, *T*. *williamsae*, and *T*. *gracilis* indicated with yellow arrows.

In summary, we suggest that clades 12 and 13 represent different species even though they are connected in one of the *ITS2*-analyses. The two clades do not share any *ITS2*-haplotypes (Fig 6), and both lineages are fairly well sampled with 23 (clade 12) and 27 specimens (clade 13). There are also insertion/deletion events in the ITS2-sequence alignments that support the two clades, however, in the analyses presented here, we treated indels as missing data. We further conclude that the separate putative species in clade 8 found in the GMYC-analysis of *COI*-data could be ignored as intraspecific genetic variation (only seven mutations in the TCS-analysis), and there is neither any differences in the ITS2-data to support such a conclusion. We do think that there is evidence that clades 20 and 28 should be regarded as the same species even though they have separate haplotype networks in the TCS-analysis on the *COI*-data, both lineages are under-sampled with only two (clade 20) and five specimens (clade 28), and the difference between the lineages is within the variation that is found in better sampled clades (compare clades 20 and 28 in Fig 6 with clade 8 in Fig 7, and clade 16 in Fig 8), and there is a good chance that the haplotypes would be connected given a larger sample size. *ITS2*-data also support this conclusion as clades 20 and 28 share the same *ITS2*-haplotype (Fig 6). Results from STACEY also give some support to this deduction. In contrast, we believe that it is likely that clade 21 represents a separate species even though it is connected with clade 20/28 in the GMYC of the *ITS2*s, differences in *COI* between 20/28 and 21 is substantial (12.1–13.2%) (Table 3), and there is also additional indel events in the *ITS2*-data alignment that suggests that they do represent different species. As was the case for clades 12 and 13, we also strongly argue that clades 5 and 16 represent different species, even though they are connected in three of the four ITS-analyses. The two clades do not share any *ITS2*-haplotypes (Fig 8), and there are also indel events and morphological data (see below) supporting their separation. Finally, clades 4, 26, and 27 is suggested to represent different species, but the lineages are poorly sampled both in numbers and in geographic distribution, and more specimens are needed. However, *COI*-differences (13,3%) as well as *ITS2*-differences (Fig 7) in the two sympatric clades 26, 27 is comparable to the differences found between other closely related species pairs in the species complex, but as only 1 (clade 26) and 2 specimens (clade 27) were found of these clades, we are less certain in this case.

To conclude, we think we have strong evidence that we have between 25 and 27 different species of *Terebellides* among the sequenced specimens. In the analyses and discussion below we will proceed with the 27 species hypothesis, and the species will be referred to as species 1, 2 etc. following the original clade numbering, until the available names can be allocated to their proper clades. The clades 20 and 28 will be referred to as species 20/28.

### Biogeographic and bathymetric analyses

**Fig 9:**
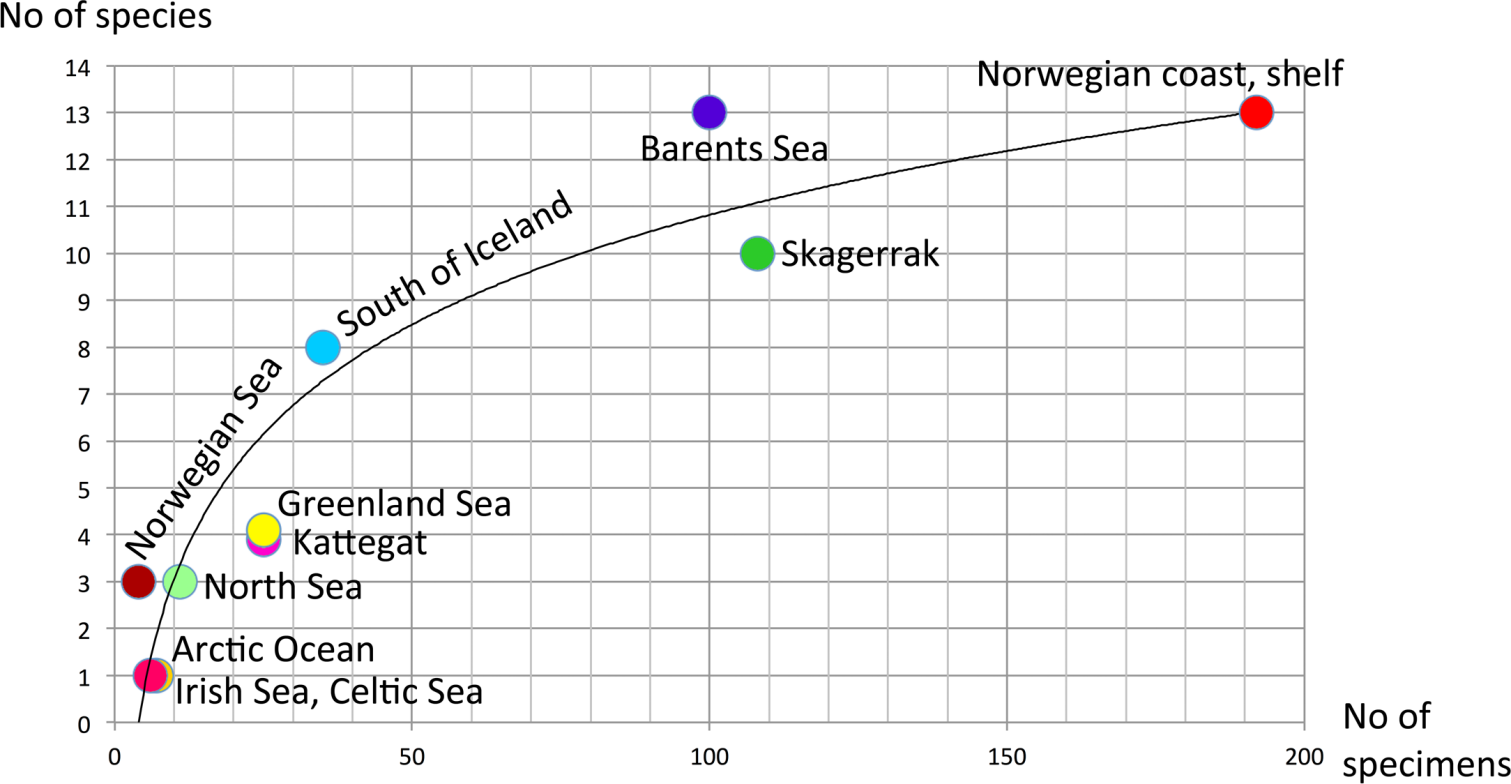
Accumulation curve showing the relationship between sampling size (number of specimens) and number of species found among the different biogeographic regions.

**Fig 10:**
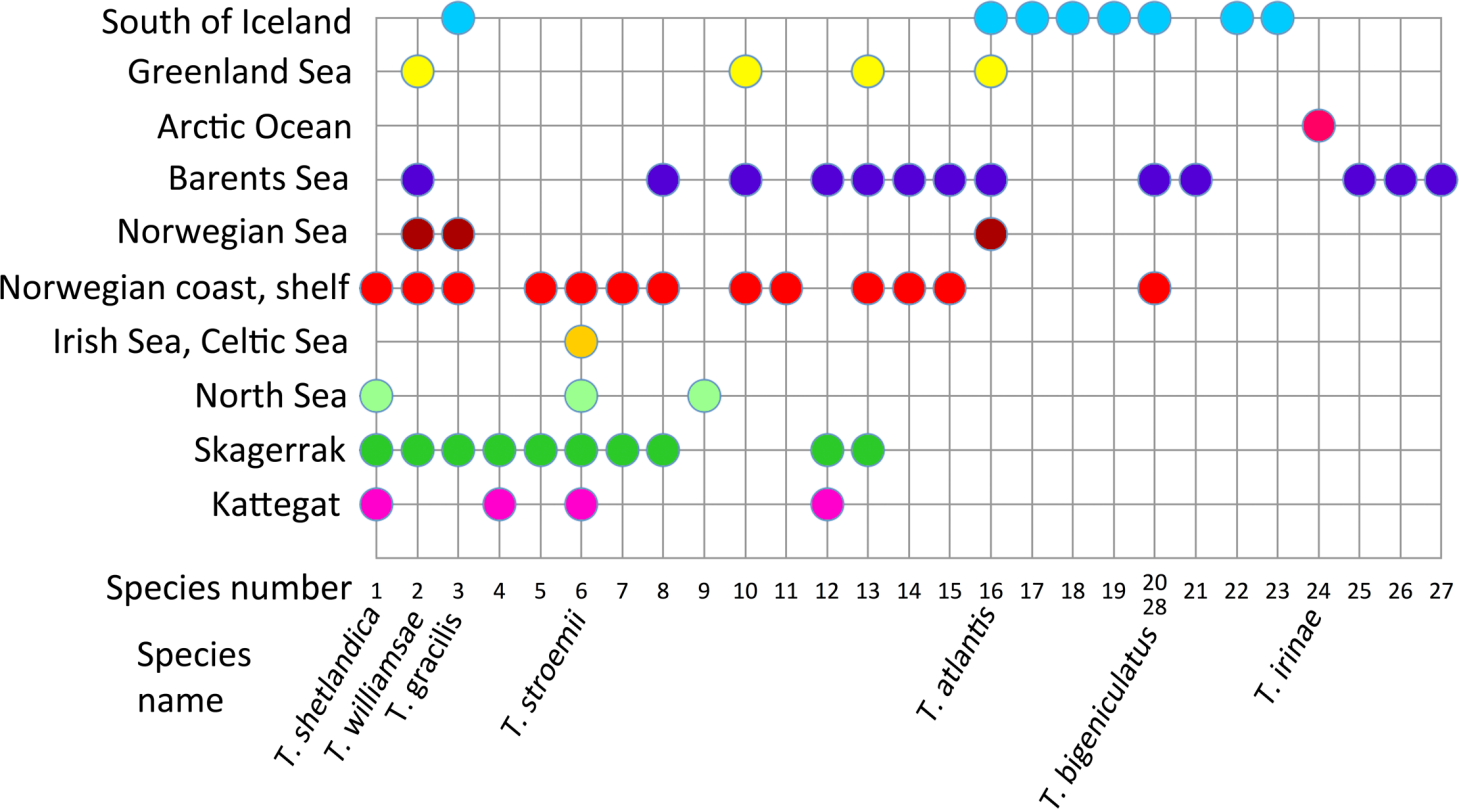
Diagram showing the distribution of different *Terebellides* species in the ten biogeographic regions.

**Fig 11:**
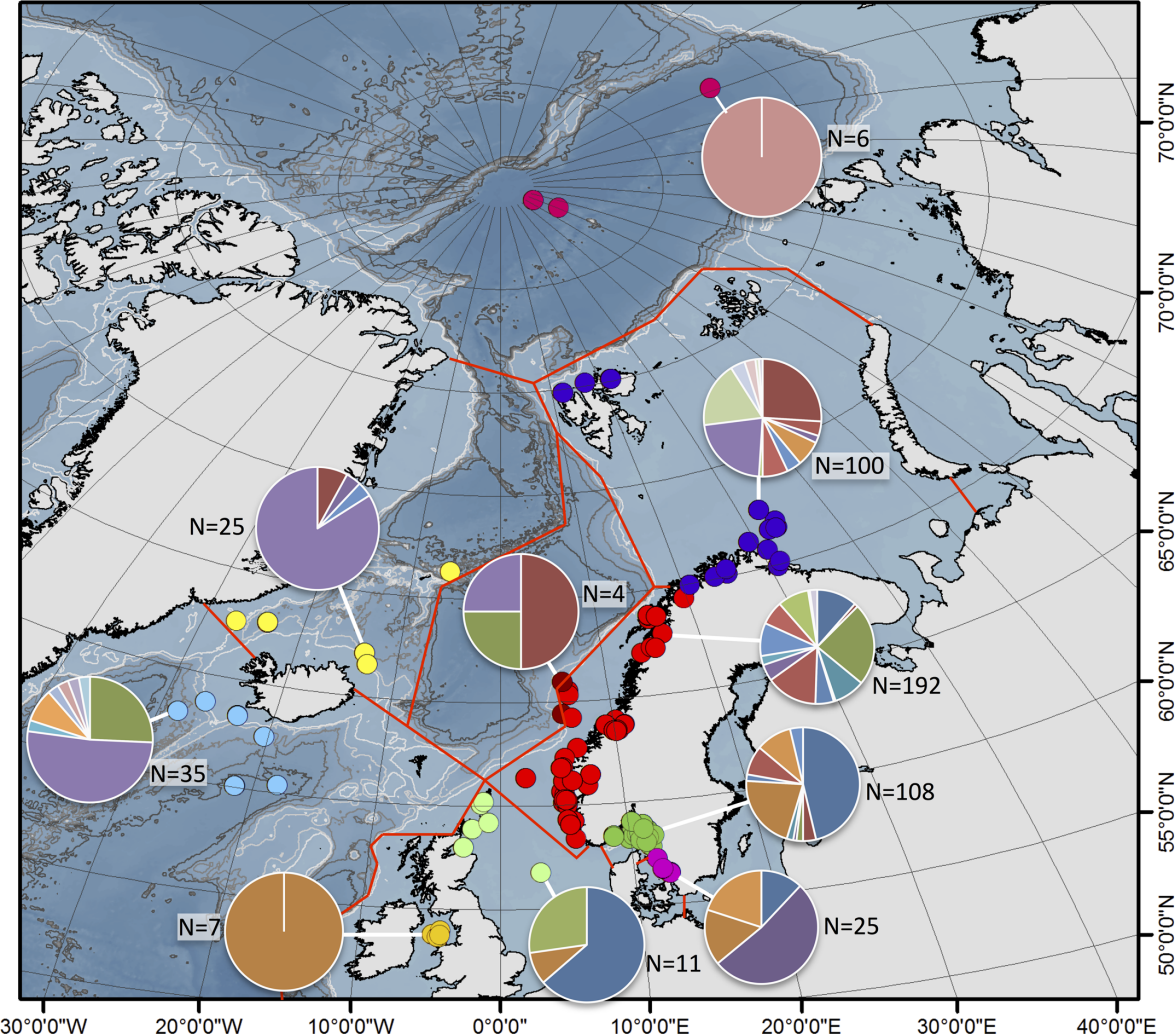
Overview of the diversity found in the ten biogeographic regions. Pie diagrams show the relative proportions of the different species found where all species have their own colour, sampling size (N) indicated next to the pie diagrams.

The number of species varied rather much between the biogeographic regions (Figs 9–11). However, as the study was not designed to assess the differences in diversity for different areas, we cannot answer if certain areas are more diverse than others. Instead, the number of species strongly correlates with how many specimens that are sequenced (Fig 9), and this probably explain much of the differences found in diversity among areas. Some sort of saturation in discovering new species seems to be reached at about 100 sequenced specimens for a biogeographic area. We found more than one species in all biogeographic regions except for the two most poorly sampled regions, Arctic Ocean, and Irish and Celtic Seas (Fig 11), while the highest diversity was found in the best sampled regions with 13 species among 192 specimens in the Norwegian coast and shelf area, 13 species among 100 specimens in Barents Sea, and 10 species among 108 specimens in Skagerrak (Figs 9 and 10).

With regard to similarity in shared *Terebellides* species between the different biogeographic regions the following may be assumed (Fig 10), Kattegat is most similar to Skagerrak, with four out of its four species in common; Skagerrak is most similar to Norwegian coast and shelf, with eight out of its 10 species in common; North Sea is most similar to either Skagerrak, or Norwegian coast and shelf, with two out of its three species in common; the single species found in Irish and Celtic Sea is also present at the Norwegian coast and shelf, North Sea, Skagerrak and Kattegat; Norwegian coast and shelf is most similar to Skagerrak, with eight out of its 14 species in common; Norwegian Sea is most similar to either Skagerrak, Norwegian coast and shelf, Barents Sea, or Greenland Sea, with two out of its three species in common; the single species found in the Arctic Ocean is endemic for the area; Greenland Sea is most similar to Barents Sea, with four out of its four species in common; and the area South of Iceland is most similar to either Norwegian Sea, or Norwegian coast and shelf, with two out of its eight species in common. Endemic species are found in the Arctic Ocean (species 24), North Sea (species 9), Norwegian coast and shelf (species 11), Barents Sea (species 21, 25, 26, and 27), and in the area South of Iceland (species 17, 18, 19, 22 and 23).

**Fig 12:**
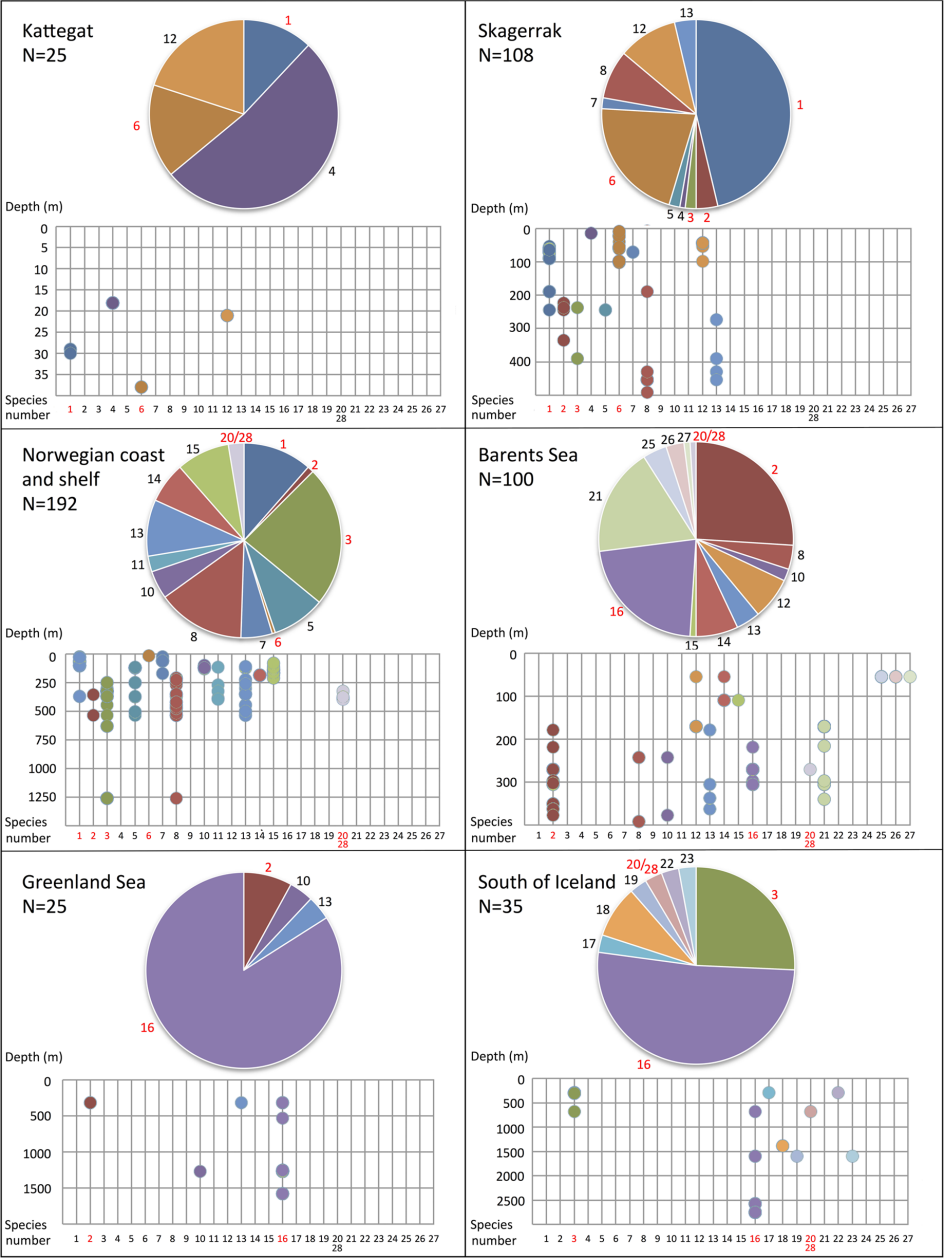
Pie diagrams from Fig 11 for the six best sampled biogeographic regions with bathymetric results in meters. Species number next to its slice in the pie diagrams, species number in red when the species is a described species, where species 1 = *T*. *shetlandica*, species 2 = *T*. *williamsae*, species 3 = *T*. *gracilis*, species 6 = *T*. *stroemii*, species 16 = *T*. *atlantis*, and species 20/28 = *T*. *bigeniculatus*.

Many of the species that that were found in the same biogeographic regions also overlapped in their bathymetric distribution (Fig 12). Yet, there is some sort of division between some of the species, e.g. species 6 and 7 are found down to about 200 meters depth, while the closely related species 8 is found below 200 meters depth. Within the same biogeographic area, up to eight different species can be found in a depth span of 100 meters, and even in the same sample from a supposedly homogenous environment from a mud bottom from 534 meters depth, in the Trondheimsfjord in Norway, using a Sneli sledge, up to five different species were found (see Table 1, siteID NCS24). We can safely conclude that a majority of the species live in sympatry with several other species in the complex.

### Haplotype and distance analyses

Distance calculations (S33–S34 Appendixes), uncorrected, are summarized in Tables 3 and 4, as are the results from the haplotype analyses. The latter are also visualized in Figs 6–8 for all different species. For most species, haplotypes, or group of closely related haplotypes, are generally not restricted to a certain area. A few species show a week tendency towards geographic sorting, e.g. in species 16 (Fig 8), the haplotypes from the area South of Iceland (light blue) may to some extent be interpreted in this way. Haplotype diversity is generally high, and in a few of the well sampled species it is extreme. In species 2 there are 25 haplotypes among 32 specimens, in species 3 there are 44 haplotypes among 50 specimens, in species 8 there are 33 haplotypes among 40 specimens, and in species 16 there are 48 haplotypes among 55 specimens.

### Morphological analyses

Group A comprises 13 species. For the time being we are not able to find any morphological character that unites the group, but two of the known species, *T*. *bigeniculatus* and *T*. *stroemii*, can be attributed to two of the clades found. *Terebellides bigeniculatus* is identified by the presence of geniculate chaetae (Figs 1B, 2A and 2C–2E) in both chaetigers 5 and 6, and this condition is found in species 21 and species 20/28, and as the latter of these two species is the only one found among our Icelandic specimens we suggest that the name *T*. *bigeniculatus*, that has its type locality north-west of Iceland, may be used for species 20/28. *Terebellides stroemii* on the other hand is characterized by a robust body, and relatively small branchiae, with partially fused lobes (Fig 1B) instead of unfused ones (Figs 1A and 2B). From the available diagnosis, any of the clades 6, 7, 8 and 9 are possible candidates for representing the true *T*. *stroemii*. *Terebellides stroemii* has a type locality from between 55–110 meters depth near Bergen in SW Norway, and species 8 is only found deeper than 200 meters and is thus excluded for being the nominal species, and with the same reasoning, we also exclude species 9 due to that it is only found in the North Sea region. However in the choice between species 6 and 7 we cannot say right now which one is more likely to be the correct *T*. *stroemii*, but our suggestion is that clade 6 could be used for the name, because in our samples it seems to be the most common and widely spread species of the two.

Group B comprises eight species. A possible morphological identifier for this group of species is that they all have small to medium sized, elongated bodies. In this group, two clades, 16 and 1, could be identified as already described taxa. Species 16 is characterized by having unfused branchial lobes, with a low number of lamellae. Due to this, and because of its distribution, found at great depths in Greenland Sea and in the area South of Iceland, congruent with the species original depth distribution, we suggest that the name *T*. *atlantis* might be applicable for this species. Species 1 should be referred to *T*. *shetlandica*, it is the only species we have found that have the characteristic gills with branchial lobes of different sizes, and provided with a long posterior filament (Fig 2B), diagnostic features for *T*. *shetlandica*. Moreover, in some specimens a parasitic copepod was found, as was also described for several specimens of *T*. *shetlandica* in the original description.

Group C comprises three species, with no apparent morphological identifier. We attribute the name *T*. *irinae* to the deep-water species 24 found only in the Arctic Ocean in our analysis. It fits the original description well, and even if our collecting sites are not near the type locality we think a distribution from Beaufort Sea to the Arctic Basin is likely.

Group D also comprises three species. The group is characterized by white ventral colouration in anterior thoracic chaetigers (1 to 4) (Fig 1A). Species 2 and 3 are further characterized by having branchiae with ventral and dorsal lobes of similar shape (Fig 2A). The combination of these characters fits the diagnosis of two already described species of *Terebellides*, *T*. *gracilis* and *T*. *williamsae*. *Terebellides williamsae* is considered to be a junior synonym to *T*. *gracilis* but we prefer to withdraw it from synonymy even though, at this moment, we do not have any morphological characters that separates them. Species 2 is suggested to represent *T*. *williamsae* as it is the only one of the two occurring in the Barents Sea (the type locality for *T*. *williamsae*), and thus species 3 is suggested to represent *T*. *gracilis* even though both species 2 and 3 are found in sympatry at the type locality for *T*. *gracilis*, the Swedish coast of Skagerrak.

## Discussion

Cryptic species are of paramount importance because of their commonality, and they are routinely found in genetic surveys, also in well-known taxa in well-studied areas [2, 54]. It is clear that the small fraction of morphological species that has been investigated so far still only represents the tip of the iceberg as Knowlton stated in her visionary paper on sibling species almost 25 years ago [55]. Considering that cryptic species literally are everywhere, in a taxonomic as well as in a geographic context, they can in no way be neglected if we want to correctly assess species diversity, understand biogeographic patterns or keep track of natural or man-made induced changes in the marine environment.

*Terebellides* is one of the most regularly encountered annelid taxa in environmental monitoring programs in the North East Atlantic [56], and it is normally reported under the species names *T*. *gracilis*, and *T*. *stroemii*, and in recent years *T*. *shetlandica* and *T*. *bigeniculatus* have been added to the list. Prior to this study, we suspected there might be cryptic species hiding among *Terebellides*, but it came as an overwhelming surprise to find so many of them, and that in cases some of them were so common.

Having a closer look at the best sampled areas (Fig 12), starting with Kattegat and Skagerrak, a very small part of the North East Atlantic. There are so far three different species reported from this area, and we have identified these in our sequenced specimens; *T*. *stroemii* (species 6), *T*. *gracilis* (species 3), and *T*. *shetlandica* (species 1). In addition we have a new record of *T*. *williamsae* (species 2), but the remainder, that is species 4, 5, 7, 8, 12 and 13 are unknown and undescribed. Out of the 133 specimens sampled and sequenced from the area, about roughly 1/3 (47 specimens) belong to this latter category of undescribed species. Continuing with the Norwegian coast and shelf, we find the same four described species present in Kattegat/Skagerrak, and in addition *T*. *bigeniculatus* (species 20/28), but species 5, 7, 8, 10, 11, 13, 14, and 15 are all undescribed. These unnamed species gather more than half of the specimens sequenced (117 out of 192 = 61%), and that means in short, that the current probability of finding an undescribed species of *Terebellides* is larger than finding a described one! This is indeed astonishing given that we are dealing with one of the best investigated marine environments in the world, the relatively shallow waters just outside the coasts of Sweden and Norway, and one of the most frequently encountered annelid taxa in the area. The situation in the Barents Sea is similar, and we find *T*. *williamsae* (species 2), *T*. *atlantis* (species 16) and *T*. *bigeniculatus* (species 20/28), but neither *T*. *stroemii* (species 6) nor *T*. *gracilis* (species 3) among our sequenced specimens; in addition we also find the undescribed species 8, 10, 12, 13, 14, 15, 21, 25, 26, and 27, and together these undescribed species represent c. 50% of the sequenced specimens. Greenland Sea and the area South of Iceland are dominated by specimens of *T*. *atlantis* (species 16), *T*. *gracilis* (species 3), and *T*. *williamsae* (species 2), but there are also quite a few undescribed species present here as well, but as the sample size is not as large as in Skagerrak, Norwegian coast and shelf, and Barents Sea the results are not really comparable.

Looking at the depth distribution for the different species in a given geographic area, we can see that most species overlap in depth, and there is, in most cases, no clear sorting of species at different depths (Fig 12). In the depth range 150–250 meters in the Norwegian coast and shelf region, we have nine species present, and they all more or less overlap and are present at most of the localities that we have been able to sample, e.g. in the area from Trondheim in the north to Bergen in the south, 11 species are found (Figs 6–8), indicating that they do not inhabit specific habitats like fjords or the open ocean. For most of our samples we have used a sledge, a dredge or a beam trawl, all these gears sample material from an unspecified area of the sea floor. But often, at least when a sledge is used, the area sampled is an apparently flat uniform habitat of mud. In 49 out of the 89 sites from where we have sequenced more than one specimen, we found more than one species (Table 1), and in the most species-rich samples, five different species of *Terebellides* were found, e.g. site NCS24, a sample from a flat mud bottom from 534 meters depth in the Trondheimsfjord. There are few samples taken with a grab, but in one of them (BS5) we found two species co-occurring. Anyway, as we did not sequence all specimens from all samples, it is difficult to assess how many species of *Terebellides* that do co-occur at the same site. For many of the sites only one or a few specimens were sequenced, thus it is likely that diversity for each separate site is underestimated, but when looking at a slightly larger scale this should not be the case.

Apart from the fact that so many species of *Terebellides* still go under the radar, and that these unknown and undescribed species are so common, and even constitute a major part of the diversity both in number of species and specimens, one other thing struck us: the extreme diversity of haplotypes found in *COI* among some species. The most note-worthy are *T*. *gracilis* (species 3), *T*. *williamsae* (species 2), *T*. *atlantis* (species 16), and species 8, where almost all specimens sampled and sequenced have their own unique haplotype (Figs 7 and 8, Table 3). This variation rarely has led to an amino-acid substitution within the species, and in *T*. *atlantis* (species 16) all 48 haplotypes found among the 55 specimens produce the same exact amino-acid sequence. As the sample size varies a lot between different species, it is difficult to make a direct comparison in haplotype diversity, but one thing to note is that all those four species mentioned above are found at greater depths than a couple of 100 meters (Figs 7 and 8), in contrast with two other species that also are well represented in the material, i.e. *T*. *shetlandica* (species 1), and *T*. *stroemii* (species 6), that are found at more shallow depths (Fig 7). The relatively low genetic diversity among these shallow water species may be explained by that they have been more affected by the recurrent ice ages that have occurred during the last 1.8 million years [57], than the species living at greater depths have been. Even so it is hard to understand and explain the extremely high diversity of haplotypes, and how it is maintained, in these deeper-living species, but see [58] that also reported high haplotype diversity in *Aonidella cf dayi*, for possible explanations to this phenomenon.

Our angle on this study has been a molecular one, in order to find out how many species that occur in North East Atlantic waters, and the full morphological investigation has to await forthcoming studies. The main purpose of the morphological examination conducted in this paper has been to connect the described and known morphological species to the correct, or at least the best, molecularly recognized species. It is our hope that we in the future will be able to find morphological characters that will help in standard morphological identification down to at least a group of possible species, and in the best of worlds also down to species level. Molecular data from this study will be vital to help us to sort out when this latter task is obtainable and when it is not.

Much water has passed under the bridge since Holthe [59] published his book on Terebellomorpha in the North East Atlantic, when he discussed the supposed cosmopolitan distribution of *T*. *stroemii*. He acknowledged that the worldwide reports were due to a confusion of closely related species, but nevertheless stated that ‘I do not suspect that there are more than one species in the Norwegian material’. Still in these days, most *Terebellides* in the North East Atlantic are routinely identified as *T*. *stroemii*, and our comprehensive study make it clear that this is a severe underestimation of the true diversity among *Terebellides*. We do not think that *Terebellides* is an unusual example of cryptic species, on the contrary, when morphospecies are properly assessed molecularly, in terms of sampling strategy and number of specimens analyzed (e.g. [60]), it is commonplace to find more than one species, sometimes several, in the material. Already Grassle [61] asked the question ‘How common are cryptic polychaetes’ when she and her husband had discovered six cryptic species of *Capitella capitata* after an oil spill in West Falmouth, Massachusetts in September 1969 [62]; we think we now have taken a small step further towards the answer to this long-held question.

## Supporting information

S1 Appendix COI-unique.nex. Alignment, in nexus-format, with the unique 271 COI-sequences.
(NEX)

S2 Appendix COI-all.nex. Alignment, in nexus-format, with all 462 COI-sequences.
(NEX)

S3 Appendix ITS2x-unique.nex. Alignment, in nexus-format, with the unique 136 ITS2-sequences, aligned with X-INS-i in MAFFT.
(NEX)

S4 Appendix ITS2x-all.nex. Alignment, in nexus-format, with all 402 ITS2-sequences, aligned with X-INS-i in MAFFT.
(NEX)

S5 Appendix ITS2s-unique.nex. Alignment, in nexus-format, with the unique 136 ITS2-sequences, aligned with RNAsalsa.
(NEX)

S6 Appendix ITS2s-all.nex. Alignment, in nexus-format, with all 402 ITS2-sequences, aligned with RNAsalsa.
(NEX)

S7 Appendix COI_and_ITS2s.nex. Alignment, in nexus-format, including specimens with both COI and ITS2-data, used in the STACEY analysis.
(NEX)

S8 Appendix CONCATx.nex. Concatenated alignment, in nexus-format, of COI, 16S rDNA, ITS2s, and 28S rDNA, including specimens with data from three of the four genetic markers.
(NEX)

S9 Appendix CONCATs.nex. Concatenated alignment, in nexus-format of COI, 16S rDNA, ITS2x, 28S rDNA, including specimens with data from three of the four genetic markers.
(NEX)

S10 Appendix CONCATmito_ML.tre. Resulting tree with support values, using Maximum Likelihood on mitochondrial data only.
(TRE)

S11 Appendix CONCATmito_BI.tre. Resulting tree with support values, using Bayesian inference on mitochondrial data only.
(TRE)

S12 Appendix CONCATnucls_ML.tre. Resulting tree with support values, using Maximum Likelihood on nuclear data only, with salsa-aligned ITS2-sequences.
(TRE)

S13 Appendix CONCATnucls_BI.tre. Resulting tree with support values, using Bayesian inference on nuclear data only, with salsa-aligned ITS2-sequences.
(TRE)

S14 Appendix CONCATnuclx_ML.tre. Resulting tree with support values, using Maximum Likelihood on nuclear data only, with xinsi-aligned ITS2-sequences.
(TRE)

S15 Appendix CONCATnuclx_BI.tre. Resulting tree with support values, using Bayesian inference on nuclear data only, with xinsi-aligned ITS2-sequences.
(TRE)

S16 Appendix CONCATs_ML.tre. Resulting tree with support values, using Maximum Likelihood on the combined mitochondrial and nuclear data set, with salsa-aligned ITS2-sequences.
(TRE)

S17 Appendix CONCATs_BI.tre. Resulting tree with support values, using Bayesian inference on the combined mitochondrial and nuclear data set, with salsa-aligned ITS2-sequences.
(TRE)

S18 Appendix CONCATx_ML.tre. Resulting tree with support values, using Maximum Likelihood on the combined mitochondrial and nuclear data set, with xinsi-aligned ITS2-sequences.
(TRE)

S19 Appendix CONCATx_BI.tre. Resulting tree with support values, using Bayesian inference on the combined mitochondrial and nuclear data set, with xinsi-aligned ITS2-sequences.
(TRE)

S20 Appendix COI_TCS_log.rtf. Log-file from the TCS-analysis on COI-all.
(RTF)

S21 Appendix ITS2x_TCS_log.rtf. Log-file from the TCS-analysis on ITS2x-all.
(RTF)

S22 Appendix ITS2s_TCS_log.rtf. Log-file from the TCS-analysis on ITS2s-all.
(RTF)

S23 Appendix COI_GMYC_code_nodes.pdf. Topology from the GMYC-analysis on COI-unique with node numbers.
(PDF)

S24 Appendix COI_GMYC_log.rtf. Log-file from the GMYC-analysis on COI-unique.
(RTF)

S25 Appendix COI_GMYC_support.xls. Support-values for nodes from the GMYC-analysis on COI-unique.
(XLS)

S26 Appendix ITS2x_GMYC_code_nodes.pdf. Topology from the GMYC-analysis on ITS2s with node numbers.
(PDF)

S27 Appendix ITS2x_GMYC_log.rtf. Log-file from the GMYC-analysis on ITS2s.
(RTF)

S28 Appendix ITS2x_GMYC_support.xls. Support-values for nodes from the GMYC-analysis on COI.
(XLS)

S29 Appendix ITS2s_GMYC_code_nodes.pdf. Topology from the GMYC-analysis on ITS2s with node numbers.
(PDF)

S30 Appendix ITS2s_GMYC_log.rtf. Log-file from the GMYC-analysis on ITS2s.
(RTF)

S31 Appendix ITS2s_GMYC_support.xls. Support-values for nodes from the GMYC-analysis on COI.
(XLS)

S32 Appendix STACEY_log.txt. Log-file from STACEY analysis on the COI_and_ITS2s data set.
(TXT)

S33 Appendix Distances_COI.xlsx. Uncorrected distances from COI-all data set.
(XLSX)

S34 Appendix Distances_ITS2s.xlsx. Uncorrected distances from ITS2s data set.
(XLSX)

S35 Appendix Uniqhaplo.pl. Pearl script originally downloaded from the web page of Dr. Naoki Takebayashi at University of Alaska Fairbanks (Department of Biology and Wildlife).
(PL) Click here for additional data file.

S36 Appendix Specimen list. List of sequenced specimens with voucher specification, site ID (see Table 1), sequence ID, and GenBank accession numbers.
(DOCX)
