# Supplementary material for: A mega-cryptic species complex hidden among one of the most common annelids in the North East Atlantic
Source: PLoS One. 2018 Jun 20;13(6):e0198356. doi: 10.1371/journal.pone.0198356 (PMC6010226; doi:10.1371/journal.pone.0198356)
Supplement: S20 Appendix — Log-file from the TCS-analysis on COI-all. (RTF) [file pone.0198356.s020.rtf]

TCS v1.21Thu Nov 02 09:47:33 CET 2017Datafile = /Users/arnenygren/Desktop/namnlös mapp 3/mcapa462_cox1.nexCurrent OS = macintoshData in NEXUS formatNumber of sequences: 462Length of sequences: 657_________________________________________________________PARSIMONY PROBABILITYFor 1 step(s),	P(95%) = 0.9994633998543423For 2 step(s),	P(95%) = 0.9978902596489967For 3 step(s),	P(95%) = 0.9956109996859103For 4 step(s),	P(95%) = 0.9925794844579223For 5 step(s),	P(95%) = 0.9887969763040089For 6 step(s),	P(95%) = 0.9842705654200288For 7 step(s),	P(95%) = 0.9790085628411356For 8 step(s),	P(95%) = 0.973020882788166For 9 step(s),	P(95%) = 0.9663190957656734For 10 step(s),	P(95%) = 0.9589163881433932For 11 step(s),	P(95%) = 0.9508275183378267For 12 step(s),	P(95%) = 0.9420687683033846RUN SETTINGSCalculated maximum connection steps at 95% = 11Gaps treated as missing dataWARNING: POSSIBLE MISSING DATA AMBIGUITIES2216_2 and 2322_2 differ only by missing or ambiguous characters841_2 and 2351_2 differ only by missing or ambiguous characters2271_2 and 2353_2 differ only by missing or ambiguous characters2216_2 and 2360_2 differ only by missing or ambiguous characters2367_2 and 2368_2 differ only by missing or ambiguous characters841_2 and 2369_2 differ only by missing or ambiguous characters2216_2 and 2382_2 differ only by missing or ambiguous characters2275_3 and 2285_3 differ only by missing or ambiguous characters2275_3 and 2287_3 differ only by missing or ambiguous characters2275_3 and 2873_3 differ only by missing or ambiguous characters2275_3 and 2878_3 differ only by missing or ambiguous characters2913_3 and 2917_3 differ only by missing or ambiguous characters2890_3 and 2933_3 differ only by missing or ambiguous characters825_1 and 828_1 differ only by missing or ambiguous characters825_1 and 830_1 differ only by missing or ambiguous characters825_1 and 831_1 differ only by missing or ambiguous characters825_1 and 834_1 differ only by missing or ambiguous characters825_1 and 835_1 differ only by missing or ambiguous characters825_1 and 836_1 differ only by missing or ambiguous characters825_1 and 837_1 differ only by missing or ambiguous characters825_1 and 843_1 differ only by missing or ambiguous characters825_1 and 856_1 differ only by missing or ambiguous characters825_1 and 857_1 differ only by missing or ambiguous characters825_1 and 1322_1 differ only by missing or ambiguous characters825_1 and 1323_1 differ only by missing or ambiguous characters826_1 and 1324_1 differ only by missing or ambiguous characters825_1 and 1326_1 differ only by missing or ambiguous characters825_1 and 1328_1 differ only by missing or ambiguous characters825_1 and 1329_1 differ only by missing or ambiguous characters825_1 and 1330_1 differ only by missing or ambiguous characters825_1 and 1333_1 differ only by missing or ambiguous characters825_1 and 1335_1 differ only by missing or ambiguous characters825_1 and 1336_1 differ only by missing or ambiguous characters825_1 and 1337_1 differ only by missing or ambiguous characters825_1 and 1338_1 differ only by missing or ambiguous characters825_1 and 1339_1 differ only by missing or ambiguous characters825_1 and 1340_1 differ only by missing or ambiguous characters825_1 and 1344_1 differ only by missing or ambiguous characters825_1 and 1938_1 differ only by missing or ambiguous characters825_1 and 1939_1 differ only by missing or ambiguous characters825_1 and 1940_1 differ only by missing or ambiguous characters825_1 and 1945_1 differ only by missing or ambiguous characters825_1 and 1947_1 differ only by missing or ambiguous characters1325_1 and 1948_1 differ only by missing or ambiguous characters825_1 and 1949_1 differ only by missing or ambiguous characters825_1 and 1950_1 differ only by missing or ambiguous characters825_1 and 1951_1 differ only by missing or ambiguous characters825_1 and 1952_1 differ only by missing or ambiguous characters825_1 and 2241_1 differ only by missing or ambiguous characters825_1 and 2439_1 differ only by missing or ambiguous characters825_1 and 2441_1 differ only by missing or ambiguous characters1325_1 and 2444_1 differ only by missing or ambiguous characters825_1 and 2446_1 differ only by missing or ambiguous characters858_1 and 2794_1 differ only by missing or ambiguous characters825_1 and 2839_1 differ only by missing or ambiguous characters1342_1 and 2841_1 differ only by missing or ambiguous characters825_1 and 2860_1 differ only by missing or ambiguous characters1342_1 and 2861_1 differ only by missing or ambiguous characters825_1 and 2862_1 differ only by missing or ambiguous characters825_1 and 2871_1 differ only by missing or ambiguous characters825_1 and 2909_1 differ only by missing or ambiguous characters858_1 and 2911_1 differ only by missing or ambiguous characters858_1 and TB29_1 differ only by missing or ambiguous characters2005_15 and 2007_15 differ only by missing or ambiguous characters2005_15 and 2009_15 differ only by missing or ambiguous characters2004_15 and 2010_15 differ only by missing or ambiguous characters2005_15 and 2011_15 differ only by missing or ambiguous characters2005_15 and 2017_15 differ only by missing or ambiguous characters2005_15 and 2018_15 differ only by missing or ambiguous characters2004_15 and 2019_15 differ only by missing or ambiguous characters2004_15 and 2043_15 differ only by missing or ambiguous characters2005_15 and 2008_15 differ only by missing or ambiguous characters2004_15 and 2012_15 differ only by missing or ambiguous characters2004_15 and 2849_15 differ only by missing or ambiguous characters2864_24 and 2865_24 differ only by missing or ambiguous characters2864_24 and 2866_24 differ only by missing or ambiguous characters2864_24 and 2867_24 differ only by missing or ambiguous characters2801_25 and 2802_25 differ only by missing or ambiguous characters2801_25 and 2810_25 differ only by missing or ambiguous characters839_6 and 845_6 differ only by missing or ambiguous characters838_6 and 846_6 differ only by missing or ambiguous characters839_6 and 847_6 differ only by missing or ambiguous characters839_6 and 848_6 differ only by missing or ambiguous characters839_6 and 849_6 differ only by missing or ambiguous characters839_6 and 860_6 differ only by missing or ambiguous characters839_6 and 1315_6 differ only by missing or ambiguous characters839_6 and 1316_6 differ only by missing or ambiguous characters839_6 and 1317_6 differ only by missing or ambiguous characters839_6 and 1318_6 differ only by missing or ambiguous characters838_6 and 1321_6 differ only by missing or ambiguous characters839_6 and 1869_6 differ only by missing or ambiguous characters1314_6 and 1873_6 differ only by missing or ambiguous characters839_6 and 1874_6 differ only by missing or ambiguous characters839_6 and 1942_6 differ only by missing or ambiguous characters839_6 and 1943_6 differ only by missing or ambiguous characters838_6 and 1944_6 differ only by missing or ambiguous characters838_6 and 2046_6 differ only by missing or ambiguous characters838_6 and 2172_6 differ only by missing or ambiguous characters839_6 and 2173_6 differ only by missing or ambiguous characters2167_6 and 2190_6 differ only by missing or ambiguous characters839_6 and 2774_6 differ only by missing or ambiguous characters829_7 and 2442_7 differ only by missing or ambiguous characters829_7 and 2443_7 differ only by missing or ambiguous characters2452_7 and 2859_7 differ only by missing or ambiguous characters2452_7 and 2863_7 differ only by missing or ambiguous characters1985_8 and 1989_8 differ only by missing or ambiguous characters1988_8 and 1991_8 differ only by missing or ambiguous characters1985_8 and 1992_8 differ only by missing or ambiguous characters1985_8 and 2000_8 differ only by missing or ambiguous characters1985_8 and 2015_8 differ only by missing or ambiguous characters2799_8 and 2920_8 differ only by missing or ambiguous characters2321_10 and 1990_10 differ only by missing or ambiguous characters2321_10 and 2024_10 differ only by missing or ambiguous characters2321_10 and 2026_10 differ only by missing or ambiguous characters2321_10 and 2029_10 differ only by missing or ambiguous characters2031_10 and 2032_10 differ only by missing or ambiguous characters2321_10 and 2033_10 differ only by missing or ambiguous characters2321_10 and TB25_10 differ only by missing or ambiguous characters2321_10 and TB26_10 differ only by missing or ambiguous characters1560_11 and 2323_11 differ only by missing or ambiguous characters2199_12 and 2223_12 differ only by missing or ambiguous characters2171_12 and 2224_12 differ only by missing or ambiguous characters2199_12 and 2225_12 differ only by missing or ambiguous characters2199_12 and 2806_12 differ only by missing or ambiguous characters2199_12 and 2824_12 differ only by missing or ambiguous characters2826_12 and 2829_12 differ only by missing or ambiguous characters2222_12 and 2832_12 differ only by missing or ambiguous characters1205_13 and 1956_13 differ only by missing or ambiguous characters1205_13 and 1960_13 differ only by missing or ambiguous characters2184_13 and 2317_13 differ only by missing or ambiguous characters2184_13 and 2454_13 differ only by missing or ambiguous characters1959_13 and 2776_13 differ only by missing or ambiguous characters2184_13 and 2813_13 differ only by missing or ambiguous characters2184_13 and 2035_13 differ only by missing or ambiguous characters1205_13 and 1999_13 differ only by missing or ambiguous characters2184_13 and 2027_13 differ only by missing or ambiguous characters1205_13 and 2028_13 differ only by missing or ambiguous characters2184_13 and T01_13 differ only by missing or ambiguous characters1959_13 and T03_13 differ only by missing or ambiguous characters2324_20 and 2329_20 differ only by missing or ambiguous characters2324_20 and 2348_20 differ only by missing or ambiguous characters2875_20 and 2903_20 differ only by missing or ambiguous characters2342_21 and 2377_21 differ only by missing or ambiguous characters2342_21 and 2384_21 differ only by missing or ambiguous characters2342_21 and 2385_21 differ only by missing or ambiguous characters2342_21 and 2815_21 differ only by missing or ambiguous characters2342_21 and 2816_21 differ only by missing or ambiguous characters2342_21 and 2817_21 differ only by missing or ambiguous characters2342_21 and 2819_21 differ only by missing or ambiguous characters2342_21 and 2820_21 differ only by missing or ambiguous characters2342_21 and 2823_21 differ only by missing or ambiguous characters2342_21 and 2825_21 differ only by missing or ambiguous characters2342_21 and 2831_21 differ only by missing or ambiguous characters2342_21 and 2833_21 differ only by missing or ambiguous characters2342_21 and 2836_21 differ only by missing or ambiguous characters2342_21 and 2895_21 differ only by missing or ambiguous characters2342_21 and 2830_21 differ only by missing or ambiguous characters2342_21 and 2834_21 differ only by missing or ambiguous characters2842_14 and 2844_14 differ only by missing or ambiguous characters2842_14 and 2845_14 differ only by missing or ambiguous characters2842_14 and 2846_14 differ only by missing or ambiguous characters2842_14 and 2848_14 differ only by missing or ambiguous characters2847_14 and 2850_14 differ only by missing or ambiguous characters2842_14 and 2851_14 differ only by missing or ambiguous characters2842_14 and 2853_14 differ only by missing or ambiguous characters2842_14 and 2854_14 differ only by missing or ambiguous characters2842_14 and 2855_14 differ only by missing or ambiguous characters2842_14 and 2042_14 differ only by missing or ambiguous characters2842_14 and 2044_14 differ only by missing or ambiguous characters2842_14 and 2477_14 differ only by missing or ambiguous characters2842_14 and POLYNOR086_13_14 differ only by missing or ambiguous characters2227_4 and 2230_4 differ only by missing or ambiguous characters2228_4 and 2231_4 differ only by missing or ambiguous characters2227_4 and 2232_4 differ only by missing or ambiguous characters2228_4 and 2233_4 differ only by missing or ambiguous characters2228_4 and 2235_4 differ only by missing or ambiguous characters2228_4 and 2236_4 differ only by missing or ambiguous characters2228_4 and TB27_4 differ only by missing or ambiguous characters2777_5 and 2778_5 differ only by missing or ambiguous characters2777_5 and 2780_5 differ only by missing or ambiguous characters2777_5 and 2790_5 differ only by missing or ambiguous characters2777_5 and 2792_5 differ only by missing or ambiguous characters2777_5 and 2793_5 differ only by missing or ambiguous characters2779_5 and 2900_5 differ only by missing or ambiguous characters2779_5 and 2926_5 differ only by missing or ambiguous characters2779_5 and 2927_5 differ only by missing or ambiguous charactersPONOR087_13_5 and T04_5 differ only by missing or ambiguous characters2284_16 and 2296_16 differ only by missing or ambiguous characters2284_16 and 2310_16 differ only by missing or ambiguous characters2267_16 and 2355_16 differ only by missing or ambiguous characters2340_16 and 2364_16 differ only by missing or ambiguous characters2295_16 and 2472_16 differ only by missing or ambiguous charactersHAPLOTYPESNumber of haplotypes = 270Haplotype list: - 841_2 :  2351_2 2369_2 - 844_2 :  - 1311_2 :  - 2180_2 :  - 2181_2 :  - 2182_2 :  - 2185_2 :  - 2187_2 :  - 2216_2 :  2322_2 2360_2 2382_2 - 2271_2 :  2353_2 - 2272_2 :  - 2326_2 :  - 2328_2 :  - 2331_2 :  - 2332_2 :  - 2333_2 :  - 2338_2 :  - 2352_2 :  - 2354_2 :  - 2367_2 :  2368_2 - 2370_2 :  - 2378_2 :  - 2381_2 :  - 1987_2 :  - POLYNOR088_13_2 :  - 1207_3 :  - 1310_3 :  - 2275_3 :  2285_3 2287_3 2873_3 2878_3 - 2380_3 :  - 2463_3 :  - 2464_3 :  - 2465_3 :  - 2812_3 :  - 2814_3 :  - 2872_3 :  - 2874_3 :  - 2876_3 :  - 2877_3 :  - 2879_3 :  - 2880_3 :  - 2881_3 :  - 2882_3 :  - 2883_3 :  - 2884_3 :  - 2885_3 :  - 2886_3 :  - 2887_3 :  - 2888_3 :  - 2889_3 :  - 2890_3 :  2933_3 - 2891_3 :  - 2901_3 :  - 2906_3 :  - 2907_3 :  - 2908_3 :  - 2910_3 :  - 2912_3 :  - 2913_3 :  2917_3 - 2915_3 :  - 2916_3 :  - 2922_3 :  - 2923_3 :  - 2929_3 :  - 2930_3 :  - 2931_3 :  - 2932_3 :  - 2934_3 :  - 2935_3 :  - 2480_3 :  - 825_1 :  828_1 830_1 831_1 834_1 835_1 836_1 837_1 843_1 856_1 857_1 1322_1 1323_1 1326_1 1328_1 1329_1 1330_1 1333_1 1335_1 1336_1 1337_1 1338_1 1339_1 1340_1 1344_1 1938_1 1939_1 1940_1 1945_1 1947_1 1949_1 1950_1 1951_1 1952_1 2241_1 2439_1 2441_1 2446_1 2839_1 2860_1 2862_1 2871_1 2909_1 - 826_1 :  1324_1 - 833_1 :  - 858_1 :  2794_1 2911_1 TB29_1 - 1325_1 :  1948_1 2444_1 - 1327_1 :  - 1342_1 :  2841_1 2861_1 - 2440_1 :  - 2445_1 :  - 2453_1 :  - 2796_1 :  - T05_1 :  - T06_1 :  - 2004_15 :  2010_15 2019_15 2043_15 2012_15 2849_15 - 2005_15 :  2007_15 2009_15 2011_15 2017_15 2018_15 2008_15 - 2006_15 :  - 2030_15 :  - 2864_24 :  2865_24 2866_24 2867_24 - 2869_24 :  - 2801_25 :  2802_25 2810_25 - 2809_25 :  - 838_6 :  846_6 1321_6 1944_6 2046_6 2172_6 - 839_6 :  845_6 847_6 848_6 849_6 860_6 1315_6 1316_6 1317_6 1318_6 1869_6 1874_6 1942_6 1943_6 2173_6 2774_6 - 850_6 :  - 1313_6 :  - 1314_6 :  1873_6 - 1319_6 :  - 1870_6 :  - 1871_6 :  - 1872_6 :  - 1875_6 :  - 2167_6 :  2190_6 - 829_7 :  2442_7 2443_7 - 1309_7 :  - 2447_7 :  - 2448_7 :  - 2449_7 :  - 2450_7 :  - 2452_7 :  2859_7 2863_7 - 2914_7 :  - 1197_8 :  - 1198_8 :  - 1199_8 :  - 1200_8 :  - 1202_8 :  - 1203_8 :  - 1561_8 :  - 1922_8 :  - 1946_8 :  - 1957_8 :  - 1958_8 :  - 1984_8 :  - 1985_8 :  1989_8 1992_8 2000_8 2015_8 - 1988_8 :  1991_8 - 1994_8 :  - 1995_8 :  - 1996_8 :  - 2001_8 :  - 2002_8 :  - 2013_8 :  - 2014_8 :  - 2036_8 :  - 2037_8 :  - 2039_8 :  - 2214_8 :  - 2456_8 :  - 2457_8 :  - 2476_8 :  - 2478_8 :  - 2775_8 :  - 2798_8 :  - 2799_8 :  2920_8 - 2896_8 :  - 2925_8 :  - 859_9 :  - 862_9 :  - 2274_17 :  - 2277_22 :  - 2278_19 :  - 2281_23 :  - 2304_10 :  - 2321_10 :  1990_10 2024_10 2026_10 2029_10 2033_10 TB25_10 TB26_10 - 2031_10 :  2032_10 - 2034_10 :  - 1560_11 :  2323_11 - 2347_11 :  - 2786_11 :  - 2899_11 :  - 1312_12 :  - 2171_12 :  2224_12 - 2193_12 :  - 2194_12 :  - 2195_12 :  - 2196_12 :  - 2197_12 :  - 2198_12 :  - 2199_12 :  2223_12 2225_12 2806_12 2824_12 - 2200_12 :  - 2201_12 :  - 2202_12 :  - 2222_12 :  2832_12 - 2818_12 :  - 2826_12 :  2829_12 - 2827_12 :  - 1201_13 :  - 1205_13 :  1956_13 1960_13 1999_13 2028_13 - 1923_13 :  - 1959_13 :  2776_13 T03_13 - 2183_13 :  - 2184_13 :  2317_13 2454_13 2813_13 2035_13 2027_13 T01_13 - 2215_13 :  - 2337_13 :  - 2458_13 :  - 2475_13 :  - 2921_13 :  - 2038_13 :  - 1986_13 :  - 1998_13 :  - 2312_18 :  - 2313_18 :  - 2314_18 :  - 2302_20 :  - 2324_20 :  2329_20 2348_20 - 2349_20 :  - 2875_20 :  2903_20 - 2342_21 :  2377_21 2384_21 2385_21 2815_21 2816_21 2817_21 2819_21 2820_21 2823_21 2825_21 2831_21 2833_21 2836_21 2895_21 2830_21 2834_21 - 2894_21 :  - 2842_14 :  2844_14 2845_14 2846_14 2848_14 2851_14 2853_14 2854_14 2855_14 2042_14 2044_14 2477_14 POLYNOR086_13_14 - 2843_14 :  - 2847_14 :  2850_14 - 2852_14 :  - 2479_14 :  - 2226_4 :  - 2227_4 :  2230_4 2232_4 - 2228_4 :  2231_4 2233_4 2235_4 2236_4 TB27_4 - 2229_4 :  - 2234_4 :  - 2045_4 :  - TB28_4 :  - 2805_26 :  - 2800_27 :  - 840_5 :  - 842_5 :  - 2777_5 :  2778_5 2780_5 2790_5 2792_5 2793_5 - 2779_5 :  2900_5 2926_5 2927_5 - 2791_5 :  - 2904_5 :  - 2918_5 :  - 2928_5 :  - PONOR087_13_5 :  T04_5 - T02_5 :  - 2267_16 :  2355_16 - 2268_16 :  - 2269_16 :  - 2270_16 :  - 2279_16 :  - 2280_16 :  - 2284_16 :  2296_16 2310_16 - 2288_16 :  - 2289_16 :  - 2290_16 :  - 2294_16 :  - 2295_16 :  2472_16 - 2297_16 :  - 2298_16 :  - 2299_16 :  - 2300_16 :  - 2303_16 :  - 2305_16 :  - 2306_16 :  - 2307_16 :  - 2308_16 :  - 2309_16 :  - 2311_16 :  - 2315_16 :  - 2316_16 :  - 2325_16 :  - 2330_16 :  - 2339_16 :  - 2340_16 :  2364_16 - 2344_16 :  - 2345_16 :  - 2356_16 :  - 2357_16 :  - 2358_16 :  - 2359_16 :  - 2361_16 :  - 2362_16 :  - 2363_16 :  - 2365_16 :  - 2366_16 :  - 2373_16 :  - 2374_16 :  - 2389_16 :  - 2466_16 :  - 2467_16 :  - 2468_16 :  - 2469_16 :  - 2470_16 :  - 2471_16 :  - 2474_16 : CONNECTIONSadding two min taxa 19[2367_2] , 8[2216_2]In clusters 17 , 22 distance 2adding two min taxa 158[2171_12] , 157[1312_12]In clusters 158 , 172 distance 2adding two min taxa 158[2171_12] , 165[2199_12]In clusters 158 , 172 distance 2adding two min taxa 159[2193_12] , 171[2826_12]In clusters 158 , 172 distance 2adding two min taxa 176[1959_13] , 174[1205_13]In clusters 184 , 185 distance 2adding two min taxa 176[1959_13] , 178[2184_13]In clusters 184 , 185 distance 2adding two min taxa 203[2228_4] , 202[2227_4]In clusters 201 , 206 distance 3adding two min taxa 222[2269_16] , 238[2306_16]In clusters 230 , 265 distance 3adding two min taxa 222[2269_16] , 223[2270_16]In clusters 230 , 265 distance 3adding two min taxa 222[2269_16] , 231[2295_16]In clusters 230 , 265 distance 3adding two min taxa 229[2290_16] , 244[2316_16]In clusters 230 , 244 distance 3adding two min taxa 229[2290_16] , 260[2373_16]In clusters 230 , 244 distance 3adding two min taxa 249[2344_16] , 244[2316_16]In clusters 230 , 244 distance 3adding two min taxa 249[2344_16] , 260[2373_16]In clusters 230 , 244 distance 3adding two min taxa 223[2270_16] , 264[2467_16]In clusters 230 , 269 distance 3adding two min taxa 223[2270_16] , 266[2469_16]In clusters 230 , 269 distance 3adding two min taxa 223[2270_16] , 268[2471_16]In clusters 230 , 269 distance 3adding two min taxa 231[2295_16] , 264[2467_16]In clusters 230 , 269 distance 3adding two min taxa 231[2295_16] , 266[2469_16]In clusters 230 , 269 distance 3adding two min taxa 231[2295_16] , 268[2471_16]In clusters 230 , 269 distance 3adding two min taxa 6[2185_2] , 9[2271_2]In clusters 17 , 20 distance 4adding two min taxa 6[2185_2] , 0[841_2]In clusters 17 , 20 distance 4adding two min taxa 6[2185_2] , 24[POLYNOR088_13_2]In clusters 17 , 20 distance 4adding two min taxa 4[2181_2] , 0[841_2]In clusters 17 , 20 distance 4adding two min taxa 18[2354_2] , 0[841_2]In clusters 17 , 20 distance 4adding two min taxa 41[2882_3] , 60[2922_3]In clusters 56 , 68 distance 4adding two min taxa 212[2777_5] , 213[2779_5]In clusters 210 , 218 distance 4adding two min taxa 29[2463_3] , 58[2915_3]In clusters 28 , 56 distance 5adding two min taxa 109[1197_8] , 131[2037_8]In clusters 128 , 140 distance 8REAL DISTANCE MATRIXpos:   		  1  2  3  4  5  6  7  8  9  10  11  12  13  14  15  16  17  18  19  20  21  22  23  24  25  26  27  28  29  30  31  32  33  34  35  36  37  38  39  40  41  42  43  44  45  46  47  48  49  50  51  52  53  54  55  56  57  58  59  60  61  62  63  64  65  66  67  68  69  70  71  72  73  74  75  76  77  78  79  80  81  82  83  84  85  86  87  88  89  90  91  92  93  94  95  96  97  98  99  100  101  102  103  104  105  106  107  108  109  110  111  112  113  114  115  116  117  118  119  120  121  122  123  124  125  126  127  128  129  130  131  132  133  134  135  136  137  138  139  140  141  142  143  144  145  146  147  148  149  150  151  152  153  154  155  156  157  158  159  160  161  162  163  164  165  166  167  168  169  170  171  172  173  174  175  176  177  178  179  180  181  182  183  184  185  186  187  188  189  190  191  192  193  194  195  196  197  198  199  200  201  202  203  204  205  206  207  208  209  210  211  212  213  214  215  216  217  218  219  220  221  222  223  224  225  226  227  228  229  230  231  232  233  234  235  236  237  238  239  240  241  242  243  244  245  246  247  248  249  250  251  252  253  254  255  256  257  258  259  260  261  262  263  264  265  266  267  268  269  270 [841_2]1 	: --  6  6  6  4  6  4  6  5  4  5  6  6  7  5  6  8  10  4  7  5  6  7  6  3  ##  ##  ##  ##  ##  ##  ##  ##  ##  ##  ##  ##  ##  ##  ##  ##  ##  ##  ##  ##  ##  ##  ##  ##  ##  ##  ##  ##  ##  ##  ##  ##  ##  ##  ##  ##  ##  ##  ##  ##  ##  ##  ##  ##  ##  ##  ##  ##  ##  ##  ##  ##  ##  ##  ##  ##  ##  ##  ##  ##  ##  ##  ##  ##  ##  ##  ##  ##  ##  ##  ##  ##  ##  ##  ##  ##  ##  ##  ##  ##  ##  ##  ##  ##  ##  ##  ##  ##  ##  ##  ##  ##  ##  ##  ##  ##  ##  ##  ##  ##  ##  ##  ##  ##  ##  ##  ##  ##  ##  ##  ##  ##  ##  ##  ##  ##  ##  ##  ##  ##  ##  ##  ##  ##  ##  ##  ##  ##  ##  ##  ##  ##  ##  ##  ##  ##  ##  ##  ##  ##  ##  ##  ##  ##  ##  ##  ##  ##  ##  ##  ##  ##  ##  ##  ##  ##  ##  ##  ##  ##  ##  ##  ##  ##  ##  ##  ##  ##  ##  ##  ##  ##  ##  ##  ##  ##  ##  ##  ##  ##  ##  ##  ##  ##  ##  ##  ##  ##  ##  ##  ##  ##  ##  ##  ##  ##  ##  ##  ##  ##  ##  ##  ##  ##  ##  ##  ##  ##  ##  ##  ##  ##  ##  ##  ##  ##  ##  ##  ##  ##  ##  ##  ##  ##  ##  ##  ##  ##  ##  ##  ##  ##  ##  ##  ##  ##  ##  ##  ##  ##  ##  ##  ##  ##  ## [844_2]2 	: 6  --  2  3  2  3  2  2  1  6  3  2  2  3  7  2  4  6  2  3  11  2  3  2  5  ##  ##  ##  ##  ##  ##  ##  ##  ##  ##  ##  ##  ##  ##  ##  ##  ##  ##  ##  ##  ##  ##  ##  ##  ##  ##  ##  ##  ##  ##  ##  ##  ##  ##  ##  ##  ##  ##  ##  ##  ##  ##  ##  ##  ##  ##  ##  ##  ##  ##  ##  ##  ##  ##  ##  ##  ##  ##  ##  ##  ##  ##  ##  ##  ##  ##  ##  ##  ##  ##  ##  ##  ##  ##  ##  ##  ##  ##  ##  ##  ##  ##  ##  ##  ##  ##  ##  ##  ##  ##  ##  ##  ##  ##  ##  ##  ##  ##  ##  ##  ##  ##  ##  ##  ##  ##  ##  ##  ##  ##  ##  ##  ##  ##  ##  ##  ##  ##  ##  ##  ##  ##  ##  ##  ##  ##  ##  ##  ##  ##  ##  ##  ##  ##  ##  ##  ##  ##  ##  ##  ##  ##  ##  ##  ##  ##  ##  ##  ##  ##  ##  ##  ##  ##  ##  ##  ##  ##  ##  ##  ##  ##  ##  ##  ##  ##  ##  ##  ##  ##  ##  ##  ##  ##  ##  ##  ##  ##  ##  ##  ##  ##  ##  ##  ##  ##  ##  ##  ##  ##  ##  ##  ##  ##  ##  ##  ##  ##  ##  ##  ##  ##  ##  ##  ##  ##  ##  ##  ##  ##  ##  ##  ##  ##  ##  ##  ##  ##  ##  ##  ##  ##  ##  ##  ##  ##  ##  ##  ##  ##  ##  ##  ##  ##  ##  ##  ##  ##  ##  ##  ##  ##  ##  ##  ## [1311_2]3 	: 6  2  --  3  2  1  2  2  1  6  3  2  2  3  7  2  4  6  2  3  11  2  3  2  5  ##  ##  ##  ##  ##  ##  ##  ##  ##  ##  ##  ##  ##  ##  ##  ##  ##  ##  ##  ##  ##  ##  ##  ##  ##  ##  ##  ##  ##  ##  ##  ##  ##  ##  ##  ##  ##  ##  ##  ##  ##  ##  ##  ##  ##  ##  ##  ##  ##  ##  ##  ##  ##  ##  ##  ##  ##  ##  ##  ##  ##  ##  ##  ##  ##  ##  ##  ##  ##  ##  ##  ##  ##  ##  ##  ##  ##  ##  ##  ##  ##  ##  ##  ##  ##  ##  ##  ##  ##  ##  ##  ##  ##  ##  ##  ##  ##  ##  ##  ##  ##  ##  ##  ##  ##  ##  ##  ##  ##  ##  ##  ##  ##  ##  ##  ##  ##  ##  ##  ##  ##  ##  ##  ##  ##  ##  ##  ##  ##  ##  ##  ##  ##  ##  ##  ##  ##  ##  ##  ##  ##  ##  ##  ##  ##  ##  ##  ##  ##  ##  ##  ##  ##  ##  ##  ##  ##  ##  ##  ##  ##  ##  ##  ##  ##  ##  ##  ##  ##  ##  ##  ##  ##  ##  ##  ##  ##  ##  ##  ##  ##  ##  ##  ##  ##  ##  ##  ##  ##  ##  ##  ##  ##  ##  ##  ##  ##  ##  ##  ##  ##  ##  ##  ##  ##  ##  ##  ##  ##  ##  ##  ##  ##  ##  ##  ##  ##  ##  ##  ##  ##  ##  ##  ##  ##  ##  ##  ##  ##  ##  ##  ##  ##  ##  ##  ##  ##  ##  ##  ##  ##  ##  ##  ##  ## [2180_2]4 	: 6  3  3  --  3  4  3  3  2  6  4  3  3  4  7  3  5  5  3  4  11  2  4  3  6  ##  ##  ##  ##  ##  ##  ##  ##  ##  ##  ##  ##  ##  ##  ##  ##  ##  ##  ##  ##  ##  ##  ##  ##  ##  ##  ##  ##  ##  ##  ##  ##  ##  ##  ##  ##  ##  ##  ##  ##  ##  ##  ##  ##  ##  ##  ##  ##  ##  ##  ##  ##  ##  ##  ##  ##  ##  ##  ##  ##  ##  ##  ##  ##  ##  ##  ##  ##  ##  ##  ##  ##  ##  ##  ##  ##  ##  ##  ##  ##  ##  ##  ##  ##  ##  ##  ##  ##  ##  ##  ##  ##  ##  ##  ##  ##  ##  ##  ##  ##  ##  ##  ##  ##  ##  ##  ##  ##  ##  ##  ##  ##  ##  ##  ##  ##  ##  ##  ##  ##  ##  ##  ##  ##  ##  ##  ##  ##  ##  ##  ##  ##  ##  ##  ##  ##  ##  ##  ##  ##  ##  ##  ##  ##  ##  ##  ##  ##  ##  ##  ##  ##  ##  ##  ##  ##  ##  ##  ##  ##  ##  ##  ##  ##  ##  ##  ##  ##  ##  ##  ##  ##  ##  ##  ##  ##  ##  ##  ##  ##  ##  ##  ##  ##  ##  ##  ##  ##  ##  ##  ##  ##  ##  ##  ##  ##  ##  ##  ##  ##  ##  ##  ##  ##  ##  ##  ##  ##  ##  ##  ##  ##  ##  ##  ##  ##  ##  ##  ##  ##  ##  ##  ##  ##  ##  ##  ##  ##  ##  ##  ##  ##  ##  ##  ##  ##  ##  ##  ##  ##  ##  ##  ##  ##  ## [2181_2]5 	: 4  2  2  3  --  3  2  2  1  6  1  2  2  3  7  2  4  6  2  3  9  2  3  2  6  ##  ##  ##  ##  ##  ##  ##  ##  ##  ##  ##  ##  ##  ##  ##  ##  ##  ##  ##  ##  ##  ##  ##  ##  ##  ##  ##  ##  ##  ##  ##  ##  ##  ##  ##  ##  ##  ##  ##  ##  ##  ##  ##  ##  ##  ##  ##  ##  ##  ##  ##  ##  ##  ##  ##  ##  ##  ##  ##  ##  ##  ##  ##  ##  ##  ##  ##  ##  ##  ##  ##  ##  ##  ##  ##  ##  ##  ##  ##  ##  ##  ##  ##  ##  ##  ##  ##  ##  ##  ##  ##  ##  ##  ##  ##  ##  ##  ##  ##  ##  ##  ##  ##  ##  ##  ##  ##  ##  ##  ##  ##  ##  ##  ##  ##  ##  ##  ##  ##  ##  ##  ##  ##  ##  ##  ##  ##  ##  ##  ##  ##  ##  ##  ##  ##  ##  ##  ##  ##  ##  ##  ##  ##  ##  ##  ##  ##  ##  ##  ##  ##  ##  ##  ##  ##  ##  ##  ##  ##  ##  ##  ##  ##  ##  ##  ##  ##  ##  ##  ##  ##  ##  ##  ##  ##  ##  ##  ##  ##  ##  ##  ##  ##  ##  ##  ##  ##  ##  ##  ##  ##  ##  ##  ##  ##  ##  ##  ##  ##  ##  ##  ##  ##  ##  ##  ##  ##  ##  ##  ##  ##  ##  ##  ##  ##  ##  ##  ##  ##  ##  ##  ##  ##  ##  ##  ##  ##  ##  ##  ##  ##  ##  ##  ##  ##  ##  ##  ##  ##  ##  ##  ##  ##  ##  ## [2182_2]6 	: 6  3  1  4  3  --  2  3  2  6  4  3  3  4  7  3  5  7  3  4  11  3  4  3  6  ##  ##  ##  ##  ##  ##  ##  ##  ##  ##  ##  ##  ##  ##  ##  ##  ##  ##  ##  ##  ##  ##  ##  ##  ##  ##  ##  ##  ##  ##  ##  ##  ##  ##  ##  ##  ##  ##  ##  ##  ##  ##  ##  ##  ##  ##  ##  ##  ##  ##  ##  ##  ##  ##  ##  ##  ##  ##  ##  ##  ##  ##  ##  ##  ##  ##  ##  ##  ##  ##  ##  ##  ##  ##  ##  ##  ##  ##  ##  ##  ##  ##  ##  ##  ##  ##  ##  ##  ##  ##  ##  ##  ##  ##  ##  ##  ##  ##  ##  ##  ##  ##  ##  ##  ##  ##  ##  ##  ##  ##  ##  ##  ##  ##  ##  ##  ##  ##  ##  ##  ##  ##  ##  ##  ##  ##  ##  ##  ##  ##  ##  ##  ##  ##  ##  ##  ##  ##  ##  ##  ##  ##  ##  ##  ##  ##  ##  ##  ##  ##  ##  ##  ##  ##  ##  ##  ##  ##  ##  ##  ##  ##  ##  ##  ##  ##  ##  ##  ##  ##  ##  ##  ##  ##  ##  ##  ##  ##  ##  ##  ##  ##  ##  ##  ##  ##  ##  ##  ##  ##  ##  ##  ##  ##  ##  ##  ##  ##  ##  ##  ##  ##  ##  ##  ##  ##  ##  ##  ##  ##  ##  ##  ##  ##  ##  ##  ##  ##  ##  ##  ##  ##  ##  ##  ##  ##  ##  ##  ##  ##  ##  ##  ##  ##  ##  ##  ##  ##  ##  ##  ##  ##  ##  ##  ## [2185_2]7 	: 4  2  2  3  2  2  --  2  1  4  3  2  2  3  5  2  4  6  2  3  9  2  3  2  4  ##  ##  ##  ##  ##  ##  ##  ##  ##  ##  ##  ##  ##  ##  ##  ##  ##  ##  ##  ##  ##  ##  ##  ##  ##  ##  ##  ##  ##  ##  ##  ##  ##  ##  ##  ##  ##  ##  ##  ##  ##  ##  ##  ##  ##  ##  ##  ##  ##  ##  ##  ##  ##  ##  ##  ##  ##  ##  ##  ##  ##  ##  ##  ##  ##  ##  ##  ##  ##  ##  ##  ##  ##  ##  ##  ##  ##  ##  ##  ##  ##  ##  ##  ##  ##  ##  ##  ##  ##  ##  ##  ##  ##  ##  ##  ##  ##  ##  ##  ##  ##  ##  ##  ##  ##  ##  ##  ##  ##  ##  ##  ##  ##  ##  ##  ##  ##  ##  ##  ##  ##  ##  ##  ##  ##  ##  ##  ##  ##  ##  ##  ##  ##  ##  ##  ##  ##  ##  ##  ##  ##  ##  ##  ##  ##  ##  ##  ##  ##  ##  ##  ##  ##  ##  ##  ##  ##  ##  ##  ##  ##  ##  ##  ##  ##  ##  ##  ##  ##  ##  ##  ##  ##  ##  ##  ##  ##  ##  ##  ##  ##  ##  ##  ##  ##  ##  ##  ##  ##  ##  ##  ##  ##  ##  ##  ##  ##  ##  ##  ##  ##  ##  ##  ##  ##  ##  ##  ##  ##  ##  ##  ##  ##  ##  ##  ##  ##  ##  ##  ##  ##  ##  ##  ##  ##  ##  ##  ##  ##  ##  ##  ##  ##  ##  ##  ##  ##  ##  ##  ##  ##  ##  ##  ##  ## [2187_2]8 	: 6  2  2  3  2  3  2  --  1  6  3  2  2  3  7  2  4  6  2  3  11  2  3  2  6  ##  ##  ##  ##  ##  ##  ##  ##  ##  ##  ##  ##  ##  ##  ##  ##  ##  ##  ##  ##  ##  ##  ##  ##  ##  ##  ##  ##  ##  ##  ##  ##  ##  ##  ##  ##  ##  ##  ##  ##  ##  ##  ##  ##  ##  ##  ##  ##  ##  ##  ##  ##  ##  ##  ##  ##  ##  ##  ##  ##  ##  ##  ##  ##  ##  ##  ##  ##  ##  ##  ##  ##  ##  ##  ##  ##  ##  ##  ##  ##  ##  ##  ##  ##  ##  ##  ##  ##  ##  ##  ##  ##  ##  ##  ##  ##  ##  ##  ##  ##  ##  ##  ##  ##  ##  ##  ##  ##  ##  ##  ##  ##  ##  ##  ##  ##  ##  ##  ##  ##  ##  ##  ##  ##  ##  ##  ##  ##  ##  ##  ##  ##  ##  ##  ##  ##  ##  ##  ##  ##  ##  ##  ##  ##  ##  ##  ##  ##  ##  ##  ##  ##  ##  ##  ##  ##  ##  ##  ##  ##  ##  ##  ##  ##  ##  ##  ##  ##  ##  ##  ##  ##  ##  ##  ##  ##  ##  ##  ##  ##  ##  ##  ##  ##  ##  ##  ##  ##  ##  ##  ##  ##  ##  ##  ##  ##  ##  ##  ##  ##  ##  ##  ##  ##  ##  ##  ##  ##  ##  ##  ##  ##  ##  ##  ##  ##  ##  ##  ##  ##  ##  ##  ##  ##  ##  ##  ##  ##  ##  ##  ##  ##  ##  ##  ##  ##  ##  ##  ##  ##  ##  ##  ##  ##  ## [2216_2]9 	: 5  1  1  2  1  2  1  1  --  5  2  1  1  2  6  1  3  5  1  2  10  1  2  1  5  ##  ##  ##  ##  ##  ##  ##  ##  ##  ##  ##  ##  ##  ##  ##  ##  ##  ##  ##  ##  ##  ##  ##  ##  ##  ##  ##  ##  ##  ##  ##  ##  ##  ##  ##  ##  ##  ##  ##  ##  ##  ##  ##  ##  ##  ##  ##  ##  ##  ##  ##  ##  ##  ##  ##  ##  ##  ##  ##  ##  ##  ##  ##  ##  ##  ##  ##  ##  ##  ##  ##  ##  ##  ##  ##  ##  ##  ##  ##  ##  ##  ##  ##  ##  ##  ##  ##  ##  ##  ##  ##  ##  ##  ##  ##  ##  ##  ##  ##  ##  ##  ##  ##  ##  ##  ##  ##  ##  ##  ##  ##  ##  ##  ##  ##  ##  ##  ##  ##  ##  ##  ##  ##  ##  ##  ##  ##  ##  ##  ##  ##  ##  ##  ##  ##  ##  ##  ##  ##  ##  ##  ##  ##  ##  ##  ##  ##  ##  ##  ##  ##  ##  ##  ##  ##  ##  ##  ##  ##  ##  ##  ##  ##  ##  ##  ##  ##  ##  ##  ##  ##  ##  ##  ##  ##  ##  ##  ##  ##  ##  ##  ##  ##  ##  ##  ##  ##  ##  ##  ##  ##  ##  ##  ##  ##  ##  ##  ##  ##  ##  ##  ##  ##  ##  ##  ##  ##  ##  ##  ##  ##  ##  ##  ##  ##  ##  ##  ##  ##  ##  ##  ##  ##  ##  ##  ##  ##  ##  ##  ##  ##  ##  ##  ##  ##  ##  ##  ##  ##  ##  ##  ##  ##  ##  ## [2271_2]10	: 4  6  6  6  6  6  4  6  5  --  7  6  6  7  1  6  8  10  6  7  7  6  7  6  1  ##  ##  ##  ##  ##  ##  ##  ##  ##  ##  ##  ##  ##  ##  ##  ##  ##  ##  ##  ##  ##  ##  ##  ##  ##  ##  ##  ##  ##  ##  ##  ##  ##  ##  ##  ##  ##  ##  ##  ##  ##  ##  ##  ##  ##  ##  ##  ##  ##  ##  ##  ##  ##  ##  ##  ##  ##  ##  ##  ##  ##  ##  ##  ##  ##  ##  ##  ##  ##  ##  ##  ##  ##  ##  ##  ##  ##  ##  ##  ##  ##  ##  ##  ##  ##  ##  ##  ##  ##  ##  ##  ##  ##  ##  ##  ##  ##  ##  ##  ##  ##  ##  ##  ##  ##  ##  ##  ##  ##  ##  ##  ##  ##  ##  ##  ##  ##  ##  ##  ##  ##  ##  ##  ##  ##  ##  ##  ##  ##  ##  ##  ##  ##  ##  ##  ##  ##  ##  ##  ##  ##  ##  ##  ##  ##  ##  ##  ##  ##  ##  ##  ##  ##  ##  ##  ##  ##  ##  ##  ##  ##  ##  ##  ##  ##  ##  ##  ##  ##  ##  ##  ##  ##  ##  ##  ##  ##  ##  ##  ##  ##  ##  ##  ##  ##  ##  ##  ##  ##  ##  ##  ##  ##  ##  ##  ##  ##  ##  ##  ##  ##  ##  ##  ##  ##  ##  ##  ##  ##  ##  ##  ##  ##  ##  ##  ##  ##  ##  ##  ##  ##  ##  ##  ##  ##  ##  ##  ##  ##  ##  ##  ##  ##  ##  ##  ##  ##  ##  ##  ##  ##  ##  ##  ##  ## [2272_2]11	: 5  3  3  4  1  4  3  3  2  7  --  3  3  4  8  3  4  6  3  3  10  3  4  3  6  ##  ##  ##  ##  ##  ##  ##  ##  ##  ##  ##  ##  ##  ##  ##  ##  ##  ##  ##  ##  ##  ##  ##  ##  ##  ##  ##  ##  ##  ##  ##  ##  ##  ##  ##  ##  ##  ##  ##  ##  ##  ##  ##  ##  ##  ##  ##  ##  ##  ##  ##  ##  ##  ##  ##  ##  ##  ##  ##  ##  ##  ##  ##  ##  ##  ##  ##  ##  ##  ##  ##  ##  ##  ##  ##  ##  ##  ##  ##  ##  ##  ##  ##  ##  ##  ##  ##  ##  ##  ##  ##  ##  ##  ##  ##  ##  ##  ##  ##  ##  ##  ##  ##  ##  ##  ##  ##  ##  ##  ##  ##  ##  ##  ##  ##  ##  ##  ##  ##  ##  ##  ##  ##  ##  ##  ##  ##  ##  ##  ##  ##  ##  ##  ##  ##  ##  ##  ##  ##  ##  ##  ##  ##  ##  ##  ##  ##  ##  ##  ##  ##  ##  ##  ##  ##  ##  ##  ##  ##  ##  ##  ##  ##  ##  ##  ##  ##  ##  ##  ##  ##  ##  ##  ##  ##  ##  ##  ##  ##  ##  ##  ##  ##  ##  ##  ##  ##  ##  ##  ##  ##  ##  ##  ##  ##  ##  ##  ##  ##  ##  ##  ##  ##  ##  ##  ##  ##  ##  ##  ##  ##  ##  ##  ##  ##  ##  ##  ##  ##  ##  ##  ##  ##  ##  ##  ##  ##  ##  ##  ##  ##  ##  ##  ##  ##  ##  ##  ##  ##  ##  ##  ##  ##  ##  ## [2326_2]12	: 6  2  2  3  2  3  2  2  1  6  3  --  2  3  7  2  4  6  2  3  11  2  3  2  6  ##  ##  ##  ##  ##  ##  ##  ##  ##  ##  ##  ##  ##  ##  ##  ##  ##  ##  ##  ##  ##  ##  ##  ##  ##  ##  ##  ##  ##  ##  ##  ##  ##  ##  ##  ##  ##  ##  ##  ##  ##  ##  ##  ##  ##  ##  ##  ##  ##  ##  ##  ##  ##  ##  ##  ##  ##  ##  ##  ##  ##  ##  ##  ##  ##  ##  ##  ##  ##  ##  ##  ##  ##  ##  ##  ##  ##  ##  ##  ##  ##  ##  ##  ##  ##  ##  ##  ##  ##  ##  ##  ##  ##  ##  ##  ##  ##  ##  ##  ##  ##  ##  ##  ##  ##  ##  ##  ##  ##  ##  ##  ##  ##  ##  ##  ##  ##  ##  ##  ##  ##  ##  ##  ##  ##  ##  ##  ##  ##  ##  ##  ##  ##  ##  ##  ##  ##  ##  ##  ##  ##  ##  ##  ##  ##  ##  ##  ##  ##  ##  ##  ##  ##  ##  ##  ##  ##  ##  ##  ##  ##  ##  ##  ##  ##  ##  ##  ##  ##  ##  ##  ##  ##  ##  ##  ##  ##  ##  ##  ##  ##  ##  ##  ##  ##  ##  ##  ##  ##  ##  ##  ##  ##  ##  ##  ##  ##  ##  ##  ##  ##  ##  ##  ##  ##  ##  ##  ##  ##  ##  ##  ##  ##  ##  ##  ##  ##  ##  ##  ##  ##  ##  ##  ##  ##  ##  ##  ##  ##  ##  ##  ##  ##  ##  ##  ##  ##  ##  ##  ##  ##  ##  ##  ##  ## [2328_2]13	: 6  2  2  3  2  3  2  2  1  6  3  2  --  3  7  2  4  6  2  3  11  2  3  2  5  ##  ##  ##  ##  ##  ##  ##  ##  ##  ##  ##  ##  ##  ##  ##  ##  ##  ##  ##  ##  ##  ##  ##  ##  ##  ##  ##  ##  ##  ##  ##  ##  ##  ##  ##  ##  ##  ##  ##  ##  ##  ##  ##  ##  ##  ##  ##  ##  ##  ##  ##  ##  ##  ##  ##  ##  ##  ##  ##  ##  ##  ##  ##  ##  ##  ##  ##  ##  ##  ##  ##  ##  ##  ##  ##  ##  ##  ##  ##  ##  ##  ##  ##  ##  ##  ##  ##  ##  ##  ##  ##  ##  ##  ##  ##  ##  ##  ##  ##  ##  ##  ##  ##  ##  ##  ##  ##  ##  ##  ##  ##  ##  ##  ##  ##  ##  ##  ##  ##  ##  ##  ##  ##  ##  ##  ##  ##  ##  ##  ##  ##  ##  ##  ##  ##  ##  ##  ##  ##  ##  ##  ##  ##  ##  ##  ##  ##  ##  ##  ##  ##  ##  ##  ##  ##  ##  ##  ##  ##  ##  ##  ##  ##  ##  ##  ##  ##  ##  ##  ##  ##  ##  ##  ##  ##  ##  ##  ##  ##  ##  ##  ##  ##  ##  ##  ##  ##  ##  ##  ##  ##  ##  ##  ##  ##  ##  ##  ##  ##  ##  ##  ##  ##  ##  ##  ##  ##  ##  ##  ##  ##  ##  ##  ##  ##  ##  ##  ##  ##  ##  ##  ##  ##  ##  ##  ##  ##  ##  ##  ##  ##  ##  ##  ##  ##  ##  ##  ##  ##  ##  ##  ##  ##  ##  ## [2331_2]14	: 7  3  3  4  3  4  3  3  2  7  4  3  3  --  8  3  5  7  3  4  10  3  4  3  6  ##  ##  ##  ##  ##  ##  ##  ##  ##  ##  ##  ##  ##  ##  ##  ##  ##  ##  ##  ##  ##  ##  ##  ##  ##  ##  ##  ##  ##  ##  ##  ##  ##  ##  ##  ##  ##  ##  ##  ##  ##  ##  ##  ##  ##  ##  ##  ##  ##  ##  ##  ##  ##  ##  ##  ##  ##  ##  ##  ##  ##  ##  ##  ##  ##  ##  ##  ##  ##  ##  ##  ##  ##  ##  ##  ##  ##  ##  ##  ##  ##  ##  ##  ##  ##  ##  ##  ##  ##  ##  ##  ##  ##  ##  ##  ##  ##  ##  ##  ##  ##  ##  ##  ##  ##  ##  ##  ##  ##  ##  ##  ##  ##  ##  ##  ##  ##  ##  ##  ##  ##  ##  ##  ##  ##  ##  ##  ##  ##  ##  ##  ##  ##  ##  ##  ##  ##  ##  ##  ##  ##  ##  ##  ##  ##  ##  ##  ##  ##  ##  ##  ##  ##  ##  ##  ##  ##  ##  ##  ##  ##  ##  ##  ##  ##  ##  ##  ##  ##  ##  ##  ##  ##  ##  ##  ##  ##  ##  ##  ##  ##  ##  ##  ##  ##  ##  ##  ##  ##  ##  ##  ##  ##  ##  ##  ##  ##  ##  ##  ##  ##  ##  ##  ##  ##  ##  ##  ##  ##  ##  ##  ##  ##  ##  ##  ##  ##  ##  ##  ##  ##  ##  ##  ##  ##  ##  ##  ##  ##  ##  ##  ##  ##  ##  ##  ##  ##  ##  ##  ##  ##  ##  ##  ##  ## [2332_2]15	: 5  7  7  7  7  7  5  7  6  1  8  7  7  8  --  7  9  11  7  8  8  7  8  7  2  ##  ##  ##  ##  ##  ##  ##  ##  ##  ##  ##  ##  ##  ##  ##  ##  ##  ##  ##  ##  ##  ##  ##  ##  ##  ##  ##  ##  ##  ##  ##  ##  ##  ##  ##  ##  ##  ##  ##  ##  ##  ##  ##  ##  ##  ##  ##  ##  ##  ##  ##  ##  ##  ##  ##  ##  ##  ##  ##  ##  ##  ##  ##  ##  ##  ##  ##  ##  ##  ##  ##  ##  ##  ##  ##  ##  ##  ##  ##  ##  ##  ##  ##  ##  ##  ##  ##  ##  ##  ##  ##  ##  ##  ##  ##  ##  ##  ##  ##  ##  ##  ##  ##  ##  ##  ##  ##  ##  ##  ##  ##  ##  ##  ##  ##  ##  ##  ##  ##  ##  ##  ##  ##  ##  ##  ##  ##  ##  ##  ##  ##  ##  ##  ##  ##  ##  ##  ##  ##  ##  ##  ##  ##  ##  ##  ##  ##  ##  ##  ##  ##  ##  ##  ##  ##  ##  ##  ##  ##  ##  ##  ##  ##  ##  ##  ##  ##  ##  ##  ##  ##  ##  ##  ##  ##  ##  ##  ##  ##  ##  ##  ##  ##  ##  ##  ##  ##  ##  ##  ##  ##  ##  ##  ##  ##  ##  ##  ##  ##  ##  ##  ##  ##  ##  ##  ##  ##  ##  ##  ##  ##  ##  ##  ##  ##  ##  ##  ##  ##  ##  ##  ##  ##  ##  ##  ##  ##  ##  ##  ##  ##  ##  ##  ##  ##  ##  ##  ##  ##  ##  ##  ##  ##  ##  ## [2333_2]16	: 6  2  2  3  2  3  2  2  1  6  3  2  2  3  7  --  4  6  2  3  11  2  3  2  5  ##  ##  ##  ##  ##  ##  ##  ##  ##  ##  ##  ##  ##  ##  ##  ##  ##  ##  ##  ##  ##  ##  ##  ##  ##  ##  ##  ##  ##  ##  ##  ##  ##  ##  ##  ##  ##  ##  ##  ##  ##  ##  ##  ##  ##  ##  ##  ##  ##  ##  ##  ##  ##  ##  ##  ##  ##  ##  ##  ##  ##  ##  ##  ##  ##  ##  ##  ##  ##  ##  ##  ##  ##  ##  ##  ##  ##  ##  ##  ##  ##  ##  ##  ##  ##  ##  ##  ##  ##  ##  ##  ##  ##  ##  ##  ##  ##  ##  ##  ##  ##  ##  ##  ##  ##  ##  ##  ##  ##  ##  ##  ##  ##  ##  ##  ##  ##  ##  ##  ##  ##  ##  ##  ##  ##  ##  ##  ##  ##  ##  ##  ##  ##  ##  ##  ##  ##  ##  ##  ##  ##  ##  ##  ##  ##  ##  ##  ##  ##  ##  ##  ##  ##  ##  ##  ##  ##  ##  ##  ##  ##  ##  ##  ##  ##  ##  ##  ##  ##  ##  ##  ##  ##  ##  ##  ##  ##  ##  ##  ##  ##  ##  ##  ##  ##  ##  ##  ##  ##  ##  ##  ##  ##  ##  ##  ##  ##  ##  ##  ##  ##  ##  ##  ##  ##  ##  ##  ##  ##  ##  ##  ##  ##  ##  ##  ##  ##  ##  ##  ##  ##  ##  ##  ##  ##  ##  ##  ##  ##  ##  ##  ##  ##  ##  ##  ##  ##  ##  ##  ##  ##  ##  ##  ##  ## [2338_2]17	: 8  4  4  5  4  5  4  4  3  8  4  4  4  5  9  4  --  4  4  1  13  4  5  4  7  ##  ##  ##  ##  ##  ##  ##  ##  ##  ##  ##  ##  ##  ##  ##  ##  ##  ##  ##  ##  ##  ##  ##  ##  ##  ##  ##  ##  ##  ##  ##  ##  ##  ##  ##  ##  ##  ##  ##  ##  ##  ##  ##  ##  ##  ##  ##  ##  ##  ##  ##  ##  ##  ##  ##  ##  ##  ##  ##  ##  ##  ##  ##  ##  ##  ##  ##  ##  ##  ##  ##  ##  ##  ##  ##  ##  ##  ##  ##  ##  ##  ##  ##  ##  ##  ##  ##  ##  ##  ##  ##  ##  ##  ##  ##  ##  ##  ##  ##  ##  ##  ##  ##  ##  ##  ##  ##  ##  ##  ##  ##  ##  ##  ##  ##  ##  ##  ##  ##  ##  ##  ##  ##  ##  ##  ##  ##  ##  ##  ##  ##  ##  ##  ##  ##  ##  ##  ##  ##  ##  ##  ##  ##  ##  ##  ##  ##  ##  ##  ##  ##  ##  ##  ##  ##  ##  ##  ##  ##  ##  ##  ##  ##  ##  ##  ##  ##  ##  ##  ##  ##  ##  ##  ##  ##  ##  ##  ##  ##  ##  ##  ##  ##  ##  ##  ##  ##  ##  ##  ##  ##  ##  ##  ##  ##  ##  ##  ##  ##  ##  ##  ##  ##  ##  ##  ##  ##  ##  ##  ##  ##  ##  ##  ##  ##  ##  ##  ##  ##  ##  ##  ##  ##  ##  ##  ##  ##  ##  ##  ##  ##  ##  ##  ##  ##  ##  ##  ##  ##  ##  ##  ##  ##  ##  ## [2352_2]18	: 10  6  6  5  6  7  6  6  5  10  6  6  6  7  11  6  4  --  5  3  15  4  7  6  10  ##  ##  ##  ##  ##  ##  ##  ##  ##  ##  ##  ##  ##  ##  ##  ##  ##  ##  ##  ##  ##  ##  ##  ##  ##  ##  ##  ##  ##  ##  ##  ##  ##  ##  ##  ##  ##  ##  ##  ##  ##  ##  ##  ##  ##  ##  ##  ##  ##  ##  ##  ##  ##  ##  ##  ##  ##  ##  ##  ##  ##  ##  ##  ##  ##  ##  ##  ##  ##  ##  ##  ##  ##  ##  ##  ##  ##  ##  ##  ##  ##  ##  ##  ##  ##  ##  ##  ##  ##  ##  ##  ##  ##  ##  ##  ##  ##  ##  ##  ##  ##  ##  ##  ##  ##  ##  ##  ##  ##  ##  ##  ##  ##  ##  ##  ##  ##  ##  ##  ##  ##  ##  ##  ##  ##  ##  ##  ##  ##  ##  ##  ##  ##  ##  ##  ##  ##  ##  ##  ##  ##  ##  ##  ##  ##  ##  ##  ##  ##  ##  ##  ##  ##  ##  ##  ##  ##  ##  ##  ##  ##  ##  ##  ##  ##  ##  ##  ##  ##  ##  ##  ##  ##  ##  ##  ##  ##  ##  ##  ##  ##  ##  ##  ##  ##  ##  ##  ##  ##  ##  ##  ##  ##  ##  ##  ##  ##  ##  ##  ##  ##  ##  ##  ##  ##  ##  ##  ##  ##  ##  ##  ##  ##  ##  ##  ##  ##  ##  ##  ##  ##  ##  ##  ##  ##  ##  ##  ##  ##  ##  ##  ##  ##  ##  ##  ##  ##  ##  ##  ##  ##  ##  ##  ##  ## [2354_2]19	: 4  2  2  3  2  3  2  2  1  6  3  2  2  3  7  2  4  5  --  3  9  2  3  2  5  ##  ##  ##  ##  ##  ##  ##  ##  ##  ##  ##  ##  ##  ##  ##  ##  ##  ##  ##  ##  ##  ##  ##  ##  ##  ##  ##  ##  ##  ##  ##  ##  ##  ##  ##  ##  ##  ##  ##  ##  ##  ##  ##  ##  ##  ##  ##  ##  ##  ##  ##  ##  ##  ##  ##  ##  ##  ##  ##  ##  ##  ##  ##  ##  ##  ##  ##  ##  ##  ##  ##  ##  ##  ##  ##  ##  ##  ##  ##  ##  ##  ##  ##  ##  ##  ##  ##  ##  ##  ##  ##  ##  ##  ##  ##  ##  ##  ##  ##  ##  ##  ##  ##  ##  ##  ##  ##  ##  ##  ##  ##  ##  ##  ##  ##  ##  ##  ##  ##  ##  ##  ##  ##  ##  ##  ##  ##  ##  ##  ##  ##  ##  ##  ##  ##  ##  ##  ##  ##  ##  ##  ##  ##  ##  ##  ##  ##  ##  ##  ##  ##  ##  ##  ##  ##  ##  ##  ##  ##  ##  ##  ##  ##  ##  ##  ##  ##  ##  ##  ##  ##  ##  ##  ##  ##  ##  ##  ##  ##  ##  ##  ##  ##  ##  ##  ##  ##  ##  ##  ##  ##  ##  ##  ##  ##  ##  ##  ##  ##  ##  ##  ##  ##  ##  ##  ##  ##  ##  ##  ##  ##  ##  ##  ##  ##  ##  ##  ##  ##  ##  ##  ##  ##  ##  ##  ##  ##  ##  ##  ##  ##  ##  ##  ##  ##  ##  ##  ##  ##  ##  ##  ##  ##  ##  ## [2367_2]20	: 7  3  3  4  3  4  3  3  2  7  3  3  3  4  8  3  1  3  3  --  12  3  4  3  6  ##  ##  ##  ##  ##  ##  ##  ##  ##  ##  ##  ##  ##  ##  ##  ##  ##  ##  ##  ##  ##  ##  ##  ##  ##  ##  ##  ##  ##  ##  ##  ##  ##  ##  ##  ##  ##  ##  ##  ##  ##  ##  ##  ##  ##  ##  ##  ##  ##  ##  ##  ##  ##  ##  ##  ##  ##  ##  ##  ##  ##  ##  ##  ##  ##  ##  ##  ##  ##  ##  ##  ##  ##  ##  ##  ##  ##  ##  ##  ##  ##  ##  ##  ##  ##  ##  ##  ##  ##  ##  ##  ##  ##  ##  ##  ##  ##  ##  ##  ##  ##  ##  ##  ##  ##  ##  ##  ##  ##  ##  ##  ##  ##  ##  ##  ##  ##  ##  ##  ##  ##  ##  ##  ##  ##  ##  ##  ##  ##  ##  ##  ##  ##  ##  ##  ##  ##  ##  ##  ##  ##  ##  ##  ##  ##  ##  ##  ##  ##  ##  ##  ##  ##  ##  ##  ##  ##  ##  ##  ##  ##  ##  ##  ##  ##  ##  ##  ##  ##  ##  ##  ##  ##  ##  ##  ##  ##  ##  ##  ##  ##  ##  ##  ##  ##  ##  ##  ##  ##  ##  ##  ##  ##  ##  ##  ##  ##  ##  ##  ##  ##  ##  ##  ##  ##  ##  ##  ##  ##  ##  ##  ##  ##  ##  ##  ##  ##  ##  ##  ##  ##  ##  ##  ##  ##  ##  ##  ##  ##  ##  ##  ##  ##  ##  ##  ##  ##  ##  ##  ##  ##  ##  ##  ##  ## [2370_2]21	: 5  11  11  11  9  11  9  11  10  7  10  11  11  10  8  11  13  15  9  12  --  11  12  11  8  ##  ##  ##  ##  ##  ##  ##  ##  ##  ##  ##  ##  ##  ##  ##  ##  ##  ##  ##  ##  ##  ##  ##  ##  ##  ##  ##  ##  ##  ##  ##  ##  ##  ##  ##  ##  ##  ##  ##  ##  ##  ##  ##  ##  ##  ##  ##  ##  ##  ##  ##  ##  ##  ##  ##  ##  ##  ##  ##  ##  ##  ##  ##  ##  ##  ##  ##  ##  ##  ##  ##  ##  ##  ##  ##  ##  ##  ##  ##  ##  ##  ##  ##  ##  ##  ##  ##  ##  ##  ##  ##  ##  ##  ##  ##  ##  ##  ##  ##  ##  ##  ##  ##  ##  ##  ##  ##  ##  ##  ##  ##  ##  ##  ##  ##  ##  ##  ##  ##  ##  ##  ##  ##  ##  ##  ##  ##  ##  ##  ##  ##  ##  ##  ##  ##  ##  ##  ##  ##  ##  ##  ##  ##  ##  ##  ##  ##  ##  ##  ##  ##  ##  ##  ##  ##  ##  ##  ##  ##  ##  ##  ##  ##  ##  ##  ##  ##  ##  ##  ##  ##  ##  ##  ##  ##  ##  ##  ##  ##  ##  ##  ##  ##  ##  ##  ##  ##  ##  ##  ##  ##  ##  ##  ##  ##  ##  ##  ##  ##  ##  ##  ##  ##  ##  ##  ##  ##  ##  ##  ##  ##  ##  ##  ##  ##  ##  ##  ##  ##  ##  ##  ##  ##  ##  ##  ##  ##  ##  ##  ##  ##  ##  ##  ##  ##  ##  ##  ##  ##  ##  ##  ##  ##  ##  ## [2378_2]22	: 6  2  2  2  2  3  2  2  1  6  3  2  2  3  7  2  4  4  2  3  11  --  3  2  5  ##  ##  ##  ##  ##  ##  ##  ##  ##  ##  ##  ##  ##  ##  ##  ##  ##  ##  ##  ##  ##  ##  ##  ##  ##  ##  ##  ##  ##  ##  ##  ##  ##  ##  ##  ##  ##  ##  ##  ##  ##  ##  ##  ##  ##  ##  ##  ##  ##  ##  ##  ##  ##  ##  ##  ##  ##  ##  ##  ##  ##  ##  ##  ##  ##  ##  ##  ##  ##  ##  ##  ##  ##  ##  ##  ##  ##  ##  ##  ##  ##  ##  ##  ##  ##  ##  ##  ##  ##  ##  ##  ##  ##  ##  ##  ##  ##  ##  ##  ##  ##  ##  ##  ##  ##  ##  ##  ##  ##  ##  ##  ##  ##  ##  ##  ##  ##  ##  ##  ##  ##  ##  ##  ##  ##  ##  ##  ##  ##  ##  ##  ##  ##  ##  ##  ##  ##  ##  ##  ##  ##  ##  ##  ##  ##  ##  ##  ##  ##  ##  ##  ##  ##  ##  ##  ##  ##  ##  ##  ##  ##  ##  ##  ##  ##  ##  ##  ##  ##  ##  ##  ##  ##  ##  ##  ##  ##  ##  ##  ##  ##  ##  ##  ##  ##  ##  ##  ##  ##  ##  ##  ##  ##  ##  ##  ##  ##  ##  ##  ##  ##  ##  ##  ##  ##  ##  ##  ##  ##  ##  ##  ##  ##  ##  ##  ##  ##  ##  ##  ##  ##  ##  ##  ##  ##  ##  ##  ##  ##  ##  ##  ##  ##  ##  ##  ##  ##  ##  ##  ##  ##  ##  ##  ##  ## [2381_2]23	: 7  3  3  4  3  4  3  3  2  7  4  3  3  4  8  3  5  7  3  4  12  3  --  3  6  ##  ##  ##  ##  ##  ##  ##  ##  ##  ##  ##  ##  ##  ##  ##  ##  ##  ##  ##  ##  ##  ##  ##  ##  ##  ##  ##  ##  ##  ##  ##  ##  ##  ##  ##  ##  ##  ##  ##  ##  ##  ##  ##  ##  ##  ##  ##  ##  ##  ##  ##  ##  ##  ##  ##  ##  ##  ##  ##  ##  ##  ##  ##  ##  ##  ##  ##  ##  ##  ##  ##  ##  ##  ##  ##  ##  ##  ##  ##  ##  ##  ##  ##  ##  ##  ##  ##  ##  ##  ##  ##  ##  ##  ##  ##  ##  ##  ##  ##  ##  ##  ##  ##  ##  ##  ##  ##  ##  ##  ##  ##  ##  ##  ##  ##  ##  ##  ##  ##  ##  ##  ##  ##  ##  ##  ##  ##  ##  ##  ##  ##  ##  ##  ##  ##  ##  ##  ##  ##  ##  ##  ##  ##  ##  ##  ##  ##  ##  ##  ##  ##  ##  ##  ##  ##  ##  ##  ##  ##  ##  ##  ##  ##  ##  ##  ##  ##  ##  ##  ##  ##  ##  ##  ##  ##  ##  ##  ##  ##  ##  ##  ##  ##  ##  ##  ##  ##  ##  ##  ##  ##  ##  ##  ##  ##  ##  ##  ##  ##  ##  ##  ##  ##  ##  ##  ##  ##  ##  ##  ##  ##  ##  ##  ##  ##  ##  ##  ##  ##  ##  ##  ##  ##  ##  ##  ##  ##  ##  ##  ##  ##  ##  ##  ##  ##  ##  ##  ##  ##  ##  ##  ##  ##  ##  ## [1987_2]24	: 6  2  2  3  2  3  2  2  1  6  3  2  2  3  7  2  4  6  2  3  11  2  3  --  6  ##  ##  ##  ##  ##  ##  ##  ##  ##  ##  ##  ##  ##  ##  ##  ##  ##  ##  ##  ##  ##  ##  ##  ##  ##  ##  ##  ##  ##  ##  ##  ##  ##  ##  ##  ##  ##  ##  ##  ##  ##  ##  ##  ##  ##  ##  ##  ##  ##  ##  ##  ##  ##  ##  ##  ##  ##  ##  ##  ##  ##  ##  ##  ##  ##  ##  ##  ##  ##  ##  ##  ##  ##  ##  ##  ##  ##  ##  ##  ##  ##  ##  ##  ##  ##  ##  ##  ##  ##  ##  ##  ##  ##  ##  ##  ##  ##  ##  ##  ##  ##  ##  ##  ##  ##  ##  ##  ##  ##  ##  ##  ##  ##  ##  ##  ##  ##  ##  ##  ##  ##  ##  ##  ##  ##  ##  ##  ##  ##  ##  ##  ##  ##  ##  ##  ##  ##  ##  ##  ##  ##  ##  ##  ##  ##  ##  ##  ##  ##  ##  ##  ##  ##  ##  ##  ##  ##  ##  ##  ##  ##  ##  ##  ##  ##  ##  ##  ##  ##  ##  ##  ##  ##  ##  ##  ##  ##  ##  ##  ##  ##  ##  ##  ##  ##  ##  ##  ##  ##  ##  ##  ##  ##  ##  ##  ##  ##  ##  ##  ##  ##  ##  ##  ##  ##  ##  ##  ##  ##  ##  ##  ##  ##  ##  ##  ##  ##  ##  ##  ##  ##  ##  ##  ##  ##  ##  ##  ##  ##  ##  ##  ##  ##  ##  ##  ##  ##  ##  ##  ##  ##  ##  ##  ##  ## [POLYNOR088_13_2]25	: 3  5  5  6  6  6  4  6  5  1  6  6  5  6  2  5  7  10  5  6  8  5  6  6  --  ##  ##  ##  ##  ##  ##  ##  ##  ##  ##  ##  ##  ##  ##  ##  ##  ##  ##  ##  ##  ##  ##  ##  ##  ##  ##  ##  ##  ##  ##  ##  ##  ##  ##  ##  ##  ##  ##  ##  ##  ##  ##  ##  ##  ##  ##  ##  ##  ##  ##  ##  ##  ##  ##  ##  ##  ##  ##  ##  ##  ##  ##  ##  ##  ##  ##  ##  ##  ##  ##  ##  ##  ##  ##  ##  ##  ##  ##  ##  ##  ##  ##  ##  ##  ##  ##  ##  ##  ##  ##  ##  ##  ##  ##  ##  ##  ##  ##  ##  ##  ##  ##  ##  ##  ##  ##  ##  ##  ##  ##  ##  ##  ##  ##  ##  ##  ##  ##  ##  ##  ##  ##  ##  ##  ##  ##  ##  ##  ##  ##  ##  ##  ##  ##  ##  ##  ##  ##  ##  ##  ##  ##  ##  ##  ##  ##  ##  ##  ##  ##  ##  ##  ##  ##  ##  ##  ##  ##  ##  ##  ##  ##  ##  ##  ##  ##  ##  ##  ##  ##  ##  ##  ##  ##  ##  ##  ##  ##  ##  ##  ##  ##  ##  ##  ##  ##  ##  ##  ##  ##  ##  ##  ##  ##  ##  ##  ##  ##  ##  ##  ##  ##  ##  ##  ##  ##  ##  ##  ##  ##  ##  ##  ##  ##  ##  ##  ##  ##  ##  ##  ##  ##  ##  ##  ##  ##  ##  ##  ##  ##  ##  ##  ##  ##  ##  ##  ##  ##  ##  ##  ##  ##  ##  ##  ## [1207_3]26	: ##  ##  ##  ##  ##  ##  ##  ##  ##  ##  ##  ##  ##  ##  ##  ##  ##  ##  ##  ##  ##  ##  ##  ##  ##  --  7  6  11  7  9  9  7  1  7  8  7  8  6  5  4  5  9  6  6  7  7  7  8  5  7  10  5  7  6  6  8  5  4  7  7  7  7  7  11  7  7  5  9  ##  ##  ##  ##  ##  ##  ##  ##  ##  ##  ##  ##  ##  ##  ##  ##  ##  ##  ##  ##  ##  ##  ##  ##  ##  ##  ##  ##  ##  ##  ##  ##  ##  ##  ##  ##  ##  ##  ##  ##  ##  ##  ##  ##  ##  ##  ##  ##  ##  ##  ##  ##  ##  ##  ##  ##  ##  ##  ##  ##  ##  ##  ##  ##  ##  ##  ##  ##  ##  ##  ##  ##  ##  ##  ##  ##  ##  ##  ##  ##  ##  ##  ##  ##  ##  ##  ##  ##  ##  ##  ##  ##  ##  ##  ##  ##  ##  ##  ##  ##  ##  ##  ##  ##  ##  ##  ##  ##  ##  ##  ##  ##  ##  ##  ##  ##  ##  ##  ##  ##  ##  ##  ##  ##  ##  ##  ##  ##  ##  ##  ##  ##  ##  ##  ##  ##  ##  ##  ##  ##  ##  ##  ##  ##  ##  ##  ##  ##  ##  ##  ##  ##  ##  ##  ##  ##  ##  ##  ##  ##  ##  ##  ##  ##  ##  ##  ##  ##  ##  ##  ##  ##  ##  ##  ##  ##  ##  ##  ##  ##  ##  ##  ##  ##  ##  ##  ##  ##  ##  ##  ##  ##  ##  ##  ##  ##  ##  ##  ##  ##  ## [1310_3]27	: ##  ##  ##  ##  ##  ##  ##  ##  ##  ##  ##  ##  ##  ##  ##  ##  ##  ##  ##  ##  ##  ##  ##  ##  ##  7  --  7  10  8  10  10  8  6  8  7  8  7  7  4  5  6  8  7  3  8  8  6  3  6  8  7  4  6  5  7  7  2  5  8  6  8  8  6  8  6  8  6  6  ##  ##  ##  ##  ##  ##  ##  ##  ##  ##  ##  ##  ##  ##  ##  ##  ##  ##  ##  ##  ##  ##  ##  ##  ##  ##  ##  ##  ##  ##  ##  ##  ##  ##  ##  ##  ##  ##  ##  ##  ##  ##  ##  ##  ##  ##  ##  ##  ##  ##  ##  ##  ##  ##  ##  ##  ##  ##  ##  ##  ##  ##  ##  ##  ##  ##  ##  ##  ##  ##  ##  ##  ##  ##  ##  ##  ##  ##  ##  ##  ##  ##  ##  ##  ##  ##  ##  ##  ##  ##  ##  ##  ##  ##  ##  ##  ##  ##  ##  ##  ##  ##  ##  ##  ##  ##  ##  ##  ##  ##  ##  ##  ##  ##  ##  ##  ##  ##  ##  ##  ##  ##  ##  ##  ##  ##  ##  ##  ##  ##  ##  ##  ##  ##  ##  ##  ##  ##  ##  ##  ##  ##  ##  ##  ##  ##  ##  ##  ##  ##  ##  ##  ##  ##  ##  ##  ##  ##  ##  ##  ##  ##  ##  ##  ##  ##  ##  ##  ##  ##  ##  ##  ##  ##  ##  ##  ##  ##  ##  ##  ##  ##  ##  ##  ##  ##  ##  ##  ##  ##  ##  ##  ##  ##  ##  ##  ##  ##  ##  ##  ## [2275_3]28	: ##  ##  ##  ##  ##  ##  ##  ##  ##  ##  ##  ##  ##  ##  ##  ##  ##  ##  ##  ##  ##  ##  ##  ##  ##  6  7  --  11  7  9  9  1  5  3  8  1  8  8  5  6  5  3  4  6  1  1  7  8  1  1  10  5  7  6  2  6  5  4  3  7  1  1  1  11  7  1  5  9  ##  ##  ##  ##  ##  ##  ##  ##  ##  ##  ##  ##  ##  ##  ##  ##  ##  ##  ##  ##  ##  ##  ##  ##  ##  ##  ##  ##  ##  ##  ##  ##  ##  ##  ##  ##  ##  ##  ##  ##  ##  ##  ##  ##  ##  ##  ##  ##  ##  ##  ##  ##  ##  ##  ##  ##  ##  ##  ##  ##  ##  ##  ##  ##  ##  ##  ##  ##  ##  ##  ##  ##  ##  ##  ##  ##  ##  ##  ##  ##  ##  ##  ##  ##  ##  ##  ##  ##  ##  ##  ##  ##  ##  ##  ##  ##  ##  ##  ##  ##  ##  ##  ##  ##  ##  ##  ##  ##  ##  ##  ##  ##  ##  ##  ##  ##  ##  ##  ##  ##  ##  ##  ##  ##  ##  ##  ##  ##  ##  ##  ##  ##  ##  ##  ##  ##  ##  ##  ##  ##  ##  ##  ##  ##  ##  ##  ##  ##  ##  ##  ##  ##  ##  ##  ##  ##  ##  ##  ##  ##  ##  ##  ##  ##  ##  ##  ##  ##  ##  ##  ##  ##  ##  ##  ##  ##  ##  ##  ##  ##  ##  ##  ##  ##  ##  ##  ##  ##  ##  ##  ##  ##  ##  ##  ##  ##  ##  ##  ##  ##  ## [2380_3]29	: ##  ##  ##  ##  ##  ##  ##  ##  ##  ##  ##  ##  ##  ##  ##  ##  ##  ##  ##  ##  ##  ##  ##  ##  ##  11  10  11  --  6  6  8  12  10  12  9  12  9  13  10  11  8  14  11  9  12  12  12  11  10  10  11  10  12  11  11  13  10  9  11  8  12  12  12  12  12  12  10  10  ##  ##  ##  ##  ##  ##  ##  ##  ##  ##  ##  ##  ##  ##  ##  ##  ##  ##  ##  ##  ##  ##  ##  ##  ##  ##  ##  ##  ##  ##  ##  ##  ##  ##  ##  ##  ##  ##  ##  ##  ##  ##  ##  ##  ##  ##  ##  ##  ##  ##  ##  ##  ##  ##  ##  ##  ##  ##  ##  ##  ##  ##  ##  ##  ##  ##  ##  ##  ##  ##  ##  ##  ##  ##  ##  ##  ##  ##  ##  ##  ##  ##  ##  ##  ##  ##  ##  ##  ##  ##  ##  ##  ##  ##  ##  ##  ##  ##  ##  ##  ##  ##  ##  ##  ##  ##  ##  ##  ##  ##  ##  ##  ##  ##  ##  ##  ##  ##  ##  ##  ##  ##  ##  ##  ##  ##  ##  ##  ##  ##  ##  ##  ##  ##  ##  ##  ##  ##  ##  ##  ##  ##  ##  ##  ##  ##  ##  ##  ##  ##  ##  ##  ##  ##  ##  ##  ##  ##  ##  ##  ##  ##  ##  ##  ##  ##  ##  ##  ##  ##  ##  ##  ##  ##  ##  ##  ##  ##  ##  ##  ##  ##  ##  ##  ##  ##  ##  ##  ##  ##  ##  ##  ##  ##  ##  ##  ##  ##  ##  ##  ## [2463_3]30	: ##  ##  ##  ##  ##  ##  ##  ##  ##  ##  ##  ##  ##  ##  ##  ##  ##  ##  ##  ##  ##  ##  ##  ##  ##  7  8  7  6  --  4  4  8  6  8  9  8  9  9  6  7  6  10  7  7  8  8  8  9  6  8  9  6  8  7  7  9  6  5  8  8  8  8  8  12  8  8  6  10  ##  ##  ##  ##  ##  ##  ##  ##  ##  ##  ##  ##  ##  ##  ##  ##  ##  ##  ##  ##  ##  ##  ##  ##  ##  ##  ##  ##  ##  ##  ##  ##  ##  ##  ##  ##  ##  ##  ##  ##  ##  ##  ##  ##  ##  ##  ##  ##  ##  ##  ##  ##  ##  ##  ##  ##  ##  ##  ##  ##  ##  ##  ##  ##  ##  ##  ##  ##  ##  ##  ##  ##  ##  ##  ##  ##  ##  ##  ##  ##  ##  ##  ##  ##  ##  ##  ##  ##  ##  ##  ##  ##  ##  ##  ##  ##  ##  ##  ##  ##  ##  ##  ##  ##  ##  ##  ##  ##  ##  ##  ##  ##  ##  ##  ##  ##  ##  ##  ##  ##  ##  ##  ##  ##  ##  ##  ##  ##  ##  ##  ##  ##  ##  ##  ##  ##  ##  ##  ##  ##  ##  ##  ##  ##  ##  ##  ##  ##  ##  ##  ##  ##  ##  ##  ##  ##  ##  ##  ##  ##  ##  ##  ##  ##  ##  ##  ##  ##  ##  ##  ##  ##  ##  ##  ##  ##  ##  ##  ##  ##  ##  ##  ##  ##  ##  ##  ##  ##  ##  ##  ##  ##  ##  ##  ##  ##  ##  ##  ##  ##  ## [2464_3]31	: ##  ##  ##  ##  ##  ##  ##  ##  ##  ##  ##  ##  ##  ##  ##  ##  ##  ##  ##  ##  ##  ##  ##  ##  ##  9  10  9  6  4  --  4  10  8  10  11  10  11  11  8  9  8  12  9  9  10  10  8  9  8  10  9  8  10  9  9  11  8  7  10  10  10  10  10  14  10  10  8  12  ##  ##  ##  ##  ##  ##  ##  ##  ##  ##  ##  ##  ##  ##  ##  ##  ##  ##  ##  ##  ##  ##  ##  ##  ##  ##  ##  ##  ##  ##  ##  ##  ##  ##  ##  ##  ##  ##  ##  ##  ##  ##  ##  ##  ##  ##  ##  ##  ##  ##  ##  ##  ##  ##  ##  ##  ##  ##  ##  ##  ##  ##  ##  ##  ##  ##  ##  ##  ##  ##  ##  ##  ##  ##  ##  ##  ##  ##  ##  ##  ##  ##  ##  ##  ##  ##  ##  ##  ##  ##  ##  ##  ##  ##  ##  ##  ##  ##  ##  ##  ##  ##  ##  ##  ##  ##  ##  ##  ##  ##  ##  ##  ##  ##  ##  ##  ##  ##  ##  ##  ##  ##  ##  ##  ##  ##  ##  ##  ##  ##  ##  ##  ##  ##  ##  ##  ##  ##  ##  ##  ##  ##  ##  ##  ##  ##  ##  ##  ##  ##  ##  ##  ##  ##  ##  ##  ##  ##  ##  ##  ##  ##  ##  ##  ##  ##  ##  ##  ##  ##  ##  ##  ##  ##  ##  ##  ##  ##  ##  ##  ##  ##  ##  ##  ##  ##  ##  ##  ##  ##  ##  ##  ##  ##  ##  ##  ##  ##  ##  ##  ## [2465_3]32	: ##  ##  ##  ##  ##  ##  ##  ##  ##  ##  ##  ##  ##  ##  ##  ##  ##  ##  ##  ##  ##  ##  ##  ##  ##  9  10  9  8  4  4  --  10  8  8  11  10  11  11  8  9  8  12  7  9  10  10  8  11  8  10  9  8  10  7  9  11  8  7  10  10  10  10  10  14  10  10  8  12  ##  ##  ##  ##  ##  ##  ##  ##  ##  ##  ##  ##  ##  ##  ##  ##  ##  ##  ##  ##  ##  ##  ##  ##  ##  ##  ##  ##  ##  ##  ##  ##  ##  ##  ##  ##  ##  ##  ##  ##  ##  ##  ##  ##  ##  ##  ##  ##  ##  ##  ##  ##  ##  ##  ##  ##  ##  ##  ##  ##  ##  ##  ##  ##  ##  ##  ##  ##  ##  ##  ##  ##  ##  ##  ##  ##  ##  ##  ##  ##  ##  ##  ##  ##  ##  ##  ##  ##  ##  ##  ##  ##  ##  ##  ##  ##  ##  ##  ##  ##  ##  ##  ##  ##  ##  ##  ##  ##  ##  ##  ##  ##  ##  ##  ##  ##  ##  ##  ##  ##  ##  ##  ##  ##  ##  ##  ##  ##  ##  ##  ##  ##  ##  ##  ##  ##  ##  ##  ##  ##  ##  ##  ##  ##  ##  ##  ##  ##  ##  ##  ##  ##  ##  ##  ##  ##  ##  ##  ##  ##  ##  ##  ##  ##  ##  ##  ##  ##  ##  ##  ##  ##  ##  ##  ##  ##  ##  ##  ##  ##  ##  ##  ##  ##  ##  ##  ##  ##  ##  ##  ##  ##  ##  ##  ##  ##  ##  ##  ##  ##  ## [2812_3]33	: ##  ##  ##  ##  ##  ##  ##  ##  ##  ##  ##  ##  ##  ##  ##  ##  ##  ##  ##  ##  ##  ##  ##  ##  ##  7  8  1  12  8  10  10  --  6  4  9  2  9  9  6  7  6  4  5  7  2  2  8  9  2  2  11  6  8  7  3  7  6  5  4  8  2  2  2  12  8  2  6  10  ##  ##  ##  ##  ##  ##  ##  ##  ##  ##  ##  ##  ##  ##  ##  ##  ##  ##  ##  ##  ##  ##  ##  ##  ##  ##  ##  ##  ##  ##  ##  ##  ##  ##  ##  ##  ##  ##  ##  ##  ##  ##  ##  ##  ##  ##  ##  ##  ##  ##  ##  ##  ##  ##  ##  ##  ##  ##  ##  ##  ##  ##  ##  ##  ##  ##  ##  ##  ##  ##  ##  ##  ##  ##  ##  ##  ##  ##  ##  ##  ##  ##  ##  ##  ##  ##  ##  ##  ##  ##  ##  ##  ##  ##  ##  ##  ##  ##  ##  ##  ##  ##  ##  ##  ##  ##  ##  ##  ##  ##  ##  ##  ##  ##  ##  ##  ##  ##  ##  ##  ##  ##  ##  ##  ##  ##  ##  ##  ##  ##  ##  ##  ##  ##  ##  ##  ##  ##  ##  ##  ##  ##  ##  ##  ##  ##  ##  ##  ##  ##  ##  ##  ##  ##  ##  ##  ##  ##  ##  ##  ##  ##  ##  ##  ##  ##  ##  ##  ##  ##  ##  ##  ##  ##  ##  ##  ##  ##  ##  ##  ##  ##  ##  ##  ##  ##  ##  ##  ##  ##  ##  ##  ##  ##  ##  ##  ##  ##  ##  ##  ## [2814_3]34	: ##  ##  ##  ##  ##  ##  ##  ##  ##  ##  ##  ##  ##  ##  ##  ##  ##  ##  ##  ##  ##  ##  ##  ##  ##  1  6  5  10  6  8  8  6  --  6  7  6  7  7  4  3  4  8  5  5  6  6  6  7  4  6  9  4  6  5  5  7  4  3  6  6  6  6  6  10  6  6  4  8  ##  ##  ##  ##  ##  ##  ##  ##  ##  ##  ##  ##  ##  ##  ##  ##  ##  ##  ##  ##  ##  ##  ##  ##  ##  ##  ##  ##  ##  ##  ##  ##  ##  ##  ##  ##  ##  ##  ##  ##  ##  ##  ##  ##  ##  ##  ##  ##  ##  ##  ##  ##  ##  ##  ##  ##  ##  ##  ##  ##  ##  ##  ##  ##  ##  ##  ##  ##  ##  ##  ##  ##  ##  ##  ##  ##  ##  ##  ##  ##  ##  ##  ##  ##  ##  ##  ##  ##  ##  ##  ##  ##  ##  ##  ##  ##  ##  ##  ##  ##  ##  ##  ##  ##  ##  ##  ##  ##  ##  ##  ##  ##  ##  ##  ##  ##  ##  ##  ##  ##  ##  ##  ##  ##  ##  ##  ##  ##  ##  ##  ##  ##  ##  ##  ##  ##  ##  ##  ##  ##  ##  ##  ##  ##  ##  ##  ##  ##  ##  ##  ##  ##  ##  ##  ##  ##  ##  ##  ##  ##  ##  ##  ##  ##  ##  ##  ##  ##  ##  ##  ##  ##  ##  ##  ##  ##  ##  ##  ##  ##  ##  ##  ##  ##  ##  ##  ##  ##  ##  ##  ##  ##  ##  ##  ##  ##  ##  ##  ##  ##  ## [2872_3]35	: ##  ##  ##  ##  ##  ##  ##  ##  ##  ##  ##  ##  ##  ##  ##  ##  ##  ##  ##  ##  ##  ##  ##  ##  ##  7  8  3  12  8  10  8  4  6  --  9  4  9  9  6  7  6  6  5  7  4  4  8  9  2  4  11  6  8  7  3  9  6  5  4  8  4  4  4  12  8  4  6  10  ##  ##  ##  ##  ##  ##  ##  ##  ##  ##  ##  ##  ##  ##  ##  ##  ##  ##  ##  ##  ##  ##  ##  ##  ##  ##  ##  ##  ##  ##  ##  ##  ##  ##  ##  ##  ##  ##  ##  ##  ##  ##  ##  ##  ##  ##  ##  ##  ##  ##  ##  ##  ##  ##  ##  ##  ##  ##  ##  ##  ##  ##  ##  ##  ##  ##  ##  ##  ##  ##  ##  ##  ##  ##  ##  ##  ##  ##  ##  ##  ##  ##  ##  ##  ##  ##  ##  ##  ##  ##  ##  ##  ##  ##  ##  ##  ##  ##  ##  ##  ##  ##  ##  ##  ##  ##  ##  ##  ##  ##  ##  ##  ##  ##  ##  ##  ##  ##  ##  ##  ##  ##  ##  ##  ##  ##  ##  ##  ##  ##  ##  ##  ##  ##  ##  ##  ##  ##  ##  ##  ##  ##  ##  ##  ##  ##  ##  ##  ##  ##  ##  ##  ##  ##  ##  ##  ##  ##  ##  ##  ##  ##  ##  ##  ##  ##  ##  ##  ##  ##  ##  ##  ##  ##  ##  ##  ##  ##  ##  ##  ##  ##  ##  ##  ##  ##  ##  ##  ##  ##  ##  ##  ##  ##  ##  ##  ##  ##  ##  ##  ## [2874_3]36	: ##  ##  ##  ##  ##  ##  ##  ##  ##  ##  ##  ##  ##  ##  ##  ##  ##  ##  ##  ##  ##  ##  ##  ##  ##  8  7  8  9  9  11  11  9  7  9  --  9  4  10  7  8  5  11  8  6  9  9  9  6  7  9  10  7  9  8  8  10  7  6  9  3  9  9  9  3  9  9  7  5  ##  ##  ##  ##  ##  ##  ##  ##  ##  ##  ##  ##  ##  ##  ##  ##  ##  ##  ##  ##  ##  ##  ##  ##  ##  ##  ##  ##  ##  ##  ##  ##  ##  ##  ##  ##  ##  ##  ##  ##  ##  ##  ##  ##  ##  ##  ##  ##  ##  ##  ##  ##  ##  ##  ##  ##  ##  ##  ##  ##  ##  ##  ##  ##  ##  ##  ##  ##  ##  ##  ##  ##  ##  ##  ##  ##  ##  ##  ##  ##  ##  ##  ##  ##  ##  ##  ##  ##  ##  ##  ##  ##  ##  ##  ##  ##  ##  ##  ##  ##  ##  ##  ##  ##  ##  ##  ##  ##  ##  ##  ##  ##  ##  ##  ##  ##  ##  ##  ##  ##  ##  ##  ##  ##  ##  ##  ##  ##  ##  ##  ##  ##  ##  ##  ##  ##  ##  ##  ##  ##  ##  ##  ##  ##  ##  ##  ##  ##  ##  ##  ##  ##  ##  ##  ##  ##  ##  ##  ##  ##  ##  ##  ##  ##  ##  ##  ##  ##  ##  ##  ##  ##  ##  ##  ##  ##  ##  ##  ##  ##  ##  ##  ##  ##  ##  ##  ##  ##  ##  ##  ##  ##  ##  ##  ##  ##  ##  ##  ##  ##  ## [2876_3]37	: ##  ##  ##  ##  ##  ##  ##  ##  ##  ##  ##  ##  ##  ##  ##  ##  ##  ##  ##  ##  ##  ##  ##  ##  ##  7  8  1  12  8  10  10  2  6  4  9  --  9  9  6  7  6  4  5  7  2  2  8  9  2  2  11  6  8  7  3  7  6  5  4  8  2  2  2  12  8  2  6  10  ##  ##  ##  ##  ##  ##  ##  ##  ##  ##  ##  ##  ##  ##  ##  ##  ##  ##  ##  ##  ##  ##  ##  ##  ##  ##  ##  ##  ##  ##  ##  ##  ##  ##  ##  ##  ##  ##  ##  ##  ##  ##  ##  ##  ##  ##  ##  ##  ##  ##  ##  ##  ##  ##  ##  ##  ##  ##  ##  ##  ##  ##  ##  ##  ##  ##  ##  ##  ##  ##  ##  ##  ##  ##  ##  ##  ##  ##  ##  ##  ##  ##  ##  ##  ##  ##  ##  ##  ##  ##  ##  ##  ##  ##  ##  ##  ##  ##  ##  ##  ##  ##  ##  ##  ##  ##  ##  ##  ##  ##  ##  ##  ##  ##  ##  ##  ##  ##  ##  ##  ##  ##  ##  ##  ##  ##  ##  ##  ##  ##  ##  ##  ##  ##  ##  ##  ##  ##  ##  ##  ##  ##  ##  ##  ##  ##  ##  ##  ##  ##  ##  ##  ##  ##  ##  ##  ##  ##  ##  ##  ##  ##  ##  ##  ##  ##  ##  ##  ##  ##  ##  ##  ##  ##  ##  ##  ##  ##  ##  ##  ##  ##  ##  ##  ##  ##  ##  ##  ##  ##  ##  ##  ##  ##  ##  ##  ##  ##  ##  ##  ## [2877_3]38	: ##  ##  ##  ##  ##  ##  ##  ##  ##  ##  ##  ##  ##  ##  ##  ##  ##  ##  ##  ##  ##  ##  ##  ##  ##  8  7  8  9  9  11  11  9  7  9  4  9  --  10  7  8  5  11  8  6  9  9  9  8  7  9  10  7  9  8  8  10  7  6  9  1  9  9  9  7  9  9  7  5  ##  ##  ##  ##  ##  ##  ##  ##  ##  ##  ##  ##  ##  ##  ##  ##  ##  ##  ##  ##  ##  ##  ##  ##  ##  ##  ##  ##  ##  ##  ##  ##  ##  ##  ##  ##  ##  ##  ##  ##  ##  ##  ##  ##  ##  ##  ##  ##  ##  ##  ##  ##  ##  ##  ##  ##  ##  ##  ##  ##  ##  ##  ##  ##  ##  ##  ##  ##  ##  ##  ##  ##  ##  ##  ##  ##  ##  ##  ##  ##  ##  ##  ##  ##  ##  ##  ##  ##  ##  ##  ##  ##  ##  ##  ##  ##  ##  ##  ##  ##  ##  ##  ##  ##  ##  ##  ##  ##  ##  ##  ##  ##  ##  ##  ##  ##  ##  ##  ##  ##  ##  ##  ##  ##  ##  ##  ##  ##  ##  ##  ##  ##  ##  ##  ##  ##  ##  ##  ##  ##  ##  ##  ##  ##  ##  ##  ##  ##  ##  ##  ##  ##  ##  ##  ##  ##  ##  ##  ##  ##  ##  ##  ##  ##  ##  ##  ##  ##  ##  ##  ##  ##  ##  ##  ##  ##  ##  ##  ##  ##  ##  ##  ##  ##  ##  ##  ##  ##  ##  ##  ##  ##  ##  ##  ##  ##  ##  ##  ##  ##  ## [2879_3]39	: ##  ##  ##  ##  ##  ##  ##  ##  ##  ##  ##  ##  ##  ##  ##  ##  ##  ##  ##  ##  ##  ##  ##  ##  ##  6  7  8  13  9  11  11  9  7  9  10  9  10  --  7  8  5  11  6  6  9  9  5  8  7  9  12  3  3  8  8  6  5  6  9  9  9  9  9  13  5  9  7  11  ##  ##  ##  ##  ##  ##  ##  ##  ##  ##  ##  ##  ##  ##  ##  ##  ##  ##  ##  ##  ##  ##  ##  ##  ##  ##  ##  ##  ##  ##  ##  ##  ##  ##  ##  ##  ##  ##  ##  ##  ##  ##  ##  ##  ##  ##  ##  ##  ##  ##  ##  ##  ##  ##  ##  ##  ##  ##  ##  ##  ##  ##  ##  ##  ##  ##  ##  ##  ##  ##  ##  ##  ##  ##  ##  ##  ##  ##  ##  ##  ##  ##  ##  ##  ##  ##  ##  ##  ##  ##  ##  ##  ##  ##  ##  ##  ##  ##  ##  ##  ##  ##  ##  ##  ##  ##  ##  ##  ##  ##  ##  ##  ##  ##  ##  ##  ##  ##  ##  ##  ##  ##  ##  ##  ##  ##  ##  ##  ##  ##  ##  ##  ##  ##  ##  ##  ##  ##  ##  ##  ##  ##  ##  ##  ##  ##  ##  ##  ##  ##  ##  ##  ##  ##  ##  ##  ##  ##  ##  ##  ##  ##  ##  ##  ##  ##  ##  ##  ##  ##  ##  ##  ##  ##  ##  ##  ##  ##  ##  ##  ##  ##  ##  ##  ##  ##  ##  ##  ##  ##  ##  ##  ##  ##  ##  ##  ##  ##  ##  ##  ## [2880_3]40	: ##  ##  ##  ##  ##  ##  ##  ##  ##  ##  ##  ##  ##  ##  ##  ##  ##  ##  ##  ##  ##  ##  ##  ##  ##  5  4  5  10  6  8  8  6  4  6  7  6  7  7  --  3  4  6  5  5  6  6  6  5  4  6  7  4  6  5  5  7  2  3  6  6  6  6  4  8  6  6  4  6  ##  ##  ##  ##  ##  ##  ##  ##  ##  ##  ##  ##  ##  ##  ##  ##  ##  ##  ##  ##  ##  ##  ##  ##  ##  ##  ##  ##  ##  ##  ##  ##  ##  ##  ##  ##  ##  ##  ##  ##  ##  ##  ##  ##  ##  ##  ##  ##  ##  ##  ##  ##  ##  ##  ##  ##  ##  ##  ##  ##  ##  ##  ##  ##  ##  ##  ##  ##  ##  ##  ##  ##  ##  ##  ##  ##  ##  ##  ##  ##  ##  ##  ##  ##  ##  ##  ##  ##  ##  ##  ##  ##  ##  ##  ##  ##  ##  ##  ##  ##  ##  ##  ##  ##  ##  ##  ##  ##  ##  ##  ##  ##  ##  ##  ##  ##  ##  ##  ##  ##  ##  ##  ##  ##  ##  ##  ##  ##  ##  ##  ##  ##  ##  ##  ##  ##  ##  ##  ##  ##  ##  ##  ##  ##  ##  ##  ##  ##  ##  ##  ##  ##  ##  ##  ##  ##  ##  ##  ##  ##  ##  ##  ##  ##  ##  ##  ##  ##  ##  ##  ##  ##  ##  ##  ##  ##  ##  ##  ##  ##  ##  ##  ##  ##  ##  ##  ##  ##  ##  ##  ##  ##  ##  ##  ##  ##  ##  ##  ##  ##  ## [2881_3]41	: ##  ##  ##  ##  ##  ##  ##  ##  ##  ##  ##  ##  ##  ##  ##  ##  ##  ##  ##  ##  ##  ##  ##  ##  ##  4  5  6  11  7  9  9  7  3  7  8  7  8  8  3  --  5  7  6  6  7  7  7  6  5  7  8  5  7  6  6  8  3  4  7  7  7  7  5  9  7  7  3  7  ##  ##  ##  ##  ##  ##  ##  ##  ##  ##  ##  ##  ##  ##  ##  ##  ##  ##  ##  ##  ##  ##  ##  ##  ##  ##  ##  ##  ##  ##  ##  ##  ##  ##  ##  ##  ##  ##  ##  ##  ##  ##  ##  ##  ##  ##  ##  ##  ##  ##  ##  ##  ##  ##  ##  ##  ##  ##  ##  ##  ##  ##  ##  ##  ##  ##  ##  ##  ##  ##  ##  ##  ##  ##  ##  ##  ##  ##  ##  ##  ##  ##  ##  ##  ##  ##  ##  ##  ##  ##  ##  ##  ##  ##  ##  ##  ##  ##  ##  ##  ##  ##  ##  ##  ##  ##  ##  ##  ##  ##  ##  ##  ##  ##  ##  ##  ##  ##  ##  ##  ##  ##  ##  ##  ##  ##  ##  ##  ##  ##  ##  ##  ##  ##  ##  ##  ##  ##  ##  ##  ##  ##  ##  ##  ##  ##  ##  ##  ##  ##  ##  ##  ##  ##  ##  ##  ##  ##  ##  ##  ##  ##  ##  ##  ##  ##  ##  ##  ##  ##  ##  ##  ##  ##  ##  ##  ##  ##  ##  ##  ##  ##  ##  ##  ##  ##  ##  ##  ##  ##  ##  ##  ##  ##  ##  ##  ##  ##  ##  ##  ## [2882_3]42	: ##  ##  ##  ##  ##  ##  ##  ##  ##  ##  ##  ##  ##  ##  ##  ##  ##  ##  ##  ##  ##  ##  ##  ##  ##  5  6  5  8  6  8  8  6  4  6  5  6  5  5  4  5  --  8  3  5  6  6  4  7  4  6  9  2  4  5  5  5  4  3  6  4  6  6  6  8  4  6  4  6  ##  ##  ##  ##  ##  ##  ##  ##  ##  ##  ##  ##  ##  ##  ##  ##  ##  ##  ##  ##  ##  ##  ##  ##  ##  ##  ##  ##  ##  ##  ##  ##  ##  ##  ##  ##  ##  ##  ##  ##  ##  ##  ##  ##  ##  ##  ##  ##  ##  ##  ##  ##  ##  ##  ##  ##  ##  ##  ##  ##  ##  ##  ##  ##  ##  ##  ##  ##  ##  ##  ##  ##  ##  ##  ##  ##  ##  ##  ##  ##  ##  ##  ##  ##  ##  ##  ##  ##  ##  ##  ##  ##  ##  ##  ##  ##  ##  ##  ##  ##  ##  ##  ##  ##  ##  ##  ##  ##  ##  ##  ##  ##  ##  ##  ##  ##  ##  ##  ##  ##  ##  ##  ##  ##  ##  ##  ##  ##  ##  ##  ##  ##  ##  ##  ##  ##  ##  ##  ##  ##  ##  ##  ##  ##  ##  ##  ##  ##  ##  ##  ##  ##  ##  ##  ##  ##  ##  ##  ##  ##  ##  ##  ##  ##  ##  ##  ##  ##  ##  ##  ##  ##  ##  ##  ##  ##  ##  ##  ##  ##  ##  ##  ##  ##  ##  ##  ##  ##  ##  ##  ##  ##  ##  ##  ##  ##  ##  ##  ##  ##  ## [2883_3]43	: ##  ##  ##  ##  ##  ##  ##  ##  ##  ##  ##  ##  ##  ##  ##  ##  ##  ##  ##  ##  ##  ##  ##  ##  ##  9  8  3  14  10  12  12  4  8  6  11  4  11  11  6  7  8  --  7  9  4  4  10  9  4  4  11  8  10  9  5  9  6  7  6  10  4  4  2  12  10  4  8  10  ##  ##  ##  ##  ##  ##  ##  ##  ##  ##  ##  ##  ##  ##  ##  ##  ##  ##  ##  ##  ##  ##  ##  ##  ##  ##  ##  ##  ##  ##  ##  ##  ##  ##  ##  ##  ##  ##  ##  ##  ##  ##  ##  ##  ##  ##  ##  ##  ##  ##  ##  ##  ##  ##  ##  ##  ##  ##  ##  ##  ##  ##  ##  ##  ##  ##  ##  ##  ##  ##  ##  ##  ##  ##  ##  ##  ##  ##  ##  ##  ##  ##  ##  ##  ##  ##  ##  ##  ##  ##  ##  ##  ##  ##  ##  ##  ##  ##  ##  ##  ##  ##  ##  ##  ##  ##  ##  ##  ##  ##  ##  ##  ##  ##  ##  ##  ##  ##  ##  ##  ##  ##  ##  ##  ##  ##  ##  ##  ##  ##  ##  ##  ##  ##  ##  ##  ##  ##  ##  ##  ##  ##  ##  ##  ##  ##  ##  ##  ##  ##  ##  ##  ##  ##  ##  ##  ##  ##  ##  ##  ##  ##  ##  ##  ##  ##  ##  ##  ##  ##  ##  ##  ##  ##  ##  ##  ##  ##  ##  ##  ##  ##  ##  ##  ##  ##  ##  ##  ##  ##  ##  ##  ##  ##  ##  ##  ##  ##  ##  ##  ## [2884_3]44	: ##  ##  ##  ##  ##  ##  ##  ##  ##  ##  ##  ##  ##  ##  ##  ##  ##  ##  ##  ##  ##  ##  ##  ##  ##  6  7  4  11  7  9  7  5  5  5  8  5  8  6  5  6  3  7  --  6  5  5  5  8  5  5  10  3  5  6  6  4  5  4  7  7  5  5  5  11  5  5  5  9  ##  ##  ##  ##  ##  ##  ##  ##  ##  ##  ##  ##  ##  ##  ##  ##  ##  ##  ##  ##  ##  ##  ##  ##  ##  ##  ##  ##  ##  ##  ##  ##  ##  ##  ##  ##  ##  ##  ##  ##  ##  ##  ##  ##  ##  ##  ##  ##  ##  ##  ##  ##  ##  ##  ##  ##  ##  ##  ##  ##  ##  ##  ##  ##  ##  ##  ##  ##  ##  ##  ##  ##  ##  ##  ##  ##  ##  ##  ##  ##  ##  ##  ##  ##  ##  ##  ##  ##  ##  ##  ##  ##  ##  ##  ##  ##  ##  ##  ##  ##  ##  ##  ##  ##  ##  ##  ##  ##  ##  ##  ##  ##  ##  ##  ##  ##  ##  ##  ##  ##  ##  ##  ##  ##  ##  ##  ##  ##  ##  ##  ##  ##  ##  ##  ##  ##  ##  ##  ##  ##  ##  ##  ##  ##  ##  ##  ##  ##  ##  ##  ##  ##  ##  ##  ##  ##  ##  ##  ##  ##  ##  ##  ##  ##  ##  ##  ##  ##  ##  ##  ##  ##  ##  ##  ##  ##  ##  ##  ##  ##  ##  ##  ##  ##  ##  ##  ##  ##  ##  ##  ##  ##  ##  ##  ##  ##  ##  ##  ##  ##  ## [2885_3]45	: ##  ##  ##  ##  ##  ##  ##  ##  ##  ##  ##  ##  ##  ##  ##  ##  ##  ##  ##  ##  ##  ##  ##  ##  ##  6  3  6  9  7  9  9  7  5  7  6  7  6  6  5  6  5  9  6  --  7  7  5  4  5  7  8  3  5  6  6  6  3  4  7  5  7  7  7  9  5  7  5  7  ##  ##  ##  ##  ##  ##  ##  ##  ##  ##  ##  ##  ##  ##  ##  ##  ##  ##  ##  ##  ##  ##  ##  ##  ##  ##  ##  ##  ##  ##  ##  ##  ##  ##  ##  ##  ##  ##  ##  ##  ##  ##  ##  ##  ##  ##  ##  ##  ##  ##  ##  ##  ##  ##  ##  ##  ##  ##  ##  ##  ##  ##  ##  ##  ##  ##  ##  ##  ##  ##  ##  ##  ##  ##  ##  ##  ##  ##  ##  ##  ##  ##  ##  ##  ##  ##  ##  ##  ##  ##  ##  ##  ##  ##  ##  ##  ##  ##  ##  ##  ##  ##  ##  ##  ##  ##  ##  ##  ##  ##  ##  ##  ##  ##  ##  ##  ##  ##  ##  ##  ##  ##  ##  ##  ##  ##  ##  ##  ##  ##  ##  ##  ##  ##  ##  ##  ##  ##  ##  ##  ##  ##  ##  ##  ##  ##  ##  ##  ##  ##  ##  ##  ##  ##  ##  ##  ##  ##  ##  ##  ##  ##  ##  ##  ##  ##  ##  ##  ##  ##  ##  ##  ##  ##  ##  ##  ##  ##  ##  ##  ##  ##  ##  ##  ##  ##  ##  ##  ##  ##  ##  ##  ##  ##  ##  ##  ##  ##  ##  ##  ## [2886_3]46	: ##  ##  ##  ##  ##  ##  ##  ##  ##  ##  ##  ##  ##  ##  ##  ##  ##  ##  ##  ##  ##  ##  ##  ##  ##  7  8  1  12  8  10  10  2  6  4  9  2  9  9  6  7  6  4  5  7  --  2  8  9  2  2  11  6  8  7  3  5  6  5  4  8  2  2  2  10  8  2  6  10  ##  ##  ##  ##  ##  ##  ##  ##  ##  ##  ##  ##  ##  ##  ##  ##  ##  ##  ##  ##  ##  ##  ##  ##  ##  ##  ##  ##  ##  ##  ##  ##  ##  ##  ##  ##  ##  ##  ##  ##  ##  ##  ##  ##  ##  ##  ##  ##  ##  ##  ##  ##  ##  ##  ##  ##  ##  ##  ##  ##  ##  ##  ##  ##  ##  ##  ##  ##  ##  ##  ##  ##  ##  ##  ##  ##  ##  ##  ##  ##  ##  ##  ##  ##  ##  ##  ##  ##  ##  ##  ##  ##  ##  ##  ##  ##  ##  ##  ##  ##  ##  ##  ##  ##  ##  ##  ##  ##  ##  ##  ##  ##  ##  ##  ##  ##  ##  ##  ##  ##  ##  ##  ##  ##  ##  ##  ##  ##  ##  ##  ##  ##  ##  ##  ##  ##  ##  ##  ##  ##  ##  ##  ##  ##  ##  ##  ##  ##  ##  ##  ##  ##  ##  ##  ##  ##  ##  ##  ##  ##  ##  ##  ##  ##  ##  ##  ##  ##  ##  ##  ##  ##  ##  ##  ##  ##  ##  ##  ##  ##  ##  ##  ##  ##  ##  ##  ##  ##  ##  ##  ##  ##  ##  ##  ##  ##  ##  ##  ##  ##  ## [2887_3]47	: ##  ##  ##  ##  ##  ##  ##  ##  ##  ##  ##  ##  ##  ##  ##  ##  ##  ##  ##  ##  ##  ##  ##  ##  ##  7  8  1  12  8  10  10  2  6  4  9  2  9  9  6  7  6  4  5  7  2  --  8  9  2  2  11  6  8  7  3  7  6  5  4  8  2  2  2  12  8  2  6  10  ##  ##  ##  ##  ##  ##  ##  ##  ##  ##  ##  ##  ##  ##  ##  ##  ##  ##  ##  ##  ##  ##  ##  ##  ##  ##  ##  ##  ##  ##  ##  ##  ##  ##  ##  ##  ##  ##  ##  ##  ##  ##  ##  ##  ##  ##  ##  ##  ##  ##  ##  ##  ##  ##  ##  ##  ##  ##  ##  ##  ##  ##  ##  ##  ##  ##  ##  ##  ##  ##  ##  ##  ##  ##  ##  ##  ##  ##  ##  ##  ##  ##  ##  ##  ##  ##  ##  ##  ##  ##  ##  ##  ##  ##  ##  ##  ##  ##  ##  ##  ##  ##  ##  ##  ##  ##  ##  ##  ##  ##  ##  ##  ##  ##  ##  ##  ##  ##  ##  ##  ##  ##  ##  ##  ##  ##  ##  ##  ##  ##  ##  ##  ##  ##  ##  ##  ##  ##  ##  ##  ##  ##  ##  ##  ##  ##  ##  ##  ##  ##  ##  ##  ##  ##  ##  ##  ##  ##  ##  ##  ##  ##  ##  ##  ##  ##  ##  ##  ##  ##  ##  ##  ##  ##  ##  ##  ##  ##  ##  ##  ##  ##  ##  ##  ##  ##  ##  ##  ##  ##  ##  ##  ##  ##  ##  ##  ##  ##  ##  ##  ## [2888_3]48	: ##  ##  ##  ##  ##  ##  ##  ##  ##  ##  ##  ##  ##  ##  ##  ##  ##  ##  ##  ##  ##  ##  ##  ##  ##  7  6  7  12  8  8  8  8  6  8  9  8  9  5  6  7  4  10  5  5  8  8  --  7  6  8  9  2  4  7  7  5  4  5  8  8  8  8  8  12  4  8  6  10  ##  ##  ##  ##  ##  ##  ##  ##  ##  ##  ##  ##  ##  ##  ##  ##  ##  ##  ##  ##  ##  ##  ##  ##  ##  ##  ##  ##  ##  ##  ##  ##  ##  ##  ##  ##  ##  ##  ##  ##  ##  ##  ##  ##  ##  ##  ##  ##  ##  ##  ##  ##  ##  ##  ##  ##  ##  ##  ##  ##  ##  ##  ##  ##  ##  ##  ##  ##  ##  ##  ##  ##  ##  ##  ##  ##  ##  ##  ##  ##  ##  ##  ##  ##  ##  ##  ##  ##  ##  ##  ##  ##  ##  ##  ##  ##  ##  ##  ##  ##  ##  ##  ##  ##  ##  ##  ##  ##  ##  ##  ##  ##  ##  ##  ##  ##  ##  ##  ##  ##  ##  ##  ##  ##  ##  ##  ##  ##  ##  ##  ##  ##  ##  ##  ##  ##  ##  ##  ##  ##  ##  ##  ##  ##  ##  ##  ##  ##  ##  ##  ##  ##  ##  ##  ##  ##  ##  ##  ##  ##  ##  ##  ##  ##  ##  ##  ##  ##  ##  ##  ##  ##  ##  ##  ##  ##  ##  ##  ##  ##  ##  ##  ##  ##  ##  ##  ##  ##  ##  ##  ##  ##  ##  ##  ##  ##  ##  ##  ##  ##  ## [2889_3]49	: ##  ##  ##  ##  ##  ##  ##  ##  ##  ##  ##  ##  ##  ##  ##  ##  ##  ##  ##  ##  ##  ##  ##  ##  ##  8  3  8  11  9  9  11  9  7  9  6  9  8  8  5  6  7  9  8  4  9  9  7  --  7  9  8  5  7  8  8  8  3  6  9  7  9  9  7  7  7  9  7  7  ##  ##  ##  ##  ##  ##  ##  ##  ##  ##  ##  ##  ##  ##  ##  ##  ##  ##  ##  ##  ##  ##  ##  ##  ##  ##  ##  ##  ##  ##  ##  ##  ##  ##  ##  ##  ##  ##  ##  ##  ##  ##  ##  ##  ##  ##  ##  ##  ##  ##  ##  ##  ##  ##  ##  ##  ##  ##  ##  ##  ##  ##  ##  ##  ##  ##  ##  ##  ##  ##  ##  ##  ##  ##  ##  ##  ##  ##  ##  ##  ##  ##  ##  ##  ##  ##  ##  ##  ##  ##  ##  ##  ##  ##  ##  ##  ##  ##  ##  ##  ##  ##  ##  ##  ##  ##  ##  ##  ##  ##  ##  ##  ##  ##  ##  ##  ##  ##  ##  ##  ##  ##  ##  ##  ##  ##  ##  ##  ##  ##  ##  ##  ##  ##  ##  ##  ##  ##  ##  ##  ##  ##  ##  ##  ##  ##  ##  ##  ##  ##  ##  ##  ##  ##  ##  ##  ##  ##  ##  ##  ##  ##  ##  ##  ##  ##  ##  ##  ##  ##  ##  ##  ##  ##  ##  ##  ##  ##  ##  ##  ##  ##  ##  ##  ##  ##  ##  ##  ##  ##  ##  ##  ##  ##  ##  ##  ##  ##  ##  ##  ## [2890_3]50	: ##  ##  ##  ##  ##  ##  ##  ##  ##  ##  ##  ##  ##  ##  ##  ##  ##  ##  ##  ##  ##  ##  ##  ##  ##  5  6  1  10  6  8  8  2  4  2  7  2  7  7  4  5  4  4  5  5  2  2  6  7  --  2  9  4  6  5  1  7  4  3  2  6  2  2  2  10  6  2  4  8  ##  ##  ##  ##  ##  ##  ##  ##  ##  ##  ##  ##  ##  ##  ##  ##  ##  ##  ##  ##  ##  ##  ##  ##  ##  ##  ##  ##  ##  ##  ##  ##  ##  ##  ##  ##  ##  ##  ##  ##  ##  ##  ##  ##  ##  ##  ##  ##  ##  ##  ##  ##  ##  ##  ##  ##  ##  ##  ##  ##  ##  ##  ##  ##  ##  ##  ##  ##  ##  ##  ##  ##  ##  ##  ##  ##  ##  ##  ##  ##  ##  ##  ##  ##  ##  ##  ##  ##  ##  ##  ##  ##  ##  ##  ##  ##  ##  ##  ##  ##  ##  ##  ##  ##  ##  ##  ##  ##  ##  ##  ##  ##  ##  ##  ##  ##  ##  ##  ##  ##  ##  ##  ##  ##  ##  ##  ##  ##  ##  ##  ##  ##  ##  ##  ##  ##  ##  ##  ##  ##  ##  ##  ##  ##  ##  ##  ##  ##  ##  ##  ##  ##  ##  ##  ##  ##  ##  ##  ##  ##  ##  ##  ##  ##  ##  ##  ##  ##  ##  ##  ##  ##  ##  ##  ##  ##  ##  ##  ##  ##  ##  ##  ##  ##  ##  ##  ##  ##  ##  ##  ##  ##  ##  ##  ##  ##  ##  ##  ##  ##  ## [2891_3]51	: ##  ##  ##  ##  ##  ##  ##  ##  ##  ##  ##  ##  ##  ##  ##  ##  ##  ##  ##  ##  ##  ##  ##  ##  ##  7  8  1  10  8  10  10  2  6  4  9  2  9  9  6  7  6  4  5  7  2  2  8  9  2  --  11  6  8  7  3  7  6  5  3  8  2  2  2  12  8  2  6  10  ##  ##  ##  ##  ##  ##  ##  ##  ##  ##  ##  ##  ##  ##  ##  ##  ##  ##  ##  ##  ##  ##  ##  ##  ##  ##  ##  ##  ##  ##  ##  ##  ##  ##  ##  ##  ##  ##  ##  ##  ##  ##  ##  ##  ##  ##  ##  ##  ##  ##  ##  ##  ##  ##  ##  ##  ##  ##  ##  ##  ##  ##  ##  ##  ##  ##  ##  ##  ##  ##  ##  ##  ##  ##  ##  ##  ##  ##  ##  ##  ##  ##  ##  ##  ##  ##  ##  ##  ##  ##  ##  ##  ##  ##  ##  ##  ##  ##  ##  ##  ##  ##  ##  ##  ##  ##  ##  ##  ##  ##  ##  ##  ##  ##  ##  ##  ##  ##  ##  ##  ##  ##  ##  ##  ##  ##  ##  ##  ##  ##  ##  ##  ##  ##  ##  ##  ##  ##  ##  ##  ##  ##  ##  ##  ##  ##  ##  ##  ##  ##  ##  ##  ##  ##  ##  ##  ##  ##  ##  ##  ##  ##  ##  ##  ##  ##  ##  ##  ##  ##  ##  ##  ##  ##  ##  ##  ##  ##  ##  ##  ##  ##  ##  ##  ##  ##  ##  ##  ##  ##  ##  ##  ##  ##  ##  ##  ##  ##  ##  ##  ## [2901_3]52	: ##  ##  ##  ##  ##  ##  ##  ##  ##  ##  ##  ##  ##  ##  ##  ##  ##  ##  ##  ##  ##  ##  ##  ##  ##  10  7  10  11  9  9  9  11  9  11  10  11  10  12  7  8  9  11  10  8  11  11  9  8  9  11  --  9  10  10  10  12  7  8  11  9  11  11  9  10  11  9  9  9  ##  ##  ##  ##  ##  ##  ##  ##  ##  ##  ##  ##  ##  ##  ##  ##  ##  ##  ##  ##  ##  ##  ##  ##  ##  ##  ##  ##  ##  ##  ##  ##  ##  ##  ##  ##  ##  ##  ##  ##  ##  ##  ##  ##  ##  ##  ##  ##  ##  ##  ##  ##  ##  ##  ##  ##  ##  ##  ##  ##  ##  ##  ##  ##  ##  ##  ##  ##  ##  ##  ##  ##  ##  ##  ##  ##  ##  ##  ##  ##  ##  ##  ##  ##  ##  ##  ##  ##  ##  ##  ##  ##  ##  ##  ##  ##  ##  ##  ##  ##  ##  ##  ##  ##  ##  ##  ##  ##  ##  ##  ##  ##  ##  ##  ##  ##  ##  ##  ##  ##  ##  ##  ##  ##  ##  ##  ##  ##  ##  ##  ##  ##  ##  ##  ##  ##  ##  ##  ##  ##  ##  ##  ##  ##  ##  ##  ##  ##  ##  ##  ##  ##  ##  ##  ##  ##  ##  ##  ##  ##  ##  ##  ##  ##  ##  ##  ##  ##  ##  ##  ##  ##  ##  ##  ##  ##  ##  ##  ##  ##  ##  ##  ##  ##  ##  ##  ##  ##  ##  ##  ##  ##  ##  ##  ##  ##  ##  ##  ##  ##  ## [2906_3]53	: ##  ##  ##  ##  ##  ##  ##  ##  ##  ##  ##  ##  ##  ##  ##  ##  ##  ##  ##  ##  ##  ##  ##  ##  ##  5  4  5  10  6  8  8  6  4  6  7  6  7  3  4  5  2  8  3  3  6  6  2  5  4  6  9  --  2  5  5  3  2  3  6  6  6  6  6  10  2  6  4  8  ##  ##  ##  ##  ##  ##  ##  ##  ##  ##  ##  ##  ##  ##  ##  ##  ##  ##  ##  ##  ##  ##  ##  ##  ##  ##  ##  ##  ##  ##  ##  ##  ##  ##  ##  ##  ##  ##  ##  ##  ##  ##  ##  ##  ##  ##  ##  ##  ##  ##  ##  ##  ##  ##  ##  ##  ##  ##  ##  ##  ##  ##  ##  ##  ##  ##  ##  ##  ##  ##  ##  ##  ##  ##  ##  ##  ##  ##  ##  ##  ##  ##  ##  ##  ##  ##  ##  ##  ##  ##  ##  ##  ##  ##  ##  ##  ##  ##  ##  ##  ##  ##  ##  ##  ##  ##  ##  ##  ##  ##  ##  ##  ##  ##  ##  ##  ##  ##  ##  ##  ##  ##  ##  ##  ##  ##  ##  ##  ##  ##  ##  ##  ##  ##  ##  ##  ##  ##  ##  ##  ##  ##  ##  ##  ##  ##  ##  ##  ##  ##  ##  ##  ##  ##  ##  ##  ##  ##  ##  ##  ##  ##  ##  ##  ##  ##  ##  ##  ##  ##  ##  ##  ##  ##  ##  ##  ##  ##  ##  ##  ##  ##  ##  ##  ##  ##  ##  ##  ##  ##  ##  ##  ##  ##  ##  ##  ##  ##  ##  ##  ## [2907_3]54	: ##  ##  ##  ##  ##  ##  ##  ##  ##  ##  ##  ##  ##  ##  ##  ##  ##  ##  ##  ##  ##  ##  ##  ##  ##  7  6  7  12  8  10  10  8  6  8  9  8  9  3  6  7  4  10  5  5  8  8  4  7  6  8  10  2  --  7  7  5  4  5  8  8  8  8  8  12  4  8  6  10  ##  ##  ##  ##  ##  ##  ##  ##  ##  ##  ##  ##  ##  ##  ##  ##  ##  ##  ##  ##  ##  ##  ##  ##  ##  ##  ##  ##  ##  ##  ##  ##  ##  ##  ##  ##  ##  ##  ##  ##  ##  ##  ##  ##  ##  ##  ##  ##  ##  ##  ##  ##  ##  ##  ##  ##  ##  ##  ##  ##  ##  ##  ##  ##  ##  ##  ##  ##  ##  ##  ##  ##  ##  ##  ##  ##  ##  ##  ##  ##  ##  ##  ##  ##  ##  ##  ##  ##  ##  ##  ##  ##  ##  ##  ##  ##  ##  ##  ##  ##  ##  ##  ##  ##  ##  ##  ##  ##  ##  ##  ##  ##  ##  ##  ##  ##  ##  ##  ##  ##  ##  ##  ##  ##  ##  ##  ##  ##  ##  ##  ##  ##  ##  ##  ##  ##  ##  ##  ##  ##  ##  ##  ##  ##  ##  ##  ##  ##  ##  ##  ##  ##  ##  ##  ##  ##  ##  ##  ##  ##  ##  ##  ##  ##  ##  ##  ##  ##  ##  ##  ##  ##  ##  ##  ##  ##  ##  ##  ##  ##  ##  ##  ##  ##  ##  ##  ##  ##  ##  ##  ##  ##  ##  ##  ##  ##  ##  ##  ##  ##  ## [2908_3]55	: ##  ##  ##  ##  ##  ##  ##  ##  ##  ##  ##  ##  ##  ##  ##  ##  ##  ##  ##  ##  ##  ##  ##  ##  ##  6  5  6  11  7  9  7  7  5  7  8  7  8  8  5  6  5  9  6  6  7  7  7  8  5  7  10  5  7  --  6  8  5  4  7  7  7  7  7  11  7  7  3  9  ##  ##  ##  ##  ##  ##  ##  ##  ##  ##  ##  ##  ##  ##  ##  ##  ##  ##  ##  ##  ##  ##  ##  ##  ##  ##  ##  ##  ##  ##  ##  ##  ##  ##  ##  ##  ##  ##  ##  ##  ##  ##  ##  ##  ##  ##  ##  ##  ##  ##  ##  ##  ##  ##  ##  ##  ##  ##  ##  ##  ##  ##  ##  ##  ##  ##  ##  ##  ##  ##  ##  ##  ##  ##  ##  ##  ##  ##  ##  ##  ##  ##  ##  ##  ##  ##  ##  ##  ##  ##  ##  ##  ##  ##  ##  ##  ##  ##  ##  ##  ##  ##  ##  ##  ##  ##  ##  ##  ##  ##  ##  ##  ##  ##  ##  ##  ##  ##  ##  ##  ##  ##  ##  ##  ##  ##  ##  ##  ##  ##  ##  ##  ##  ##  ##  ##  ##  ##  ##  ##  ##  ##  ##  ##  ##  ##  ##  ##  ##  ##  ##  ##  ##  ##  ##  ##  ##  ##  ##  ##  ##  ##  ##  ##  ##  ##  ##  ##  ##  ##  ##  ##  ##  ##  ##  ##  ##  ##  ##  ##  ##  ##  ##  ##  ##  ##  ##  ##  ##  ##  ##  ##  ##  ##  ##  ##  ##  ##  ##  ##  ## [2910_3]56	: ##  ##  ##  ##  ##  ##  ##  ##  ##  ##  ##  ##  ##  ##  ##  ##  ##  ##  ##  ##  ##  ##  ##  ##  ##  6  7  2  11  7  9  9  3  5  3  8  3  8  8  5  6  5  5  6  6  3  3  7  8  1  3  10  5  7  6  --  8  5  4  3  7  3  3  3  11  7  3  5  9  ##  ##  ##  ##  ##  ##  ##  ##  ##  ##  ##  ##  ##  ##  ##  ##  ##  ##  ##  ##  ##  ##  ##  ##  ##  ##  ##  ##  ##  ##  ##  ##  ##  ##  ##  ##  ##  ##  ##  ##  ##  ##  ##  ##  ##  ##  ##  ##  ##  ##  ##  ##  ##  ##  ##  ##  ##  ##  ##  ##  ##  ##  ##  ##  ##  ##  ##  ##  ##  ##  ##  ##  ##  ##  ##  ##  ##  ##  ##  ##  ##  ##  ##  ##  ##  ##  ##  ##  ##  ##  ##  ##  ##  ##  ##  ##  ##  ##  ##  ##  ##  ##  ##  ##  ##  ##  ##  ##  ##  ##  ##  ##  ##  ##  ##  ##  ##  ##  ##  ##  ##  ##  ##  ##  ##  ##  ##  ##  ##  ##  ##  ##  ##  ##  ##  ##  ##  ##  ##  ##  ##  ##  ##  ##  ##  ##  ##  ##  ##  ##  ##  ##  ##  ##  ##  ##  ##  ##  ##  ##  ##  ##  ##  ##  ##  ##  ##  ##  ##  ##  ##  ##  ##  ##  ##  ##  ##  ##  ##  ##  ##  ##  ##  ##  ##  ##  ##  ##  ##  ##  ##  ##  ##  ##  ##  ##  ##  ##  ##  ##  ## [2912_3]57	: ##  ##  ##  ##  ##  ##  ##  ##  ##  ##  ##  ##  ##  ##  ##  ##  ##  ##  ##  ##  ##  ##  ##  ##  ##  8  7  6  13  9  11  11  7  7  9  10  7  10  6  7  8  5  9  4  6  5  7  5  8  7  7  12  3  5  8  8  --  5  6  9  9  7  7  7  11  3  7  7  11  ##  ##  ##  ##  ##  ##  ##  ##  ##  ##  ##  ##  ##  ##  ##  ##  ##  ##  ##  ##  ##  ##  ##  ##  ##  ##  ##  ##  ##  ##  ##  ##  ##  ##  ##  ##  ##  ##  ##  ##  ##  ##  ##  ##  ##  ##  ##  ##  ##  ##  ##  ##  ##  ##  ##  ##  ##  ##  ##  ##  ##  ##  ##  ##  ##  ##  ##  ##  ##  ##  ##  ##  ##  ##  ##  ##  ##  ##  ##  ##  ##  ##  ##  ##  ##  ##  ##  ##  ##  ##  ##  ##  ##  ##  ##  ##  ##  ##  ##  ##  ##  ##  ##  ##  ##  ##  ##  ##  ##  ##  ##  ##  ##  ##  ##  ##  ##  ##  ##  ##  ##  ##  ##  ##  ##  ##  ##  ##  ##  ##  ##  ##  ##  ##  ##  ##  ##  ##  ##  ##  ##  ##  ##  ##  ##  ##  ##  ##  ##  ##  ##  ##  ##  ##  ##  ##  ##  ##  ##  ##  ##  ##  ##  ##  ##  ##  ##  ##  ##  ##  ##  ##  ##  ##  ##  ##  ##  ##  ##  ##  ##  ##  ##  ##  ##  ##  ##  ##  ##  ##  ##  ##  ##  ##  ##  ##  ##  ##  ##  ##  ## [2913_3]58	: ##  ##  ##  ##  ##  ##  ##  ##  ##  ##  ##  ##  ##  ##  ##  ##  ##  ##  ##  ##  ##  ##  ##  ##  ##  5  2  5  10  6  8  8  6  4  6  7  6  7  5  2  3  4  6  5  3  6  6  4  3  4  6  7  2  4  5  5  5  --  3  6  6  6  6  4  8  4  6  4  6  ##  ##  ##  ##  ##  ##  ##  ##  ##  ##  ##  ##  ##  ##  ##  ##  ##  ##  ##  ##  ##  ##  ##  ##  ##  ##  ##  ##  ##  ##  ##  ##  ##  ##  ##  ##  ##  ##  ##  ##  ##  ##  ##  ##  ##  ##  ##  ##  ##  ##  ##  ##  ##  ##  ##  ##  ##  ##  ##  ##  ##  ##  ##  ##  ##  ##  ##  ##  ##  ##  ##  ##  ##  ##  ##  ##  ##  ##  ##  ##  ##  ##  ##  ##  ##  ##  ##  ##  ##  ##  ##  ##  ##  ##  ##  ##  ##  ##  ##  ##  ##  ##  ##  ##  ##  ##  ##  ##  ##  ##  ##  ##  ##  ##  ##  ##  ##  ##  ##  ##  ##  ##  ##  ##  ##  ##  ##  ##  ##  ##  ##  ##  ##  ##  ##  ##  ##  ##  ##  ##  ##  ##  ##  ##  ##  ##  ##  ##  ##  ##  ##  ##  ##  ##  ##  ##  ##  ##  ##  ##  ##  ##  ##  ##  ##  ##  ##  ##  ##  ##  ##  ##  ##  ##  ##  ##  ##  ##  ##  ##  ##  ##  ##  ##  ##  ##  ##  ##  ##  ##  ##  ##  ##  ##  ##  ##  ##  ##  ##  ##  ## [2915_3]59	: ##  ##  ##  ##  ##  ##  ##  ##  ##  ##  ##  ##  ##  ##  ##  ##  ##  ##  ##  ##  ##  ##  ##  ##  ##  4  5  4  9  5  7  7  5  3  5  6  5  6  6  3  4  3  7  4  4  5  5  5  6  3  5  8  3  5  4  4  6  3  --  5  5  5  5  5  9  5  5  3  7  ##  ##  ##  ##  ##  ##  ##  ##  ##  ##  ##  ##  ##  ##  ##  ##  ##  ##  ##  ##  ##  ##  ##  ##  ##  ##  ##  ##  ##  ##  ##  ##  ##  ##  ##  ##  ##  ##  ##  ##  ##  ##  ##  ##  ##  ##  ##  ##  ##  ##  ##  ##  ##  ##  ##  ##  ##  ##  ##  ##  ##  ##  ##  ##  ##  ##  ##  ##  ##  ##  ##  ##  ##  ##  ##  ##  ##  ##  ##  ##  ##  ##  ##  ##  ##  ##  ##  ##  ##  ##  ##  ##  ##  ##  ##  ##  ##  ##  ##  ##  ##  ##  ##  ##  ##  ##  ##  ##  ##  ##  ##  ##  ##  ##  ##  ##  ##  ##  ##  ##  ##  ##  ##  ##  ##  ##  ##  ##  ##  ##  ##  ##  ##  ##  ##  ##  ##  ##  ##  ##  ##  ##  ##  ##  ##  ##  ##  ##  ##  ##  ##  ##  ##  ##  ##  ##  ##  ##  ##  ##  ##  ##  ##  ##  ##  ##  ##  ##  ##  ##  ##  ##  ##  ##  ##  ##  ##  ##  ##  ##  ##  ##  ##  ##  ##  ##  ##  ##  ##  ##  ##  ##  ##  ##  ##  ##  ##  ##  ##  ##  ## [2916_3]60	: ##  ##  ##  ##  ##  ##  ##  ##  ##  ##  ##  ##  ##  ##  ##  ##  ##  ##  ##  ##  ##  ##  ##  ##  ##  7  8  3  11  8  10  10  4  6  4  9  4  9  9  6  7  6  6  7  7  4  4  8  9  2  3  11  6  8  7  3  9  6  5  --  8  4  2  4  12  8  4  6  10  ##  ##  ##  ##  ##  ##  ##  ##  ##  ##  ##  ##  ##  ##  ##  ##  ##  ##  ##  ##  ##  ##  ##  ##  ##  ##  ##  ##  ##  ##  ##  ##  ##  ##  ##  ##  ##  ##  ##  ##  ##  ##  ##  ##  ##  ##  ##  ##  ##  ##  ##  ##  ##  ##  ##  ##  ##  ##  ##  ##  ##  ##  ##  ##  ##  ##  ##  ##  ##  ##  ##  ##  ##  ##  ##  ##  ##  ##  ##  ##  ##  ##  ##  ##  ##  ##  ##  ##  ##  ##  ##  ##  ##  ##  ##  ##  ##  ##  ##  ##  ##  ##  ##  ##  ##  ##  ##  ##  ##  ##  ##  ##  ##  ##  ##  ##  ##  ##  ##  ##  ##  ##  ##  ##  ##  ##  ##  ##  ##  ##  ##  ##  ##  ##  ##  ##  ##  ##  ##  ##  ##  ##  ##  ##  ##  ##  ##  ##  ##  ##  ##  ##  ##  ##  ##  ##  ##  ##  ##  ##  ##  ##  ##  ##  ##  ##  ##  ##  ##  ##  ##  ##  ##  ##  ##  ##  ##  ##  ##  ##  ##  ##  ##  ##  ##  ##  ##  ##  ##  ##  ##  ##  ##  ##  ##  ##  ##  ##  ##  ##  ## [2922_3]61	: ##  ##  ##  ##  ##  ##  ##  ##  ##  ##  ##  ##  ##  ##  ##  ##  ##  ##  ##  ##  ##  ##  ##  ##  ##  7  6  7  8  8  10  10  8  6  8  3  8  1  9  6  7  4  10  7  5  8  8  8  7  6  8  9  6  8  7  7  9  6  5  8  --  8  8  8  6  8  8  6  4  ##  ##  ##  ##  ##  ##  ##  ##  ##  ##  ##  ##  ##  ##  ##  ##  ##  ##  ##  ##  ##  ##  ##  ##  ##  ##  ##  ##  ##  ##  ##  ##  ##  ##  ##  ##  ##  ##  ##  ##  ##  ##  ##  ##  ##  ##  ##  ##  ##  ##  ##  ##  ##  ##  ##  ##  ##  ##  ##  ##  ##  ##  ##  ##  ##  ##  ##  ##  ##  ##  ##  ##  ##  ##  ##  ##  ##  ##  ##  ##  ##  ##  ##  ##  ##  ##  ##  ##  ##  ##  ##  ##  ##  ##  ##  ##  ##  ##  ##  ##  ##  ##  ##  ##  ##  ##  ##  ##  ##  ##  ##  ##  ##  ##  ##  ##  ##  ##  ##  ##  ##  ##  ##  ##  ##  ##  ##  ##  ##  ##  ##  ##  ##  ##  ##  ##  ##  ##  ##  ##  ##  ##  ##  ##  ##  ##  ##  ##  ##  ##  ##  ##  ##  ##  ##  ##  ##  ##  ##  ##  ##  ##  ##  ##  ##  ##  ##  ##  ##  ##  ##  ##  ##  ##  ##  ##  ##  ##  ##  ##  ##  ##  ##  ##  ##  ##  ##  ##  ##  ##  ##  ##  ##  ##  ##  ##  ##  ##  ##  ##  ## [2923_3]62	: ##  ##  ##  ##  ##  ##  ##  ##  ##  ##  ##  ##  ##  ##  ##  ##  ##  ##  ##  ##  ##  ##  ##  ##  ##  7  8  1  12  8  10  10  2  6  4  9  2  9  9  6  7  6  4  5  7  2  2  8  9  2  2  11  6  8  7  3  7  6  5  4  8  --  2  2  12  8  2  6  10  ##  ##  ##  ##  ##  ##  ##  ##  ##  ##  ##  ##  ##  ##  ##  ##  ##  ##  ##  ##  ##  ##  ##  ##  ##  ##  ##  ##  ##  ##  ##  ##  ##  ##  ##  ##  ##  ##  ##  ##  ##  ##  ##  ##  ##  ##  ##  ##  ##  ##  ##  ##  ##  ##  ##  ##  ##  ##  ##  ##  ##  ##  ##  ##  ##  ##  ##  ##  ##  ##  ##  ##  ##  ##  ##  ##  ##  ##  ##  ##  ##  ##  ##  ##  ##  ##  ##  ##  ##  ##  ##  ##  ##  ##  ##  ##  ##  ##  ##  ##  ##  ##  ##  ##  ##  ##  ##  ##  ##  ##  ##  ##  ##  ##  ##  ##  ##  ##  ##  ##  ##  ##  ##  ##  ##  ##  ##  ##  ##  ##  ##  ##  ##  ##  ##  ##  ##  ##  ##  ##  ##  ##  ##  ##  ##  ##  ##  ##  ##  ##  ##  ##  ##  ##  ##  ##  ##  ##  ##  ##  ##  ##  ##  ##  ##  ##  ##  ##  ##  ##  ##  ##  ##  ##  ##  ##  ##  ##  ##  ##  ##  ##  ##  ##  ##  ##  ##  ##  ##  ##  ##  ##  ##  ##  ##  ##  ##  ##  ##  ##  ## [2929_3]63	: ##  ##  ##  ##  ##  ##  ##  ##  ##  ##  ##  ##  ##  ##  ##  ##  ##  ##  ##  ##  ##  ##  ##  ##  ##  7  8  1  12  8  10  10  2  6  4  9  2  9  9  6  7  6  4  5  7  2  2  8  9  2  2  11  6  8  7  3  7  6  5  2  8  2  --  2  12  8  2  6  10  ##  ##  ##  ##  ##  ##  ##  ##  ##  ##  ##  ##  ##  ##  ##  ##  ##  ##  ##  ##  ##  ##  ##  ##  ##  ##  ##  ##  ##  ##  ##  ##  ##  ##  ##  ##  ##  ##  ##  ##  ##  ##  ##  ##  ##  ##  ##  ##  ##  ##  ##  ##  ##  ##  ##  ##  ##  ##  ##  ##  ##  ##  ##  ##  ##  ##  ##  ##  ##  ##  ##  ##  ##  ##  ##  ##  ##  ##  ##  ##  ##  ##  ##  ##  ##  ##  ##  ##  ##  ##  ##  ##  ##  ##  ##  ##  ##  ##  ##  ##  ##  ##  ##  ##  ##  ##  ##  ##  ##  ##  ##  ##  ##  ##  ##  ##  ##  ##  ##  ##  ##  ##  ##  ##  ##  ##  ##  ##  ##  ##  ##  ##  ##  ##  ##  ##  ##  ##  ##  ##  ##  ##  ##  ##  ##  ##  ##  ##  ##  ##  ##  ##  ##  ##  ##  ##  ##  ##  ##  ##  ##  ##  ##  ##  ##  ##  ##  ##  ##  ##  ##  ##  ##  ##  ##  ##  ##  ##  ##  ##  ##  ##  ##  ##  ##  ##  ##  ##  ##  ##  ##  ##  ##  ##  ##  ##  ##  ##  ##  ##  ## [2930_3]64	: ##  ##  ##  ##  ##  ##  ##  ##  ##  ##  ##  ##  ##  ##  ##  ##  ##  ##  ##  ##  ##  ##  ##  ##  ##  7  6  1  12  8  10  10  2  6  4  9  2  9  9  4  5  6  2  5  7  2  2  8  7  2  2  9  6  8  7  3  7  4  5  4  8  2  2  --  10  8  2  6  8  ##  ##  ##  ##  ##  ##  ##  ##  ##  ##  ##  ##  ##  ##  ##  ##  ##  ##  ##  ##  ##  ##  ##  ##  ##  ##  ##  ##  ##  ##  ##  ##  ##  ##  ##  ##  ##  ##  ##  ##  ##  ##  ##  ##  ##  ##  ##  ##  ##  ##  ##  ##  ##  ##  ##  ##  ##  ##  ##  ##  ##  ##  ##  ##  ##  ##  ##  ##  ##  ##  ##  ##  ##  ##  ##  ##  ##  ##  ##  ##  ##  ##  ##  ##  ##  ##  ##  ##  ##  ##  ##  ##  ##  ##  ##  ##  ##  ##  ##  ##  ##  ##  ##  ##  ##  ##  ##  ##  ##  ##  ##  ##  ##  ##  ##  ##  ##  ##  ##  ##  ##  ##  ##  ##  ##  ##  ##  ##  ##  ##  ##  ##  ##  ##  ##  ##  ##  ##  ##  ##  ##  ##  ##  ##  ##  ##  ##  ##  ##  ##  ##  ##  ##  ##  ##  ##  ##  ##  ##  ##  ##  ##  ##  ##  ##  ##  ##  ##  ##  ##  ##  ##  ##  ##  ##  ##  ##  ##  ##  ##  ##  ##  ##  ##  ##  ##  ##  ##  ##  ##  ##  ##  ##  ##  ##  ##  ##  ##  ##  ##  ## [2931_3]65	: ##  ##  ##  ##  ##  ##  ##  ##  ##  ##  ##  ##  ##  ##  ##  ##  ##  ##  ##  ##  ##  ##  ##  ##  ##  11  8  11  12  12  14  14  12  10  12  3  12  7  13  8  9  8  12  11  9  10  12  12  7  10  12  10  10  12  11  11  11  8  9  12  6  12  12  10  --  12  12  10  6  ##  ##  ##  ##  ##  ##  ##  ##  ##  ##  ##  ##  ##  ##  ##  ##  ##  ##  ##  ##  ##  ##  ##  ##  ##  ##  ##  ##  ##  ##  ##  ##  ##  ##  ##  ##  ##  ##  ##  ##  ##  ##  ##  ##  ##  ##  ##  ##  ##  ##  ##  ##  ##  ##  ##  ##  ##  ##  ##  ##  ##  ##  ##  ##  ##  ##  ##  ##  ##  ##  ##  ##  ##  ##  ##  ##  ##  ##  ##  ##  ##  ##  ##  ##  ##  ##  ##  ##  ##  ##  ##  ##  ##  ##  ##  ##  ##  ##  ##  ##  ##  ##  ##  ##  ##  ##  ##  ##  ##  ##  ##  ##  ##  ##  ##  ##  ##  ##  ##  ##  ##  ##  ##  ##  ##  ##  ##  ##  ##  ##  ##  ##  ##  ##  ##  ##  ##  ##  ##  ##  ##  ##  ##  ##  ##  ##  ##  ##  ##  ##  ##  ##  ##  ##  ##  ##  ##  ##  ##  ##  ##  ##  ##  ##  ##  ##  ##  ##  ##  ##  ##  ##  ##  ##  ##  ##  ##  ##  ##  ##  ##  ##  ##  ##  ##  ##  ##  ##  ##  ##  ##  ##  ##  ##  ##  ##  ##  ##  ##  ##  ## [2932_3]66	: ##  ##  ##  ##  ##  ##  ##  ##  ##  ##  ##  ##  ##  ##  ##  ##  ##  ##  ##  ##  ##  ##  ##  ##  ##  7  6  7  12  8  10  10  8  6  8  9  8  9  5  6  7  4  10  5  5  8  8  4  7  6  8  11  2  4  7  7  3  4  5  8  8  8  8  8  12  --  8  6  10  ##  ##  ##  ##  ##  ##  ##  ##  ##  ##  ##  ##  ##  ##  ##  ##  ##  ##  ##  ##  ##  ##  ##  ##  ##  ##  ##  ##  ##  ##  ##  ##  ##  ##  ##  ##  ##  ##  ##  ##  ##  ##  ##  ##  ##  ##  ##  ##  ##  ##  ##  ##  ##  ##  ##  ##  ##  ##  ##  ##  ##  ##  ##  ##  ##  ##  ##  ##  ##  ##  ##  ##  ##  ##  ##  ##  ##  ##  ##  ##  ##  ##  ##  ##  ##  ##  ##  ##  ##  ##  ##  ##  ##  ##  ##  ##  ##  ##  ##  ##  ##  ##  ##  ##  ##  ##  ##  ##  ##  ##  ##  ##  ##  ##  ##  ##  ##  ##  ##  ##  ##  ##  ##  ##  ##  ##  ##  ##  ##  ##  ##  ##  ##  ##  ##  ##  ##  ##  ##  ##  ##  ##  ##  ##  ##  ##  ##  ##  ##  ##  ##  ##  ##  ##  ##  ##  ##  ##  ##  ##  ##  ##  ##  ##  ##  ##  ##  ##  ##  ##  ##  ##  ##  ##  ##  ##  ##  ##  ##  ##  ##  ##  ##  ##  ##  ##  ##  ##  ##  ##  ##  ##  ##  ##  ##  ##  ##  ##  ##  ##  ## [2934_3]67	: ##  ##  ##  ##  ##  ##  ##  ##  ##  ##  ##  ##  ##  ##  ##  ##  ##  ##  ##  ##  ##  ##  ##  ##  ##  7  8  1  12  8  10  10  2  6  4  9  2  9  9  6  7  6  4  5  7  2  2  8  9  2  2  9  6  8  7  3  7  6  5  4  8  2  2  2  12  8  --  6  10  ##  ##  ##  ##  ##  ##  ##  ##  ##  ##  ##  ##  ##  ##  ##  ##  ##  ##  ##  ##  ##  ##  ##  ##  ##  ##  ##  ##  ##  ##  ##  ##  ##  ##  ##  ##  ##  ##  ##  ##  ##  ##  ##  ##  ##  ##  ##  ##  ##  ##  ##  ##  ##  ##  ##  ##  ##  ##  ##  ##  ##  ##  ##  ##  ##  ##  ##  ##  ##  ##  ##  ##  ##  ##  ##  ##  ##  ##  ##  ##  ##  ##  ##  ##  ##  ##  ##  ##  ##  ##  ##  ##  ##  ##  ##  ##  ##  ##  ##  ##  ##  ##  ##  ##  ##  ##  ##  ##  ##  ##  ##  ##  ##  ##  ##  ##  ##  ##  ##  ##  ##  ##  ##  ##  ##  ##  ##  ##  ##  ##  ##  ##  ##  ##  ##  ##  ##  ##  ##  ##  ##  ##  ##  ##  ##  ##  ##  ##  ##  ##  ##  ##  ##  ##  ##  ##  ##  ##  ##  ##  ##  ##  ##  ##  ##  ##  ##  ##  ##  ##  ##  ##  ##  ##  ##  ##  ##  ##  ##  ##  ##  ##  ##  ##  ##  ##  ##  ##  ##  ##  ##  ##  ##  ##  ##  ##  ##  ##  ##  ##  ## [2935_3]68	: ##  ##  ##  ##  ##  ##  ##  ##  ##  ##  ##  ##  ##  ##  ##  ##  ##  ##  ##  ##  ##  ##  ##  ##  ##  5  6  5  10  6  8  8  6  4  6  7  6  7  7  4  3  4  8  5  5  6  6  6  7  4  6  9  4  6  3  5  7  4  3  6  6  6  6  6  10  6  6  --  8  ##  ##  ##  ##  ##  ##  ##  ##  ##  ##  ##  ##  ##  ##  ##  ##  ##  ##  ##  ##  ##  ##  ##  ##  ##  ##  ##  ##  ##  ##  ##  ##  ##  ##  ##  ##  ##  ##  ##  ##  ##  ##  ##  ##  ##  ##  ##  ##  ##  ##  ##  ##  ##  ##  ##  ##  ##  ##  ##  ##  ##  ##  ##  ##  ##  ##  ##  ##  ##  ##  ##  ##  ##  ##  ##  ##  ##  ##  ##  ##  ##  ##  ##  ##  ##  ##  ##  ##  ##  ##  ##  ##  ##  ##  ##  ##  ##  ##  ##  ##  ##  ##  ##  ##  ##  ##  ##  ##  ##  ##  ##  ##  ##  ##  ##  ##  ##  ##  ##  ##  ##  ##  ##  ##  ##  ##  ##  ##  ##  ##  ##  ##  ##  ##  ##  ##  ##  ##  ##  ##  ##  ##  ##  ##  ##  ##  ##  ##  ##  ##  ##  ##  ##  ##  ##  ##  ##  ##  ##  ##  ##  ##  ##  ##  ##  ##  ##  ##  ##  ##  ##  ##  ##  ##  ##  ##  ##  ##  ##  ##  ##  ##  ##  ##  ##  ##  ##  ##  ##  ##  ##  ##  ##  ##  ##  ##  ##  ##  ##  ##  ## [2480_3]69	: ##  ##  ##  ##  ##  ##  ##  ##  ##  ##  ##  ##  ##  ##  ##  ##  ##  ##  ##  ##  ##  ##  ##  ##  ##  9  6  9  10  10  12  12  10  8  10  5  10  5  11  6  7  6  10  9  7  10  10  10  7  8  10  9  8  10  9  9  11  6  7  10  4  10  10  8  6  10  10  8  --  ##  ##  ##  ##  ##  ##  ##  ##  ##  ##  ##  ##  ##  ##  ##  ##  ##  ##  ##  ##  ##  ##  ##  ##  ##  ##  ##  ##  ##  ##  ##  ##  ##  ##  ##  ##  ##  ##  ##  ##  ##  ##  ##  ##  ##  ##  ##  ##  ##  ##  ##  ##  ##  ##  ##  ##  ##  ##  ##  ##  ##  ##  ##  ##  ##  ##  ##  ##  ##  ##  ##  ##  ##  ##  ##  ##  ##  ##  ##  ##  ##  ##  ##  ##  ##  ##  ##  ##  ##  ##  ##  ##  ##  ##  ##  ##  ##  ##  ##  ##  ##  ##  ##  ##  ##  ##  ##  ##  ##  ##  ##  ##  ##  ##  ##  ##  ##  ##  ##  ##  ##  ##  ##  ##  ##  ##  ##  ##  ##  ##  ##  ##  ##  ##  ##  ##  ##  ##  ##  ##  ##  ##  ##  ##  ##  ##  ##  ##  ##  ##  ##  ##  ##  ##  ##  ##  ##  ##  ##  ##  ##  ##  ##  ##  ##  ##  ##  ##  ##  ##  ##  ##  ##  ##  ##  ##  ##  ##  ##  ##  ##  ##  ##  ##  ##  ##  ##  ##  ##  ##  ##  ##  ##  ##  ##  ##  ##  ##  ##  ##  ## [825_1]70	: ##  ##  ##  ##  ##  ##  ##  ##  ##  ##  ##  ##  ##  ##  ##  ##  ##  ##  ##  ##  ##  ##  ##  ##  ##  ##  ##  ##  ##  ##  ##  ##  ##  ##  ##  ##  ##  ##  ##  ##  ##  ##  ##  ##  ##  ##  ##  ##  ##  ##  ##  ##  ##  ##  ##  ##  ##  ##  ##  ##  ##  ##  ##  ##  ##  ##  ##  ##  ##  --  1  1  1  9  1  1  1  1  2  1  2  4  ##  ##  ##  ##  ##  ##  ##  ##  ##  ##  ##  ##  ##  ##  ##  ##  ##  ##  ##  ##  ##  ##  ##  ##  ##  ##  ##  ##  ##  ##  ##  ##  ##  ##  ##  ##  ##  ##  ##  ##  ##  ##  ##  ##  ##  ##  ##  ##  ##  ##  ##  ##  ##  ##  ##  ##  ##  ##  ##  ##  ##  ##  ##  ##  ##  ##  ##  ##  ##  ##  ##  ##  ##  ##  ##  ##  ##  ##  ##  ##  ##  ##  ##  ##  ##  ##  ##  ##  ##  ##  ##  ##  ##  ##  ##  ##  ##  ##  ##  ##  ##  ##  ##  ##  ##  ##  ##  ##  ##  ##  ##  ##  ##  ##  ##  ##  ##  ##  ##  ##  ##  ##  ##  ##  ##  ##  ##  ##  ##  ##  ##  ##  ##  ##  ##  ##  ##  ##  ##  ##  ##  ##  ##  ##  ##  ##  ##  ##  ##  ##  ##  ##  ##  ##  ##  ##  ##  ##  ##  ##  ##  ##  ##  ##  ##  ##  ##  ##  ##  ##  ##  ##  ##  ##  ##  ##  ##  ##  ##  ##  ##  ##  ##  ##  ##  ##  ##  ## [826_1]71	: ##  ##  ##  ##  ##  ##  ##  ##  ##  ##  ##  ##  ##  ##  ##  ##  ##  ##  ##  ##  ##  ##  ##  ##  ##  ##  ##  ##  ##  ##  ##  ##  ##  ##  ##  ##  ##  ##  ##  ##  ##  ##  ##  ##  ##  ##  ##  ##  ##  ##  ##  ##  ##  ##  ##  ##  ##  ##  ##  ##  ##  ##  ##  ##  ##  ##  ##  ##  ##  1  --  2  2  10  2  2  2  2  3  2  2  5  ##  ##  ##  ##  ##  ##  ##  ##  ##  ##  ##  ##  ##  ##  ##  ##  ##  ##  ##  ##  ##  ##  ##  ##  ##  ##  ##  ##  ##  ##  ##  ##  ##  ##  ##  ##  ##  ##  ##  ##  ##  ##  ##  ##  ##  ##  ##  ##  ##  ##  ##  ##  ##  ##  ##  ##  ##  ##  ##  ##  ##  ##  ##  ##  ##  ##  ##  ##  ##  ##  ##  ##  ##  ##  ##  ##  ##  ##  ##  ##  ##  ##  ##  ##  ##  ##  ##  ##  ##  ##  ##  ##  ##  ##  ##  ##  ##  ##  ##  ##  ##  ##  ##  ##  ##  ##  ##  ##  ##  ##  ##  ##  ##  ##  ##  ##  ##  ##  ##  ##  ##  ##  ##  ##  ##  ##  ##  ##  ##  ##  ##  ##  ##  ##  ##  ##  ##  ##  ##  ##  ##  ##  ##  ##  ##  ##  ##  ##  ##  ##  ##  ##  ##  ##  ##  ##  ##  ##  ##  ##  ##  ##  ##  ##  ##  ##  ##  ##  ##  ##  ##  ##  ##  ##  ##  ##  ##  ##  ##  ##  ##  ##  ##  ##  ##  ##  ##  ## [833_1]72	: ##  ##  ##  ##  ##  ##  ##  ##  ##  ##  ##  ##  ##  ##  ##  ##  ##  ##  ##  ##  ##  ##  ##  ##  ##  ##  ##  ##  ##  ##  ##  ##  ##  ##  ##  ##  ##  ##  ##  ##  ##  ##  ##  ##  ##  ##  ##  ##  ##  ##  ##  ##  ##  ##  ##  ##  ##  ##  ##  ##  ##  ##  ##  ##  ##  ##  ##  ##  ##  1  2  --  2  8  2  2  2  2  3  2  3  5  ##  ##  ##  ##  ##  ##  ##  ##  ##  ##  ##  ##  ##  ##  ##  ##  ##  ##  ##  ##  ##  ##  ##  ##  ##  ##  ##  ##  ##  ##  ##  ##  ##  ##  ##  ##  ##  ##  ##  ##  ##  ##  ##  ##  ##  ##  ##  ##  ##  ##  ##  ##  ##  ##  ##  ##  ##  ##  ##  ##  ##  ##  ##  ##  ##  ##  ##  ##  ##  ##  ##  ##  ##  ##  ##  ##  ##  ##  ##  ##  ##  ##  ##  ##  ##  ##  ##  ##  ##  ##  ##  ##  ##  ##  ##  ##  ##  ##  ##  ##  ##  ##  ##  ##  ##  ##  ##  ##  ##  ##  ##  ##  ##  ##  ##  ##  ##  ##  ##  ##  ##  ##  ##  ##  ##  ##  ##  ##  ##  ##  ##  ##  ##  ##  ##  ##  ##  ##  ##  ##  ##  ##  ##  ##  ##  ##  ##  ##  ##  ##  ##  ##  ##  ##  ##  ##  ##  ##  ##  ##  ##  ##  ##  ##  ##  ##  ##  ##  ##  ##  ##  ##  ##  ##  ##  ##  ##  ##  ##  ##  ##  ##  ##  ##  ##  ##  ##  ## [858_1]73	: ##  ##  ##  ##  ##  ##  ##  ##  ##  ##  ##  ##  ##  ##  ##  ##  ##  ##  ##  ##  ##  ##  ##  ##  ##  ##  ##  ##  ##  ##  ##  ##  ##  ##  ##  ##  ##  ##  ##  ##  ##  ##  ##  ##  ##  ##  ##  ##  ##  ##  ##  ##  ##  ##  ##  ##  ##  ##  ##  ##  ##  ##  ##  ##  ##  ##  ##  ##  ##  1  2  2  --  10  2  2  2  2  3  2  3  5  ##  ##  ##  ##  ##  ##  ##  ##  ##  ##  ##  ##  ##  ##  ##  ##  ##  ##  ##  ##  ##  ##  ##  ##  ##  ##  ##  ##  ##  ##  ##  ##  ##  ##  ##  ##  ##  ##  ##  ##  ##  ##  ##  ##  ##  ##  ##  ##  ##  ##  ##  ##  ##  ##  ##  ##  ##  ##  ##  ##  ##  ##  ##  ##  ##  ##  ##  ##  ##  ##  ##  ##  ##  ##  ##  ##  ##  ##  ##  ##  ##  ##  ##  ##  ##  ##  ##  ##  ##  ##  ##  ##  ##  ##  ##  ##  ##  ##  ##  ##  ##  ##  ##  ##  ##  ##  ##  ##  ##  ##  ##  ##  ##  ##  ##  ##  ##  ##  ##  ##  ##  ##  ##  ##  ##  ##  ##  ##  ##  ##  ##  ##  ##  ##  ##  ##  ##  ##  ##  ##  ##  ##  ##  ##  ##  ##  ##  ##  ##  ##  ##  ##  ##  ##  ##  ##  ##  ##  ##  ##  ##  ##  ##  ##  ##  ##  ##  ##  ##  ##  ##  ##  ##  ##  ##  ##  ##  ##  ##  ##  ##  ##  ##  ##  ##  ##  ##  ## [1325_1]74	: ##  ##  ##  ##  ##  ##  ##  ##  ##  ##  ##  ##  ##  ##  ##  ##  ##  ##  ##  ##  ##  ##  ##  ##  ##  ##  ##  ##  ##  ##  ##  ##  ##  ##  ##  ##  ##  ##  ##  ##  ##  ##  ##  ##  ##  ##  ##  ##  ##  ##  ##  ##  ##  ##  ##  ##  ##  ##  ##  ##  ##  ##  ##  ##  ##  ##  ##  ##  ##  9  10  8  10  --  10  10  10  10  11  10  9  10  ##  ##  ##  ##  ##  ##  ##  ##  ##  ##  ##  ##  ##  ##  ##  ##  ##  ##  ##  ##  ##  ##  ##  ##  ##  ##  ##  ##  ##  ##  ##  ##  ##  ##  ##  ##  ##  ##  ##  ##  ##  ##  ##  ##  ##  ##  ##  ##  ##  ##  ##  ##  ##  ##  ##  ##  ##  ##  ##  ##  ##  ##  ##  ##  ##  ##  ##  ##  ##  ##  ##  ##  ##  ##  ##  ##  ##  ##  ##  ##  ##  ##  ##  ##  ##  ##  ##  ##  ##  ##  ##  ##  ##  ##  ##  ##  ##  ##  ##  ##  ##  ##  ##  ##  ##  ##  ##  ##  ##  ##  ##  ##  ##  ##  ##  ##  ##  ##  ##  ##  ##  ##  ##  ##  ##  ##  ##  ##  ##  ##  ##  ##  ##  ##  ##  ##  ##  ##  ##  ##  ##  ##  ##  ##  ##  ##  ##  ##  ##  ##  ##  ##  ##  ##  ##  ##  ##  ##  ##  ##  ##  ##  ##  ##  ##  ##  ##  ##  ##  ##  ##  ##  ##  ##  ##  ##  ##  ##  ##  ##  ##  ##  ##  ##  ##  ##  ##  ## [1327_1]75	: ##  ##  ##  ##  ##  ##  ##  ##  ##  ##  ##  ##  ##  ##  ##  ##  ##  ##  ##  ##  ##  ##  ##  ##  ##  ##  ##  ##  ##  ##  ##  ##  ##  ##  ##  ##  ##  ##  ##  ##  ##  ##  ##  ##  ##  ##  ##  ##  ##  ##  ##  ##  ##  ##  ##  ##  ##  ##  ##  ##  ##  ##  ##  ##  ##  ##  ##  ##  ##  1  2  2  2  10  --  2  2  2  3  2  2  5  ##  ##  ##  ##  ##  ##  ##  ##  ##  ##  ##  ##  ##  ##  ##  ##  ##  ##  ##  ##  ##  ##  ##  ##  ##  ##  ##  ##  ##  ##  ##  ##  ##  ##  ##  ##  ##  ##  ##  ##  ##  ##  ##  ##  ##  ##  ##  ##  ##  ##  ##  ##  ##  ##  ##  ##  ##  ##  ##  ##  ##  ##  ##  ##  ##  ##  ##  ##  ##  ##  ##  ##  ##  ##  ##  ##  ##  ##  ##  ##  ##  ##  ##  ##  ##  ##  ##  ##  ##  ##  ##  ##  ##  ##  ##  ##  ##  ##  ##  ##  ##  ##  ##  ##  ##  ##  ##  ##  ##  ##  ##  ##  ##  ##  ##  ##  ##  ##  ##  ##  ##  ##  ##  ##  ##  ##  ##  ##  ##  ##  ##  ##  ##  ##  ##  ##  ##  ##  ##  ##  ##  ##  ##  ##  ##  ##  ##  ##  ##  ##  ##  ##  ##  ##  ##  ##  ##  ##  ##  ##  ##  ##  ##  ##  ##  ##  ##  ##  ##  ##  ##  ##  ##  ##  ##  ##  ##  ##  ##  ##  ##  ##  ##  ##  ##  ##  ##  ## [1342_1]76	: ##  ##  ##  ##  ##  ##  ##  ##  ##  ##  ##  ##  ##  ##  ##  ##  ##  ##  ##  ##  ##  ##  ##  ##  ##  ##  ##  ##  ##  ##  ##  ##  ##  ##  ##  ##  ##  ##  ##  ##  ##  ##  ##  ##  ##  ##  ##  ##  ##  ##  ##  ##  ##  ##  ##  ##  ##  ##  ##  ##  ##  ##  ##  ##  ##  ##  ##  ##  ##  1  2  2  2  10  2  --  2  2  3  2  3  3  ##  ##  ##  ##  ##  ##  ##  ##  ##  ##  ##  ##  ##  ##  ##  ##  ##  ##  ##  ##  ##  ##  ##  ##  ##  ##  ##  ##  ##  ##  ##  ##  ##  ##  ##  ##  ##  ##  ##  ##  ##  ##  ##  ##  ##  ##  ##  ##  ##  ##  ##  ##  ##  ##  ##  ##  ##  ##  ##  ##  ##  ##  ##  ##  ##  ##  ##  ##  ##  ##  ##  ##  ##  ##  ##  ##  ##  ##  ##  ##  ##  ##  ##  ##  ##  ##  ##  ##  ##  ##  ##  ##  ##  ##  ##  ##  ##  ##  ##  ##  ##  ##  ##  ##  ##  ##  ##  ##  ##  ##  ##  ##  ##  ##  ##  ##  ##  ##  ##  ##  ##  ##  ##  ##  ##  ##  ##  ##  ##  ##  ##  ##  ##  ##  ##  ##  ##  ##  ##  ##  ##  ##  ##  ##  ##  ##  ##  ##  ##  ##  ##  ##  ##  ##  ##  ##  ##  ##  ##  ##  ##  ##  ##  ##  ##  ##  ##  ##  ##  ##  ##  ##  ##  ##  ##  ##  ##  ##  ##  ##  ##  ##  ##  ##  ##  ##  ##  ## [2440_1]77	: ##  ##  ##  ##  ##  ##  ##  ##  ##  ##  ##  ##  ##  ##  ##  ##  ##  ##  ##  ##  ##  ##  ##  ##  ##  ##  ##  ##  ##  ##  ##  ##  ##  ##  ##  ##  ##  ##  ##  ##  ##  ##  ##  ##  ##  ##  ##  ##  ##  ##  ##  ##  ##  ##  ##  ##  ##  ##  ##  ##  ##  ##  ##  ##  ##  ##  ##  ##  ##  1  2  2  2  10  2  2  --  2  3  2  3  5  ##  ##  ##  ##  ##  ##  ##  ##  ##  ##  ##  ##  ##  ##  ##  ##  ##  ##  ##  ##  ##  ##  ##  ##  ##  ##  ##  ##  ##  ##  ##  ##  ##  ##  ##  ##  ##  ##  ##  ##  ##  ##  ##  ##  ##  ##  ##  ##  ##  ##  ##  ##  ##  ##  ##  ##  ##  ##  ##  ##  ##  ##  ##  ##  ##  ##  ##  ##  ##  ##  ##  ##  ##  ##  ##  ##  ##  ##  ##  ##  ##  ##  ##  ##  ##  ##  ##  ##  ##  ##  ##  ##  ##  ##  ##  ##  ##  ##  ##  ##  ##  ##  ##  ##  ##  ##  ##  ##  ##  ##  ##  ##  ##  ##  ##  ##  ##  ##  ##  ##  ##  ##  ##  ##  ##  ##  ##  ##  ##  ##  ##  ##  ##  ##  ##  ##  ##  ##  ##  ##  ##  ##  ##  ##  ##  ##  ##  ##  ##  ##  ##  ##  ##  ##  ##  ##  ##  ##  ##  ##  ##  ##  ##  ##  ##  ##  ##  ##  ##  ##  ##  ##  ##  ##  ##  ##  ##  ##  ##  ##  ##  ##  ##  ##  ##  ##  ##  ## [2445_1]78	: ##  ##  ##  ##  ##  ##  ##  ##  ##  ##  ##  ##  ##  ##  ##  ##  ##  ##  ##  ##  ##  ##  ##  ##  ##  ##  ##  ##  ##  ##  ##  ##  ##  ##  ##  ##  ##  ##  ##  ##  ##  ##  ##  ##  ##  ##  ##  ##  ##  ##  ##  ##  ##  ##  ##  ##  ##  ##  ##  ##  ##  ##  ##  ##  ##  ##  ##  ##  ##  1  2  2  2  10  2  2  2  --  3  2  3  4  ##  ##  ##  ##  ##  ##  ##  ##  ##  ##  ##  ##  ##  ##  ##  ##  ##  ##  ##  ##  ##  ##  ##  ##  ##  ##  ##  ##  ##  ##  ##  ##  ##  ##  ##  ##  ##  ##  ##  ##  ##  ##  ##  ##  ##  ##  ##  ##  ##  ##  ##  ##  ##  ##  ##  ##  ##  ##  ##  ##  ##  ##  ##  ##  ##  ##  ##  ##  ##  ##  ##  ##  ##  ##  ##  ##  ##  ##  ##  ##  ##  ##  ##  ##  ##  ##  ##  ##  ##  ##  ##  ##  ##  ##  ##  ##  ##  ##  ##  ##  ##  ##  ##  ##  ##  ##  ##  ##  ##  ##  ##  ##  ##  ##  ##  ##  ##  ##  ##  ##  ##  ##  ##  ##  ##  ##  ##  ##  ##  ##  ##  ##  ##  ##  ##  ##  ##  ##  ##  ##  ##  ##  ##  ##  ##  ##  ##  ##  ##  ##  ##  ##  ##  ##  ##  ##  ##  ##  ##  ##  ##  ##  ##  ##  ##  ##  ##  ##  ##  ##  ##  ##  ##  ##  ##  ##  ##  ##  ##  ##  ##  ##  ##  ##  ##  ##  ##  ## [2453_1]79	: ##  ##  ##  ##  ##  ##  ##  ##  ##  ##  ##  ##  ##  ##  ##  ##  ##  ##  ##  ##  ##  ##  ##  ##  ##  ##  ##  ##  ##  ##  ##  ##  ##  ##  ##  ##  ##  ##  ##  ##  ##  ##  ##  ##  ##  ##  ##  ##  ##  ##  ##  ##  ##  ##  ##  ##  ##  ##  ##  ##  ##  ##  ##  ##  ##  ##  ##  ##  ##  2  3  3  3  11  3  3  3  3  --  1  4  6  ##  ##  ##  ##  ##  ##  ##  ##  ##  ##  ##  ##  ##  ##  ##  ##  ##  ##  ##  ##  ##  ##  ##  ##  ##  ##  ##  ##  ##  ##  ##  ##  ##  ##  ##  ##  ##  ##  ##  ##  ##  ##  ##  ##  ##  ##  ##  ##  ##  ##  ##  ##  ##  ##  ##  ##  ##  ##  ##  ##  ##  ##  ##  ##  ##  ##  ##  ##  ##  ##  ##  ##  ##  ##  ##  ##  ##  ##  ##  ##  ##  ##  ##  ##  ##  ##  ##  ##  ##  ##  ##  ##  ##  ##  ##  ##  ##  ##  ##  ##  ##  ##  ##  ##  ##  ##  ##  ##  ##  ##  ##  ##  ##  ##  ##  ##  ##  ##  ##  ##  ##  ##  ##  ##  ##  ##  ##  ##  ##  ##  ##  ##  ##  ##  ##  ##  ##  ##  ##  ##  ##  ##  ##  ##  ##  ##  ##  ##  ##  ##  ##  ##  ##  ##  ##  ##  ##  ##  ##  ##  ##  ##  ##  ##  ##  ##  ##  ##  ##  ##  ##  ##  ##  ##  ##  ##  ##  ##  ##  ##  ##  ##  ##  ##  ##  ##  ##  ## [2796_1]80	: ##  ##  ##  ##  ##  ##  ##  ##  ##  ##  ##  ##  ##  ##  ##  ##  ##  ##  ##  ##  ##  ##  ##  ##  ##  ##  ##  ##  ##  ##  ##  ##  ##  ##  ##  ##  ##  ##  ##  ##  ##  ##  ##  ##  ##  ##  ##  ##  ##  ##  ##  ##  ##  ##  ##  ##  ##  ##  ##  ##  ##  ##  ##  ##  ##  ##  ##  ##  ##  1  2  2  2  10  2  2  2  2  1  --  3  5  ##  ##  ##  ##  ##  ##  ##  ##  ##  ##  ##  ##  ##  ##  ##  ##  ##  ##  ##  ##  ##  ##  ##  ##  ##  ##  ##  ##  ##  ##  ##  ##  ##  ##  ##  ##  ##  ##  ##  ##  ##  ##  ##  ##  ##  ##  ##  ##  ##  ##  ##  ##  ##  ##  ##  ##  ##  ##  ##  ##  ##  ##  ##  ##  ##  ##  ##  ##  ##  ##  ##  ##  ##  ##  ##  ##  ##  ##  ##  ##  ##  ##  ##  ##  ##  ##  ##  ##  ##  ##  ##  ##  ##  ##  ##  ##  ##  ##  ##  ##  ##  ##  ##  ##  ##  ##  ##  ##  ##  ##  ##  ##  ##  ##  ##  ##  ##  ##  ##  ##  ##  ##  ##  ##  ##  ##  ##  ##  ##  ##  ##  ##  ##  ##  ##  ##  ##  ##  ##  ##  ##  ##  ##  ##  ##  ##  ##  ##  ##  ##  ##  ##  ##  ##  ##  ##  ##  ##  ##  ##  ##  ##  ##  ##  ##  ##  ##  ##  ##  ##  ##  ##  ##  ##  ##  ##  ##  ##  ##  ##  ##  ##  ##  ##  ##  ##  ##  ## [T05_1]81	: ##  ##  ##  ##  ##  ##  ##  ##  ##  ##  ##  ##  ##  ##  ##  ##  ##  ##  ##  ##  ##  ##  ##  ##  ##  ##  ##  ##  ##  ##  ##  ##  ##  ##  ##  ##  ##  ##  ##  ##  ##  ##  ##  ##  ##  ##  ##  ##  ##  ##  ##  ##  ##  ##  ##  ##  ##  ##  ##  ##  ##  ##  ##  ##  ##  ##  ##  ##  ##  2  2  3  3  9  2  3  3  3  4  3  --  6  ##  ##  ##  ##  ##  ##  ##  ##  ##  ##  ##  ##  ##  ##  ##  ##  ##  ##  ##  ##  ##  ##  ##  ##  ##  ##  ##  ##  ##  ##  ##  ##  ##  ##  ##  ##  ##  ##  ##  ##  ##  ##  ##  ##  ##  ##  ##  ##  ##  ##  ##  ##  ##  ##  ##  ##  ##  ##  ##  ##  ##  ##  ##  ##  ##  ##  ##  ##  ##  ##  ##  ##  ##  ##  ##  ##  ##  ##  ##  ##  ##  ##  ##  ##  ##  ##  ##  ##  ##  ##  ##  ##  ##  ##  ##  ##  ##  ##  ##  ##  ##  ##  ##  ##  ##  ##  ##  ##  ##  ##  ##  ##  ##  ##  ##  ##  ##  ##  ##  ##  ##  ##  ##  ##  ##  ##  ##  ##  ##  ##  ##  ##  ##  ##  ##  ##  ##  ##  ##  ##  ##  ##  ##  ##  ##  ##  ##  ##  ##  ##  ##  ##  ##  ##  ##  ##  ##  ##  ##  ##  ##  ##  ##  ##  ##  ##  ##  ##  ##  ##  ##  ##  ##  ##  ##  ##  ##  ##  ##  ##  ##  ##  ##  ##  ##  ##  ##  ## [T06_1]82	: ##  ##  ##  ##  ##  ##  ##  ##  ##  ##  ##  ##  ##  ##  ##  ##  ##  ##  ##  ##  ##  ##  ##  ##  ##  ##  ##  ##  ##  ##  ##  ##  ##  ##  ##  ##  ##  ##  ##  ##  ##  ##  ##  ##  ##  ##  ##  ##  ##  ##  ##  ##  ##  ##  ##  ##  ##  ##  ##  ##  ##  ##  ##  ##  ##  ##  ##  ##  ##  4  5  5  5  10  5  3  5  4  6  5  6  --  ##  ##  ##  ##  ##  ##  ##  ##  ##  ##  ##  ##  ##  ##  ##  ##  ##  ##  ##  ##  ##  ##  ##  ##  ##  ##  ##  ##  ##  ##  ##  ##  ##  ##  ##  ##  ##  ##  ##  ##  ##  ##  ##  ##  ##  ##  ##  ##  ##  ##  ##  ##  ##  ##  ##  ##  ##  ##  ##  ##  ##  ##  ##  ##  ##  ##  ##  ##  ##  ##  ##  ##  ##  ##  ##  ##  ##  ##  ##  ##  ##  ##  ##  ##  ##  ##  ##  ##  ##  ##  ##  ##  ##  ##  ##  ##  ##  ##  ##  ##  ##  ##  ##  ##  ##  ##  ##  ##  ##  ##  ##  ##  ##  ##  ##  ##  ##  ##  ##  ##  ##  ##  ##  ##  ##  ##  ##  ##  ##  ##  ##  ##  ##  ##  ##  ##  ##  ##  ##  ##  ##  ##  ##  ##  ##  ##  ##  ##  ##  ##  ##  ##  ##  ##  ##  ##  ##  ##  ##  ##  ##  ##  ##  ##  ##  ##  ##  ##  ##  ##  ##  ##  ##  ##  ##  ##  ##  ##  ##  ##  ##  ##  ##  ##  ##  ##  ##  ## [2004_15]83	: ##  ##  ##  ##  ##  ##  ##  ##  ##  ##  ##  ##  ##  ##  ##  ##  ##  ##  ##  ##  ##  ##  ##  ##  ##  ##  ##  ##  ##  ##  ##  ##  ##  ##  ##  ##  ##  ##  ##  ##  ##  ##  ##  ##  ##  ##  ##  ##  ##  ##  ##  ##  ##  ##  ##  ##  ##  ##  ##  ##  ##  ##  ##  ##  ##  ##  ##  ##  ##  ##  ##  ##  ##  ##  ##  ##  ##  ##  ##  ##  ##  ##  --  1  2  1  ##  ##  ##  ##  ##  ##  ##  ##  ##  ##  ##  ##  ##  ##  ##  ##  ##  ##  ##  ##  ##  ##  ##  ##  ##  ##  ##  ##  ##  ##  ##  ##  ##  ##  ##  ##  ##  ##  ##  ##  ##  ##  ##  ##  ##  ##  ##  ##  ##  ##  ##  ##  ##  ##  ##  ##  ##  ##  ##  ##  ##  ##  ##  ##  ##  ##  ##  ##  ##  ##  ##  ##  ##  ##  ##  ##  ##  ##  ##  ##  ##  ##  ##  ##  ##  ##  ##  ##  ##  ##  ##  ##  ##  ##  ##  ##  ##  ##  ##  ##  ##  ##  ##  ##  ##  ##  ##  ##  ##  ##  ##  ##  ##  ##  ##  ##  ##  ##  ##  ##  ##  ##  ##  ##  ##  ##  ##  ##  ##  ##  ##  ##  ##  ##  ##  ##  ##  ##  ##  ##  ##  ##  ##  ##  ##  ##  ##  ##  ##  ##  ##  ##  ##  ##  ##  ##  ##  ##  ##  ##  ##  ##  ##  ##  ##  ##  ##  ##  ##  ##  ##  ##  ##  ##  ##  ##  ##  ##  ##  ##  ##  ##  ##  ## [2005_15]84	: ##  ##  ##  ##  ##  ##  ##  ##  ##  ##  ##  ##  ##  ##  ##  ##  ##  ##  ##  ##  ##  ##  ##  ##  ##  ##  ##  ##  ##  ##  ##  ##  ##  ##  ##  ##  ##  ##  ##  ##  ##  ##  ##  ##  ##  ##  ##  ##  ##  ##  ##  ##  ##  ##  ##  ##  ##  ##  ##  ##  ##  ##  ##  ##  ##  ##  ##  ##  ##  ##  ##  ##  ##  ##  ##  ##  ##  ##  ##  ##  ##  ##  1  --  1  2  ##  ##  ##  ##  ##  ##  ##  ##  ##  ##  ##  ##  ##  ##  ##  ##  ##  ##  ##  ##  ##  ##  ##  ##  ##  ##  ##  ##  ##  ##  ##  ##  ##  ##  ##  ##  ##  ##  ##  ##  ##  ##  ##  ##  ##  ##  ##  ##  ##  ##  ##  ##  ##  ##  ##  ##  ##  ##  ##  ##  ##  ##  ##  ##  ##  ##  ##  ##  ##  ##  ##  ##  ##  ##  ##  ##  ##  ##  ##  ##  ##  ##  ##  ##  ##  ##  ##  ##  ##  ##  ##  ##  ##  ##  ##  ##  ##  ##  ##  ##  ##  ##  ##  ##  ##  ##  ##  ##  ##  ##  ##  ##  ##  ##  ##  ##  ##  ##  ##  ##  ##  ##  ##  ##  ##  ##  ##  ##  ##  ##  ##  ##  ##  ##  ##  ##  ##  ##  ##  ##  ##  ##  ##  ##  ##  ##  ##  ##  ##  ##  ##  ##  ##  ##  ##  ##  ##  ##  ##  ##  ##  ##  ##  ##  ##  ##  ##  ##  ##  ##  ##  ##  ##  ##  ##  ##  ##  ##  ##  ##  ##  ##  ##  ## [2006_15]85	: ##  ##  ##  ##  ##  ##  ##  ##  ##  ##  ##  ##  ##  ##  ##  ##  ##  ##  ##  ##  ##  ##  ##  ##  ##  ##  ##  ##  ##  ##  ##  ##  ##  ##  ##  ##  ##  ##  ##  ##  ##  ##  ##  ##  ##  ##  ##  ##  ##  ##  ##  ##  ##  ##  ##  ##  ##  ##  ##  ##  ##  ##  ##  ##  ##  ##  ##  ##  ##  ##  ##  ##  ##  ##  ##  ##  ##  ##  ##  ##  ##  ##  2  1  --  3  ##  ##  ##  ##  ##  ##  ##  ##  ##  ##  ##  ##  ##  ##  ##  ##  ##  ##  ##  ##  ##  ##  ##  ##  ##  ##  ##  ##  ##  ##  ##  ##  ##  ##  ##  ##  ##  ##  ##  ##  ##  ##  ##  ##  ##  ##  ##  ##  ##  ##  ##  ##  ##  ##  ##  ##  ##  ##  ##  ##  ##  ##  ##  ##  ##  ##  ##  ##  ##  ##  ##  ##  ##  ##  ##  ##  ##  ##  ##  ##  ##  ##  ##  ##  ##  ##  ##  ##  ##  ##  ##  ##  ##  ##  ##  ##  ##  ##  ##  ##  ##  ##  ##  ##  ##  ##  ##  ##  ##  ##  ##  ##  ##  ##  ##  ##  ##  ##  ##  ##  ##  ##  ##  ##  ##  ##  ##  ##  ##  ##  ##  ##  ##  ##  ##  ##  ##  ##  ##  ##  ##  ##  ##  ##  ##  ##  ##  ##  ##  ##  ##  ##  ##  ##  ##  ##  ##  ##  ##  ##  ##  ##  ##  ##  ##  ##  ##  ##  ##  ##  ##  ##  ##  ##  ##  ##  ##  ##  ##  ##  ##  ##  ##  ## [2030_15]86	: ##  ##  ##  ##  ##  ##  ##  ##  ##  ##  ##  ##  ##  ##  ##  ##  ##  ##  ##  ##  ##  ##  ##  ##  ##  ##  ##  ##  ##  ##  ##  ##  ##  ##  ##  ##  ##  ##  ##  ##  ##  ##  ##  ##  ##  ##  ##  ##  ##  ##  ##  ##  ##  ##  ##  ##  ##  ##  ##  ##  ##  ##  ##  ##  ##  ##  ##  ##  ##  ##  ##  ##  ##  ##  ##  ##  ##  ##  ##  ##  ##  ##  1  2  3  --  ##  ##  ##  ##  ##  ##  ##  ##  ##  ##  ##  ##  ##  ##  ##  ##  ##  ##  ##  ##  ##  ##  ##  ##  ##  ##  ##  ##  ##  ##  ##  ##  ##  ##  ##  ##  ##  ##  ##  ##  ##  ##  ##  ##  ##  ##  ##  ##  ##  ##  ##  ##  ##  ##  ##  ##  ##  ##  ##  ##  ##  ##  ##  ##  ##  ##  ##  ##  ##  ##  ##  ##  ##  ##  ##  ##  ##  ##  ##  ##  ##  ##  ##  ##  ##  ##  ##  ##  ##  ##  ##  ##  ##  ##  ##  ##  ##  ##  ##  ##  ##  ##  ##  ##  ##  ##  ##  ##  ##  ##  ##  ##  ##  ##  ##  ##  ##  ##  ##  ##  ##  ##  ##  ##  ##  ##  ##  ##  ##  ##  ##  ##  ##  ##  ##  ##  ##  ##  ##  ##  ##  ##  ##  ##  ##  ##  ##  ##  ##  ##  ##  ##  ##  ##  ##  ##  ##  ##  ##  ##  ##  ##  ##  ##  ##  ##  ##  ##  ##  ##  ##  ##  ##  ##  ##  ##  ##  ##  ##  ##  ##  ##  ##  ## [2864_24]87	: ##  ##  ##  ##  ##  ##  ##  ##  ##  ##  ##  ##  ##  ##  ##  ##  ##  ##  ##  ##  ##  ##  ##  ##  ##  ##  ##  ##  ##  ##  ##  ##  ##  ##  ##  ##  ##  ##  ##  ##  ##  ##  ##  ##  ##  ##  ##  ##  ##  ##  ##  ##  ##  ##  ##  ##  ##  ##  ##  ##  ##  ##  ##  ##  ##  ##  ##  ##  ##  ##  ##  ##  ##  ##  ##  ##  ##  ##  ##  ##  ##  ##  ##  ##  ##  ##  --  1  ##  ##  ##  ##  ##  ##  ##  ##  ##  ##  ##  ##  ##  ##  ##  ##  ##  ##  ##  ##  ##  ##  ##  ##  ##  ##  ##  ##  ##  ##  ##  ##  ##  ##  ##  ##  ##  ##  ##  ##  ##  ##  ##  ##  ##  ##  ##  ##  ##  ##  ##  ##  ##  ##  ##  ##  ##  ##  ##  ##  ##  ##  ##  ##  ##  ##  ##  ##  ##  ##  ##  ##  ##  ##  ##  ##  ##  ##  ##  ##  ##  ##  ##  ##  ##  ##  ##  ##  ##  ##  ##  ##  ##  ##  ##  ##  ##  ##  ##  ##  ##  ##  ##  ##  ##  ##  ##  ##  ##  ##  ##  ##  ##  ##  ##  ##  ##  ##  ##  ##  ##  ##  ##  ##  ##  ##  ##  ##  ##  ##  ##  ##  ##  ##  ##  ##  ##  ##  ##  ##  ##  ##  ##  ##  ##  ##  ##  ##  ##  ##  ##  ##  ##  ##  ##  ##  ##  ##  ##  ##  ##  ##  ##  ##  ##  ##  ##  ##  ##  ##  ##  ##  ##  ##  ##  ##  ##  ##  ##  ##  ##  ## [2869_24]88	: ##  ##  ##  ##  ##  ##  ##  ##  ##  ##  ##  ##  ##  ##  ##  ##  ##  ##  ##  ##  ##  ##  ##  ##  ##  ##  ##  ##  ##  ##  ##  ##  ##  ##  ##  ##  ##  ##  ##  ##  ##  ##  ##  ##  ##  ##  ##  ##  ##  ##  ##  ##  ##  ##  ##  ##  ##  ##  ##  ##  ##  ##  ##  ##  ##  ##  ##  ##  ##  ##  ##  ##  ##  ##  ##  ##  ##  ##  ##  ##  ##  ##  ##  ##  ##  ##  1  --  ##  ##  ##  ##  ##  ##  ##  ##  ##  ##  ##  ##  ##  ##  ##  ##  ##  ##  ##  ##  ##  ##  ##  ##  ##  ##  ##  ##  ##  ##  ##  ##  ##  ##  ##  ##  ##  ##  ##  ##  ##  ##  ##  ##  ##  ##  ##  ##  ##  ##  ##  ##  ##  ##  ##  ##  ##  ##  ##  ##  ##  ##  ##  ##  ##  ##  ##  ##  ##  ##  ##  ##  ##  ##  ##  ##  ##  ##  ##  ##  ##  ##  ##  ##  ##  ##  ##  ##  ##  ##  ##  ##  ##  ##  ##  ##  ##  ##  ##  ##  ##  ##  ##  ##  ##  ##  ##  ##  ##  ##  ##  ##  ##  ##  ##  ##  ##  ##  ##  ##  ##  ##  ##  ##  ##  ##  ##  ##  ##  ##  ##  ##  ##  ##  ##  ##  ##  ##  ##  ##  ##  ##  ##  ##  ##  ##  ##  ##  ##  ##  ##  ##  ##  ##  ##  ##  ##  ##  ##  ##  ##  ##  ##  ##  ##  ##  ##  ##  ##  ##  ##  ##  ##  ##  ##  ##  ##  ##  ##  ##  ##  ## [2801_25]89	: ##  ##  ##  ##  ##  ##  ##  ##  ##  ##  ##  ##  ##  ##  ##  ##  ##  ##  ##  ##  ##  ##  ##  ##  ##  ##  ##  ##  ##  ##  ##  ##  ##  ##  ##  ##  ##  ##  ##  ##  ##  ##  ##  ##  ##  ##  ##  ##  ##  ##  ##  ##  ##  ##  ##  ##  ##  ##  ##  ##  ##  ##  ##  ##  ##  ##  ##  ##  ##  ##  ##  ##  ##  ##  ##  ##  ##  ##  ##  ##  ##  ##  ##  ##  ##  ##  ##  ##  --  5  ##  ##  ##  ##  ##  ##  ##  ##  ##  ##  ##  ##  ##  ##  ##  ##  ##  ##  ##  ##  ##  ##  ##  ##  ##  ##  ##  ##  ##  ##  ##  ##  ##  ##  ##  ##  ##  ##  ##  ##  ##  ##  ##  ##  ##  ##  ##  ##  ##  ##  ##  ##  ##  ##  ##  ##  ##  ##  ##  ##  ##  ##  ##  ##  ##  ##  ##  ##  ##  ##  ##  ##  ##  ##  ##  ##  ##  ##  ##  ##  ##  ##  ##  ##  ##  ##  ##  ##  ##  ##  ##  ##  ##  ##  ##  ##  ##  ##  ##  ##  ##  ##  ##  ##  ##  ##  ##  ##  ##  ##  ##  ##  ##  ##  ##  ##  ##  ##  ##  ##  ##  ##  ##  ##  ##  ##  ##  ##  ##  ##  ##  ##  ##  ##  ##  ##  ##  ##  ##  ##  ##  ##  ##  ##  ##  ##  ##  ##  ##  ##  ##  ##  ##  ##  ##  ##  ##  ##  ##  ##  ##  ##  ##  ##  ##  ##  ##  ##  ##  ##  ##  ##  ##  ##  ##  ##  ##  ##  ##  ## [2809_25]90	: ##  ##  ##  ##  ##  ##  ##  ##  ##  ##  ##  ##  ##  ##  ##  ##  ##  ##  ##  ##  ##  ##  ##  ##  ##  ##  ##  ##  ##  ##  ##  ##  ##  ##  ##  ##  ##  ##  ##  ##  ##  ##  ##  ##  ##  ##  ##  ##  ##  ##  ##  ##  ##  ##  ##  ##  ##  ##  ##  ##  ##  ##  ##  ##  ##  ##  ##  ##  ##  ##  ##  ##  ##  ##  ##  ##  ##  ##  ##  ##  ##  ##  ##  ##  ##  ##  ##  ##  5  --  ##  ##  ##  ##  ##  ##  ##  ##  ##  ##  ##  ##  ##  ##  ##  ##  ##  ##  ##  ##  ##  ##  ##  ##  ##  ##  ##  ##  ##  ##  ##  ##  ##  ##  ##  ##  ##  ##  ##  ##  ##  ##  ##  ##  ##  ##  ##  ##  ##  ##  ##  ##  ##  ##  ##  ##  ##  ##  ##  ##  ##  ##  ##  ##  ##  ##  ##  ##  ##  ##  ##  ##  ##  ##  ##  ##  ##  ##  ##  ##  ##  ##  ##  ##  ##  ##  ##  ##  ##  ##  ##  ##  ##  ##  ##  ##  ##  ##  ##  ##  ##  ##  ##  ##  ##  ##  ##  ##  ##  ##  ##  ##  ##  ##  ##  ##  ##  ##  ##  ##  ##  ##  ##  ##  ##  ##  ##  ##  ##  ##  ##  ##  ##  ##  ##  ##  ##  ##  ##  ##  ##  ##  ##  ##  ##  ##  ##  ##  ##  ##  ##  ##  ##  ##  ##  ##  ##  ##  ##  ##  ##  ##  ##  ##  ##  ##  ##  ##  ##  ##  ##  ##  ##  ##  ##  ##  ##  ##  ##  ## [838_6]91	: ##  ##  ##  ##  ##  ##  ##  ##  ##  ##  ##  ##  ##  ##  ##  ##  ##  ##  ##  ##  ##  ##  ##  ##  ##  ##  ##  ##  ##  ##  ##  ##  ##  ##  ##  ##  ##  ##  ##  ##  ##  ##  ##  ##  ##  ##  ##  ##  ##  ##  ##  ##  ##  ##  ##  ##  ##  ##  ##  ##  ##  ##  ##  ##  ##  ##  ##  ##  ##  ##  ##  ##  ##  ##  ##  ##  ##  ##  ##  ##  ##  ##  ##  ##  ##  ##  ##  ##  ##  ##  --  1  4  1  1  4  2  2  1  2  1  ##  ##  ##  ##  ##  ##  ##  ##  ##  ##  ##  ##  ##  ##  ##  ##  ##  ##  ##  ##  ##  ##  ##  ##  ##  ##  ##  ##  ##  ##  ##  ##  ##  ##  ##  ##  ##  ##  ##  ##  ##  ##  ##  ##  ##  ##  ##  ##  ##  ##  ##  ##  ##  ##  ##  ##  ##  ##  ##  ##  ##  ##  ##  ##  ##  ##  ##  ##  ##  ##  ##  ##  ##  ##  ##  ##  ##  ##  ##  ##  ##  ##  ##  ##  ##  ##  ##  ##  ##  ##  ##  ##  ##  ##  ##  ##  ##  ##  ##  ##  ##  ##  ##  ##  ##  ##  ##  ##  ##  ##  ##  ##  ##  ##  ##  ##  ##  ##  ##  ##  ##  ##  ##  ##  ##  ##  ##  ##  ##  ##  ##  ##  ##  ##  ##  ##  ##  ##  ##  ##  ##  ##  ##  ##  ##  ##  ##  ##  ##  ##  ##  ##  ##  ##  ##  ##  ##  ##  ##  ##  ##  ##  ##  ##  ##  ##  ##  ##  ## [839_6]92	: ##  ##  ##  ##  ##  ##  ##  ##  ##  ##  ##  ##  ##  ##  ##  ##  ##  ##  ##  ##  ##  ##  ##  ##  ##  ##  ##  ##  ##  ##  ##  ##  ##  ##  ##  ##  ##  ##  ##  ##  ##  ##  ##  ##  ##  ##  ##  ##  ##  ##  ##  ##  ##  ##  ##  ##  ##  ##  ##  ##  ##  ##  ##  ##  ##  ##  ##  ##  ##  ##  ##  ##  ##  ##  ##  ##  ##  ##  ##  ##  ##  ##  ##  ##  ##  ##  ##  ##  ##  ##  1  --  4  2  1  3  2  1  1  1  2  ##  ##  ##  ##  ##  ##  ##  ##  ##  ##  ##  ##  ##  ##  ##  ##  ##  ##  ##  ##  ##  ##  ##  ##  ##  ##  ##  ##  ##  ##  ##  ##  ##  ##  ##  ##  ##  ##  ##  ##  ##  ##  ##  ##  ##  ##  ##  ##  ##  ##  ##  ##  ##  ##  ##  ##  ##  ##  ##  ##  ##  ##  ##  ##  ##  ##  ##  ##  ##  ##  ##  ##  ##  ##  ##  ##  ##  ##  ##  ##  ##  ##  ##  ##  ##  ##  ##  ##  ##  ##  ##  ##  ##  ##  ##  ##  ##  ##  ##  ##  ##  ##  ##  ##  ##  ##  ##  ##  ##  ##  ##  ##  ##  ##  ##  ##  ##  ##  ##  ##  ##  ##  ##  ##  ##  ##  ##  ##  ##  ##  ##  ##  ##  ##  ##  ##  ##  ##  ##  ##  ##  ##  ##  ##  ##  ##  ##  ##  ##  ##  ##  ##  ##  ##  ##  ##  ##  ##  ##  ##  ##  ##  ##  ##  ##  ##  ##  ##  ## [850_6]93	: ##  ##  ##  ##  ##  ##  ##  ##  ##  ##  ##  ##  ##  ##  ##  ##  ##  ##  ##  ##  ##  ##  ##  ##  ##  ##  ##  ##  ##  ##  ##  ##  ##  ##  ##  ##  ##  ##  ##  ##  ##  ##  ##  ##  ##  ##  ##  ##  ##  ##  ##  ##  ##  ##  ##  ##  ##  ##  ##  ##  ##  ##  ##  ##  ##  ##  ##  ##  ##  ##  ##  ##  ##  ##  ##  ##  ##  ##  ##  ##  ##  ##  ##  ##  ##  ##  ##  ##  ##  ##  4  4  --  5  1  3  2  5  5  5  5  ##  ##  ##  ##  ##  ##  ##  ##  ##  ##  ##  ##  ##  ##  ##  ##  ##  ##  ##  ##  ##  ##  ##  ##  ##  ##  ##  ##  ##  ##  ##  ##  ##  ##  ##  ##  ##  ##  ##  ##  ##  ##  ##  ##  ##  ##  ##  ##  ##  ##  ##  ##  ##  ##  ##  ##  ##  ##  ##  ##  ##  ##  ##  ##  ##  ##  ##  ##  ##  ##  ##  ##  ##  ##  ##  ##  ##  ##  ##  ##  ##  ##  ##  ##  ##  ##  ##  ##  ##  ##  ##  ##  ##  ##  ##  ##  ##  ##  ##  ##  ##  ##  ##  ##  ##  ##  ##  ##  ##  ##  ##  ##  ##  ##  ##  ##  ##  ##  ##  ##  ##  ##  ##  ##  ##  ##  ##  ##  ##  ##  ##  ##  ##  ##  ##  ##  ##  ##  ##  ##  ##  ##  ##  ##  ##  ##  ##  ##  ##  ##  ##  ##  ##  ##  ##  ##  ##  ##  ##  ##  ##  ##  ##  ##  ##  ##  ##  ##  ## [1313_6]94	: ##  ##  ##  ##  ##  ##  ##  ##  ##  ##  ##  ##  ##  ##  ##  ##  ##  ##  ##  ##  ##  ##  ##  ##  ##  ##  ##  ##  ##  ##  ##  ##  ##  ##  ##  ##  ##  ##  ##  ##  ##  ##  ##  ##  ##  ##  ##  ##  ##  ##  ##  ##  ##  ##  ##  ##  ##  ##  ##  ##  ##  ##  ##  ##  ##  ##  ##  ##  ##  ##  ##  ##  ##  ##  ##  ##  ##  ##  ##  ##  ##  ##  ##  ##  ##  ##  ##  ##  ##  ##  1  2  5  --  2  5  3  3  2  2  2  ##  ##  ##  ##  ##  ##  ##  ##  ##  ##  ##  ##  ##  ##  ##  ##  ##  ##  ##  ##  ##  ##  ##  ##  ##  ##  ##  ##  ##  ##  ##  ##  ##  ##  ##  ##  ##  ##  ##  ##  ##  ##  ##  ##  ##  ##  ##  ##  ##  ##  ##  ##  ##  ##  ##  ##  ##  ##  ##  ##  ##  ##  ##  ##  ##  ##  ##  ##  ##  ##  ##  ##  ##  ##  ##  ##  ##  ##  ##  ##  ##  ##  ##  ##  ##  ##  ##  ##  ##  ##  ##  ##  ##  ##  ##  ##  ##  ##  ##  ##  ##  ##  ##  ##  ##  ##  ##  ##  ##  ##  ##  ##  ##  ##  ##  ##  ##  ##  ##  ##  ##  ##  ##  ##  ##  ##  ##  ##  ##  ##  ##  ##  ##  ##  ##  ##  ##  ##  ##  ##  ##  ##  ##  ##  ##  ##  ##  ##  ##  ##  ##  ##  ##  ##  ##  ##  ##  ##  ##  ##  ##  ##  ##  ##  ##  ##  ##  ##  ## [1314_6]95	: ##  ##  ##  ##  ##  ##  ##  ##  ##  ##  ##  ##  ##  ##  ##  ##  ##  ##  ##  ##  ##  ##  ##  ##  ##  ##  ##  ##  ##  ##  ##  ##  ##  ##  ##  ##  ##  ##  ##  ##  ##  ##  ##  ##  ##  ##  ##  ##  ##  ##  ##  ##  ##  ##  ##  ##  ##  ##  ##  ##  ##  ##  ##  ##  ##  ##  ##  ##  ##  ##  ##  ##  ##  ##  ##  ##  ##  ##  ##  ##  ##  ##  ##  ##  ##  ##  ##  ##  ##  ##  1  1  1  2  --  2  1  2  2  2  1  ##  ##  ##  ##  ##  ##  ##  ##  ##  ##  ##  ##  ##  ##  ##  ##  ##  ##  ##  ##  ##  ##  ##  ##  ##  ##  ##  ##  ##  ##  ##  ##  ##  ##  ##  ##  ##  ##  ##  ##  ##  ##  ##  ##  ##  ##  ##  ##  ##  ##  ##  ##  ##  ##  ##  ##  ##  ##  ##  ##  ##  ##  ##  ##  ##  ##  ##  ##  ##  ##  ##  ##  ##  ##  ##  ##  ##  ##  ##  ##  ##  ##  ##  ##  ##  ##  ##  ##  ##  ##  ##  ##  ##  ##  ##  ##  ##  ##  ##  ##  ##  ##  ##  ##  ##  ##  ##  ##  ##  ##  ##  ##  ##  ##  ##  ##  ##  ##  ##  ##  ##  ##  ##  ##  ##  ##  ##  ##  ##  ##  ##  ##  ##  ##  ##  ##  ##  ##  ##  ##  ##  ##  ##  ##  ##  ##  ##  ##  ##  ##  ##  ##  ##  ##  ##  ##  ##  ##  ##  ##  ##  ##  ##  ##  ##  ##  ##  ##  ## [1319_6]96	: ##  ##  ##  ##  ##  ##  ##  ##  ##  ##  ##  ##  ##  ##  ##  ##  ##  ##  ##  ##  ##  ##  ##  ##  ##  ##  ##  ##  ##  ##  ##  ##  ##  ##  ##  ##  ##  ##  ##  ##  ##  ##  ##  ##  ##  ##  ##  ##  ##  ##  ##  ##  ##  ##  ##  ##  ##  ##  ##  ##  ##  ##  ##  ##  ##  ##  ##  ##  ##  ##  ##  ##  ##  ##  ##  ##  ##  ##  ##  ##  ##  ##  ##  ##  ##  ##  ##  ##  ##  ##  4  3  3  5  2  --  1  4  4  4  5  ##  ##  ##  ##  ##  ##  ##  ##  ##  ##  ##  ##  ##  ##  ##  ##  ##  ##  ##  ##  ##  ##  ##  ##  ##  ##  ##  ##  ##  ##  ##  ##  ##  ##  ##  ##  ##  ##  ##  ##  ##  ##  ##  ##  ##  ##  ##  ##  ##  ##  ##  ##  ##  ##  ##  ##  ##  ##  ##  ##  ##  ##  ##  ##  ##  ##  ##  ##  ##  ##  ##  ##  ##  ##  ##  ##  ##  ##  ##  ##  ##  ##  ##  ##  ##  ##  ##  ##  ##  ##  ##  ##  ##  ##  ##  ##  ##  ##  ##  ##  ##  ##  ##  ##  ##  ##  ##  ##  ##  ##  ##  ##  ##  ##  ##  ##  ##  ##  ##  ##  ##  ##  ##  ##  ##  ##  ##  ##  ##  ##  ##  ##  ##  ##  ##  ##  ##  ##  ##  ##  ##  ##  ##  ##  ##  ##  ##  ##  ##  ##  ##  ##  ##  ##  ##  ##  ##  ##  ##  ##  ##  ##  ##  ##  ##  ##  ##  ##  ## [1870_6]97	: ##  ##  ##  ##  ##  ##  ##  ##  ##  ##  ##  ##  ##  ##  ##  ##  ##  ##  ##  ##  ##  ##  ##  ##  ##  ##  ##  ##  ##  ##  ##  ##  ##  ##  ##  ##  ##  ##  ##  ##  ##  ##  ##  ##  ##  ##  ##  ##  ##  ##  ##  ##  ##  ##  ##  ##  ##  ##  ##  ##  ##  ##  ##  ##  ##  ##  ##  ##  ##  ##  ##  ##  ##  ##  ##  ##  ##  ##  ##  ##  ##  ##  ##  ##  ##  ##  ##  ##  ##  ##  2  2  2  3  1  1  --  3  3  3  3  ##  ##  ##  ##  ##  ##  ##  ##  ##  ##  ##  ##  ##  ##  ##  ##  ##  ##  ##  ##  ##  ##  ##  ##  ##  ##  ##  ##  ##  ##  ##  ##  ##  ##  ##  ##  ##  ##  ##  ##  ##  ##  ##  ##  ##  ##  ##  ##  ##  ##  ##  ##  ##  ##  ##  ##  ##  ##  ##  ##  ##  ##  ##  ##  ##  ##  ##  ##  ##  ##  ##  ##  ##  ##  ##  ##  ##  ##  ##  ##  ##  ##  ##  ##  ##  ##  ##  ##  ##  ##  ##  ##  ##  ##  ##  ##  ##  ##  ##  ##  ##  ##  ##  ##  ##  ##  ##  ##  ##  ##  ##  ##  ##  ##  ##  ##  ##  ##  ##  ##  ##  ##  ##  ##  ##  ##  ##  ##  ##  ##  ##  ##  ##  ##  ##  ##  ##  ##  ##  ##  ##  ##  ##  ##  ##  ##  ##  ##  ##  ##  ##  ##  ##  ##  ##  ##  ##  ##  ##  ##  ##  ##  ##  ##  ##  ##  ##  ##  ## [1871_6]98	: ##  ##  ##  ##  ##  ##  ##  ##  ##  ##  ##  ##  ##  ##  ##  ##  ##  ##  ##  ##  ##  ##  ##  ##  ##  ##  ##  ##  ##  ##  ##  ##  ##  ##  ##  ##  ##  ##  ##  ##  ##  ##  ##  ##  ##  ##  ##  ##  ##  ##  ##  ##  ##  ##  ##  ##  ##  ##  ##  ##  ##  ##  ##  ##  ##  ##  ##  ##  ##  ##  ##  ##  ##  ##  ##  ##  ##  ##  ##  ##  ##  ##  ##  ##  ##  ##  ##  ##  ##  ##  2  1  5  3  2  4  3  --  2  2  3  ##  ##  ##  ##  ##  ##  ##  ##  ##  ##  ##  ##  ##  ##  ##  ##  ##  ##  ##  ##  ##  ##  ##  ##  ##  ##  ##  ##  ##  ##  ##  ##  ##  ##  ##  ##  ##  ##  ##  ##  ##  ##  ##  ##  ##  ##  ##  ##  ##  ##  ##  ##  ##  ##  ##  ##  ##  ##  ##  ##  ##  ##  ##  ##  ##  ##  ##  ##  ##  ##  ##  ##  ##  ##  ##  ##  ##  ##  ##  ##  ##  ##  ##  ##  ##  ##  ##  ##  ##  ##  ##  ##  ##  ##  ##  ##  ##  ##  ##  ##  ##  ##  ##  ##  ##  ##  ##  ##  ##  ##  ##  ##  ##  ##  ##  ##  ##  ##  ##  ##  ##  ##  ##  ##  ##  ##  ##  ##  ##  ##  ##  ##  ##  ##  ##  ##  ##  ##  ##  ##  ##  ##  ##  ##  ##  ##  ##  ##  ##  ##  ##  ##  ##  ##  ##  ##  ##  ##  ##  ##  ##  ##  ##  ##  ##  ##  ##  ##  ## [1872_6]99	: ##  ##  ##  ##  ##  ##  ##  ##  ##  ##  ##  ##  ##  ##  ##  ##  ##  ##  ##  ##  ##  ##  ##  ##  ##  ##  ##  ##  ##  ##  ##  ##  ##  ##  ##  ##  ##  ##  ##  ##  ##  ##  ##  ##  ##  ##  ##  ##  ##  ##  ##  ##  ##  ##  ##  ##  ##  ##  ##  ##  ##  ##  ##  ##  ##  ##  ##  ##  ##  ##  ##  ##  ##  ##  ##  ##  ##  ##  ##  ##  ##  ##  ##  ##  ##  ##  ##  ##  ##  ##  1  1  5  2  2  4  3  2  --  2  2  ##  ##  ##  ##  ##  ##  ##  ##  ##  ##  ##  ##  ##  ##  ##  ##  ##  ##  ##  ##  ##  ##  ##  ##  ##  ##  ##  ##  ##  ##  ##  ##  ##  ##  ##  ##  ##  ##  ##  ##  ##  ##  ##  ##  ##  ##  ##  ##  ##  ##  ##  ##  ##  ##  ##  ##  ##  ##  ##  ##  ##  ##  ##  ##  ##  ##  ##  ##  ##  ##  ##  ##  ##  ##  ##  ##  ##  ##  ##  ##  ##  ##  ##  ##  ##  ##  ##  ##  ##  ##  ##  ##  ##  ##  ##  ##  ##  ##  ##  ##  ##  ##  ##  ##  ##  ##  ##  ##  ##  ##  ##  ##  ##  ##  ##  ##  ##  ##  ##  ##  ##  ##  ##  ##  ##  ##  ##  ##  ##  ##  ##  ##  ##  ##  ##  ##  ##  ##  ##  ##  ##  ##  ##  ##  ##  ##  ##  ##  ##  ##  ##  ##  ##  ##  ##  ##  ##  ##  ##  ##  ##  ##  ##  ##  ##  ##  ##  ##  ## [1875_6]100	: ##  ##  ##  ##  ##  ##  ##  ##  ##  ##  ##  ##  ##  ##  ##  ##  ##  ##  ##  ##  ##  ##  ##  ##  ##  ##  ##  ##  ##  ##  ##  ##  ##  ##  ##  ##  ##  ##  ##  ##  ##  ##  ##  ##  ##  ##  ##  ##  ##  ##  ##  ##  ##  ##  ##  ##  ##  ##  ##  ##  ##  ##  ##  ##  ##  ##  ##  ##  ##  ##  ##  ##  ##  ##  ##  ##  ##  ##  ##  ##  ##  ##  ##  ##  ##  ##  ##  ##  ##  ##  2  1  5  2  2  4  3  2  2  --  3  ##  ##  ##  ##  ##  ##  ##  ##  ##  ##  ##  ##  ##  ##  ##  ##  ##  ##  ##  ##  ##  ##  ##  ##  ##  ##  ##  ##  ##  ##  ##  ##  ##  ##  ##  ##  ##  ##  ##  ##  ##  ##  ##  ##  ##  ##  ##  ##  ##  ##  ##  ##  ##  ##  ##  ##  ##  ##  ##  ##  ##  ##  ##  ##  ##  ##  ##  ##  ##  ##  ##  ##  ##  ##  ##  ##  ##  ##  ##  ##  ##  ##  ##  ##  ##  ##  ##  ##  ##  ##  ##  ##  ##  ##  ##  ##  ##  ##  ##  ##  ##  ##  ##  ##  ##  ##  ##  ##  ##  ##  ##  ##  ##  ##  ##  ##  ##  ##  ##  ##  ##  ##  ##  ##  ##  ##  ##  ##  ##  ##  ##  ##  ##  ##  ##  ##  ##  ##  ##  ##  ##  ##  ##  ##  ##  ##  ##  ##  ##  ##  ##  ##  ##  ##  ##  ##  ##  ##  ##  ##  ##  ##  ##  ##  ##  ##  ##  ##  ## [2167_6]101	: ##  ##  ##  ##  ##  ##  ##  ##  ##  ##  ##  ##  ##  ##  ##  ##  ##  ##  ##  ##  ##  ##  ##  ##  ##  ##  ##  ##  ##  ##  ##  ##  ##  ##  ##  ##  ##  ##  ##  ##  ##  ##  ##  ##  ##  ##  ##  ##  ##  ##  ##  ##  ##  ##  ##  ##  ##  ##  ##  ##  ##  ##  ##  ##  ##  ##  ##  ##  ##  ##  ##  ##  ##  ##  ##  ##  ##  ##  ##  ##  ##  ##  ##  ##  ##  ##  ##  ##  ##  ##  1  2  5  2  1  5  3  3  2  3  --  ##  ##  ##  ##  ##  ##  ##  ##  ##  ##  ##  ##  ##  ##  ##  ##  ##  ##  ##  ##  ##  ##  ##  ##  ##  ##  ##  ##  ##  ##  ##  ##  ##  ##  ##  ##  ##  ##  ##  ##  ##  ##  ##  ##  ##  ##  ##  ##  ##  ##  ##  ##  ##  ##  ##  ##  ##  ##  ##  ##  ##  ##  ##  ##  ##  ##  ##  ##  ##  ##  ##  ##  ##  ##  ##  ##  ##  ##  ##  ##  ##  ##  ##  ##  ##  ##  ##  ##  ##  ##  ##  ##  ##  ##  ##  ##  ##  ##  ##  ##  ##  ##  ##  ##  ##  ##  ##  ##  ##  ##  ##  ##  ##  ##  ##  ##  ##  ##  ##  ##  ##  ##  ##  ##  ##  ##  ##  ##  ##  ##  ##  ##  ##  ##  ##  ##  ##  ##  ##  ##  ##  ##  ##  ##  ##  ##  ##  ##  ##  ##  ##  ##  ##  ##  ##  ##  ##  ##  ##  ##  ##  ##  ##  ##  ##  ##  ##  ##  ## [829_7]102	: ##  ##  ##  ##  ##  ##  ##  ##  ##  ##  ##  ##  ##  ##  ##  ##  ##  ##  ##  ##  ##  ##  ##  ##  ##  ##  ##  ##  ##  ##  ##  ##  ##  ##  ##  ##  ##  ##  ##  ##  ##  ##  ##  ##  ##  ##  ##  ##  ##  ##  ##  ##  ##  ##  ##  ##  ##  ##  ##  ##  ##  ##  ##  ##  ##  ##  ##  ##  ##  ##  ##  ##  ##  ##  ##  ##  ##  ##  ##  ##  ##  ##  ##  ##  ##  ##  ##  ##  ##  ##  ##  ##  ##  ##  ##  ##  ##  ##  ##  ##  ##  --  2  1  1  2  2  1  2  ##  ##  ##  ##  ##  ##  ##  ##  ##  ##  ##  ##  ##  ##  ##  ##  ##  ##  ##  ##  ##  ##  ##  ##  ##  ##  ##  ##  ##  ##  ##  ##  ##  ##  ##  ##  ##  ##  ##  ##  ##  ##  ##  ##  ##  ##  ##  ##  ##  ##  ##  ##  ##  ##  ##  ##  ##  ##  ##  ##  ##  ##  ##  ##  ##  ##  ##  ##  ##  ##  ##  ##  ##  ##  ##  ##  ##  ##  ##  ##  ##  ##  ##  ##  ##  ##  ##  ##  ##  ##  ##  ##  ##  ##  ##  ##  ##  ##  ##  ##  ##  ##  ##  ##  ##  ##  ##  ##  ##  ##  ##  ##  ##  ##  ##  ##  ##  ##  ##  ##  ##  ##  ##  ##  ##  ##  ##  ##  ##  ##  ##  ##  ##  ##  ##  ##  ##  ##  ##  ##  ##  ##  ##  ##  ##  ##  ##  ##  ##  ##  ##  ##  ##  ##  ##  ##  ##  ##  ##  ##  ## [1309_7]103	: ##  ##  ##  ##  ##  ##  ##  ##  ##  ##  ##  ##  ##  ##  ##  ##  ##  ##  ##  ##  ##  ##  ##  ##  ##  ##  ##  ##  ##  ##  ##  ##  ##  ##  ##  ##  ##  ##  ##  ##  ##  ##  ##  ##  ##  ##  ##  ##  ##  ##  ##  ##  ##  ##  ##  ##  ##  ##  ##  ##  ##  ##  ##  ##  ##  ##  ##  ##  ##  ##  ##  ##  ##  ##  ##  ##  ##  ##  ##  ##  ##  ##  ##  ##  ##  ##  ##  ##  ##  ##  ##  ##  ##  ##  ##  ##  ##  ##  ##  ##  ##  2  --  2  2  3  3  2  3  ##  ##  ##  ##  ##  ##  ##  ##  ##  ##  ##  ##  ##  ##  ##  ##  ##  ##  ##  ##  ##  ##  ##  ##  ##  ##  ##  ##  ##  ##  ##  ##  ##  ##  ##  ##  ##  ##  ##  ##  ##  ##  ##  ##  ##  ##  ##  ##  ##  ##  ##  ##  ##  ##  ##  ##  ##  ##  ##  ##  ##  ##  ##  ##  ##  ##  ##  ##  ##  ##  ##  ##  ##  ##  ##  ##  ##  ##  ##  ##  ##  ##  ##  ##  ##  ##  ##  ##  ##  ##  ##  ##  ##  ##  ##  ##  ##  ##  ##  ##  ##  ##  ##  ##  ##  ##  ##  ##  ##  ##  ##  ##  ##  ##  ##  ##  ##  ##  ##  ##  ##  ##  ##  ##  ##  ##  ##  ##  ##  ##  ##  ##  ##  ##  ##  ##  ##  ##  ##  ##  ##  ##  ##  ##  ##  ##  ##  ##  ##  ##  ##  ##  ##  ##  ##  ##  ##  ##  ##  ##  ## [2447_7]104	: ##  ##  ##  ##  ##  ##  ##  ##  ##  ##  ##  ##  ##  ##  ##  ##  ##  ##  ##  ##  ##  ##  ##  ##  ##  ##  ##  ##  ##  ##  ##  ##  ##  ##  ##  ##  ##  ##  ##  ##  ##  ##  ##  ##  ##  ##  ##  ##  ##  ##  ##  ##  ##  ##  ##  ##  ##  ##  ##  ##  ##  ##  ##  ##  ##  ##  ##  ##  ##  ##  ##  ##  ##  ##  ##  ##  ##  ##  ##  ##  ##  ##  ##  ##  ##  ##  ##  ##  ##  ##  ##  ##  ##  ##  ##  ##  ##  ##  ##  ##  ##  1  2  --  2  3  3  2  3  ##  ##  ##  ##  ##  ##  ##  ##  ##  ##  ##  ##  ##  ##  ##  ##  ##  ##  ##  ##  ##  ##  ##  ##  ##  ##  ##  ##  ##  ##  ##  ##  ##  ##  ##  ##  ##  ##  ##  ##  ##  ##  ##  ##  ##  ##  ##  ##  ##  ##  ##  ##  ##  ##  ##  ##  ##  ##  ##  ##  ##  ##  ##  ##  ##  ##  ##  ##  ##  ##  ##  ##  ##  ##  ##  ##  ##  ##  ##  ##  ##  ##  ##  ##  ##  ##  ##  ##  ##  ##  ##  ##  ##  ##  ##  ##  ##  ##  ##  ##  ##  ##  ##  ##  ##  ##  ##  ##  ##  ##  ##  ##  ##  ##  ##  ##  ##  ##  ##  ##  ##  ##  ##  ##  ##  ##  ##  ##  ##  ##  ##  ##  ##  ##  ##  ##  ##  ##  ##  ##  ##  ##  ##  ##  ##  ##  ##  ##  ##  ##  ##  ##  ##  ##  ##  ##  ##  ##  ##  ##  ## [2448_7]105	: ##  ##  ##  ##  ##  ##  ##  ##  ##  ##  ##  ##  ##  ##  ##  ##  ##  ##  ##  ##  ##  ##  ##  ##  ##  ##  ##  ##  ##  ##  ##  ##  ##  ##  ##  ##  ##  ##  ##  ##  ##  ##  ##  ##  ##  ##  ##  ##  ##  ##  ##  ##  ##  ##  ##  ##  ##  ##  ##  ##  ##  ##  ##  ##  ##  ##  ##  ##  ##  ##  ##  ##  ##  ##  ##  ##  ##  ##  ##  ##  ##  ##  ##  ##  ##  ##  ##  ##  ##  ##  ##  ##  ##  ##  ##  ##  ##  ##  ##  ##  ##  1  2  2  --  3  3  2  3  ##  ##  ##  ##  ##  ##  ##  ##  ##  ##  ##  ##  ##  ##  ##  ##  ##  ##  ##  ##  ##  ##  ##  ##  ##  ##  ##  ##  ##  ##  ##  ##  ##  ##  ##  ##  ##  ##  ##  ##  ##  ##  ##  ##  ##  ##  ##  ##  ##  ##  ##  ##  ##  ##  ##  ##  ##  ##  ##  ##  ##  ##  ##  ##  ##  ##  ##  ##  ##  ##  ##  ##  ##  ##  ##  ##  ##  ##  ##  ##  ##  ##  ##  ##  ##  ##  ##  ##  ##  ##  ##  ##  ##  ##  ##  ##  ##  ##  ##  ##  ##  ##  ##  ##  ##  ##  ##  ##  ##  ##  ##  ##  ##  ##  ##  ##  ##  ##  ##  ##  ##  ##  ##  ##  ##  ##  ##  ##  ##  ##  ##  ##  ##  ##  ##  ##  ##  ##  ##  ##  ##  ##  ##  ##  ##  ##  ##  ##  ##  ##  ##  ##  ##  ##  ##  ##  ##  ##  ##  ##  ## [2449_7]106	: ##  ##  ##  ##  ##  ##  ##  ##  ##  ##  ##  ##  ##  ##  ##  ##  ##  ##  ##  ##  ##  ##  ##  ##  ##  ##  ##  ##  ##  ##  ##  ##  ##  ##  ##  ##  ##  ##  ##  ##  ##  ##  ##  ##  ##  ##  ##  ##  ##  ##  ##  ##  ##  ##  ##  ##  ##  ##  ##  ##  ##  ##  ##  ##  ##  ##  ##  ##  ##  ##  ##  ##  ##  ##  ##  ##  ##  ##  ##  ##  ##  ##  ##  ##  ##  ##  ##  ##  ##  ##  ##  ##  ##  ##  ##  ##  ##  ##  ##  ##  ##  2  3  3  3  --  4  3  2  ##  ##  ##  ##  ##  ##  ##  ##  ##  ##  ##  ##  ##  ##  ##  ##  ##  ##  ##  ##  ##  ##  ##  ##  ##  ##  ##  ##  ##  ##  ##  ##  ##  ##  ##  ##  ##  ##  ##  ##  ##  ##  ##  ##  ##  ##  ##  ##  ##  ##  ##  ##  ##  ##  ##  ##  ##  ##  ##  ##  ##  ##  ##  ##  ##  ##  ##  ##  ##  ##  ##  ##  ##  ##  ##  ##  ##  ##  ##  ##  ##  ##  ##  ##  ##  ##  ##  ##  ##  ##  ##  ##  ##  ##  ##  ##  ##  ##  ##  ##  ##  ##  ##  ##  ##  ##  ##  ##  ##  ##  ##  ##  ##  ##  ##  ##  ##  ##  ##  ##  ##  ##  ##  ##  ##  ##  ##  ##  ##  ##  ##  ##  ##  ##  ##  ##  ##  ##  ##  ##  ##  ##  ##  ##  ##  ##  ##  ##  ##  ##  ##  ##  ##  ##  ##  ##  ##  ##  ##  ##  ## [2450_7]107	: ##  ##  ##  ##  ##  ##  ##  ##  ##  ##  ##  ##  ##  ##  ##  ##  ##  ##  ##  ##  ##  ##  ##  ##  ##  ##  ##  ##  ##  ##  ##  ##  ##  ##  ##  ##  ##  ##  ##  ##  ##  ##  ##  ##  ##  ##  ##  ##  ##  ##  ##  ##  ##  ##  ##  ##  ##  ##  ##  ##  ##  ##  ##  ##  ##  ##  ##  ##  ##  ##  ##  ##  ##  ##  ##  ##  ##  ##  ##  ##  ##  ##  ##  ##  ##  ##  ##  ##  ##  ##  ##  ##  ##  ##  ##  ##  ##  ##  ##  ##  ##  2  3  3  3  4  --  3  4  ##  ##  ##  ##  ##  ##  ##  ##  ##  ##  ##  ##  ##  ##  ##  ##  ##  ##  ##  ##  ##  ##  ##  ##  ##  ##  ##  ##  ##  ##  ##  ##  ##  ##  ##  ##  ##  ##  ##  ##  ##  ##  ##  ##  ##  ##  ##  ##  ##  ##  ##  ##  ##  ##  ##  ##  ##  ##  ##  ##  ##  ##  ##  ##  ##  ##  ##  ##  ##  ##  ##  ##  ##  ##  ##  ##  ##  ##  ##  ##  ##  ##  ##  ##  ##  ##  ##  ##  ##  ##  ##  ##  ##  ##  ##  ##  ##  ##  ##  ##  ##  ##  ##  ##  ##  ##  ##  ##  ##  ##  ##  ##  ##  ##  ##  ##  ##  ##  ##  ##  ##  ##  ##  ##  ##  ##  ##  ##  ##  ##  ##  ##  ##  ##  ##  ##  ##  ##  ##  ##  ##  ##  ##  ##  ##  ##  ##  ##  ##  ##  ##  ##  ##  ##  ##  ##  ##  ##  ##  ##  ## [2452_7]108	: ##  ##  ##  ##  ##  ##  ##  ##  ##  ##  ##  ##  ##  ##  ##  ##  ##  ##  ##  ##  ##  ##  ##  ##  ##  ##  ##  ##  ##  ##  ##  ##  ##  ##  ##  ##  ##  ##  ##  ##  ##  ##  ##  ##  ##  ##  ##  ##  ##  ##  ##  ##  ##  ##  ##  ##  ##  ##  ##  ##  ##  ##  ##  ##  ##  ##  ##  ##  ##  ##  ##  ##  ##  ##  ##  ##  ##  ##  ##  ##  ##  ##  ##  ##  ##  ##  ##  ##  ##  ##  ##  ##  ##  ##  ##  ##  ##  ##  ##  ##  ##  1  2  2  2  3  3  --  3  ##  ##  ##  ##  ##  ##  ##  ##  ##  ##  ##  ##  ##  ##  ##  ##  ##  ##  ##  ##  ##  ##  ##  ##  ##  ##  ##  ##  ##  ##  ##  ##  ##  ##  ##  ##  ##  ##  ##  ##  ##  ##  ##  ##  ##  ##  ##  ##  ##  ##  ##  ##  ##  ##  ##  ##  ##  ##  ##  ##  ##  ##  ##  ##  ##  ##  ##  ##  ##  ##  ##  ##  ##  ##  ##  ##  ##  ##  ##  ##  ##  ##  ##  ##  ##  ##  ##  ##  ##  ##  ##  ##  ##  ##  ##  ##  ##  ##  ##  ##  ##  ##  ##  ##  ##  ##  ##  ##  ##  ##  ##  ##  ##  ##  ##  ##  ##  ##  ##  ##  ##  ##  ##  ##  ##  ##  ##  ##  ##  ##  ##  ##  ##  ##  ##  ##  ##  ##  ##  ##  ##  ##  ##  ##  ##  ##  ##  ##  ##  ##  ##  ##  ##  ##  ##  ##  ##  ##  ##  ##  ## [2914_7]109	: ##  ##  ##  ##  ##  ##  ##  ##  ##  ##  ##  ##  ##  ##  ##  ##  ##  ##  ##  ##  ##  ##  ##  ##  ##  ##  ##  ##  ##  ##  ##  ##  ##  ##  ##  ##  ##  ##  ##  ##  ##  ##  ##  ##  ##  ##  ##  ##  ##  ##  ##  ##  ##  ##  ##  ##  ##  ##  ##  ##  ##  ##  ##  ##  ##  ##  ##  ##  ##  ##  ##  ##  ##  ##  ##  ##  ##  ##  ##  ##  ##  ##  ##  ##  ##  ##  ##  ##  ##  ##  ##  ##  ##  ##  ##  ##  ##  ##  ##  ##  ##  2  3  3  3  2  4  3  --  ##  ##  ##  ##  ##  ##  ##  ##  ##  ##  ##  ##  ##  ##  ##  ##  ##  ##  ##  ##  ##  ##  ##  ##  ##  ##  ##  ##  ##  ##  ##  ##  ##  ##  ##  ##  ##  ##  ##  ##  ##  ##  ##  ##  ##  ##  ##  ##  ##  ##  ##  ##  ##  ##  ##  ##  ##  ##  ##  ##  ##  ##  ##  ##  ##  ##  ##  ##  ##  ##  ##  ##  ##  ##  ##  ##  ##  ##  ##  ##  ##  ##  ##  ##  ##  ##  ##  ##  ##  ##  ##  ##  ##  ##  ##  ##  ##  ##  ##  ##  ##  ##  ##  ##  ##  ##  ##  ##  ##  ##  ##  ##  ##  ##  ##  ##  ##  ##  ##  ##  ##  ##  ##  ##  ##  ##  ##  ##  ##  ##  ##  ##  ##  ##  ##  ##  ##  ##  ##  ##  ##  ##  ##  ##  ##  ##  ##  ##  ##  ##  ##  ##  ##  ##  ##  ##  ##  ##  ##  ##  ## [1197_8]110	: ##  ##  ##  ##  ##  ##  ##  ##  ##  ##  ##  ##  ##  ##  ##  ##  ##  ##  ##  ##  ##  ##  ##  ##  ##  ##  ##  ##  ##  ##  ##  ##  ##  ##  ##  ##  ##  ##  ##  ##  ##  ##  ##  ##  ##  ##  ##  ##  ##  ##  ##  ##  ##  ##  ##  ##  ##  ##  ##  ##  ##  ##  ##  ##  ##  ##  ##  ##  ##  ##  ##  ##  ##  ##  ##  ##  ##  ##  ##  ##  ##  ##  ##  ##  ##  ##  ##  ##  ##  ##  ##  ##  ##  ##  ##  ##  ##  ##  ##  ##  ##  ##  ##  ##  ##  ##  ##  ##  ##  --  4  11  12  11  10  11  9  11  11  10  10  9  10  11  7  11  13  10  4  3  9  8  13  3  12  13  10  13  10  9  11  12  12  ##  ##  ##  ##  ##  ##  ##  ##  ##  ##  ##  ##  ##  ##  ##  ##  ##  ##  ##  ##  ##  ##  ##  ##  ##  ##  ##  ##  ##  ##  ##  ##  ##  ##  ##  ##  ##  ##  ##  ##  ##  ##  ##  ##  ##  ##  ##  ##  ##  ##  ##  ##  ##  ##  ##  ##  ##  ##  ##  ##  ##  ##  ##  ##  ##  ##  ##  ##  ##  ##  ##  ##  ##  ##  ##  ##  ##  ##  ##  ##  ##  ##  ##  ##  ##  ##  ##  ##  ##  ##  ##  ##  ##  ##  ##  ##  ##  ##  ##  ##  ##  ##  ##  ##  ##  ##  ##  ##  ##  ##  ##  ##  ##  ##  ##  ##  ##  ##  ##  ##  ##  ##  ##  ##  ##  ##  ## [1198_8]111	: ##  ##  ##  ##  ##  ##  ##  ##  ##  ##  ##  ##  ##  ##  ##  ##  ##  ##  ##  ##  ##  ##  ##  ##  ##  ##  ##  ##  ##  ##  ##  ##  ##  ##  ##  ##  ##  ##  ##  ##  ##  ##  ##  ##  ##  ##  ##  ##  ##  ##  ##  ##  ##  ##  ##  ##  ##  ##  ##  ##  ##  ##  ##  ##  ##  ##  ##  ##  ##  ##  ##  ##  ##  ##  ##  ##  ##  ##  ##  ##  ##  ##  ##  ##  ##  ##  ##  ##  ##  ##  ##  ##  ##  ##  ##  ##  ##  ##  ##  ##  ##  ##  ##  ##  ##  ##  ##  ##  ##  4  --  13  14  13  12  15  13  13  13  12  10  11  12  13  9  11  13  12  6  5  13  12  15  7  14  17  12  17  12  13  13  14  14  ##  ##  ##  ##  ##  ##  ##  ##  ##  ##  ##  ##  ##  ##  ##  ##  ##  ##  ##  ##  ##  ##  ##  ##  ##  ##  ##  ##  ##  ##  ##  ##  ##  ##  ##  ##  ##  ##  ##  ##  ##  ##  ##  ##  ##  ##  ##  ##  ##  ##  ##  ##  ##  ##  ##  ##  ##  ##  ##  ##  ##  ##  ##  ##  ##  ##  ##  ##  ##  ##  ##  ##  ##  ##  ##  ##  ##  ##  ##  ##  ##  ##  ##  ##  ##  ##  ##  ##  ##  ##  ##  ##  ##  ##  ##  ##  ##  ##  ##  ##  ##  ##  ##  ##  ##  ##  ##  ##  ##  ##  ##  ##  ##  ##  ##  ##  ##  ##  ##  ##  ##  ##  ##  ##  ##  ##  ## [1199_8]112	: ##  ##  ##  ##  ##  ##  ##  ##  ##  ##  ##  ##  ##  ##  ##  ##  ##  ##  ##  ##  ##  ##  ##  ##  ##  ##  ##  ##  ##  ##  ##  ##  ##  ##  ##  ##  ##  ##  ##  ##  ##  ##  ##  ##  ##  ##  ##  ##  ##  ##  ##  ##  ##  ##  ##  ##  ##  ##  ##  ##  ##  ##  ##  ##  ##  ##  ##  ##  ##  ##  ##  ##  ##  ##  ##  ##  ##  ##  ##  ##  ##  ##  ##  ##  ##  ##  ##  ##  ##  ##  ##  ##  ##  ##  ##  ##  ##  ##  ##  ##  ##  ##  ##  ##  ##  ##  ##  ##  ##  11  13  --  6  7  4  9  7  7  6  4  4  3  4  7  14  5  7  4  13  12  7  6  7  14  8  11  8  11  4  7  5  8  8  ##  ##  ##  ##  ##  ##  ##  ##  ##  ##  ##  ##  ##  ##  ##  ##  ##  ##  ##  ##  ##  ##  ##  ##  ##  ##  ##  ##  ##  ##  ##  ##  ##  ##  ##  ##  ##  ##  ##  ##  ##  ##  ##  ##  ##  ##  ##  ##  ##  ##  ##  ##  ##  ##  ##  ##  ##  ##  ##  ##  ##  ##  ##  ##  ##  ##  ##  ##  ##  ##  ##  ##  ##  ##  ##  ##  ##  ##  ##  ##  ##  ##  ##  ##  ##  ##  ##  ##  ##  ##  ##  ##  ##  ##  ##  ##  ##  ##  ##  ##  ##  ##  ##  ##  ##  ##  ##  ##  ##  ##  ##  ##  ##  ##  ##  ##  ##  ##  ##  ##  ##  ##  ##  ##  ##  ##  ## [1200_8]113	: ##  ##  ##  ##  ##  ##  ##  ##  ##  ##  ##  ##  ##  ##  ##  ##  ##  ##  ##  ##  ##  ##  ##  ##  ##  ##  ##  ##  ##  ##  ##  ##  ##  ##  ##  ##  ##  ##  ##  ##  ##  ##  ##  ##  ##  ##  ##  ##  ##  ##  ##  ##  ##  ##  ##  ##  ##  ##  ##  ##  ##  ##  ##  ##  ##  ##  ##  ##  ##  ##  ##  ##  ##  ##  ##  ##  ##  ##  ##  ##  ##  ##  ##  ##  ##  ##  ##  ##  ##  ##  ##  ##  ##  ##  ##  ##  ##  ##  ##  ##  ##  ##  ##  ##  ##  ##  ##  ##  ##  12  14  6  --  7  4  9  7  7  8  4  4  3  4  4  14  3  5  2  14  13  7  6  5  15  8  11  8  11  4  7  5  8  8  ##  ##  ##  ##  ##  ##  ##  ##  ##  ##  ##  ##  ##  ##  ##  ##  ##  ##  ##  ##  ##  ##  ##  ##  ##  ##  ##  ##  ##  ##  ##  ##  ##  ##  ##  ##  ##  ##  ##  ##  ##  ##  ##  ##  ##  ##  ##  ##  ##  ##  ##  ##  ##  ##  ##  ##  ##  ##  ##  ##  ##  ##  ##  ##  ##  ##  ##  ##  ##  ##  ##  ##  ##  ##  ##  ##  ##  ##  ##  ##  ##  ##  ##  ##  ##  ##  ##  ##  ##  ##  ##  ##  ##  ##  ##  ##  ##  ##  ##  ##  ##  ##  ##  ##  ##  ##  ##  ##  ##  ##  ##  ##  ##  ##  ##  ##  ##  ##  ##  ##  ##  ##  ##  ##  ##  ##  ## [1202_8]114	: ##  ##  ##  ##  ##  ##  ##  ##  ##  ##  ##  ##  ##  ##  ##  ##  ##  ##  ##  ##  ##  ##  ##  ##  ##  ##  ##  ##  ##  ##  ##  ##  ##  ##  ##  ##  ##  ##  ##  ##  ##  ##  ##  ##  ##  ##  ##  ##  ##  ##  ##  ##  ##  ##  ##  ##  ##  ##  ##  ##  ##  ##  ##  ##  ##  ##  ##  ##  ##  ##  ##  ##  ##  ##  ##  ##  ##  ##  ##  ##  ##  ##  ##  ##  ##  ##  ##  ##  ##  ##  ##  ##  ##  ##  ##  ##  ##  ##  ##  ##  ##  ##  ##  ##  ##  ##  ##  ##  ##  11  13  7  7  --  5  6  4  4  5  5  5  4  3  8  15  6  6  5  13  12  4  3  8  10  5  8  5  8  5  4  6  5  3  ##  ##  ##  ##  ##  ##  ##  ##  ##  ##  ##  ##  ##  ##  ##  ##  ##  ##  ##  ##  ##  ##  ##  ##  ##  ##  ##  ##  ##  ##  ##  ##  ##  ##  ##  ##  ##  ##  ##  ##  ##  ##  ##  ##  ##  ##  ##  ##  ##  ##  ##  ##  ##  ##  ##  ##  ##  ##  ##  ##  ##  ##  ##  ##  ##  ##  ##  ##  ##  ##  ##  ##  ##  ##  ##  ##  ##  ##  ##  ##  ##  ##  ##  ##  ##  ##  ##  ##  ##  ##  ##  ##  ##  ##  ##  ##  ##  ##  ##  ##  ##  ##  ##  ##  ##  ##  ##  ##  ##  ##  ##  ##  ##  ##  ##  ##  ##  ##  ##  ##  ##  ##  ##  ##  ##  ##  ## [1203_8]115	: ##  ##  ##  ##  ##  ##  ##  ##  ##  ##  ##  ##  ##  ##  ##  ##  ##  ##  ##  ##  ##  ##  ##  ##  ##  ##  ##  ##  ##  ##  ##  ##  ##  ##  ##  ##  ##  ##  ##  ##  ##  ##  ##  ##  ##  ##  ##  ##  ##  ##  ##  ##  ##  ##  ##  ##  ##  ##  ##  ##  ##  ##  ##  ##  ##  ##  ##  ##  ##  ##  ##  ##  ##  ##  ##  ##  ##  ##  ##  ##  ##  ##  ##  ##  ##  ##  ##  ##  ##  ##  ##  ##  ##  ##  ##  ##  ##  ##  ##  ##  ##  ##  ##  ##  ##  ##  ##  ##  ##  10  12  4  4  5  --  6  5  5  6  1  2  1  2  5  13  3  5  2  11  10  5  4  5  13  6  8  6  9  2  5  3  6  6  ##  ##  ##  ##  ##  ##  ##  ##  ##  ##  ##  ##  ##  ##  ##  ##  ##  ##  ##  ##  ##  ##  ##  ##  ##  ##  ##  ##  ##  ##  ##  ##  ##  ##  ##  ##  ##  ##  ##  ##  ##  ##  ##  ##  ##  ##  ##  ##  ##  ##  ##  ##  ##  ##  ##  ##  ##  ##  ##  ##  ##  ##  ##  ##  ##  ##  ##  ##  ##  ##  ##  ##  ##  ##  ##  ##  ##  ##  ##  ##  ##  ##  ##  ##  ##  ##  ##  ##  ##  ##  ##  ##  ##  ##  ##  ##  ##  ##  ##  ##  ##  ##  ##  ##  ##  ##  ##  ##  ##  ##  ##  ##  ##  ##  ##  ##  ##  ##  ##  ##  ##  ##  ##  ##  ##  ##  ## [1561_8]116	: ##  ##  ##  ##  ##  ##  ##  ##  ##  ##  ##  ##  ##  ##  ##  ##  ##  ##  ##  ##  ##  ##  ##  ##  ##  ##  ##  ##  ##  ##  ##  ##  ##  ##  ##  ##  ##  ##  ##  ##  ##  ##  ##  ##  ##  ##  ##  ##  ##  ##  ##  ##  ##  ##  ##  ##  ##  ##  ##  ##  ##  ##  ##  ##  ##  ##  ##  ##  ##  ##  ##  ##  ##  ##  ##  ##  ##  ##  ##  ##  ##  ##  ##  ##  ##  ##  ##  ##  ##  ##  ##  ##  ##  ##  ##  ##  ##  ##  ##  ##  ##  ##  ##  ##  ##  ##  ##  ##  ##  11  15  9  9  6  6  --  4  6  7  7  7  6  5  10  17  8  10  7  15  14  4  3  10  12  7  8  5  8  7  4  8  7  7  ##  ##  ##  ##  ##  ##  ##  ##  ##  ##  ##  ##  ##  ##  ##  ##  ##  ##  ##  ##  ##  ##  ##  ##  ##  ##  ##  ##  ##  ##  ##  ##  ##  ##  ##  ##  ##  ##  ##  ##  ##  ##  ##  ##  ##  ##  ##  ##  ##  ##  ##  ##  ##  ##  ##  ##  ##  ##  ##  ##  ##  ##  ##  ##  ##  ##  ##  ##  ##  ##  ##  ##  ##  ##  ##  ##  ##  ##  ##  ##  ##  ##  ##  ##  ##  ##  ##  ##  ##  ##  ##  ##  ##  ##  ##  ##  ##  ##  ##  ##  ##  ##  ##  ##  ##  ##  ##  ##  ##  ##  ##  ##  ##  ##  ##  ##  ##  ##  ##  ##  ##  ##  ##  ##  ##  ##  ## [1922_8]117	: ##  ##  ##  ##  ##  ##  ##  ##  ##  ##  ##  ##  ##  ##  ##  ##  ##  ##  ##  ##  ##  ##  ##  ##  ##  ##  ##  ##  ##  ##  ##  ##  ##  ##  ##  ##  ##  ##  ##  ##  ##  ##  ##  ##  ##  ##  ##  ##  ##  ##  ##  ##  ##  ##  ##  ##  ##  ##  ##  ##  ##  ##  ##  ##  ##  ##  ##  ##  ##  ##  ##  ##  ##  ##  ##  ##  ##  ##  ##  ##  ##  ##  ##  ##  ##  ##  ##  ##  ##  ##  ##  ##  ##  ##  ##  ##  ##  ##  ##  ##  ##  ##  ##  ##  ##  ##  ##  ##  ##  9  13  7  7  4  5  4  --  4  5  5  6  4  3  8  15  6  8  5  13  12  2  1  8  10  5  6  3  4  5  2  6  5  5  ##  ##  ##  ##  ##  ##  ##  ##  ##  ##  ##  ##  ##  ##  ##  ##  ##  ##  ##  ##  ##  ##  ##  ##  ##  ##  ##  ##  ##  ##  ##  ##  ##  ##  ##  ##  ##  ##  ##  ##  ##  ##  ##  ##  ##  ##  ##  ##  ##  ##  ##  ##  ##  ##  ##  ##  ##  ##  ##  ##  ##  ##  ##  ##  ##  ##  ##  ##  ##  ##  ##  ##  ##  ##  ##  ##  ##  ##  ##  ##  ##  ##  ##  ##  ##  ##  ##  ##  ##  ##  ##  ##  ##  ##  ##  ##  ##  ##  ##  ##  ##  ##  ##  ##  ##  ##  ##  ##  ##  ##  ##  ##  ##  ##  ##  ##  ##  ##  ##  ##  ##  ##  ##  ##  ##  ##  ## [1946_8]118	: ##  ##  ##  ##  ##  ##  ##  ##  ##  ##  ##  ##  ##  ##  ##  ##  ##  ##  ##  ##  ##  ##  ##  ##  ##  ##  ##  ##  ##  ##  ##  ##  ##  ##  ##  ##  ##  ##  ##  ##  ##  ##  ##  ##  ##  ##  ##  ##  ##  ##  ##  ##  ##  ##  ##  ##  ##  ##  ##  ##  ##  ##  ##  ##  ##  ##  ##  ##  ##  ##  ##  ##  ##  ##  ##  ##  ##  ##  ##  ##  ##  ##  ##  ##  ##  ##  ##  ##  ##  ##  ##  ##  ##  ##  ##  ##  ##  ##  ##  ##  ##  ##  ##  ##  ##  ##  ##  ##  ##  11  13  7  7  4  5  6  4  --  5  5  6  4  3  8  15  6  8  5  13  12  4  3  8  12  1  8  5  8  5  4  6  1  1  ##  ##  ##  ##  ##  ##  ##  ##  ##  ##  ##  ##  ##  ##  ##  ##  ##  ##  ##  ##  ##  ##  ##  ##  ##  ##  ##  ##  ##  ##  ##  ##  ##  ##  ##  ##  ##  ##  ##  ##  ##  ##  ##  ##  ##  ##  ##  ##  ##  ##  ##  ##  ##  ##  ##  ##  ##  ##  ##  ##  ##  ##  ##  ##  ##  ##  ##  ##  ##  ##  ##  ##  ##  ##  ##  ##  ##  ##  ##  ##  ##  ##  ##  ##  ##  ##  ##  ##  ##  ##  ##  ##  ##  ##  ##  ##  ##  ##  ##  ##  ##  ##  ##  ##  ##  ##  ##  ##  ##  ##  ##  ##  ##  ##  ##  ##  ##  ##  ##  ##  ##  ##  ##  ##  ##  ##  ## [1957_8]119	: ##  ##  ##  ##  ##  ##  ##  ##  ##  ##  ##  ##  ##  ##  ##  ##  ##  ##  ##  ##  ##  ##  ##  ##  ##  ##  ##  ##  ##  ##  ##  ##  ##  ##  ##  ##  ##  ##  ##  ##  ##  ##  ##  ##  ##  ##  ##  ##  ##  ##  ##  ##  ##  ##  ##  ##  ##  ##  ##  ##  ##  ##  ##  ##  ##  ##  ##  ##  ##  ##  ##  ##  ##  ##  ##  ##  ##  ##  ##  ##  ##  ##  ##  ##  ##  ##  ##  ##  ##  ##  ##  ##  ##  ##  ##  ##  ##  ##  ##  ##  ##  ##  ##  ##  ##  ##  ##  ##  ##  11  13  6  8  5  6  7  5  5  --  6  7  5  4  9  14  7  9  6  13  12  5  4  9  12  6  9  6  9  6  5  7  6  6  ##  ##  ##  ##  ##  ##  ##  ##  ##  ##  ##  ##  ##  ##  ##  ##  ##  ##  ##  ##  ##  ##  ##  ##  ##  ##  ##  ##  ##  ##  ##  ##  ##  ##  ##  ##  ##  ##  ##  ##  ##  ##  ##  ##  ##  ##  ##  ##  ##  ##  ##  ##  ##  ##  ##  ##  ##  ##  ##  ##  ##  ##  ##  ##  ##  ##  ##  ##  ##  ##  ##  ##  ##  ##  ##  ##  ##  ##  ##  ##  ##  ##  ##  ##  ##  ##  ##  ##  ##  ##  ##  ##  ##  ##  ##  ##  ##  ##  ##  ##  ##  ##  ##  ##  ##  ##  ##  ##  ##  ##  ##  ##  ##  ##  ##  ##  ##  ##  ##  ##  ##  ##  ##  ##  ##  ##  ## [1958_8]120	: ##  ##  ##  ##  ##  ##  ##  ##  ##  ##  ##  ##  ##  ##  ##  ##  ##  ##  ##  ##  ##  ##  ##  ##  ##  ##  ##  ##  ##  ##  ##  ##  ##  ##  ##  ##  ##  ##  ##  ##  ##  ##  ##  ##  ##  ##  ##  ##  ##  ##  ##  ##  ##  ##  ##  ##  ##  ##  ##  ##  ##  ##  ##  ##  ##  ##  ##  ##  ##  ##  ##  ##  ##  ##  ##  ##  ##  ##  ##  ##  ##  ##  ##  ##  ##  ##  ##  ##  ##  ##  ##  ##  ##  ##  ##  ##  ##  ##  ##  ##  ##  ##  ##  ##  ##  ##  ##  ##  ##  10  12  4  4  5  1  7  5  5  6  --  3  1  2  5  12  3  5  2  12  11  5  4  5  13  6  9  6  9  2  5  3  6  6  ##  ##  ##  ##  ##  ##  ##  ##  ##  ##  ##  ##  ##  ##  ##  ##  ##  ##  ##  ##  ##  ##  ##  ##  ##  ##  ##  ##  ##  ##  ##  ##  ##  ##  ##  ##  ##  ##  ##  ##  ##  ##  ##  ##  ##  ##  ##  ##  ##  ##  ##  ##  ##  ##  ##  ##  ##  ##  ##  ##  ##  ##  ##  ##  ##  ##  ##  ##  ##  ##  ##  ##  ##  ##  ##  ##  ##  ##  ##  ##  ##  ##  ##  ##  ##  ##  ##  ##  ##  ##  ##  ##  ##  ##  ##  ##  ##  ##  ##  ##  ##  ##  ##  ##  ##  ##  ##  ##  ##  ##  ##  ##  ##  ##  ##  ##  ##  ##  ##  ##  ##  ##  ##  ##  ##  ##  ## [1984_8]121	: ##  ##  ##  ##  ##  ##  ##  ##  ##  ##  ##  ##  ##  ##  ##  ##  ##  ##  ##  ##  ##  ##  ##  ##  ##  ##  ##  ##  ##  ##  ##  ##  ##  ##  ##  ##  ##  ##  ##  ##  ##  ##  ##  ##  ##  ##  ##  ##  ##  ##  ##  ##  ##  ##  ##  ##  ##  ##  ##  ##  ##  ##  ##  ##  ##  ##  ##  ##  ##  ##  ##  ##  ##  ##  ##  ##  ##  ##  ##  ##  ##  ##  ##  ##  ##  ##  ##  ##  ##  ##  ##  ##  ##  ##  ##  ##  ##  ##  ##  ##  ##  ##  ##  ##  ##  ##  ##  ##  ##  10  10  4  4  5  2  7  6  6  7  3  --  2  3  6  15  4  6  3  13  12  6  5  6  14  6  9  6  9  2  5  3  6  6  ##  ##  ##  ##  ##  ##  ##  ##  ##  ##  ##  ##  ##  ##  ##  ##  ##  ##  ##  ##  ##  ##  ##  ##  ##  ##  ##  ##  ##  ##  ##  ##  ##  ##  ##  ##  ##  ##  ##  ##  ##  ##  ##  ##  ##  ##  ##  ##  ##  ##  ##  ##  ##  ##  ##  ##  ##  ##  ##  ##  ##  ##  ##  ##  ##  ##  ##  ##  ##  ##  ##  ##  ##  ##  ##  ##  ##  ##  ##  ##  ##  ##  ##  ##  ##  ##  ##  ##  ##  ##  ##  ##  ##  ##  ##  ##  ##  ##  ##  ##  ##  ##  ##  ##  ##  ##  ##  ##  ##  ##  ##  ##  ##  ##  ##  ##  ##  ##  ##  ##  ##  ##  ##  ##  ##  ##  ## [1985_8]122	: ##  ##  ##  ##  ##  ##  ##  ##  ##  ##  ##  ##  ##  ##  ##  ##  ##  ##  ##  ##  ##  ##  ##  ##  ##  ##  ##  ##  ##  ##  ##  ##  ##  ##  ##  ##  ##  ##  ##  ##  ##  ##  ##  ##  ##  ##  ##  ##  ##  ##  ##  ##  ##  ##  ##  ##  ##  ##  ##  ##  ##  ##  ##  ##  ##  ##  ##  ##  ##  ##  ##  ##  ##  ##  ##  ##  ##  ##  ##  ##  ##  ##  ##  ##  ##  ##  ##  ##  ##  ##  ##  ##  ##  ##  ##  ##  ##  ##  ##  ##  ##  ##  ##  ##  ##  ##  ##  ##  ##  9  11  3  3  4  1  6  4  4  5  1  2  --  1  4  13  2  4  1  11  10  4  3  4  12  5  8  5  8  1  4  2  5  5  ##  ##  ##  ##  ##  ##  ##  ##  ##  ##  ##  ##  ##  ##  ##  ##  ##  ##  ##  ##  ##  ##  ##  ##  ##  ##  ##  ##  ##  ##  ##  ##  ##  ##  ##  ##  ##  ##  ##  ##  ##  ##  ##  ##  ##  ##  ##  ##  ##  ##  ##  ##  ##  ##  ##  ##  ##  ##  ##  ##  ##  ##  ##  ##  ##  ##  ##  ##  ##  ##  ##  ##  ##  ##  ##  ##  ##  ##  ##  ##  ##  ##  ##  ##  ##  ##  ##  ##  ##  ##  ##  ##  ##  ##  ##  ##  ##  ##  ##  ##  ##  ##  ##  ##  ##  ##  ##  ##  ##  ##  ##  ##  ##  ##  ##  ##  ##  ##  ##  ##  ##  ##  ##  ##  ##  ##  ## [1988_8]123	: ##  ##  ##  ##  ##  ##  ##  ##  ##  ##  ##  ##  ##  ##  ##  ##  ##  ##  ##  ##  ##  ##  ##  ##  ##  ##  ##  ##  ##  ##  ##  ##  ##  ##  ##  ##  ##  ##  ##  ##  ##  ##  ##  ##  ##  ##  ##  ##  ##  ##  ##  ##  ##  ##  ##  ##  ##  ##  ##  ##  ##  ##  ##  ##  ##  ##  ##  ##  ##  ##  ##  ##  ##  ##  ##  ##  ##  ##  ##  ##  ##  ##  ##  ##  ##  ##  ##  ##  ##  ##  ##  ##  ##  ##  ##  ##  ##  ##  ##  ##  ##  ##  ##  ##  ##  ##  ##  ##  ##  10  12  4  4  3  2  5  3  3  4  2  3  1  --  5  14  3  5  2  12  11  3  2  5  11  4  7  4  7  2  3  3  4  4  ##  ##  ##  ##  ##  ##  ##  ##  ##  ##  ##  ##  ##  ##  ##  ##  ##  ##  ##  ##  ##  ##  ##  ##  ##  ##  ##  ##  ##  ##  ##  ##  ##  ##  ##  ##  ##  ##  ##  ##  ##  ##  ##  ##  ##  ##  ##  ##  ##  ##  ##  ##  ##  ##  ##  ##  ##  ##  ##  ##  ##  ##  ##  ##  ##  ##  ##  ##  ##  ##  ##  ##  ##  ##  ##  ##  ##  ##  ##  ##  ##  ##  ##  ##  ##  ##  ##  ##  ##  ##  ##  ##  ##  ##  ##  ##  ##  ##  ##  ##  ##  ##  ##  ##  ##  ##  ##  ##  ##  ##  ##  ##  ##  ##  ##  ##  ##  ##  ##  ##  ##  ##  ##  ##  ##  ##  ## [1994_8]124	: ##  ##  ##  ##  ##  ##  ##  ##  ##  ##  ##  ##  ##  ##  ##  ##  ##  ##  ##  ##  ##  ##  ##  ##  ##  ##  ##  ##  ##  ##  ##  ##  ##  ##  ##  ##  ##  ##  ##  ##  ##  ##  ##  ##  ##  ##  ##  ##  ##  ##  ##  ##  ##  ##  ##  ##  ##  ##  ##  ##  ##  ##  ##  ##  ##  ##  ##  ##  ##  ##  ##  ##  ##  ##  ##  ##  ##  ##  ##  ##  ##  ##  ##  ##  ##  ##  ##  ##  ##  ##  ##  ##  ##  ##  ##  ##  ##  ##  ##  ##  ##  ##  ##  ##  ##  ##  ##  ##  ##  11  13  7  4  8  5  10  8  8  9  5  6  4  5  --  14  4  6  3  13  12  8  7  6  14  9  12  9  12  5  8  6  9  9  ##  ##  ##  ##  ##  ##  ##  ##  ##  ##  ##  ##  ##  ##  ##  ##  ##  ##  ##  ##  ##  ##  ##  ##  ##  ##  ##  ##  ##  ##  ##  ##  ##  ##  ##  ##  ##  ##  ##  ##  ##  ##  ##  ##  ##  ##  ##  ##  ##  ##  ##  ##  ##  ##  ##  ##  ##  ##  ##  ##  ##  ##  ##  ##  ##  ##  ##  ##  ##  ##  ##  ##  ##  ##  ##  ##  ##  ##  ##  ##  ##  ##  ##  ##  ##  ##  ##  ##  ##  ##  ##  ##  ##  ##  ##  ##  ##  ##  ##  ##  ##  ##  ##  ##  ##  ##  ##  ##  ##  ##  ##  ##  ##  ##  ##  ##  ##  ##  ##  ##  ##  ##  ##  ##  ##  ##  ## [1995_8]125	: ##  ##  ##  ##  ##  ##  ##  ##  ##  ##  ##  ##  ##  ##  ##  ##  ##  ##  ##  ##  ##  ##  ##  ##  ##  ##  ##  ##  ##  ##  ##  ##  ##  ##  ##  ##  ##  ##  ##  ##  ##  ##  ##  ##  ##  ##  ##  ##  ##  ##  ##  ##  ##  ##  ##  ##  ##  ##  ##  ##  ##  ##  ##  ##  ##  ##  ##  ##  ##  ##  ##  ##  ##  ##  ##  ##  ##  ##  ##  ##  ##  ##  ##  ##  ##  ##  ##  ##  ##  ##  ##  ##  ##  ##  ##  ##  ##  ##  ##  ##  ##  ##  ##  ##  ##  ##  ##  ##  ##  7  9  14  14  15  13  17  15  15  14  12  15  13  14  14  --  15  15  14  9  8  15  14  16  8  14  19  16  16  14  15  13  16  16  ##  ##  ##  ##  ##  ##  ##  ##  ##  ##  ##  ##  ##  ##  ##  ##  ##  ##  ##  ##  ##  ##  ##  ##  ##  ##  ##  ##  ##  ##  ##  ##  ##  ##  ##  ##  ##  ##  ##  ##  ##  ##  ##  ##  ##  ##  ##  ##  ##  ##  ##  ##  ##  ##  ##  ##  ##  ##  ##  ##  ##  ##  ##  ##  ##  ##  ##  ##  ##  ##  ##  ##  ##  ##  ##  ##  ##  ##  ##  ##  ##  ##  ##  ##  ##  ##  ##  ##  ##  ##  ##  ##  ##  ##  ##  ##  ##  ##  ##  ##  ##  ##  ##  ##  ##  ##  ##  ##  ##  ##  ##  ##  ##  ##  ##  ##  ##  ##  ##  ##  ##  ##  ##  ##  ##  ##  ## [1996_8]126	: ##  ##  ##  ##  ##  ##  ##  ##  ##  ##  ##  ##  ##  ##  ##  ##  ##  ##  ##  ##  ##  ##  ##  ##  ##  ##  ##  ##  ##  ##  ##  ##  ##  ##  ##  ##  ##  ##  ##  ##  ##  ##  ##  ##  ##  ##  ##  ##  ##  ##  ##  ##  ##  ##  ##  ##  ##  ##  ##  ##  ##  ##  ##  ##  ##  ##  ##  ##  ##  ##  ##  ##  ##  ##  ##  ##  ##  ##  ##  ##  ##  ##  ##  ##  ##  ##  ##  ##  ##  ##  ##  ##  ##  ##  ##  ##  ##  ##  ##  ##  ##  ##  ##  ##  ##  ##  ##  ##  ##  11  11  5  3  6  3  8  6  6  7  3  4  2  3  4  15  --  2  1  13  12  6  5  4  14  7  10  5  10  3  6  4  7  7  ##  ##  ##  ##  ##  ##  ##  ##  ##  ##  ##  ##  ##  ##  ##  ##  ##  ##  ##  ##  ##  ##  ##  ##  ##  ##  ##  ##  ##  ##  ##  ##  ##  ##  ##  ##  ##  ##  ##  ##  ##  ##  ##  ##  ##  ##  ##  ##  ##  ##  ##  ##  ##  ##  ##  ##  ##  ##  ##  ##  ##  ##  ##  ##  ##  ##  ##  ##  ##  ##  ##  ##  ##  ##  ##  ##  ##  ##  ##  ##  ##  ##  ##  ##  ##  ##  ##  ##  ##  ##  ##  ##  ##  ##  ##  ##  ##  ##  ##  ##  ##  ##  ##  ##  ##  ##  ##  ##  ##  ##  ##  ##  ##  ##  ##  ##  ##  ##  ##  ##  ##  ##  ##  ##  ##  ##  ## [2001_8]127	: ##  ##  ##  ##  ##  ##  ##  ##  ##  ##  ##  ##  ##  ##  ##  ##  ##  ##  ##  ##  ##  ##  ##  ##  ##  ##  ##  ##  ##  ##  ##  ##  ##  ##  ##  ##  ##  ##  ##  ##  ##  ##  ##  ##  ##  ##  ##  ##  ##  ##  ##  ##  ##  ##  ##  ##  ##  ##  ##  ##  ##  ##  ##  ##  ##  ##  ##  ##  ##  ##  ##  ##  ##  ##  ##  ##  ##  ##  ##  ##  ##  ##  ##  ##  ##  ##  ##  ##  ##  ##  ##  ##  ##  ##  ##  ##  ##  ##  ##  ##  ##  ##  ##  ##  ##  ##  ##  ##  ##  13  13  7  5  6  5  10  8  8  9  5  6  4  5  6  15  2  --  3  15  14  8  7  6  12  7  12  7  10  5  8  4  9  9  ##  ##  ##  ##  ##  ##  ##  ##  ##  ##  ##  ##  ##  ##  ##  ##  ##  ##  ##  ##  ##  ##  ##  ##  ##  ##  ##  ##  ##  ##  ##  ##  ##  ##  ##  ##  ##  ##  ##  ##  ##  ##  ##  ##  ##  ##  ##  ##  ##  ##  ##  ##  ##  ##  ##  ##  ##  ##  ##  ##  ##  ##  ##  ##  ##  ##  ##  ##  ##  ##  ##  ##  ##  ##  ##  ##  ##  ##  ##  ##  ##  ##  ##  ##  ##  ##  ##  ##  ##  ##  ##  ##  ##  ##  ##  ##  ##  ##  ##  ##  ##  ##  ##  ##  ##  ##  ##  ##  ##  ##  ##  ##  ##  ##  ##  ##  ##  ##  ##  ##  ##  ##  ##  ##  ##  ##  ## [2002_8]128	: ##  ##  ##  ##  ##  ##  ##  ##  ##  ##  ##  ##  ##  ##  ##  ##  ##  ##  ##  ##  ##  ##  ##  ##  ##  ##  ##  ##  ##  ##  ##  ##  ##  ##  ##  ##  ##  ##  ##  ##  ##  ##  ##  ##  ##  ##  ##  ##  ##  ##  ##  ##  ##  ##  ##  ##  ##  ##  ##  ##  ##  ##  ##  ##  ##  ##  ##  ##  ##  ##  ##  ##  ##  ##  ##  ##  ##  ##  ##  ##  ##  ##  ##  ##  ##  ##  ##  ##  ##  ##  ##  ##  ##  ##  ##  ##  ##  ##  ##  ##  ##  ##  ##  ##  ##  ##  ##  ##  ##  10  12  4  2  5  2  7  5  5  6  2  3  1  2  3  14  1  3  --  12  11  5  4  3  13  6  9  6  9  2  5  3  6  6  ##  ##  ##  ##  ##  ##  ##  ##  ##  ##  ##  ##  ##  ##  ##  ##  ##  ##  ##  ##  ##  ##  ##  ##  ##  ##  ##  ##  ##  ##  ##  ##  ##  ##  ##  ##  ##  ##  ##  ##  ##  ##  ##  ##  ##  ##  ##  ##  ##  ##  ##  ##  ##  ##  ##  ##  ##  ##  ##  ##  ##  ##  ##  ##  ##  ##  ##  ##  ##  ##  ##  ##  ##  ##  ##  ##  ##  ##  ##  ##  ##  ##  ##  ##  ##  ##  ##  ##  ##  ##  ##  ##  ##  ##  ##  ##  ##  ##  ##  ##  ##  ##  ##  ##  ##  ##  ##  ##  ##  ##  ##  ##  ##  ##  ##  ##  ##  ##  ##  ##  ##  ##  ##  ##  ##  ##  ## [2013_8]129	: ##  ##  ##  ##  ##  ##  ##  ##  ##  ##  ##  ##  ##  ##  ##  ##  ##  ##  ##  ##  ##  ##  ##  ##  ##  ##  ##  ##  ##  ##  ##  ##  ##  ##  ##  ##  ##  ##  ##  ##  ##  ##  ##  ##  ##  ##  ##  ##  ##  ##  ##  ##  ##  ##  ##  ##  ##  ##  ##  ##  ##  ##  ##  ##  ##  ##  ##  ##  ##  ##  ##  ##  ##  ##  ##  ##  ##  ##  ##  ##  ##  ##  ##  ##  ##  ##  ##  ##  ##  ##  ##  ##  ##  ##  ##  ##  ##  ##  ##  ##  ##  ##  ##  ##  ##  ##  ##  ##  ##  4  6  13  14  13  11  15  13  13  13  12  13  11  12  13  9  13  15  12  --  1  13  12  15  7  14  17  14  17  12  13  13  14  14  ##  ##  ##  ##  ##  ##  ##  ##  ##  ##  ##  ##  ##  ##  ##  ##  ##  ##  ##  ##  ##  ##  ##  ##  ##  ##  ##  ##  ##  ##  ##  ##  ##  ##  ##  ##  ##  ##  ##  ##  ##  ##  ##  ##  ##  ##  ##  ##  ##  ##  ##  ##  ##  ##  ##  ##  ##  ##  ##  ##  ##  ##  ##  ##  ##  ##  ##  ##  ##  ##  ##  ##  ##  ##  ##  ##  ##  ##  ##  ##  ##  ##  ##  ##  ##  ##  ##  ##  ##  ##  ##  ##  ##  ##  ##  ##  ##  ##  ##  ##  ##  ##  ##  ##  ##  ##  ##  ##  ##  ##  ##  ##  ##  ##  ##  ##  ##  ##  ##  ##  ##  ##  ##  ##  ##  ##  ## [2014_8]130	: ##  ##  ##  ##  ##  ##  ##  ##  ##  ##  ##  ##  ##  ##  ##  ##  ##  ##  ##  ##  ##  ##  ##  ##  ##  ##  ##  ##  ##  ##  ##  ##  ##  ##  ##  ##  ##  ##  ##  ##  ##  ##  ##  ##  ##  ##  ##  ##  ##  ##  ##  ##  ##  ##  ##  ##  ##  ##  ##  ##  ##  ##  ##  ##  ##  ##  ##  ##  ##  ##  ##  ##  ##  ##  ##  ##  ##  ##  ##  ##  ##  ##  ##  ##  ##  ##  ##  ##  ##  ##  ##  ##  ##  ##  ##  ##  ##  ##  ##  ##  ##  ##  ##  ##  ##  ##  ##  ##  ##  3  5  12  13  12  10  14  12  12  12  11  12  10  11  12  8  12  14  11  1  --  12  11  14  6  13  16  13  16  11  12  12  13  13  ##  ##  ##  ##  ##  ##  ##  ##  ##  ##  ##  ##  ##  ##  ##  ##  ##  ##  ##  ##  ##  ##  ##  ##  ##  ##  ##  ##  ##  ##  ##  ##  ##  ##  ##  ##  ##  ##  ##  ##  ##  ##  ##  ##  ##  ##  ##  ##  ##  ##  ##  ##  ##  ##  ##  ##  ##  ##  ##  ##  ##  ##  ##  ##  ##  ##  ##  ##  ##  ##  ##  ##  ##  ##  ##  ##  ##  ##  ##  ##  ##  ##  ##  ##  ##  ##  ##  ##  ##  ##  ##  ##  ##  ##  ##  ##  ##  ##  ##  ##  ##  ##  ##  ##  ##  ##  ##  ##  ##  ##  ##  ##  ##  ##  ##  ##  ##  ##  ##  ##  ##  ##  ##  ##  ##  ##  ## [2036_8]131	: ##  ##  ##  ##  ##  ##  ##  ##  ##  ##  ##  ##  ##  ##  ##  ##  ##  ##  ##  ##  ##  ##  ##  ##  ##  ##  ##  ##  ##  ##  ##  ##  ##  ##  ##  ##  ##  ##  ##  ##  ##  ##  ##  ##  ##  ##  ##  ##  ##  ##  ##  ##  ##  ##  ##  ##  ##  ##  ##  ##  ##  ##  ##  ##  ##  ##  ##  ##  ##  ##  ##  ##  ##  ##  ##  ##  ##  ##  ##  ##  ##  ##  ##  ##  ##  ##  ##  ##  ##  ##  ##  ##  ##  ##  ##  ##  ##  ##  ##  ##  ##  ##  ##  ##  ##  ##  ##  ##  ##  9  13  7  7  4  5  4  2  4  5  5  6  4  3  8  15  6  8  5  13  12  --  1  8  10  5  6  3  6  5  2  6  5  5  ##  ##  ##  ##  ##  ##  ##  ##  ##  ##  ##  ##  ##  ##  ##  ##  ##  ##  ##  ##  ##  ##  ##  ##  ##  ##  ##  ##  ##  ##  ##  ##  ##  ##  ##  ##  ##  ##  ##  ##  ##  ##  ##  ##  ##  ##  ##  ##  ##  ##  ##  ##  ##  ##  ##  ##  ##  ##  ##  ##  ##  ##  ##  ##  ##  ##  ##  ##  ##  ##  ##  ##  ##  ##  ##  ##  ##  ##  ##  ##  ##  ##  ##  ##  ##  ##  ##  ##  ##  ##  ##  ##  ##  ##  ##  ##  ##  ##  ##  ##  ##  ##  ##  ##  ##  ##  ##  ##  ##  ##  ##  ##  ##  ##  ##  ##  ##  ##  ##  ##  ##  ##  ##  ##  ##  ##  ## [2037_8]132	: ##  ##  ##  ##  ##  ##  ##  ##  ##  ##  ##  ##  ##  ##  ##  ##  ##  ##  ##  ##  ##  ##  ##  ##  ##  ##  ##  ##  ##  ##  ##  ##  ##  ##  ##  ##  ##  ##  ##  ##  ##  ##  ##  ##  ##  ##  ##  ##  ##  ##  ##  ##  ##  ##  ##  ##  ##  ##  ##  ##  ##  ##  ##  ##  ##  ##  ##  ##  ##  ##  ##  ##  ##  ##  ##  ##  ##  ##  ##  ##  ##  ##  ##  ##  ##  ##  ##  ##  ##  ##  ##  ##  ##  ##  ##  ##  ##  ##  ##  ##  ##  ##  ##  ##  ##  ##  ##  ##  ##  8  12  6  6  3  4  3  1  3  4  4  5  3  2  7  14  5  7  4  12  11  1  --  7  9  4  5  2  5  4  1  5  4  4  ##  ##  ##  ##  ##  ##  ##  ##  ##  ##  ##  ##  ##  ##  ##  ##  ##  ##  ##  ##  ##  ##  ##  ##  ##  ##  ##  ##  ##  ##  ##  ##  ##  ##  ##  ##  ##  ##  ##  ##  ##  ##  ##  ##  ##  ##  ##  ##  ##  ##  ##  ##  ##  ##  ##  ##  ##  ##  ##  ##  ##  ##  ##  ##  ##  ##  ##  ##  ##  ##  ##  ##  ##  ##  ##  ##  ##  ##  ##  ##  ##  ##  ##  ##  ##  ##  ##  ##  ##  ##  ##  ##  ##  ##  ##  ##  ##  ##  ##  ##  ##  ##  ##  ##  ##  ##  ##  ##  ##  ##  ##  ##  ##  ##  ##  ##  ##  ##  ##  ##  ##  ##  ##  ##  ##  ##  ## [2039_8]133	: ##  ##  ##  ##  ##  ##  ##  ##  ##  ##  ##  ##  ##  ##  ##  ##  ##  ##  ##  ##  ##  ##  ##  ##  ##  ##  ##  ##  ##  ##  ##  ##  ##  ##  ##  ##  ##  ##  ##  ##  ##  ##  ##  ##  ##  ##  ##  ##  ##  ##  ##  ##  ##  ##  ##  ##  ##  ##  ##  ##  ##  ##  ##  ##  ##  ##  ##  ##  ##  ##  ##  ##  ##  ##  ##  ##  ##  ##  ##  ##  ##  ##  ##  ##  ##  ##  ##  ##  ##  ##  ##  ##  ##  ##  ##  ##  ##  ##  ##  ##  ##  ##  ##  ##  ##  ##  ##  ##  ##  13  15  7  5  8  5  10  8  8  9  5  6  4  5  6  16  4  6  3  15  14  8  7  --  16  9  12  9  12  5  8  6  9  9  ##  ##  ##  ##  ##  ##  ##  ##  ##  ##  ##  ##  ##  ##  ##  ##  ##  ##  ##  ##  ##  ##  ##  ##  ##  ##  ##  ##  ##  ##  ##  ##  ##  ##  ##  ##  ##  ##  ##  ##  ##  ##  ##  ##  ##  ##  ##  ##  ##  ##  ##  ##  ##  ##  ##  ##  ##  ##  ##  ##  ##  ##  ##  ##  ##  ##  ##  ##  ##  ##  ##  ##  ##  ##  ##  ##  ##  ##  ##  ##  ##  ##  ##  ##  ##  ##  ##  ##  ##  ##  ##  ##  ##  ##  ##  ##  ##  ##  ##  ##  ##  ##  ##  ##  ##  ##  ##  ##  ##  ##  ##  ##  ##  ##  ##  ##  ##  ##  ##  ##  ##  ##  ##  ##  ##  ##  ## [2214_8]134	: ##  ##  ##  ##  ##  ##  ##  ##  ##  ##  ##  ##  ##  ##  ##  ##  ##  ##  ##  ##  ##  ##  ##  ##  ##  ##  ##  ##  ##  ##  ##  ##  ##  ##  ##  ##  ##  ##  ##  ##  ##  ##  ##  ##  ##  ##  ##  ##  ##  ##  ##  ##  ##  ##  ##  ##  ##  ##  ##  ##  ##  ##  ##  ##  ##  ##  ##  ##  ##  ##  ##  ##  ##  ##  ##  ##  ##  ##  ##  ##  ##  ##  ##  ##  ##  ##  ##  ##  ##  ##  ##  ##  ##  ##  ##  ##  ##  ##  ##  ##  ##  ##  ##  ##  ##  ##  ##  ##  ##  3  7  14  15  10  13  12  10  12  12  13  14  12  11  14  8  14  12  13  7  6  10  9  16  --  11  14  11  12  13  10  12  13  13  ##  ##  ##  ##  ##  ##  ##  ##  ##  ##  ##  ##  ##  ##  ##  ##  ##  ##  ##  ##  ##  ##  ##  ##  ##  ##  ##  ##  ##  ##  ##  ##  ##  ##  ##  ##  ##  ##  ##  ##  ##  ##  ##  ##  ##  ##  ##  ##  ##  ##  ##  ##  ##  ##  ##  ##  ##  ##  ##  ##  ##  ##  ##  ##  ##  ##  ##  ##  ##  ##  ##  ##  ##  ##  ##  ##  ##  ##  ##  ##  ##  ##  ##  ##  ##  ##  ##  ##  ##  ##  ##  ##  ##  ##  ##  ##  ##  ##  ##  ##  ##  ##  ##  ##  ##  ##  ##  ##  ##  ##  ##  ##  ##  ##  ##  ##  ##  ##  ##  ##  ##  ##  ##  ##  ##  ##  ## [2456_8]135	: ##  ##  ##  ##  ##  ##  ##  ##  ##  ##  ##  ##  ##  ##  ##  ##  ##  ##  ##  ##  ##  ##  ##  ##  ##  ##  ##  ##  ##  ##  ##  ##  ##  ##  ##  ##  ##  ##  ##  ##  ##  ##  ##  ##  ##  ##  ##  ##  ##  ##  ##  ##  ##  ##  ##  ##  ##  ##  ##  ##  ##  ##  ##  ##  ##  ##  ##  ##  ##  ##  ##  ##  ##  ##  ##  ##  ##  ##  ##  ##  ##  ##  ##  ##  ##  ##  ##  ##  ##  ##  ##  ##  ##  ##  ##  ##  ##  ##  ##  ##  ##  ##  ##  ##  ##  ##  ##  ##  ##  12  14  8  8  5  6  7  5  1  6  6  6  5  4  9  14  7  7  6  14  13  5  4  9  11  --  9  6  7  6  5  5  2  2  ##  ##  ##  ##  ##  ##  ##  ##  ##  ##  ##  ##  ##  ##  ##  ##  ##  ##  ##  ##  ##  ##  ##  ##  ##  ##  ##  ##  ##  ##  ##  ##  ##  ##  ##  ##  ##  ##  ##  ##  ##  ##  ##  ##  ##  ##  ##  ##  ##  ##  ##  ##  ##  ##  ##  ##  ##  ##  ##  ##  ##  ##  ##  ##  ##  ##  ##  ##  ##  ##  ##  ##  ##  ##  ##  ##  ##  ##  ##  ##  ##  ##  ##  ##  ##  ##  ##  ##  ##  ##  ##  ##  ##  ##  ##  ##  ##  ##  ##  ##  ##  ##  ##  ##  ##  ##  ##  ##  ##  ##  ##  ##  ##  ##  ##  ##  ##  ##  ##  ##  ##  ##  ##  ##  ##  ##  ## [2457_8]136	: ##  ##  ##  ##  ##  ##  ##  ##  ##  ##  ##  ##  ##  ##  ##  ##  ##  ##  ##  ##  ##  ##  ##  ##  ##  ##  ##  ##  ##  ##  ##  ##  ##  ##  ##  ##  ##  ##  ##  ##  ##  ##  ##  ##  ##  ##  ##  ##  ##  ##  ##  ##  ##  ##  ##  ##  ##  ##  ##  ##  ##  ##  ##  ##  ##  ##  ##  ##  ##  ##  ##  ##  ##  ##  ##  ##  ##  ##  ##  ##  ##  ##  ##  ##  ##  ##  ##  ##  ##  ##  ##  ##  ##  ##  ##  ##  ##  ##  ##  ##  ##  ##  ##  ##  ##  ##  ##  ##  ##  13  17  11  11  8  8  8  6  8  9  9  9  8  7  12  19  10  12  9  17  16  6  5  12  14  9  --  7  10  9  6  10  9  9  ##  ##  ##  ##  ##  ##  ##  ##  ##  ##  ##  ##  ##  ##  ##  ##  ##  ##  ##  ##  ##  ##  ##  ##  ##  ##  ##  ##  ##  ##  ##  ##  ##  ##  ##  ##  ##  ##  ##  ##  ##  ##  ##  ##  ##  ##  ##  ##  ##  ##  ##  ##  ##  ##  ##  ##  ##  ##  ##  ##  ##  ##  ##  ##  ##  ##  ##  ##  ##  ##  ##  ##  ##  ##  ##  ##  ##  ##  ##  ##  ##  ##  ##  ##  ##  ##  ##  ##  ##  ##  ##  ##  ##  ##  ##  ##  ##  ##  ##  ##  ##  ##  ##  ##  ##  ##  ##  ##  ##  ##  ##  ##  ##  ##  ##  ##  ##  ##  ##  ##  ##  ##  ##  ##  ##  ##  ## [2476_8]137	: ##  ##  ##  ##  ##  ##  ##  ##  ##  ##  ##  ##  ##  ##  ##  ##  ##  ##  ##  ##  ##  ##  ##  ##  ##  ##  ##  ##  ##  ##  ##  ##  ##  ##  ##  ##  ##  ##  ##  ##  ##  ##  ##  ##  ##  ##  ##  ##  ##  ##  ##  ##  ##  ##  ##  ##  ##  ##  ##  ##  ##  ##  ##  ##  ##  ##  ##  ##  ##  ##  ##  ##  ##  ##  ##  ##  ##  ##  ##  ##  ##  ##  ##  ##  ##  ##  ##  ##  ##  ##  ##  ##  ##  ##  ##  ##  ##  ##  ##  ##  ##  ##  ##  ##  ##  ##  ##  ##  ##  10  12  8  8  5  6  5  3  5  6  6  6  5  4  9  16  5  7  6  14  13  3  2  9  11  6  7  --  7  6  3  7  6  6  ##  ##  ##  ##  ##  ##  ##  ##  ##  ##  ##  ##  ##  ##  ##  ##  ##  ##  ##  ##  ##  ##  ##  ##  ##  ##  ##  ##  ##  ##  ##  ##  ##  ##  ##  ##  ##  ##  ##  ##  ##  ##  ##  ##  ##  ##  ##  ##  ##  ##  ##  ##  ##  ##  ##  ##  ##  ##  ##  ##  ##  ##  ##  ##  ##  ##  ##  ##  ##  ##  ##  ##  ##  ##  ##  ##  ##  ##  ##  ##  ##  ##  ##  ##  ##  ##  ##  ##  ##  ##  ##  ##  ##  ##  ##  ##  ##  ##  ##  ##  ##  ##  ##  ##  ##  ##  ##  ##  ##  ##  ##  ##  ##  ##  ##  ##  ##  ##  ##  ##  ##  ##  ##  ##  ##  ##  ## [2478_8]138	: ##  ##  ##  ##  ##  ##  ##  ##  ##  ##  ##  ##  ##  ##  ##  ##  ##  ##  ##  ##  ##  ##  ##  ##  ##  ##  ##  ##  ##  ##  ##  ##  ##  ##  ##  ##  ##  ##  ##  ##  ##  ##  ##  ##  ##  ##  ##  ##  ##  ##  ##  ##  ##  ##  ##  ##  ##  ##  ##  ##  ##  ##  ##  ##  ##  ##  ##  ##  ##  ##  ##  ##  ##  ##  ##  ##  ##  ##  ##  ##  ##  ##  ##  ##  ##  ##  ##  ##  ##  ##  ##  ##  ##  ##  ##  ##  ##  ##  ##  ##  ##  ##  ##  ##  ##  ##  ##  ##  ##  13  17  11  11  8  9  8  4  8  9  9  9  8  7  12  16  10  10  9  17  16  6  5  12  12  7  10  7  --  9  6  8  9  9  ##  ##  ##  ##  ##  ##  ##  ##  ##  ##  ##  ##  ##  ##  ##  ##  ##  ##  ##  ##  ##  ##  ##  ##  ##  ##  ##  ##  ##  ##  ##  ##  ##  ##  ##  ##  ##  ##  ##  ##  ##  ##  ##  ##  ##  ##  ##  ##  ##  ##  ##  ##  ##  ##  ##  ##  ##  ##  ##  ##  ##  ##  ##  ##  ##  ##  ##  ##  ##  ##  ##  ##  ##  ##  ##  ##  ##  ##  ##  ##  ##  ##  ##  ##  ##  ##  ##  ##  ##  ##  ##  ##  ##  ##  ##  ##  ##  ##  ##  ##  ##  ##  ##  ##  ##  ##  ##  ##  ##  ##  ##  ##  ##  ##  ##  ##  ##  ##  ##  ##  ##  ##  ##  ##  ##  ##  ## [2775_8]139	: ##  ##  ##  ##  ##  ##  ##  ##  ##  ##  ##  ##  ##  ##  ##  ##  ##  ##  ##  ##  ##  ##  ##  ##  ##  ##  ##  ##  ##  ##  ##  ##  ##  ##  ##  ##  ##  ##  ##  ##  ##  ##  ##  ##  ##  ##  ##  ##  ##  ##  ##  ##  ##  ##  ##  ##  ##  ##  ##  ##  ##  ##  ##  ##  ##  ##  ##  ##  ##  ##  ##  ##  ##  ##  ##  ##  ##  ##  ##  ##  ##  ##  ##  ##  ##  ##  ##  ##  ##  ##  ##  ##  ##  ##  ##  ##  ##  ##  ##  ##  ##  ##  ##  ##  ##  ##  ##  ##  ##  10  12  4  4  5  2  7  5  5  6  2  2  1  2  5  14  3  5  2  12  11  5  4  5  13  6  9  6  9  --  5  3  6  6  ##  ##  ##  ##  ##  ##  ##  ##  ##  ##  ##  ##  ##  ##  ##  ##  ##  ##  ##  ##  ##  ##  ##  ##  ##  ##  ##  ##  ##  ##  ##  ##  ##  ##  ##  ##  ##  ##  ##  ##  ##  ##  ##  ##  ##  ##  ##  ##  ##  ##  ##  ##  ##  ##  ##  ##  ##  ##  ##  ##  ##  ##  ##  ##  ##  ##  ##  ##  ##  ##  ##  ##  ##  ##  ##  ##  ##  ##  ##  ##  ##  ##  ##  ##  ##  ##  ##  ##  ##  ##  ##  ##  ##  ##  ##  ##  ##  ##  ##  ##  ##  ##  ##  ##  ##  ##  ##  ##  ##  ##  ##  ##  ##  ##  ##  ##  ##  ##  ##  ##  ##  ##  ##  ##  ##  ##  ## [2798_8]140	: ##  ##  ##  ##  ##  ##  ##  ##  ##  ##  ##  ##  ##  ##  ##  ##  ##  ##  ##  ##  ##  ##  ##  ##  ##  ##  ##  ##  ##  ##  ##  ##  ##  ##  ##  ##  ##  ##  ##  ##  ##  ##  ##  ##  ##  ##  ##  ##  ##  ##  ##  ##  ##  ##  ##  ##  ##  ##  ##  ##  ##  ##  ##  ##  ##  ##  ##  ##  ##  ##  ##  ##  ##  ##  ##  ##  ##  ##  ##  ##  ##  ##  ##  ##  ##  ##  ##  ##  ##  ##  ##  ##  ##  ##  ##  ##  ##  ##  ##  ##  ##  ##  ##  ##  ##  ##  ##  ##  ##  9  13  7  7  4  5  4  2  4  5  5  5  4  3  8  15  6  8  5  13  12  2  1  8  10  5  6  3  6  5  --  6  5  5  ##  ##  ##  ##  ##  ##  ##  ##  ##  ##  ##  ##  ##  ##  ##  ##  ##  ##  ##  ##  ##  ##  ##  ##  ##  ##  ##  ##  ##  ##  ##  ##  ##  ##  ##  ##  ##  ##  ##  ##  ##  ##  ##  ##  ##  ##  ##  ##  ##  ##  ##  ##  ##  ##  ##  ##  ##  ##  ##  ##  ##  ##  ##  ##  ##  ##  ##  ##  ##  ##  ##  ##  ##  ##  ##  ##  ##  ##  ##  ##  ##  ##  ##  ##  ##  ##  ##  ##  ##  ##  ##  ##  ##  ##  ##  ##  ##  ##  ##  ##  ##  ##  ##  ##  ##  ##  ##  ##  ##  ##  ##  ##  ##  ##  ##  ##  ##  ##  ##  ##  ##  ##  ##  ##  ##  ##  ## [2799_8]141	: ##  ##  ##  ##  ##  ##  ##  ##  ##  ##  ##  ##  ##  ##  ##  ##  ##  ##  ##  ##  ##  ##  ##  ##  ##  ##  ##  ##  ##  ##  ##  ##  ##  ##  ##  ##  ##  ##  ##  ##  ##  ##  ##  ##  ##  ##  ##  ##  ##  ##  ##  ##  ##  ##  ##  ##  ##  ##  ##  ##  ##  ##  ##  ##  ##  ##  ##  ##  ##  ##  ##  ##  ##  ##  ##  ##  ##  ##  ##  ##  ##  ##  ##  ##  ##  ##  ##  ##  ##  ##  ##  ##  ##  ##  ##  ##  ##  ##  ##  ##  ##  ##  ##  ##  ##  ##  ##  ##  ##  11  13  5  5  6  3  8  6  6  7  3  3  2  3  6  13  4  4  3  13  12  6  5  6  12  5  10  7  8  3  6  --  5  7  ##  ##  ##  ##  ##  ##  ##  ##  ##  ##  ##  ##  ##  ##  ##  ##  ##  ##  ##  ##  ##  ##  ##  ##  ##  ##  ##  ##  ##  ##  ##  ##  ##  ##  ##  ##  ##  ##  ##  ##  ##  ##  ##  ##  ##  ##  ##  ##  ##  ##  ##  ##  ##  ##  ##  ##  ##  ##  ##  ##  ##  ##  ##  ##  ##  ##  ##  ##  ##  ##  ##  ##  ##  ##  ##  ##  ##  ##  ##  ##  ##  ##  ##  ##  ##  ##  ##  ##  ##  ##  ##  ##  ##  ##  ##  ##  ##  ##  ##  ##  ##  ##  ##  ##  ##  ##  ##  ##  ##  ##  ##  ##  ##  ##  ##  ##  ##  ##  ##  ##  ##  ##  ##  ##  ##  ##  ## [2896_8]142	: ##  ##  ##  ##  ##  ##  ##  ##  ##  ##  ##  ##  ##  ##  ##  ##  ##  ##  ##  ##  ##  ##  ##  ##  ##  ##  ##  ##  ##  ##  ##  ##  ##  ##  ##  ##  ##  ##  ##  ##  ##  ##  ##  ##  ##  ##  ##  ##  ##  ##  ##  ##  ##  ##  ##  ##  ##  ##  ##  ##  ##  ##  ##  ##  ##  ##  ##  ##  ##  ##  ##  ##  ##  ##  ##  ##  ##  ##  ##  ##  ##  ##  ##  ##  ##  ##  ##  ##  ##  ##  ##  ##  ##  ##  ##  ##  ##  ##  ##  ##  ##  ##  ##  ##  ##  ##  ##  ##  ##  12  14  8  8  5  6  7  5  1  6  6  6  5  4  9  16  7  9  6  14  13  5  4  9  13  2  9  6  9  6  5  5  --  2  ##  ##  ##  ##  ##  ##  ##  ##  ##  ##  ##  ##  ##  ##  ##  ##  ##  ##  ##  ##  ##  ##  ##  ##  ##  ##  ##  ##  ##  ##  ##  ##  ##  ##  ##  ##  ##  ##  ##  ##  ##  ##  ##  ##  ##  ##  ##  ##  ##  ##  ##  ##  ##  ##  ##  ##  ##  ##  ##  ##  ##  ##  ##  ##  ##  ##  ##  ##  ##  ##  ##  ##  ##  ##  ##  ##  ##  ##  ##  ##  ##  ##  ##  ##  ##  ##  ##  ##  ##  ##  ##  ##  ##  ##  ##  ##  ##  ##  ##  ##  ##  ##  ##  ##  ##  ##  ##  ##  ##  ##  ##  ##  ##  ##  ##  ##  ##  ##  ##  ##  ##  ##  ##  ##  ##  ##  ## [2925_8]143	: ##  ##  ##  ##  ##  ##  ##  ##  ##  ##  ##  ##  ##  ##  ##  ##  ##  ##  ##  ##  ##  ##  ##  ##  ##  ##  ##  ##  ##  ##  ##  ##  ##  ##  ##  ##  ##  ##  ##  ##  ##  ##  ##  ##  ##  ##  ##  ##  ##  ##  ##  ##  ##  ##  ##  ##  ##  ##  ##  ##  ##  ##  ##  ##  ##  ##  ##  ##  ##  ##  ##  ##  ##  ##  ##  ##  ##  ##  ##  ##  ##  ##  ##  ##  ##  ##  ##  ##  ##  ##  ##  ##  ##  ##  ##  ##  ##  ##  ##  ##  ##  ##  ##  ##  ##  ##  ##  ##  ##  12  14  8  8  3  6  7  5  1  6  6  6  5  4  9  16  7  9  6  14  13  5  4  9  13  2  9  6  9  6  5  7  2  --  ##  ##  ##  ##  ##  ##  ##  ##  ##  ##  ##  ##  ##  ##  ##  ##  ##  ##  ##  ##  ##  ##  ##  ##  ##  ##  ##  ##  ##  ##  ##  ##  ##  ##  ##  ##  ##  ##  ##  ##  ##  ##  ##  ##  ##  ##  ##  ##  ##  ##  ##  ##  ##  ##  ##  ##  ##  ##  ##  ##  ##  ##  ##  ##  ##  ##  ##  ##  ##  ##  ##  ##  ##  ##  ##  ##  ##  ##  ##  ##  ##  ##  ##  ##  ##  ##  ##  ##  ##  ##  ##  ##  ##  ##  ##  ##  ##  ##  ##  ##  ##  ##  ##  ##  ##  ##  ##  ##  ##  ##  ##  ##  ##  ##  ##  ##  ##  ##  ##  ##  ##  ##  ##  ##  ##  ##  ## [859_9]144	: ##  ##  ##  ##  ##  ##  ##  ##  ##  ##  ##  ##  ##  ##  ##  ##  ##  ##  ##  ##  ##  ##  ##  ##  ##  ##  ##  ##  ##  ##  ##  ##  ##  ##  ##  ##  ##  ##  ##  ##  ##  ##  ##  ##  ##  ##  ##  ##  ##  ##  ##  ##  ##  ##  ##  ##  ##  ##  ##  ##  ##  ##  ##  ##  ##  ##  ##  ##  ##  ##  ##  ##  ##  ##  ##  ##  ##  ##  ##  ##  ##  ##  ##  ##  ##  ##  ##  ##  ##  ##  ##  ##  ##  ##  ##  ##  ##  ##  ##  ##  ##  ##  ##  ##  ##  ##  ##  ##  ##  ##  ##  ##  ##  ##  ##  ##  ##  ##  ##  ##  ##  ##  ##  ##  ##  ##  ##  ##  ##  ##  ##  ##  ##  ##  ##  ##  ##  ##  ##  ##  ##  ##  ##  --  1  ##  ##  ##  ##  ##  ##  ##  ##  ##  ##  ##  ##  ##  ##  ##  ##  ##  ##  ##  ##  ##  ##  ##  ##  ##  ##  ##  ##  ##  ##  ##  ##  ##  ##  ##  ##  ##  ##  ##  ##  ##  ##  ##  ##  ##  ##  ##  ##  ##  ##  ##  ##  ##  ##  ##  ##  ##  ##  ##  ##  ##  ##  ##  ##  ##  ##  ##  ##  ##  ##  ##  ##  ##  ##  ##  ##  ##  ##  ##  ##  ##  ##  ##  ##  ##  ##  ##  ##  ##  ##  ##  ##  ##  ##  ##  ##  ##  ##  ##  ##  ##  ##  ##  ##  ##  ##  ##  ##  ##  ##  ##  ##  ##  ##  ##  ##  ##  ##  ##  ##  ##  ##  ##  ##  ## [862_9]145	: ##  ##  ##  ##  ##  ##  ##  ##  ##  ##  ##  ##  ##  ##  ##  ##  ##  ##  ##  ##  ##  ##  ##  ##  ##  ##  ##  ##  ##  ##  ##  ##  ##  ##  ##  ##  ##  ##  ##  ##  ##  ##  ##  ##  ##  ##  ##  ##  ##  ##  ##  ##  ##  ##  ##  ##  ##  ##  ##  ##  ##  ##  ##  ##  ##  ##  ##  ##  ##  ##  ##  ##  ##  ##  ##  ##  ##  ##  ##  ##  ##  ##  ##  ##  ##  ##  ##  ##  ##  ##  ##  ##  ##  ##  ##  ##  ##  ##  ##  ##  ##  ##  ##  ##  ##  ##  ##  ##  ##  ##  ##  ##  ##  ##  ##  ##  ##  ##  ##  ##  ##  ##  ##  ##  ##  ##  ##  ##  ##  ##  ##  ##  ##  ##  ##  ##  ##  ##  ##  ##  ##  ##  ##  1  --  ##  ##  ##  ##  ##  ##  ##  ##  ##  ##  ##  ##  ##  ##  ##  ##  ##  ##  ##  ##  ##  ##  ##  ##  ##  ##  ##  ##  ##  ##  ##  ##  ##  ##  ##  ##  ##  ##  ##  ##  ##  ##  ##  ##  ##  ##  ##  ##  ##  ##  ##  ##  ##  ##  ##  ##  ##  ##  ##  ##  ##  ##  ##  ##  ##  ##  ##  ##  ##  ##  ##  ##  ##  ##  ##  ##  ##  ##  ##  ##  ##  ##  ##  ##  ##  ##  ##  ##  ##  ##  ##  ##  ##  ##  ##  ##  ##  ##  ##  ##  ##  ##  ##  ##  ##  ##  ##  ##  ##  ##  ##  ##  ##  ##  ##  ##  ##  ##  ##  ##  ##  ##  ##  ##  ## [2274_17]146	: ##  ##  ##  ##  ##  ##  ##  ##  ##  ##  ##  ##  ##  ##  ##  ##  ##  ##  ##  ##  ##  ##  ##  ##  ##  ##  ##  ##  ##  ##  ##  ##  ##  ##  ##  ##  ##  ##  ##  ##  ##  ##  ##  ##  ##  ##  ##  ##  ##  ##  ##  ##  ##  ##  ##  ##  ##  ##  ##  ##  ##  ##  ##  ##  ##  ##  ##  ##  ##  ##  ##  ##  ##  ##  ##  ##  ##  ##  ##  ##  ##  ##  ##  ##  ##  ##  ##  ##  ##  ##  ##  ##  ##  ##  ##  ##  ##  ##  ##  ##  ##  ##  ##  ##  ##  ##  ##  ##  ##  ##  ##  ##  ##  ##  ##  ##  ##  ##  ##  ##  ##  ##  ##  ##  ##  ##  ##  ##  ##  ##  ##  ##  ##  ##  ##  ##  ##  ##  ##  ##  ##  ##  ##  ##  ##  --  ##  ##  ##  ##  ##  ##  ##  ##  ##  ##  ##  ##  ##  ##  ##  ##  ##  ##  ##  ##  ##  ##  ##  ##  ##  ##  ##  ##  ##  ##  ##  ##  ##  ##  ##  ##  ##  ##  ##  ##  ##  ##  ##  ##  ##  ##  ##  ##  ##  ##  ##  ##  ##  ##  ##  ##  ##  ##  ##  ##  ##  ##  ##  ##  ##  ##  ##  ##  ##  ##  ##  ##  ##  ##  ##  ##  ##  ##  ##  ##  ##  ##  ##  ##  ##  ##  ##  ##  ##  ##  ##  ##  ##  ##  ##  ##  ##  ##  ##  ##  ##  ##  ##  ##  ##  ##  ##  ##  ##  ##  ##  ##  ##  ##  ##  ##  ##  ##  ##  ##  ##  ##  ##  ## [2277_22]147	: ##  ##  ##  ##  ##  ##  ##  ##  ##  ##  ##  ##  ##  ##  ##  ##  ##  ##  ##  ##  ##  ##  ##  ##  ##  ##  ##  ##  ##  ##  ##  ##  ##  ##  ##  ##  ##  ##  ##  ##  ##  ##  ##  ##  ##  ##  ##  ##  ##  ##  ##  ##  ##  ##  ##  ##  ##  ##  ##  ##  ##  ##  ##  ##  ##  ##  ##  ##  ##  ##  ##  ##  ##  ##  ##  ##  ##  ##  ##  ##  ##  ##  ##  ##  ##  ##  ##  ##  ##  ##  ##  ##  ##  ##  ##  ##  ##  ##  ##  ##  ##  ##  ##  ##  ##  ##  ##  ##  ##  ##  ##  ##  ##  ##  ##  ##  ##  ##  ##  ##  ##  ##  ##  ##  ##  ##  ##  ##  ##  ##  ##  ##  ##  ##  ##  ##  ##  ##  ##  ##  ##  ##  ##  ##  ##  ##  --  ##  ##  ##  ##  ##  ##  ##  ##  ##  ##  ##  ##  ##  ##  ##  ##  ##  ##  ##  ##  ##  ##  ##  ##  ##  ##  ##  ##  ##  ##  ##  ##  ##  ##  ##  ##  ##  ##  ##  ##  ##  ##  ##  ##  ##  ##  ##  ##  ##  ##  ##  ##  ##  ##  ##  ##  ##  ##  ##  ##  ##  ##  ##  ##  ##  ##  ##  ##  ##  ##  ##  ##  ##  ##  ##  ##  ##  ##  ##  ##  ##  ##  ##  ##  ##  ##  ##  ##  ##  ##  ##  ##  ##  ##  ##  ##  ##  ##  ##  ##  ##  ##  ##  ##  ##  ##  ##  ##  ##  ##  ##  ##  ##  ##  ##  ##  ##  ##  ##  ##  ##  ##  ## [2278_19]148	: ##  ##  ##  ##  ##  ##  ##  ##  ##  ##  ##  ##  ##  ##  ##  ##  ##  ##  ##  ##  ##  ##  ##  ##  ##  ##  ##  ##  ##  ##  ##  ##  ##  ##  ##  ##  ##  ##  ##  ##  ##  ##  ##  ##  ##  ##  ##  ##  ##  ##  ##  ##  ##  ##  ##  ##  ##  ##  ##  ##  ##  ##  ##  ##  ##  ##  ##  ##  ##  ##  ##  ##  ##  ##  ##  ##  ##  ##  ##  ##  ##  ##  ##  ##  ##  ##  ##  ##  ##  ##  ##  ##  ##  ##  ##  ##  ##  ##  ##  ##  ##  ##  ##  ##  ##  ##  ##  ##  ##  ##  ##  ##  ##  ##  ##  ##  ##  ##  ##  ##  ##  ##  ##  ##  ##  ##  ##  ##  ##  ##  ##  ##  ##  ##  ##  ##  ##  ##  ##  ##  ##  ##  ##  ##  ##  ##  ##  --  ##  ##  ##  ##  ##  ##  ##  ##  ##  ##  ##  ##  ##  ##  ##  ##  ##  ##  ##  ##  ##  ##  ##  ##  ##  ##  ##  ##  ##  ##  ##  ##  ##  ##  ##  ##  ##  ##  ##  ##  ##  ##  ##  ##  ##  ##  ##  ##  ##  ##  ##  ##  ##  ##  ##  ##  ##  ##  ##  ##  ##  ##  ##  ##  ##  ##  ##  ##  ##  ##  ##  ##  ##  ##  ##  ##  ##  ##  ##  ##  ##  ##  ##  ##  ##  ##  ##  ##  ##  ##  ##  ##  ##  ##  ##  ##  ##  ##  ##  ##  ##  ##  ##  ##  ##  ##  ##  ##  ##  ##  ##  ##  ##  ##  ##  ##  ##  ##  ##  ##  ##  ## [2281_23]149	: ##  ##  ##  ##  ##  ##  ##  ##  ##  ##  ##  ##  ##  ##  ##  ##  ##  ##  ##  ##  ##  ##  ##  ##  ##  ##  ##  ##  ##  ##  ##  ##  ##  ##  ##  ##  ##  ##  ##  ##  ##  ##  ##  ##  ##  ##  ##  ##  ##  ##  ##  ##  ##  ##  ##  ##  ##  ##  ##  ##  ##  ##  ##  ##  ##  ##  ##  ##  ##  ##  ##  ##  ##  ##  ##  ##  ##  ##  ##  ##  ##  ##  ##  ##  ##  ##  ##  ##  ##  ##  ##  ##  ##  ##  ##  ##  ##  ##  ##  ##  ##  ##  ##  ##  ##  ##  ##  ##  ##  ##  ##  ##  ##  ##  ##  ##  ##  ##  ##  ##  ##  ##  ##  ##  ##  ##  ##  ##  ##  ##  ##  ##  ##  ##  ##  ##  ##  ##  ##  ##  ##  ##  ##  ##  ##  ##  ##  ##  --  ##  ##  ##  ##  ##  ##  ##  ##  ##  ##  ##  ##  ##  ##  ##  ##  ##  ##  ##  ##  ##  ##  ##  ##  ##  ##  ##  ##  ##  ##  ##  ##  ##  ##  ##  ##  ##  ##  ##  ##  ##  ##  ##  ##  ##  ##  ##  ##  ##  ##  ##  ##  ##  ##  ##  ##  ##  ##  ##  ##  ##  ##  ##  ##  ##  ##  ##  ##  ##  ##  ##  ##  ##  ##  ##  ##  ##  ##  ##  ##  ##  ##  ##  ##  ##  ##  ##  ##  ##  ##  ##  ##  ##  ##  ##  ##  ##  ##  ##  ##  ##  ##  ##  ##  ##  ##  ##  ##  ##  ##  ##  ##  ##  ##  ##  ##  ##  ##  ##  ##  ## [2304_10]150	: ##  ##  ##  ##  ##  ##  ##  ##  ##  ##  ##  ##  ##  ##  ##  ##  ##  ##  ##  ##  ##  ##  ##  ##  ##  ##  ##  ##  ##  ##  ##  ##  ##  ##  ##  ##  ##  ##  ##  ##  ##  ##  ##  ##  ##  ##  ##  ##  ##  ##  ##  ##  ##  ##  ##  ##  ##  ##  ##  ##  ##  ##  ##  ##  ##  ##  ##  ##  ##  ##  ##  ##  ##  ##  ##  ##  ##  ##  ##  ##  ##  ##  ##  ##  ##  ##  ##  ##  ##  ##  ##  ##  ##  ##  ##  ##  ##  ##  ##  ##  ##  ##  ##  ##  ##  ##  ##  ##  ##  ##  ##  ##  ##  ##  ##  ##  ##  ##  ##  ##  ##  ##  ##  ##  ##  ##  ##  ##  ##  ##  ##  ##  ##  ##  ##  ##  ##  ##  ##  ##  ##  ##  ##  ##  ##  ##  ##  ##  ##  --  11  12  12  ##  ##  ##  ##  ##  ##  ##  ##  ##  ##  ##  ##  ##  ##  ##  ##  ##  ##  ##  ##  ##  ##  ##  ##  ##  ##  ##  ##  ##  ##  ##  ##  ##  ##  ##  ##  ##  ##  ##  ##  ##  ##  ##  ##  ##  ##  ##  ##  ##  ##  ##  ##  ##  ##  ##  ##  ##  ##  ##  ##  ##  ##  ##  ##  ##  ##  ##  ##  ##  ##  ##  ##  ##  ##  ##  ##  ##  ##  ##  ##  ##  ##  ##  ##  ##  ##  ##  ##  ##  ##  ##  ##  ##  ##  ##  ##  ##  ##  ##  ##  ##  ##  ##  ##  ##  ##  ##  ##  ##  ##  ##  ##  ##  ##  ##  ##  ## [2321_10]151	: ##  ##  ##  ##  ##  ##  ##  ##  ##  ##  ##  ##  ##  ##  ##  ##  ##  ##  ##  ##  ##  ##  ##  ##  ##  ##  ##  ##  ##  ##  ##  ##  ##  ##  ##  ##  ##  ##  ##  ##  ##  ##  ##  ##  ##  ##  ##  ##  ##  ##  ##  ##  ##  ##  ##  ##  ##  ##  ##  ##  ##  ##  ##  ##  ##  ##  ##  ##  ##  ##  ##  ##  ##  ##  ##  ##  ##  ##  ##  ##  ##  ##  ##  ##  ##  ##  ##  ##  ##  ##  ##  ##  ##  ##  ##  ##  ##  ##  ##  ##  ##  ##  ##  ##  ##  ##  ##  ##  ##  ##  ##  ##  ##  ##  ##  ##  ##  ##  ##  ##  ##  ##  ##  ##  ##  ##  ##  ##  ##  ##  ##  ##  ##  ##  ##  ##  ##  ##  ##  ##  ##  ##  ##  ##  ##  ##  ##  ##  ##  11  --  1  1  ##  ##  ##  ##  ##  ##  ##  ##  ##  ##  ##  ##  ##  ##  ##  ##  ##  ##  ##  ##  ##  ##  ##  ##  ##  ##  ##  ##  ##  ##  ##  ##  ##  ##  ##  ##  ##  ##  ##  ##  ##  ##  ##  ##  ##  ##  ##  ##  ##  ##  ##  ##  ##  ##  ##  ##  ##  ##  ##  ##  ##  ##  ##  ##  ##  ##  ##  ##  ##  ##  ##  ##  ##  ##  ##  ##  ##  ##  ##  ##  ##  ##  ##  ##  ##  ##  ##  ##  ##  ##  ##  ##  ##  ##  ##  ##  ##  ##  ##  ##  ##  ##  ##  ##  ##  ##  ##  ##  ##  ##  ##  ##  ##  ##  ##  ##  ## [2031_10]152	: ##  ##  ##  ##  ##  ##  ##  ##  ##  ##  ##  ##  ##  ##  ##  ##  ##  ##  ##  ##  ##  ##  ##  ##  ##  ##  ##  ##  ##  ##  ##  ##  ##  ##  ##  ##  ##  ##  ##  ##  ##  ##  ##  ##  ##  ##  ##  ##  ##  ##  ##  ##  ##  ##  ##  ##  ##  ##  ##  ##  ##  ##  ##  ##  ##  ##  ##  ##  ##  ##  ##  ##  ##  ##  ##  ##  ##  ##  ##  ##  ##  ##  ##  ##  ##  ##  ##  ##  ##  ##  ##  ##  ##  ##  ##  ##  ##  ##  ##  ##  ##  ##  ##  ##  ##  ##  ##  ##  ##  ##  ##  ##  ##  ##  ##  ##  ##  ##  ##  ##  ##  ##  ##  ##  ##  ##  ##  ##  ##  ##  ##  ##  ##  ##  ##  ##  ##  ##  ##  ##  ##  ##  ##  ##  ##  ##  ##  ##  ##  12  1  --  2  ##  ##  ##  ##  ##  ##  ##  ##  ##  ##  ##  ##  ##  ##  ##  ##  ##  ##  ##  ##  ##  ##  ##  ##  ##  ##  ##  ##  ##  ##  ##  ##  ##  ##  ##  ##  ##  ##  ##  ##  ##  ##  ##  ##  ##  ##  ##  ##  ##  ##  ##  ##  ##  ##  ##  ##  ##  ##  ##  ##  ##  ##  ##  ##  ##  ##  ##  ##  ##  ##  ##  ##  ##  ##  ##  ##  ##  ##  ##  ##  ##  ##  ##  ##  ##  ##  ##  ##  ##  ##  ##  ##  ##  ##  ##  ##  ##  ##  ##  ##  ##  ##  ##  ##  ##  ##  ##  ##  ##  ##  ##  ##  ##  ##  ##  ##  ## [2034_10]153	: ##  ##  ##  ##  ##  ##  ##  ##  ##  ##  ##  ##  ##  ##  ##  ##  ##  ##  ##  ##  ##  ##  ##  ##  ##  ##  ##  ##  ##  ##  ##  ##  ##  ##  ##  ##  ##  ##  ##  ##  ##  ##  ##  ##  ##  ##  ##  ##  ##  ##  ##  ##  ##  ##  ##  ##  ##  ##  ##  ##  ##  ##  ##  ##  ##  ##  ##  ##  ##  ##  ##  ##  ##  ##  ##  ##  ##  ##  ##  ##  ##  ##  ##  ##  ##  ##  ##  ##  ##  ##  ##  ##  ##  ##  ##  ##  ##  ##  ##  ##  ##  ##  ##  ##  ##  ##  ##  ##  ##  ##  ##  ##  ##  ##  ##  ##  ##  ##  ##  ##  ##  ##  ##  ##  ##  ##  ##  ##  ##  ##  ##  ##  ##  ##  ##  ##  ##  ##  ##  ##  ##  ##  ##  ##  ##  ##  ##  ##  ##  12  1  2  --  ##  ##  ##  ##  ##  ##  ##  ##  ##  ##  ##  ##  ##  ##  ##  ##  ##  ##  ##  ##  ##  ##  ##  ##  ##  ##  ##  ##  ##  ##  ##  ##  ##  ##  ##  ##  ##  ##  ##  ##  ##  ##  ##  ##  ##  ##  ##  ##  ##  ##  ##  ##  ##  ##  ##  ##  ##  ##  ##  ##  ##  ##  ##  ##  ##  ##  ##  ##  ##  ##  ##  ##  ##  ##  ##  ##  ##  ##  ##  ##  ##  ##  ##  ##  ##  ##  ##  ##  ##  ##  ##  ##  ##  ##  ##  ##  ##  ##  ##  ##  ##  ##  ##  ##  ##  ##  ##  ##  ##  ##  ##  ##  ##  ##  ##  ##  ## [1560_11]154	: ##  ##  ##  ##  ##  ##  ##  ##  ##  ##  ##  ##  ##  ##  ##  ##  ##  ##  ##  ##  ##  ##  ##  ##  ##  ##  ##  ##  ##  ##  ##  ##  ##  ##  ##  ##  ##  ##  ##  ##  ##  ##  ##  ##  ##  ##  ##  ##  ##  ##  ##  ##  ##  ##  ##  ##  ##  ##  ##  ##  ##  ##  ##  ##  ##  ##  ##  ##  ##  ##  ##  ##  ##  ##  ##  ##  ##  ##  ##  ##  ##  ##  ##  ##  ##  ##  ##  ##  ##  ##  ##  ##  ##  ##  ##  ##  ##  ##  ##  ##  ##  ##  ##  ##  ##  ##  ##  ##  ##  ##  ##  ##  ##  ##  ##  ##  ##  ##  ##  ##  ##  ##  ##  ##  ##  ##  ##  ##  ##  ##  ##  ##  ##  ##  ##  ##  ##  ##  ##  ##  ##  ##  ##  ##  ##  ##  ##  ##  ##  ##  ##  ##  ##  --  1  3  4  ##  ##  ##  ##  ##  ##  ##  ##  ##  ##  ##  ##  ##  ##  ##  ##  ##  ##  ##  ##  ##  ##  ##  ##  ##  ##  ##  ##  ##  ##  ##  ##  ##  ##  ##  ##  ##  ##  ##  ##  ##  ##  ##  ##  ##  ##  ##  ##  ##  ##  ##  ##  ##  ##  ##  ##  ##  ##  ##  ##  ##  ##  ##  ##  ##  ##  ##  ##  ##  ##  ##  ##  ##  ##  ##  ##  ##  ##  ##  ##  ##  ##  ##  ##  ##  ##  ##  ##  ##  ##  ##  ##  ##  ##  ##  ##  ##  ##  ##  ##  ##  ##  ##  ##  ##  ##  ##  ##  ##  ##  ##  ##  ## [2347_11]155	: ##  ##  ##  ##  ##  ##  ##  ##  ##  ##  ##  ##  ##  ##  ##  ##  ##  ##  ##  ##  ##  ##  ##  ##  ##  ##  ##  ##  ##  ##  ##  ##  ##  ##  ##  ##  ##  ##  ##  ##  ##  ##  ##  ##  ##  ##  ##  ##  ##  ##  ##  ##  ##  ##  ##  ##  ##  ##  ##  ##  ##  ##  ##  ##  ##  ##  ##  ##  ##  ##  ##  ##  ##  ##  ##  ##  ##  ##  ##  ##  ##  ##  ##  ##  ##  ##  ##  ##  ##  ##  ##  ##  ##  ##  ##  ##  ##  ##  ##  ##  ##  ##  ##  ##  ##  ##  ##  ##  ##  ##  ##  ##  ##  ##  ##  ##  ##  ##  ##  ##  ##  ##  ##  ##  ##  ##  ##  ##  ##  ##  ##  ##  ##  ##  ##  ##  ##  ##  ##  ##  ##  ##  ##  ##  ##  ##  ##  ##  ##  ##  ##  ##  ##  1  --  4  5  ##  ##  ##  ##  ##  ##  ##  ##  ##  ##  ##  ##  ##  ##  ##  ##  ##  ##  ##  ##  ##  ##  ##  ##  ##  ##  ##  ##  ##  ##  ##  ##  ##  ##  ##  ##  ##  ##  ##  ##  ##  ##  ##  ##  ##  ##  ##  ##  ##  ##  ##  ##  ##  ##  ##  ##  ##  ##  ##  ##  ##  ##  ##  ##  ##  ##  ##  ##  ##  ##  ##  ##  ##  ##  ##  ##  ##  ##  ##  ##  ##  ##  ##  ##  ##  ##  ##  ##  ##  ##  ##  ##  ##  ##  ##  ##  ##  ##  ##  ##  ##  ##  ##  ##  ##  ##  ##  ##  ##  ##  ##  ##  ## [2786_11]156	: ##  ##  ##  ##  ##  ##  ##  ##  ##  ##  ##  ##  ##  ##  ##  ##  ##  ##  ##  ##  ##  ##  ##  ##  ##  ##  ##  ##  ##  ##  ##  ##  ##  ##  ##  ##  ##  ##  ##  ##  ##  ##  ##  ##  ##  ##  ##  ##  ##  ##  ##  ##  ##  ##  ##  ##  ##  ##  ##  ##  ##  ##  ##  ##  ##  ##  ##  ##  ##  ##  ##  ##  ##  ##  ##  ##  ##  ##  ##  ##  ##  ##  ##  ##  ##  ##  ##  ##  ##  ##  ##  ##  ##  ##  ##  ##  ##  ##  ##  ##  ##  ##  ##  ##  ##  ##  ##  ##  ##  ##  ##  ##  ##  ##  ##  ##  ##  ##  ##  ##  ##  ##  ##  ##  ##  ##  ##  ##  ##  ##  ##  ##  ##  ##  ##  ##  ##  ##  ##  ##  ##  ##  ##  ##  ##  ##  ##  ##  ##  ##  ##  ##  ##  3  4  --  7  ##  ##  ##  ##  ##  ##  ##  ##  ##  ##  ##  ##  ##  ##  ##  ##  ##  ##  ##  ##  ##  ##  ##  ##  ##  ##  ##  ##  ##  ##  ##  ##  ##  ##  ##  ##  ##  ##  ##  ##  ##  ##  ##  ##  ##  ##  ##  ##  ##  ##  ##  ##  ##  ##  ##  ##  ##  ##  ##  ##  ##  ##  ##  ##  ##  ##  ##  ##  ##  ##  ##  ##  ##  ##  ##  ##  ##  ##  ##  ##  ##  ##  ##  ##  ##  ##  ##  ##  ##  ##  ##  ##  ##  ##  ##  ##  ##  ##  ##  ##  ##  ##  ##  ##  ##  ##  ##  ##  ##  ##  ##  ##  ## [2899_11]157	: ##  ##  ##  ##  ##  ##  ##  ##  ##  ##  ##  ##  ##  ##  ##  ##  ##  ##  ##  ##  ##  ##  ##  ##  ##  ##  ##  ##  ##  ##  ##  ##  ##  ##  ##  ##  ##  ##  ##  ##  ##  ##  ##  ##  ##  ##  ##  ##  ##  ##  ##  ##  ##  ##  ##  ##  ##  ##  ##  ##  ##  ##  ##  ##  ##  ##  ##  ##  ##  ##  ##  ##  ##  ##  ##  ##  ##  ##  ##  ##  ##  ##  ##  ##  ##  ##  ##  ##  ##  ##  ##  ##  ##  ##  ##  ##  ##  ##  ##  ##  ##  ##  ##  ##  ##  ##  ##  ##  ##  ##  ##  ##  ##  ##  ##  ##  ##  ##  ##  ##  ##  ##  ##  ##  ##  ##  ##  ##  ##  ##  ##  ##  ##  ##  ##  ##  ##  ##  ##  ##  ##  ##  ##  ##  ##  ##  ##  ##  ##  ##  ##  ##  ##  4  5  7  --  ##  ##  ##  ##  ##  ##  ##  ##  ##  ##  ##  ##  ##  ##  ##  ##  ##  ##  ##  ##  ##  ##  ##  ##  ##  ##  ##  ##  ##  ##  ##  ##  ##  ##  ##  ##  ##  ##  ##  ##  ##  ##  ##  ##  ##  ##  ##  ##  ##  ##  ##  ##  ##  ##  ##  ##  ##  ##  ##  ##  ##  ##  ##  ##  ##  ##  ##  ##  ##  ##  ##  ##  ##  ##  ##  ##  ##  ##  ##  ##  ##  ##  ##  ##  ##  ##  ##  ##  ##  ##  ##  ##  ##  ##  ##  ##  ##  ##  ##  ##  ##  ##  ##  ##  ##  ##  ##  ##  ##  ##  ##  ##  ## [1312_12]158	: ##  ##  ##  ##  ##  ##  ##  ##  ##  ##  ##  ##  ##  ##  ##  ##  ##  ##  ##  ##  ##  ##  ##  ##  ##  ##  ##  ##  ##  ##  ##  ##  ##  ##  ##  ##  ##  ##  ##  ##  ##  ##  ##  ##  ##  ##  ##  ##  ##  ##  ##  ##  ##  ##  ##  ##  ##  ##  ##  ##  ##  ##  ##  ##  ##  ##  ##  ##  ##  ##  ##  ##  ##  ##  ##  ##  ##  ##  ##  ##  ##  ##  ##  ##  ##  ##  ##  ##  ##  ##  ##  ##  ##  ##  ##  ##  ##  ##  ##  ##  ##  ##  ##  ##  ##  ##  ##  ##  ##  ##  ##  ##  ##  ##  ##  ##  ##  ##  ##  ##  ##  ##  ##  ##  ##  ##  ##  ##  ##  ##  ##  ##  ##  ##  ##  ##  ##  ##  ##  ##  ##  ##  ##  ##  ##  ##  ##  ##  ##  ##  ##  ##  ##  ##  ##  ##  ##  --  2  3  5  2  3  3  2  1  3  3  2  2  5  2  2  ##  ##  ##  ##  ##  ##  ##  ##  ##  ##  ##  ##  ##  ##  ##  ##  ##  ##  ##  ##  ##  ##  ##  ##  ##  ##  ##  ##  ##  ##  ##  ##  ##  ##  ##  ##  ##  ##  ##  ##  ##  ##  ##  ##  ##  ##  ##  ##  ##  ##  ##  ##  ##  ##  ##  ##  ##  ##  ##  ##  ##  ##  ##  ##  ##  ##  ##  ##  ##  ##  ##  ##  ##  ##  ##  ##  ##  ##  ##  ##  ##  ##  ##  ##  ##  ##  ##  ##  ##  ##  ##  ##  ##  ##  ##  ##  ## [2171_12]159	: ##  ##  ##  ##  ##  ##  ##  ##  ##  ##  ##  ##  ##  ##  ##  ##  ##  ##  ##  ##  ##  ##  ##  ##  ##  ##  ##  ##  ##  ##  ##  ##  ##  ##  ##  ##  ##  ##  ##  ##  ##  ##  ##  ##  ##  ##  ##  ##  ##  ##  ##  ##  ##  ##  ##  ##  ##  ##  ##  ##  ##  ##  ##  ##  ##  ##  ##  ##  ##  ##  ##  ##  ##  ##  ##  ##  ##  ##  ##  ##  ##  ##  ##  ##  ##  ##  ##  ##  ##  ##  ##  ##  ##  ##  ##  ##  ##  ##  ##  ##  ##  ##  ##  ##  ##  ##  ##  ##  ##  ##  ##  ##  ##  ##  ##  ##  ##  ##  ##  ##  ##  ##  ##  ##  ##  ##  ##  ##  ##  ##  ##  ##  ##  ##  ##  ##  ##  ##  ##  ##  ##  ##  ##  ##  ##  ##  ##  ##  ##  ##  ##  ##  ##  ##  ##  ##  ##  2  --  1  4  3  4  4  3  2  5  4  3  3  6  3  3  ##  ##  ##  ##  ##  ##  ##  ##  ##  ##  ##  ##  ##  ##  ##  ##  ##  ##  ##  ##  ##  ##  ##  ##  ##  ##  ##  ##  ##  ##  ##  ##  ##  ##  ##  ##  ##  ##  ##  ##  ##  ##  ##  ##  ##  ##  ##  ##  ##  ##  ##  ##  ##  ##  ##  ##  ##  ##  ##  ##  ##  ##  ##  ##  ##  ##  ##  ##  ##  ##  ##  ##  ##  ##  ##  ##  ##  ##  ##  ##  ##  ##  ##  ##  ##  ##  ##  ##  ##  ##  ##  ##  ##  ##  ##  ##  ## [2193_12]160	: ##  ##  ##  ##  ##  ##  ##  ##  ##  ##  ##  ##  ##  ##  ##  ##  ##  ##  ##  ##  ##  ##  ##  ##  ##  ##  ##  ##  ##  ##  ##  ##  ##  ##  ##  ##  ##  ##  ##  ##  ##  ##  ##  ##  ##  ##  ##  ##  ##  ##  ##  ##  ##  ##  ##  ##  ##  ##  ##  ##  ##  ##  ##  ##  ##  ##  ##  ##  ##  ##  ##  ##  ##  ##  ##  ##  ##  ##  ##  ##  ##  ##  ##  ##  ##  ##  ##  ##  ##  ##  ##  ##  ##  ##  ##  ##  ##  ##  ##  ##  ##  ##  ##  ##  ##  ##  ##  ##  ##  ##  ##  ##  ##  ##  ##  ##  ##  ##  ##  ##  ##  ##  ##  ##  ##  ##  ##  ##  ##  ##  ##  ##  ##  ##  ##  ##  ##  ##  ##  ##  ##  ##  ##  ##  ##  ##  ##  ##  ##  ##  ##  ##  ##  ##  ##  ##  ##  3  1  --  5  4  5  5  4  3  6  5  4  4  7  2  4  ##  ##  ##  ##  ##  ##  ##  ##  ##  ##  ##  ##  ##  ##  ##  ##  ##  ##  ##  ##  ##  ##  ##  ##  ##  ##  ##  ##  ##  ##  ##  ##  ##  ##  ##  ##  ##  ##  ##  ##  ##  ##  ##  ##  ##  ##  ##  ##  ##  ##  ##  ##  ##  ##  ##  ##  ##  ##  ##  ##  ##  ##  ##  ##  ##  ##  ##  ##  ##  ##  ##  ##  ##  ##  ##  ##  ##  ##  ##  ##  ##  ##  ##  ##  ##  ##  ##  ##  ##  ##  ##  ##  ##  ##  ##  ##  ## [2194_12]161	: ##  ##  ##  ##  ##  ##  ##  ##  ##  ##  ##  ##  ##  ##  ##  ##  ##  ##  ##  ##  ##  ##  ##  ##  ##  ##  ##  ##  ##  ##  ##  ##  ##  ##  ##  ##  ##  ##  ##  ##  ##  ##  ##  ##  ##  ##  ##  ##  ##  ##  ##  ##  ##  ##  ##  ##  ##  ##  ##  ##  ##  ##  ##  ##  ##  ##  ##  ##  ##  ##  ##  ##  ##  ##  ##  ##  ##  ##  ##  ##  ##  ##  ##  ##  ##  ##  ##  ##  ##  ##  ##  ##  ##  ##  ##  ##  ##  ##  ##  ##  ##  ##  ##  ##  ##  ##  ##  ##  ##  ##  ##  ##  ##  ##  ##  ##  ##  ##  ##  ##  ##  ##  ##  ##  ##  ##  ##  ##  ##  ##  ##  ##  ##  ##  ##  ##  ##  ##  ##  ##  ##  ##  ##  ##  ##  ##  ##  ##  ##  ##  ##  ##  ##  ##  ##  ##  ##  5  4  5  --  5  6  4  5  4  8  6  3  5  8  5  5  ##  ##  ##  ##  ##  ##  ##  ##  ##  ##  ##  ##  ##  ##  ##  ##  ##  ##  ##  ##  ##  ##  ##  ##  ##  ##  ##  ##  ##  ##  ##  ##  ##  ##  ##  ##  ##  ##  ##  ##  ##  ##  ##  ##  ##  ##  ##  ##  ##  ##  ##  ##  ##  ##  ##  ##  ##  ##  ##  ##  ##  ##  ##  ##  ##  ##  ##  ##  ##  ##  ##  ##  ##  ##  ##  ##  ##  ##  ##  ##  ##  ##  ##  ##  ##  ##  ##  ##  ##  ##  ##  ##  ##  ##  ##  ##  ## [2195_12]162	: ##  ##  ##  ##  ##  ##  ##  ##  ##  ##  ##  ##  ##  ##  ##  ##  ##  ##  ##  ##  ##  ##  ##  ##  ##  ##  ##  ##  ##  ##  ##  ##  ##  ##  ##  ##  ##  ##  ##  ##  ##  ##  ##  ##  ##  ##  ##  ##  ##  ##  ##  ##  ##  ##  ##  ##  ##  ##  ##  ##  ##  ##  ##  ##  ##  ##  ##  ##  ##  ##  ##  ##  ##  ##  ##  ##  ##  ##  ##  ##  ##  ##  ##  ##  ##  ##  ##  ##  ##  ##  ##  ##  ##  ##  ##  ##  ##  ##  ##  ##  ##  ##  ##  ##  ##  ##  ##  ##  ##  ##  ##  ##  ##  ##  ##  ##  ##  ##  ##  ##  ##  ##  ##  ##  ##  ##  ##  ##  ##  ##  ##  ##  ##  ##  ##  ##  ##  ##  ##  ##  ##  ##  ##  ##  ##  ##  ##  ##  ##  ##  ##  ##  ##  ##  ##  ##  ##  2  3  4  5  --  3  3  2  1  5  3  2  2  5  2  2  ##  ##  ##  ##  ##  ##  ##  ##  ##  ##  ##  ##  ##  ##  ##  ##  ##  ##  ##  ##  ##  ##  ##  ##  ##  ##  ##  ##  ##  ##  ##  ##  ##  ##  ##  ##  ##  ##  ##  ##  ##  ##  ##  ##  ##  ##  ##  ##  ##  ##  ##  ##  ##  ##  ##  ##  ##  ##  ##  ##  ##  ##  ##  ##  ##  ##  ##  ##  ##  ##  ##  ##  ##  ##  ##  ##  ##  ##  ##  ##  ##  ##  ##  ##  ##  ##  ##  ##  ##  ##  ##  ##  ##  ##  ##  ##  ## [2196_12]163	: ##  ##  ##  ##  ##  ##  ##  ##  ##  ##  ##  ##  ##  ##  ##  ##  ##  ##  ##  ##  ##  ##  ##  ##  ##  ##  ##  ##  ##  ##  ##  ##  ##  ##  ##  ##  ##  ##  ##  ##  ##  ##  ##  ##  ##  ##  ##  ##  ##  ##  ##  ##  ##  ##  ##  ##  ##  ##  ##  ##  ##  ##  ##  ##  ##  ##  ##  ##  ##  ##  ##  ##  ##  ##  ##  ##  ##  ##  ##  ##  ##  ##  ##  ##  ##  ##  ##  ##  ##  ##  ##  ##  ##  ##  ##  ##  ##  ##  ##  ##  ##  ##  ##  ##  ##  ##  ##  ##  ##  ##  ##  ##  ##  ##  ##  ##  ##  ##  ##  ##  ##  ##  ##  ##  ##  ##  ##  ##  ##  ##  ##  ##  ##  ##  ##  ##  ##  ##  ##  ##  ##  ##  ##  ##  ##  ##  ##  ##  ##  ##  ##  ##  ##  ##  ##  ##  ##  3  4  5  6  3  --  4  3  2  6  4  3  3  6  3  3  ##  ##  ##  ##  ##  ##  ##  ##  ##  ##  ##  ##  ##  ##  ##  ##  ##  ##  ##  ##  ##  ##  ##  ##  ##  ##  ##  ##  ##  ##  ##  ##  ##  ##  ##  ##  ##  ##  ##  ##  ##  ##  ##  ##  ##  ##  ##  ##  ##  ##  ##  ##  ##  ##  ##  ##  ##  ##  ##  ##  ##  ##  ##  ##  ##  ##  ##  ##  ##  ##  ##  ##  ##  ##  ##  ##  ##  ##  ##  ##  ##  ##  ##  ##  ##  ##  ##  ##  ##  ##  ##  ##  ##  ##  ##  ##  ## [2197_12]164	: ##  ##  ##  ##  ##  ##  ##  ##  ##  ##  ##  ##  ##  ##  ##  ##  ##  ##  ##  ##  ##  ##  ##  ##  ##  ##  ##  ##  ##  ##  ##  ##  ##  ##  ##  ##  ##  ##  ##  ##  ##  ##  ##  ##  ##  ##  ##  ##  ##  ##  ##  ##  ##  ##  ##  ##  ##  ##  ##  ##  ##  ##  ##  ##  ##  ##  ##  ##  ##  ##  ##  ##  ##  ##  ##  ##  ##  ##  ##  ##  ##  ##  ##  ##  ##  ##  ##  ##  ##  ##  ##  ##  ##  ##  ##  ##  ##  ##  ##  ##  ##  ##  ##  ##  ##  ##  ##  ##  ##  ##  ##  ##  ##  ##  ##  ##  ##  ##  ##  ##  ##  ##  ##  ##  ##  ##  ##  ##  ##  ##  ##  ##  ##  ##  ##  ##  ##  ##  ##  ##  ##  ##  ##  ##  ##  ##  ##  ##  ##  ##  ##  ##  ##  ##  ##  ##  ##  3  4  5  4  3  4  --  3  2  4  4  3  3  4  3  3  ##  ##  ##  ##  ##  ##  ##  ##  ##  ##  ##  ##  ##  ##  ##  ##  ##  ##  ##  ##  ##  ##  ##  ##  ##  ##  ##  ##  ##  ##  ##  ##  ##  ##  ##  ##  ##  ##  ##  ##  ##  ##  ##  ##  ##  ##  ##  ##  ##  ##  ##  ##  ##  ##  ##  ##  ##  ##  ##  ##  ##  ##  ##  ##  ##  ##  ##  ##  ##  ##  ##  ##  ##  ##  ##  ##  ##  ##  ##  ##  ##  ##  ##  ##  ##  ##  ##  ##  ##  ##  ##  ##  ##  ##  ##  ##  ## [2198_12]165	: ##  ##  ##  ##  ##  ##  ##  ##  ##  ##  ##  ##  ##  ##  ##  ##  ##  ##  ##  ##  ##  ##  ##  ##  ##  ##  ##  ##  ##  ##  ##  ##  ##  ##  ##  ##  ##  ##  ##  ##  ##  ##  ##  ##  ##  ##  ##  ##  ##  ##  ##  ##  ##  ##  ##  ##  ##  ##  ##  ##  ##  ##  ##  ##  ##  ##  ##  ##  ##  ##  ##  ##  ##  ##  ##  ##  ##  ##  ##  ##  ##  ##  ##  ##  ##  ##  ##  ##  ##  ##  ##  ##  ##  ##  ##  ##  ##  ##  ##  ##  ##  ##  ##  ##  ##  ##  ##  ##  ##  ##  ##  ##  ##  ##  ##  ##  ##  ##  ##  ##  ##  ##  ##  ##  ##  ##  ##  ##  ##  ##  ##  ##  ##  ##  ##  ##  ##  ##  ##  ##  ##  ##  ##  ##  ##  ##  ##  ##  ##  ##  ##  ##  ##  ##  ##  ##  ##  2  3  4  5  2  3  3  --  1  5  3  2  2  5  2  2  ##  ##  ##  ##  ##  ##  ##  ##  ##  ##  ##  ##  ##  ##  ##  ##  ##  ##  ##  ##  ##  ##  ##  ##  ##  ##  ##  ##  ##  ##  ##  ##  ##  ##  ##  ##  ##  ##  ##  ##  ##  ##  ##  ##  ##  ##  ##  ##  ##  ##  ##  ##  ##  ##  ##  ##  ##  ##  ##  ##  ##  ##  ##  ##  ##  ##  ##  ##  ##  ##  ##  ##  ##  ##  ##  ##  ##  ##  ##  ##  ##  ##  ##  ##  ##  ##  ##  ##  ##  ##  ##  ##  ##  ##  ##  ##  ## [2199_12]166	: ##  ##  ##  ##  ##  ##  ##  ##  ##  ##  ##  ##  ##  ##  ##  ##  ##  ##  ##  ##  ##  ##  ##  ##  ##  ##  ##  ##  ##  ##  ##  ##  ##  ##  ##  ##  ##  ##  ##  ##  ##  ##  ##  ##  ##  ##  ##  ##  ##  ##  ##  ##  ##  ##  ##  ##  ##  ##  ##  ##  ##  ##  ##  ##  ##  ##  ##  ##  ##  ##  ##  ##  ##  ##  ##  ##  ##  ##  ##  ##  ##  ##  ##  ##  ##  ##  ##  ##  ##  ##  ##  ##  ##  ##  ##  ##  ##  ##  ##  ##  ##  ##  ##  ##  ##  ##  ##  ##  ##  ##  ##  ##  ##  ##  ##  ##  ##  ##  ##  ##  ##  ##  ##  ##  ##  ##  ##  ##  ##  ##  ##  ##  ##  ##  ##  ##  ##  ##  ##  ##  ##  ##  ##  ##  ##  ##  ##  ##  ##  ##  ##  ##  ##  ##  ##  ##  ##  1  2  3  4  1  2  2  1  --  4  2  1  1  4  1  1  ##  ##  ##  ##  ##  ##  ##  ##  ##  ##  ##  ##  ##  ##  ##  ##  ##  ##  ##  ##  ##  ##  ##  ##  ##  ##  ##  ##  ##  ##  ##  ##  ##  ##  ##  ##  ##  ##  ##  ##  ##  ##  ##  ##  ##  ##  ##  ##  ##  ##  ##  ##  ##  ##  ##  ##  ##  ##  ##  ##  ##  ##  ##  ##  ##  ##  ##  ##  ##  ##  ##  ##  ##  ##  ##  ##  ##  ##  ##  ##  ##  ##  ##  ##  ##  ##  ##  ##  ##  ##  ##  ##  ##  ##  ##  ##  ## [2200_12]167	: ##  ##  ##  ##  ##  ##  ##  ##  ##  ##  ##  ##  ##  ##  ##  ##  ##  ##  ##  ##  ##  ##  ##  ##  ##  ##  ##  ##  ##  ##  ##  ##  ##  ##  ##  ##  ##  ##  ##  ##  ##  ##  ##  ##  ##  ##  ##  ##  ##  ##  ##  ##  ##  ##  ##  ##  ##  ##  ##  ##  ##  ##  ##  ##  ##  ##  ##  ##  ##  ##  ##  ##  ##  ##  ##  ##  ##  ##  ##  ##  ##  ##  ##  ##  ##  ##  ##  ##  ##  ##  ##  ##  ##  ##  ##  ##  ##  ##  ##  ##  ##  ##  ##  ##  ##  ##  ##  ##  ##  ##  ##  ##  ##  ##  ##  ##  ##  ##  ##  ##  ##  ##  ##  ##  ##  ##  ##  ##  ##  ##  ##  ##  ##  ##  ##  ##  ##  ##  ##  ##  ##  ##  ##  ##  ##  ##  ##  ##  ##  ##  ##  ##  ##  ##  ##  ##  ##  3  5  6  8  5  6  4  5  4  --  6  5  5  6  5  5  ##  ##  ##  ##  ##  ##  ##  ##  ##  ##  ##  ##  ##  ##  ##  ##  ##  ##  ##  ##  ##  ##  ##  ##  ##  ##  ##  ##  ##  ##  ##  ##  ##  ##  ##  ##  ##  ##  ##  ##  ##  ##  ##  ##  ##  ##  ##  ##  ##  ##  ##  ##  ##  ##  ##  ##  ##  ##  ##  ##  ##  ##  ##  ##  ##  ##  ##  ##  ##  ##  ##  ##  ##  ##  ##  ##  ##  ##  ##  ##  ##  ##  ##  ##  ##  ##  ##  ##  ##  ##  ##  ##  ##  ##  ##  ##  ## [2201_12]168	: ##  ##  ##  ##  ##  ##  ##  ##  ##  ##  ##  ##  ##  ##  ##  ##  ##  ##  ##  ##  ##  ##  ##  ##  ##  ##  ##  ##  ##  ##  ##  ##  ##  ##  ##  ##  ##  ##  ##  ##  ##  ##  ##  ##  ##  ##  ##  ##  ##  ##  ##  ##  ##  ##  ##  ##  ##  ##  ##  ##  ##  ##  ##  ##  ##  ##  ##  ##  ##  ##  ##  ##  ##  ##  ##  ##  ##  ##  ##  ##  ##  ##  ##  ##  ##  ##  ##  ##  ##  ##  ##  ##  ##  ##  ##  ##  ##  ##  ##  ##  ##  ##  ##  ##  ##  ##  ##  ##  ##  ##  ##  ##  ##  ##  ##  ##  ##  ##  ##  ##  ##  ##  ##  ##  ##  ##  ##  ##  ##  ##  ##  ##  ##  ##  ##  ##  ##  ##  ##  ##  ##  ##  ##  ##  ##  ##  ##  ##  ##  ##  ##  ##  ##  ##  ##  ##  ##  3  4  5  6  3  4  4  3  2  6  --  3  3  6  3  3  ##  ##  ##  ##  ##  ##  ##  ##  ##  ##  ##  ##  ##  ##  ##  ##  ##  ##  ##  ##  ##  ##  ##  ##  ##  ##  ##  ##  ##  ##  ##  ##  ##  ##  ##  ##  ##  ##  ##  ##  ##  ##  ##  ##  ##  ##  ##  ##  ##  ##  ##  ##  ##  ##  ##  ##  ##  ##  ##  ##  ##  ##  ##  ##  ##  ##  ##  ##  ##  ##  ##  ##  ##  ##  ##  ##  ##  ##  ##  ##  ##  ##  ##  ##  ##  ##  ##  ##  ##  ##  ##  ##  ##  ##  ##  ##  ## [2202_12]169	: ##  ##  ##  ##  ##  ##  ##  ##  ##  ##  ##  ##  ##  ##  ##  ##  ##  ##  ##  ##  ##  ##  ##  ##  ##  ##  ##  ##  ##  ##  ##  ##  ##  ##  ##  ##  ##  ##  ##  ##  ##  ##  ##  ##  ##  ##  ##  ##  ##  ##  ##  ##  ##  ##  ##  ##  ##  ##  ##  ##  ##  ##  ##  ##  ##  ##  ##  ##  ##  ##  ##  ##  ##  ##  ##  ##  ##  ##  ##  ##  ##  ##  ##  ##  ##  ##  ##  ##  ##  ##  ##  ##  ##  ##  ##  ##  ##  ##  ##  ##  ##  ##  ##  ##  ##  ##  ##  ##  ##  ##  ##  ##  ##  ##  ##  ##  ##  ##  ##  ##  ##  ##  ##  ##  ##  ##  ##  ##  ##  ##  ##  ##  ##  ##  ##  ##  ##  ##  ##  ##  ##  ##  ##  ##  ##  ##  ##  ##  ##  ##  ##  ##  ##  ##  ##  ##  ##  2  3  4  3  2  3  3  2  1  5  3  --  2  5  2  2  ##  ##  ##  ##  ##  ##  ##  ##  ##  ##  ##  ##  ##  ##  ##  ##  ##  ##  ##  ##  ##  ##  ##  ##  ##  ##  ##  ##  ##  ##  ##  ##  ##  ##  ##  ##  ##  ##  ##  ##  ##  ##  ##  ##  ##  ##  ##  ##  ##  ##  ##  ##  ##  ##  ##  ##  ##  ##  ##  ##  ##  ##  ##  ##  ##  ##  ##  ##  ##  ##  ##  ##  ##  ##  ##  ##  ##  ##  ##  ##  ##  ##  ##  ##  ##  ##  ##  ##  ##  ##  ##  ##  ##  ##  ##  ##  ## [2222_12]170	: ##  ##  ##  ##  ##  ##  ##  ##  ##  ##  ##  ##  ##  ##  ##  ##  ##  ##  ##  ##  ##  ##  ##  ##  ##  ##  ##  ##  ##  ##  ##  ##  ##  ##  ##  ##  ##  ##  ##  ##  ##  ##  ##  ##  ##  ##  ##  ##  ##  ##  ##  ##  ##  ##  ##  ##  ##  ##  ##  ##  ##  ##  ##  ##  ##  ##  ##  ##  ##  ##  ##  ##  ##  ##  ##  ##  ##  ##  ##  ##  ##  ##  ##  ##  ##  ##  ##  ##  ##  ##  ##  ##  ##  ##  ##  ##  ##  ##  ##  ##  ##  ##  ##  ##  ##  ##  ##  ##  ##  ##  ##  ##  ##  ##  ##  ##  ##  ##  ##  ##  ##  ##  ##  ##  ##  ##  ##  ##  ##  ##  ##  ##  ##  ##  ##  ##  ##  ##  ##  ##  ##  ##  ##  ##  ##  ##  ##  ##  ##  ##  ##  ##  ##  ##  ##  ##  ##  2  3  4  5  2  3  3  2  1  5  3  2  --  5  2  2  ##  ##  ##  ##  ##  ##  ##  ##  ##  ##  ##  ##  ##  ##  ##  ##  ##  ##  ##  ##  ##  ##  ##  ##  ##  ##  ##  ##  ##  ##  ##  ##  ##  ##  ##  ##  ##  ##  ##  ##  ##  ##  ##  ##  ##  ##  ##  ##  ##  ##  ##  ##  ##  ##  ##  ##  ##  ##  ##  ##  ##  ##  ##  ##  ##  ##  ##  ##  ##  ##  ##  ##  ##  ##  ##  ##  ##  ##  ##  ##  ##  ##  ##  ##  ##  ##  ##  ##  ##  ##  ##  ##  ##  ##  ##  ##  ## [2818_12]171	: ##  ##  ##  ##  ##  ##  ##  ##  ##  ##  ##  ##  ##  ##  ##  ##  ##  ##  ##  ##  ##  ##  ##  ##  ##  ##  ##  ##  ##  ##  ##  ##  ##  ##  ##  ##  ##  ##  ##  ##  ##  ##  ##  ##  ##  ##  ##  ##  ##  ##  ##  ##  ##  ##  ##  ##  ##  ##  ##  ##  ##  ##  ##  ##  ##  ##  ##  ##  ##  ##  ##  ##  ##  ##  ##  ##  ##  ##  ##  ##  ##  ##  ##  ##  ##  ##  ##  ##  ##  ##  ##  ##  ##  ##  ##  ##  ##  ##  ##  ##  ##  ##  ##  ##  ##  ##  ##  ##  ##  ##  ##  ##  ##  ##  ##  ##  ##  ##  ##  ##  ##  ##  ##  ##  ##  ##  ##  ##  ##  ##  ##  ##  ##  ##  ##  ##  ##  ##  ##  ##  ##  ##  ##  ##  ##  ##  ##  ##  ##  ##  ##  ##  ##  ##  ##  ##  ##  5  6  7  8  5  6  4  5  4  6  6  5  5  --  5  5  ##  ##  ##  ##  ##  ##  ##  ##  ##  ##  ##  ##  ##  ##  ##  ##  ##  ##  ##  ##  ##  ##  ##  ##  ##  ##  ##  ##  ##  ##  ##  ##  ##  ##  ##  ##  ##  ##  ##  ##  ##  ##  ##  ##  ##  ##  ##  ##  ##  ##  ##  ##  ##  ##  ##  ##  ##  ##  ##  ##  ##  ##  ##  ##  ##  ##  ##  ##  ##  ##  ##  ##  ##  ##  ##  ##  ##  ##  ##  ##  ##  ##  ##  ##  ##  ##  ##  ##  ##  ##  ##  ##  ##  ##  ##  ##  ## [2826_12]172	: ##  ##  ##  ##  ##  ##  ##  ##  ##  ##  ##  ##  ##  ##  ##  ##  ##  ##  ##  ##  ##  ##  ##  ##  ##  ##  ##  ##  ##  ##  ##  ##  ##  ##  ##  ##  ##  ##  ##  ##  ##  ##  ##  ##  ##  ##  ##  ##  ##  ##  ##  ##  ##  ##  ##  ##  ##  ##  ##  ##  ##  ##  ##  ##  ##  ##  ##  ##  ##  ##  ##  ##  ##  ##  ##  ##  ##  ##  ##  ##  ##  ##  ##  ##  ##  ##  ##  ##  ##  ##  ##  ##  ##  ##  ##  ##  ##  ##  ##  ##  ##  ##  ##  ##  ##  ##  ##  ##  ##  ##  ##  ##  ##  ##  ##  ##  ##  ##  ##  ##  ##  ##  ##  ##  ##  ##  ##  ##  ##  ##  ##  ##  ##  ##  ##  ##  ##  ##  ##  ##  ##  ##  ##  ##  ##  ##  ##  ##  ##  ##  ##  ##  ##  ##  ##  ##  ##  2  3  2  5  2  3  3  2  1  5  3  2  2  5  --  2  ##  ##  ##  ##  ##  ##  ##  ##  ##  ##  ##  ##  ##  ##  ##  ##  ##  ##  ##  ##  ##  ##  ##  ##  ##  ##  ##  ##  ##  ##  ##  ##  ##  ##  ##  ##  ##  ##  ##  ##  ##  ##  ##  ##  ##  ##  ##  ##  ##  ##  ##  ##  ##  ##  ##  ##  ##  ##  ##  ##  ##  ##  ##  ##  ##  ##  ##  ##  ##  ##  ##  ##  ##  ##  ##  ##  ##  ##  ##  ##  ##  ##  ##  ##  ##  ##  ##  ##  ##  ##  ##  ##  ##  ##  ##  ##  ## [2827_12]173	: ##  ##  ##  ##  ##  ##  ##  ##  ##  ##  ##  ##  ##  ##  ##  ##  ##  ##  ##  ##  ##  ##  ##  ##  ##  ##  ##  ##  ##  ##  ##  ##  ##  ##  ##  ##  ##  ##  ##  ##  ##  ##  ##  ##  ##  ##  ##  ##  ##  ##  ##  ##  ##  ##  ##  ##  ##  ##  ##  ##  ##  ##  ##  ##  ##  ##  ##  ##  ##  ##  ##  ##  ##  ##  ##  ##  ##  ##  ##  ##  ##  ##  ##  ##  ##  ##  ##  ##  ##  ##  ##  ##  ##  ##  ##  ##  ##  ##  ##  ##  ##  ##  ##  ##  ##  ##  ##  ##  ##  ##  ##  ##  ##  ##  ##  ##  ##  ##  ##  ##  ##  ##  ##  ##  ##  ##  ##  ##  ##  ##  ##  ##  ##  ##  ##  ##  ##  ##  ##  ##  ##  ##  ##  ##  ##  ##  ##  ##  ##  ##  ##  ##  ##  ##  ##  ##  ##  2  3  4  5  2  3  3  2  1  5  3  2  2  5  2  --  ##  ##  ##  ##  ##  ##  ##  ##  ##  ##  ##  ##  ##  ##  ##  ##  ##  ##  ##  ##  ##  ##  ##  ##  ##  ##  ##  ##  ##  ##  ##  ##  ##  ##  ##  ##  ##  ##  ##  ##  ##  ##  ##  ##  ##  ##  ##  ##  ##  ##  ##  ##  ##  ##  ##  ##  ##  ##  ##  ##  ##  ##  ##  ##  ##  ##  ##  ##  ##  ##  ##  ##  ##  ##  ##  ##  ##  ##  ##  ##  ##  ##  ##  ##  ##  ##  ##  ##  ##  ##  ##  ##  ##  ##  ##  ##  ## [1201_13]174	: ##  ##  ##  ##  ##  ##  ##  ##  ##  ##  ##  ##  ##  ##  ##  ##  ##  ##  ##  ##  ##  ##  ##  ##  ##  ##  ##  ##  ##  ##  ##  ##  ##  ##  ##  ##  ##  ##  ##  ##  ##  ##  ##  ##  ##  ##  ##  ##  ##  ##  ##  ##  ##  ##  ##  ##  ##  ##  ##  ##  ##  ##  ##  ##  ##  ##  ##  ##  ##  ##  ##  ##  ##  ##  ##  ##  ##  ##  ##  ##  ##  ##  ##  ##  ##  ##  ##  ##  ##  ##  ##  ##  ##  ##  ##  ##  ##  ##  ##  ##  ##  ##  ##  ##  ##  ##  ##  ##  ##  ##  ##  ##  ##  ##  ##  ##  ##  ##  ##  ##  ##  ##  ##  ##  ##  ##  ##  ##  ##  ##  ##  ##  ##  ##  ##  ##  ##  ##  ##  ##  ##  ##  ##  ##  ##  ##  ##  ##  ##  ##  ##  ##  ##  ##  ##  ##  ##  ##  ##  ##  ##  ##  ##  ##  ##  ##  ##  ##  ##  ##  ##  ##  ##  --  3  5  1  7  4  5  8  5  4  6  3  7  5  ##  ##  ##  ##  ##  ##  ##  ##  ##  ##  ##  ##  ##  ##  ##  ##  ##  ##  ##  ##  ##  ##  ##  ##  ##  ##  ##  ##  ##  ##  ##  ##  ##  ##  ##  ##  ##  ##  ##  ##  ##  ##  ##  ##  ##  ##  ##  ##  ##  ##  ##  ##  ##  ##  ##  ##  ##  ##  ##  ##  ##  ##  ##  ##  ##  ##  ##  ##  ##  ##  ##  ##  ##  ##  ##  ##  ##  ##  ##  ##  ##  ##  ## [1205_13]175	: ##  ##  ##  ##  ##  ##  ##  ##  ##  ##  ##  ##  ##  ##  ##  ##  ##  ##  ##  ##  ##  ##  ##  ##  ##  ##  ##  ##  ##  ##  ##  ##  ##  ##  ##  ##  ##  ##  ##  ##  ##  ##  ##  ##  ##  ##  ##  ##  ##  ##  ##  ##  ##  ##  ##  ##  ##  ##  ##  ##  ##  ##  ##  ##  ##  ##  ##  ##  ##  ##  ##  ##  ##  ##  ##  ##  ##  ##  ##  ##  ##  ##  ##  ##  ##  ##  ##  ##  ##  ##  ##  ##  ##  ##  ##  ##  ##  ##  ##  ##  ##  ##  ##  ##  ##  ##  ##  ##  ##  ##  ##  ##  ##  ##  ##  ##  ##  ##  ##  ##  ##  ##  ##  ##  ##  ##  ##  ##  ##  ##  ##  ##  ##  ##  ##  ##  ##  ##  ##  ##  ##  ##  ##  ##  ##  ##  ##  ##  ##  ##  ##  ##  ##  ##  ##  ##  ##  ##  ##  ##  ##  ##  ##  ##  ##  ##  ##  ##  ##  ##  ##  ##  ##  3  --  2  2  4  1  2  5  2  1  7  4  4  2  ##  ##  ##  ##  ##  ##  ##  ##  ##  ##  ##  ##  ##  ##  ##  ##  ##  ##  ##  ##  ##  ##  ##  ##  ##  ##  ##  ##  ##  ##  ##  ##  ##  ##  ##  ##  ##  ##  ##  ##  ##  ##  ##  ##  ##  ##  ##  ##  ##  ##  ##  ##  ##  ##  ##  ##  ##  ##  ##  ##  ##  ##  ##  ##  ##  ##  ##  ##  ##  ##  ##  ##  ##  ##  ##  ##  ##  ##  ##  ##  ##  ##  ## [1923_13]176	: ##  ##  ##  ##  ##  ##  ##  ##  ##  ##  ##  ##  ##  ##  ##  ##  ##  ##  ##  ##  ##  ##  ##  ##  ##  ##  ##  ##  ##  ##  ##  ##  ##  ##  ##  ##  ##  ##  ##  ##  ##  ##  ##  ##  ##  ##  ##  ##  ##  ##  ##  ##  ##  ##  ##  ##  ##  ##  ##  ##  ##  ##  ##  ##  ##  ##  ##  ##  ##  ##  ##  ##  ##  ##  ##  ##  ##  ##  ##  ##  ##  ##  ##  ##  ##  ##  ##  ##  ##  ##  ##  ##  ##  ##  ##  ##  ##  ##  ##  ##  ##  ##  ##  ##  ##  ##  ##  ##  ##  ##  ##  ##  ##  ##  ##  ##  ##  ##  ##  ##  ##  ##  ##  ##  ##  ##  ##  ##  ##  ##  ##  ##  ##  ##  ##  ##  ##  ##  ##  ##  ##  ##  ##  ##  ##  ##  ##  ##  ##  ##  ##  ##  ##  ##  ##  ##  ##  ##  ##  ##  ##  ##  ##  ##  ##  ##  ##  ##  ##  ##  ##  ##  ##  5  2  --  3  4  1  2  5  4  3  9  6  4  2  ##  ##  ##  ##  ##  ##  ##  ##  ##  ##  ##  ##  ##  ##  ##  ##  ##  ##  ##  ##  ##  ##  ##  ##  ##  ##  ##  ##  ##  ##  ##  ##  ##  ##  ##  ##  ##  ##  ##  ##  ##  ##  ##  ##  ##  ##  ##  ##  ##  ##  ##  ##  ##  ##  ##  ##  ##  ##  ##  ##  ##  ##  ##  ##  ##  ##  ##  ##  ##  ##  ##  ##  ##  ##  ##  ##  ##  ##  ##  ##  ##  ##  ## [1959_13]177	: ##  ##  ##  ##  ##  ##  ##  ##  ##  ##  ##  ##  ##  ##  ##  ##  ##  ##  ##  ##  ##  ##  ##  ##  ##  ##  ##  ##  ##  ##  ##  ##  ##  ##  ##  ##  ##  ##  ##  ##  ##  ##  ##  ##  ##  ##  ##  ##  ##  ##  ##  ##  ##  ##  ##  ##  ##  ##  ##  ##  ##  ##  ##  ##  ##  ##  ##  ##  ##  ##  ##  ##  ##  ##  ##  ##  ##  ##  ##  ##  ##  ##  ##  ##  ##  ##  ##  ##  ##  ##  ##  ##  ##  ##  ##  ##  ##  ##  ##  ##  ##  ##  ##  ##  ##  ##  ##  ##  ##  ##  ##  ##  ##  ##  ##  ##  ##  ##  ##  ##  ##  ##  ##  ##  ##  ##  ##  ##  ##  ##  ##  ##  ##  ##  ##  ##  ##  ##  ##  ##  ##  ##  ##  ##  ##  ##  ##  ##  ##  ##  ##  ##  ##  ##  ##  ##  ##  ##  ##  ##  ##  ##  ##  ##  ##  ##  ##  ##  ##  ##  ##  ##  ##  1  2  3  --  5  2  3  6  4  3  7  2  5  3  ##  ##  ##  ##  ##  ##  ##  ##  ##  ##  ##  ##  ##  ##  ##  ##  ##  ##  ##  ##  ##  ##  ##  ##  ##  ##  ##  ##  ##  ##  ##  ##  ##  ##  ##  ##  ##  ##  ##  ##  ##  ##  ##  ##  ##  ##  ##  ##  ##  ##  ##  ##  ##  ##  ##  ##  ##  ##  ##  ##  ##  ##  ##  ##  ##  ##  ##  ##  ##  ##  ##  ##  ##  ##  ##  ##  ##  ##  ##  ##  ##  ##  ## [2183_13]178	: ##  ##  ##  ##  ##  ##  ##  ##  ##  ##  ##  ##  ##  ##  ##  ##  ##  ##  ##  ##  ##  ##  ##  ##  ##  ##  ##  ##  ##  ##  ##  ##  ##  ##  ##  ##  ##  ##  ##  ##  ##  ##  ##  ##  ##  ##  ##  ##  ##  ##  ##  ##  ##  ##  ##  ##  ##  ##  ##  ##  ##  ##  ##  ##  ##  ##  ##  ##  ##  ##  ##  ##  ##  ##  ##  ##  ##  ##  ##  ##  ##  ##  ##  ##  ##  ##  ##  ##  ##  ##  ##  ##  ##  ##  ##  ##  ##  ##  ##  ##  ##  ##  ##  ##  ##  ##  ##  ##  ##  ##  ##  ##  ##  ##  ##  ##  ##  ##  ##  ##  ##  ##  ##  ##  ##  ##  ##  ##  ##  ##  ##  ##  ##  ##  ##  ##  ##  ##  ##  ##  ##  ##  ##  ##  ##  ##  ##  ##  ##  ##  ##  ##  ##  ##  ##  ##  ##  ##  ##  ##  ##  ##  ##  ##  ##  ##  ##  ##  ##  ##  ##  ##  ##  7  4  4  5  --  3  4  5  6  5  11  8  4  2  ##  ##  ##  ##  ##  ##  ##  ##  ##  ##  ##  ##  ##  ##  ##  ##  ##  ##  ##  ##  ##  ##  ##  ##  ##  ##  ##  ##  ##  ##  ##  ##  ##  ##  ##  ##  ##  ##  ##  ##  ##  ##  ##  ##  ##  ##  ##  ##  ##  ##  ##  ##  ##  ##  ##  ##  ##  ##  ##  ##  ##  ##  ##  ##  ##  ##  ##  ##  ##  ##  ##  ##  ##  ##  ##  ##  ##  ##  ##  ##  ##  ##  ## [2184_13]179	: ##  ##  ##  ##  ##  ##  ##  ##  ##  ##  ##  ##  ##  ##  ##  ##  ##  ##  ##  ##  ##  ##  ##  ##  ##  ##  ##  ##  ##  ##  ##  ##  ##  ##  ##  ##  ##  ##  ##  ##  ##  ##  ##  ##  ##  ##  ##  ##  ##  ##  ##  ##  ##  ##  ##  ##  ##  ##  ##  ##  ##  ##  ##  ##  ##  ##  ##  ##  ##  ##  ##  ##  ##  ##  ##  ##  ##  ##  ##  ##  ##  ##  ##  ##  ##  ##  ##  ##  ##  ##  ##  ##  ##  ##  ##  ##  ##  ##  ##  ##  ##  ##  ##  ##  ##  ##  ##  ##  ##  ##  ##  ##  ##  ##  ##  ##  ##  ##  ##  ##  ##  ##  ##  ##  ##  ##  ##  ##  ##  ##  ##  ##  ##  ##  ##  ##  ##  ##  ##  ##  ##  ##  ##  ##  ##  ##  ##  ##  ##  ##  ##  ##  ##  ##  ##  ##  ##  ##  ##  ##  ##  ##  ##  ##  ##  ##  ##  ##  ##  ##  ##  ##  ##  4  1  1  2  3  --  1  4  3  2  8  5  3  1  ##  ##  ##  ##  ##  ##  ##  ##  ##  ##  ##  ##  ##  ##  ##  ##  ##  ##  ##  ##  ##  ##  ##  ##  ##  ##  ##  ##  ##  ##  ##  ##  ##  ##  ##  ##  ##  ##  ##  ##  ##  ##  ##  ##  ##  ##  ##  ##  ##  ##  ##  ##  ##  ##  ##  ##  ##  ##  ##  ##  ##  ##  ##  ##  ##  ##  ##  ##  ##  ##  ##  ##  ##  ##  ##  ##  ##  ##  ##  ##  ##  ##  ## [2215_13]180	: ##  ##  ##  ##  ##  ##  ##  ##  ##  ##  ##  ##  ##  ##  ##  ##  ##  ##  ##  ##  ##  ##  ##  ##  ##  ##  ##  ##  ##  ##  ##  ##  ##  ##  ##  ##  ##  ##  ##  ##  ##  ##  ##  ##  ##  ##  ##  ##  ##  ##  ##  ##  ##  ##  ##  ##  ##  ##  ##  ##  ##  ##  ##  ##  ##  ##  ##  ##  ##  ##  ##  ##  ##  ##  ##  ##  ##  ##  ##  ##  ##  ##  ##  ##  ##  ##  ##  ##  ##  ##  ##  ##  ##  ##  ##  ##  ##  ##  ##  ##  ##  ##  ##  ##  ##  ##  ##  ##  ##  ##  ##  ##  ##  ##  ##  ##  ##  ##  ##  ##  ##  ##  ##  ##  ##  ##  ##  ##  ##  ##  ##  ##  ##  ##  ##  ##  ##  ##  ##  ##  ##  ##  ##  ##  ##  ##  ##  ##  ##  ##  ##  ##  ##  ##  ##  ##  ##  ##  ##  ##  ##  ##  ##  ##  ##  ##  ##  ##  ##  ##  ##  ##  ##  5  2  2  3  4  1  --  5  4  3  9  6  4  2  ##  ##  ##  ##  ##  ##  ##  ##  ##  ##  ##  ##  ##  ##  ##  ##  ##  ##  ##  ##  ##  ##  ##  ##  ##  ##  ##  ##  ##  ##  ##  ##  ##  ##  ##  ##  ##  ##  ##  ##  ##  ##  ##  ##  ##  ##  ##  ##  ##  ##  ##  ##  ##  ##  ##  ##  ##  ##  ##  ##  ##  ##  ##  ##  ##  ##  ##  ##  ##  ##  ##  ##  ##  ##  ##  ##  ##  ##  ##  ##  ##  ##  ## [2337_13]181	: ##  ##  ##  ##  ##  ##  ##  ##  ##  ##  ##  ##  ##  ##  ##  ##  ##  ##  ##  ##  ##  ##  ##  ##  ##  ##  ##  ##  ##  ##  ##  ##  ##  ##  ##  ##  ##  ##  ##  ##  ##  ##  ##  ##  ##  ##  ##  ##  ##  ##  ##  ##  ##  ##  ##  ##  ##  ##  ##  ##  ##  ##  ##  ##  ##  ##  ##  ##  ##  ##  ##  ##  ##  ##  ##  ##  ##  ##  ##  ##  ##  ##  ##  ##  ##  ##  ##  ##  ##  ##  ##  ##  ##  ##  ##  ##  ##  ##  ##  ##  ##  ##  ##  ##  ##  ##  ##  ##  ##  ##  ##  ##  ##  ##  ##  ##  ##  ##  ##  ##  ##  ##  ##  ##  ##  ##  ##  ##  ##  ##  ##  ##  ##  ##  ##  ##  ##  ##  ##  ##  ##  ##  ##  ##  ##  ##  ##  ##  ##  ##  ##  ##  ##  ##  ##  ##  ##  ##  ##  ##  ##  ##  ##  ##  ##  ##  ##  ##  ##  ##  ##  ##  ##  8  5  5  6  5  4  5  --  5  6  12  9  7  5  ##  ##  ##  ##  ##  ##  ##  ##  ##  ##  ##  ##  ##  ##  ##  ##  ##  ##  ##  ##  ##  ##  ##  ##  ##  ##  ##  ##  ##  ##  ##  ##  ##  ##  ##  ##  ##  ##  ##  ##  ##  ##  ##  ##  ##  ##  ##  ##  ##  ##  ##  ##  ##  ##  ##  ##  ##  ##  ##  ##  ##  ##  ##  ##  ##  ##  ##  ##  ##  ##  ##  ##  ##  ##  ##  ##  ##  ##  ##  ##  ##  ##  ## [2458_13]182	: ##  ##  ##  ##  ##  ##  ##  ##  ##  ##  ##  ##  ##  ##  ##  ##  ##  ##  ##  ##  ##  ##  ##  ##  ##  ##  ##  ##  ##  ##  ##  ##  ##  ##  ##  ##  ##  ##  ##  ##  ##  ##  ##  ##  ##  ##  ##  ##  ##  ##  ##  ##  ##  ##  ##  ##  ##  ##  ##  ##  ##  ##  ##  ##  ##  ##  ##  ##  ##  ##  ##  ##  ##  ##  ##  ##  ##  ##  ##  ##  ##  ##  ##  ##  ##  ##  ##  ##  ##  ##  ##  ##  ##  ##  ##  ##  ##  ##  ##  ##  ##  ##  ##  ##  ##  ##  ##  ##  ##  ##  ##  ##  ##  ##  ##  ##  ##  ##  ##  ##  ##  ##  ##  ##  ##  ##  ##  ##  ##  ##  ##  ##  ##  ##  ##  ##  ##  ##  ##  ##  ##  ##  ##  ##  ##  ##  ##  ##  ##  ##  ##  ##  ##  ##  ##  ##  ##  ##  ##  ##  ##  ##  ##  ##  ##  ##  ##  ##  ##  ##  ##  ##  ##  5  2  4  4  6  3  4  5  --  3  9  6  6  4  ##  ##  ##  ##  ##  ##  ##  ##  ##  ##  ##  ##  ##  ##  ##  ##  ##  ##  ##  ##  ##  ##  ##  ##  ##  ##  ##  ##  ##  ##  ##  ##  ##  ##  ##  ##  ##  ##  ##  ##  ##  ##  ##  ##  ##  ##  ##  ##  ##  ##  ##  ##  ##  ##  ##  ##  ##  ##  ##  ##  ##  ##  ##  ##  ##  ##  ##  ##  ##  ##  ##  ##  ##  ##  ##  ##  ##  ##  ##  ##  ##  ##  ## [2475_13]183	: ##  ##  ##  ##  ##  ##  ##  ##  ##  ##  ##  ##  ##  ##  ##  ##  ##  ##  ##  ##  ##  ##  ##  ##  ##  ##  ##  ##  ##  ##  ##  ##  ##  ##  ##  ##  ##  ##  ##  ##  ##  ##  ##  ##  ##  ##  ##  ##  ##  ##  ##  ##  ##  ##  ##  ##  ##  ##  ##  ##  ##  ##  ##  ##  ##  ##  ##  ##  ##  ##  ##  ##  ##  ##  ##  ##  ##  ##  ##  ##  ##  ##  ##  ##  ##  ##  ##  ##  ##  ##  ##  ##  ##  ##  ##  ##  ##  ##  ##  ##  ##  ##  ##  ##  ##  ##  ##  ##  ##  ##  ##  ##  ##  ##  ##  ##  ##  ##  ##  ##  ##  ##  ##  ##  ##  ##  ##  ##  ##  ##  ##  ##  ##  ##  ##  ##  ##  ##  ##  ##  ##  ##  ##  ##  ##  ##  ##  ##  ##  ##  ##  ##  ##  ##  ##  ##  ##  ##  ##  ##  ##  ##  ##  ##  ##  ##  ##  ##  ##  ##  ##  ##  ##  4  1  3  3  5  2  3  6  3  --  8  5  5  3  ##  ##  ##  ##  ##  ##  ##  ##  ##  ##  ##  ##  ##  ##  ##  ##  ##  ##  ##  ##  ##  ##  ##  ##  ##  ##  ##  ##  ##  ##  ##  ##  ##  ##  ##  ##  ##  ##  ##  ##  ##  ##  ##  ##  ##  ##  ##  ##  ##  ##  ##  ##  ##  ##  ##  ##  ##  ##  ##  ##  ##  ##  ##  ##  ##  ##  ##  ##  ##  ##  ##  ##  ##  ##  ##  ##  ##  ##  ##  ##  ##  ##  ## [2921_13]184	: ##  ##  ##  ##  ##  ##  ##  ##  ##  ##  ##  ##  ##  ##  ##  ##  ##  ##  ##  ##  ##  ##  ##  ##  ##  ##  ##  ##  ##  ##  ##  ##  ##  ##  ##  ##  ##  ##  ##  ##  ##  ##  ##  ##  ##  ##  ##  ##  ##  ##  ##  ##  ##  ##  ##  ##  ##  ##  ##  ##  ##  ##  ##  ##  ##  ##  ##  ##  ##  ##  ##  ##  ##  ##  ##  ##  ##  ##  ##  ##  ##  ##  ##  ##  ##  ##  ##  ##  ##  ##  ##  ##  ##  ##  ##  ##  ##  ##  ##  ##  ##  ##  ##  ##  ##  ##  ##  ##  ##  ##  ##  ##  ##  ##  ##  ##  ##  ##  ##  ##  ##  ##  ##  ##  ##  ##  ##  ##  ##  ##  ##  ##  ##  ##  ##  ##  ##  ##  ##  ##  ##  ##  ##  ##  ##  ##  ##  ##  ##  ##  ##  ##  ##  ##  ##  ##  ##  ##  ##  ##  ##  ##  ##  ##  ##  ##  ##  ##  ##  ##  ##  ##  ##  6  7  9  7  11  8  9  12  9  8  --  9  9  9  ##  ##  ##  ##  ##  ##  ##  ##  ##  ##  ##  ##  ##  ##  ##  ##  ##  ##  ##  ##  ##  ##  ##  ##  ##  ##  ##  ##  ##  ##  ##  ##  ##  ##  ##  ##  ##  ##  ##  ##  ##  ##  ##  ##  ##  ##  ##  ##  ##  ##  ##  ##  ##  ##  ##  ##  ##  ##  ##  ##  ##  ##  ##  ##  ##  ##  ##  ##  ##  ##  ##  ##  ##  ##  ##  ##  ##  ##  ##  ##  ##  ##  ## [2038_13]185	: ##  ##  ##  ##  ##  ##  ##  ##  ##  ##  ##  ##  ##  ##  ##  ##  ##  ##  ##  ##  ##  ##  ##  ##  ##  ##  ##  ##  ##  ##  ##  ##  ##  ##  ##  ##  ##  ##  ##  ##  ##  ##  ##  ##  ##  ##  ##  ##  ##  ##  ##  ##  ##  ##  ##  ##  ##  ##  ##  ##  ##  ##  ##  ##  ##  ##  ##  ##  ##  ##  ##  ##  ##  ##  ##  ##  ##  ##  ##  ##  ##  ##  ##  ##  ##  ##  ##  ##  ##  ##  ##  ##  ##  ##  ##  ##  ##  ##  ##  ##  ##  ##  ##  ##  ##  ##  ##  ##  ##  ##  ##  ##  ##  ##  ##  ##  ##  ##  ##  ##  ##  ##  ##  ##  ##  ##  ##  ##  ##  ##  ##  ##  ##  ##  ##  ##  ##  ##  ##  ##  ##  ##  ##  ##  ##  ##  ##  ##  ##  ##  ##  ##  ##  ##  ##  ##  ##  ##  ##  ##  ##  ##  ##  ##  ##  ##  ##  ##  ##  ##  ##  ##  ##  3  4  6  2  8  5  6  9  6  5  9  --  8  6  ##  ##  ##  ##  ##  ##  ##  ##  ##  ##  ##  ##  ##  ##  ##  ##  ##  ##  ##  ##  ##  ##  ##  ##  ##  ##  ##  ##  ##  ##  ##  ##  ##  ##  ##  ##  ##  ##  ##  ##  ##  ##  ##  ##  ##  ##  ##  ##  ##  ##  ##  ##  ##  ##  ##  ##  ##  ##  ##  ##  ##  ##  ##  ##  ##  ##  ##  ##  ##  ##  ##  ##  ##  ##  ##  ##  ##  ##  ##  ##  ##  ##  ## [1986_13]186	: ##  ##  ##  ##  ##  ##  ##  ##  ##  ##  ##  ##  ##  ##  ##  ##  ##  ##  ##  ##  ##  ##  ##  ##  ##  ##  ##  ##  ##  ##  ##  ##  ##  ##  ##  ##  ##  ##  ##  ##  ##  ##  ##  ##  ##  ##  ##  ##  ##  ##  ##  ##  ##  ##  ##  ##  ##  ##  ##  ##  ##  ##  ##  ##  ##  ##  ##  ##  ##  ##  ##  ##  ##  ##  ##  ##  ##  ##  ##  ##  ##  ##  ##  ##  ##  ##  ##  ##  ##  ##  ##  ##  ##  ##  ##  ##  ##  ##  ##  ##  ##  ##  ##  ##  ##  ##  ##  ##  ##  ##  ##  ##  ##  ##  ##  ##  ##  ##  ##  ##  ##  ##  ##  ##  ##  ##  ##  ##  ##  ##  ##  ##  ##  ##  ##  ##  ##  ##  ##  ##  ##  ##  ##  ##  ##  ##  ##  ##  ##  ##  ##  ##  ##  ##  ##  ##  ##  ##  ##  ##  ##  ##  ##  ##  ##  ##  ##  ##  ##  ##  ##  ##  ##  7  4  4  5  4  3  4  7  6  5  9  8  --  2  ##  ##  ##  ##  ##  ##  ##  ##  ##  ##  ##  ##  ##  ##  ##  ##  ##  ##  ##  ##  ##  ##  ##  ##  ##  ##  ##  ##  ##  ##  ##  ##  ##  ##  ##  ##  ##  ##  ##  ##  ##  ##  ##  ##  ##  ##  ##  ##  ##  ##  ##  ##  ##  ##  ##  ##  ##  ##  ##  ##  ##  ##  ##  ##  ##  ##  ##  ##  ##  ##  ##  ##  ##  ##  ##  ##  ##  ##  ##  ##  ##  ##  ## [1998_13]187	: ##  ##  ##  ##  ##  ##  ##  ##  ##  ##  ##  ##  ##  ##  ##  ##  ##  ##  ##  ##  ##  ##  ##  ##  ##  ##  ##  ##  ##  ##  ##  ##  ##  ##  ##  ##  ##  ##  ##  ##  ##  ##  ##  ##  ##  ##  ##  ##  ##  ##  ##  ##  ##  ##  ##  ##  ##  ##  ##  ##  ##  ##  ##  ##  ##  ##  ##  ##  ##  ##  ##  ##  ##  ##  ##  ##  ##  ##  ##  ##  ##  ##  ##  ##  ##  ##  ##  ##  ##  ##  ##  ##  ##  ##  ##  ##  ##  ##  ##  ##  ##  ##  ##  ##  ##  ##  ##  ##  ##  ##  ##  ##  ##  ##  ##  ##  ##  ##  ##  ##  ##  ##  ##  ##  ##  ##  ##  ##  ##  ##  ##  ##  ##  ##  ##  ##  ##  ##  ##  ##  ##  ##  ##  ##  ##  ##  ##  ##  ##  ##  ##  ##  ##  ##  ##  ##  ##  ##  ##  ##  ##  ##  ##  ##  ##  ##  ##  ##  ##  ##  ##  ##  ##  5  2  2  3  2  1  2  5  4  3  9  6  2  --  ##  ##  ##  ##  ##  ##  ##  ##  ##  ##  ##  ##  ##  ##  ##  ##  ##  ##  ##  ##  ##  ##  ##  ##  ##  ##  ##  ##  ##  ##  ##  ##  ##  ##  ##  ##  ##  ##  ##  ##  ##  ##  ##  ##  ##  ##  ##  ##  ##  ##  ##  ##  ##  ##  ##  ##  ##  ##  ##  ##  ##  ##  ##  ##  ##  ##  ##  ##  ##  ##  ##  ##  ##  ##  ##  ##  ##  ##  ##  ##  ##  ##  ## [2312_18]188	: ##  ##  ##  ##  ##  ##  ##  ##  ##  ##  ##  ##  ##  ##  ##  ##  ##  ##  ##  ##  ##  ##  ##  ##  ##  ##  ##  ##  ##  ##  ##  ##  ##  ##  ##  ##  ##  ##  ##  ##  ##  ##  ##  ##  ##  ##  ##  ##  ##  ##  ##  ##  ##  ##  ##  ##  ##  ##  ##  ##  ##  ##  ##  ##  ##  ##  ##  ##  ##  ##  ##  ##  ##  ##  ##  ##  ##  ##  ##  ##  ##  ##  ##  ##  ##  ##  ##  ##  ##  ##  ##  ##  ##  ##  ##  ##  ##  ##  ##  ##  ##  ##  ##  ##  ##  ##  ##  ##  ##  ##  ##  ##  ##  ##  ##  ##  ##  ##  ##  ##  ##  ##  ##  ##  ##  ##  ##  ##  ##  ##  ##  ##  ##  ##  ##  ##  ##  ##  ##  ##  ##  ##  ##  ##  ##  ##  ##  ##  ##  ##  ##  ##  ##  ##  ##  ##  ##  ##  ##  ##  ##  ##  ##  ##  ##  ##  ##  ##  ##  ##  ##  ##  ##  ##  ##  ##  ##  ##  ##  ##  ##  ##  ##  ##  ##  ##  ##  --  4  3  ##  ##  ##  ##  ##  ##  ##  ##  ##  ##  ##  ##  ##  ##  ##  ##  ##  ##  ##  ##  ##  ##  ##  ##  ##  ##  ##  ##  ##  ##  ##  ##  ##  ##  ##  ##  ##  ##  ##  ##  ##  ##  ##  ##  ##  ##  ##  ##  ##  ##  ##  ##  ##  ##  ##  ##  ##  ##  ##  ##  ##  ##  ##  ##  ##  ##  ##  ##  ##  ##  ##  ##  ##  ##  ##  ##  ##  ##  ##  ## [2313_18]189	: ##  ##  ##  ##  ##  ##  ##  ##  ##  ##  ##  ##  ##  ##  ##  ##  ##  ##  ##  ##  ##  ##  ##  ##  ##  ##  ##  ##  ##  ##  ##  ##  ##  ##  ##  ##  ##  ##  ##  ##  ##  ##  ##  ##  ##  ##  ##  ##  ##  ##  ##  ##  ##  ##  ##  ##  ##  ##  ##  ##  ##  ##  ##  ##  ##  ##  ##  ##  ##  ##  ##  ##  ##  ##  ##  ##  ##  ##  ##  ##  ##  ##  ##  ##  ##  ##  ##  ##  ##  ##  ##  ##  ##  ##  ##  ##  ##  ##  ##  ##  ##  ##  ##  ##  ##  ##  ##  ##  ##  ##  ##  ##  ##  ##  ##  ##  ##  ##  ##  ##  ##  ##  ##  ##  ##  ##  ##  ##  ##  ##  ##  ##  ##  ##  ##  ##  ##  ##  ##  ##  ##  ##  ##  ##  ##  ##  ##  ##  ##  ##  ##  ##  ##  ##  ##  ##  ##  ##  ##  ##  ##  ##  ##  ##  ##  ##  ##  ##  ##  ##  ##  ##  ##  ##  ##  ##  ##  ##  ##  ##  ##  ##  ##  ##  ##  ##  ##  4  --  3  ##  ##  ##  ##  ##  ##  ##  ##  ##  ##  ##  ##  ##  ##  ##  ##  ##  ##  ##  ##  ##  ##  ##  ##  ##  ##  ##  ##  ##  ##  ##  ##  ##  ##  ##  ##  ##  ##  ##  ##  ##  ##  ##  ##  ##  ##  ##  ##  ##  ##  ##  ##  ##  ##  ##  ##  ##  ##  ##  ##  ##  ##  ##  ##  ##  ##  ##  ##  ##  ##  ##  ##  ##  ##  ##  ##  ##  ##  ##  ## [2314_18]190	: ##  ##  ##  ##  ##  ##  ##  ##  ##  ##  ##  ##  ##  ##  ##  ##  ##  ##  ##  ##  ##  ##  ##  ##  ##  ##  ##  ##  ##  ##  ##  ##  ##  ##  ##  ##  ##  ##  ##  ##  ##  ##  ##  ##  ##  ##  ##  ##  ##  ##  ##  ##  ##  ##  ##  ##  ##  ##  ##  ##  ##  ##  ##  ##  ##  ##  ##  ##  ##  ##  ##  ##  ##  ##  ##  ##  ##  ##  ##  ##  ##  ##  ##  ##  ##  ##  ##  ##  ##  ##  ##  ##  ##  ##  ##  ##  ##  ##  ##  ##  ##  ##  ##  ##  ##  ##  ##  ##  ##  ##  ##  ##  ##  ##  ##  ##  ##  ##  ##  ##  ##  ##  ##  ##  ##  ##  ##  ##  ##  ##  ##  ##  ##  ##  ##  ##  ##  ##  ##  ##  ##  ##  ##  ##  ##  ##  ##  ##  ##  ##  ##  ##  ##  ##  ##  ##  ##  ##  ##  ##  ##  ##  ##  ##  ##  ##  ##  ##  ##  ##  ##  ##  ##  ##  ##  ##  ##  ##  ##  ##  ##  ##  ##  ##  ##  ##  ##  3  3  --  ##  ##  ##  ##  ##  ##  ##  ##  ##  ##  ##  ##  ##  ##  ##  ##  ##  ##  ##  ##  ##  ##  ##  ##  ##  ##  ##  ##  ##  ##  ##  ##  ##  ##  ##  ##  ##  ##  ##  ##  ##  ##  ##  ##  ##  ##  ##  ##  ##  ##  ##  ##  ##  ##  ##  ##  ##  ##  ##  ##  ##  ##  ##  ##  ##  ##  ##  ##  ##  ##  ##  ##  ##  ##  ##  ##  ##  ##  ##  ## [2302_20]191	: ##  ##  ##  ##  ##  ##  ##  ##  ##  ##  ##  ##  ##  ##  ##  ##  ##  ##  ##  ##  ##  ##  ##  ##  ##  ##  ##  ##  ##  ##  ##  ##  ##  ##  ##  ##  ##  ##  ##  ##  ##  ##  ##  ##  ##  ##  ##  ##  ##  ##  ##  ##  ##  ##  ##  ##  ##  ##  ##  ##  ##  ##  ##  ##  ##  ##  ##  ##  ##  ##  ##  ##  ##  ##  ##  ##  ##  ##  ##  ##  ##  ##  ##  ##  ##  ##  ##  ##  ##  ##  ##  ##  ##  ##  ##  ##  ##  ##  ##  ##  ##  ##  ##  ##  ##  ##  ##  ##  ##  ##  ##  ##  ##  ##  ##  ##  ##  ##  ##  ##  ##  ##  ##  ##  ##  ##  ##  ##  ##  ##  ##  ##  ##  ##  ##  ##  ##  ##  ##  ##  ##  ##  ##  ##  ##  ##  ##  ##  ##  ##  ##  ##  ##  ##  ##  ##  ##  ##  ##  ##  ##  ##  ##  ##  ##  ##  ##  ##  ##  ##  ##  ##  ##  ##  ##  ##  ##  ##  ##  ##  ##  ##  ##  ##  ##  ##  ##  ##  ##  ##  --  ##  4  ##  ##  ##  ##  ##  ##  ##  ##  ##  ##  ##  ##  ##  ##  ##  ##  ##  ##  ##  ##  ##  ##  ##  ##  ##  ##  ##  ##  ##  ##  ##  ##  ##  ##  ##  ##  ##  ##  ##  ##  ##  ##  ##  ##  ##  ##  ##  ##  ##  ##  ##  ##  ##  ##  ##  ##  ##  ##  ##  ##  ##  ##  ##  ##  ##  ##  ##  ##  ##  ##  ##  ##  ##  ##  ##  ##  ## [2324_20]192	: ##  ##  ##  ##  ##  ##  ##  ##  ##  ##  ##  ##  ##  ##  ##  ##  ##  ##  ##  ##  ##  ##  ##  ##  ##  ##  ##  ##  ##  ##  ##  ##  ##  ##  ##  ##  ##  ##  ##  ##  ##  ##  ##  ##  ##  ##  ##  ##  ##  ##  ##  ##  ##  ##  ##  ##  ##  ##  ##  ##  ##  ##  ##  ##  ##  ##  ##  ##  ##  ##  ##  ##  ##  ##  ##  ##  ##  ##  ##  ##  ##  ##  ##  ##  ##  ##  ##  ##  ##  ##  ##  ##  ##  ##  ##  ##  ##  ##  ##  ##  ##  ##  ##  ##  ##  ##  ##  ##  ##  ##  ##  ##  ##  ##  ##  ##  ##  ##  ##  ##  ##  ##  ##  ##  ##  ##  ##  ##  ##  ##  ##  ##  ##  ##  ##  ##  ##  ##  ##  ##  ##  ##  ##  ##  ##  ##  ##  ##  ##  ##  ##  ##  ##  ##  ##  ##  ##  ##  ##  ##  ##  ##  ##  ##  ##  ##  ##  ##  ##  ##  ##  ##  ##  ##  ##  ##  ##  ##  ##  ##  ##  ##  ##  ##  ##  ##  ##  ##  ##  ##  ##  --  ##  5  ##  ##  ##  ##  ##  ##  ##  ##  ##  ##  ##  ##  ##  ##  ##  ##  ##  ##  ##  ##  ##  ##  ##  ##  ##  ##  ##  ##  ##  ##  ##  ##  ##  ##  ##  ##  ##  ##  ##  ##  ##  ##  ##  ##  ##  ##  ##  ##  ##  ##  ##  ##  ##  ##  ##  ##  ##  ##  ##  ##  ##  ##  ##  ##  ##  ##  ##  ##  ##  ##  ##  ##  ##  ##  ##  ## [2349_20]193	: ##  ##  ##  ##  ##  ##  ##  ##  ##  ##  ##  ##  ##  ##  ##  ##  ##  ##  ##  ##  ##  ##  ##  ##  ##  ##  ##  ##  ##  ##  ##  ##  ##  ##  ##  ##  ##  ##  ##  ##  ##  ##  ##  ##  ##  ##  ##  ##  ##  ##  ##  ##  ##  ##  ##  ##  ##  ##  ##  ##  ##  ##  ##  ##  ##  ##  ##  ##  ##  ##  ##  ##  ##  ##  ##  ##  ##  ##  ##  ##  ##  ##  ##  ##  ##  ##  ##  ##  ##  ##  ##  ##  ##  ##  ##  ##  ##  ##  ##  ##  ##  ##  ##  ##  ##  ##  ##  ##  ##  ##  ##  ##  ##  ##  ##  ##  ##  ##  ##  ##  ##  ##  ##  ##  ##  ##  ##  ##  ##  ##  ##  ##  ##  ##  ##  ##  ##  ##  ##  ##  ##  ##  ##  ##  ##  ##  ##  ##  ##  ##  ##  ##  ##  ##  ##  ##  ##  ##  ##  ##  ##  ##  ##  ##  ##  ##  ##  ##  ##  ##  ##  ##  ##  ##  ##  ##  ##  ##  ##  ##  ##  ##  ##  ##  ##  ##  ##  ##  ##  ##  4  ##  --  ##  ##  ##  ##  ##  ##  ##  ##  ##  ##  ##  ##  ##  ##  ##  ##  ##  ##  ##  ##  ##  ##  ##  ##  ##  ##  ##  ##  ##  ##  ##  ##  ##  ##  ##  ##  ##  ##  ##  ##  ##  ##  ##  ##  ##  ##  ##  ##  ##  ##  ##  ##  ##  ##  ##  ##  ##  ##  ##  ##  ##  ##  ##  ##  ##  ##  ##  ##  ##  ##  ##  ##  ##  ##  ##  ##  ## [2875_20]194	: ##  ##  ##  ##  ##  ##  ##  ##  ##  ##  ##  ##  ##  ##  ##  ##  ##  ##  ##  ##  ##  ##  ##  ##  ##  ##  ##  ##  ##  ##  ##  ##  ##  ##  ##  ##  ##  ##  ##  ##  ##  ##  ##  ##  ##  ##  ##  ##  ##  ##  ##  ##  ##  ##  ##  ##  ##  ##  ##  ##  ##  ##  ##  ##  ##  ##  ##  ##  ##  ##  ##  ##  ##  ##  ##  ##  ##  ##  ##  ##  ##  ##  ##  ##  ##  ##  ##  ##  ##  ##  ##  ##  ##  ##  ##  ##  ##  ##  ##  ##  ##  ##  ##  ##  ##  ##  ##  ##  ##  ##  ##  ##  ##  ##  ##  ##  ##  ##  ##  ##  ##  ##  ##  ##  ##  ##  ##  ##  ##  ##  ##  ##  ##  ##  ##  ##  ##  ##  ##  ##  ##  ##  ##  ##  ##  ##  ##  ##  ##  ##  ##  ##  ##  ##  ##  ##  ##  ##  ##  ##  ##  ##  ##  ##  ##  ##  ##  ##  ##  ##  ##  ##  ##  ##  ##  ##  ##  ##  ##  ##  ##  ##  ##  ##  ##  ##  ##  ##  ##  ##  ##  5  ##  --  ##  ##  ##  ##  ##  ##  ##  ##  ##  ##  ##  ##  ##  ##  ##  ##  ##  ##  ##  ##  ##  ##  ##  ##  ##  ##  ##  ##  ##  ##  ##  ##  ##  ##  ##  ##  ##  ##  ##  ##  ##  ##  ##  ##  ##  ##  ##  ##  ##  ##  ##  ##  ##  ##  ##  ##  ##  ##  ##  ##  ##  ##  ##  ##  ##  ##  ##  ##  ##  ##  ##  ##  ##  ##  ##  ## [2342_21]195	: ##  ##  ##  ##  ##  ##  ##  ##  ##  ##  ##  ##  ##  ##  ##  ##  ##  ##  ##  ##  ##  ##  ##  ##  ##  ##  ##  ##  ##  ##  ##  ##  ##  ##  ##  ##  ##  ##  ##  ##  ##  ##  ##  ##  ##  ##  ##  ##  ##  ##  ##  ##  ##  ##  ##  ##  ##  ##  ##  ##  ##  ##  ##  ##  ##  ##  ##  ##  ##  ##  ##  ##  ##  ##  ##  ##  ##  ##  ##  ##  ##  ##  ##  ##  ##  ##  ##  ##  ##  ##  ##  ##  ##  ##  ##  ##  ##  ##  ##  ##  ##  ##  ##  ##  ##  ##  ##  ##  ##  ##  ##  ##  ##  ##  ##  ##  ##  ##  ##  ##  ##  ##  ##  ##  ##  ##  ##  ##  ##  ##  ##  ##  ##  ##  ##  ##  ##  ##  ##  ##  ##  ##  ##  ##  ##  ##  ##  ##  ##  ##  ##  ##  ##  ##  ##  ##  ##  ##  ##  ##  ##  ##  ##  ##  ##  ##  ##  ##  ##  ##  ##  ##  ##  ##  ##  ##  ##  ##  ##  ##  ##  ##  ##  ##  ##  ##  ##  ##  ##  ##  ##  ##  ##  ##  --  2  ##  ##  ##  ##  ##  ##  ##  ##  ##  ##  ##  ##  ##  ##  ##  ##  ##  ##  ##  ##  ##  ##  ##  ##  ##  ##  ##  ##  ##  ##  ##  ##  ##  ##  ##  ##  ##  ##  ##  ##  ##  ##  ##  ##  ##  ##  ##  ##  ##  ##  ##  ##  ##  ##  ##  ##  ##  ##  ##  ##  ##  ##  ##  ##  ##  ##  ##  ##  ##  ##  ##  ##  ##  ## [2894_21]196	: ##  ##  ##  ##  ##  ##  ##  ##  ##  ##  ##  ##  ##  ##  ##  ##  ##  ##  ##  ##  ##  ##  ##  ##  ##  ##  ##  ##  ##  ##  ##  ##  ##  ##  ##  ##  ##  ##  ##  ##  ##  ##  ##  ##  ##  ##  ##  ##  ##  ##  ##  ##  ##  ##  ##  ##  ##  ##  ##  ##  ##  ##  ##  ##  ##  ##  ##  ##  ##  ##  ##  ##  ##  ##  ##  ##  ##  ##  ##  ##  ##  ##  ##  ##  ##  ##  ##  ##  ##  ##  ##  ##  ##  ##  ##  ##  ##  ##  ##  ##  ##  ##  ##  ##  ##  ##  ##  ##  ##  ##  ##  ##  ##  ##  ##  ##  ##  ##  ##  ##  ##  ##  ##  ##  ##  ##  ##  ##  ##  ##  ##  ##  ##  ##  ##  ##  ##  ##  ##  ##  ##  ##  ##  ##  ##  ##  ##  ##  ##  ##  ##  ##  ##  ##  ##  ##  ##  ##  ##  ##  ##  ##  ##  ##  ##  ##  ##  ##  ##  ##  ##  ##  ##  ##  ##  ##  ##  ##  ##  ##  ##  ##  ##  ##  ##  ##  ##  ##  ##  ##  ##  ##  ##  ##  2  --  ##  ##  ##  ##  ##  ##  ##  ##  ##  ##  ##  ##  ##  ##  ##  ##  ##  ##  ##  ##  ##  ##  ##  ##  ##  ##  ##  ##  ##  ##  ##  ##  ##  ##  ##  ##  ##  ##  ##  ##  ##  ##  ##  ##  ##  ##  ##  ##  ##  ##  ##  ##  ##  ##  ##  ##  ##  ##  ##  ##  ##  ##  ##  ##  ##  ##  ##  ##  ##  ##  ##  ##  ##  ## [2842_14]197	: ##  ##  ##  ##  ##  ##  ##  ##  ##  ##  ##  ##  ##  ##  ##  ##  ##  ##  ##  ##  ##  ##  ##  ##  ##  ##  ##  ##  ##  ##  ##  ##  ##  ##  ##  ##  ##  ##  ##  ##  ##  ##  ##  ##  ##  ##  ##  ##  ##  ##  ##  ##  ##  ##  ##  ##  ##  ##  ##  ##  ##  ##  ##  ##  ##  ##  ##  ##  ##  ##  ##  ##  ##  ##  ##  ##  ##  ##  ##  ##  ##  ##  ##  ##  ##  ##  ##  ##  ##  ##  ##  ##  ##  ##  ##  ##  ##  ##  ##  ##  ##  ##  ##  ##  ##  ##  ##  ##  ##  ##  ##  ##  ##  ##  ##  ##  ##  ##  ##  ##  ##  ##  ##  ##  ##  ##  ##  ##  ##  ##  ##  ##  ##  ##  ##  ##  ##  ##  ##  ##  ##  ##  ##  ##  ##  ##  ##  ##  ##  ##  ##  ##  ##  ##  ##  ##  ##  ##  ##  ##  ##  ##  ##  ##  ##  ##  ##  ##  ##  ##  ##  ##  ##  ##  ##  ##  ##  ##  ##  ##  ##  ##  ##  ##  ##  ##  ##  ##  ##  ##  ##  ##  ##  ##  ##  ##  --  1  1  1  1  ##  ##  ##  ##  ##  ##  ##  ##  ##  ##  ##  ##  ##  ##  ##  ##  ##  ##  ##  ##  ##  ##  ##  ##  ##  ##  ##  ##  ##  ##  ##  ##  ##  ##  ##  ##  ##  ##  ##  ##  ##  ##  ##  ##  ##  ##  ##  ##  ##  ##  ##  ##  ##  ##  ##  ##  ##  ##  ##  ##  ##  ##  ##  ##  ##  ##  ##  ##  ## [2843_14]198	: ##  ##  ##  ##  ##  ##  ##  ##  ##  ##  ##  ##  ##  ##  ##  ##  ##  ##  ##  ##  ##  ##  ##  ##  ##  ##  ##  ##  ##  ##  ##  ##  ##  ##  ##  ##  ##  ##  ##  ##  ##  ##  ##  ##  ##  ##  ##  ##  ##  ##  ##  ##  ##  ##  ##  ##  ##  ##  ##  ##  ##  ##  ##  ##  ##  ##  ##  ##  ##  ##  ##  ##  ##  ##  ##  ##  ##  ##  ##  ##  ##  ##  ##  ##  ##  ##  ##  ##  ##  ##  ##  ##  ##  ##  ##  ##  ##  ##  ##  ##  ##  ##  ##  ##  ##  ##  ##  ##  ##  ##  ##  ##  ##  ##  ##  ##  ##  ##  ##  ##  ##  ##  ##  ##  ##  ##  ##  ##  ##  ##  ##  ##  ##  ##  ##  ##  ##  ##  ##  ##  ##  ##  ##  ##  ##  ##  ##  ##  ##  ##  ##  ##  ##  ##  ##  ##  ##  ##  ##  ##  ##  ##  ##  ##  ##  ##  ##  ##  ##  ##  ##  ##  ##  ##  ##  ##  ##  ##  ##  ##  ##  ##  ##  ##  ##  ##  ##  ##  ##  ##  ##  ##  ##  ##  ##  ##  1  --  2  2  2  ##  ##  ##  ##  ##  ##  ##  ##  ##  ##  ##  ##  ##  ##  ##  ##  ##  ##  ##  ##  ##  ##  ##  ##  ##  ##  ##  ##  ##  ##  ##  ##  ##  ##  ##  ##  ##  ##  ##  ##  ##  ##  ##  ##  ##  ##  ##  ##  ##  ##  ##  ##  ##  ##  ##  ##  ##  ##  ##  ##  ##  ##  ##  ##  ##  ##  ##  ##  ## [2847_14]199	: ##  ##  ##  ##  ##  ##  ##  ##  ##  ##  ##  ##  ##  ##  ##  ##  ##  ##  ##  ##  ##  ##  ##  ##  ##  ##  ##  ##  ##  ##  ##  ##  ##  ##  ##  ##  ##  ##  ##  ##  ##  ##  ##  ##  ##  ##  ##  ##  ##  ##  ##  ##  ##  ##  ##  ##  ##  ##  ##  ##  ##  ##  ##  ##  ##  ##  ##  ##  ##  ##  ##  ##  ##  ##  ##  ##  ##  ##  ##  ##  ##  ##  ##  ##  ##  ##  ##  ##  ##  ##  ##  ##  ##  ##  ##  ##  ##  ##  ##  ##  ##  ##  ##  ##  ##  ##  ##  ##  ##  ##  ##  ##  ##  ##  ##  ##  ##  ##  ##  ##  ##  ##  ##  ##  ##  ##  ##  ##  ##  ##  ##  ##  ##  ##  ##  ##  ##  ##  ##  ##  ##  ##  ##  ##  ##  ##  ##  ##  ##  ##  ##  ##  ##  ##  ##  ##  ##  ##  ##  ##  ##  ##  ##  ##  ##  ##  ##  ##  ##  ##  ##  ##  ##  ##  ##  ##  ##  ##  ##  ##  ##  ##  ##  ##  ##  ##  ##  ##  ##  ##  ##  ##  ##  ##  ##  ##  1  2  --  2  2  ##  ##  ##  ##  ##  ##  ##  ##  ##  ##  ##  ##  ##  ##  ##  ##  ##  ##  ##  ##  ##  ##  ##  ##  ##  ##  ##  ##  ##  ##  ##  ##  ##  ##  ##  ##  ##  ##  ##  ##  ##  ##  ##  ##  ##  ##  ##  ##  ##  ##  ##  ##  ##  ##  ##  ##  ##  ##  ##  ##  ##  ##  ##  ##  ##  ##  ##  ##  ## [2852_14]200	: ##  ##  ##  ##  ##  ##  ##  ##  ##  ##  ##  ##  ##  ##  ##  ##  ##  ##  ##  ##  ##  ##  ##  ##  ##  ##  ##  ##  ##  ##  ##  ##  ##  ##  ##  ##  ##  ##  ##  ##  ##  ##  ##  ##  ##  ##  ##  ##  ##  ##  ##  ##  ##  ##  ##  ##  ##  ##  ##  ##  ##  ##  ##  ##  ##  ##  ##  ##  ##  ##  ##  ##  ##  ##  ##  ##  ##  ##  ##  ##  ##  ##  ##  ##  ##  ##  ##  ##  ##  ##  ##  ##  ##  ##  ##  ##  ##  ##  ##  ##  ##  ##  ##  ##  ##  ##  ##  ##  ##  ##  ##  ##  ##  ##  ##  ##  ##  ##  ##  ##  ##  ##  ##  ##  ##  ##  ##  ##  ##  ##  ##  ##  ##  ##  ##  ##  ##  ##  ##  ##  ##  ##  ##  ##  ##  ##  ##  ##  ##  ##  ##  ##  ##  ##  ##  ##  ##  ##  ##  ##  ##  ##  ##  ##  ##  ##  ##  ##  ##  ##  ##  ##  ##  ##  ##  ##  ##  ##  ##  ##  ##  ##  ##  ##  ##  ##  ##  ##  ##  ##  ##  ##  ##  ##  ##  ##  1  2  2  --  2  ##  ##  ##  ##  ##  ##  ##  ##  ##  ##  ##  ##  ##  ##  ##  ##  ##  ##  ##  ##  ##  ##  ##  ##  ##  ##  ##  ##  ##  ##  ##  ##  ##  ##  ##  ##  ##  ##  ##  ##  ##  ##  ##  ##  ##  ##  ##  ##  ##  ##  ##  ##  ##  ##  ##  ##  ##  ##  ##  ##  ##  ##  ##  ##  ##  ##  ##  ##  ## [2479_14]201	: ##  ##  ##  ##  ##  ##  ##  ##  ##  ##  ##  ##  ##  ##  ##  ##  ##  ##  ##  ##  ##  ##  ##  ##  ##  ##  ##  ##  ##  ##  ##  ##  ##  ##  ##  ##  ##  ##  ##  ##  ##  ##  ##  ##  ##  ##  ##  ##  ##  ##  ##  ##  ##  ##  ##  ##  ##  ##  ##  ##  ##  ##  ##  ##  ##  ##  ##  ##  ##  ##  ##  ##  ##  ##  ##  ##  ##  ##  ##  ##  ##  ##  ##  ##  ##  ##  ##  ##  ##  ##  ##  ##  ##  ##  ##  ##  ##  ##  ##  ##  ##  ##  ##  ##  ##  ##  ##  ##  ##  ##  ##  ##  ##  ##  ##  ##  ##  ##  ##  ##  ##  ##  ##  ##  ##  ##  ##  ##  ##  ##  ##  ##  ##  ##  ##  ##  ##  ##  ##  ##  ##  ##  ##  ##  ##  ##  ##  ##  ##  ##  ##  ##  ##  ##  ##  ##  ##  ##  ##  ##  ##  ##  ##  ##  ##  ##  ##  ##  ##  ##  ##  ##  ##  ##  ##  ##  ##  ##  ##  ##  ##  ##  ##  ##  ##  ##  ##  ##  ##  ##  ##  ##  ##  ##  ##  ##  1  2  2  2  --  ##  ##  ##  ##  ##  ##  ##  ##  ##  ##  ##  ##  ##  ##  ##  ##  ##  ##  ##  ##  ##  ##  ##  ##  ##  ##  ##  ##  ##  ##  ##  ##  ##  ##  ##  ##  ##  ##  ##  ##  ##  ##  ##  ##  ##  ##  ##  ##  ##  ##  ##  ##  ##  ##  ##  ##  ##  ##  ##  ##  ##  ##  ##  ##  ##  ##  ##  ##  ## [2226_4]202	: ##  ##  ##  ##  ##  ##  ##  ##  ##  ##  ##  ##  ##  ##  ##  ##  ##  ##  ##  ##  ##  ##  ##  ##  ##  ##  ##  ##  ##  ##  ##  ##  ##  ##  ##  ##  ##  ##  ##  ##  ##  ##  ##  ##  ##  ##  ##  ##  ##  ##  ##  ##  ##  ##  ##  ##  ##  ##  ##  ##  ##  ##  ##  ##  ##  ##  ##  ##  ##  ##  ##  ##  ##  ##  ##  ##  ##  ##  ##  ##  ##  ##  ##  ##  ##  ##  ##  ##  ##  ##  ##  ##  ##  ##  ##  ##  ##  ##  ##  ##  ##  ##  ##  ##  ##  ##  ##  ##  ##  ##  ##  ##  ##  ##  ##  ##  ##  ##  ##  ##  ##  ##  ##  ##  ##  ##  ##  ##  ##  ##  ##  ##  ##  ##  ##  ##  ##  ##  ##  ##  ##  ##  ##  ##  ##  ##  ##  ##  ##  ##  ##  ##  ##  ##  ##  ##  ##  ##  ##  ##  ##  ##  ##  ##  ##  ##  ##  ##  ##  ##  ##  ##  ##  ##  ##  ##  ##  ##  ##  ##  ##  ##  ##  ##  ##  ##  ##  ##  ##  ##  ##  ##  ##  ##  ##  ##  ##  ##  ##  ##  ##  --  4  1  2  5  6  2  ##  ##  ##  ##  ##  ##  ##  ##  ##  ##  ##  ##  ##  ##  ##  ##  ##  ##  ##  ##  ##  ##  ##  ##  ##  ##  ##  ##  ##  ##  ##  ##  ##  ##  ##  ##  ##  ##  ##  ##  ##  ##  ##  ##  ##  ##  ##  ##  ##  ##  ##  ##  ##  ##  ##  ##  ##  ##  ##  ##  ##  ## [2227_4]203	: ##  ##  ##  ##  ##  ##  ##  ##  ##  ##  ##  ##  ##  ##  ##  ##  ##  ##  ##  ##  ##  ##  ##  ##  ##  ##  ##  ##  ##  ##  ##  ##  ##  ##  ##  ##  ##  ##  ##  ##  ##  ##  ##  ##  ##  ##  ##  ##  ##  ##  ##  ##  ##  ##  ##  ##  ##  ##  ##  ##  ##  ##  ##  ##  ##  ##  ##  ##  ##  ##  ##  ##  ##  ##  ##  ##  ##  ##  ##  ##  ##  ##  ##  ##  ##  ##  ##  ##  ##  ##  ##  ##  ##  ##  ##  ##  ##  ##  ##  ##  ##  ##  ##  ##  ##  ##  ##  ##  ##  ##  ##  ##  ##  ##  ##  ##  ##  ##  ##  ##  ##  ##  ##  ##  ##  ##  ##  ##  ##  ##  ##  ##  ##  ##  ##  ##  ##  ##  ##  ##  ##  ##  ##  ##  ##  ##  ##  ##  ##  ##  ##  ##  ##  ##  ##  ##  ##  ##  ##  ##  ##  ##  ##  ##  ##  ##  ##  ##  ##  ##  ##  ##  ##  ##  ##  ##  ##  ##  ##  ##  ##  ##  ##  ##  ##  ##  ##  ##  ##  ##  ##  ##  ##  ##  ##  ##  ##  ##  ##  ##  ##  4  --  3  4  1  2  4  ##  ##  ##  ##  ##  ##  ##  ##  ##  ##  ##  ##  ##  ##  ##  ##  ##  ##  ##  ##  ##  ##  ##  ##  ##  ##  ##  ##  ##  ##  ##  ##  ##  ##  ##  ##  ##  ##  ##  ##  ##  ##  ##  ##  ##  ##  ##  ##  ##  ##  ##  ##  ##  ##  ##  ##  ##  ##  ##  ##  ##  ## [2228_4]204	: ##  ##  ##  ##  ##  ##  ##  ##  ##  ##  ##  ##  ##  ##  ##  ##  ##  ##  ##  ##  ##  ##  ##  ##  ##  ##  ##  ##  ##  ##  ##  ##  ##  ##  ##  ##  ##  ##  ##  ##  ##  ##  ##  ##  ##  ##  ##  ##  ##  ##  ##  ##  ##  ##  ##  ##  ##  ##  ##  ##  ##  ##  ##  ##  ##  ##  ##  ##  ##  ##  ##  ##  ##  ##  ##  ##  ##  ##  ##  ##  ##  ##  ##  ##  ##  ##  ##  ##  ##  ##  ##  ##  ##  ##  ##  ##  ##  ##  ##  ##  ##  ##  ##  ##  ##  ##  ##  ##  ##  ##  ##  ##  ##  ##  ##  ##  ##  ##  ##  ##  ##  ##  ##  ##  ##  ##  ##  ##  ##  ##  ##  ##  ##  ##  ##  ##  ##  ##  ##  ##  ##  ##  ##  ##  ##  ##  ##  ##  ##  ##  ##  ##  ##  ##  ##  ##  ##  ##  ##  ##  ##  ##  ##  ##  ##  ##  ##  ##  ##  ##  ##  ##  ##  ##  ##  ##  ##  ##  ##  ##  ##  ##  ##  ##  ##  ##  ##  ##  ##  ##  ##  ##  ##  ##  ##  ##  ##  ##  ##  ##  ##  1  3  --  1  4  5  1  ##  ##  ##  ##  ##  ##  ##  ##  ##  ##  ##  ##  ##  ##  ##  ##  ##  ##  ##  ##  ##  ##  ##  ##  ##  ##  ##  ##  ##  ##  ##  ##  ##  ##  ##  ##  ##  ##  ##  ##  ##  ##  ##  ##  ##  ##  ##  ##  ##  ##  ##  ##  ##  ##  ##  ##  ##  ##  ##  ##  ##  ## [2229_4]205	: ##  ##  ##  ##  ##  ##  ##  ##  ##  ##  ##  ##  ##  ##  ##  ##  ##  ##  ##  ##  ##  ##  ##  ##  ##  ##  ##  ##  ##  ##  ##  ##  ##  ##  ##  ##  ##  ##  ##  ##  ##  ##  ##  ##  ##  ##  ##  ##  ##  ##  ##  ##  ##  ##  ##  ##  ##  ##  ##  ##  ##  ##  ##  ##  ##  ##  ##  ##  ##  ##  ##  ##  ##  ##  ##  ##  ##  ##  ##  ##  ##  ##  ##  ##  ##  ##  ##  ##  ##  ##  ##  ##  ##  ##  ##  ##  ##  ##  ##  ##  ##  ##  ##  ##  ##  ##  ##  ##  ##  ##  ##  ##  ##  ##  ##  ##  ##  ##  ##  ##  ##  ##  ##  ##  ##  ##  ##  ##  ##  ##  ##  ##  ##  ##  ##  ##  ##  ##  ##  ##  ##  ##  ##  ##  ##  ##  ##  ##  ##  ##  ##  ##  ##  ##  ##  ##  ##  ##  ##  ##  ##  ##  ##  ##  ##  ##  ##  ##  ##  ##  ##  ##  ##  ##  ##  ##  ##  ##  ##  ##  ##  ##  ##  ##  ##  ##  ##  ##  ##  ##  ##  ##  ##  ##  ##  ##  ##  ##  ##  ##  ##  2  4  1  --  5  6  2  ##  ##  ##  ##  ##  ##  ##  ##  ##  ##  ##  ##  ##  ##  ##  ##  ##  ##  ##  ##  ##  ##  ##  ##  ##  ##  ##  ##  ##  ##  ##  ##  ##  ##  ##  ##  ##  ##  ##  ##  ##  ##  ##  ##  ##  ##  ##  ##  ##  ##  ##  ##  ##  ##  ##  ##  ##  ##  ##  ##  ##  ## [2234_4]206	: ##  ##  ##  ##  ##  ##  ##  ##  ##  ##  ##  ##  ##  ##  ##  ##  ##  ##  ##  ##  ##  ##  ##  ##  ##  ##  ##  ##  ##  ##  ##  ##  ##  ##  ##  ##  ##  ##  ##  ##  ##  ##  ##  ##  ##  ##  ##  ##  ##  ##  ##  ##  ##  ##  ##  ##  ##  ##  ##  ##  ##  ##  ##  ##  ##  ##  ##  ##  ##  ##  ##  ##  ##  ##  ##  ##  ##  ##  ##  ##  ##  ##  ##  ##  ##  ##  ##  ##  ##  ##  ##  ##  ##  ##  ##  ##  ##  ##  ##  ##  ##  ##  ##  ##  ##  ##  ##  ##  ##  ##  ##  ##  ##  ##  ##  ##  ##  ##  ##  ##  ##  ##  ##  ##  ##  ##  ##  ##  ##  ##  ##  ##  ##  ##  ##  ##  ##  ##  ##  ##  ##  ##  ##  ##  ##  ##  ##  ##  ##  ##  ##  ##  ##  ##  ##  ##  ##  ##  ##  ##  ##  ##  ##  ##  ##  ##  ##  ##  ##  ##  ##  ##  ##  ##  ##  ##  ##  ##  ##  ##  ##  ##  ##  ##  ##  ##  ##  ##  ##  ##  ##  ##  ##  ##  ##  ##  ##  ##  ##  ##  ##  5  1  4  5  --  3  5  ##  ##  ##  ##  ##  ##  ##  ##  ##  ##  ##  ##  ##  ##  ##  ##  ##  ##  ##  ##  ##  ##  ##  ##  ##  ##  ##  ##  ##  ##  ##  ##  ##  ##  ##  ##  ##  ##  ##  ##  ##  ##  ##  ##  ##  ##  ##  ##  ##  ##  ##  ##  ##  ##  ##  ##  ##  ##  ##  ##  ##  ## [2045_4]207	: ##  ##  ##  ##  ##  ##  ##  ##  ##  ##  ##  ##  ##  ##  ##  ##  ##  ##  ##  ##  ##  ##  ##  ##  ##  ##  ##  ##  ##  ##  ##  ##  ##  ##  ##  ##  ##  ##  ##  ##  ##  ##  ##  ##  ##  ##  ##  ##  ##  ##  ##  ##  ##  ##  ##  ##  ##  ##  ##  ##  ##  ##  ##  ##  ##  ##  ##  ##  ##  ##  ##  ##  ##  ##  ##  ##  ##  ##  ##  ##  ##  ##  ##  ##  ##  ##  ##  ##  ##  ##  ##  ##  ##  ##  ##  ##  ##  ##  ##  ##  ##  ##  ##  ##  ##  ##  ##  ##  ##  ##  ##  ##  ##  ##  ##  ##  ##  ##  ##  ##  ##  ##  ##  ##  ##  ##  ##  ##  ##  ##  ##  ##  ##  ##  ##  ##  ##  ##  ##  ##  ##  ##  ##  ##  ##  ##  ##  ##  ##  ##  ##  ##  ##  ##  ##  ##  ##  ##  ##  ##  ##  ##  ##  ##  ##  ##  ##  ##  ##  ##  ##  ##  ##  ##  ##  ##  ##  ##  ##  ##  ##  ##  ##  ##  ##  ##  ##  ##  ##  ##  ##  ##  ##  ##  ##  ##  ##  ##  ##  ##  ##  6  2  5  6  3  --  6  ##  ##  ##  ##  ##  ##  ##  ##  ##  ##  ##  ##  ##  ##  ##  ##  ##  ##  ##  ##  ##  ##  ##  ##  ##  ##  ##  ##  ##  ##  ##  ##  ##  ##  ##  ##  ##  ##  ##  ##  ##  ##  ##  ##  ##  ##  ##  ##  ##  ##  ##  ##  ##  ##  ##  ##  ##  ##  ##  ##  ##  ## [TB28_4]208	: ##  ##  ##  ##  ##  ##  ##  ##  ##  ##  ##  ##  ##  ##  ##  ##  ##  ##  ##  ##  ##  ##  ##  ##  ##  ##  ##  ##  ##  ##  ##  ##  ##  ##  ##  ##  ##  ##  ##  ##  ##  ##  ##  ##  ##  ##  ##  ##  ##  ##  ##  ##  ##  ##  ##  ##  ##  ##  ##  ##  ##  ##  ##  ##  ##  ##  ##  ##  ##  ##  ##  ##  ##  ##  ##  ##  ##  ##  ##  ##  ##  ##  ##  ##  ##  ##  ##  ##  ##  ##  ##  ##  ##  ##  ##  ##  ##  ##  ##  ##  ##  ##  ##  ##  ##  ##  ##  ##  ##  ##  ##  ##  ##  ##  ##  ##  ##  ##  ##  ##  ##  ##  ##  ##  ##  ##  ##  ##  ##  ##  ##  ##  ##  ##  ##  ##  ##  ##  ##  ##  ##  ##  ##  ##  ##  ##  ##  ##  ##  ##  ##  ##  ##  ##  ##  ##  ##  ##  ##  ##  ##  ##  ##  ##  ##  ##  ##  ##  ##  ##  ##  ##  ##  ##  ##  ##  ##  ##  ##  ##  ##  ##  ##  ##  ##  ##  ##  ##  ##  ##  ##  ##  ##  ##  ##  ##  ##  ##  ##  ##  ##  2  4  1  2  5  6  --  ##  ##  ##  ##  ##  ##  ##  ##  ##  ##  ##  ##  ##  ##  ##  ##  ##  ##  ##  ##  ##  ##  ##  ##  ##  ##  ##  ##  ##  ##  ##  ##  ##  ##  ##  ##  ##  ##  ##  ##  ##  ##  ##  ##  ##  ##  ##  ##  ##  ##  ##  ##  ##  ##  ##  ##  ##  ##  ##  ##  ##  ## [2805_26]209	: ##  ##  ##  ##  ##  ##  ##  ##  ##  ##  ##  ##  ##  ##  ##  ##  ##  ##  ##  ##  ##  ##  ##  ##  ##  ##  ##  ##  ##  ##  ##  ##  ##  ##  ##  ##  ##  ##  ##  ##  ##  ##  ##  ##  ##  ##  ##  ##  ##  ##  ##  ##  ##  ##  ##  ##  ##  ##  ##  ##  ##  ##  ##  ##  ##  ##  ##  ##  ##  ##  ##  ##  ##  ##  ##  ##  ##  ##  ##  ##  ##  ##  ##  ##  ##  ##  ##  ##  ##  ##  ##  ##  ##  ##  ##  ##  ##  ##  ##  ##  ##  ##  ##  ##  ##  ##  ##  ##  ##  ##  ##  ##  ##  ##  ##  ##  ##  ##  ##  ##  ##  ##  ##  ##  ##  ##  ##  ##  ##  ##  ##  ##  ##  ##  ##  ##  ##  ##  ##  ##  ##  ##  ##  ##  ##  ##  ##  ##  ##  ##  ##  ##  ##  ##  ##  ##  ##  ##  ##  ##  ##  ##  ##  ##  ##  ##  ##  ##  ##  ##  ##  ##  ##  ##  ##  ##  ##  ##  ##  ##  ##  ##  ##  ##  ##  ##  ##  ##  ##  ##  ##  ##  ##  ##  ##  ##  ##  ##  ##  ##  ##  ##  ##  ##  ##  ##  ##  ##  --  ##  ##  ##  ##  ##  ##  ##  ##  ##  ##  ##  ##  ##  ##  ##  ##  ##  ##  ##  ##  ##  ##  ##  ##  ##  ##  ##  ##  ##  ##  ##  ##  ##  ##  ##  ##  ##  ##  ##  ##  ##  ##  ##  ##  ##  ##  ##  ##  ##  ##  ##  ##  ##  ##  ##  ##  ##  ##  ##  ##  ## [2800_27]210	: ##  ##  ##  ##  ##  ##  ##  ##  ##  ##  ##  ##  ##  ##  ##  ##  ##  ##  ##  ##  ##  ##  ##  ##  ##  ##  ##  ##  ##  ##  ##  ##  ##  ##  ##  ##  ##  ##  ##  ##  ##  ##  ##  ##  ##  ##  ##  ##  ##  ##  ##  ##  ##  ##  ##  ##  ##  ##  ##  ##  ##  ##  ##  ##  ##  ##  ##  ##  ##  ##  ##  ##  ##  ##  ##  ##  ##  ##  ##  ##  ##  ##  ##  ##  ##  ##  ##  ##  ##  ##  ##  ##  ##  ##  ##  ##  ##  ##  ##  ##  ##  ##  ##  ##  ##  ##  ##  ##  ##  ##  ##  ##  ##  ##  ##  ##  ##  ##  ##  ##  ##  ##  ##  ##  ##  ##  ##  ##  ##  ##  ##  ##  ##  ##  ##  ##  ##  ##  ##  ##  ##  ##  ##  ##  ##  ##  ##  ##  ##  ##  ##  ##  ##  ##  ##  ##  ##  ##  ##  ##  ##  ##  ##  ##  ##  ##  ##  ##  ##  ##  ##  ##  ##  ##  ##  ##  ##  ##  ##  ##  ##  ##  ##  ##  ##  ##  ##  ##  ##  ##  ##  ##  ##  ##  ##  ##  ##  ##  ##  ##  ##  ##  ##  ##  ##  ##  ##  ##  ##  --  ##  ##  ##  ##  ##  ##  ##  ##  ##  ##  ##  ##  ##  ##  ##  ##  ##  ##  ##  ##  ##  ##  ##  ##  ##  ##  ##  ##  ##  ##  ##  ##  ##  ##  ##  ##  ##  ##  ##  ##  ##  ##  ##  ##  ##  ##  ##  ##  ##  ##  ##  ##  ##  ##  ##  ##  ##  ##  ##  ## [840_5]211	: ##  ##  ##  ##  ##  ##  ##  ##  ##  ##  ##  ##  ##  ##  ##  ##  ##  ##  ##  ##  ##  ##  ##  ##  ##  ##  ##  ##  ##  ##  ##  ##  ##  ##  ##  ##  ##  ##  ##  ##  ##  ##  ##  ##  ##  ##  ##  ##  ##  ##  ##  ##  ##  ##  ##  ##  ##  ##  ##  ##  ##  ##  ##  ##  ##  ##  ##  ##  ##  ##  ##  ##  ##  ##  ##  ##  ##  ##  ##  ##  ##  ##  ##  ##  ##  ##  ##  ##  ##  ##  ##  ##  ##  ##  ##  ##  ##  ##  ##  ##  ##  ##  ##  ##  ##  ##  ##  ##  ##  ##  ##  ##  ##  ##  ##  ##  ##  ##  ##  ##  ##  ##  ##  ##  ##  ##  ##  ##  ##  ##  ##  ##  ##  ##  ##  ##  ##  ##  ##  ##  ##  ##  ##  ##  ##  ##  ##  ##  ##  ##  ##  ##  ##  ##  ##  ##  ##  ##  ##  ##  ##  ##  ##  ##  ##  ##  ##  ##  ##  ##  ##  ##  ##  ##  ##  ##  ##  ##  ##  ##  ##  ##  ##  ##  ##  ##  ##  ##  ##  ##  ##  ##  ##  ##  ##  ##  ##  ##  ##  ##  ##  ##  ##  ##  ##  ##  ##  ##  ##  ##  --  6  1  5  6  7  6  7  6  6  ##  ##  ##  ##  ##  ##  ##  ##  ##  ##  ##  ##  ##  ##  ##  ##  ##  ##  ##  ##  ##  ##  ##  ##  ##  ##  ##  ##  ##  ##  ##  ##  ##  ##  ##  ##  ##  ##  ##  ##  ##  ##  ##  ##  ##  ##  ##  ##  ##  ## [842_5]212	: ##  ##  ##  ##  ##  ##  ##  ##  ##  ##  ##  ##  ##  ##  ##  ##  ##  ##  ##  ##  ##  ##  ##  ##  ##  ##  ##  ##  ##  ##  ##  ##  ##  ##  ##  ##  ##  ##  ##  ##  ##  ##  ##  ##  ##  ##  ##  ##  ##  ##  ##  ##  ##  ##  ##  ##  ##  ##  ##  ##  ##  ##  ##  ##  ##  ##  ##  ##  ##  ##  ##  ##  ##  ##  ##  ##  ##  ##  ##  ##  ##  ##  ##  ##  ##  ##  ##  ##  ##  ##  ##  ##  ##  ##  ##  ##  ##  ##  ##  ##  ##  ##  ##  ##  ##  ##  ##  ##  ##  ##  ##  ##  ##  ##  ##  ##  ##  ##  ##  ##  ##  ##  ##  ##  ##  ##  ##  ##  ##  ##  ##  ##  ##  ##  ##  ##  ##  ##  ##  ##  ##  ##  ##  ##  ##  ##  ##  ##  ##  ##  ##  ##  ##  ##  ##  ##  ##  ##  ##  ##  ##  ##  ##  ##  ##  ##  ##  ##  ##  ##  ##  ##  ##  ##  ##  ##  ##  ##  ##  ##  ##  ##  ##  ##  ##  ##  ##  ##  ##  ##  ##  ##  ##  ##  ##  ##  ##  ##  ##  ##  ##  ##  ##  ##  ##  ##  ##  ##  ##  ##  6  --  5  1  2  4  2  1  2  2  ##  ##  ##  ##  ##  ##  ##  ##  ##  ##  ##  ##  ##  ##  ##  ##  ##  ##  ##  ##  ##  ##  ##  ##  ##  ##  ##  ##  ##  ##  ##  ##  ##  ##  ##  ##  ##  ##  ##  ##  ##  ##  ##  ##  ##  ##  ##  ##  ##  ## [2777_5]213	: ##  ##  ##  ##  ##  ##  ##  ##  ##  ##  ##  ##  ##  ##  ##  ##  ##  ##  ##  ##  ##  ##  ##  ##  ##  ##  ##  ##  ##  ##  ##  ##  ##  ##  ##  ##  ##  ##  ##  ##  ##  ##  ##  ##  ##  ##  ##  ##  ##  ##  ##  ##  ##  ##  ##  ##  ##  ##  ##  ##  ##  ##  ##  ##  ##  ##  ##  ##  ##  ##  ##  ##  ##  ##  ##  ##  ##  ##  ##  ##  ##  ##  ##  ##  ##  ##  ##  ##  ##  ##  ##  ##  ##  ##  ##  ##  ##  ##  ##  ##  ##  ##  ##  ##  ##  ##  ##  ##  ##  ##  ##  ##  ##  ##  ##  ##  ##  ##  ##  ##  ##  ##  ##  ##  ##  ##  ##  ##  ##  ##  ##  ##  ##  ##  ##  ##  ##  ##  ##  ##  ##  ##  ##  ##  ##  ##  ##  ##  ##  ##  ##  ##  ##  ##  ##  ##  ##  ##  ##  ##  ##  ##  ##  ##  ##  ##  ##  ##  ##  ##  ##  ##  ##  ##  ##  ##  ##  ##  ##  ##  ##  ##  ##  ##  ##  ##  ##  ##  ##  ##  ##  ##  ##  ##  ##  ##  ##  ##  ##  ##  ##  ##  ##  ##  ##  ##  ##  ##  ##  ##  1  5  --  4  5  6  5  6  5  5  ##  ##  ##  ##  ##  ##  ##  ##  ##  ##  ##  ##  ##  ##  ##  ##  ##  ##  ##  ##  ##  ##  ##  ##  ##  ##  ##  ##  ##  ##  ##  ##  ##  ##  ##  ##  ##  ##  ##  ##  ##  ##  ##  ##  ##  ##  ##  ##  ##  ## [2779_5]214	: ##  ##  ##  ##  ##  ##  ##  ##  ##  ##  ##  ##  ##  ##  ##  ##  ##  ##  ##  ##  ##  ##  ##  ##  ##  ##  ##  ##  ##  ##  ##  ##  ##  ##  ##  ##  ##  ##  ##  ##  ##  ##  ##  ##  ##  ##  ##  ##  ##  ##  ##  ##  ##  ##  ##  ##  ##  ##  ##  ##  ##  ##  ##  ##  ##  ##  ##  ##  ##  ##  ##  ##  ##  ##  ##  ##  ##  ##  ##  ##  ##  ##  ##  ##  ##  ##  ##  ##  ##  ##  ##  ##  ##  ##  ##  ##  ##  ##  ##  ##  ##  ##  ##  ##  ##  ##  ##  ##  ##  ##  ##  ##  ##  ##  ##  ##  ##  ##  ##  ##  ##  ##  ##  ##  ##  ##  ##  ##  ##  ##  ##  ##  ##  ##  ##  ##  ##  ##  ##  ##  ##  ##  ##  ##  ##  ##  ##  ##  ##  ##  ##  ##  ##  ##  ##  ##  ##  ##  ##  ##  ##  ##  ##  ##  ##  ##  ##  ##  ##  ##  ##  ##  ##  ##  ##  ##  ##  ##  ##  ##  ##  ##  ##  ##  ##  ##  ##  ##  ##  ##  ##  ##  ##  ##  ##  ##  ##  ##  ##  ##  ##  ##  ##  ##  ##  ##  ##  ##  ##  ##  5  1  4  --  1  3  1  2  1  1  ##  ##  ##  ##  ##  ##  ##  ##  ##  ##  ##  ##  ##  ##  ##  ##  ##  ##  ##  ##  ##  ##  ##  ##  ##  ##  ##  ##  ##  ##  ##  ##  ##  ##  ##  ##  ##  ##  ##  ##  ##  ##  ##  ##  ##  ##  ##  ##  ##  ## [2791_5]215	: ##  ##  ##  ##  ##  ##  ##  ##  ##  ##  ##  ##  ##  ##  ##  ##  ##  ##  ##  ##  ##  ##  ##  ##  ##  ##  ##  ##  ##  ##  ##  ##  ##  ##  ##  ##  ##  ##  ##  ##  ##  ##  ##  ##  ##  ##  ##  ##  ##  ##  ##  ##  ##  ##  ##  ##  ##  ##  ##  ##  ##  ##  ##  ##  ##  ##  ##  ##  ##  ##  ##  ##  ##  ##  ##  ##  ##  ##  ##  ##  ##  ##  ##  ##  ##  ##  ##  ##  ##  ##  ##  ##  ##  ##  ##  ##  ##  ##  ##  ##  ##  ##  ##  ##  ##  ##  ##  ##  ##  ##  ##  ##  ##  ##  ##  ##  ##  ##  ##  ##  ##  ##  ##  ##  ##  ##  ##  ##  ##  ##  ##  ##  ##  ##  ##  ##  ##  ##  ##  ##  ##  ##  ##  ##  ##  ##  ##  ##  ##  ##  ##  ##  ##  ##  ##  ##  ##  ##  ##  ##  ##  ##  ##  ##  ##  ##  ##  ##  ##  ##  ##  ##  ##  ##  ##  ##  ##  ##  ##  ##  ##  ##  ##  ##  ##  ##  ##  ##  ##  ##  ##  ##  ##  ##  ##  ##  ##  ##  ##  ##  ##  ##  ##  ##  ##  ##  ##  ##  ##  ##  6  2  5  1  --  4  2  3  2  2  ##  ##  ##  ##  ##  ##  ##  ##  ##  ##  ##  ##  ##  ##  ##  ##  ##  ##  ##  ##  ##  ##  ##  ##  ##  ##  ##  ##  ##  ##  ##  ##  ##  ##  ##  ##  ##  ##  ##  ##  ##  ##  ##  ##  ##  ##  ##  ##  ##  ## [2904_5]216	: ##  ##  ##  ##  ##  ##  ##  ##  ##  ##  ##  ##  ##  ##  ##  ##  ##  ##  ##  ##  ##  ##  ##  ##  ##  ##  ##  ##  ##  ##  ##  ##  ##  ##  ##  ##  ##  ##  ##  ##  ##  ##  ##  ##  ##  ##  ##  ##  ##  ##  ##  ##  ##  ##  ##  ##  ##  ##  ##  ##  ##  ##  ##  ##  ##  ##  ##  ##  ##  ##  ##  ##  ##  ##  ##  ##  ##  ##  ##  ##  ##  ##  ##  ##  ##  ##  ##  ##  ##  ##  ##  ##  ##  ##  ##  ##  ##  ##  ##  ##  ##  ##  ##  ##  ##  ##  ##  ##  ##  ##  ##  ##  ##  ##  ##  ##  ##  ##  ##  ##  ##  ##  ##  ##  ##  ##  ##  ##  ##  ##  ##  ##  ##  ##  ##  ##  ##  ##  ##  ##  ##  ##  ##  ##  ##  ##  ##  ##  ##  ##  ##  ##  ##  ##  ##  ##  ##  ##  ##  ##  ##  ##  ##  ##  ##  ##  ##  ##  ##  ##  ##  ##  ##  ##  ##  ##  ##  ##  ##  ##  ##  ##  ##  ##  ##  ##  ##  ##  ##  ##  ##  ##  ##  ##  ##  ##  ##  ##  ##  ##  ##  ##  ##  ##  ##  ##  ##  ##  ##  ##  7  4  6  3  4  --  4  5  4  4  ##  ##  ##  ##  ##  ##  ##  ##  ##  ##  ##  ##  ##  ##  ##  ##  ##  ##  ##  ##  ##  ##  ##  ##  ##  ##  ##  ##  ##  ##  ##  ##  ##  ##  ##  ##  ##  ##  ##  ##  ##  ##  ##  ##  ##  ##  ##  ##  ##  ## [2918_5]217	: ##  ##  ##  ##  ##  ##  ##  ##  ##  ##  ##  ##  ##  ##  ##  ##  ##  ##  ##  ##  ##  ##  ##  ##  ##  ##  ##  ##  ##  ##  ##  ##  ##  ##  ##  ##  ##  ##  ##  ##  ##  ##  ##  ##  ##  ##  ##  ##  ##  ##  ##  ##  ##  ##  ##  ##  ##  ##  ##  ##  ##  ##  ##  ##  ##  ##  ##  ##  ##  ##  ##  ##  ##  ##  ##  ##  ##  ##  ##  ##  ##  ##  ##  ##  ##  ##  ##  ##  ##  ##  ##  ##  ##  ##  ##  ##  ##  ##  ##  ##  ##  ##  ##  ##  ##  ##  ##  ##  ##  ##  ##  ##  ##  ##  ##  ##  ##  ##  ##  ##  ##  ##  ##  ##  ##  ##  ##  ##  ##  ##  ##  ##  ##  ##  ##  ##  ##  ##  ##  ##  ##  ##  ##  ##  ##  ##  ##  ##  ##  ##  ##  ##  ##  ##  ##  ##  ##  ##  ##  ##  ##  ##  ##  ##  ##  ##  ##  ##  ##  ##  ##  ##  ##  ##  ##  ##  ##  ##  ##  ##  ##  ##  ##  ##  ##  ##  ##  ##  ##  ##  ##  ##  ##  ##  ##  ##  ##  ##  ##  ##  ##  ##  ##  ##  ##  ##  ##  ##  ##  ##  6  2  5  1  2  4  --  3  2  2  ##  ##  ##  ##  ##  ##  ##  ##  ##  ##  ##  ##  ##  ##  ##  ##  ##  ##  ##  ##  ##  ##  ##  ##  ##  ##  ##  ##  ##  ##  ##  ##  ##  ##  ##  ##  ##  ##  ##  ##  ##  ##  ##  ##  ##  ##  ##  ##  ##  ## [2928_5]218	: ##  ##  ##  ##  ##  ##  ##  ##  ##  ##  ##  ##  ##  ##  ##  ##  ##  ##  ##  ##  ##  ##  ##  ##  ##  ##  ##  ##  ##  ##  ##  ##  ##  ##  ##  ##  ##  ##  ##  ##  ##  ##  ##  ##  ##  ##  ##  ##  ##  ##  ##  ##  ##  ##  ##  ##  ##  ##  ##  ##  ##  ##  ##  ##  ##  ##  ##  ##  ##  ##  ##  ##  ##  ##  ##  ##  ##  ##  ##  ##  ##  ##  ##  ##  ##  ##  ##  ##  ##  ##  ##  ##  ##  ##  ##  ##  ##  ##  ##  ##  ##  ##  ##  ##  ##  ##  ##  ##  ##  ##  ##  ##  ##  ##  ##  ##  ##  ##  ##  ##  ##  ##  ##  ##  ##  ##  ##  ##  ##  ##  ##  ##  ##  ##  ##  ##  ##  ##  ##  ##  ##  ##  ##  ##  ##  ##  ##  ##  ##  ##  ##  ##  ##  ##  ##  ##  ##  ##  ##  ##  ##  ##  ##  ##  ##  ##  ##  ##  ##  ##  ##  ##  ##  ##  ##  ##  ##  ##  ##  ##  ##  ##  ##  ##  ##  ##  ##  ##  ##  ##  ##  ##  ##  ##  ##  ##  ##  ##  ##  ##  ##  ##  ##  ##  ##  ##  ##  ##  ##  ##  7  1  6  2  3  5  3  --  3  3  ##  ##  ##  ##  ##  ##  ##  ##  ##  ##  ##  ##  ##  ##  ##  ##  ##  ##  ##  ##  ##  ##  ##  ##  ##  ##  ##  ##  ##  ##  ##  ##  ##  ##  ##  ##  ##  ##  ##  ##  ##  ##  ##  ##  ##  ##  ##  ##  ##  ## [PONOR087_13_5]219	: ##  ##  ##  ##  ##  ##  ##  ##  ##  ##  ##  ##  ##  ##  ##  ##  ##  ##  ##  ##  ##  ##  ##  ##  ##  ##  ##  ##  ##  ##  ##  ##  ##  ##  ##  ##  ##  ##  ##  ##  ##  ##  ##  ##  ##  ##  ##  ##  ##  ##  ##  ##  ##  ##  ##  ##  ##  ##  ##  ##  ##  ##  ##  ##  ##  ##  ##  ##  ##  ##  ##  ##  ##  ##  ##  ##  ##  ##  ##  ##  ##  ##  ##  ##  ##  ##  ##  ##  ##  ##  ##  ##  ##  ##  ##  ##  ##  ##  ##  ##  ##  ##  ##  ##  ##  ##  ##  ##  ##  ##  ##  ##  ##  ##  ##  ##  ##  ##  ##  ##  ##  ##  ##  ##  ##  ##  ##  ##  ##  ##  ##  ##  ##  ##  ##  ##  ##  ##  ##  ##  ##  ##  ##  ##  ##  ##  ##  ##  ##  ##  ##  ##  ##  ##  ##  ##  ##  ##  ##  ##  ##  ##  ##  ##  ##  ##  ##  ##  ##  ##  ##  ##  ##  ##  ##  ##  ##  ##  ##  ##  ##  ##  ##  ##  ##  ##  ##  ##  ##  ##  ##  ##  ##  ##  ##  ##  ##  ##  ##  ##  ##  ##  ##  ##  ##  ##  ##  ##  ##  ##  6  2  5  1  2  4  2  3  --  2  ##  ##  ##  ##  ##  ##  ##  ##  ##  ##  ##  ##  ##  ##  ##  ##  ##  ##  ##  ##  ##  ##  ##  ##  ##  ##  ##  ##  ##  ##  ##  ##  ##  ##  ##  ##  ##  ##  ##  ##  ##  ##  ##  ##  ##  ##  ##  ##  ##  ## [T02_5]220	: ##  ##  ##  ##  ##  ##  ##  ##  ##  ##  ##  ##  ##  ##  ##  ##  ##  ##  ##  ##  ##  ##  ##  ##  ##  ##  ##  ##  ##  ##  ##  ##  ##  ##  ##  ##  ##  ##  ##  ##  ##  ##  ##  ##  ##  ##  ##  ##  ##  ##  ##  ##  ##  ##  ##  ##  ##  ##  ##  ##  ##  ##  ##  ##  ##  ##  ##  ##  ##  ##  ##  ##  ##  ##  ##  ##  ##  ##  ##  ##  ##  ##  ##  ##  ##  ##  ##  ##  ##  ##  ##  ##  ##  ##  ##  ##  ##  ##  ##  ##  ##  ##  ##  ##  ##  ##  ##  ##  ##  ##  ##  ##  ##  ##  ##  ##  ##  ##  ##  ##  ##  ##  ##  ##  ##  ##  ##  ##  ##  ##  ##  ##  ##  ##  ##  ##  ##  ##  ##  ##  ##  ##  ##  ##  ##  ##  ##  ##  ##  ##  ##  ##  ##  ##  ##  ##  ##  ##  ##  ##  ##  ##  ##  ##  ##  ##  ##  ##  ##  ##  ##  ##  ##  ##  ##  ##  ##  ##  ##  ##  ##  ##  ##  ##  ##  ##  ##  ##  ##  ##  ##  ##  ##  ##  ##  ##  ##  ##  ##  ##  ##  ##  ##  ##  ##  ##  ##  ##  ##  ##  6  2  5  1  2  4  2  3  2  --  ##  ##  ##  ##  ##  ##  ##  ##  ##  ##  ##  ##  ##  ##  ##  ##  ##  ##  ##  ##  ##  ##  ##  ##  ##  ##  ##  ##  ##  ##  ##  ##  ##  ##  ##  ##  ##  ##  ##  ##  ##  ##  ##  ##  ##  ##  ##  ##  ##  ## [2267_16]221	: ##  ##  ##  ##  ##  ##  ##  ##  ##  ##  ##  ##  ##  ##  ##  ##  ##  ##  ##  ##  ##  ##  ##  ##  ##  ##  ##  ##  ##  ##  ##  ##  ##  ##  ##  ##  ##  ##  ##  ##  ##  ##  ##  ##  ##  ##  ##  ##  ##  ##  ##  ##  ##  ##  ##  ##  ##  ##  ##  ##  ##  ##  ##  ##  ##  ##  ##  ##  ##  ##  ##  ##  ##  ##  ##  ##  ##  ##  ##  ##  ##  ##  ##  ##  ##  ##  ##  ##  ##  ##  ##  ##  ##  ##  ##  ##  ##  ##  ##  ##  ##  ##  ##  ##  ##  ##  ##  ##  ##  ##  ##  ##  ##  ##  ##  ##  ##  ##  ##  ##  ##  ##  ##  ##  ##  ##  ##  ##  ##  ##  ##  ##  ##  ##  ##  ##  ##  ##  ##  ##  ##  ##  ##  ##  ##  ##  ##  ##  ##  ##  ##  ##  ##  ##  ##  ##  ##  ##  ##  ##  ##  ##  ##  ##  ##  ##  ##  ##  ##  ##  ##  ##  ##  ##  ##  ##  ##  ##  ##  ##  ##  ##  ##  ##  ##  ##  ##  ##  ##  ##  ##  ##  ##  ##  ##  ##  ##  ##  ##  ##  ##  ##  ##  ##  ##  ##  ##  ##  ##  ##  ##  ##  ##  ##  ##  ##  ##  ##  ##  ##  --  9  5  6  6  5  2  4  4  1  9  6  4  4  4  6  4  1  4  2  4  3  1  4  4  3  3  2  3  3  4  2  5  2  3  4  8  5  5  3  4  4  3  6  7  6  7  7  7  9 [2268_16]222	: ##  ##  ##  ##  ##  ##  ##  ##  ##  ##  ##  ##  ##  ##  ##  ##  ##  ##  ##  ##  ##  ##  ##  ##  ##  ##  ##  ##  ##  ##  ##  ##  ##  ##  ##  ##  ##  ##  ##  ##  ##  ##  ##  ##  ##  ##  ##  ##  ##  ##  ##  ##  ##  ##  ##  ##  ##  ##  ##  ##  ##  ##  ##  ##  ##  ##  ##  ##  ##  ##  ##  ##  ##  ##  ##  ##  ##  ##  ##  ##  ##  ##  ##  ##  ##  ##  ##  ##  ##  ##  ##  ##  ##  ##  ##  ##  ##  ##  ##  ##  ##  ##  ##  ##  ##  ##  ##  ##  ##  ##  ##  ##  ##  ##  ##  ##  ##  ##  ##  ##  ##  ##  ##  ##  ##  ##  ##  ##  ##  ##  ##  ##  ##  ##  ##  ##  ##  ##  ##  ##  ##  ##  ##  ##  ##  ##  ##  ##  ##  ##  ##  ##  ##  ##  ##  ##  ##  ##  ##  ##  ##  ##  ##  ##  ##  ##  ##  ##  ##  ##  ##  ##  ##  ##  ##  ##  ##  ##  ##  ##  ##  ##  ##  ##  ##  ##  ##  ##  ##  ##  ##  ##  ##  ##  ##  ##  ##  ##  ##  ##  ##  ##  ##  ##  ##  ##  ##  ##  ##  ##  ##  ##  ##  ##  ##  ##  ##  ##  ##  ##  9  --  6  3  11  7  7  7  11  8  8  5  13  10  11  6  7  10  7  9  9  10  10  9  9  9  8  9  8  10  9  9  12  9  9  9  14  10  10  10  11  11  8  7  6  7  6  8  6  7 [2269_16]223	: ##  ##  ##  ##  ##  ##  ##  ##  ##  ##  ##  ##  ##  ##  ##  ##  ##  ##  ##  ##  ##  ##  ##  ##  ##  ##  ##  ##  ##  ##  ##  ##  ##  ##  ##  ##  ##  ##  ##  ##  ##  ##  ##  ##  ##  ##  ##  ##  ##  ##  ##  ##  ##  ##  ##  ##  ##  ##  ##  ##  ##  ##  ##  ##  ##  ##  ##  ##  ##  ##  ##  ##  ##  ##  ##  ##  ##  ##  ##  ##  ##  ##  ##  ##  ##  ##  ##  ##  ##  ##  ##  ##  ##  ##  ##  ##  ##  ##  ##  ##  ##  ##  ##  ##  ##  ##  ##  ##  ##  ##  ##  ##  ##  ##  ##  ##  ##  ##  ##  ##  ##  ##  ##  ##  ##  ##  ##  ##  ##  ##  ##  ##  ##  ##  ##  ##  ##  ##  ##  ##  ##  ##  ##  ##  ##  ##  ##  ##  ##  ##  ##  ##  ##  ##  ##  ##  ##  ##  ##  ##  ##  ##  ##  ##  ##  ##  ##  ##  ##  ##  ##  ##  ##  ##  ##  ##  ##  ##  ##  ##  ##  ##  ##  ##  ##  ##  ##  ##  ##  ##  ##  ##  ##  ##  ##  ##  ##  ##  ##  ##  ##  ##  ##  ##  ##  ##  ##  ##  ##  ##  ##  ##  ##  ##  ##  ##  ##  ##  ##  ##  5  6  --  3  7  6  5  5  7  4  4  3  9  7  7  4  5  6  3  5  7  6  6  7  7  7  4  5  4  6  7  5  8  5  7  7  11  8  8  6  7  7  4  1  4  5  4  2  4  7 [2270_16]224	: ##  ##  ##  ##  ##  ##  ##  ##  ##  ##  ##  ##  ##  ##  ##  ##  ##  ##  ##  ##  ##  ##  ##  ##  ##  ##  ##  ##  ##  ##  ##  ##  ##  ##  ##  ##  ##  ##  ##  ##  ##  ##  ##  ##  ##  ##  ##  ##  ##  ##  ##  ##  ##  ##  ##  ##  ##  ##  ##  ##  ##  ##  ##  ##  ##  ##  ##  ##  ##  ##  ##  ##  ##  ##  ##  ##  ##  ##  ##  ##  ##  ##  ##  ##  ##  ##  ##  ##  ##  ##  ##  ##  ##  ##  ##  ##  ##  ##  ##  ##  ##  ##  ##  ##  ##  ##  ##  ##  ##  ##  ##  ##  ##  ##  ##  ##  ##  ##  ##  ##  ##  ##  ##  ##  ##  ##  ##  ##  ##  ##  ##  ##  ##  ##  ##  ##  ##  ##  ##  ##  ##  ##  ##  ##  ##  ##  ##  ##  ##  ##  ##  ##  ##  ##  ##  ##  ##  ##  ##  ##  ##  ##  ##  ##  ##  ##  ##  ##  ##  ##  ##  ##  ##  ##  ##  ##  ##  ##  ##  ##  ##  ##  ##  ##  ##  ##  ##  ##  ##  ##  ##  ##  ##  ##  ##  ##  ##  ##  ##  ##  ##  ##  ##  ##  ##  ##  ##  ##  ##  ##  ##  ##  ##  ##  ##  ##  ##  ##  ##  ##  6  3  3  --  8  5  4  4  8  5  7  2  10  7  8  3  4  7  4  6  8  7  7  6  6  8  5  6  5  7  6  6  9  6  6  8  12  9  7  7  8  8  5  4  3  4  3  5  3  6 [2279_16]225	: ##  ##  ##  ##  ##  ##  ##  ##  ##  ##  ##  ##  ##  ##  ##  ##  ##  ##  ##  ##  ##  ##  ##  ##  ##  ##  ##  ##  ##  ##  ##  ##  ##  ##  ##  ##  ##  ##  ##  ##  ##  ##  ##  ##  ##  ##  ##  ##  ##  ##  ##  ##  ##  ##  ##  ##  ##  ##  ##  ##  ##  ##  ##  ##  ##  ##  ##  ##  ##  ##  ##  ##  ##  ##  ##  ##  ##  ##  ##  ##  ##  ##  ##  ##  ##  ##  ##  ##  ##  ##  ##  ##  ##  ##  ##  ##  ##  ##  ##  ##  ##  ##  ##  ##  ##  ##  ##  ##  ##  ##  ##  ##  ##  ##  ##  ##  ##  ##  ##  ##  ##  ##  ##  ##  ##  ##  ##  ##  ##  ##  ##  ##  ##  ##  ##  ##  ##  ##  ##  ##  ##  ##  ##  ##  ##  ##  ##  ##  ##  ##  ##  ##  ##  ##  ##  ##  ##  ##  ##  ##  ##  ##  ##  ##  ##  ##  ##  ##  ##  ##  ##  ##  ##  ##  ##  ##  ##  ##  ##  ##  ##  ##  ##  ##  ##  ##  ##  ##  ##  ##  ##  ##  ##  ##  ##  ##  ##  ##  ##  ##  ##  ##  ##  ##  ##  ##  ##  ##  ##  ##  ##  ##  ##  ##  ##  ##  ##  ##  ##  ##  6  11  7  8  --  9  8  8  10  7  11  8  10  10  8  7  8  7  6  8  10  9  7  10  10  8  5  8  7  9  10  8  11  8  9  10  14  11  11  9  10  10  7  8  9  8  9  9  9  10 [2280_16]226	: ##  ##  ##  ##  ##  ##  ##  ##  ##  ##  ##  ##  ##  ##  ##  ##  ##  ##  ##  ##  ##  ##  ##  ##  ##  ##  ##  ##  ##  ##  ##  ##  ##  ##  ##  ##  ##  ##  ##  ##  ##  ##  ##  ##  ##  ##  ##  ##  ##  ##  ##  ##  ##  ##  ##  ##  ##  ##  ##  ##  ##  ##  ##  ##  ##  ##  ##  ##  ##  ##  ##  ##  ##  ##  ##  ##  ##  ##  ##  ##  ##  ##  ##  ##  ##  ##  ##  ##  ##  ##  ##  ##  ##  ##  ##  ##  ##  ##  ##  ##  ##  ##  ##  ##  ##  ##  ##  ##  ##  ##  ##  ##  ##  ##  ##  ##  ##  ##  ##  ##  ##  ##  ##  ##  ##  ##  ##  ##  ##  ##  ##  ##  ##  ##  ##  ##  ##  ##  ##  ##  ##  ##  ##  ##  ##  ##  ##  ##  ##  ##  ##  ##  ##  ##  ##  ##  ##  ##  ##  ##  ##  ##  ##  ##  ##  ##  ##  ##  ##  ##  ##  ##  ##  ##  ##  ##  ##  ##  ##  ##  ##  ##  ##  ##  ##  ##  ##  ##  ##  ##  ##  ##  ##  ##  ##  ##  ##  ##  ##  ##  ##  ##  ##  ##  ##  ##  ##  ##  ##  ##  ##  ##  ##  ##  ##  ##  ##  ##  ##  ##  5  7  6  5  9  --  3  3  7  4  9  5  9  6  7  6  4  6  4  5  7  4  4  5  7  5  4  3  3  4  6  5  8  5  5  4  9  8  6  4  7  4  4  6  6  5  5  8  6  7 [2284_16]227	: ##  ##  ##  ##  ##  ##  ##  ##  ##  ##  ##  ##  ##  ##  ##  ##  ##  ##  ##  ##  ##  ##  ##  ##  ##  ##  ##  ##  ##  ##  ##  ##  ##  ##  ##  ##  ##  ##  ##  ##  ##  ##  ##  ##  ##  ##  ##  ##  ##  ##  ##  ##  ##  ##  ##  ##  ##  ##  ##  ##  ##  ##  ##  ##  ##  ##  ##  ##  ##  ##  ##  ##  ##  ##  ##  ##  ##  ##  ##  ##  ##  ##  ##  ##  ##  ##  ##  ##  ##  ##  ##  ##  ##  ##  ##  ##  ##  ##  ##  ##  ##  ##  ##  ##  ##  ##  ##  ##  ##  ##  ##  ##  ##  ##  ##  ##  ##  ##  ##  ##  ##  ##  ##  ##  ##  ##  ##  ##  ##  ##  ##  ##  ##  ##  ##  ##  ##  ##  ##  ##  ##  ##  ##  ##  ##  ##  ##  ##  ##  ##  ##  ##  ##  ##  ##  ##  ##  ##  ##  ##  ##  ##  ##  ##  ##  ##  ##  ##  ##  ##  ##  ##  ##  ##  ##  ##  ##  ##  ##  ##  ##  ##  ##  ##  ##  ##  ##  ##  ##  ##  ##  ##  ##  ##  ##  ##  ##  ##  ##  ##  ##  ##  ##  ##  ##  ##  ##  ##  ##  ##  ##  ##  ##  ##  ##  ##  ##  ##  ##  ##  2  7  5  4  8  3  --  2  4  1  9  4  6  3  4  6  2  3  4  2  4  3  3  2  4  5  3  2  3  3  4  2  5  2  2  4  8  5  3  3  4  4  3  6  5  4  5  7  5  7 [2288_16]228	: ##  ##  ##  ##  ##  ##  ##  ##  ##  ##  ##  ##  ##  ##  ##  ##  ##  ##  ##  ##  ##  ##  ##  ##  ##  ##  ##  ##  ##  ##  ##  ##  ##  ##  ##  ##  ##  ##  ##  ##  ##  ##  ##  ##  ##  ##  ##  ##  ##  ##  ##  ##  ##  ##  ##  ##  ##  ##  ##  ##  ##  ##  ##  ##  ##  ##  ##  ##  ##  ##  ##  ##  ##  ##  ##  ##  ##  ##  ##  ##  ##  ##  ##  ##  ##  ##  ##  ##  ##  ##  ##  ##  ##  ##  ##  ##  ##  ##  ##  ##  ##  ##  ##  ##  ##  ##  ##  ##  ##  ##  ##  ##  ##  ##  ##  ##  ##  ##  ##  ##  ##  ##  ##  ##  ##  ##  ##  ##  ##  ##  ##  ##  ##  ##  ##  ##  ##  ##  ##  ##  ##  ##  ##  ##  ##  ##  ##  ##  ##  ##  ##  ##  ##  ##  ##  ##  ##  ##  ##  ##  ##  ##  ##  ##  ##  ##  ##  ##  ##  ##  ##  ##  ##  ##  ##  ##  ##  ##  ##  ##  ##  ##  ##  ##  ##  ##  ##  ##  ##  ##  ##  ##  ##  ##  ##  ##  ##  ##  ##  ##  ##  ##  ##  ##  ##  ##  ##  ##  ##  ##  ##  ##  ##  ##  ##  ##  ##  ##  ##  ##  4  7  5  4  8  3  2  --  6  3  9  4  8  5  6  6  4  5  4  4  6  5  5  4  6  6  3  4  3  5  6  4  7  4  4  6  10  7  5  5  6  6  3  6  5  4  5  7  5  7 [2289_16]229	: ##  ##  ##  ##  ##  ##  ##  ##  ##  ##  ##  ##  ##  ##  ##  ##  ##  ##  ##  ##  ##  ##  ##  ##  ##  ##  ##  ##  ##  ##  ##  ##  ##  ##  ##  ##  ##  ##  ##  ##  ##  ##  ##  ##  ##  ##  ##  ##  ##  ##  ##  ##  ##  ##  ##  ##  ##  ##  ##  ##  ##  ##  ##  ##  ##  ##  ##  ##  ##  ##  ##  ##  ##  ##  ##  ##  ##  ##  ##  ##  ##  ##  ##  ##  ##  ##  ##  ##  ##  ##  ##  ##  ##  ##  ##  ##  ##  ##  ##  ##  ##  ##  ##  ##  ##  ##  ##  ##  ##  ##  ##  ##  ##  ##  ##  ##  ##  ##  ##  ##  ##  ##  ##  ##  ##  ##  ##  ##  ##  ##  ##  ##  ##  ##  ##  ##  ##  ##  ##  ##  ##  ##  ##  ##  ##  ##  ##  ##  ##  ##  ##  ##  ##  ##  ##  ##  ##  ##  ##  ##  ##  ##  ##  ##  ##  ##  ##  ##  ##  ##  ##  ##  ##  ##  ##  ##  ##  ##  ##  ##  ##  ##  ##  ##  ##  ##  ##  ##  ##  ##  ##  ##  ##  ##  ##  ##  ##  ##  ##  ##  ##  ##  ##  ##  ##  ##  ##  ##  ##  ##  ##  ##  ##  ##  ##  ##  ##  ##  ##  ##  4  11  7  8  10  7  4  6  --  3  11  8  8  6  6  8  6  5  6  4  6  5  5  6  6  7  5  4  5  5  6  4  7  4  6  6  10  5  7  5  6  6  5  8  9  8  9  9  9  11 [2290_16]230	: ##  ##  ##  ##  ##  ##  ##  ##  ##  ##  ##  ##  ##  ##  ##  ##  ##  ##  ##  ##  ##  ##  ##  ##  ##  ##  ##  ##  ##  ##  ##  ##  ##  ##  ##  ##  ##  ##  ##  ##  ##  ##  ##  ##  ##  ##  ##  ##  ##  ##  ##  ##  ##  ##  ##  ##  ##  ##  ##  ##  ##  ##  ##  ##  ##  ##  ##  ##  ##  ##  ##  ##  ##  ##  ##  ##  ##  ##  ##  ##  ##  ##  ##  ##  ##  ##  ##  ##  ##  ##  ##  ##  ##  ##  ##  ##  ##  ##  ##  ##  ##  ##  ##  ##  ##  ##  ##  ##  ##  ##  ##  ##  ##  ##  ##  ##  ##  ##  ##  ##  ##  ##  ##  ##  ##  ##  ##  ##  ##  ##  ##  ##  ##  ##  ##  ##  ##  ##  ##  ##  ##  ##  ##  ##  ##  ##  ##  ##  ##  ##  ##  ##  ##  ##  ##  ##  ##  ##  ##  ##  ##  ##  ##  ##  ##  ##  ##  ##  ##  ##  ##  ##  ##  ##  ##  ##  ##  ##  ##  ##  ##  ##  ##  ##  ##  ##  ##  ##  ##  ##  ##  ##  ##  ##  ##  ##  ##  ##  ##  ##  ##  ##  ##  ##  ##  ##  ##  ##  ##  ##  ##  ##  ##  ##  ##  ##  ##  ##  ##  ##  1  8  4  5  7  4  1  3  3  --  8  5  5  3  3  5  3  2  3  1  3  2  2  3  3  4  2  1  2  2  3  1  4  1  3  3  7  4  4  2  3  3  2  5  6  5  6  6  6  8 [2294_16]231	: ##  ##  ##  ##  ##  ##  ##  ##  ##  ##  ##  ##  ##  ##  ##  ##  ##  ##  ##  ##  ##  ##  ##  ##  ##  ##  ##  ##  ##  ##  ##  ##  ##  ##  ##  ##  ##  ##  ##  ##  ##  ##  ##  ##  ##  ##  ##  ##  ##  ##  ##  ##  ##  ##  ##  ##  ##  ##  ##  ##  ##  ##  ##  ##  ##  ##  ##  ##  ##  ##  ##  ##  ##  ##  ##  ##  ##  ##  ##  ##  ##  ##  ##  ##  ##  ##  ##  ##  ##  ##  ##  ##  ##  ##  ##  ##  ##  ##  ##  ##  ##  ##  ##  ##  ##  ##  ##  ##  ##  ##  ##  ##  ##  ##  ##  ##  ##  ##  ##  ##  ##  ##  ##  ##  ##  ##  ##  ##  ##  ##  ##  ##  ##  ##  ##  ##  ##  ##  ##  ##  ##  ##  ##  ##  ##  ##  ##  ##  ##  ##  ##  ##  ##  ##  ##  ##  ##  ##  ##  ##  ##  ##  ##  ##  ##  ##  ##  ##  ##  ##  ##  ##  ##  ##  ##  ##  ##  ##  ##  ##  ##  ##  ##  ##  ##  ##  ##  ##  ##  ##  ##  ##  ##  ##  ##  ##  ##  ##  ##  ##  ##  ##  ##  ##  ##  ##  ##  ##  ##  ##  ##  ##  ##  ##  ##  ##  ##  ##  ##  ##  9  8  4  7  11  9  9  9  11  8  --  7  11  11  11  6  9  10  7  9  11  10  10  11  11  10  8  9  8  10  10  9  12  9  9  11  14  12  12  10  11  11  8  5  6  9  8  6  8  11 [2295_16]232	: ##  ##  ##  ##  ##  ##  ##  ##  ##  ##  ##  ##  ##  ##  ##  ##  ##  ##  ##  ##  ##  ##  ##  ##  ##  ##  ##  ##  ##  ##  ##  ##  ##  ##  ##  ##  ##  ##  ##  ##  ##  ##  ##  ##  ##  ##  ##  ##  ##  ##  ##  ##  ##  ##  ##  ##  ##  ##  ##  ##  ##  ##  ##  ##  ##  ##  ##  ##  ##  ##  ##  ##  ##  ##  ##  ##  ##  ##  ##  ##  ##  ##  ##  ##  ##  ##  ##  ##  ##  ##  ##  ##  ##  ##  ##  ##  ##  ##  ##  ##  ##  ##  ##  ##  ##  ##  ##  ##  ##  ##  ##  ##  ##  ##  ##  ##  ##  ##  ##  ##  ##  ##  ##  ##  ##  ##  ##  ##  ##  ##  ##  ##  ##  ##  ##  ##  ##  ##  ##  ##  ##  ##  ##  ##  ##  ##  ##  ##  ##  ##  ##  ##  ##  ##  ##  ##  ##  ##  ##  ##  ##  ##  ##  ##  ##  ##  ##  ##  ##  ##  ##  ##  ##  ##  ##  ##  ##  ##  ##  ##  ##  ##  ##  ##  ##  ##  ##  ##  ##  ##  ##  ##  ##  ##  ##  ##  ##  ##  ##  ##  ##  ##  ##  ##  ##  ##  ##  ##  ##  ##  ##  ##  ##  ##  ##  ##  ##  ##  ##  ##  6  5  3  2  8  5  4  4  8  5  7  --  10  7  8  5  2  7  4  6  8  7  7  6  8  6  5  6  5  7  8  6  9  6  6  8  10  9  7  7  8  8  5  4  3  4  3  5  3  6 [2297_16]233	: ##  ##  ##  ##  ##  ##  ##  ##  ##  ##  ##  ##  ##  ##  ##  ##  ##  ##  ##  ##  ##  ##  ##  ##  ##  ##  ##  ##  ##  ##  ##  ##  ##  ##  ##  ##  ##  ##  ##  ##  ##  ##  ##  ##  ##  ##  ##  ##  ##  ##  ##  ##  ##  ##  ##  ##  ##  ##  ##  ##  ##  ##  ##  ##  ##  ##  ##  ##  ##  ##  ##  ##  ##  ##  ##  ##  ##  ##  ##  ##  ##  ##  ##  ##  ##  ##  ##  ##  ##  ##  ##  ##  ##  ##  ##  ##  ##  ##  ##  ##  ##  ##  ##  ##  ##  ##  ##  ##  ##  ##  ##  ##  ##  ##  ##  ##  ##  ##  ##  ##  ##  ##  ##  ##  ##  ##  ##  ##  ##  ##  ##  ##  ##  ##  ##  ##  ##  ##  ##  ##  ##  ##  ##  ##  ##  ##  ##  ##  ##  ##  ##  ##  ##  ##  ##  ##  ##  ##  ##  ##  ##  ##  ##  ##  ##  ##  ##  ##  ##  ##  ##  ##  ##  ##  ##  ##  ##  ##  ##  ##  ##  ##  ##  ##  ##  ##  ##  ##  ##  ##  ##  ##  ##  ##  ##  ##  ##  ##  ##  ##  ##  ##  ##  ##  ##  ##  ##  ##  ##  ##  ##  ##  ##  ##  ##  ##  ##  ##  ##  ##  4  13  9  10  10  9  6  8  8  5  11  10  --  8  8  8  8  5  8  6  8  7  5  8  8  7  7  6  7  7  8  6  9  6  5  8  12  9  9  7  8  8  7  10  9  10  11  11  9  13 [2298_16]234	: ##  ##  ##  ##  ##  ##  ##  ##  ##  ##  ##  ##  ##  ##  ##  ##  ##  ##  ##  ##  ##  ##  ##  ##  ##  ##  ##  ##  ##  ##  ##  ##  ##  ##  ##  ##  ##  ##  ##  ##  ##  ##  ##  ##  ##  ##  ##  ##  ##  ##  ##  ##  ##  ##  ##  ##  ##  ##  ##  ##  ##  ##  ##  ##  ##  ##  ##  ##  ##  ##  ##  ##  ##  ##  ##  ##  ##  ##  ##  ##  ##  ##  ##  ##  ##  ##  ##  ##  ##  ##  ##  ##  ##  ##  ##  ##  ##  ##  ##  ##  ##  ##  ##  ##  ##  ##  ##  ##  ##  ##  ##  ##  ##  ##  ##  ##  ##  ##  ##  ##  ##  ##  ##  ##  ##  ##  ##  ##  ##  ##  ##  ##  ##  ##  ##  ##  ##  ##  ##  ##  ##  ##  ##  ##  ##  ##  ##  ##  ##  ##  ##  ##  ##  ##  ##  ##  ##  ##  ##  ##  ##  ##  ##  ##  ##  ##  ##  ##  ##  ##  ##  ##  ##  ##  ##  ##  ##  ##  ##  ##  ##  ##  ##  ##  ##  ##  ##  ##  ##  ##  ##  ##  ##  ##  ##  ##  ##  ##  ##  ##  ##  ##  ##  ##  ##  ##  ##  ##  ##  ##  ##  ##  ##  ##  ##  ##  ##  ##  ##  ##  4  10  7  7  10  6  3  5  6  3  11  7  8  --  4  8  5  5  6  4  6  5  5  5  6  7  5  4  5  5  6  4  7  4  5  6  10  7  6  5  6  6  5  8  8  7  8  9  8  10 [2299_16]235	: ##  ##  ##  ##  ##  ##  ##  ##  ##  ##  ##  ##  ##  ##  ##  ##  ##  ##  ##  ##  ##  ##  ##  ##  ##  ##  ##  ##  ##  ##  ##  ##  ##  ##  ##  ##  ##  ##  ##  ##  ##  ##  ##  ##  ##  ##  ##  ##  ##  ##  ##  ##  ##  ##  ##  ##  ##  ##  ##  ##  ##  ##  ##  ##  ##  ##  ##  ##  ##  ##  ##  ##  ##  ##  ##  ##  ##  ##  ##  ##  ##  ##  ##  ##  ##  ##  ##  ##  ##  ##  ##  ##  ##  ##  ##  ##  ##  ##  ##  ##  ##  ##  ##  ##  ##  ##  ##  ##  ##  ##  ##  ##  ##  ##  ##  ##  ##  ##  ##  ##  ##  ##  ##  ##  ##  ##  ##  ##  ##  ##  ##  ##  ##  ##  ##  ##  ##  ##  ##  ##  ##  ##  ##  ##  ##  ##  ##  ##  ##  ##  ##  ##  ##  ##  ##  ##  ##  ##  ##  ##  ##  ##  ##  ##  ##  ##  ##  ##  ##  ##  ##  ##  ##  ##  ##  ##  ##  ##  ##  ##  ##  ##  ##  ##  ##  ##  ##  ##  ##  ##  ##  ##  ##  ##  ##  ##  ##  ##  ##  ##  ##  ##  ##  ##  ##  ##  ##  ##  ##  ##  ##  ##  ##  ##  ##  ##  ##  ##  ##  ##  4  11  7  8  8  7  4  6  6  3  11  8  8  4  --  8  6  5  6  4  6  5  5  6  6  7  5  4  5  5  6  4  7  4  6  6  10  7  7  5  6  6  5  8  9  8  9  9  9  11 [2300_16]236	: ##  ##  ##  ##  ##  ##  ##  ##  ##  ##  ##  ##  ##  ##  ##  ##  ##  ##  ##  ##  ##  ##  ##  ##  ##  ##  ##  ##  ##  ##  ##  ##  ##  ##  ##  ##  ##  ##  ##  ##  ##  ##  ##  ##  ##  ##  ##  ##  ##  ##  ##  ##  ##  ##  ##  ##  ##  ##  ##  ##  ##  ##  ##  ##  ##  ##  ##  ##  ##  ##  ##  ##  ##  ##  ##  ##  ##  ##  ##  ##  ##  ##  ##  ##  ##  ##  ##  ##  ##  ##  ##  ##  ##  ##  ##  ##  ##  ##  ##  ##  ##  ##  ##  ##  ##  ##  ##  ##  ##  ##  ##  ##  ##  ##  ##  ##  ##  ##  ##  ##  ##  ##  ##  ##  ##  ##  ##  ##  ##  ##  ##  ##  ##  ##  ##  ##  ##  ##  ##  ##  ##  ##  ##  ##  ##  ##  ##  ##  ##  ##  ##  ##  ##  ##  ##  ##  ##  ##  ##  ##  ##  ##  ##  ##  ##  ##  ##  ##  ##  ##  ##  ##  ##  ##  ##  ##  ##  ##  ##  ##  ##  ##  ##  ##  ##  ##  ##  ##  ##  ##  ##  ##  ##  ##  ##  ##  ##  ##  ##  ##  ##  ##  ##  ##  ##  ##  ##  ##  ##  ##  ##  ##  ##  ##  ##  ##  ##  ##  ##  ##  6  6  4  3  7  6  6  6  8  5  6  5  8  8  8  --  6  7  4  6  8  7  7  8  6  8  5  6  5  7  6  6  9  6  6  8  11  9  9  7  8  8  5  5  4  6  6  6  6  9 [2303_16]237	: ##  ##  ##  ##  ##  ##  ##  ##  ##  ##  ##  ##  ##  ##  ##  ##  ##  ##  ##  ##  ##  ##  ##  ##  ##  ##  ##  ##  ##  ##  ##  ##  ##  ##  ##  ##  ##  ##  ##  ##  ##  ##  ##  ##  ##  ##  ##  ##  ##  ##  ##  ##  ##  ##  ##  ##  ##  ##  ##  ##  ##  ##  ##  ##  ##  ##  ##  ##  ##  ##  ##  ##  ##  ##  ##  ##  ##  ##  ##  ##  ##  ##  ##  ##  ##  ##  ##  ##  ##  ##  ##  ##  ##  ##  ##  ##  ##  ##  ##  ##  ##  ##  ##  ##  ##  ##  ##  ##  ##  ##  ##  ##  ##  ##  ##  ##  ##  ##  ##  ##  ##  ##  ##  ##  ##  ##  ##  ##  ##  ##  ##  ##  ##  ##  ##  ##  ##  ##  ##  ##  ##  ##  ##  ##  ##  ##  ##  ##  ##  ##  ##  ##  ##  ##  ##  ##  ##  ##  ##  ##  ##  ##  ##  ##  ##  ##  ##  ##  ##  ##  ##  ##  ##  ##  ##  ##  ##  ##  ##  ##  ##  ##  ##  ##  ##  ##  ##  ##  ##  ##  ##  ##  ##  ##  ##  ##  ##  ##  ##  ##  ##  ##  ##  ##  ##  ##  ##  ##  ##  ##  ##  ##  ##  ##  ##  ##  ##  ##  ##  ##  4  7  5  4  8  4  2  4  6  3  9  2  8  5  6  6  --  5  4  4  6  5  5  4  6  5  5  4  5  5  6  4  7  4  4  6  7  7  5  5  6  6  5  6  5  4  5  7  5  7 [2305_16]238	: ##  ##  ##  ##  ##  ##  ##  ##  ##  ##  ##  ##  ##  ##  ##  ##  ##  ##  ##  ##  ##  ##  ##  ##  ##  ##  ##  ##  ##  ##  ##  ##  ##  ##  ##  ##  ##  ##  ##  ##  ##  ##  ##  ##  ##  ##  ##  ##  ##  ##  ##  ##  ##  ##  ##  ##  ##  ##  ##  ##  ##  ##  ##  ##  ##  ##  ##  ##  ##  ##  ##  ##  ##  ##  ##  ##  ##  ##  ##  ##  ##  ##  ##  ##  ##  ##  ##  ##  ##  ##  ##  ##  ##  ##  ##  ##  ##  ##  ##  ##  ##  ##  ##  ##  ##  ##  ##  ##  ##  ##  ##  ##  ##  ##  ##  ##  ##  ##  ##  ##  ##  ##  ##  ##  ##  ##  ##  ##  ##  ##  ##  ##  ##  ##  ##  ##  ##  ##  ##  ##  ##  ##  ##  ##  ##  ##  ##  ##  ##  ##  ##  ##  ##  ##  ##  ##  ##  ##  ##  ##  ##  ##  ##  ##  ##  ##  ##  ##  ##  ##  ##  ##  ##  ##  ##  ##  ##  ##  ##  ##  ##  ##  ##  ##  ##  ##  ##  ##  ##  ##  ##  ##  ##  ##  ##  ##  ##  ##  ##  ##  ##  ##  ##  ##  ##  ##  ##  ##  ##  ##  ##  ##  ##  ##  ##  ##  ##  ##  ##  ##  1  10  6  7  7  6  3  5  5  2  10  7  5  5  5  7  5  --  5  3  5  4  2  5  5  4  4  3  4  4  5  3  6  3  4  5  9  6  6  4  5  5  4  7  8  7  8  8  8  10 [2306_16]239	: ##  ##  ##  ##  ##  ##  ##  ##  ##  ##  ##  ##  ##  ##  ##  ##  ##  ##  ##  ##  ##  ##  ##  ##  ##  ##  ##  ##  ##  ##  ##  ##  ##  ##  ##  ##  ##  ##  ##  ##  ##  ##  ##  ##  ##  ##  ##  ##  ##  ##  ##  ##  ##  ##  ##  ##  ##  ##  ##  ##  ##  ##  ##  ##  ##  ##  ##  ##  ##  ##  ##  ##  ##  ##  ##  ##  ##  ##  ##  ##  ##  ##  ##  ##  ##  ##  ##  ##  ##  ##  ##  ##  ##  ##  ##  ##  ##  ##  ##  ##  ##  ##  ##  ##  ##  ##  ##  ##  ##  ##  ##  ##  ##  ##  ##  ##  ##  ##  ##  ##  ##  ##  ##  ##  ##  ##  ##  ##  ##  ##  ##  ##  ##  ##  ##  ##  ##  ##  ##  ##  ##  ##  ##  ##  ##  ##  ##  ##  ##  ##  ##  ##  ##  ##  ##  ##  ##  ##  ##  ##  ##  ##  ##  ##  ##  ##  ##  ##  ##  ##  ##  ##  ##  ##  ##  ##  ##  ##  ##  ##  ##  ##  ##  ##  ##  ##  ##  ##  ##  ##  ##  ##  ##  ##  ##  ##  ##  ##  ##  ##  ##  ##  ##  ##  ##  ##  ##  ##  ##  ##  ##  ##  ##  ##  ##  ##  ##  ##  ##  ##  4  7  3  4  6  4  4  4  6  3  7  4  8  6  6  4  4  5  --  4  6  5  5  6  6  6  3  4  3  5  6  4  7  4  6  6  9  7  7  5  6  6  3  4  5  4  5  5  5  7 [2307_16]240	: ##  ##  ##  ##  ##  ##  ##  ##  ##  ##  ##  ##  ##  ##  ##  ##  ##  ##  ##  ##  ##  ##  ##  ##  ##  ##  ##  ##  ##  ##  ##  ##  ##  ##  ##  ##  ##  ##  ##  ##  ##  ##  ##  ##  ##  ##  ##  ##  ##  ##  ##  ##  ##  ##  ##  ##  ##  ##  ##  ##  ##  ##  ##  ##  ##  ##  ##  ##  ##  ##  ##  ##  ##  ##  ##  ##  ##  ##  ##  ##  ##  ##  ##  ##  ##  ##  ##  ##  ##  ##  ##  ##  ##  ##  ##  ##  ##  ##  ##  ##  ##  ##  ##  ##  ##  ##  ##  ##  ##  ##  ##  ##  ##  ##  ##  ##  ##  ##  ##  ##  ##  ##  ##  ##  ##  ##  ##  ##  ##  ##  ##  ##  ##  ##  ##  ##  ##  ##  ##  ##  ##  ##  ##  ##  ##  ##  ##  ##  ##  ##  ##  ##  ##  ##  ##  ##  ##  ##  ##  ##  ##  ##  ##  ##  ##  ##  ##  ##  ##  ##  ##  ##  ##  ##  ##  ##  ##  ##  ##  ##  ##  ##  ##  ##  ##  ##  ##  ##  ##  ##  ##  ##  ##  ##  ##  ##  ##  ##  ##  ##  ##  ##  ##  ##  ##  ##  ##  ##  ##  ##  ##  ##  ##  ##  ##  ##  ##  ##  ##  ##  2  9  5  6  8  5  2  4  4  1  9  6  6  4  4  6  4  3  4  --  4  3  3  4  4  5  3  2  1  3  4  2  5  2  4  4  8  5  5  3  4  4  3  6  7  6  7  7  7  7 [2308_16]241	: ##  ##  ##  ##  ##  ##  ##  ##  ##  ##  ##  ##  ##  ##  ##  ##  ##  ##  ##  ##  ##  ##  ##  ##  ##  ##  ##  ##  ##  ##  ##  ##  ##  ##  ##  ##  ##  ##  ##  ##  ##  ##  ##  ##  ##  ##  ##  ##  ##  ##  ##  ##  ##  ##  ##  ##  ##  ##  ##  ##  ##  ##  ##  ##  ##  ##  ##  ##  ##  ##  ##  ##  ##  ##  ##  ##  ##  ##  ##  ##  ##  ##  ##  ##  ##  ##  ##  ##  ##  ##  ##  ##  ##  ##  ##  ##  ##  ##  ##  ##  ##  ##  ##  ##  ##  ##  ##  ##  ##  ##  ##  ##  ##  ##  ##  ##  ##  ##  ##  ##  ##  ##  ##  ##  ##  ##  ##  ##  ##  ##  ##  ##  ##  ##  ##  ##  ##  ##  ##  ##  ##  ##  ##  ##  ##  ##  ##  ##  ##  ##  ##  ##  ##  ##  ##  ##  ##  ##  ##  ##  ##  ##  ##  ##  ##  ##  ##  ##  ##  ##  ##  ##  ##  ##  ##  ##  ##  ##  ##  ##  ##  ##  ##  ##  ##  ##  ##  ##  ##  ##  ##  ##  ##  ##  ##  ##  ##  ##  ##  ##  ##  ##  ##  ##  ##  ##  ##  ##  ##  ##  ##  ##  ##  ##  ##  ##  ##  ##  ##  ##  4  9  7  8  10  7  4  6  6  3  11  8  8  6  6  8  6  5  6  4  --  5  5  6  6  4  5  4  5  5  6  4  7  4  6  2  10  1  7  5  6  6  5  8  9  8  9  7  9  7 [2309_16]242	: ##  ##  ##  ##  ##  ##  ##  ##  ##  ##  ##  ##  ##  ##  ##  ##  ##  ##  ##  ##  ##  ##  ##  ##  ##  ##  ##  ##  ##  ##  ##  ##  ##  ##  ##  ##  ##  ##  ##  ##  ##  ##  ##  ##  ##  ##  ##  ##  ##  ##  ##  ##  ##  ##  ##  ##  ##  ##  ##  ##  ##  ##  ##  ##  ##  ##  ##  ##  ##  ##  ##  ##  ##  ##  ##  ##  ##  ##  ##  ##  ##  ##  ##  ##  ##  ##  ##  ##  ##  ##  ##  ##  ##  ##  ##  ##  ##  ##  ##  ##  ##  ##  ##  ##  ##  ##  ##  ##  ##  ##  ##  ##  ##  ##  ##  ##  ##  ##  ##  ##  ##  ##  ##  ##  ##  ##  ##  ##  ##  ##  ##  ##  ##  ##  ##  ##  ##  ##  ##  ##  ##  ##  ##  ##  ##  ##  ##  ##  ##  ##  ##  ##  ##  ##  ##  ##  ##  ##  ##  ##  ##  ##  ##  ##  ##  ##  ##  ##  ##  ##  ##  ##  ##  ##  ##  ##  ##  ##  ##  ##  ##  ##  ##  ##  ##  ##  ##  ##  ##  ##  ##  ##  ##  ##  ##  ##  ##  ##  ##  ##  ##  ##  ##  ##  ##  ##  ##  ##  ##  ##  ##  ##  ##  ##  ##  ##  ##  ##  ##  ##  3  10  6  7  9  4  3  5  5  2  10  7  7  5  5  7  5  4  5  3  5  --  2  5  5  4  4  1  4  2  5  3  6  3  5  3  9  6  6  2  5  3  4  7  8  7  8  8  8  10 [2311_16]243	: ##  ##  ##  ##  ##  ##  ##  ##  ##  ##  ##  ##  ##  ##  ##  ##  ##  ##  ##  ##  ##  ##  ##  ##  ##  ##  ##  ##  ##  ##  ##  ##  ##  ##  ##  ##  ##  ##  ##  ##  ##  ##  ##  ##  ##  ##  ##  ##  ##  ##  ##  ##  ##  ##  ##  ##  ##  ##  ##  ##  ##  ##  ##  ##  ##  ##  ##  ##  ##  ##  ##  ##  ##  ##  ##  ##  ##  ##  ##  ##  ##  ##  ##  ##  ##  ##  ##  ##  ##  ##  ##  ##  ##  ##  ##  ##  ##  ##  ##  ##  ##  ##  ##  ##  ##  ##  ##  ##  ##  ##  ##  ##  ##  ##  ##  ##  ##  ##  ##  ##  ##  ##  ##  ##  ##  ##  ##  ##  ##  ##  ##  ##  ##  ##  ##  ##  ##  ##  ##  ##  ##  ##  ##  ##  ##  ##  ##  ##  ##  ##  ##  ##  ##  ##  ##  ##  ##  ##  ##  ##  ##  ##  ##  ##  ##  ##  ##  ##  ##  ##  ##  ##  ##  ##  ##  ##  ##  ##  ##  ##  ##  ##  ##  ##  ##  ##  ##  ##  ##  ##  ##  ##  ##  ##  ##  ##  ##  ##  ##  ##  ##  ##  ##  ##  ##  ##  ##  ##  ##  ##  ##  ##  ##  ##  ##  ##  ##  ##  ##  ##  1  10  6  7  7  4  3  5  5  2  10  7  5  5  5  7  5  2  5  3  5  2  --  5  5  2  4  1  4  2  5  3  6  3  4  3  9  6  6  2  5  3  4  7  8  7  8  8  8  10 [2315_16]244	: ##  ##  ##  ##  ##  ##  ##  ##  ##  ##  ##  ##  ##  ##  ##  ##  ##  ##  ##  ##  ##  ##  ##  ##  ##  ##  ##  ##  ##  ##  ##  ##  ##  ##  ##  ##  ##  ##  ##  ##  ##  ##  ##  ##  ##  ##  ##  ##  ##  ##  ##  ##  ##  ##  ##  ##  ##  ##  ##  ##  ##  ##  ##  ##  ##  ##  ##  ##  ##  ##  ##  ##  ##  ##  ##  ##  ##  ##  ##  ##  ##  ##  ##  ##  ##  ##  ##  ##  ##  ##  ##  ##  ##  ##  ##  ##  ##  ##  ##  ##  ##  ##  ##  ##  ##  ##  ##  ##  ##  ##  ##  ##  ##  ##  ##  ##  ##  ##  ##  ##  ##  ##  ##  ##  ##  ##  ##  ##  ##  ##  ##  ##  ##  ##  ##  ##  ##  ##  ##  ##  ##  ##  ##  ##  ##  ##  ##  ##  ##  ##  ##  ##  ##  ##  ##  ##  ##  ##  ##  ##  ##  ##  ##  ##  ##  ##  ##  ##  ##  ##  ##  ##  ##  ##  ##  ##  ##  ##  ##  ##  ##  ##  ##  ##  ##  ##  ##  ##  ##  ##  ##  ##  ##  ##  ##  ##  ##  ##  ##  ##  ##  ##  ##  ##  ##  ##  ##  ##  ##  ##  ##  ##  ##  ##  ##  ##  ##  ##  ##  ##  4  9  7  6  10  5  2  4  6  3  11  6  8  5  6  8  4  5  6  4  6  5  5  --  6  6  5  4  5  5  6  4  7  4  4  6  10  7  5  5  6  6  5  8  7  6  7  9  7  8 [2316_16]245	: ##  ##  ##  ##  ##  ##  ##  ##  ##  ##  ##  ##  ##  ##  ##  ##  ##  ##  ##  ##  ##  ##  ##  ##  ##  ##  ##  ##  ##  ##  ##  ##  ##  ##  ##  ##  ##  ##  ##  ##  ##  ##  ##  ##  ##  ##  ##  ##  ##  ##  ##  ##  ##  ##  ##  ##  ##  ##  ##  ##  ##  ##  ##  ##  ##  ##  ##  ##  ##  ##  ##  ##  ##  ##  ##  ##  ##  ##  ##  ##  ##  ##  ##  ##  ##  ##  ##  ##  ##  ##  ##  ##  ##  ##  ##  ##  ##  ##  ##  ##  ##  ##  ##  ##  ##  ##  ##  ##  ##  ##  ##  ##  ##  ##  ##  ##  ##  ##  ##  ##  ##  ##  ##  ##  ##  ##  ##  ##  ##  ##  ##  ##  ##  ##  ##  ##  ##  ##  ##  ##  ##  ##  ##  ##  ##  ##  ##  ##  ##  ##  ##  ##  ##  ##  ##  ##  ##  ##  ##  ##  ##  ##  ##  ##  ##  ##  ##  ##  ##  ##  ##  ##  ##  ##  ##  ##  ##  ##  ##  ##  ##  ##  ##  ##  ##  ##  ##  ##  ##  ##  ##  ##  ##  ##  ##  ##  ##  ##  ##  ##  ##  ##  ##  ##  ##  ##  ##  ##  ##  ##  ##  ##  ##  ##  ##  ##  ##  ##  ##  ##  4  9  7  6  10  7  4  6  6  3  11  8  8  6  6  6  6  5  6  4  6  5  5  6  --  7  5  4  5  3  4  4  7  4  6  6  10  7  7  5  2  6  5  8  9  8  9  9  9  11 [2325_16]246	: ##  ##  ##  ##  ##  ##  ##  ##  ##  ##  ##  ##  ##  ##  ##  ##  ##  ##  ##  ##  ##  ##  ##  ##  ##  ##  ##  ##  ##  ##  ##  ##  ##  ##  ##  ##  ##  ##  ##  ##  ##  ##  ##  ##  ##  ##  ##  ##  ##  ##  ##  ##  ##  ##  ##  ##  ##  ##  ##  ##  ##  ##  ##  ##  ##  ##  ##  ##  ##  ##  ##  ##  ##  ##  ##  ##  ##  ##  ##  ##  ##  ##  ##  ##  ##  ##  ##  ##  ##  ##  ##  ##  ##  ##  ##  ##  ##  ##  ##  ##  ##  ##  ##  ##  ##  ##  ##  ##  ##  ##  ##  ##  ##  ##  ##  ##  ##  ##  ##  ##  ##  ##  ##  ##  ##  ##  ##  ##  ##  ##  ##  ##  ##  ##  ##  ##  ##  ##  ##  ##  ##  ##  ##  ##  ##  ##  ##  ##  ##  ##  ##  ##  ##  ##  ##  ##  ##  ##  ##  ##  ##  ##  ##  ##  ##  ##  ##  ##  ##  ##  ##  ##  ##  ##  ##  ##  ##  ##  ##  ##  ##  ##  ##  ##  ##  ##  ##  ##  ##  ##  ##  ##  ##  ##  ##  ##  ##  ##  ##  ##  ##  ##  ##  ##  ##  ##  ##  ##  ##  ##  ##  ##  ##  ##  ##  ##  ##  ##  ##  ##  3  9  7  8  8  5  5  6  7  4  10  6  7  7  7  8  5  4  6  5  4  4  2  6  7  --  5  3  5  4  7  5  7  5  6  2  8  5  8  4  7  5  5  8  9  8  9  8  9  8 [2330_16]247	: ##  ##  ##  ##  ##  ##  ##  ##  ##  ##  ##  ##  ##  ##  ##  ##  ##  ##  ##  ##  ##  ##  ##  ##  ##  ##  ##  ##  ##  ##  ##  ##  ##  ##  ##  ##  ##  ##  ##  ##  ##  ##  ##  ##  ##  ##  ##  ##  ##  ##  ##  ##  ##  ##  ##  ##  ##  ##  ##  ##  ##  ##  ##  ##  ##  ##  ##  ##  ##  ##  ##  ##  ##  ##  ##  ##  ##  ##  ##  ##  ##  ##  ##  ##  ##  ##  ##  ##  ##  ##  ##  ##  ##  ##  ##  ##  ##  ##  ##  ##  ##  ##  ##  ##  ##  ##  ##  ##  ##  ##  ##  ##  ##  ##  ##  ##  ##  ##  ##  ##  ##  ##  ##  ##  ##  ##  ##  ##  ##  ##  ##  ##  ##  ##  ##  ##  ##  ##  ##  ##  ##  ##  ##  ##  ##  ##  ##  ##  ##  ##  ##  ##  ##  ##  ##  ##  ##  ##  ##  ##  ##  ##  ##  ##  ##  ##  ##  ##  ##  ##  ##  ##  ##  ##  ##  ##  ##  ##  ##  ##  ##  ##  ##  ##  ##  ##  ##  ##  ##  ##  ##  ##  ##  ##  ##  ##  ##  ##  ##  ##  ##  ##  ##  ##  ##  ##  ##  ##  ##  ##  ##  ##  ##  ##  ##  ##  ##  ##  ##  ##  3  8  4  5  5  4  3  3  5  2  8  5  7  5  5  5  5  4  3  3  5  4  4  5  5  5  --  3  2  4  5  3  6  3  5  5  9  6  6  4  5  5  2  5  6  5  6  6  6  8 [2339_16]248	: ##  ##  ##  ##  ##  ##  ##  ##  ##  ##  ##  ##  ##  ##  ##  ##  ##  ##  ##  ##  ##  ##  ##  ##  ##  ##  ##  ##  ##  ##  ##  ##  ##  ##  ##  ##  ##  ##  ##  ##  ##  ##  ##  ##  ##  ##  ##  ##  ##  ##  ##  ##  ##  ##  ##  ##  ##  ##  ##  ##  ##  ##  ##  ##  ##  ##  ##  ##  ##  ##  ##  ##  ##  ##  ##  ##  ##  ##  ##  ##  ##  ##  ##  ##  ##  ##  ##  ##  ##  ##  ##  ##  ##  ##  ##  ##  ##  ##  ##  ##  ##  ##  ##  ##  ##  ##  ##  ##  ##  ##  ##  ##  ##  ##  ##  ##  ##  ##  ##  ##  ##  ##  ##  ##  ##  ##  ##  ##  ##  ##  ##  ##  ##  ##  ##  ##  ##  ##  ##  ##  ##  ##  ##  ##  ##  ##  ##  ##  ##  ##  ##  ##  ##  ##  ##  ##  ##  ##  ##  ##  ##  ##  ##  ##  ##  ##  ##  ##  ##  ##  ##  ##  ##  ##  ##  ##  ##  ##  ##  ##  ##  ##  ##  ##  ##  ##  ##  ##  ##  ##  ##  ##  ##  ##  ##  ##  ##  ##  ##  ##  ##  ##  ##  ##  ##  ##  ##  ##  ##  ##  ##  ##  ##  ##  ##  ##  ##  ##  ##  ##  2  9  5  6  8  3  2  4  4  1  9  6  6  4  4  6  4  3  4  2  4  1  1  4  4  3  3  --  3  1  4  2  5  2  4  2  8  5  5  1  4  2  3  6  7  6  7  7  7  9 [2340_16]249	: ##  ##  ##  ##  ##  ##  ##  ##  ##  ##  ##  ##  ##  ##  ##  ##  ##  ##  ##  ##  ##  ##  ##  ##  ##  ##  ##  ##  ##  ##  ##  ##  ##  ##  ##  ##  ##  ##  ##  ##  ##  ##  ##  ##  ##  ##  ##  ##  ##  ##  ##  ##  ##  ##  ##  ##  ##  ##  ##  ##  ##  ##  ##  ##  ##  ##  ##  ##  ##  ##  ##  ##  ##  ##  ##  ##  ##  ##  ##  ##  ##  ##  ##  ##  ##  ##  ##  ##  ##  ##  ##  ##  ##  ##  ##  ##  ##  ##  ##  ##  ##  ##  ##  ##  ##  ##  ##  ##  ##  ##  ##  ##  ##  ##  ##  ##  ##  ##  ##  ##  ##  ##  ##  ##  ##  ##  ##  ##  ##  ##  ##  ##  ##  ##  ##  ##  ##  ##  ##  ##  ##  ##  ##  ##  ##  ##  ##  ##  ##  ##  ##  ##  ##  ##  ##  ##  ##  ##  ##  ##  ##  ##  ##  ##  ##  ##  ##  ##  ##  ##  ##  ##  ##  ##  ##  ##  ##  ##  ##  ##  ##  ##  ##  ##  ##  ##  ##  ##  ##  ##  ##  ##  ##  ##  ##  ##  ##  ##  ##  ##  ##  ##  ##  ##  ##  ##  ##  ##  ##  ##  ##  ##  ##  ##  ##  ##  ##  ##  ##  ##  3  8  4  5  7  3  3  3  5  2  8  5  7  5  5  5  5  4  3  1  5  4  4  5  5  5  2  3  --  4  5  3  6  3  5  5  8  6  6  4  5  5  2  5  6  5  6  6  6  6 [2344_16]250	: ##  ##  ##  ##  ##  ##  ##  ##  ##  ##  ##  ##  ##  ##  ##  ##  ##  ##  ##  ##  ##  ##  ##  ##  ##  ##  ##  ##  ##  ##  ##  ##  ##  ##  ##  ##  ##  ##  ##  ##  ##  ##  ##  ##  ##  ##  ##  ##  ##  ##  ##  ##  ##  ##  ##  ##  ##  ##  ##  ##  ##  ##  ##  ##  ##  ##  ##  ##  ##  ##  ##  ##  ##  ##  ##  ##  ##  ##  ##  ##  ##  ##  ##  ##  ##  ##  ##  ##  ##  ##  ##  ##  ##  ##  ##  ##  ##  ##  ##  ##  ##  ##  ##  ##  ##  ##  ##  ##  ##  ##  ##  ##  ##  ##  ##  ##  ##  ##  ##  ##  ##  ##  ##  ##  ##  ##  ##  ##  ##  ##  ##  ##  ##  ##  ##  ##  ##  ##  ##  ##  ##  ##  ##  ##  ##  ##  ##  ##  ##  ##  ##  ##  ##  ##  ##  ##  ##  ##  ##  ##  ##  ##  ##  ##  ##  ##  ##  ##  ##  ##  ##  ##  ##  ##  ##  ##  ##  ##  ##  ##  ##  ##  ##  ##  ##  ##  ##  ##  ##  ##  ##  ##  ##  ##  ##  ##  ##  ##  ##  ##  ##  ##  ##  ##  ##  ##  ##  ##  ##  ##  ##  ##  ##  ##  ##  ##  ##  ##  ##  ##  3  10  6  7  9  4  3  5  5  2  10  7  7  5  5  7  5  4  5  3  5  2  2  5  3  4  4  1  4  --  5  3  6  3  5  3  9  6  6  2  3  3  4  7  8  7  8  8  8  10 [2345_16]251	: ##  ##  ##  ##  ##  ##  ##  ##  ##  ##  ##  ##  ##  ##  ##  ##  ##  ##  ##  ##  ##  ##  ##  ##  ##  ##  ##  ##  ##  ##  ##  ##  ##  ##  ##  ##  ##  ##  ##  ##  ##  ##  ##  ##  ##  ##  ##  ##  ##  ##  ##  ##  ##  ##  ##  ##  ##  ##  ##  ##  ##  ##  ##  ##  ##  ##  ##  ##  ##  ##  ##  ##  ##  ##  ##  ##  ##  ##  ##  ##  ##  ##  ##  ##  ##  ##  ##  ##  ##  ##  ##  ##  ##  ##  ##  ##  ##  ##  ##  ##  ##  ##  ##  ##  ##  ##  ##  ##  ##  ##  ##  ##  ##  ##  ##  ##  ##  ##  ##  ##  ##  ##  ##  ##  ##  ##  ##  ##  ##  ##  ##  ##  ##  ##  ##  ##  ##  ##  ##  ##  ##  ##  ##  ##  ##  ##  ##  ##  ##  ##  ##  ##  ##  ##  ##  ##  ##  ##  ##  ##  ##  ##  ##  ##  ##  ##  ##  ##  ##  ##  ##  ##  ##  ##  ##  ##  ##  ##  ##  ##  ##  ##  ##  ##  ##  ##  ##  ##  ##  ##  ##  ##  ##  ##  ##  ##  ##  ##  ##  ##  ##  ##  ##  ##  ##  ##  ##  ##  ##  ##  ##  ##  ##  ##  ##  ##  ##  ##  ##  ##  4  9  7  6  10  6  4  6  6  3  10  8  8  6  6  6  6  5  6  4  6  5  5  6  4  7  5  4  5  5  --  4  7  4  6  6  9  7  7  5  6  6  5  8  9  8  9  9  9  11 [2356_16]252	: ##  ##  ##  ##  ##  ##  ##  ##  ##  ##  ##  ##  ##  ##  ##  ##  ##  ##  ##  ##  ##  ##  ##  ##  ##  ##  ##  ##  ##  ##  ##  ##  ##  ##  ##  ##  ##  ##  ##  ##  ##  ##  ##  ##  ##  ##  ##  ##  ##  ##  ##  ##  ##  ##  ##  ##  ##  ##  ##  ##  ##  ##  ##  ##  ##  ##  ##  ##  ##  ##  ##  ##  ##  ##  ##  ##  ##  ##  ##  ##  ##  ##  ##  ##  ##  ##  ##  ##  ##  ##  ##  ##  ##  ##  ##  ##  ##  ##  ##  ##  ##  ##  ##  ##  ##  ##  ##  ##  ##  ##  ##  ##  ##  ##  ##  ##  ##  ##  ##  ##  ##  ##  ##  ##  ##  ##  ##  ##  ##  ##  ##  ##  ##  ##  ##  ##  ##  ##  ##  ##  ##  ##  ##  ##  ##  ##  ##  ##  ##  ##  ##  ##  ##  ##  ##  ##  ##  ##  ##  ##  ##  ##  ##  ##  ##  ##  ##  ##  ##  ##  ##  ##  ##  ##  ##  ##  ##  ##  ##  ##  ##  ##  ##  ##  ##  ##  ##  ##  ##  ##  ##  ##  ##  ##  ##  ##  ##  ##  ##  ##  ##  ##  ##  ##  ##  ##  ##  ##  ##  ##  ##  ##  ##  ##  ##  ##  ##  ##  ##  ##  2  9  5  6  8  5  2  4  4  1  9  6  6  4  4  6  4  3  4  2  4  3  3  4  4  5  3  2  3  3  4  --  5  2  4  4  8  5  5  3  4  4  3  6  7  6  7  7  7  9 [2357_16]253	: ##  ##  ##  ##  ##  ##  ##  ##  ##  ##  ##  ##  ##  ##  ##  ##  ##  ##  ##  ##  ##  ##  ##  ##  ##  ##  ##  ##  ##  ##  ##  ##  ##  ##  ##  ##  ##  ##  ##  ##  ##  ##  ##  ##  ##  ##  ##  ##  ##  ##  ##  ##  ##  ##  ##  ##  ##  ##  ##  ##  ##  ##  ##  ##  ##  ##  ##  ##  ##  ##  ##  ##  ##  ##  ##  ##  ##  ##  ##  ##  ##  ##  ##  ##  ##  ##  ##  ##  ##  ##  ##  ##  ##  ##  ##  ##  ##  ##  ##  ##  ##  ##  ##  ##  ##  ##  ##  ##  ##  ##  ##  ##  ##  ##  ##  ##  ##  ##  ##  ##  ##  ##  ##  ##  ##  ##  ##  ##  ##  ##  ##  ##  ##  ##  ##  ##  ##  ##  ##  ##  ##  ##  ##  ##  ##  ##  ##  ##  ##  ##  ##  ##  ##  ##  ##  ##  ##  ##  ##  ##  ##  ##  ##  ##  ##  ##  ##  ##  ##  ##  ##  ##  ##  ##  ##  ##  ##  ##  ##  ##  ##  ##  ##  ##  ##  ##  ##  ##  ##  ##  ##  ##  ##  ##  ##  ##  ##  ##  ##  ##  ##  ##  ##  ##  ##  ##  ##  ##  ##  ##  ##  ##  ##  ##  ##  ##  ##  ##  ##  ##  5  12  8  9  11  8  5  7  7  4  12  9  9  7  7  9  7  6  7  5  7  6  6  7  7  7  6  5  6  6  7  5  --  5  7  7  11  8  8  6  7  7  6  9  8  9  8  10  10  10 [2358_16]254	: ##  ##  ##  ##  ##  ##  ##  ##  ##  ##  ##  ##  ##  ##  ##  ##  ##  ##  ##  ##  ##  ##  ##  ##  ##  ##  ##  ##  ##  ##  ##  ##  ##  ##  ##  ##  ##  ##  ##  ##  ##  ##  ##  ##  ##  ##  ##  ##  ##  ##  ##  ##  ##  ##  ##  ##  ##  ##  ##  ##  ##  ##  ##  ##  ##  ##  ##  ##  ##  ##  ##  ##  ##  ##  ##  ##  ##  ##  ##  ##  ##  ##  ##  ##  ##  ##  ##  ##  ##  ##  ##  ##  ##  ##  ##  ##  ##  ##  ##  ##  ##  ##  ##  ##  ##  ##  ##  ##  ##  ##  ##  ##  ##  ##  ##  ##  ##  ##  ##  ##  ##  ##  ##  ##  ##  ##  ##  ##  ##  ##  ##  ##  ##  ##  ##  ##  ##  ##  ##  ##  ##  ##  ##  ##  ##  ##  ##  ##  ##  ##  ##  ##  ##  ##  ##  ##  ##  ##  ##  ##  ##  ##  ##  ##  ##  ##  ##  ##  ##  ##  ##  ##  ##  ##  ##  ##  ##  ##  ##  ##  ##  ##  ##  ##  ##  ##  ##  ##  ##  ##  ##  ##  ##  ##  ##  ##  ##  ##  ##  ##  ##  ##  ##  ##  ##  ##  ##  ##  ##  ##  ##  ##  ##  ##  ##  ##  ##  ##  ##  ##  2  9  5  6  8  5  2  4  4  1  9  6  6  4  4  6  4  3  4  2  4  3  3  4  4  5  3  2  3  3  4  2  5  --  4  4  8  5  5  3  4  4  3  6  7  6  7  7  7  9 [2359_16]255	: ##  ##  ##  ##  ##  ##  ##  ##  ##  ##  ##  ##  ##  ##  ##  ##  ##  ##  ##  ##  ##  ##  ##  ##  ##  ##  ##  ##  ##  ##  ##  ##  ##  ##  ##  ##  ##  ##  ##  ##  ##  ##  ##  ##  ##  ##  ##  ##  ##  ##  ##  ##  ##  ##  ##  ##  ##  ##  ##  ##  ##  ##  ##  ##  ##  ##  ##  ##  ##  ##  ##  ##  ##  ##  ##  ##  ##  ##  ##  ##  ##  ##  ##  ##  ##  ##  ##  ##  ##  ##  ##  ##  ##  ##  ##  ##  ##  ##  ##  ##  ##  ##  ##  ##  ##  ##  ##  ##  ##  ##  ##  ##  ##  ##  ##  ##  ##  ##  ##  ##  ##  ##  ##  ##  ##  ##  ##  ##  ##  ##  ##  ##  ##  ##  ##  ##  ##  ##  ##  ##  ##  ##  ##  ##  ##  ##  ##  ##  ##  ##  ##  ##  ##  ##  ##  ##  ##  ##  ##  ##  ##  ##  ##  ##  ##  ##  ##  ##  ##  ##  ##  ##  ##  ##  ##  ##  ##  ##  ##  ##  ##  ##  ##  ##  ##  ##  ##  ##  ##  ##  ##  ##  ##  ##  ##  ##  ##  ##  ##  ##  ##  ##  ##  ##  ##  ##  ##  ##  ##  ##  ##  ##  ##  ##  ##  ##  ##  ##  ##  ##  3  9  7  6  9  5  2  4  6  3  9  6  5  5  6  6  4  4  6  4  6  5  4  4  6  6  5  4  5  5  6  4  7  4  --  6  10  7  5  5  6  6  5  8  5  6  7  9  7  9 [2361_16]256	: ##  ##  ##  ##  ##  ##  ##  ##  ##  ##  ##  ##  ##  ##  ##  ##  ##  ##  ##  ##  ##  ##  ##  ##  ##  ##  ##  ##  ##  ##  ##  ##  ##  ##  ##  ##  ##  ##  ##  ##  ##  ##  ##  ##  ##  ##  ##  ##  ##  ##  ##  ##  ##  ##  ##  ##  ##  ##  ##  ##  ##  ##  ##  ##  ##  ##  ##  ##  ##  ##  ##  ##  ##  ##  ##  ##  ##  ##  ##  ##  ##  ##  ##  ##  ##  ##  ##  ##  ##  ##  ##  ##  ##  ##  ##  ##  ##  ##  ##  ##  ##  ##  ##  ##  ##  ##  ##  ##  ##  ##  ##  ##  ##  ##  ##  ##  ##  ##  ##  ##  ##  ##  ##  ##  ##  ##  ##  ##  ##  ##  ##  ##  ##  ##  ##  ##  ##  ##  ##  ##  ##  ##  ##  ##  ##  ##  ##  ##  ##  ##  ##  ##  ##  ##  ##  ##  ##  ##  ##  ##  ##  ##  ##  ##  ##  ##  ##  ##  ##  ##  ##  ##  ##  ##  ##  ##  ##  ##  ##  ##  ##  ##  ##  ##  ##  ##  ##  ##  ##  ##  ##  ##  ##  ##  ##  ##  ##  ##  ##  ##  ##  ##  ##  ##  ##  ##  ##  ##  ##  ##  ##  ##  ##  ##  ##  ##  ##  ##  ##  ##  4  9  7  8  10  4  4  6  6  3  11  8  8  6  6  8  6  5  6  4  2  3  3  6  6  2  5  2  5  3  6  4  7  4  6  --  9  3  7  3  6  4  5  8  9  8  9  7  9  7 [2362_16]257	: ##  ##  ##  ##  ##  ##  ##  ##  ##  ##  ##  ##  ##  ##  ##  ##  ##  ##  ##  ##  ##  ##  ##  ##  ##  ##  ##  ##  ##  ##  ##  ##  ##  ##  ##  ##  ##  ##  ##  ##  ##  ##  ##  ##  ##  ##  ##  ##  ##  ##  ##  ##  ##  ##  ##  ##  ##  ##  ##  ##  ##  ##  ##  ##  ##  ##  ##  ##  ##  ##  ##  ##  ##  ##  ##  ##  ##  ##  ##  ##  ##  ##  ##  ##  ##  ##  ##  ##  ##  ##  ##  ##  ##  ##  ##  ##  ##  ##  ##  ##  ##  ##  ##  ##  ##  ##  ##  ##  ##  ##  ##  ##  ##  ##  ##  ##  ##  ##  ##  ##  ##  ##  ##  ##  ##  ##  ##  ##  ##  ##  ##  ##  ##  ##  ##  ##  ##  ##  ##  ##  ##  ##  ##  ##  ##  ##  ##  ##  ##  ##  ##  ##  ##  ##  ##  ##  ##  ##  ##  ##  ##  ##  ##  ##  ##  ##  ##  ##  ##  ##  ##  ##  ##  ##  ##  ##  ##  ##  ##  ##  ##  ##  ##  ##  ##  ##  ##  ##  ##  ##  ##  ##  ##  ##  ##  ##  ##  ##  ##  ##  ##  ##  ##  ##  ##  ##  ##  ##  ##  ##  ##  ##  ##  ##  ##  ##  ##  ##  ##  ##  8  14  11  12  14  9  8  10  10  7  14  10  12  10  10  11  7  9  9  8  10  9  9  10  10  8  9  8  8  9  9  8  11  8  10  9  --  11  11  9  10  9  9  11  13  12  12  13  13  14 [2363_16]258	: ##  ##  ##  ##  ##  ##  ##  ##  ##  ##  ##  ##  ##  ##  ##  ##  ##  ##  ##  ##  ##  ##  ##  ##  ##  ##  ##  ##  ##  ##  ##  ##  ##  ##  ##  ##  ##  ##  ##  ##  ##  ##  ##  ##  ##  ##  ##  ##  ##  ##  ##  ##  ##  ##  ##  ##  ##  ##  ##  ##  ##  ##  ##  ##  ##  ##  ##  ##  ##  ##  ##  ##  ##  ##  ##  ##  ##  ##  ##  ##  ##  ##  ##  ##  ##  ##  ##  ##  ##  ##  ##  ##  ##  ##  ##  ##  ##  ##  ##  ##  ##  ##  ##  ##  ##  ##  ##  ##  ##  ##  ##  ##  ##  ##  ##  ##  ##  ##  ##  ##  ##  ##  ##  ##  ##  ##  ##  ##  ##  ##  ##  ##  ##  ##  ##  ##  ##  ##  ##  ##  ##  ##  ##  ##  ##  ##  ##  ##  ##  ##  ##  ##  ##  ##  ##  ##  ##  ##  ##  ##  ##  ##  ##  ##  ##  ##  ##  ##  ##  ##  ##  ##  ##  ##  ##  ##  ##  ##  ##  ##  ##  ##  ##  ##  ##  ##  ##  ##  ##  ##  ##  ##  ##  ##  ##  ##  ##  ##  ##  ##  ##  ##  ##  ##  ##  ##  ##  ##  ##  ##  ##  ##  ##  ##  ##  ##  ##  ##  ##  ##  5  10  8  9  11  8  5  7  5  4  12  9  9  7  7  9  7  6  7  5  1  6  6  7  7  5  6  5  6  6  7  5  8  5  7  3  11  --  8  6  7  7  6  9  10  9  10  8  10  8 [2365_16]259	: ##  ##  ##  ##  ##  ##  ##  ##  ##  ##  ##  ##  ##  ##  ##  ##  ##  ##  ##  ##  ##  ##  ##  ##  ##  ##  ##  ##  ##  ##  ##  ##  ##  ##  ##  ##  ##  ##  ##  ##  ##  ##  ##  ##  ##  ##  ##  ##  ##  ##  ##  ##  ##  ##  ##  ##  ##  ##  ##  ##  ##  ##  ##  ##  ##  ##  ##  ##  ##  ##  ##  ##  ##  ##  ##  ##  ##  ##  ##  ##  ##  ##  ##  ##  ##  ##  ##  ##  ##  ##  ##  ##  ##  ##  ##  ##  ##  ##  ##  ##  ##  ##  ##  ##  ##  ##  ##  ##  ##  ##  ##  ##  ##  ##  ##  ##  ##  ##  ##  ##  ##  ##  ##  ##  ##  ##  ##  ##  ##  ##  ##  ##  ##  ##  ##  ##  ##  ##  ##  ##  ##  ##  ##  ##  ##  ##  ##  ##  ##  ##  ##  ##  ##  ##  ##  ##  ##  ##  ##  ##  ##  ##  ##  ##  ##  ##  ##  ##  ##  ##  ##  ##  ##  ##  ##  ##  ##  ##  ##  ##  ##  ##  ##  ##  ##  ##  ##  ##  ##  ##  ##  ##  ##  ##  ##  ##  ##  ##  ##  ##  ##  ##  ##  ##  ##  ##  ##  ##  ##  ##  ##  ##  ##  ##  ##  ##  ##  ##  ##  ##  5  10  8  7  11  6  3  5  7  4  12  7  9  6  7  9  5  6  7  5  7  6  6  5  7  8  6  5  6  6  7  5  8  5  5  7  11  8  --  6  7  5  6  9  8  7  8  10  8  10 [2366_16]260	: ##  ##  ##  ##  ##  ##  ##  ##  ##  ##  ##  ##  ##  ##  ##  ##  ##  ##  ##  ##  ##  ##  ##  ##  ##  ##  ##  ##  ##  ##  ##  ##  ##  ##  ##  ##  ##  ##  ##  ##  ##  ##  ##  ##  ##  ##  ##  ##  ##  ##  ##  ##  ##  ##  ##  ##  ##  ##  ##  ##  ##  ##  ##  ##  ##  ##  ##  ##  ##  ##  ##  ##  ##  ##  ##  ##  ##  ##  ##  ##  ##  ##  ##  ##  ##  ##  ##  ##  ##  ##  ##  ##  ##  ##  ##  ##  ##  ##  ##  ##  ##  ##  ##  ##  ##  ##  ##  ##  ##  ##  ##  ##  ##  ##  ##  ##  ##  ##  ##  ##  ##  ##  ##  ##  ##  ##  ##  ##  ##  ##  ##  ##  ##  ##  ##  ##  ##  ##  ##  ##  ##  ##  ##  ##  ##  ##  ##  ##  ##  ##  ##  ##  ##  ##  ##  ##  ##  ##  ##  ##  ##  ##  ##  ##  ##  ##  ##  ##  ##  ##  ##  ##  ##  ##  ##  ##  ##  ##  ##  ##  ##  ##  ##  ##  ##  ##  ##  ##  ##  ##  ##  ##  ##  ##  ##  ##  ##  ##  ##  ##  ##  ##  ##  ##  ##  ##  ##  ##  ##  ##  ##  ##  ##  ##  ##  ##  ##  ##  ##  ##  3  10  6  7  9  4  3  5  5  2  10  7  7  5  5  7  5  4  5  3  5  2  2  5  5  4  4  1  4  2  5  3  6  3  5  3  9  6  6  --  5  3  4  7  8  7  8  8  8  10 [2373_16]261	: ##  ##  ##  ##  ##  ##  ##  ##  ##  ##  ##  ##  ##  ##  ##  ##  ##  ##  ##  ##  ##  ##  ##  ##  ##  ##  ##  ##  ##  ##  ##  ##  ##  ##  ##  ##  ##  ##  ##  ##  ##  ##  ##  ##  ##  ##  ##  ##  ##  ##  ##  ##  ##  ##  ##  ##  ##  ##  ##  ##  ##  ##  ##  ##  ##  ##  ##  ##  ##  ##  ##  ##  ##  ##  ##  ##  ##  ##  ##  ##  ##  ##  ##  ##  ##  ##  ##  ##  ##  ##  ##  ##  ##  ##  ##  ##  ##  ##  ##  ##  ##  ##  ##  ##  ##  ##  ##  ##  ##  ##  ##  ##  ##  ##  ##  ##  ##  ##  ##  ##  ##  ##  ##  ##  ##  ##  ##  ##  ##  ##  ##  ##  ##  ##  ##  ##  ##  ##  ##  ##  ##  ##  ##  ##  ##  ##  ##  ##  ##  ##  ##  ##  ##  ##  ##  ##  ##  ##  ##  ##  ##  ##  ##  ##  ##  ##  ##  ##  ##  ##  ##  ##  ##  ##  ##  ##  ##  ##  ##  ##  ##  ##  ##  ##  ##  ##  ##  ##  ##  ##  ##  ##  ##  ##  ##  ##  ##  ##  ##  ##  ##  ##  ##  ##  ##  ##  ##  ##  ##  ##  ##  ##  ##  ##  ##  ##  ##  ##  ##  ##  4  11  7  8  10  7  4  6  6  3  11  8  8  6  6  8  6  5  6  4  6  5  5  6  2  7  5  4  5  3  6  4  7  4  6  6  10  7  7  5  --  6  5  8  9  8  9  9  9  11 [2374_16]262	: ##  ##  ##  ##  ##  ##  ##  ##  ##  ##  ##  ##  ##  ##  ##  ##  ##  ##  ##  ##  ##  ##  ##  ##  ##  ##  ##  ##  ##  ##  ##  ##  ##  ##  ##  ##  ##  ##  ##  ##  ##  ##  ##  ##  ##  ##  ##  ##  ##  ##  ##  ##  ##  ##  ##  ##  ##  ##  ##  ##  ##  ##  ##  ##  ##  ##  ##  ##  ##  ##  ##  ##  ##  ##  ##  ##  ##  ##  ##  ##  ##  ##  ##  ##  ##  ##  ##  ##  ##  ##  ##  ##  ##  ##  ##  ##  ##  ##  ##  ##  ##  ##  ##  ##  ##  ##  ##  ##  ##  ##  ##  ##  ##  ##  ##  ##  ##  ##  ##  ##  ##  ##  ##  ##  ##  ##  ##  ##  ##  ##  ##  ##  ##  ##  ##  ##  ##  ##  ##  ##  ##  ##  ##  ##  ##  ##  ##  ##  ##  ##  ##  ##  ##  ##  ##  ##  ##  ##  ##  ##  ##  ##  ##  ##  ##  ##  ##  ##  ##  ##  ##  ##  ##  ##  ##  ##  ##  ##  ##  ##  ##  ##  ##  ##  ##  ##  ##  ##  ##  ##  ##  ##  ##  ##  ##  ##  ##  ##  ##  ##  ##  ##  ##  ##  ##  ##  ##  ##  ##  ##  ##  ##  ##  ##  ##  ##  ##  ##  ##  ##  4  11  7  8  10  4  4  6  6  3  11  8  8  6  6  8  6  5  6  4  6  3  3  6  6  5  5  2  5  3  6  4  7  4  6  4  9  7  5  3  6  --  5  8  9  8  9  9  9  11 [2389_16]263	: ##  ##  ##  ##  ##  ##  ##  ##  ##  ##  ##  ##  ##  ##  ##  ##  ##  ##  ##  ##  ##  ##  ##  ##  ##  ##  ##  ##  ##  ##  ##  ##  ##  ##  ##  ##  ##  ##  ##  ##  ##  ##  ##  ##  ##  ##  ##  ##  ##  ##  ##  ##  ##  ##  ##  ##  ##  ##  ##  ##  ##  ##  ##  ##  ##  ##  ##  ##  ##  ##  ##  ##  ##  ##  ##  ##  ##  ##  ##  ##  ##  ##  ##  ##  ##  ##  ##  ##  ##  ##  ##  ##  ##  ##  ##  ##  ##  ##  ##  ##  ##  ##  ##  ##  ##  ##  ##  ##  ##  ##  ##  ##  ##  ##  ##  ##  ##  ##  ##  ##  ##  ##  ##  ##  ##  ##  ##  ##  ##  ##  ##  ##  ##  ##  ##  ##  ##  ##  ##  ##  ##  ##  ##  ##  ##  ##  ##  ##  ##  ##  ##  ##  ##  ##  ##  ##  ##  ##  ##  ##  ##  ##  ##  ##  ##  ##  ##  ##  ##  ##  ##  ##  ##  ##  ##  ##  ##  ##  ##  ##  ##  ##  ##  ##  ##  ##  ##  ##  ##  ##  ##  ##  ##  ##  ##  ##  ##  ##  ##  ##  ##  ##  ##  ##  ##  ##  ##  ##  ##  ##  ##  ##  ##  ##  ##  ##  ##  ##  ##  ##  3  8  4  5  7  4  3  3  5  2  8  5  7  5  5  5  5  4  3  3  5  4  4  5  5  5  2  3  2  4  5  3  6  3  5  5  9  6  6  4  5  5  --  5  6  3  6  6  6  8 [2466_16]264	: ##  ##  ##  ##  ##  ##  ##  ##  ##  ##  ##  ##  ##  ##  ##  ##  ##  ##  ##  ##  ##  ##  ##  ##  ##  ##  ##  ##  ##  ##  ##  ##  ##  ##  ##  ##  ##  ##  ##  ##  ##  ##  ##  ##  ##  ##  ##  ##  ##  ##  ##  ##  ##  ##  ##  ##  ##  ##  ##  ##  ##  ##  ##  ##  ##  ##  ##  ##  ##  ##  ##  ##  ##  ##  ##  ##  ##  ##  ##  ##  ##  ##  ##  ##  ##  ##  ##  ##  ##  ##  ##  ##  ##  ##  ##  ##  ##  ##  ##  ##  ##  ##  ##  ##  ##  ##  ##  ##  ##  ##  ##  ##  ##  ##  ##  ##  ##  ##  ##  ##  ##  ##  ##  ##  ##  ##  ##  ##  ##  ##  ##  ##  ##  ##  ##  ##  ##  ##  ##  ##  ##  ##  ##  ##  ##  ##  ##  ##  ##  ##  ##  ##  ##  ##  ##  ##  ##  ##  ##  ##  ##  ##  ##  ##  ##  ##  ##  ##  ##  ##  ##  ##  ##  ##  ##  ##  ##  ##  ##  ##  ##  ##  ##  ##  ##  ##  ##  ##  ##  ##  ##  ##  ##  ##  ##  ##  ##  ##  ##  ##  ##  ##  ##  ##  ##  ##  ##  ##  ##  ##  ##  ##  ##  ##  ##  ##  ##  ##  ##  ##  6  7  1  4  8  6  6  6  8  5  5  4  10  8  8  5  6  7  4  6  8  7  7  8  8  8  5  6  5  7  8  6  9  6  8  8  11  9  9  7  8  8  5  --  5  6  5  1  5  8 [2467_16]265	: ##  ##  ##  ##  ##  ##  ##  ##  ##  ##  ##  ##  ##  ##  ##  ##  ##  ##  ##  ##  ##  ##  ##  ##  ##  ##  ##  ##  ##  ##  ##  ##  ##  ##  ##  ##  ##  ##  ##  ##  ##  ##  ##  ##  ##  ##  ##  ##  ##  ##  ##  ##  ##  ##  ##  ##  ##  ##  ##  ##  ##  ##  ##  ##  ##  ##  ##  ##  ##  ##  ##  ##  ##  ##  ##  ##  ##  ##  ##  ##  ##  ##  ##  ##  ##  ##  ##  ##  ##  ##  ##  ##  ##  ##  ##  ##  ##  ##  ##  ##  ##  ##  ##  ##  ##  ##  ##  ##  ##  ##  ##  ##  ##  ##  ##  ##  ##  ##  ##  ##  ##  ##  ##  ##  ##  ##  ##  ##  ##  ##  ##  ##  ##  ##  ##  ##  ##  ##  ##  ##  ##  ##  ##  ##  ##  ##  ##  ##  ##  ##  ##  ##  ##  ##  ##  ##  ##  ##  ##  ##  ##  ##  ##  ##  ##  ##  ##  ##  ##  ##  ##  ##  ##  ##  ##  ##  ##  ##  ##  ##  ##  ##  ##  ##  ##  ##  ##  ##  ##  ##  ##  ##  ##  ##  ##  ##  ##  ##  ##  ##  ##  ##  ##  ##  ##  ##  ##  ##  ##  ##  ##  ##  ##  ##  ##  ##  ##  ##  ##  ##  7  6  4  3  9  6  5  5  9  6  6  3  9  8  9  4  5  8  5  7  9  8  8  7  9  9  6  7  6  8  9  7  8  7  5  9  13  10  8  8  9  9  6  5  --  5  2  6  4  5 [2468_16]266	: ##  ##  ##  ##  ##  ##  ##  ##  ##  ##  ##  ##  ##  ##  ##  ##  ##  ##  ##  ##  ##  ##  ##  ##  ##  ##  ##  ##  ##  ##  ##  ##  ##  ##  ##  ##  ##  ##  ##  ##  ##  ##  ##  ##  ##  ##  ##  ##  ##  ##  ##  ##  ##  ##  ##  ##  ##  ##  ##  ##  ##  ##  ##  ##  ##  ##  ##  ##  ##  ##  ##  ##  ##  ##  ##  ##  ##  ##  ##  ##  ##  ##  ##  ##  ##  ##  ##  ##  ##  ##  ##  ##  ##  ##  ##  ##  ##  ##  ##  ##  ##  ##  ##  ##  ##  ##  ##  ##  ##  ##  ##  ##  ##  ##  ##  ##  ##  ##  ##  ##  ##  ##  ##  ##  ##  ##  ##  ##  ##  ##  ##  ##  ##  ##  ##  ##  ##  ##  ##  ##  ##  ##  ##  ##  ##  ##  ##  ##  ##  ##  ##  ##  ##  ##  ##  ##  ##  ##  ##  ##  ##  ##  ##  ##  ##  ##  ##  ##  ##  ##  ##  ##  ##  ##  ##  ##  ##  ##  ##  ##  ##  ##  ##  ##  ##  ##  ##  ##  ##  ##  ##  ##  ##  ##  ##  ##  ##  ##  ##  ##  ##  ##  ##  ##  ##  ##  ##  ##  ##  ##  ##  ##  ##  ##  ##  ##  ##  ##  ##  ##  6  7  5  4  8  5  4  4  8  5  9  4  10  7  8  6  4  7  4  6  8  7  7  6  8  8  5  6  5  7  8  6  9  6  6  8  12  9  7  7  8  8  3  6  5  --  5  7  5  7 [2469_16]267	: ##  ##  ##  ##  ##  ##  ##  ##  ##  ##  ##  ##  ##  ##  ##  ##  ##  ##  ##  ##  ##  ##  ##  ##  ##  ##  ##  ##  ##  ##  ##  ##  ##  ##  ##  ##  ##  ##  ##  ##  ##  ##  ##  ##  ##  ##  ##  ##  ##  ##  ##  ##  ##  ##  ##  ##  ##  ##  ##  ##  ##  ##  ##  ##  ##  ##  ##  ##  ##  ##  ##  ##  ##  ##  ##  ##  ##  ##  ##  ##  ##  ##  ##  ##  ##  ##  ##  ##  ##  ##  ##  ##  ##  ##  ##  ##  ##  ##  ##  ##  ##  ##  ##  ##  ##  ##  ##  ##  ##  ##  ##  ##  ##  ##  ##  ##  ##  ##  ##  ##  ##  ##  ##  ##  ##  ##  ##  ##  ##  ##  ##  ##  ##  ##  ##  ##  ##  ##  ##  ##  ##  ##  ##  ##  ##  ##  ##  ##  ##  ##  ##  ##  ##  ##  ##  ##  ##  ##  ##  ##  ##  ##  ##  ##  ##  ##  ##  ##  ##  ##  ##  ##  ##  ##  ##  ##  ##  ##  ##  ##  ##  ##  ##  ##  ##  ##  ##  ##  ##  ##  ##  ##  ##  ##  ##  ##  ##  ##  ##  ##  ##  ##  ##  ##  ##  ##  ##  ##  ##  ##  ##  ##  ##  ##  ##  ##  ##  ##  ##  ##  7  6  4  3  9  5  5  5  9  6  8  3  11  8  9  6  5  8  5  7  9  8  8  7  9  9  6  7  6  8  9  7  8  7  7  9  12  10  8  8  9  9  6  5  2  5  --  6  2  5 [2470_16]268	: ##  ##  ##  ##  ##  ##  ##  ##  ##  ##  ##  ##  ##  ##  ##  ##  ##  ##  ##  ##  ##  ##  ##  ##  ##  ##  ##  ##  ##  ##  ##  ##  ##  ##  ##  ##  ##  ##  ##  ##  ##  ##  ##  ##  ##  ##  ##  ##  ##  ##  ##  ##  ##  ##  ##  ##  ##  ##  ##  ##  ##  ##  ##  ##  ##  ##  ##  ##  ##  ##  ##  ##  ##  ##  ##  ##  ##  ##  ##  ##  ##  ##  ##  ##  ##  ##  ##  ##  ##  ##  ##  ##  ##  ##  ##  ##  ##  ##  ##  ##  ##  ##  ##  ##  ##  ##  ##  ##  ##  ##  ##  ##  ##  ##  ##  ##  ##  ##  ##  ##  ##  ##  ##  ##  ##  ##  ##  ##  ##  ##  ##  ##  ##  ##  ##  ##  ##  ##  ##  ##  ##  ##  ##  ##  ##  ##  ##  ##  ##  ##  ##  ##  ##  ##  ##  ##  ##  ##  ##  ##  ##  ##  ##  ##  ##  ##  ##  ##  ##  ##  ##  ##  ##  ##  ##  ##  ##  ##  ##  ##  ##  ##  ##  ##  ##  ##  ##  ##  ##  ##  ##  ##  ##  ##  ##  ##  ##  ##  ##  ##  ##  ##  ##  ##  ##  ##  ##  ##  ##  ##  ##  ##  ##  ##  ##  ##  ##  ##  ##  ##  7  8  2  5  9  8  7  7  9  6  6  5  11  9  9  6  7  8  5  7  7  8  8  9  9  8  6  7  6  8  9  7  10  7  9  7  13  8  10  8  9  9  6  1  6  7  6  --  6  7 [2471_16]269	: ##  ##  ##  ##  ##  ##  ##  ##  ##  ##  ##  ##  ##  ##  ##  ##  ##  ##  ##  ##  ##  ##  ##  ##  ##  ##  ##  ##  ##  ##  ##  ##  ##  ##  ##  ##  ##  ##  ##  ##  ##  ##  ##  ##  ##  ##  ##  ##  ##  ##  ##  ##  ##  ##  ##  ##  ##  ##  ##  ##  ##  ##  ##  ##  ##  ##  ##  ##  ##  ##  ##  ##  ##  ##  ##  ##  ##  ##  ##  ##  ##  ##  ##  ##  ##  ##  ##  ##  ##  ##  ##  ##  ##  ##  ##  ##  ##  ##  ##  ##  ##  ##  ##  ##  ##  ##  ##  ##  ##  ##  ##  ##  ##  ##  ##  ##  ##  ##  ##  ##  ##  ##  ##  ##  ##  ##  ##  ##  ##  ##  ##  ##  ##  ##  ##  ##  ##  ##  ##  ##  ##  ##  ##  ##  ##  ##  ##  ##  ##  ##  ##  ##  ##  ##  ##  ##  ##  ##  ##  ##  ##  ##  ##  ##  ##  ##  ##  ##  ##  ##  ##  ##  ##  ##  ##  ##  ##  ##  ##  ##  ##  ##  ##  ##  ##  ##  ##  ##  ##  ##  ##  ##  ##  ##  ##  ##  ##  ##  ##  ##  ##  ##  ##  ##  ##  ##  ##  ##  ##  ##  ##  ##  ##  ##  ##  ##  ##  ##  ##  ##  7  6  4  3  9  6  5  5  9  6  8  3  9  8  9  6  5  8  5  7  9  8  8  7  9  9  6  7  6  8  9  7  10  7  7  9  13  10  8  8  9  9  6  5  4  5  2  6  --  7 [2474_16]270	: ##  ##  ##  ##  ##  ##  ##  ##  ##  ##  ##  ##  ##  ##  ##  ##  ##  ##  ##  ##  ##  ##  ##  ##  ##  ##  ##  ##  ##  ##  ##  ##  ##  ##  ##  ##  ##  ##  ##  ##  ##  ##  ##  ##  ##  ##  ##  ##  ##  ##  ##  ##  ##  ##  ##  ##  ##  ##  ##  ##  ##  ##  ##  ##  ##  ##  ##  ##  ##  ##  ##  ##  ##  ##  ##  ##  ##  ##  ##  ##  ##  ##  ##  ##  ##  ##  ##  ##  ##  ##  ##  ##  ##  ##  ##  ##  ##  ##  ##  ##  ##  ##  ##  ##  ##  ##  ##  ##  ##  ##  ##  ##  ##  ##  ##  ##  ##  ##  ##  ##  ##  ##  ##  ##  ##  ##  ##  ##  ##  ##  ##  ##  ##  ##  ##  ##  ##  ##  ##  ##  ##  ##  ##  ##  ##  ##  ##  ##  ##  ##  ##  ##  ##  ##  ##  ##  ##  ##  ##  ##  ##  ##  ##  ##  ##  ##  ##  ##  ##  ##  ##  ##  ##  ##  ##  ##  ##  ##  ##  ##  ##  ##  ##  ##  ##  ##  ##  ##  ##  ##  ##  ##  ##  ##  ##  ##  ##  ##  ##  ##  ##  ##  ##  ##  ##  ##  ##  ##  ##  ##  ##  ##  ##  ##  ##  ##  ##  ##  ##  ##  9  7  7  6  10  7  7  7  11  8  11  6  13  10  11  9  7  10  7  7  7  10  10  8  11  8  8  9  6  10  11  9  10  9  9  7  14  8  10  10  11  11  8  8  5  7  5  7  7  -- COMPUTED FROM THE NETWORK DISTANCE MATRIXpos:   		  1  2  3  4  5  6  7  8  9  10  11  12  13  14  15  16  17  18  19  20  21  22  23  24  25  26  27  28  29  30  31  32  33  34  35  36  37  38  39  40  41  42  43  44  45  46  47  48  49  50  51  52  53  54  55  56  57  58  59  60  61  62  63  64  65  66  67  68  69  70  71  72  73  74  75  76  77  78  79  80  81  82  83  84  85  86  87  88  89  90  91  92  93  94  95  96  97  98  99  100  101  102  103  104  105  106  107  108  109  110  111  112  113  114  115  116  117  118  119  120  121  122  123  124  125  126  127  128  129  130  131  132  133  134  135  136  137  138  139  140  141  142  143  144  145  146  147  148  149  150  151  152  153  154  155  156  157  158  159  160  161  162  163  164  165  166  167  168  169  170  171  172  173  174  175  176  177  178  179  180  181  182  183  184  185  186  187  188  189  190  191  192  193  194  195  196  197  198  199  200  201  202  203  204  205  206  207  208  209  210  211  212  213  214  215  216  217  218  219  220  221  222  223  224  225  226  227  228  229  230  231  232  233  234  235  236  237  238  239  240  241  242  243  244  245  246  247  248  249  250  251  252  253  254  255  256  257  258  259  260  261  262  263  264  265  266  267  268  269  270 [841_2]1 	: --  6  6  7  4  7  4  6  5  4  5  6  6  7  5  6  8  10  4  7  5  6  7  6  3  ##  ##  ##  ##  ##  ##  ##  ##  ##  ##  ##  ##  ##  ##  ##  ##  ##  ##  ##  ##  ##  ##  ##  ##  ##  ##  ##  ##  ##  ##  ##  ##  ##  ##  ##  ##  ##  ##  ##  ##  ##  ##  ##  ##  ##  ##  ##  ##  ##  ##  ##  ##  ##  ##  ##  ##  ##  ##  ##  ##  ##  ##  ##  ##  ##  ##  ##  ##  ##  ##  ##  ##  ##  ##  ##  ##  ##  ##  ##  ##  ##  ##  ##  ##  ##  ##  ##  ##  ##  ##  ##  ##  ##  ##  ##  ##  ##  ##  ##  ##  ##  ##  ##  ##  ##  ##  ##  ##  ##  ##  ##  ##  ##  ##  ##  ##  ##  ##  ##  ##  ##  ##  ##  ##  ##  ##  ##  ##  ##  ##  ##  ##  ##  ##  ##  ##  ##  ##  ##  ##  ##  ##  ##  ##  ##  ##  ##  ##  ##  ##  ##  ##  ##  ##  ##  ##  ##  ##  ##  ##  ##  ##  ##  ##  ##  ##  ##  ##  ##  ##  ##  ##  ##  ##  ##  ##  ##  ##  ##  ##  ##  ##  ##  ##  ##  ##  ##  ##  ##  ##  ##  ##  ##  ##  ##  ##  ##  ##  ##  ##  ##  ##  ##  ##  ##  ##  ##  ##  ##  ##  ##  ##  ##  ##  ##  ##  ##  ##  ##  ##  ##  ##  ##  ##  ##  ##  ##  ##  ##  ##  ##  ##  ##  ##  ##  ##  ##  ##  ##  ##  ##  ##  ##  ##  ## [844_2]2 	: 6  --  2  3  2  3  2  2  1  6  3  2  2  3  7  2  4  6  2  3  11  2  3  2  6  ##  ##  ##  ##  ##  ##  ##  ##  ##  ##  ##  ##  ##  ##  ##  ##  ##  ##  ##  ##  ##  ##  ##  ##  ##  ##  ##  ##  ##  ##  ##  ##  ##  ##  ##  ##  ##  ##  ##  ##  ##  ##  ##  ##  ##  ##  ##  ##  ##  ##  ##  ##  ##  ##  ##  ##  ##  ##  ##  ##  ##  ##  ##  ##  ##  ##  ##  ##  ##  ##  ##  ##  ##  ##  ##  ##  ##  ##  ##  ##  ##  ##  ##  ##  ##  ##  ##  ##  ##  ##  ##  ##  ##  ##  ##  ##  ##  ##  ##  ##  ##  ##  ##  ##  ##  ##  ##  ##  ##  ##  ##  ##  ##  ##  ##  ##  ##  ##  ##  ##  ##  ##  ##  ##  ##  ##  ##  ##  ##  ##  ##  ##  ##  ##  ##  ##  ##  ##  ##  ##  ##  ##  ##  ##  ##  ##  ##  ##  ##  ##  ##  ##  ##  ##  ##  ##  ##  ##  ##  ##  ##  ##  ##  ##  ##  ##  ##  ##  ##  ##  ##  ##  ##  ##  ##  ##  ##  ##  ##  ##  ##  ##  ##  ##  ##  ##  ##  ##  ##  ##  ##  ##  ##  ##  ##  ##  ##  ##  ##  ##  ##  ##  ##  ##  ##  ##  ##  ##  ##  ##  ##  ##  ##  ##  ##  ##  ##  ##  ##  ##  ##  ##  ##  ##  ##  ##  ##  ##  ##  ##  ##  ##  ##  ##  ##  ##  ##  ##  ##  ##  ##  ##  ##  ##  ## [1311_2]3 	: 6  2  --  3  2  1  2  2  1  6  3  2  2  3  7  2  4  6  2  3  11  2  3  2  6  ##  ##  ##  ##  ##  ##  ##  ##  ##  ##  ##  ##  ##  ##  ##  ##  ##  ##  ##  ##  ##  ##  ##  ##  ##  ##  ##  ##  ##  ##  ##  ##  ##  ##  ##  ##  ##  ##  ##  ##  ##  ##  ##  ##  ##  ##  ##  ##  ##  ##  ##  ##  ##  ##  ##  ##  ##  ##  ##  ##  ##  ##  ##  ##  ##  ##  ##  ##  ##  ##  ##  ##  ##  ##  ##  ##  ##  ##  ##  ##  ##  ##  ##  ##  ##  ##  ##  ##  ##  ##  ##  ##  ##  ##  ##  ##  ##  ##  ##  ##  ##  ##  ##  ##  ##  ##  ##  ##  ##  ##  ##  ##  ##  ##  ##  ##  ##  ##  ##  ##  ##  ##  ##  ##  ##  ##  ##  ##  ##  ##  ##  ##  ##  ##  ##  ##  ##  ##  ##  ##  ##  ##  ##  ##  ##  ##  ##  ##  ##  ##  ##  ##  ##  ##  ##  ##  ##  ##  ##  ##  ##  ##  ##  ##  ##  ##  ##  ##  ##  ##  ##  ##  ##  ##  ##  ##  ##  ##  ##  ##  ##  ##  ##  ##  ##  ##  ##  ##  ##  ##  ##  ##  ##  ##  ##  ##  ##  ##  ##  ##  ##  ##  ##  ##  ##  ##  ##  ##  ##  ##  ##  ##  ##  ##  ##  ##  ##  ##  ##  ##  ##  ##  ##  ##  ##  ##  ##  ##  ##  ##  ##  ##  ##  ##  ##  ##  ##  ##  ##  ##  ##  ##  ##  ##  ## [2180_2]4 	: 7  3  3  --  3  4  3  3  2  7  4  3  3  4  8  3  5  7  3  4  12  2  4  3  7  ##  ##  ##  ##  ##  ##  ##  ##  ##  ##  ##  ##  ##  ##  ##  ##  ##  ##  ##  ##  ##  ##  ##  ##  ##  ##  ##  ##  ##  ##  ##  ##  ##  ##  ##  ##  ##  ##  ##  ##  ##  ##  ##  ##  ##  ##  ##  ##  ##  ##  ##  ##  ##  ##  ##  ##  ##  ##  ##  ##  ##  ##  ##  ##  ##  ##  ##  ##  ##  ##  ##  ##  ##  ##  ##  ##  ##  ##  ##  ##  ##  ##  ##  ##  ##  ##  ##  ##  ##  ##  ##  ##  ##  ##  ##  ##  ##  ##  ##  ##  ##  ##  ##  ##  ##  ##  ##  ##  ##  ##  ##  ##  ##  ##  ##  ##  ##  ##  ##  ##  ##  ##  ##  ##  ##  ##  ##  ##  ##  ##  ##  ##  ##  ##  ##  ##  ##  ##  ##  ##  ##  ##  ##  ##  ##  ##  ##  ##  ##  ##  ##  ##  ##  ##  ##  ##  ##  ##  ##  ##  ##  ##  ##  ##  ##  ##  ##  ##  ##  ##  ##  ##  ##  ##  ##  ##  ##  ##  ##  ##  ##  ##  ##  ##  ##  ##  ##  ##  ##  ##  ##  ##  ##  ##  ##  ##  ##  ##  ##  ##  ##  ##  ##  ##  ##  ##  ##  ##  ##  ##  ##  ##  ##  ##  ##  ##  ##  ##  ##  ##  ##  ##  ##  ##  ##  ##  ##  ##  ##  ##  ##  ##  ##  ##  ##  ##  ##  ##  ##  ##  ##  ##  ##  ##  ## [2181_2]5 	: 4  2  2  3  --  3  2  2  1  6  1  2  2  3  7  2  4  6  2  3  9  2  3  2  6  ##  ##  ##  ##  ##  ##  ##  ##  ##  ##  ##  ##  ##  ##  ##  ##  ##  ##  ##  ##  ##  ##  ##  ##  ##  ##  ##  ##  ##  ##  ##  ##  ##  ##  ##  ##  ##  ##  ##  ##  ##  ##  ##  ##  ##  ##  ##  ##  ##  ##  ##  ##  ##  ##  ##  ##  ##  ##  ##  ##  ##  ##  ##  ##  ##  ##  ##  ##  ##  ##  ##  ##  ##  ##  ##  ##  ##  ##  ##  ##  ##  ##  ##  ##  ##  ##  ##  ##  ##  ##  ##  ##  ##  ##  ##  ##  ##  ##  ##  ##  ##  ##  ##  ##  ##  ##  ##  ##  ##  ##  ##  ##  ##  ##  ##  ##  ##  ##  ##  ##  ##  ##  ##  ##  ##  ##  ##  ##  ##  ##  ##  ##  ##  ##  ##  ##  ##  ##  ##  ##  ##  ##  ##  ##  ##  ##  ##  ##  ##  ##  ##  ##  ##  ##  ##  ##  ##  ##  ##  ##  ##  ##  ##  ##  ##  ##  ##  ##  ##  ##  ##  ##  ##  ##  ##  ##  ##  ##  ##  ##  ##  ##  ##  ##  ##  ##  ##  ##  ##  ##  ##  ##  ##  ##  ##  ##  ##  ##  ##  ##  ##  ##  ##  ##  ##  ##  ##  ##  ##  ##  ##  ##  ##  ##  ##  ##  ##  ##  ##  ##  ##  ##  ##  ##  ##  ##  ##  ##  ##  ##  ##  ##  ##  ##  ##  ##  ##  ##  ##  ##  ##  ##  ##  ##  ## [2182_2]6 	: 7  3  1  4  3  --  3  3  2  7  4  3  3  4  8  3  5  7  3  4  12  3  4  3  7  ##  ##  ##  ##  ##  ##  ##  ##  ##  ##  ##  ##  ##  ##  ##  ##  ##  ##  ##  ##  ##  ##  ##  ##  ##  ##  ##  ##  ##  ##  ##  ##  ##  ##  ##  ##  ##  ##  ##  ##  ##  ##  ##  ##  ##  ##  ##  ##  ##  ##  ##  ##  ##  ##  ##  ##  ##  ##  ##  ##  ##  ##  ##  ##  ##  ##  ##  ##  ##  ##  ##  ##  ##  ##  ##  ##  ##  ##  ##  ##  ##  ##  ##  ##  ##  ##  ##  ##  ##  ##  ##  ##  ##  ##  ##  ##  ##  ##  ##  ##  ##  ##  ##  ##  ##  ##  ##  ##  ##  ##  ##  ##  ##  ##  ##  ##  ##  ##  ##  ##  ##  ##  ##  ##  ##  ##  ##  ##  ##  ##  ##  ##  ##  ##  ##  ##  ##  ##  ##  ##  ##  ##  ##  ##  ##  ##  ##  ##  ##  ##  ##  ##  ##  ##  ##  ##  ##  ##  ##  ##  ##  ##  ##  ##  ##  ##  ##  ##  ##  ##  ##  ##  ##  ##  ##  ##  ##  ##  ##  ##  ##  ##  ##  ##  ##  ##  ##  ##  ##  ##  ##  ##  ##  ##  ##  ##  ##  ##  ##  ##  ##  ##  ##  ##  ##  ##  ##  ##  ##  ##  ##  ##  ##  ##  ##  ##  ##  ##  ##  ##  ##  ##  ##  ##  ##  ##  ##  ##  ##  ##  ##  ##  ##  ##  ##  ##  ##  ##  ##  ##  ##  ##  ##  ##  ## [2185_2]7 	: 4  2  2  3  2  3  --  2  1  4  3  2  2  3  5  2  4  6  2  3  9  2  3  2  4  ##  ##  ##  ##  ##  ##  ##  ##  ##  ##  ##  ##  ##  ##  ##  ##  ##  ##  ##  ##  ##  ##  ##  ##  ##  ##  ##  ##  ##  ##  ##  ##  ##  ##  ##  ##  ##  ##  ##  ##  ##  ##  ##  ##  ##  ##  ##  ##  ##  ##  ##  ##  ##  ##  ##  ##  ##  ##  ##  ##  ##  ##  ##  ##  ##  ##  ##  ##  ##  ##  ##  ##  ##  ##  ##  ##  ##  ##  ##  ##  ##  ##  ##  ##  ##  ##  ##  ##  ##  ##  ##  ##  ##  ##  ##  ##  ##  ##  ##  ##  ##  ##  ##  ##  ##  ##  ##  ##  ##  ##  ##  ##  ##  ##  ##  ##  ##  ##  ##  ##  ##  ##  ##  ##  ##  ##  ##  ##  ##  ##  ##  ##  ##  ##  ##  ##  ##  ##  ##  ##  ##  ##  ##  ##  ##  ##  ##  ##  ##  ##  ##  ##  ##  ##  ##  ##  ##  ##  ##  ##  ##  ##  ##  ##  ##  ##  ##  ##  ##  ##  ##  ##  ##  ##  ##  ##  ##  ##  ##  ##  ##  ##  ##  ##  ##  ##  ##  ##  ##  ##  ##  ##  ##  ##  ##  ##  ##  ##  ##  ##  ##  ##  ##  ##  ##  ##  ##  ##  ##  ##  ##  ##  ##  ##  ##  ##  ##  ##  ##  ##  ##  ##  ##  ##  ##  ##  ##  ##  ##  ##  ##  ##  ##  ##  ##  ##  ##  ##  ##  ##  ##  ##  ##  ##  ## [2187_2]8 	: 6  2  2  3  2  3  2  --  1  6  3  2  2  3  7  2  4  6  2  3  11  2  3  2  6  ##  ##  ##  ##  ##  ##  ##  ##  ##  ##  ##  ##  ##  ##  ##  ##  ##  ##  ##  ##  ##  ##  ##  ##  ##  ##  ##  ##  ##  ##  ##  ##  ##  ##  ##  ##  ##  ##  ##  ##  ##  ##  ##  ##  ##  ##  ##  ##  ##  ##  ##  ##  ##  ##  ##  ##  ##  ##  ##  ##  ##  ##  ##  ##  ##  ##  ##  ##  ##  ##  ##  ##  ##  ##  ##  ##  ##  ##  ##  ##  ##  ##  ##  ##  ##  ##  ##  ##  ##  ##  ##  ##  ##  ##  ##  ##  ##  ##  ##  ##  ##  ##  ##  ##  ##  ##  ##  ##  ##  ##  ##  ##  ##  ##  ##  ##  ##  ##  ##  ##  ##  ##  ##  ##  ##  ##  ##  ##  ##  ##  ##  ##  ##  ##  ##  ##  ##  ##  ##  ##  ##  ##  ##  ##  ##  ##  ##  ##  ##  ##  ##  ##  ##  ##  ##  ##  ##  ##  ##  ##  ##  ##  ##  ##  ##  ##  ##  ##  ##  ##  ##  ##  ##  ##  ##  ##  ##  ##  ##  ##  ##  ##  ##  ##  ##  ##  ##  ##  ##  ##  ##  ##  ##  ##  ##  ##  ##  ##  ##  ##  ##  ##  ##  ##  ##  ##  ##  ##  ##  ##  ##  ##  ##  ##  ##  ##  ##  ##  ##  ##  ##  ##  ##  ##  ##  ##  ##  ##  ##  ##  ##  ##  ##  ##  ##  ##  ##  ##  ##  ##  ##  ##  ##  ##  ## [2216_2]9 	: 5  1  1  2  1  2  1  1  --  5  2  1  1  2  6  1  3  5  1  2  10  1  2  1  5  ##  ##  ##  ##  ##  ##  ##  ##  ##  ##  ##  ##  ##  ##  ##  ##  ##  ##  ##  ##  ##  ##  ##  ##  ##  ##  ##  ##  ##  ##  ##  ##  ##  ##  ##  ##  ##  ##  ##  ##  ##  ##  ##  ##  ##  ##  ##  ##  ##  ##  ##  ##  ##  ##  ##  ##  ##  ##  ##  ##  ##  ##  ##  ##  ##  ##  ##  ##  ##  ##  ##  ##  ##  ##  ##  ##  ##  ##  ##  ##  ##  ##  ##  ##  ##  ##  ##  ##  ##  ##  ##  ##  ##  ##  ##  ##  ##  ##  ##  ##  ##  ##  ##  ##  ##  ##  ##  ##  ##  ##  ##  ##  ##  ##  ##  ##  ##  ##  ##  ##  ##  ##  ##  ##  ##  ##  ##  ##  ##  ##  ##  ##  ##  ##  ##  ##  ##  ##  ##  ##  ##  ##  ##  ##  ##  ##  ##  ##  ##  ##  ##  ##  ##  ##  ##  ##  ##  ##  ##  ##  ##  ##  ##  ##  ##  ##  ##  ##  ##  ##  ##  ##  ##  ##  ##  ##  ##  ##  ##  ##  ##  ##  ##  ##  ##  ##  ##  ##  ##  ##  ##  ##  ##  ##  ##  ##  ##  ##  ##  ##  ##  ##  ##  ##  ##  ##  ##  ##  ##  ##  ##  ##  ##  ##  ##  ##  ##  ##  ##  ##  ##  ##  ##  ##  ##  ##  ##  ##  ##  ##  ##  ##  ##  ##  ##  ##  ##  ##  ##  ##  ##  ##  ##  ##  ## [2271_2]10	: 4  6  6  7  6  7  4  6  5  --  7  6  6  7  1  6  8  10  6  7  9  6  7  6  1  ##  ##  ##  ##  ##  ##  ##  ##  ##  ##  ##  ##  ##  ##  ##  ##  ##  ##  ##  ##  ##  ##  ##  ##  ##  ##  ##  ##  ##  ##  ##  ##  ##  ##  ##  ##  ##  ##  ##  ##  ##  ##  ##  ##  ##  ##  ##  ##  ##  ##  ##  ##  ##  ##  ##  ##  ##  ##  ##  ##  ##  ##  ##  ##  ##  ##  ##  ##  ##  ##  ##  ##  ##  ##  ##  ##  ##  ##  ##  ##  ##  ##  ##  ##  ##  ##  ##  ##  ##  ##  ##  ##  ##  ##  ##  ##  ##  ##  ##  ##  ##  ##  ##  ##  ##  ##  ##  ##  ##  ##  ##  ##  ##  ##  ##  ##  ##  ##  ##  ##  ##  ##  ##  ##  ##  ##  ##  ##  ##  ##  ##  ##  ##  ##  ##  ##  ##  ##  ##  ##  ##  ##  ##  ##  ##  ##  ##  ##  ##  ##  ##  ##  ##  ##  ##  ##  ##  ##  ##  ##  ##  ##  ##  ##  ##  ##  ##  ##  ##  ##  ##  ##  ##  ##  ##  ##  ##  ##  ##  ##  ##  ##  ##  ##  ##  ##  ##  ##  ##  ##  ##  ##  ##  ##  ##  ##  ##  ##  ##  ##  ##  ##  ##  ##  ##  ##  ##  ##  ##  ##  ##  ##  ##  ##  ##  ##  ##  ##  ##  ##  ##  ##  ##  ##  ##  ##  ##  ##  ##  ##  ##  ##  ##  ##  ##  ##  ##  ##  ##  ##  ##  ##  ##  ##  ## [2272_2]11	: 5  3  3  4  1  4  3  3  2  7  --  3  3  4  8  3  5  7  3  4  10  3  4  3  7  ##  ##  ##  ##  ##  ##  ##  ##  ##  ##  ##  ##  ##  ##  ##  ##  ##  ##  ##  ##  ##  ##  ##  ##  ##  ##  ##  ##  ##  ##  ##  ##  ##  ##  ##  ##  ##  ##  ##  ##  ##  ##  ##  ##  ##  ##  ##  ##  ##  ##  ##  ##  ##  ##  ##  ##  ##  ##  ##  ##  ##  ##  ##  ##  ##  ##  ##  ##  ##  ##  ##  ##  ##  ##  ##  ##  ##  ##  ##  ##  ##  ##  ##  ##  ##  ##  ##  ##  ##  ##  ##  ##  ##  ##  ##  ##  ##  ##  ##  ##  ##  ##  ##  ##  ##  ##  ##  ##  ##  ##  ##  ##  ##  ##  ##  ##  ##  ##  ##  ##  ##  ##  ##  ##  ##  ##  ##  ##  ##  ##  ##  ##  ##  ##  ##  ##  ##  ##  ##  ##  ##  ##  ##  ##  ##  ##  ##  ##  ##  ##  ##  ##  ##  ##  ##  ##  ##  ##  ##  ##  ##  ##  ##  ##  ##  ##  ##  ##  ##  ##  ##  ##  ##  ##  ##  ##  ##  ##  ##  ##  ##  ##  ##  ##  ##  ##  ##  ##  ##  ##  ##  ##  ##  ##  ##  ##  ##  ##  ##  ##  ##  ##  ##  ##  ##  ##  ##  ##  ##  ##  ##  ##  ##  ##  ##  ##  ##  ##  ##  ##  ##  ##  ##  ##  ##  ##  ##  ##  ##  ##  ##  ##  ##  ##  ##  ##  ##  ##  ##  ##  ##  ##  ##  ##  ## [2326_2]12	: 6  2  2  3  2  3  2  2  1  6  3  --  2  3  7  2  4  6  2  3  11  2  3  2  6  ##  ##  ##  ##  ##  ##  ##  ##  ##  ##  ##  ##  ##  ##  ##  ##  ##  ##  ##  ##  ##  ##  ##  ##  ##  ##  ##  ##  ##  ##  ##  ##  ##  ##  ##  ##  ##  ##  ##  ##  ##  ##  ##  ##  ##  ##  ##  ##  ##  ##  ##  ##  ##  ##  ##  ##  ##  ##  ##  ##  ##  ##  ##  ##  ##  ##  ##  ##  ##  ##  ##  ##  ##  ##  ##  ##  ##  ##  ##  ##  ##  ##  ##  ##  ##  ##  ##  ##  ##  ##  ##  ##  ##  ##  ##  ##  ##  ##  ##  ##  ##  ##  ##  ##  ##  ##  ##  ##  ##  ##  ##  ##  ##  ##  ##  ##  ##  ##  ##  ##  ##  ##  ##  ##  ##  ##  ##  ##  ##  ##  ##  ##  ##  ##  ##  ##  ##  ##  ##  ##  ##  ##  ##  ##  ##  ##  ##  ##  ##  ##  ##  ##  ##  ##  ##  ##  ##  ##  ##  ##  ##  ##  ##  ##  ##  ##  ##  ##  ##  ##  ##  ##  ##  ##  ##  ##  ##  ##  ##  ##  ##  ##  ##  ##  ##  ##  ##  ##  ##  ##  ##  ##  ##  ##  ##  ##  ##  ##  ##  ##  ##  ##  ##  ##  ##  ##  ##  ##  ##  ##  ##  ##  ##  ##  ##  ##  ##  ##  ##  ##  ##  ##  ##  ##  ##  ##  ##  ##  ##  ##  ##  ##  ##  ##  ##  ##  ##  ##  ##  ##  ##  ##  ##  ##  ## [2328_2]13	: 6  2  2  3  2  3  2  2  1  6  3  2  --  3  7  2  4  6  2  3  11  2  3  2  6  ##  ##  ##  ##  ##  ##  ##  ##  ##  ##  ##  ##  ##  ##  ##  ##  ##  ##  ##  ##  ##  ##  ##  ##  ##  ##  ##  ##  ##  ##  ##  ##  ##  ##  ##  ##  ##  ##  ##  ##  ##  ##  ##  ##  ##  ##  ##  ##  ##  ##  ##  ##  ##  ##  ##  ##  ##  ##  ##  ##  ##  ##  ##  ##  ##  ##  ##  ##  ##  ##  ##  ##  ##  ##  ##  ##  ##  ##  ##  ##  ##  ##  ##  ##  ##  ##  ##  ##  ##  ##  ##  ##  ##  ##  ##  ##  ##  ##  ##  ##  ##  ##  ##  ##  ##  ##  ##  ##  ##  ##  ##  ##  ##  ##  ##  ##  ##  ##  ##  ##  ##  ##  ##  ##  ##  ##  ##  ##  ##  ##  ##  ##  ##  ##  ##  ##  ##  ##  ##  ##  ##  ##  ##  ##  ##  ##  ##  ##  ##  ##  ##  ##  ##  ##  ##  ##  ##  ##  ##  ##  ##  ##  ##  ##  ##  ##  ##  ##  ##  ##  ##  ##  ##  ##  ##  ##  ##  ##  ##  ##  ##  ##  ##  ##  ##  ##  ##  ##  ##  ##  ##  ##  ##  ##  ##  ##  ##  ##  ##  ##  ##  ##  ##  ##  ##  ##  ##  ##  ##  ##  ##  ##  ##  ##  ##  ##  ##  ##  ##  ##  ##  ##  ##  ##  ##  ##  ##  ##  ##  ##  ##  ##  ##  ##  ##  ##  ##  ##  ##  ##  ##  ##  ##  ##  ## [2331_2]14	: 7  3  3  4  3  4  3  3  2  7  4  3  3  --  8  3  5  7  3  4  12  3  4  3  7  ##  ##  ##  ##  ##  ##  ##  ##  ##  ##  ##  ##  ##  ##  ##  ##  ##  ##  ##  ##  ##  ##  ##  ##  ##  ##  ##  ##  ##  ##  ##  ##  ##  ##  ##  ##  ##  ##  ##  ##  ##  ##  ##  ##  ##  ##  ##  ##  ##  ##  ##  ##  ##  ##  ##  ##  ##  ##  ##  ##  ##  ##  ##  ##  ##  ##  ##  ##  ##  ##  ##  ##  ##  ##  ##  ##  ##  ##  ##  ##  ##  ##  ##  ##  ##  ##  ##  ##  ##  ##  ##  ##  ##  ##  ##  ##  ##  ##  ##  ##  ##  ##  ##  ##  ##  ##  ##  ##  ##  ##  ##  ##  ##  ##  ##  ##  ##  ##  ##  ##  ##  ##  ##  ##  ##  ##  ##  ##  ##  ##  ##  ##  ##  ##  ##  ##  ##  ##  ##  ##  ##  ##  ##  ##  ##  ##  ##  ##  ##  ##  ##  ##  ##  ##  ##  ##  ##  ##  ##  ##  ##  ##  ##  ##  ##  ##  ##  ##  ##  ##  ##  ##  ##  ##  ##  ##  ##  ##  ##  ##  ##  ##  ##  ##  ##  ##  ##  ##  ##  ##  ##  ##  ##  ##  ##  ##  ##  ##  ##  ##  ##  ##  ##  ##  ##  ##  ##  ##  ##  ##  ##  ##  ##  ##  ##  ##  ##  ##  ##  ##  ##  ##  ##  ##  ##  ##  ##  ##  ##  ##  ##  ##  ##  ##  ##  ##  ##  ##  ##  ##  ##  ##  ##  ##  ## [2332_2]15	: 5  7  7  8  7  8  5  7  6  1  8  7  7  8  --  7  9  11  7  8  10  7  8  7  2  ##  ##  ##  ##  ##  ##  ##  ##  ##  ##  ##  ##  ##  ##  ##  ##  ##  ##  ##  ##  ##  ##  ##  ##  ##  ##  ##  ##  ##  ##  ##  ##  ##  ##  ##  ##  ##  ##  ##  ##  ##  ##  ##  ##  ##  ##  ##  ##  ##  ##  ##  ##  ##  ##  ##  ##  ##  ##  ##  ##  ##  ##  ##  ##  ##  ##  ##  ##  ##  ##  ##  ##  ##  ##  ##  ##  ##  ##  ##  ##  ##  ##  ##  ##  ##  ##  ##  ##  ##  ##  ##  ##  ##  ##  ##  ##  ##  ##  ##  ##  ##  ##  ##  ##  ##  ##  ##  ##  ##  ##  ##  ##  ##  ##  ##  ##  ##  ##  ##  ##  ##  ##  ##  ##  ##  ##  ##  ##  ##  ##  ##  ##  ##  ##  ##  ##  ##  ##  ##  ##  ##  ##  ##  ##  ##  ##  ##  ##  ##  ##  ##  ##  ##  ##  ##  ##  ##  ##  ##  ##  ##  ##  ##  ##  ##  ##  ##  ##  ##  ##  ##  ##  ##  ##  ##  ##  ##  ##  ##  ##  ##  ##  ##  ##  ##  ##  ##  ##  ##  ##  ##  ##  ##  ##  ##  ##  ##  ##  ##  ##  ##  ##  ##  ##  ##  ##  ##  ##  ##  ##  ##  ##  ##  ##  ##  ##  ##  ##  ##  ##  ##  ##  ##  ##  ##  ##  ##  ##  ##  ##  ##  ##  ##  ##  ##  ##  ##  ##  ##  ##  ##  ##  ##  ##  ## [2333_2]16	: 6  2  2  3  2  3  2  2  1  6  3  2  2  3  7  --  4  6  2  3  11  2  3  2  6  ##  ##  ##  ##  ##  ##  ##  ##  ##  ##  ##  ##  ##  ##  ##  ##  ##  ##  ##  ##  ##  ##  ##  ##  ##  ##  ##  ##  ##  ##  ##  ##  ##  ##  ##  ##  ##  ##  ##  ##  ##  ##  ##  ##  ##  ##  ##  ##  ##  ##  ##  ##  ##  ##  ##  ##  ##  ##  ##  ##  ##  ##  ##  ##  ##  ##  ##  ##  ##  ##  ##  ##  ##  ##  ##  ##  ##  ##  ##  ##  ##  ##  ##  ##  ##  ##  ##  ##  ##  ##  ##  ##  ##  ##  ##  ##  ##  ##  ##  ##  ##  ##  ##  ##  ##  ##  ##  ##  ##  ##  ##  ##  ##  ##  ##  ##  ##  ##  ##  ##  ##  ##  ##  ##  ##  ##  ##  ##  ##  ##  ##  ##  ##  ##  ##  ##  ##  ##  ##  ##  ##  ##  ##  ##  ##  ##  ##  ##  ##  ##  ##  ##  ##  ##  ##  ##  ##  ##  ##  ##  ##  ##  ##  ##  ##  ##  ##  ##  ##  ##  ##  ##  ##  ##  ##  ##  ##  ##  ##  ##  ##  ##  ##  ##  ##  ##  ##  ##  ##  ##  ##  ##  ##  ##  ##  ##  ##  ##  ##  ##  ##  ##  ##  ##  ##  ##  ##  ##  ##  ##  ##  ##  ##  ##  ##  ##  ##  ##  ##  ##  ##  ##  ##  ##  ##  ##  ##  ##  ##  ##  ##  ##  ##  ##  ##  ##  ##  ##  ##  ##  ##  ##  ##  ##  ## [2338_2]17	: 8  4  4  5  4  5  4  4  3  8  5  4  4  5  9  4  --  4  4  1  13  4  5  4  8  ##  ##  ##  ##  ##  ##  ##  ##  ##  ##  ##  ##  ##  ##  ##  ##  ##  ##  ##  ##  ##  ##  ##  ##  ##  ##  ##  ##  ##  ##  ##  ##  ##  ##  ##  ##  ##  ##  ##  ##  ##  ##  ##  ##  ##  ##  ##  ##  ##  ##  ##  ##  ##  ##  ##  ##  ##  ##  ##  ##  ##  ##  ##  ##  ##  ##  ##  ##  ##  ##  ##  ##  ##  ##  ##  ##  ##  ##  ##  ##  ##  ##  ##  ##  ##  ##  ##  ##  ##  ##  ##  ##  ##  ##  ##  ##  ##  ##  ##  ##  ##  ##  ##  ##  ##  ##  ##  ##  ##  ##  ##  ##  ##  ##  ##  ##  ##  ##  ##  ##  ##  ##  ##  ##  ##  ##  ##  ##  ##  ##  ##  ##  ##  ##  ##  ##  ##  ##  ##  ##  ##  ##  ##  ##  ##  ##  ##  ##  ##  ##  ##  ##  ##  ##  ##  ##  ##  ##  ##  ##  ##  ##  ##  ##  ##  ##  ##  ##  ##  ##  ##  ##  ##  ##  ##  ##  ##  ##  ##  ##  ##  ##  ##  ##  ##  ##  ##  ##  ##  ##  ##  ##  ##  ##  ##  ##  ##  ##  ##  ##  ##  ##  ##  ##  ##  ##  ##  ##  ##  ##  ##  ##  ##  ##  ##  ##  ##  ##  ##  ##  ##  ##  ##  ##  ##  ##  ##  ##  ##  ##  ##  ##  ##  ##  ##  ##  ##  ##  ##  ##  ##  ##  ##  ##  ## [2352_2]18	: 10  6  6  7  6  7  6  6  5  10  7  6  6  7  11  6  4  --  6  3  15  6  7  6  10  ##  ##  ##  ##  ##  ##  ##  ##  ##  ##  ##  ##  ##  ##  ##  ##  ##  ##  ##  ##  ##  ##  ##  ##  ##  ##  ##  ##  ##  ##  ##  ##  ##  ##  ##  ##  ##  ##  ##  ##  ##  ##  ##  ##  ##  ##  ##  ##  ##  ##  ##  ##  ##  ##  ##  ##  ##  ##  ##  ##  ##  ##  ##  ##  ##  ##  ##  ##  ##  ##  ##  ##  ##  ##  ##  ##  ##  ##  ##  ##  ##  ##  ##  ##  ##  ##  ##  ##  ##  ##  ##  ##  ##  ##  ##  ##  ##  ##  ##  ##  ##  ##  ##  ##  ##  ##  ##  ##  ##  ##  ##  ##  ##  ##  ##  ##  ##  ##  ##  ##  ##  ##  ##  ##  ##  ##  ##  ##  ##  ##  ##  ##  ##  ##  ##  ##  ##  ##  ##  ##  ##  ##  ##  ##  ##  ##  ##  ##  ##  ##  ##  ##  ##  ##  ##  ##  ##  ##  ##  ##  ##  ##  ##  ##  ##  ##  ##  ##  ##  ##  ##  ##  ##  ##  ##  ##  ##  ##  ##  ##  ##  ##  ##  ##  ##  ##  ##  ##  ##  ##  ##  ##  ##  ##  ##  ##  ##  ##  ##  ##  ##  ##  ##  ##  ##  ##  ##  ##  ##  ##  ##  ##  ##  ##  ##  ##  ##  ##  ##  ##  ##  ##  ##  ##  ##  ##  ##  ##  ##  ##  ##  ##  ##  ##  ##  ##  ##  ##  ##  ##  ##  ##  ##  ##  ## [2354_2]19	: 4  2  2  3  2  3  2  2  1  6  3  2  2  3  7  2  4  6  --  3  9  2  3  2  5  ##  ##  ##  ##  ##  ##  ##  ##  ##  ##  ##  ##  ##  ##  ##  ##  ##  ##  ##  ##  ##  ##  ##  ##  ##  ##  ##  ##  ##  ##  ##  ##  ##  ##  ##  ##  ##  ##  ##  ##  ##  ##  ##  ##  ##  ##  ##  ##  ##  ##  ##  ##  ##  ##  ##  ##  ##  ##  ##  ##  ##  ##  ##  ##  ##  ##  ##  ##  ##  ##  ##  ##  ##  ##  ##  ##  ##  ##  ##  ##  ##  ##  ##  ##  ##  ##  ##  ##  ##  ##  ##  ##  ##  ##  ##  ##  ##  ##  ##  ##  ##  ##  ##  ##  ##  ##  ##  ##  ##  ##  ##  ##  ##  ##  ##  ##  ##  ##  ##  ##  ##  ##  ##  ##  ##  ##  ##  ##  ##  ##  ##  ##  ##  ##  ##  ##  ##  ##  ##  ##  ##  ##  ##  ##  ##  ##  ##  ##  ##  ##  ##  ##  ##  ##  ##  ##  ##  ##  ##  ##  ##  ##  ##  ##  ##  ##  ##  ##  ##  ##  ##  ##  ##  ##  ##  ##  ##  ##  ##  ##  ##  ##  ##  ##  ##  ##  ##  ##  ##  ##  ##  ##  ##  ##  ##  ##  ##  ##  ##  ##  ##  ##  ##  ##  ##  ##  ##  ##  ##  ##  ##  ##  ##  ##  ##  ##  ##  ##  ##  ##  ##  ##  ##  ##  ##  ##  ##  ##  ##  ##  ##  ##  ##  ##  ##  ##  ##  ##  ##  ##  ##  ##  ##  ##  ## [2367_2]20	: 7  3  3  4  3  4  3  3  2  7  4  3  3  4  8  3  1  3  3  --  12  3  4  3  7  ##  ##  ##  ##  ##  ##  ##  ##  ##  ##  ##  ##  ##  ##  ##  ##  ##  ##  ##  ##  ##  ##  ##  ##  ##  ##  ##  ##  ##  ##  ##  ##  ##  ##  ##  ##  ##  ##  ##  ##  ##  ##  ##  ##  ##  ##  ##  ##  ##  ##  ##  ##  ##  ##  ##  ##  ##  ##  ##  ##  ##  ##  ##  ##  ##  ##  ##  ##  ##  ##  ##  ##  ##  ##  ##  ##  ##  ##  ##  ##  ##  ##  ##  ##  ##  ##  ##  ##  ##  ##  ##  ##  ##  ##  ##  ##  ##  ##  ##  ##  ##  ##  ##  ##  ##  ##  ##  ##  ##  ##  ##  ##  ##  ##  ##  ##  ##  ##  ##  ##  ##  ##  ##  ##  ##  ##  ##  ##  ##  ##  ##  ##  ##  ##  ##  ##  ##  ##  ##  ##  ##  ##  ##  ##  ##  ##  ##  ##  ##  ##  ##  ##  ##  ##  ##  ##  ##  ##  ##  ##  ##  ##  ##  ##  ##  ##  ##  ##  ##  ##  ##  ##  ##  ##  ##  ##  ##  ##  ##  ##  ##  ##  ##  ##  ##  ##  ##  ##  ##  ##  ##  ##  ##  ##  ##  ##  ##  ##  ##  ##  ##  ##  ##  ##  ##  ##  ##  ##  ##  ##  ##  ##  ##  ##  ##  ##  ##  ##  ##  ##  ##  ##  ##  ##  ##  ##  ##  ##  ##  ##  ##  ##  ##  ##  ##  ##  ##  ##  ##  ##  ##  ##  ##  ##  ## [2370_2]21	: 5  11  11  12  9  12  9  11  10  9  10  11  11  12  10  11  13  15  9  12  --  11  12  11  8  ##  ##  ##  ##  ##  ##  ##  ##  ##  ##  ##  ##  ##  ##  ##  ##  ##  ##  ##  ##  ##  ##  ##  ##  ##  ##  ##  ##  ##  ##  ##  ##  ##  ##  ##  ##  ##  ##  ##  ##  ##  ##  ##  ##  ##  ##  ##  ##  ##  ##  ##  ##  ##  ##  ##  ##  ##  ##  ##  ##  ##  ##  ##  ##  ##  ##  ##  ##  ##  ##  ##  ##  ##  ##  ##  ##  ##  ##  ##  ##  ##  ##  ##  ##  ##  ##  ##  ##  ##  ##  ##  ##  ##  ##  ##  ##  ##  ##  ##  ##  ##  ##  ##  ##  ##  ##  ##  ##  ##  ##  ##  ##  ##  ##  ##  ##  ##  ##  ##  ##  ##  ##  ##  ##  ##  ##  ##  ##  ##  ##  ##  ##  ##  ##  ##  ##  ##  ##  ##  ##  ##  ##  ##  ##  ##  ##  ##  ##  ##  ##  ##  ##  ##  ##  ##  ##  ##  ##  ##  ##  ##  ##  ##  ##  ##  ##  ##  ##  ##  ##  ##  ##  ##  ##  ##  ##  ##  ##  ##  ##  ##  ##  ##  ##  ##  ##  ##  ##  ##  ##  ##  ##  ##  ##  ##  ##  ##  ##  ##  ##  ##  ##  ##  ##  ##  ##  ##  ##  ##  ##  ##  ##  ##  ##  ##  ##  ##  ##  ##  ##  ##  ##  ##  ##  ##  ##  ##  ##  ##  ##  ##  ##  ##  ##  ##  ##  ##  ##  ##  ##  ##  ##  ##  ##  ## [2378_2]22	: 6  2  2  2  2  3  2  2  1  6  3  2  2  3  7  2  4  6  2  3  11  --  3  2  6  ##  ##  ##  ##  ##  ##  ##  ##  ##  ##  ##  ##  ##  ##  ##  ##  ##  ##  ##  ##  ##  ##  ##  ##  ##  ##  ##  ##  ##  ##  ##  ##  ##  ##  ##  ##  ##  ##  ##  ##  ##  ##  ##  ##  ##  ##  ##  ##  ##  ##  ##  ##  ##  ##  ##  ##  ##  ##  ##  ##  ##  ##  ##  ##  ##  ##  ##  ##  ##  ##  ##  ##  ##  ##  ##  ##  ##  ##  ##  ##  ##  ##  ##  ##  ##  ##  ##  ##  ##  ##  ##  ##  ##  ##  ##  ##  ##  ##  ##  ##  ##  ##  ##  ##  ##  ##  ##  ##  ##  ##  ##  ##  ##  ##  ##  ##  ##  ##  ##  ##  ##  ##  ##  ##  ##  ##  ##  ##  ##  ##  ##  ##  ##  ##  ##  ##  ##  ##  ##  ##  ##  ##  ##  ##  ##  ##  ##  ##  ##  ##  ##  ##  ##  ##  ##  ##  ##  ##  ##  ##  ##  ##  ##  ##  ##  ##  ##  ##  ##  ##  ##  ##  ##  ##  ##  ##  ##  ##  ##  ##  ##  ##  ##  ##  ##  ##  ##  ##  ##  ##  ##  ##  ##  ##  ##  ##  ##  ##  ##  ##  ##  ##  ##  ##  ##  ##  ##  ##  ##  ##  ##  ##  ##  ##  ##  ##  ##  ##  ##  ##  ##  ##  ##  ##  ##  ##  ##  ##  ##  ##  ##  ##  ##  ##  ##  ##  ##  ##  ##  ##  ##  ##  ##  ##  ## [2381_2]23	: 7  3  3  4  3  4  3  3  2  7  4  3  3  4  8  3  5  7  3  4  12  3  --  3  7  ##  ##  ##  ##  ##  ##  ##  ##  ##  ##  ##  ##  ##  ##  ##  ##  ##  ##  ##  ##  ##  ##  ##  ##  ##  ##  ##  ##  ##  ##  ##  ##  ##  ##  ##  ##  ##  ##  ##  ##  ##  ##  ##  ##  ##  ##  ##  ##  ##  ##  ##  ##  ##  ##  ##  ##  ##  ##  ##  ##  ##  ##  ##  ##  ##  ##  ##  ##  ##  ##  ##  ##  ##  ##  ##  ##  ##  ##  ##  ##  ##  ##  ##  ##  ##  ##  ##  ##  ##  ##  ##  ##  ##  ##  ##  ##  ##  ##  ##  ##  ##  ##  ##  ##  ##  ##  ##  ##  ##  ##  ##  ##  ##  ##  ##  ##  ##  ##  ##  ##  ##  ##  ##  ##  ##  ##  ##  ##  ##  ##  ##  ##  ##  ##  ##  ##  ##  ##  ##  ##  ##  ##  ##  ##  ##  ##  ##  ##  ##  ##  ##  ##  ##  ##  ##  ##  ##  ##  ##  ##  ##  ##  ##  ##  ##  ##  ##  ##  ##  ##  ##  ##  ##  ##  ##  ##  ##  ##  ##  ##  ##  ##  ##  ##  ##  ##  ##  ##  ##  ##  ##  ##  ##  ##  ##  ##  ##  ##  ##  ##  ##  ##  ##  ##  ##  ##  ##  ##  ##  ##  ##  ##  ##  ##  ##  ##  ##  ##  ##  ##  ##  ##  ##  ##  ##  ##  ##  ##  ##  ##  ##  ##  ##  ##  ##  ##  ##  ##  ##  ##  ##  ##  ##  ##  ## [1987_2]24	: 6  2  2  3  2  3  2  2  1  6  3  2  2  3  7  2  4  6  2  3  11  2  3  --  6  ##  ##  ##  ##  ##  ##  ##  ##  ##  ##  ##  ##  ##  ##  ##  ##  ##  ##  ##  ##  ##  ##  ##  ##  ##  ##  ##  ##  ##  ##  ##  ##  ##  ##  ##  ##  ##  ##  ##  ##  ##  ##  ##  ##  ##  ##  ##  ##  ##  ##  ##  ##  ##  ##  ##  ##  ##  ##  ##  ##  ##  ##  ##  ##  ##  ##  ##  ##  ##  ##  ##  ##  ##  ##  ##  ##  ##  ##  ##  ##  ##  ##  ##  ##  ##  ##  ##  ##  ##  ##  ##  ##  ##  ##  ##  ##  ##  ##  ##  ##  ##  ##  ##  ##  ##  ##  ##  ##  ##  ##  ##  ##  ##  ##  ##  ##  ##  ##  ##  ##  ##  ##  ##  ##  ##  ##  ##  ##  ##  ##  ##  ##  ##  ##  ##  ##  ##  ##  ##  ##  ##  ##  ##  ##  ##  ##  ##  ##  ##  ##  ##  ##  ##  ##  ##  ##  ##  ##  ##  ##  ##  ##  ##  ##  ##  ##  ##  ##  ##  ##  ##  ##  ##  ##  ##  ##  ##  ##  ##  ##  ##  ##  ##  ##  ##  ##  ##  ##  ##  ##  ##  ##  ##  ##  ##  ##  ##  ##  ##  ##  ##  ##  ##  ##  ##  ##  ##  ##  ##  ##  ##  ##  ##  ##  ##  ##  ##  ##  ##  ##  ##  ##  ##  ##  ##  ##  ##  ##  ##  ##  ##  ##  ##  ##  ##  ##  ##  ##  ##  ##  ##  ##  ##  ##  ## [POLYNOR088_13_2]25	: 3  6  6  7  6  7  4  6  5  1  7  6  6  7  2  6  8  10  5  7  8  6  7  6  --  ##  ##  ##  ##  ##  ##  ##  ##  ##  ##  ##  ##  ##  ##  ##  ##  ##  ##  ##  ##  ##  ##  ##  ##  ##  ##  ##  ##  ##  ##  ##  ##  ##  ##  ##  ##  ##  ##  ##  ##  ##  ##  ##  ##  ##  ##  ##  ##  ##  ##  ##  ##  ##  ##  ##  ##  ##  ##  ##  ##  ##  ##  ##  ##  ##  ##  ##  ##  ##  ##  ##  ##  ##  ##  ##  ##  ##  ##  ##  ##  ##  ##  ##  ##  ##  ##  ##  ##  ##  ##  ##  ##  ##  ##  ##  ##  ##  ##  ##  ##  ##  ##  ##  ##  ##  ##  ##  ##  ##  ##  ##  ##  ##  ##  ##  ##  ##  ##  ##  ##  ##  ##  ##  ##  ##  ##  ##  ##  ##  ##  ##  ##  ##  ##  ##  ##  ##  ##  ##  ##  ##  ##  ##  ##  ##  ##  ##  ##  ##  ##  ##  ##  ##  ##  ##  ##  ##  ##  ##  ##  ##  ##  ##  ##  ##  ##  ##  ##  ##  ##  ##  ##  ##  ##  ##  ##  ##  ##  ##  ##  ##  ##  ##  ##  ##  ##  ##  ##  ##  ##  ##  ##  ##  ##  ##  ##  ##  ##  ##  ##  ##  ##  ##  ##  ##  ##  ##  ##  ##  ##  ##  ##  ##  ##  ##  ##  ##  ##  ##  ##  ##  ##  ##  ##  ##  ##  ##  ##  ##  ##  ##  ##  ##  ##  ##  ##  ##  ##  ##  ##  ##  ##  ##  ##  ## [1207_3]26	: ##  ##  ##  ##  ##  ##  ##  ##  ##  ##  ##  ##  ##  ##  ##  ##  ##  ##  ##  ##  ##  ##  ##  ##  ##  --  7  10  15  11  13  13  11  1  11  14  11  14  10  5  4  9  13  10  8  11  11  9  8  9  11  10  7  9  8  10  10  5  8  11  13  11  11  11  17  9  11  5  15  ##  ##  ##  ##  ##  ##  ##  ##  ##  ##  ##  ##  ##  ##  ##  ##  ##  ##  ##  ##  ##  ##  ##  ##  ##  ##  ##  ##  ##  ##  ##  ##  ##  ##  ##  ##  ##  ##  ##  ##  ##  ##  ##  ##  ##  ##  ##  ##  ##  ##  ##  ##  ##  ##  ##  ##  ##  ##  ##  ##  ##  ##  ##  ##  ##  ##  ##  ##  ##  ##  ##  ##  ##  ##  ##  ##  ##  ##  ##  ##  ##  ##  ##  ##  ##  ##  ##  ##  ##  ##  ##  ##  ##  ##  ##  ##  ##  ##  ##  ##  ##  ##  ##  ##  ##  ##  ##  ##  ##  ##  ##  ##  ##  ##  ##  ##  ##  ##  ##  ##  ##  ##  ##  ##  ##  ##  ##  ##  ##  ##  ##  ##  ##  ##  ##  ##  ##  ##  ##  ##  ##  ##  ##  ##  ##  ##  ##  ##  ##  ##  ##  ##  ##  ##  ##  ##  ##  ##  ##  ##  ##  ##  ##  ##  ##  ##  ##  ##  ##  ##  ##  ##  ##  ##  ##  ##  ##  ##  ##  ##  ##  ##  ##  ##  ##  ##  ##  ##  ##  ##  ##  ##  ##  ##  ##  ##  ##  ##  ##  ##  ## [1310_3]27	: ##  ##  ##  ##  ##  ##  ##  ##  ##  ##  ##  ##  ##  ##  ##  ##  ##  ##  ##  ##  ##  ##  ##  ##  ##  7  --  11  16  12  14  14  12  6  12  11  12  11  7  4  5  6  14  7  3  12  12  6  3  10  12  7  4  6  9  11  7  2  9  12  10  12  12  12  14  6  12  6  12  ##  ##  ##  ##  ##  ##  ##  ##  ##  ##  ##  ##  ##  ##  ##  ##  ##  ##  ##  ##  ##  ##  ##  ##  ##  ##  ##  ##  ##  ##  ##  ##  ##  ##  ##  ##  ##  ##  ##  ##  ##  ##  ##  ##  ##  ##  ##  ##  ##  ##  ##  ##  ##  ##  ##  ##  ##  ##  ##  ##  ##  ##  ##  ##  ##  ##  ##  ##  ##  ##  ##  ##  ##  ##  ##  ##  ##  ##  ##  ##  ##  ##  ##  ##  ##  ##  ##  ##  ##  ##  ##  ##  ##  ##  ##  ##  ##  ##  ##  ##  ##  ##  ##  ##  ##  ##  ##  ##  ##  ##  ##  ##  ##  ##  ##  ##  ##  ##  ##  ##  ##  ##  ##  ##  ##  ##  ##  ##  ##  ##  ##  ##  ##  ##  ##  ##  ##  ##  ##  ##  ##  ##  ##  ##  ##  ##  ##  ##  ##  ##  ##  ##  ##  ##  ##  ##  ##  ##  ##  ##  ##  ##  ##  ##  ##  ##  ##  ##  ##  ##  ##  ##  ##  ##  ##  ##  ##  ##  ##  ##  ##  ##  ##  ##  ##  ##  ##  ##  ##  ##  ##  ##  ##  ##  ##  ##  ##  ##  ##  ##  ## [2275_3]28	: ##  ##  ##  ##  ##  ##  ##  ##  ##  ##  ##  ##  ##  ##  ##  ##  ##  ##  ##  ##  ##  ##  ##  ##  ##  10  11  --  11  7  9  9  1  9  3  18  1  18  14  9  8  13  3  14  12  1  1  13  12  1  1  14  11  13  6  2  14  9  4  3  17  1  1  1  21  13  1  5  19  ##  ##  ##  ##  ##  ##  ##  ##  ##  ##  ##  ##  ##  ##  ##  ##  ##  ##  ##  ##  ##  ##  ##  ##  ##  ##  ##  ##  ##  ##  ##  ##  ##  ##  ##  ##  ##  ##  ##  ##  ##  ##  ##  ##  ##  ##  ##  ##  ##  ##  ##  ##  ##  ##  ##  ##  ##  ##  ##  ##  ##  ##  ##  ##  ##  ##  ##  ##  ##  ##  ##  ##  ##  ##  ##  ##  ##  ##  ##  ##  ##  ##  ##  ##  ##  ##  ##  ##  ##  ##  ##  ##  ##  ##  ##  ##  ##  ##  ##  ##  ##  ##  ##  ##  ##  ##  ##  ##  ##  ##  ##  ##  ##  ##  ##  ##  ##  ##  ##  ##  ##  ##  ##  ##  ##  ##  ##  ##  ##  ##  ##  ##  ##  ##  ##  ##  ##  ##  ##  ##  ##  ##  ##  ##  ##  ##  ##  ##  ##  ##  ##  ##  ##  ##  ##  ##  ##  ##  ##  ##  ##  ##  ##  ##  ##  ##  ##  ##  ##  ##  ##  ##  ##  ##  ##  ##  ##  ##  ##  ##  ##  ##  ##  ##  ##  ##  ##  ##  ##  ##  ##  ##  ##  ##  ##  ##  ##  ##  ##  ##  ## [2380_3]29	: ##  ##  ##  ##  ##  ##  ##  ##  ##  ##  ##  ##  ##  ##  ##  ##  ##  ##  ##  ##  ##  ##  ##  ##  ##  15  16  11  --  6  6  8  12  14  12  23  12  23  19  14  13  18  14  19  17  12  12  18  17  10  12  19  16  18  11  11  19  14  9  12  22  12  12  12  26  18  12  10  24  ##  ##  ##  ##  ##  ##  ##  ##  ##  ##  ##  ##  ##  ##  ##  ##  ##  ##  ##  ##  ##  ##  ##  ##  ##  ##  ##  ##  ##  ##  ##  ##  ##  ##  ##  ##  ##  ##  ##  ##  ##  ##  ##  ##  ##  ##  ##  ##  ##  ##  ##  ##  ##  ##  ##  ##  ##  ##  ##  ##  ##  ##  ##  ##  ##  ##  ##  ##  ##  ##  ##  ##  ##  ##  ##  ##  ##  ##  ##  ##  ##  ##  ##  ##  ##  ##  ##  ##  ##  ##  ##  ##  ##  ##  ##  ##  ##  ##  ##  ##  ##  ##  ##  ##  ##  ##  ##  ##  ##  ##  ##  ##  ##  ##  ##  ##  ##  ##  ##  ##  ##  ##  ##  ##  ##  ##  ##  ##  ##  ##  ##  ##  ##  ##  ##  ##  ##  ##  ##  ##  ##  ##  ##  ##  ##  ##  ##  ##  ##  ##  ##  ##  ##  ##  ##  ##  ##  ##  ##  ##  ##  ##  ##  ##  ##  ##  ##  ##  ##  ##  ##  ##  ##  ##  ##  ##  ##  ##  ##  ##  ##  ##  ##  ##  ##  ##  ##  ##  ##  ##  ##  ##  ##  ##  ##  ##  ##  ##  ##  ##  ## [2463_3]30	: ##  ##  ##  ##  ##  ##  ##  ##  ##  ##  ##  ##  ##  ##  ##  ##  ##  ##  ##  ##  ##  ##  ##  ##  ##  11  12  7  6  --  4  4  8  10  8  19  8  19  15  10  9  14  10  15  13  8  8  14  13  6  8  15  12  14  7  7  15  10  5  8  18  8  8  8  22  14  8  6  20  ##  ##  ##  ##  ##  ##  ##  ##  ##  ##  ##  ##  ##  ##  ##  ##  ##  ##  ##  ##  ##  ##  ##  ##  ##  ##  ##  ##  ##  ##  ##  ##  ##  ##  ##  ##  ##  ##  ##  ##  ##  ##  ##  ##  ##  ##  ##  ##  ##  ##  ##  ##  ##  ##  ##  ##  ##  ##  ##  ##  ##  ##  ##  ##  ##  ##  ##  ##  ##  ##  ##  ##  ##  ##  ##  ##  ##  ##  ##  ##  ##  ##  ##  ##  ##  ##  ##  ##  ##  ##  ##  ##  ##  ##  ##  ##  ##  ##  ##  ##  ##  ##  ##  ##  ##  ##  ##  ##  ##  ##  ##  ##  ##  ##  ##  ##  ##  ##  ##  ##  ##  ##  ##  ##  ##  ##  ##  ##  ##  ##  ##  ##  ##  ##  ##  ##  ##  ##  ##  ##  ##  ##  ##  ##  ##  ##  ##  ##  ##  ##  ##  ##  ##  ##  ##  ##  ##  ##  ##  ##  ##  ##  ##  ##  ##  ##  ##  ##  ##  ##  ##  ##  ##  ##  ##  ##  ##  ##  ##  ##  ##  ##  ##  ##  ##  ##  ##  ##  ##  ##  ##  ##  ##  ##  ##  ##  ##  ##  ##  ##  ## [2464_3]31	: ##  ##  ##  ##  ##  ##  ##  ##  ##  ##  ##  ##  ##  ##  ##  ##  ##  ##  ##  ##  ##  ##  ##  ##  ##  13  14  9  6  4  --  4  10  12  10  21  10  21  17  12  11  16  12  17  15  10  10  16  15  8  10  17  14  16  9  9  17  12  7  10  20  10  10  10  24  16  10  8  22  ##  ##  ##  ##  ##  ##  ##  ##  ##  ##  ##  ##  ##  ##  ##  ##  ##  ##  ##  ##  ##  ##  ##  ##  ##  ##  ##  ##  ##  ##  ##  ##  ##  ##  ##  ##  ##  ##  ##  ##  ##  ##  ##  ##  ##  ##  ##  ##  ##  ##  ##  ##  ##  ##  ##  ##  ##  ##  ##  ##  ##  ##  ##  ##  ##  ##  ##  ##  ##  ##  ##  ##  ##  ##  ##  ##  ##  ##  ##  ##  ##  ##  ##  ##  ##  ##  ##  ##  ##  ##  ##  ##  ##  ##  ##  ##  ##  ##  ##  ##  ##  ##  ##  ##  ##  ##  ##  ##  ##  ##  ##  ##  ##  ##  ##  ##  ##  ##  ##  ##  ##  ##  ##  ##  ##  ##  ##  ##  ##  ##  ##  ##  ##  ##  ##  ##  ##  ##  ##  ##  ##  ##  ##  ##  ##  ##  ##  ##  ##  ##  ##  ##  ##  ##  ##  ##  ##  ##  ##  ##  ##  ##  ##  ##  ##  ##  ##  ##  ##  ##  ##  ##  ##  ##  ##  ##  ##  ##  ##  ##  ##  ##  ##  ##  ##  ##  ##  ##  ##  ##  ##  ##  ##  ##  ##  ##  ##  ##  ##  ##  ## [2465_3]32	: ##  ##  ##  ##  ##  ##  ##  ##  ##  ##  ##  ##  ##  ##  ##  ##  ##  ##  ##  ##  ##  ##  ##  ##  ##  13  14  9  8  4  4  --  10  12  10  21  10  21  17  12  11  16  12  17  15  10  10  16  15  8  10  17  14  16  9  9  17  12  7  10  20  10  10  10  24  16  10  8  22  ##  ##  ##  ##  ##  ##  ##  ##  ##  ##  ##  ##  ##  ##  ##  ##  ##  ##  ##  ##  ##  ##  ##  ##  ##  ##  ##  ##  ##  ##  ##  ##  ##  ##  ##  ##  ##  ##  ##  ##  ##  ##  ##  ##  ##  ##  ##  ##  ##  ##  ##  ##  ##  ##  ##  ##  ##  ##  ##  ##  ##  ##  ##  ##  ##  ##  ##  ##  ##  ##  ##  ##  ##  ##  ##  ##  ##  ##  ##  ##  ##  ##  ##  ##  ##  ##  ##  ##  ##  ##  ##  ##  ##  ##  ##  ##  ##  ##  ##  ##  ##  ##  ##  ##  ##  ##  ##  ##  ##  ##  ##  ##  ##  ##  ##  ##  ##  ##  ##  ##  ##  ##  ##  ##  ##  ##  ##  ##  ##  ##  ##  ##  ##  ##  ##  ##  ##  ##  ##  ##  ##  ##  ##  ##  ##  ##  ##  ##  ##  ##  ##  ##  ##  ##  ##  ##  ##  ##  ##  ##  ##  ##  ##  ##  ##  ##  ##  ##  ##  ##  ##  ##  ##  ##  ##  ##  ##  ##  ##  ##  ##  ##  ##  ##  ##  ##  ##  ##  ##  ##  ##  ##  ##  ##  ##  ##  ##  ##  ##  ##  ## [2812_3]33	: ##  ##  ##  ##  ##  ##  ##  ##  ##  ##  ##  ##  ##  ##  ##  ##  ##  ##  ##  ##  ##  ##  ##  ##  ##  11  12  1  12  8  10  10  --  10  4  19  2  19  15  10  9  14  4  15  13  2  2  14  13  2  2  15  12  14  7  3  15  10  5  4  18  2  2  2  22  14  2  6  20  ##  ##  ##  ##  ##  ##  ##  ##  ##  ##  ##  ##  ##  ##  ##  ##  ##  ##  ##  ##  ##  ##  ##  ##  ##  ##  ##  ##  ##  ##  ##  ##  ##  ##  ##  ##  ##  ##  ##  ##  ##  ##  ##  ##  ##  ##  ##  ##  ##  ##  ##  ##  ##  ##  ##  ##  ##  ##  ##  ##  ##  ##  ##  ##  ##  ##  ##  ##  ##  ##  ##  ##  ##  ##  ##  ##  ##  ##  ##  ##  ##  ##  ##  ##  ##  ##  ##  ##  ##  ##  ##  ##  ##  ##  ##  ##  ##  ##  ##  ##  ##  ##  ##  ##  ##  ##  ##  ##  ##  ##  ##  ##  ##  ##  ##  ##  ##  ##  ##  ##  ##  ##  ##  ##  ##  ##  ##  ##  ##  ##  ##  ##  ##  ##  ##  ##  ##  ##  ##  ##  ##  ##  ##  ##  ##  ##  ##  ##  ##  ##  ##  ##  ##  ##  ##  ##  ##  ##  ##  ##  ##  ##  ##  ##  ##  ##  ##  ##  ##  ##  ##  ##  ##  ##  ##  ##  ##  ##  ##  ##  ##  ##  ##  ##  ##  ##  ##  ##  ##  ##  ##  ##  ##  ##  ##  ##  ##  ##  ##  ##  ## [2814_3]34	: ##  ##  ##  ##  ##  ##  ##  ##  ##  ##  ##  ##  ##  ##  ##  ##  ##  ##  ##  ##  ##  ##  ##  ##  ##  1  6  9  14  10  12  12  10  --  10  13  10  13  9  4  3  8  12  9  7  10  10  8  7  8  10  9  6  8  7  9  9  4  7  10  12  10  10  10  16  8  10  4  14  ##  ##  ##  ##  ##  ##  ##  ##  ##  ##  ##  ##  ##  ##  ##  ##  ##  ##  ##  ##  ##  ##  ##  ##  ##  ##  ##  ##  ##  ##  ##  ##  ##  ##  ##  ##  ##  ##  ##  ##  ##  ##  ##  ##  ##  ##  ##  ##  ##  ##  ##  ##  ##  ##  ##  ##  ##  ##  ##  ##  ##  ##  ##  ##  ##  ##  ##  ##  ##  ##  ##  ##  ##  ##  ##  ##  ##  ##  ##  ##  ##  ##  ##  ##  ##  ##  ##  ##  ##  ##  ##  ##  ##  ##  ##  ##  ##  ##  ##  ##  ##  ##  ##  ##  ##  ##  ##  ##  ##  ##  ##  ##  ##  ##  ##  ##  ##  ##  ##  ##  ##  ##  ##  ##  ##  ##  ##  ##  ##  ##  ##  ##  ##  ##  ##  ##  ##  ##  ##  ##  ##  ##  ##  ##  ##  ##  ##  ##  ##  ##  ##  ##  ##  ##  ##  ##  ##  ##  ##  ##  ##  ##  ##  ##  ##  ##  ##  ##  ##  ##  ##  ##  ##  ##  ##  ##  ##  ##  ##  ##  ##  ##  ##  ##  ##  ##  ##  ##  ##  ##  ##  ##  ##  ##  ##  ##  ##  ##  ##  ##  ## [2872_3]35	: ##  ##  ##  ##  ##  ##  ##  ##  ##  ##  ##  ##  ##  ##  ##  ##  ##  ##  ##  ##  ##  ##  ##  ##  ##  11  12  3  12  8  10  10  4  10  --  19  4  19  15  10  9  14  6  15  13  4  4  14  13  2  4  15  12  14  7  3  15  10  5  4  18  4  4  4  22  14  4  6  20  ##  ##  ##  ##  ##  ##  ##  ##  ##  ##  ##  ##  ##  ##  ##  ##  ##  ##  ##  ##  ##  ##  ##  ##  ##  ##  ##  ##  ##  ##  ##  ##  ##  ##  ##  ##  ##  ##  ##  ##  ##  ##  ##  ##  ##  ##  ##  ##  ##  ##  ##  ##  ##  ##  ##  ##  ##  ##  ##  ##  ##  ##  ##  ##  ##  ##  ##  ##  ##  ##  ##  ##  ##  ##  ##  ##  ##  ##  ##  ##  ##  ##  ##  ##  ##  ##  ##  ##  ##  ##  ##  ##  ##  ##  ##  ##  ##  ##  ##  ##  ##  ##  ##  ##  ##  ##  ##  ##  ##  ##  ##  ##  ##  ##  ##  ##  ##  ##  ##  ##  ##  ##  ##  ##  ##  ##  ##  ##  ##  ##  ##  ##  ##  ##  ##  ##  ##  ##  ##  ##  ##  ##  ##  ##  ##  ##  ##  ##  ##  ##  ##  ##  ##  ##  ##  ##  ##  ##  ##  ##  ##  ##  ##  ##  ##  ##  ##  ##  ##  ##  ##  ##  ##  ##  ##  ##  ##  ##  ##  ##  ##  ##  ##  ##  ##  ##  ##  ##  ##  ##  ##  ##  ##  ##  ##  ##  ##  ##  ##  ##  ## [2874_3]36	: ##  ##  ##  ##  ##  ##  ##  ##  ##  ##  ##  ##  ##  ##  ##  ##  ##  ##  ##  ##  ##  ##  ##  ##  ##  14  11  18  23  19  21  21  19  13  19  --  19  4  10  11  12  5  21  8  10  19  19  9  12  17  19  16  7  9  16  18  10  9  16  19  3  19  19  19  3  9  19  13  5  ##  ##  ##  ##  ##  ##  ##  ##  ##  ##  ##  ##  ##  ##  ##  ##  ##  ##  ##  ##  ##  ##  ##  ##  ##  ##  ##  ##  ##  ##  ##  ##  ##  ##  ##  ##  ##  ##  ##  ##  ##  ##  ##  ##  ##  ##  ##  ##  ##  ##  ##  ##  ##  ##  ##  ##  ##  ##  ##  ##  ##  ##  ##  ##  ##  ##  ##  ##  ##  ##  ##  ##  ##  ##  ##  ##  ##  ##  ##  ##  ##  ##  ##  ##  ##  ##  ##  ##  ##  ##  ##  ##  ##  ##  ##  ##  ##  ##  ##  ##  ##  ##  ##  ##  ##  ##  ##  ##  ##  ##  ##  ##  ##  ##  ##  ##  ##  ##  ##  ##  ##  ##  ##  ##  ##  ##  ##  ##  ##  ##  ##  ##  ##  ##  ##  ##  ##  ##  ##  ##  ##  ##  ##  ##  ##  ##  ##  ##  ##  ##  ##  ##  ##  ##  ##  ##  ##  ##  ##  ##  ##  ##  ##  ##  ##  ##  ##  ##  ##  ##  ##  ##  ##  ##  ##  ##  ##  ##  ##  ##  ##  ##  ##  ##  ##  ##  ##  ##  ##  ##  ##  ##  ##  ##  ##  ##  ##  ##  ##  ##  ## [2876_3]37	: ##  ##  ##  ##  ##  ##  ##  ##  ##  ##  ##  ##  ##  ##  ##  ##  ##  ##  ##  ##  ##  ##  ##  ##  ##  11  12  1  12  8  10  10  2  10  4  19  --  19  15  10  9  14  4  15  13  2  2  14  13  2  2  15  12  14  7  3  15  10  5  4  18  2  2  2  22  14  2  6  20  ##  ##  ##  ##  ##  ##  ##  ##  ##  ##  ##  ##  ##  ##  ##  ##  ##  ##  ##  ##  ##  ##  ##  ##  ##  ##  ##  ##  ##  ##  ##  ##  ##  ##  ##  ##  ##  ##  ##  ##  ##  ##  ##  ##  ##  ##  ##  ##  ##  ##  ##  ##  ##  ##  ##  ##  ##  ##  ##  ##  ##  ##  ##  ##  ##  ##  ##  ##  ##  ##  ##  ##  ##  ##  ##  ##  ##  ##  ##  ##  ##  ##  ##  ##  ##  ##  ##  ##  ##  ##  ##  ##  ##  ##  ##  ##  ##  ##  ##  ##  ##  ##  ##  ##  ##  ##  ##  ##  ##  ##  ##  ##  ##  ##  ##  ##  ##  ##  ##  ##  ##  ##  ##  ##  ##  ##  ##  ##  ##  ##  ##  ##  ##  ##  ##  ##  ##  ##  ##  ##  ##  ##  ##  ##  ##  ##  ##  ##  ##  ##  ##  ##  ##  ##  ##  ##  ##  ##  ##  ##  ##  ##  ##  ##  ##  ##  ##  ##  ##  ##  ##  ##  ##  ##  ##  ##  ##  ##  ##  ##  ##  ##  ##  ##  ##  ##  ##  ##  ##  ##  ##  ##  ##  ##  ##  ##  ##  ##  ##  ##  ## [2877_3]38	: ##  ##  ##  ##  ##  ##  ##  ##  ##  ##  ##  ##  ##  ##  ##  ##  ##  ##  ##  ##  ##  ##  ##  ##  ##  14  11  18  23  19  21  21  19  13  19  4  19  --  10  11  12  5  21  8  10  19  19  9  12  17  19  16  7  9  16  18  10  9  16  19  1  19  19  19  7  9  19  13  5  ##  ##  ##  ##  ##  ##  ##  ##  ##  ##  ##  ##  ##  ##  ##  ##  ##  ##  ##  ##  ##  ##  ##  ##  ##  ##  ##  ##  ##  ##  ##  ##  ##  ##  ##  ##  ##  ##  ##  ##  ##  ##  ##  ##  ##  ##  ##  ##  ##  ##  ##  ##  ##  ##  ##  ##  ##  ##  ##  ##  ##  ##  ##  ##  ##  ##  ##  ##  ##  ##  ##  ##  ##  ##  ##  ##  ##  ##  ##  ##  ##  ##  ##  ##  ##  ##  ##  ##  ##  ##  ##  ##  ##  ##  ##  ##  ##  ##  ##  ##  ##  ##  ##  ##  ##  ##  ##  ##  ##  ##  ##  ##  ##  ##  ##  ##  ##  ##  ##  ##  ##  ##  ##  ##  ##  ##  ##  ##  ##  ##  ##  ##  ##  ##  ##  ##  ##  ##  ##  ##  ##  ##  ##  ##  ##  ##  ##  ##  ##  ##  ##  ##  ##  ##  ##  ##  ##  ##  ##  ##  ##  ##  ##  ##  ##  ##  ##  ##  ##  ##  ##  ##  ##  ##  ##  ##  ##  ##  ##  ##  ##  ##  ##  ##  ##  ##  ##  ##  ##  ##  ##  ##  ##  ##  ##  ##  ##  ##  ##  ##  ## [2879_3]39	: ##  ##  ##  ##  ##  ##  ##  ##  ##  ##  ##  ##  ##  ##  ##  ##  ##  ##  ##  ##  ##  ##  ##  ##  ##  10  7  14  19  15  17  17  15  9  15  10  15  10  --  7  8  5  17  6  6  15  15  5  8  13  15  12  3  3  12  14  6  5  12  15  9  15  15  15  13  5  15  9  11  ##  ##  ##  ##  ##  ##  ##  ##  ##  ##  ##  ##  ##  ##  ##  ##  ##  ##  ##  ##  ##  ##  ##  ##  ##  ##  ##  ##  ##  ##  ##  ##  ##  ##  ##  ##  ##  ##  ##  ##  ##  ##  ##  ##  ##  ##  ##  ##  ##  ##  ##  ##  ##  ##  ##  ##  ##  ##  ##  ##  ##  ##  ##  ##  ##  ##  ##  ##  ##  ##  ##  ##  ##  ##  ##  ##  ##  ##  ##  ##  ##  ##  ##  ##  ##  ##  ##  ##  ##  ##  ##  ##  ##  ##  ##  ##  ##  ##  ##  ##  ##  ##  ##  ##  ##  ##  ##  ##  ##  ##  ##  ##  ##  ##  ##  ##  ##  ##  ##  ##  ##  ##  ##  ##  ##  ##  ##  ##  ##  ##  ##  ##  ##  ##  ##  ##  ##  ##  ##  ##  ##  ##  ##  ##  ##  ##  ##  ##  ##  ##  ##  ##  ##  ##  ##  ##  ##  ##  ##  ##  ##  ##  ##  ##  ##  ##  ##  ##  ##  ##  ##  ##  ##  ##  ##  ##  ##  ##  ##  ##  ##  ##  ##  ##  ##  ##  ##  ##  ##  ##  ##  ##  ##  ##  ##  ##  ##  ##  ##  ##  ## [2880_3]40	: ##  ##  ##  ##  ##  ##  ##  ##  ##  ##  ##  ##  ##  ##  ##  ##  ##  ##  ##  ##  ##  ##  ##  ##  ##  5  4  9  14  10  12  12  10  4  10  11  10  11  7  --  3  6  12  7  5  10  10  6  5  8  10  7  4  6  7  9  7  2  7  10  10  10  10  10  14  6  10  4  12  ##  ##  ##  ##  ##  ##  ##  ##  ##  ##  ##  ##  ##  ##  ##  ##  ##  ##  ##  ##  ##  ##  ##  ##  ##  ##  ##  ##  ##  ##  ##  ##  ##  ##  ##  ##  ##  ##  ##  ##  ##  ##  ##  ##  ##  ##  ##  ##  ##  ##  ##  ##  ##  ##  ##  ##  ##  ##  ##  ##  ##  ##  ##  ##  ##  ##  ##  ##  ##  ##  ##  ##  ##  ##  ##  ##  ##  ##  ##  ##  ##  ##  ##  ##  ##  ##  ##  ##  ##  ##  ##  ##  ##  ##  ##  ##  ##  ##  ##  ##  ##  ##  ##  ##  ##  ##  ##  ##  ##  ##  ##  ##  ##  ##  ##  ##  ##  ##  ##  ##  ##  ##  ##  ##  ##  ##  ##  ##  ##  ##  ##  ##  ##  ##  ##  ##  ##  ##  ##  ##  ##  ##  ##  ##  ##  ##  ##  ##  ##  ##  ##  ##  ##  ##  ##  ##  ##  ##  ##  ##  ##  ##  ##  ##  ##  ##  ##  ##  ##  ##  ##  ##  ##  ##  ##  ##  ##  ##  ##  ##  ##  ##  ##  ##  ##  ##  ##  ##  ##  ##  ##  ##  ##  ##  ##  ##  ##  ##  ##  ##  ## [2881_3]41	: ##  ##  ##  ##  ##  ##  ##  ##  ##  ##  ##  ##  ##  ##  ##  ##  ##  ##  ##  ##  ##  ##  ##  ##  ##  4  5  8  13  9  11  11  9  3  9  12  9  12  8  3  --  7  11  8  6  9  9  7  6  7  9  8  5  7  6  8  8  3  6  9  11  9  9  9  15  7  9  3  13  ##  ##  ##  ##  ##  ##  ##  ##  ##  ##  ##  ##  ##  ##  ##  ##  ##  ##  ##  ##  ##  ##  ##  ##  ##  ##  ##  ##  ##  ##  ##  ##  ##  ##  ##  ##  ##  ##  ##  ##  ##  ##  ##  ##  ##  ##  ##  ##  ##  ##  ##  ##  ##  ##  ##  ##  ##  ##  ##  ##  ##  ##  ##  ##  ##  ##  ##  ##  ##  ##  ##  ##  ##  ##  ##  ##  ##  ##  ##  ##  ##  ##  ##  ##  ##  ##  ##  ##  ##  ##  ##  ##  ##  ##  ##  ##  ##  ##  ##  ##  ##  ##  ##  ##  ##  ##  ##  ##  ##  ##  ##  ##  ##  ##  ##  ##  ##  ##  ##  ##  ##  ##  ##  ##  ##  ##  ##  ##  ##  ##  ##  ##  ##  ##  ##  ##  ##  ##  ##  ##  ##  ##  ##  ##  ##  ##  ##  ##  ##  ##  ##  ##  ##  ##  ##  ##  ##  ##  ##  ##  ##  ##  ##  ##  ##  ##  ##  ##  ##  ##  ##  ##  ##  ##  ##  ##  ##  ##  ##  ##  ##  ##  ##  ##  ##  ##  ##  ##  ##  ##  ##  ##  ##  ##  ##  ##  ##  ##  ##  ##  ## [2882_3]42	: ##  ##  ##  ##  ##  ##  ##  ##  ##  ##  ##  ##  ##  ##  ##  ##  ##  ##  ##  ##  ##  ##  ##  ##  ##  9  6  13  18  14  16  16  14  8  14  5  14  5  5  6  7  --  16  3  5  14  14  4  7  12  14  11  2  4  11  13  5  4  11  14  4  14  14  14  8  4  14  8  6  ##  ##  ##  ##  ##  ##  ##  ##  ##  ##  ##  ##  ##  ##  ##  ##  ##  ##  ##  ##  ##  ##  ##  ##  ##  ##  ##  ##  ##  ##  ##  ##  ##  ##  ##  ##  ##  ##  ##  ##  ##  ##  ##  ##  ##  ##  ##  ##  ##  ##  ##  ##  ##  ##  ##  ##  ##  ##  ##  ##  ##  ##  ##  ##  ##  ##  ##  ##  ##  ##  ##  ##  ##  ##  ##  ##  ##  ##  ##  ##  ##  ##  ##  ##  ##  ##  ##  ##  ##  ##  ##  ##  ##  ##  ##  ##  ##  ##  ##  ##  ##  ##  ##  ##  ##  ##  ##  ##  ##  ##  ##  ##  ##  ##  ##  ##  ##  ##  ##  ##  ##  ##  ##  ##  ##  ##  ##  ##  ##  ##  ##  ##  ##  ##  ##  ##  ##  ##  ##  ##  ##  ##  ##  ##  ##  ##  ##  ##  ##  ##  ##  ##  ##  ##  ##  ##  ##  ##  ##  ##  ##  ##  ##  ##  ##  ##  ##  ##  ##  ##  ##  ##  ##  ##  ##  ##  ##  ##  ##  ##  ##  ##  ##  ##  ##  ##  ##  ##  ##  ##  ##  ##  ##  ##  ##  ##  ##  ##  ##  ##  ## [2883_3]43	: ##  ##  ##  ##  ##  ##  ##  ##  ##  ##  ##  ##  ##  ##  ##  ##  ##  ##  ##  ##  ##  ##  ##  ##  ##  13  14  3  14  10  12  12  4  12  6  21  4  21  17  12  11  16  --  17  15  4  4  16  15  4  4  17  14  16  9  5  17  12  7  6  20  4  4  2  24  16  4  8  22  ##  ##  ##  ##  ##  ##  ##  ##  ##  ##  ##  ##  ##  ##  ##  ##  ##  ##  ##  ##  ##  ##  ##  ##  ##  ##  ##  ##  ##  ##  ##  ##  ##  ##  ##  ##  ##  ##  ##  ##  ##  ##  ##  ##  ##  ##  ##  ##  ##  ##  ##  ##  ##  ##  ##  ##  ##  ##  ##  ##  ##  ##  ##  ##  ##  ##  ##  ##  ##  ##  ##  ##  ##  ##  ##  ##  ##  ##  ##  ##  ##  ##  ##  ##  ##  ##  ##  ##  ##  ##  ##  ##  ##  ##  ##  ##  ##  ##  ##  ##  ##  ##  ##  ##  ##  ##  ##  ##  ##  ##  ##  ##  ##  ##  ##  ##  ##  ##  ##  ##  ##  ##  ##  ##  ##  ##  ##  ##  ##  ##  ##  ##  ##  ##  ##  ##  ##  ##  ##  ##  ##  ##  ##  ##  ##  ##  ##  ##  ##  ##  ##  ##  ##  ##  ##  ##  ##  ##  ##  ##  ##  ##  ##  ##  ##  ##  ##  ##  ##  ##  ##  ##  ##  ##  ##  ##  ##  ##  ##  ##  ##  ##  ##  ##  ##  ##  ##  ##  ##  ##  ##  ##  ##  ##  ##  ##  ##  ##  ##  ##  ## [2884_3]44	: ##  ##  ##  ##  ##  ##  ##  ##  ##  ##  ##  ##  ##  ##  ##  ##  ##  ##  ##  ##  ##  ##  ##  ##  ##  10  7  14  19  15  17  17  15  9  15  8  15  8  6  7  8  3  17  --  6  15  15  5  8  13  15  12  3  5  12  14  6  5  12  15  7  15  15  15  11  5  15  9  9  ##  ##  ##  ##  ##  ##  ##  ##  ##  ##  ##  ##  ##  ##  ##  ##  ##  ##  ##  ##  ##  ##  ##  ##  ##  ##  ##  ##  ##  ##  ##  ##  ##  ##  ##  ##  ##  ##  ##  ##  ##  ##  ##  ##  ##  ##  ##  ##  ##  ##  ##  ##  ##  ##  ##  ##  ##  ##  ##  ##  ##  ##  ##  ##  ##  ##  ##  ##  ##  ##  ##  ##  ##  ##  ##  ##  ##  ##  ##  ##  ##  ##  ##  ##  ##  ##  ##  ##  ##  ##  ##  ##  ##  ##  ##  ##  ##  ##  ##  ##  ##  ##  ##  ##  ##  ##  ##  ##  ##  ##  ##  ##  ##  ##  ##  ##  ##  ##  ##  ##  ##  ##  ##  ##  ##  ##  ##  ##  ##  ##  ##  ##  ##  ##  ##  ##  ##  ##  ##  ##  ##  ##  ##  ##  ##  ##  ##  ##  ##  ##  ##  ##  ##  ##  ##  ##  ##  ##  ##  ##  ##  ##  ##  ##  ##  ##  ##  ##  ##  ##  ##  ##  ##  ##  ##  ##  ##  ##  ##  ##  ##  ##  ##  ##  ##  ##  ##  ##  ##  ##  ##  ##  ##  ##  ##  ##  ##  ##  ##  ##  ## [2885_3]45	: ##  ##  ##  ##  ##  ##  ##  ##  ##  ##  ##  ##  ##  ##  ##  ##  ##  ##  ##  ##  ##  ##  ##  ##  ##  8  3  12  17  13  15  15  13  7  13  10  13  10  6  5  6  5  15  6  --  13  13  5  4  11  13  8  3  5  10  12  6  3  10  13  9  13  13  13  13  5  13  7  11  ##  ##  ##  ##  ##  ##  ##  ##  ##  ##  ##  ##  ##  ##  ##  ##  ##  ##  ##  ##  ##  ##  ##  ##  ##  ##  ##  ##  ##  ##  ##  ##  ##  ##  ##  ##  ##  ##  ##  ##  ##  ##  ##  ##  ##  ##  ##  ##  ##  ##  ##  ##  ##  ##  ##  ##  ##  ##  ##  ##  ##  ##  ##  ##  ##  ##  ##  ##  ##  ##  ##  ##  ##  ##  ##  ##  ##  ##  ##  ##  ##  ##  ##  ##  ##  ##  ##  ##  ##  ##  ##  ##  ##  ##  ##  ##  ##  ##  ##  ##  ##  ##  ##  ##  ##  ##  ##  ##  ##  ##  ##  ##  ##  ##  ##  ##  ##  ##  ##  ##  ##  ##  ##  ##  ##  ##  ##  ##  ##  ##  ##  ##  ##  ##  ##  ##  ##  ##  ##  ##  ##  ##  ##  ##  ##  ##  ##  ##  ##  ##  ##  ##  ##  ##  ##  ##  ##  ##  ##  ##  ##  ##  ##  ##  ##  ##  ##  ##  ##  ##  ##  ##  ##  ##  ##  ##  ##  ##  ##  ##  ##  ##  ##  ##  ##  ##  ##  ##  ##  ##  ##  ##  ##  ##  ##  ##  ##  ##  ##  ##  ## [2886_3]46	: ##  ##  ##  ##  ##  ##  ##  ##  ##  ##  ##  ##  ##  ##  ##  ##  ##  ##  ##  ##  ##  ##  ##  ##  ##  11  12  1  12  8  10  10  2  10  4  19  2  19  15  10  9  14  4  15  13  --  2  14  13  2  2  15  12  14  7  3  15  10  5  4  18  2  2  2  22  14  2  6  20  ##  ##  ##  ##  ##  ##  ##  ##  ##  ##  ##  ##  ##  ##  ##  ##  ##  ##  ##  ##  ##  ##  ##  ##  ##  ##  ##  ##  ##  ##  ##  ##  ##  ##  ##  ##  ##  ##  ##  ##  ##  ##  ##  ##  ##  ##  ##  ##  ##  ##  ##  ##  ##  ##  ##  ##  ##  ##  ##  ##  ##  ##  ##  ##  ##  ##  ##  ##  ##  ##  ##  ##  ##  ##  ##  ##  ##  ##  ##  ##  ##  ##  ##  ##  ##  ##  ##  ##  ##  ##  ##  ##  ##  ##  ##  ##  ##  ##  ##  ##  ##  ##  ##  ##  ##  ##  ##  ##  ##  ##  ##  ##  ##  ##  ##  ##  ##  ##  ##  ##  ##  ##  ##  ##  ##  ##  ##  ##  ##  ##  ##  ##  ##  ##  ##  ##  ##  ##  ##  ##  ##  ##  ##  ##  ##  ##  ##  ##  ##  ##  ##  ##  ##  ##  ##  ##  ##  ##  ##  ##  ##  ##  ##  ##  ##  ##  ##  ##  ##  ##  ##  ##  ##  ##  ##  ##  ##  ##  ##  ##  ##  ##  ##  ##  ##  ##  ##  ##  ##  ##  ##  ##  ##  ##  ##  ##  ##  ##  ##  ##  ## [2887_3]47	: ##  ##  ##  ##  ##  ##  ##  ##  ##  ##  ##  ##  ##  ##  ##  ##  ##  ##  ##  ##  ##  ##  ##  ##  ##  11  12  1  12  8  10  10  2  10  4  19  2  19  15  10  9  14  4  15  13  2  --  14  13  2  2  15  12  14  7  3  15  10  5  4  18  2  2  2  22  14  2  6  20  ##  ##  ##  ##  ##  ##  ##  ##  ##  ##  ##  ##  ##  ##  ##  ##  ##  ##  ##  ##  ##  ##  ##  ##  ##  ##  ##  ##  ##  ##  ##  ##  ##  ##  ##  ##  ##  ##  ##  ##  ##  ##  ##  ##  ##  ##  ##  ##  ##  ##  ##  ##  ##  ##  ##  ##  ##  ##  ##  ##  ##  ##  ##  ##  ##  ##  ##  ##  ##  ##  ##  ##  ##  ##  ##  ##  ##  ##  ##  ##  ##  ##  ##  ##  ##  ##  ##  ##  ##  ##  ##  ##  ##  ##  ##  ##  ##  ##  ##  ##  ##  ##  ##  ##  ##  ##  ##  ##  ##  ##  ##  ##  ##  ##  ##  ##  ##  ##  ##  ##  ##  ##  ##  ##  ##  ##  ##  ##  ##  ##  ##  ##  ##  ##  ##  ##  ##  ##  ##  ##  ##  ##  ##  ##  ##  ##  ##  ##  ##  ##  ##  ##  ##  ##  ##  ##  ##  ##  ##  ##  ##  ##  ##  ##  ##  ##  ##  ##  ##  ##  ##  ##  ##  ##  ##  ##  ##  ##  ##  ##  ##  ##  ##  ##  ##  ##  ##  ##  ##  ##  ##  ##  ##  ##  ##  ##  ##  ##  ##  ##  ## [2888_3]48	: ##  ##  ##  ##  ##  ##  ##  ##  ##  ##  ##  ##  ##  ##  ##  ##  ##  ##  ##  ##  ##  ##  ##  ##  ##  9  6  13  18  14  16  16  14  8  14  9  14  9  5  6  7  4  16  5  5  14  14  --  7  12  14  11  2  4  11  13  5  4  11  14  8  14  14  14  12  4  14  8  10  ##  ##  ##  ##  ##  ##  ##  ##  ##  ##  ##  ##  ##  ##  ##  ##  ##  ##  ##  ##  ##  ##  ##  ##  ##  ##  ##  ##  ##  ##  ##  ##  ##  ##  ##  ##  ##  ##  ##  ##  ##  ##  ##  ##  ##  ##  ##  ##  ##  ##  ##  ##  ##  ##  ##  ##  ##  ##  ##  ##  ##  ##  ##  ##  ##  ##  ##  ##  ##  ##  ##  ##  ##  ##  ##  ##  ##  ##  ##  ##  ##  ##  ##  ##  ##  ##  ##  ##  ##  ##  ##  ##  ##  ##  ##  ##  ##  ##  ##  ##  ##  ##  ##  ##  ##  ##  ##  ##  ##  ##  ##  ##  ##  ##  ##  ##  ##  ##  ##  ##  ##  ##  ##  ##  ##  ##  ##  ##  ##  ##  ##  ##  ##  ##  ##  ##  ##  ##  ##  ##  ##  ##  ##  ##  ##  ##  ##  ##  ##  ##  ##  ##  ##  ##  ##  ##  ##  ##  ##  ##  ##  ##  ##  ##  ##  ##  ##  ##  ##  ##  ##  ##  ##  ##  ##  ##  ##  ##  ##  ##  ##  ##  ##  ##  ##  ##  ##  ##  ##  ##  ##  ##  ##  ##  ##  ##  ##  ##  ##  ##  ## [2889_3]49	: ##  ##  ##  ##  ##  ##  ##  ##  ##  ##  ##  ##  ##  ##  ##  ##  ##  ##  ##  ##  ##  ##  ##  ##  ##  8  3  12  17  13  15  15  13  7  13  12  13  12  8  5  6  7  15  8  4  13  13  7  --  11  13  8  5  7  10  12  8  3  10  13  11  13  13  13  15  7  13  7  13  ##  ##  ##  ##  ##  ##  ##  ##  ##  ##  ##  ##  ##  ##  ##  ##  ##  ##  ##  ##  ##  ##  ##  ##  ##  ##  ##  ##  ##  ##  ##  ##  ##  ##  ##  ##  ##  ##  ##  ##  ##  ##  ##  ##  ##  ##  ##  ##  ##  ##  ##  ##  ##  ##  ##  ##  ##  ##  ##  ##  ##  ##  ##  ##  ##  ##  ##  ##  ##  ##  ##  ##  ##  ##  ##  ##  ##  ##  ##  ##  ##  ##  ##  ##  ##  ##  ##  ##  ##  ##  ##  ##  ##  ##  ##  ##  ##  ##  ##  ##  ##  ##  ##  ##  ##  ##  ##  ##  ##  ##  ##  ##  ##  ##  ##  ##  ##  ##  ##  ##  ##  ##  ##  ##  ##  ##  ##  ##  ##  ##  ##  ##  ##  ##  ##  ##  ##  ##  ##  ##  ##  ##  ##  ##  ##  ##  ##  ##  ##  ##  ##  ##  ##  ##  ##  ##  ##  ##  ##  ##  ##  ##  ##  ##  ##  ##  ##  ##  ##  ##  ##  ##  ##  ##  ##  ##  ##  ##  ##  ##  ##  ##  ##  ##  ##  ##  ##  ##  ##  ##  ##  ##  ##  ##  ##  ##  ##  ##  ##  ##  ## [2890_3]50	: ##  ##  ##  ##  ##  ##  ##  ##  ##  ##  ##  ##  ##  ##  ##  ##  ##  ##  ##  ##  ##  ##  ##  ##  ##  9  10  1  10  6  8  8  2  8  2  17  2  17  13  8  7  12  4  13  11  2  2  12  11  --  2  13  10  12  5  1  13  8  3  2  16  2  2  2  20  12  2  4  18  ##  ##  ##  ##  ##  ##  ##  ##  ##  ##  ##  ##  ##  ##  ##  ##  ##  ##  ##  ##  ##  ##  ##  ##  ##  ##  ##  ##  ##  ##  ##  ##  ##  ##  ##  ##  ##  ##  ##  ##  ##  ##  ##  ##  ##  ##  ##  ##  ##  ##  ##  ##  ##  ##  ##  ##  ##  ##  ##  ##  ##  ##  ##  ##  ##  ##  ##  ##  ##  ##  ##  ##  ##  ##  ##  ##  ##  ##  ##  ##  ##  ##  ##  ##  ##  ##  ##  ##  ##  ##  ##  ##  ##  ##  ##  ##  ##  ##  ##  ##  ##  ##  ##  ##  ##  ##  ##  ##  ##  ##  ##  ##  ##  ##  ##  ##  ##  ##  ##  ##  ##  ##  ##  ##  ##  ##  ##  ##  ##  ##  ##  ##  ##  ##  ##  ##  ##  ##  ##  ##  ##  ##  ##  ##  ##  ##  ##  ##  ##  ##  ##  ##  ##  ##  ##  ##  ##  ##  ##  ##  ##  ##  ##  ##  ##  ##  ##  ##  ##  ##  ##  ##  ##  ##  ##  ##  ##  ##  ##  ##  ##  ##  ##  ##  ##  ##  ##  ##  ##  ##  ##  ##  ##  ##  ##  ##  ##  ##  ##  ##  ## [2891_3]51	: ##  ##  ##  ##  ##  ##  ##  ##  ##  ##  ##  ##  ##  ##  ##  ##  ##  ##  ##  ##  ##  ##  ##  ##  ##  11  12  1  12  8  10  10  2  10  4  19  2  19  15  10  9  14  4  15  13  2  2  14  13  2  --  15  12  14  7  3  15  10  5  4  18  2  2  2  22  14  2  6  20  ##  ##  ##  ##  ##  ##  ##  ##  ##  ##  ##  ##  ##  ##  ##  ##  ##  ##  ##  ##  ##  ##  ##  ##  ##  ##  ##  ##  ##  ##  ##  ##  ##  ##  ##  ##  ##  ##  ##  ##  ##  ##  ##  ##  ##  ##  ##  ##  ##  ##  ##  ##  ##  ##  ##  ##  ##  ##  ##  ##  ##  ##  ##  ##  ##  ##  ##  ##  ##  ##  ##  ##  ##  ##  ##  ##  ##  ##  ##  ##  ##  ##  ##  ##  ##  ##  ##  ##  ##  ##  ##  ##  ##  ##  ##  ##  ##  ##  ##  ##  ##  ##  ##  ##  ##  ##  ##  ##  ##  ##  ##  ##  ##  ##  ##  ##  ##  ##  ##  ##  ##  ##  ##  ##  ##  ##  ##  ##  ##  ##  ##  ##  ##  ##  ##  ##  ##  ##  ##  ##  ##  ##  ##  ##  ##  ##  ##  ##  ##  ##  ##  ##  ##  ##  ##  ##  ##  ##  ##  ##  ##  ##  ##  ##  ##  ##  ##  ##  ##  ##  ##  ##  ##  ##  ##  ##  ##  ##  ##  ##  ##  ##  ##  ##  ##  ##  ##  ##  ##  ##  ##  ##  ##  ##  ##  ##  ##  ##  ##  ##  ## [2901_3]52	: ##  ##  ##  ##  ##  ##  ##  ##  ##  ##  ##  ##  ##  ##  ##  ##  ##  ##  ##  ##  ##  ##  ##  ##  ##  10  7  14  19  15  17  17  15  9  15  16  15  16  12  7  8  11  17  12  8  15  15  11  8  13  15  --  9  11  12  14  12  7  12  15  15  15  15  15  19  11  15  9  17  ##  ##  ##  ##  ##  ##  ##  ##  ##  ##  ##  ##  ##  ##  ##  ##  ##  ##  ##  ##  ##  ##  ##  ##  ##  ##  ##  ##  ##  ##  ##  ##  ##  ##  ##  ##  ##  ##  ##  ##  ##  ##  ##  ##  ##  ##  ##  ##  ##  ##  ##  ##  ##  ##  ##  ##  ##  ##  ##  ##  ##  ##  ##  ##  ##  ##  ##  ##  ##  ##  ##  ##  ##  ##  ##  ##  ##  ##  ##  ##  ##  ##  ##  ##  ##  ##  ##  ##  ##  ##  ##  ##  ##  ##  ##  ##  ##  ##  ##  ##  ##  ##  ##  ##  ##  ##  ##  ##  ##  ##  ##  ##  ##  ##  ##  ##  ##  ##  ##  ##  ##  ##  ##  ##  ##  ##  ##  ##  ##  ##  ##  ##  ##  ##  ##  ##  ##  ##  ##  ##  ##  ##  ##  ##  ##  ##  ##  ##  ##  ##  ##  ##  ##  ##  ##  ##  ##  ##  ##  ##  ##  ##  ##  ##  ##  ##  ##  ##  ##  ##  ##  ##  ##  ##  ##  ##  ##  ##  ##  ##  ##  ##  ##  ##  ##  ##  ##  ##  ##  ##  ##  ##  ##  ##  ##  ##  ##  ##  ##  ##  ## [2906_3]53	: ##  ##  ##  ##  ##  ##  ##  ##  ##  ##  ##  ##  ##  ##  ##  ##  ##  ##  ##  ##  ##  ##  ##  ##  ##  7  4  11  16  12  14  14  12  6  12  7  12  7  3  4  5  2  14  3  3  12  12  2  5  10  12  9  --  2  9  11  3  2  9  12  6  12  12  12  10  2  12  6  8  ##  ##  ##  ##  ##  ##  ##  ##  ##  ##  ##  ##  ##  ##  ##  ##  ##  ##  ##  ##  ##  ##  ##  ##  ##  ##  ##  ##  ##  ##  ##  ##  ##  ##  ##  ##  ##  ##  ##  ##  ##  ##  ##  ##  ##  ##  ##  ##  ##  ##  ##  ##  ##  ##  ##  ##  ##  ##  ##  ##  ##  ##  ##  ##  ##  ##  ##  ##  ##  ##  ##  ##  ##  ##  ##  ##  ##  ##  ##  ##  ##  ##  ##  ##  ##  ##  ##  ##  ##  ##  ##  ##  ##  ##  ##  ##  ##  ##  ##  ##  ##  ##  ##  ##  ##  ##  ##  ##  ##  ##  ##  ##  ##  ##  ##  ##  ##  ##  ##  ##  ##  ##  ##  ##  ##  ##  ##  ##  ##  ##  ##  ##  ##  ##  ##  ##  ##  ##  ##  ##  ##  ##  ##  ##  ##  ##  ##  ##  ##  ##  ##  ##  ##  ##  ##  ##  ##  ##  ##  ##  ##  ##  ##  ##  ##  ##  ##  ##  ##  ##  ##  ##  ##  ##  ##  ##  ##  ##  ##  ##  ##  ##  ##  ##  ##  ##  ##  ##  ##  ##  ##  ##  ##  ##  ##  ##  ##  ##  ##  ##  ## [2907_3]54	: ##  ##  ##  ##  ##  ##  ##  ##  ##  ##  ##  ##  ##  ##  ##  ##  ##  ##  ##  ##  ##  ##  ##  ##  ##  9  6  13  18  14  16  16  14  8  14  9  14  9  3  6  7  4  16  5  5  14  14  4  7  12  14  11  2  --  11  13  5  4  11  14  8  14  14  14  12  4  14  8  10  ##  ##  ##  ##  ##  ##  ##  ##  ##  ##  ##  ##  ##  ##  ##  ##  ##  ##  ##  ##  ##  ##  ##  ##  ##  ##  ##  ##  ##  ##  ##  ##  ##  ##  ##  ##  ##  ##  ##  ##  ##  ##  ##  ##  ##  ##  ##  ##  ##  ##  ##  ##  ##  ##  ##  ##  ##  ##  ##  ##  ##  ##  ##  ##  ##  ##  ##  ##  ##  ##  ##  ##  ##  ##  ##  ##  ##  ##  ##  ##  ##  ##  ##  ##  ##  ##  ##  ##  ##  ##  ##  ##  ##  ##  ##  ##  ##  ##  ##  ##  ##  ##  ##  ##  ##  ##  ##  ##  ##  ##  ##  ##  ##  ##  ##  ##  ##  ##  ##  ##  ##  ##  ##  ##  ##  ##  ##  ##  ##  ##  ##  ##  ##  ##  ##  ##  ##  ##  ##  ##  ##  ##  ##  ##  ##  ##  ##  ##  ##  ##  ##  ##  ##  ##  ##  ##  ##  ##  ##  ##  ##  ##  ##  ##  ##  ##  ##  ##  ##  ##  ##  ##  ##  ##  ##  ##  ##  ##  ##  ##  ##  ##  ##  ##  ##  ##  ##  ##  ##  ##  ##  ##  ##  ##  ##  ##  ##  ##  ##  ##  ## [2908_3]55	: ##  ##  ##  ##  ##  ##  ##  ##  ##  ##  ##  ##  ##  ##  ##  ##  ##  ##  ##  ##  ##  ##  ##  ##  ##  8  9  6  11  7  9  9  7  7  7  16  7  16  12  7  6  11  9  12  10  7  7  11  10  5  7  12  9  11  --  6  12  7  4  7  15  7  7  7  19  11  7  3  17  ##  ##  ##  ##  ##  ##  ##  ##  ##  ##  ##  ##  ##  ##  ##  ##  ##  ##  ##  ##  ##  ##  ##  ##  ##  ##  ##  ##  ##  ##  ##  ##  ##  ##  ##  ##  ##  ##  ##  ##  ##  ##  ##  ##  ##  ##  ##  ##  ##  ##  ##  ##  ##  ##  ##  ##  ##  ##  ##  ##  ##  ##  ##  ##  ##  ##  ##  ##  ##  ##  ##  ##  ##  ##  ##  ##  ##  ##  ##  ##  ##  ##  ##  ##  ##  ##  ##  ##  ##  ##  ##  ##  ##  ##  ##  ##  ##  ##  ##  ##  ##  ##  ##  ##  ##  ##  ##  ##  ##  ##  ##  ##  ##  ##  ##  ##  ##  ##  ##  ##  ##  ##  ##  ##  ##  ##  ##  ##  ##  ##  ##  ##  ##  ##  ##  ##  ##  ##  ##  ##  ##  ##  ##  ##  ##  ##  ##  ##  ##  ##  ##  ##  ##  ##  ##  ##  ##  ##  ##  ##  ##  ##  ##  ##  ##  ##  ##  ##  ##  ##  ##  ##  ##  ##  ##  ##  ##  ##  ##  ##  ##  ##  ##  ##  ##  ##  ##  ##  ##  ##  ##  ##  ##  ##  ##  ##  ##  ##  ##  ##  ## [2910_3]56	: ##  ##  ##  ##  ##  ##  ##  ##  ##  ##  ##  ##  ##  ##  ##  ##  ##  ##  ##  ##  ##  ##  ##  ##  ##  10  11  2  11  7  9  9  3  9  3  18  3  18  14  9  8  13  5  14  12  3  3  13  12  1  3  14  11  13  6  --  14  9  4  3  17  3  3  3  21  13  3  5  19  ##  ##  ##  ##  ##  ##  ##  ##  ##  ##  ##  ##  ##  ##  ##  ##  ##  ##  ##  ##  ##  ##  ##  ##  ##  ##  ##  ##  ##  ##  ##  ##  ##  ##  ##  ##  ##  ##  ##  ##  ##  ##  ##  ##  ##  ##  ##  ##  ##  ##  ##  ##  ##  ##  ##  ##  ##  ##  ##  ##  ##  ##  ##  ##  ##  ##  ##  ##  ##  ##  ##  ##  ##  ##  ##  ##  ##  ##  ##  ##  ##  ##  ##  ##  ##  ##  ##  ##  ##  ##  ##  ##  ##  ##  ##  ##  ##  ##  ##  ##  ##  ##  ##  ##  ##  ##  ##  ##  ##  ##  ##  ##  ##  ##  ##  ##  ##  ##  ##  ##  ##  ##  ##  ##  ##  ##  ##  ##  ##  ##  ##  ##  ##  ##  ##  ##  ##  ##  ##  ##  ##  ##  ##  ##  ##  ##  ##  ##  ##  ##  ##  ##  ##  ##  ##  ##  ##  ##  ##  ##  ##  ##  ##  ##  ##  ##  ##  ##  ##  ##  ##  ##  ##  ##  ##  ##  ##  ##  ##  ##  ##  ##  ##  ##  ##  ##  ##  ##  ##  ##  ##  ##  ##  ##  ##  ##  ##  ##  ##  ##  ## [2912_3]57	: ##  ##  ##  ##  ##  ##  ##  ##  ##  ##  ##  ##  ##  ##  ##  ##  ##  ##  ##  ##  ##  ##  ##  ##  ##  10  7  14  19  15  17  17  15  9  15  10  15  10  6  7  8  5  17  6  6  15  15  5  8  13  15  12  3  5  12  14  --  5  12  15  9  15  15  15  13  3  15  9  11  ##  ##  ##  ##  ##  ##  ##  ##  ##  ##  ##  ##  ##  ##  ##  ##  ##  ##  ##  ##  ##  ##  ##  ##  ##  ##  ##  ##  ##  ##  ##  ##  ##  ##  ##  ##  ##  ##  ##  ##  ##  ##  ##  ##  ##  ##  ##  ##  ##  ##  ##  ##  ##  ##  ##  ##  ##  ##  ##  ##  ##  ##  ##  ##  ##  ##  ##  ##  ##  ##  ##  ##  ##  ##  ##  ##  ##  ##  ##  ##  ##  ##  ##  ##  ##  ##  ##  ##  ##  ##  ##  ##  ##  ##  ##  ##  ##  ##  ##  ##  ##  ##  ##  ##  ##  ##  ##  ##  ##  ##  ##  ##  ##  ##  ##  ##  ##  ##  ##  ##  ##  ##  ##  ##  ##  ##  ##  ##  ##  ##  ##  ##  ##  ##  ##  ##  ##  ##  ##  ##  ##  ##  ##  ##  ##  ##  ##  ##  ##  ##  ##  ##  ##  ##  ##  ##  ##  ##  ##  ##  ##  ##  ##  ##  ##  ##  ##  ##  ##  ##  ##  ##  ##  ##  ##  ##  ##  ##  ##  ##  ##  ##  ##  ##  ##  ##  ##  ##  ##  ##  ##  ##  ##  ##  ##  ##  ##  ##  ##  ##  ## [2913_3]58	: ##  ##  ##  ##  ##  ##  ##  ##  ##  ##  ##  ##  ##  ##  ##  ##  ##  ##  ##  ##  ##  ##  ##  ##  ##  5  2  9  14  10  12  12  10  4  10  9  10  9  5  2  3  4  12  5  3  10  10  4  3  8  10  7  2  4  7  9  5  --  7  10  8  10  10  10  12  4  10  4  10  ##  ##  ##  ##  ##  ##  ##  ##  ##  ##  ##  ##  ##  ##  ##  ##  ##  ##  ##  ##  ##  ##  ##  ##  ##  ##  ##  ##  ##  ##  ##  ##  ##  ##  ##  ##  ##  ##  ##  ##  ##  ##  ##  ##  ##  ##  ##  ##  ##  ##  ##  ##  ##  ##  ##  ##  ##  ##  ##  ##  ##  ##  ##  ##  ##  ##  ##  ##  ##  ##  ##  ##  ##  ##  ##  ##  ##  ##  ##  ##  ##  ##  ##  ##  ##  ##  ##  ##  ##  ##  ##  ##  ##  ##  ##  ##  ##  ##  ##  ##  ##  ##  ##  ##  ##  ##  ##  ##  ##  ##  ##  ##  ##  ##  ##  ##  ##  ##  ##  ##  ##  ##  ##  ##  ##  ##  ##  ##  ##  ##  ##  ##  ##  ##  ##  ##  ##  ##  ##  ##  ##  ##  ##  ##  ##  ##  ##  ##  ##  ##  ##  ##  ##  ##  ##  ##  ##  ##  ##  ##  ##  ##  ##  ##  ##  ##  ##  ##  ##  ##  ##  ##  ##  ##  ##  ##  ##  ##  ##  ##  ##  ##  ##  ##  ##  ##  ##  ##  ##  ##  ##  ##  ##  ##  ##  ##  ##  ##  ##  ##  ## [2915_3]59	: ##  ##  ##  ##  ##  ##  ##  ##  ##  ##  ##  ##  ##  ##  ##  ##  ##  ##  ##  ##  ##  ##  ##  ##  ##  8  9  4  9  5  7  7  5  7  5  16  5  16  12  7  6  11  7  12  10  5  5  11  10  3  5  12  9  11  4  4  12  7  --  5  15  5  5  5  19  11  5  3  17  ##  ##  ##  ##  ##  ##  ##  ##  ##  ##  ##  ##  ##  ##  ##  ##  ##  ##  ##  ##  ##  ##  ##  ##  ##  ##  ##  ##  ##  ##  ##  ##  ##  ##  ##  ##  ##  ##  ##  ##  ##  ##  ##  ##  ##  ##  ##  ##  ##  ##  ##  ##  ##  ##  ##  ##  ##  ##  ##  ##  ##  ##  ##  ##  ##  ##  ##  ##  ##  ##  ##  ##  ##  ##  ##  ##  ##  ##  ##  ##  ##  ##  ##  ##  ##  ##  ##  ##  ##  ##  ##  ##  ##  ##  ##  ##  ##  ##  ##  ##  ##  ##  ##  ##  ##  ##  ##  ##  ##  ##  ##  ##  ##  ##  ##  ##  ##  ##  ##  ##  ##  ##  ##  ##  ##  ##  ##  ##  ##  ##  ##  ##  ##  ##  ##  ##  ##  ##  ##  ##  ##  ##  ##  ##  ##  ##  ##  ##  ##  ##  ##  ##  ##  ##  ##  ##  ##  ##  ##  ##  ##  ##  ##  ##  ##  ##  ##  ##  ##  ##  ##  ##  ##  ##  ##  ##  ##  ##  ##  ##  ##  ##  ##  ##  ##  ##  ##  ##  ##  ##  ##  ##  ##  ##  ##  ##  ##  ##  ##  ##  ## [2916_3]60	: ##  ##  ##  ##  ##  ##  ##  ##  ##  ##  ##  ##  ##  ##  ##  ##  ##  ##  ##  ##  ##  ##  ##  ##  ##  11  12  3  12  8  10  10  4  10  4  19  4  19  15  10  9  14  6  15  13  4  4  14  13  2  4  15  12  14  7  3  15  10  5  --  18  4  2  4  22  14  4  6  20  ##  ##  ##  ##  ##  ##  ##  ##  ##  ##  ##  ##  ##  ##  ##  ##  ##  ##  ##  ##  ##  ##  ##  ##  ##  ##  ##  ##  ##  ##  ##  ##  ##  ##  ##  ##  ##  ##  ##  ##  ##  ##  ##  ##  ##  ##  ##  ##  ##  ##  ##  ##  ##  ##  ##  ##  ##  ##  ##  ##  ##  ##  ##  ##  ##  ##  ##  ##  ##  ##  ##  ##  ##  ##  ##  ##  ##  ##  ##  ##  ##  ##  ##  ##  ##  ##  ##  ##  ##  ##  ##  ##  ##  ##  ##  ##  ##  ##  ##  ##  ##  ##  ##  ##  ##  ##  ##  ##  ##  ##  ##  ##  ##  ##  ##  ##  ##  ##  ##  ##  ##  ##  ##  ##  ##  ##  ##  ##  ##  ##  ##  ##  ##  ##  ##  ##  ##  ##  ##  ##  ##  ##  ##  ##  ##  ##  ##  ##  ##  ##  ##  ##  ##  ##  ##  ##  ##  ##  ##  ##  ##  ##  ##  ##  ##  ##  ##  ##  ##  ##  ##  ##  ##  ##  ##  ##  ##  ##  ##  ##  ##  ##  ##  ##  ##  ##  ##  ##  ##  ##  ##  ##  ##  ##  ##  ##  ##  ##  ##  ##  ## [2922_3]61	: ##  ##  ##  ##  ##  ##  ##  ##  ##  ##  ##  ##  ##  ##  ##  ##  ##  ##  ##  ##  ##  ##  ##  ##  ##  13  10  17  22  18  20  20  18  12  18  3  18  1  9  10  11  4  20  7  9  18  18  8  11  16  18  15  6  8  15  17  9  8  15  18  --  18  18  18  6  8  18  12  4  ##  ##  ##  ##  ##  ##  ##  ##  ##  ##  ##  ##  ##  ##  ##  ##  ##  ##  ##  ##  ##  ##  ##  ##  ##  ##  ##  ##  ##  ##  ##  ##  ##  ##  ##  ##  ##  ##  ##  ##  ##  ##  ##  ##  ##  ##  ##  ##  ##  ##  ##  ##  ##  ##  ##  ##  ##  ##  ##  ##  ##  ##  ##  ##  ##  ##  ##  ##  ##  ##  ##  ##  ##  ##  ##  ##  ##  ##  ##  ##  ##  ##  ##  ##  ##  ##  ##  ##  ##  ##  ##  ##  ##  ##  ##  ##  ##  ##  ##  ##  ##  ##  ##  ##  ##  ##  ##  ##  ##  ##  ##  ##  ##  ##  ##  ##  ##  ##  ##  ##  ##  ##  ##  ##  ##  ##  ##  ##  ##  ##  ##  ##  ##  ##  ##  ##  ##  ##  ##  ##  ##  ##  ##  ##  ##  ##  ##  ##  ##  ##  ##  ##  ##  ##  ##  ##  ##  ##  ##  ##  ##  ##  ##  ##  ##  ##  ##  ##  ##  ##  ##  ##  ##  ##  ##  ##  ##  ##  ##  ##  ##  ##  ##  ##  ##  ##  ##  ##  ##  ##  ##  ##  ##  ##  ##  ##  ##  ##  ##  ##  ## [2923_3]62	: ##  ##  ##  ##  ##  ##  ##  ##  ##  ##  ##  ##  ##  ##  ##  ##  ##  ##  ##  ##  ##  ##  ##  ##  ##  11  12  1  12  8  10  10  2  10  4  19  2  19  15  10  9  14  4  15  13  2  2  14  13  2  2  15  12  14  7  3  15  10  5  4  18  --  2  2  22  14  2  6  20  ##  ##  ##  ##  ##  ##  ##  ##  ##  ##  ##  ##  ##  ##  ##  ##  ##  ##  ##  ##  ##  ##  ##  ##  ##  ##  ##  ##  ##  ##  ##  ##  ##  ##  ##  ##  ##  ##  ##  ##  ##  ##  ##  ##  ##  ##  ##  ##  ##  ##  ##  ##  ##  ##  ##  ##  ##  ##  ##  ##  ##  ##  ##  ##  ##  ##  ##  ##  ##  ##  ##  ##  ##  ##  ##  ##  ##  ##  ##  ##  ##  ##  ##  ##  ##  ##  ##  ##  ##  ##  ##  ##  ##  ##  ##  ##  ##  ##  ##  ##  ##  ##  ##  ##  ##  ##  ##  ##  ##  ##  ##  ##  ##  ##  ##  ##  ##  ##  ##  ##  ##  ##  ##  ##  ##  ##  ##  ##  ##  ##  ##  ##  ##  ##  ##  ##  ##  ##  ##  ##  ##  ##  ##  ##  ##  ##  ##  ##  ##  ##  ##  ##  ##  ##  ##  ##  ##  ##  ##  ##  ##  ##  ##  ##  ##  ##  ##  ##  ##  ##  ##  ##  ##  ##  ##  ##  ##  ##  ##  ##  ##  ##  ##  ##  ##  ##  ##  ##  ##  ##  ##  ##  ##  ##  ##  ##  ##  ##  ##  ##  ## [2929_3]63	: ##  ##  ##  ##  ##  ##  ##  ##  ##  ##  ##  ##  ##  ##  ##  ##  ##  ##  ##  ##  ##  ##  ##  ##  ##  11  12  1  12  8  10  10  2  10  4  19  2  19  15  10  9  14  4  15  13  2  2  14  13  2  2  15  12  14  7  3  15  10  5  2  18  2  --  2  22  14  2  6  20  ##  ##  ##  ##  ##  ##  ##  ##  ##  ##  ##  ##  ##  ##  ##  ##  ##  ##  ##  ##  ##  ##  ##  ##  ##  ##  ##  ##  ##  ##  ##  ##  ##  ##  ##  ##  ##  ##  ##  ##  ##  ##  ##  ##  ##  ##  ##  ##  ##  ##  ##  ##  ##  ##  ##  ##  ##  ##  ##  ##  ##  ##  ##  ##  ##  ##  ##  ##  ##  ##  ##  ##  ##  ##  ##  ##  ##  ##  ##  ##  ##  ##  ##  ##  ##  ##  ##  ##  ##  ##  ##  ##  ##  ##  ##  ##  ##  ##  ##  ##  ##  ##  ##  ##  ##  ##  ##  ##  ##  ##  ##  ##  ##  ##  ##  ##  ##  ##  ##  ##  ##  ##  ##  ##  ##  ##  ##  ##  ##  ##  ##  ##  ##  ##  ##  ##  ##  ##  ##  ##  ##  ##  ##  ##  ##  ##  ##  ##  ##  ##  ##  ##  ##  ##  ##  ##  ##  ##  ##  ##  ##  ##  ##  ##  ##  ##  ##  ##  ##  ##  ##  ##  ##  ##  ##  ##  ##  ##  ##  ##  ##  ##  ##  ##  ##  ##  ##  ##  ##  ##  ##  ##  ##  ##  ##  ##  ##  ##  ##  ##  ## [2930_3]64	: ##  ##  ##  ##  ##  ##  ##  ##  ##  ##  ##  ##  ##  ##  ##  ##  ##  ##  ##  ##  ##  ##  ##  ##  ##  11  12  1  12  8  10  10  2  10  4  19  2  19  15  10  9  14  2  15  13  2  2  14  13  2  2  15  12  14  7  3  15  10  5  4  18  2  2  --  22  14  2  6  20  ##  ##  ##  ##  ##  ##  ##  ##  ##  ##  ##  ##  ##  ##  ##  ##  ##  ##  ##  ##  ##  ##  ##  ##  ##  ##  ##  ##  ##  ##  ##  ##  ##  ##  ##  ##  ##  ##  ##  ##  ##  ##  ##  ##  ##  ##  ##  ##  ##  ##  ##  ##  ##  ##  ##  ##  ##  ##  ##  ##  ##  ##  ##  ##  ##  ##  ##  ##  ##  ##  ##  ##  ##  ##  ##  ##  ##  ##  ##  ##  ##  ##  ##  ##  ##  ##  ##  ##  ##  ##  ##  ##  ##  ##  ##  ##  ##  ##  ##  ##  ##  ##  ##  ##  ##  ##  ##  ##  ##  ##  ##  ##  ##  ##  ##  ##  ##  ##  ##  ##  ##  ##  ##  ##  ##  ##  ##  ##  ##  ##  ##  ##  ##  ##  ##  ##  ##  ##  ##  ##  ##  ##  ##  ##  ##  ##  ##  ##  ##  ##  ##  ##  ##  ##  ##  ##  ##  ##  ##  ##  ##  ##  ##  ##  ##  ##  ##  ##  ##  ##  ##  ##  ##  ##  ##  ##  ##  ##  ##  ##  ##  ##  ##  ##  ##  ##  ##  ##  ##  ##  ##  ##  ##  ##  ##  ##  ##  ##  ##  ##  ## [2931_3]65	: ##  ##  ##  ##  ##  ##  ##  ##  ##  ##  ##  ##  ##  ##  ##  ##  ##  ##  ##  ##  ##  ##  ##  ##  ##  17  14  21  26  22  24  24  22  16  22  3  22  7  13  14  15  8  24  11  13  22  22  12  15  20  22  19  10  12  19  21  13  12  19  22  6  22  22  22  --  12  22  16  8  ##  ##  ##  ##  ##  ##  ##  ##  ##  ##  ##  ##  ##  ##  ##  ##  ##  ##  ##  ##  ##  ##  ##  ##  ##  ##  ##  ##  ##  ##  ##  ##  ##  ##  ##  ##  ##  ##  ##  ##  ##  ##  ##  ##  ##  ##  ##  ##  ##  ##  ##  ##  ##  ##  ##  ##  ##  ##  ##  ##  ##  ##  ##  ##  ##  ##  ##  ##  ##  ##  ##  ##  ##  ##  ##  ##  ##  ##  ##  ##  ##  ##  ##  ##  ##  ##  ##  ##  ##  ##  ##  ##  ##  ##  ##  ##  ##  ##  ##  ##  ##  ##  ##  ##  ##  ##  ##  ##  ##  ##  ##  ##  ##  ##  ##  ##  ##  ##  ##  ##  ##  ##  ##  ##  ##  ##  ##  ##  ##  ##  ##  ##  ##  ##  ##  ##  ##  ##  ##  ##  ##  ##  ##  ##  ##  ##  ##  ##  ##  ##  ##  ##  ##  ##  ##  ##  ##  ##  ##  ##  ##  ##  ##  ##  ##  ##  ##  ##  ##  ##  ##  ##  ##  ##  ##  ##  ##  ##  ##  ##  ##  ##  ##  ##  ##  ##  ##  ##  ##  ##  ##  ##  ##  ##  ##  ##  ##  ##  ##  ##  ## [2932_3]66	: ##  ##  ##  ##  ##  ##  ##  ##  ##  ##  ##  ##  ##  ##  ##  ##  ##  ##  ##  ##  ##  ##  ##  ##  ##  9  6  13  18  14  16  16  14  8  14  9  14  9  5  6  7  4  16  5  5  14  14  4  7  12  14  11  2  4  11  13  3  4  11  14  8  14  14  14  12  --  14  8  10  ##  ##  ##  ##  ##  ##  ##  ##  ##  ##  ##  ##  ##  ##  ##  ##  ##  ##  ##  ##  ##  ##  ##  ##  ##  ##  ##  ##  ##  ##  ##  ##  ##  ##  ##  ##  ##  ##  ##  ##  ##  ##  ##  ##  ##  ##  ##  ##  ##  ##  ##  ##  ##  ##  ##  ##  ##  ##  ##  ##  ##  ##  ##  ##  ##  ##  ##  ##  ##  ##  ##  ##  ##  ##  ##  ##  ##  ##  ##  ##  ##  ##  ##  ##  ##  ##  ##  ##  ##  ##  ##  ##  ##  ##  ##  ##  ##  ##  ##  ##  ##  ##  ##  ##  ##  ##  ##  ##  ##  ##  ##  ##  ##  ##  ##  ##  ##  ##  ##  ##  ##  ##  ##  ##  ##  ##  ##  ##  ##  ##  ##  ##  ##  ##  ##  ##  ##  ##  ##  ##  ##  ##  ##  ##  ##  ##  ##  ##  ##  ##  ##  ##  ##  ##  ##  ##  ##  ##  ##  ##  ##  ##  ##  ##  ##  ##  ##  ##  ##  ##  ##  ##  ##  ##  ##  ##  ##  ##  ##  ##  ##  ##  ##  ##  ##  ##  ##  ##  ##  ##  ##  ##  ##  ##  ##  ##  ##  ##  ##  ##  ## [2934_3]67	: ##  ##  ##  ##  ##  ##  ##  ##  ##  ##  ##  ##  ##  ##  ##  ##  ##  ##  ##  ##  ##  ##  ##  ##  ##  11  12  1  12  8  10  10  2  10  4  19  2  19  15  10  9  14  4  15  13  2  2  14  13  2  2  15  12  14  7  3  15  10  5  4  18  2  2  2  22  14  --  6  20  ##  ##  ##  ##  ##  ##  ##  ##  ##  ##  ##  ##  ##  ##  ##  ##  ##  ##  ##  ##  ##  ##  ##  ##  ##  ##  ##  ##  ##  ##  ##  ##  ##  ##  ##  ##  ##  ##  ##  ##  ##  ##  ##  ##  ##  ##  ##  ##  ##  ##  ##  ##  ##  ##  ##  ##  ##  ##  ##  ##  ##  ##  ##  ##  ##  ##  ##  ##  ##  ##  ##  ##  ##  ##  ##  ##  ##  ##  ##  ##  ##  ##  ##  ##  ##  ##  ##  ##  ##  ##  ##  ##  ##  ##  ##  ##  ##  ##  ##  ##  ##  ##  ##  ##  ##  ##  ##  ##  ##  ##  ##  ##  ##  ##  ##  ##  ##  ##  ##  ##  ##  ##  ##  ##  ##  ##  ##  ##  ##  ##  ##  ##  ##  ##  ##  ##  ##  ##  ##  ##  ##  ##  ##  ##  ##  ##  ##  ##  ##  ##  ##  ##  ##  ##  ##  ##  ##  ##  ##  ##  ##  ##  ##  ##  ##  ##  ##  ##  ##  ##  ##  ##  ##  ##  ##  ##  ##  ##  ##  ##  ##  ##  ##  ##  ##  ##  ##  ##  ##  ##  ##  ##  ##  ##  ##  ##  ##  ##  ##  ##  ## [2935_3]68	: ##  ##  ##  ##  ##  ##  ##  ##  ##  ##  ##  ##  ##  ##  ##  ##  ##  ##  ##  ##  ##  ##  ##  ##  ##  5  6  5  10  6  8  8  6  4  6  13  6  13  9  4  3  8  8  9  7  6  6  8  7  4  6  9  6  8  3  5  9  4  3  6  12  6  6  6  16  8  6  --  14  ##  ##  ##  ##  ##  ##  ##  ##  ##  ##  ##  ##  ##  ##  ##  ##  ##  ##  ##  ##  ##  ##  ##  ##  ##  ##  ##  ##  ##  ##  ##  ##  ##  ##  ##  ##  ##  ##  ##  ##  ##  ##  ##  ##  ##  ##  ##  ##  ##  ##  ##  ##  ##  ##  ##  ##  ##  ##  ##  ##  ##  ##  ##  ##  ##  ##  ##  ##  ##  ##  ##  ##  ##  ##  ##  ##  ##  ##  ##  ##  ##  ##  ##  ##  ##  ##  ##  ##  ##  ##  ##  ##  ##  ##  ##  ##  ##  ##  ##  ##  ##  ##  ##  ##  ##  ##  ##  ##  ##  ##  ##  ##  ##  ##  ##  ##  ##  ##  ##  ##  ##  ##  ##  ##  ##  ##  ##  ##  ##  ##  ##  ##  ##  ##  ##  ##  ##  ##  ##  ##  ##  ##  ##  ##  ##  ##  ##  ##  ##  ##  ##  ##  ##  ##  ##  ##  ##  ##  ##  ##  ##  ##  ##  ##  ##  ##  ##  ##  ##  ##  ##  ##  ##  ##  ##  ##  ##  ##  ##  ##  ##  ##  ##  ##  ##  ##  ##  ##  ##  ##  ##  ##  ##  ##  ##  ##  ##  ##  ##  ##  ## [2480_3]69	: ##  ##  ##  ##  ##  ##  ##  ##  ##  ##  ##  ##  ##  ##  ##  ##  ##  ##  ##  ##  ##  ##  ##  ##  ##  15  12  19  24  20  22  22  20  14  20  5  20  5  11  12  13  6  22  9  11  20  20  10  13  18  20  17  8  10  17  19  11  10  17  20  4  20  20  20  8  10  20  14  --  ##  ##  ##  ##  ##  ##  ##  ##  ##  ##  ##  ##  ##  ##  ##  ##  ##  ##  ##  ##  ##  ##  ##  ##  ##  ##  ##  ##  ##  ##  ##  ##  ##  ##  ##  ##  ##  ##  ##  ##  ##  ##  ##  ##  ##  ##  ##  ##  ##  ##  ##  ##  ##  ##  ##  ##  ##  ##  ##  ##  ##  ##  ##  ##  ##  ##  ##  ##  ##  ##  ##  ##  ##  ##  ##  ##  ##  ##  ##  ##  ##  ##  ##  ##  ##  ##  ##  ##  ##  ##  ##  ##  ##  ##  ##  ##  ##  ##  ##  ##  ##  ##  ##  ##  ##  ##  ##  ##  ##  ##  ##  ##  ##  ##  ##  ##  ##  ##  ##  ##  ##  ##  ##  ##  ##  ##  ##  ##  ##  ##  ##  ##  ##  ##  ##  ##  ##  ##  ##  ##  ##  ##  ##  ##  ##  ##  ##  ##  ##  ##  ##  ##  ##  ##  ##  ##  ##  ##  ##  ##  ##  ##  ##  ##  ##  ##  ##  ##  ##  ##  ##  ##  ##  ##  ##  ##  ##  ##  ##  ##  ##  ##  ##  ##  ##  ##  ##  ##  ##  ##  ##  ##  ##  ##  ##  ##  ##  ##  ##  ##  ## [825_1]70	: ##  ##  ##  ##  ##  ##  ##  ##  ##  ##  ##  ##  ##  ##  ##  ##  ##  ##  ##  ##  ##  ##  ##  ##  ##  ##  ##  ##  ##  ##  ##  ##  ##  ##  ##  ##  ##  ##  ##  ##  ##  ##  ##  ##  ##  ##  ##  ##  ##  ##  ##  ##  ##  ##  ##  ##  ##  ##  ##  ##  ##  ##  ##  ##  ##  ##  ##  ##  ##  --  1  1  1  9  1  1  1  1  2  1  2  4  ##  ##  ##  ##  ##  ##  ##  ##  ##  ##  ##  ##  ##  ##  ##  ##  ##  ##  ##  ##  ##  ##  ##  ##  ##  ##  ##  ##  ##  ##  ##  ##  ##  ##  ##  ##  ##  ##  ##  ##  ##  ##  ##  ##  ##  ##  ##  ##  ##  ##  ##  ##  ##  ##  ##  ##  ##  ##  ##  ##  ##  ##  ##  ##  ##  ##  ##  ##  ##  ##  ##  ##  ##  ##  ##  ##  ##  ##  ##  ##  ##  ##  ##  ##  ##  ##  ##  ##  ##  ##  ##  ##  ##  ##  ##  ##  ##  ##  ##  ##  ##  ##  ##  ##  ##  ##  ##  ##  ##  ##  ##  ##  ##  ##  ##  ##  ##  ##  ##  ##  ##  ##  ##  ##  ##  ##  ##  ##  ##  ##  ##  ##  ##  ##  ##  ##  ##  ##  ##  ##  ##  ##  ##  ##  ##  ##  ##  ##  ##  ##  ##  ##  ##  ##  ##  ##  ##  ##  ##  ##  ##  ##  ##  ##  ##  ##  ##  ##  ##  ##  ##  ##  ##  ##  ##  ##  ##  ##  ##  ##  ##  ##  ##  ##  ##  ##  ##  ## [826_1]71	: ##  ##  ##  ##  ##  ##  ##  ##  ##  ##  ##  ##  ##  ##  ##  ##  ##  ##  ##  ##  ##  ##  ##  ##  ##  ##  ##  ##  ##  ##  ##  ##  ##  ##  ##  ##  ##  ##  ##  ##  ##  ##  ##  ##  ##  ##  ##  ##  ##  ##  ##  ##  ##  ##  ##  ##  ##  ##  ##  ##  ##  ##  ##  ##  ##  ##  ##  ##  ##  1  --  2  2  10  2  2  2  2  3  2  2  5  ##  ##  ##  ##  ##  ##  ##  ##  ##  ##  ##  ##  ##  ##  ##  ##  ##  ##  ##  ##  ##  ##  ##  ##  ##  ##  ##  ##  ##  ##  ##  ##  ##  ##  ##  ##  ##  ##  ##  ##  ##  ##  ##  ##  ##  ##  ##  ##  ##  ##  ##  ##  ##  ##  ##  ##  ##  ##  ##  ##  ##  ##  ##  ##  ##  ##  ##  ##  ##  ##  ##  ##  ##  ##  ##  ##  ##  ##  ##  ##  ##  ##  ##  ##  ##  ##  ##  ##  ##  ##  ##  ##  ##  ##  ##  ##  ##  ##  ##  ##  ##  ##  ##  ##  ##  ##  ##  ##  ##  ##  ##  ##  ##  ##  ##  ##  ##  ##  ##  ##  ##  ##  ##  ##  ##  ##  ##  ##  ##  ##  ##  ##  ##  ##  ##  ##  ##  ##  ##  ##  ##  ##  ##  ##  ##  ##  ##  ##  ##  ##  ##  ##  ##  ##  ##  ##  ##  ##  ##  ##  ##  ##  ##  ##  ##  ##  ##  ##  ##  ##  ##  ##  ##  ##  ##  ##  ##  ##  ##  ##  ##  ##  ##  ##  ##  ##  ##  ## [833_1]72	: ##  ##  ##  ##  ##  ##  ##  ##  ##  ##  ##  ##  ##  ##  ##  ##  ##  ##  ##  ##  ##  ##  ##  ##  ##  ##  ##  ##  ##  ##  ##  ##  ##  ##  ##  ##  ##  ##  ##  ##  ##  ##  ##  ##  ##  ##  ##  ##  ##  ##  ##  ##  ##  ##  ##  ##  ##  ##  ##  ##  ##  ##  ##  ##  ##  ##  ##  ##  ##  1  2  --  2  8  2  2  2  2  3  2  3  5  ##  ##  ##  ##  ##  ##  ##  ##  ##  ##  ##  ##  ##  ##  ##  ##  ##  ##  ##  ##  ##  ##  ##  ##  ##  ##  ##  ##  ##  ##  ##  ##  ##  ##  ##  ##  ##  ##  ##  ##  ##  ##  ##  ##  ##  ##  ##  ##  ##  ##  ##  ##  ##  ##  ##  ##  ##  ##  ##  ##  ##  ##  ##  ##  ##  ##  ##  ##  ##  ##  ##  ##  ##  ##  ##  ##  ##  ##  ##  ##  ##  ##  ##  ##  ##  ##  ##  ##  ##  ##  ##  ##  ##  ##  ##  ##  ##  ##  ##  ##  ##  ##  ##  ##  ##  ##  ##  ##  ##  ##  ##  ##  ##  ##  ##  ##  ##  ##  ##  ##  ##  ##  ##  ##  ##  ##  ##  ##  ##  ##  ##  ##  ##  ##  ##  ##  ##  ##  ##  ##  ##  ##  ##  ##  ##  ##  ##  ##  ##  ##  ##  ##  ##  ##  ##  ##  ##  ##  ##  ##  ##  ##  ##  ##  ##  ##  ##  ##  ##  ##  ##  ##  ##  ##  ##  ##  ##  ##  ##  ##  ##  ##  ##  ##  ##  ##  ##  ## [858_1]73	: ##  ##  ##  ##  ##  ##  ##  ##  ##  ##  ##  ##  ##  ##  ##  ##  ##  ##  ##  ##  ##  ##  ##  ##  ##  ##  ##  ##  ##  ##  ##  ##  ##  ##  ##  ##  ##  ##  ##  ##  ##  ##  ##  ##  ##  ##  ##  ##  ##  ##  ##  ##  ##  ##  ##  ##  ##  ##  ##  ##  ##  ##  ##  ##  ##  ##  ##  ##  ##  1  2  2  --  10  2  2  2  2  3  2  3  5  ##  ##  ##  ##  ##  ##  ##  ##  ##  ##  ##  ##  ##  ##  ##  ##  ##  ##  ##  ##  ##  ##  ##  ##  ##  ##  ##  ##  ##  ##  ##  ##  ##  ##  ##  ##  ##  ##  ##  ##  ##  ##  ##  ##  ##  ##  ##  ##  ##  ##  ##  ##  ##  ##  ##  ##  ##  ##  ##  ##  ##  ##  ##  ##  ##  ##  ##  ##  ##  ##  ##  ##  ##  ##  ##  ##  ##  ##  ##  ##  ##  ##  ##  ##  ##  ##  ##  ##  ##  ##  ##  ##  ##  ##  ##  ##  ##  ##  ##  ##  ##  ##  ##  ##  ##  ##  ##  ##  ##  ##  ##  ##  ##  ##  ##  ##  ##  ##  ##  ##  ##  ##  ##  ##  ##  ##  ##  ##  ##  ##  ##  ##  ##  ##  ##  ##  ##  ##  ##  ##  ##  ##  ##  ##  ##  ##  ##  ##  ##  ##  ##  ##  ##  ##  ##  ##  ##  ##  ##  ##  ##  ##  ##  ##  ##  ##  ##  ##  ##  ##  ##  ##  ##  ##  ##  ##  ##  ##  ##  ##  ##  ##  ##  ##  ##  ##  ##  ## [1325_1]74	: ##  ##  ##  ##  ##  ##  ##  ##  ##  ##  ##  ##  ##  ##  ##  ##  ##  ##  ##  ##  ##  ##  ##  ##  ##  ##  ##  ##  ##  ##  ##  ##  ##  ##  ##  ##  ##  ##  ##  ##  ##  ##  ##  ##  ##  ##  ##  ##  ##  ##  ##  ##  ##  ##  ##  ##  ##  ##  ##  ##  ##  ##  ##  ##  ##  ##  ##  ##  ##  9  10  8  10  --  10  10  10  10  11  10  11  13  ##  ##  ##  ##  ##  ##  ##  ##  ##  ##  ##  ##  ##  ##  ##  ##  ##  ##  ##  ##  ##  ##  ##  ##  ##  ##  ##  ##  ##  ##  ##  ##  ##  ##  ##  ##  ##  ##  ##  ##  ##  ##  ##  ##  ##  ##  ##  ##  ##  ##  ##  ##  ##  ##  ##  ##  ##  ##  ##  ##  ##  ##  ##  ##  ##  ##  ##  ##  ##  ##  ##  ##  ##  ##  ##  ##  ##  ##  ##  ##  ##  ##  ##  ##  ##  ##  ##  ##  ##  ##  ##  ##  ##  ##  ##  ##  ##  ##  ##  ##  ##  ##  ##  ##  ##  ##  ##  ##  ##  ##  ##  ##  ##  ##  ##  ##  ##  ##  ##  ##  ##  ##  ##  ##  ##  ##  ##  ##  ##  ##  ##  ##  ##  ##  ##  ##  ##  ##  ##  ##  ##  ##  ##  ##  ##  ##  ##  ##  ##  ##  ##  ##  ##  ##  ##  ##  ##  ##  ##  ##  ##  ##  ##  ##  ##  ##  ##  ##  ##  ##  ##  ##  ##  ##  ##  ##  ##  ##  ##  ##  ##  ##  ##  ##  ##  ##  ##  ## [1327_1]75	: ##  ##  ##  ##  ##  ##  ##  ##  ##  ##  ##  ##  ##  ##  ##  ##  ##  ##  ##  ##  ##  ##  ##  ##  ##  ##  ##  ##  ##  ##  ##  ##  ##  ##  ##  ##  ##  ##  ##  ##  ##  ##  ##  ##  ##  ##  ##  ##  ##  ##  ##  ##  ##  ##  ##  ##  ##  ##  ##  ##  ##  ##  ##  ##  ##  ##  ##  ##  ##  1  2  2  2  10  --  2  2  2  3  2  2  5  ##  ##  ##  ##  ##  ##  ##  ##  ##  ##  ##  ##  ##  ##  ##  ##  ##  ##  ##  ##  ##  ##  ##  ##  ##  ##  ##  ##  ##  ##  ##  ##  ##  ##  ##  ##  ##  ##  ##  ##  ##  ##  ##  ##  ##  ##  ##  ##  ##  ##  ##  ##  ##  ##  ##  ##  ##  ##  ##  ##  ##  ##  ##  ##  ##  ##  ##  ##  ##  ##  ##  ##  ##  ##  ##  ##  ##  ##  ##  ##  ##  ##  ##  ##  ##  ##  ##  ##  ##  ##  ##  ##  ##  ##  ##  ##  ##  ##  ##  ##  ##  ##  ##  ##  ##  ##  ##  ##  ##  ##  ##  ##  ##  ##  ##  ##  ##  ##  ##  ##  ##  ##  ##  ##  ##  ##  ##  ##  ##  ##  ##  ##  ##  ##  ##  ##  ##  ##  ##  ##  ##  ##  ##  ##  ##  ##  ##  ##  ##  ##  ##  ##  ##  ##  ##  ##  ##  ##  ##  ##  ##  ##  ##  ##  ##  ##  ##  ##  ##  ##  ##  ##  ##  ##  ##  ##  ##  ##  ##  ##  ##  ##  ##  ##  ##  ##  ##  ## [1342_1]76	: ##  ##  ##  ##  ##  ##  ##  ##  ##  ##  ##  ##  ##  ##  ##  ##  ##  ##  ##  ##  ##  ##  ##  ##  ##  ##  ##  ##  ##  ##  ##  ##  ##  ##  ##  ##  ##  ##  ##  ##  ##  ##  ##  ##  ##  ##  ##  ##  ##  ##  ##  ##  ##  ##  ##  ##  ##  ##  ##  ##  ##  ##  ##  ##  ##  ##  ##  ##  ##  1  2  2  2  10  2  --  2  2  3  2  3  3  ##  ##  ##  ##  ##  ##  ##  ##  ##  ##  ##  ##  ##  ##  ##  ##  ##  ##  ##  ##  ##  ##  ##  ##  ##  ##  ##  ##  ##  ##  ##  ##  ##  ##  ##  ##  ##  ##  ##  ##  ##  ##  ##  ##  ##  ##  ##  ##  ##  ##  ##  ##  ##  ##  ##  ##  ##  ##  ##  ##  ##  ##  ##  ##  ##  ##  ##  ##  ##  ##  ##  ##  ##  ##  ##  ##  ##  ##  ##  ##  ##  ##  ##  ##  ##  ##  ##  ##  ##  ##  ##  ##  ##  ##  ##  ##  ##  ##  ##  ##  ##  ##  ##  ##  ##  ##  ##  ##  ##  ##  ##  ##  ##  ##  ##  ##  ##  ##  ##  ##  ##  ##  ##  ##  ##  ##  ##  ##  ##  ##  ##  ##  ##  ##  ##  ##  ##  ##  ##  ##  ##  ##  ##  ##  ##  ##  ##  ##  ##  ##  ##  ##  ##  ##  ##  ##  ##  ##  ##  ##  ##  ##  ##  ##  ##  ##  ##  ##  ##  ##  ##  ##  ##  ##  ##  ##  ##  ##  ##  ##  ##  ##  ##  ##  ##  ##  ##  ## [2440_1]77	: ##  ##  ##  ##  ##  ##  ##  ##  ##  ##  ##  ##  ##  ##  ##  ##  ##  ##  ##  ##  ##  ##  ##  ##  ##  ##  ##  ##  ##  ##  ##  ##  ##  ##  ##  ##  ##  ##  ##  ##  ##  ##  ##  ##  ##  ##  ##  ##  ##  ##  ##  ##  ##  ##  ##  ##  ##  ##  ##  ##  ##  ##  ##  ##  ##  ##  ##  ##  ##  1  2  2  2  10  2  2  --  2  3  2  3  5  ##  ##  ##  ##  ##  ##  ##  ##  ##  ##  ##  ##  ##  ##  ##  ##  ##  ##  ##  ##  ##  ##  ##  ##  ##  ##  ##  ##  ##  ##  ##  ##  ##  ##  ##  ##  ##  ##  ##  ##  ##  ##  ##  ##  ##  ##  ##  ##  ##  ##  ##  ##  ##  ##  ##  ##  ##  ##  ##  ##  ##  ##  ##  ##  ##  ##  ##  ##  ##  ##  ##  ##  ##  ##  ##  ##  ##  ##  ##  ##  ##  ##  ##  ##  ##  ##  ##  ##  ##  ##  ##  ##  ##  ##  ##  ##  ##  ##  ##  ##  ##  ##  ##  ##  ##  ##  ##  ##  ##  ##  ##  ##  ##  ##  ##  ##  ##  ##  ##  ##  ##  ##  ##  ##  ##  ##  ##  ##  ##  ##  ##  ##  ##  ##  ##  ##  ##  ##  ##  ##  ##  ##  ##  ##  ##  ##  ##  ##  ##  ##  ##  ##  ##  ##  ##  ##  ##  ##  ##  ##  ##  ##  ##  ##  ##  ##  ##  ##  ##  ##  ##  ##  ##  ##  ##  ##  ##  ##  ##  ##  ##  ##  ##  ##  ##  ##  ##  ## [2445_1]78	: ##  ##  ##  ##  ##  ##  ##  ##  ##  ##  ##  ##  ##  ##  ##  ##  ##  ##  ##  ##  ##  ##  ##  ##  ##  ##  ##  ##  ##  ##  ##  ##  ##  ##  ##  ##  ##  ##  ##  ##  ##  ##  ##  ##  ##  ##  ##  ##  ##  ##  ##  ##  ##  ##  ##  ##  ##  ##  ##  ##  ##  ##  ##  ##  ##  ##  ##  ##  ##  1  2  2  2  10  2  2  2  --  3  2  3  5  ##  ##  ##  ##  ##  ##  ##  ##  ##  ##  ##  ##  ##  ##  ##  ##  ##  ##  ##  ##  ##  ##  ##  ##  ##  ##  ##  ##  ##  ##  ##  ##  ##  ##  ##  ##  ##  ##  ##  ##  ##  ##  ##  ##  ##  ##  ##  ##  ##  ##  ##  ##  ##  ##  ##  ##  ##  ##  ##  ##  ##  ##  ##  ##  ##  ##  ##  ##  ##  ##  ##  ##  ##  ##  ##  ##  ##  ##  ##  ##  ##  ##  ##  ##  ##  ##  ##  ##  ##  ##  ##  ##  ##  ##  ##  ##  ##  ##  ##  ##  ##  ##  ##  ##  ##  ##  ##  ##  ##  ##  ##  ##  ##  ##  ##  ##  ##  ##  ##  ##  ##  ##  ##  ##  ##  ##  ##  ##  ##  ##  ##  ##  ##  ##  ##  ##  ##  ##  ##  ##  ##  ##  ##  ##  ##  ##  ##  ##  ##  ##  ##  ##  ##  ##  ##  ##  ##  ##  ##  ##  ##  ##  ##  ##  ##  ##  ##  ##  ##  ##  ##  ##  ##  ##  ##  ##  ##  ##  ##  ##  ##  ##  ##  ##  ##  ##  ##  ## [2453_1]79	: ##  ##  ##  ##  ##  ##  ##  ##  ##  ##  ##  ##  ##  ##  ##  ##  ##  ##  ##  ##  ##  ##  ##  ##  ##  ##  ##  ##  ##  ##  ##  ##  ##  ##  ##  ##  ##  ##  ##  ##  ##  ##  ##  ##  ##  ##  ##  ##  ##  ##  ##  ##  ##  ##  ##  ##  ##  ##  ##  ##  ##  ##  ##  ##  ##  ##  ##  ##  ##  2  3  3  3  11  3  3  3  3  --  1  4  6  ##  ##  ##  ##  ##  ##  ##  ##  ##  ##  ##  ##  ##  ##  ##  ##  ##  ##  ##  ##  ##  ##  ##  ##  ##  ##  ##  ##  ##  ##  ##  ##  ##  ##  ##  ##  ##  ##  ##  ##  ##  ##  ##  ##  ##  ##  ##  ##  ##  ##  ##  ##  ##  ##  ##  ##  ##  ##  ##  ##  ##  ##  ##  ##  ##  ##  ##  ##  ##  ##  ##  ##  ##  ##  ##  ##  ##  ##  ##  ##  ##  ##  ##  ##  ##  ##  ##  ##  ##  ##  ##  ##  ##  ##  ##  ##  ##  ##  ##  ##  ##  ##  ##  ##  ##  ##  ##  ##  ##  ##  ##  ##  ##  ##  ##  ##  ##  ##  ##  ##  ##  ##  ##  ##  ##  ##  ##  ##  ##  ##  ##  ##  ##  ##  ##  ##  ##  ##  ##  ##  ##  ##  ##  ##  ##  ##  ##  ##  ##  ##  ##  ##  ##  ##  ##  ##  ##  ##  ##  ##  ##  ##  ##  ##  ##  ##  ##  ##  ##  ##  ##  ##  ##  ##  ##  ##  ##  ##  ##  ##  ##  ##  ##  ##  ##  ##  ##  ## [2796_1]80	: ##  ##  ##  ##  ##  ##  ##  ##  ##  ##  ##  ##  ##  ##  ##  ##  ##  ##  ##  ##  ##  ##  ##  ##  ##  ##  ##  ##  ##  ##  ##  ##  ##  ##  ##  ##  ##  ##  ##  ##  ##  ##  ##  ##  ##  ##  ##  ##  ##  ##  ##  ##  ##  ##  ##  ##  ##  ##  ##  ##  ##  ##  ##  ##  ##  ##  ##  ##  ##  1  2  2  2  10  2  2  2  2  1  --  3  5  ##  ##  ##  ##  ##  ##  ##  ##  ##  ##  ##  ##  ##  ##  ##  ##  ##  ##  ##  ##  ##  ##  ##  ##  ##  ##  ##  ##  ##  ##  ##  ##  ##  ##  ##  ##  ##  ##  ##  ##  ##  ##  ##  ##  ##  ##  ##  ##  ##  ##  ##  ##  ##  ##  ##  ##  ##  ##  ##  ##  ##  ##  ##  ##  ##  ##  ##  ##  ##  ##  ##  ##  ##  ##  ##  ##  ##  ##  ##  ##  ##  ##  ##  ##  ##  ##  ##  ##  ##  ##  ##  ##  ##  ##  ##  ##  ##  ##  ##  ##  ##  ##  ##  ##  ##  ##  ##  ##  ##  ##  ##  ##  ##  ##  ##  ##  ##  ##  ##  ##  ##  ##  ##  ##  ##  ##  ##  ##  ##  ##  ##  ##  ##  ##  ##  ##  ##  ##  ##  ##  ##  ##  ##  ##  ##  ##  ##  ##  ##  ##  ##  ##  ##  ##  ##  ##  ##  ##  ##  ##  ##  ##  ##  ##  ##  ##  ##  ##  ##  ##  ##  ##  ##  ##  ##  ##  ##  ##  ##  ##  ##  ##  ##  ##  ##  ##  ##  ## [T05_1]81	: ##  ##  ##  ##  ##  ##  ##  ##  ##  ##  ##  ##  ##  ##  ##  ##  ##  ##  ##  ##  ##  ##  ##  ##  ##  ##  ##  ##  ##  ##  ##  ##  ##  ##  ##  ##  ##  ##  ##  ##  ##  ##  ##  ##  ##  ##  ##  ##  ##  ##  ##  ##  ##  ##  ##  ##  ##  ##  ##  ##  ##  ##  ##  ##  ##  ##  ##  ##  ##  2  2  3  3  11  2  3  3  3  4  3  --  6  ##  ##  ##  ##  ##  ##  ##  ##  ##  ##  ##  ##  ##  ##  ##  ##  ##  ##  ##  ##  ##  ##  ##  ##  ##  ##  ##  ##  ##  ##  ##  ##  ##  ##  ##  ##  ##  ##  ##  ##  ##  ##  ##  ##  ##  ##  ##  ##  ##  ##  ##  ##  ##  ##  ##  ##  ##  ##  ##  ##  ##  ##  ##  ##  ##  ##  ##  ##  ##  ##  ##  ##  ##  ##  ##  ##  ##  ##  ##  ##  ##  ##  ##  ##  ##  ##  ##  ##  ##  ##  ##  ##  ##  ##  ##  ##  ##  ##  ##  ##  ##  ##  ##  ##  ##  ##  ##  ##  ##  ##  ##  ##  ##  ##  ##  ##  ##  ##  ##  ##  ##  ##  ##  ##  ##  ##  ##  ##  ##  ##  ##  ##  ##  ##  ##  ##  ##  ##  ##  ##  ##  ##  ##  ##  ##  ##  ##  ##  ##  ##  ##  ##  ##  ##  ##  ##  ##  ##  ##  ##  ##  ##  ##  ##  ##  ##  ##  ##  ##  ##  ##  ##  ##  ##  ##  ##  ##  ##  ##  ##  ##  ##  ##  ##  ##  ##  ##  ## [T06_1]82	: ##  ##  ##  ##  ##  ##  ##  ##  ##  ##  ##  ##  ##  ##  ##  ##  ##  ##  ##  ##  ##  ##  ##  ##  ##  ##  ##  ##  ##  ##  ##  ##  ##  ##  ##  ##  ##  ##  ##  ##  ##  ##  ##  ##  ##  ##  ##  ##  ##  ##  ##  ##  ##  ##  ##  ##  ##  ##  ##  ##  ##  ##  ##  ##  ##  ##  ##  ##  ##  4  5  5  5  13  5  3  5  5  6  5  6  --  ##  ##  ##  ##  ##  ##  ##  ##  ##  ##  ##  ##  ##  ##  ##  ##  ##  ##  ##  ##  ##  ##  ##  ##  ##  ##  ##  ##  ##  ##  ##  ##  ##  ##  ##  ##  ##  ##  ##  ##  ##  ##  ##  ##  ##  ##  ##  ##  ##  ##  ##  ##  ##  ##  ##  ##  ##  ##  ##  ##  ##  ##  ##  ##  ##  ##  ##  ##  ##  ##  ##  ##  ##  ##  ##  ##  ##  ##  ##  ##  ##  ##  ##  ##  ##  ##  ##  ##  ##  ##  ##  ##  ##  ##  ##  ##  ##  ##  ##  ##  ##  ##  ##  ##  ##  ##  ##  ##  ##  ##  ##  ##  ##  ##  ##  ##  ##  ##  ##  ##  ##  ##  ##  ##  ##  ##  ##  ##  ##  ##  ##  ##  ##  ##  ##  ##  ##  ##  ##  ##  ##  ##  ##  ##  ##  ##  ##  ##  ##  ##  ##  ##  ##  ##  ##  ##  ##  ##  ##  ##  ##  ##  ##  ##  ##  ##  ##  ##  ##  ##  ##  ##  ##  ##  ##  ##  ##  ##  ##  ##  ##  ##  ##  ##
[truncated: 503,029 more chars]
