# Supplementary material for: A mega-cryptic species complex hidden among one of the most common annelids in the North East Atlantic
Source: PLoS One. 2018 Jun 20;13(6):e0198356. doi: 10.1371/journal.pone.0198356 (PMC6010226; doi:10.1371/journal.pone.0198356)
Supplement: S21 Appendix — Log-file from the TCS-analysis on ITS2x-all. (RTF) [file pone.0198356.s021.rtf]

TCS v1.21Thu Nov 02 09:55:00 CET 2017Datafile = /Users/arnenygren/Desktop/namnlös mapp 3/its_xinsi_402.nexCurrent OS = macintoshData in NEXUS formatNumber of sequences: 402Length of sequences: 615_________________________________________________________PARSIMONY PROBABILITYFor 1 step(s),	P(95%) = 0.9994134002854367For 2 step(s),	P(95%) = 0.9977402008215183For 3 step(s),	P(95%) = 0.995306273323496For 4 step(s),	P(95%) = 0.9920684431098743For 5 step(s),	P(95%) = 0.9880292503953421For 6 step(s),	P(95%) = 0.9831966286583387For 7 step(s),	P(95%) = 0.9775800291181853For 8 step(s),	P(95%) = 0.9711907594818975For 9 step(s),	P(95%) = 0.9640420091898244For 10 step(s),	P(95%) = 0.9561488016359576For 11 step(s),	P(95%) = 0.9475279415450858RUN SETTINGSCalculated maximum connection steps at 95% = 10Gaps treated as missing dataWARNING: POSSIBLE MISSING DATA AMBIGUITIES1945_1 and 2220_1 differ only by missing or ambiguous characters2794_1 and 1949_1 differ only by missing or ambiguous characters2219_1 and 1954_1 differ only by missing or ambiguous characters2440_1 and 828_1 differ only by missing or ambiguous characters1311_2 and 2181_2 differ only by missing or ambiguous characters1311_2 and 2271_2 differ only by missing or ambiguous characters1311_2 and 2328_2 differ only by missing or ambiguous characters1311_2 and 2331_2 differ only by missing or ambiguous characters1311_2 and 2338_2 differ only by missing or ambiguous characters1311_2 and 2381_2 differ only by missing or ambiguous characters1311_2 and 844_2 differ only by missing or ambiguous characters2180_2 and 2216_2 differ only by missing or ambiguous characters2180_2 and 2272_2 differ only by missing or ambiguous characters2180_2 and 2322_2 differ only by missing or ambiguous characters2180_2 and 2332_2 differ only by missing or ambiguous characters2180_2 and 2352_2 differ only by missing or ambiguous characters2180_2 and 2354_2 differ only by missing or ambiguous characters2180_2 and 2367_2 differ only by missing or ambiguous characters2180_2 and 2368_2 differ only by missing or ambiguous characters2180_2 and 2369_2 differ only by missing or ambiguous characters2180_2 and 2336_2 differ only by missing or ambiguous characters2180_2 and 2376_2 differ only by missing or ambiguous characters1311_2 and 2387_2 differ only by missing or ambiguous characters2180_2 and 2185_2 differ only by missing or ambiguous characters2333_2 and 2370_2 differ only by missing or ambiguous characters1207_3 and 1310_3 differ only by missing or ambiguous characters1207_3 and 2380_3 differ only by missing or ambiguous characters1207_3 and 2812_3 differ only by missing or ambiguous characters1207_3 and 2872_3 differ only by missing or ambiguous characters1207_3 and 2873_3 differ only by missing or ambiguous characters1207_3 and 2874_3 differ only by missing or ambiguous characters1207_3 and 2876_3 differ only by missing or ambiguous characters1207_3 and 2877_3 differ only by missing or ambiguous characters1207_3 and 2879_3 differ only by missing or ambiguous characters1207_3 and 2880_3 differ only by missing or ambiguous characters1207_3 and 2881_3 differ only by missing or ambiguous characters1207_3 and 2882_3 differ only by missing or ambiguous characters1207_3 and 2885_3 differ only by missing or ambiguous characters1207_3 and 2886_3 differ only by missing or ambiguous characters1207_3 and 2887_3 differ only by missing or ambiguous characters1207_3 and 2888_3 differ only by missing or ambiguous characters1207_3 and 2889_3 differ only by missing or ambiguous characters1207_3 and 2890_3 differ only by missing or ambiguous characters1207_3 and 2891_3 differ only by missing or ambiguous characters1207_3 and 2901_3 differ only by missing or ambiguous characters1207_3 and 2906_3 differ only by missing or ambiguous characters1207_3 and 2907_3 differ only by missing or ambiguous characters1207_3 and 2908_3 differ only by missing or ambiguous characters1207_3 and 2910_3 differ only by missing or ambiguous characters1207_3 and 2912_3 differ only by missing or ambiguous characters1207_3 and 2915_3 differ only by missing or ambiguous characters1207_3 and 2916_3 differ only by missing or ambiguous characters1207_3 and 2917_3 differ only by missing or ambiguous characters1207_3 and 2922_3 differ only by missing or ambiguous characters1207_3 and 2923_3 differ only by missing or ambiguous characters1207_3 and 2930_3 differ only by missing or ambiguous characters1207_3 and 2931_3 differ only by missing or ambiguous characters1207_3 and 2932_3 differ only by missing or ambiguous characters1207_3 and 2933_3 differ only by missing or ambiguous characters1207_3 and 2934_3 differ only by missing or ambiguous characters1207_3 and 2935_3 differ only by missing or ambiguous characters1207_3 and 2902_3 differ only by missing or ambiguous characters1207_3 and 2924_3 differ only by missing or ambiguous characters1207_3 and 2784_3 differ only by missing or ambiguous characters2275_3 and 2285_3 differ only by missing or ambiguous characters2275_3 and 2464_3 differ only by missing or ambiguous characters2275_3 and 2301_3 differ only by missing or ambiguous characters2287_3 and 2913_3 differ only by missing or ambiguous characters2287_3 and 2480_3 differ only by missing or ambiguous characters2287_3 and 2291_3 differ only by missing or ambiguous characters2287_3 and 2781_3 differ only by missing or ambiguous characters2463_3 and 2465_3 differ only by missing or ambiguous characters2226_4 and 2045_4 differ only by missing or ambiguous characters2226_4 and 2227_4 differ only by missing or ambiguous characters2226_4 and 2228_4 differ only by missing or ambiguous characters2226_4 and 2229_4 differ only by missing or ambiguous characters2226_4 and 2230_4 differ only by missing or ambiguous characters2226_4 and 2231_4 differ only by missing or ambiguous characters2226_4 and 2232_4 differ only by missing or ambiguous characters2226_4 and 2233_4 differ only by missing or ambiguous characters2226_4 and 2234_4 differ only by missing or ambiguous characters2226_4 and 2235_4 differ only by missing or ambiguous characters2226_4 and 2236_4 differ only by missing or ambiguous characters2226_4 and TB28_4 differ only by missing or ambiguous characters840_5 and 2777_5 differ only by missing or ambiguous characters840_5 and 2779_5 differ only by missing or ambiguous characters840_5 and 2790_5 differ only by missing or ambiguous characters840_5 and 2792_5 differ only by missing or ambiguous characters840_5 and 2904_5 differ only by missing or ambiguous characters840_5 and 2918_5 differ only by missing or ambiguous characters840_5 and 2927_5 differ only by missing or ambiguous characters840_5 and 2928_5 differ only by missing or ambiguous characters840_5 and T02_5 differ only by missing or ambiguous characters2778_5 and 2780_5 differ only by missing or ambiguous characters2778_5 and 2791_5 differ only by missing or ambiguous characters2778_5 and 2793_5 differ only by missing or ambiguous characters2778_5 and 2926_5 differ only by missing or ambiguous characters2778_5 and T04_5 differ only by missing or ambiguous characters1874_6 and 838_6 differ only by missing or ambiguous characters846_6 and 847_6 differ only by missing or ambiguous characters846_6 and 849_6 differ only by missing or ambiguous characters846_6 and 1313_6 differ only by missing or ambiguous characters846_6 and 1321_6 differ only by missing or ambiguous characters846_6 and 1872_6 differ only by missing or ambiguous characters846_6 and 1320_6 differ only by missing or ambiguous characters1943_6 and 1316_6 differ only by missing or ambiguous characters1943_6 and 1319_6 differ only by missing or ambiguous characters1318_6 and 1875_6 differ only by missing or ambiguous characters1869_6 and 1942_6 differ only by missing or ambiguous characters1318_6 and 1873_6 differ only by missing or ambiguous characters1318_6 and 1870_6 differ only by missing or ambiguous characters1309_7 and 2859_7 differ only by missing or ambiguous characters1309_7 and 2443_7 differ only by missing or ambiguous characters1202_8 and 1958_8 differ only by missing or ambiguous characters1202_8 and 2013_8 differ only by missing or ambiguous characters1202_8 and 2039_8 differ only by missing or ambiguous characters1202_8 and 2478_8 differ only by missing or ambiguous characters1202_8 and 2798_8 differ only by missing or ambiguous characters1202_8 and 2178_8 differ only by missing or ambiguous characters1202_8 and 1197_8 differ only by missing or ambiguous characters1202_8 and 1561_8 differ only by missing or ambiguous characters1202_8 and 2014_8 differ only by missing or ambiguous characters1202_8 and 2015_8 differ only by missing or ambiguous characters1203_8 and 2799_8 differ only by missing or ambiguous characters1946_8 and 2925_8 differ only by missing or ambiguous characters1198_8 and 1200_8 differ only by missing or ambiguous characters1198_8 and 2036_8 differ only by missing or ambiguous characters1198_8 and 2037_8 differ only by missing or ambiguous characters1198_8 and 2214_8 differ only by missing or ambiguous characters1198_8 and 2457_8 differ only by missing or ambiguous characters1198_8 and 2775_8 differ only by missing or ambiguous characters1198_8 and 1989_8 differ only by missing or ambiguous characters1198_8 and 2896_8 differ only by missing or ambiguous characters1198_8 and 1199_8 differ only by missing or ambiguous characters1198_8 and 1988_8 differ only by missing or ambiguous characters862_9 and 861_9 differ only by missing or ambiguous characters2031_10 and 2034_10 differ only by missing or ambiguous characters2031_10 and 2024_10 differ only by missing or ambiguous characters2031_10 and 2029_10 differ only by missing or ambiguous characters2031_10 and 2033_10 differ only by missing or ambiguous characters2031_10 and TB25_10 differ only by missing or ambiguous characters2786_11 and 1560_11 differ only by missing or ambiguous characters2196_12 and 2200_12 differ only by missing or ambiguous characters2196_12 and 2222_12 differ only by missing or ambiguous characters2806_12 and 2826_12 differ only by missing or ambiguous characters2194_12 and 2818_12 differ only by missing or ambiguous characters2806_12 and 2223_12 differ only by missing or ambiguous characters2196_12 and 2199_12 differ only by missing or ambiguous characters2194_12 and 2829_12 differ only by missing or ambiguous characters1923_13 and 1959_13 differ only by missing or ambiguous characters1923_13 and 1960_13 differ only by missing or ambiguous characters1923_13 and 2184_13 differ only by missing or ambiguous characters1923_13 and 2776_13 differ only by missing or ambiguous characters1923_13 and 2027_13 differ only by missing or ambiguous characters1923_13 and 2175_13 differ only by missing or ambiguous characters1923_13 and 2215_13 differ only by missing or ambiguous characters1923_13 and 1986_13 differ only by missing or ambiguous characters1923_13 and 2183_13 differ only by missing or ambiguous characters1923_13 and 2317_13 differ only by missing or ambiguous characters1923_13 and 2337_13 differ only by missing or ambiguous characters1923_13 and 2458_13 differ only by missing or ambiguous characters1923_13 and 2813_13 differ only by missing or ambiguous characters1923_13 and 2921_13 differ only by missing or ambiguous characters1923_13 and 2038_13 differ only by missing or ambiguous characters1923_13 and T01_13 differ only by missing or ambiguous characters1923_13 and T03_13 differ only by missing or ambiguous characters1923_13 and 1201_13 differ only by missing or ambiguous characters1923_13 and 2035_13 differ only by missing or ambiguous characters1923_13 and 2475_13 differ only by missing or ambiguous characters1923_13 and 1205_13 differ only by missing or ambiguous characters1923_13 and 1999_13 differ only by missing or ambiguous characters2044_14 and 2842_14 differ only by missing or ambiguous characters2044_14 and 2845_14 differ only by missing or ambiguous characters2044_14 and 2846_14 differ only by missing or ambiguous characters2044_14 and 2852_14 differ only by missing or ambiguous characters2044_14 and 2855_14 differ only by missing or ambiguous characters2044_14 and 2479_14 differ only by missing or ambiguous characters2044_14 and 2477_14 differ only by missing or ambiguous characters2042_14 and 2843_14 differ only by missing or ambiguous characters2042_14 and 2844_14 differ only by missing or ambiguous characters2850_14 and 2851_14 differ only by missing or ambiguous characters2850_14 and 2854_14 differ only by missing or ambiguous characters2850_14 and 2847_14 differ only by missing or ambiguous characters2042_14 and 2811_14 differ only by missing or ambiguous characters2004_15 and 2003_15 differ only by missing or ambiguous characters2004_15 and 2005_15 differ only by missing or ambiguous characters2004_15 and 2008_15 differ only by missing or ambiguous characters2004_15 and 2009_15 differ only by missing or ambiguous characters2004_15 and 2010_15 differ only by missing or ambiguous characters2004_15 and 2011_15 differ only by missing or ambiguous characters2004_15 and 2012_15 differ only by missing or ambiguous characters2004_15 and 2017_15 differ only by missing or ambiguous characters2004_15 and 2018_15 differ only by missing or ambiguous characters2004_15 and 2019_15 differ only by missing or ambiguous characters2004_15 and 2020_15 differ only by missing or ambiguous characters2004_15 and 2025_15 differ only by missing or ambiguous characters2004_15 and 2030_15 differ only by missing or ambiguous characters2004_15 and 2043_15 differ only by missing or ambiguous characters2004_15 and 2849_15 differ only by missing or ambiguous characters2267_16 and 2838_16 differ only by missing or ambiguous characters2267_16 and 2289_16 differ only by missing or ambiguous characters2267_16 and 2290_16 differ only by missing or ambiguous characters2267_16 and 2296_16 differ only by missing or ambiguous characters2267_16 and 2297_16 differ only by missing or ambiguous characters2267_16 and 2298_16 differ only by missing or ambiguous characters2267_16 and 2299_16 differ only by missing or ambiguous characters2267_16 and 2300_16 differ only by missing or ambiguous characters2267_16 and 2305_16 differ only by missing or ambiguous characters2267_16 and 2306_16 differ only by missing or ambiguous characters2267_16 and 2307_16 differ only by missing or ambiguous characters2267_16 and 2309_16 differ only by missing or ambiguous characters2267_16 and 2310_16 differ only by missing or ambiguous characters2267_16 and 2316_16 differ only by missing or ambiguous characters2267_16 and 2325_16 differ only by missing or ambiguous characters2267_16 and 2330_16 differ only by missing or ambiguous characters2267_16 and 2339_16 differ only by missing or ambiguous characters2267_16 and 2341_16 differ only by missing or ambiguous characters2267_16 and 2355_16 differ only by missing or ambiguous characters2267_16 and 2356_16 differ only by missing or ambiguous characters2267_16 and 2357_16 differ only by missing or ambiguous characters2267_16 and 2358_16 differ only by missing or ambiguous characters2267_16 and 2359_16 differ only by missing or ambiguous characters2267_16 and 2361_16 differ only by missing or ambiguous characters2267_16 and 2362_16 differ only by missing or ambiguous characters2267_16 and 2363_16 differ only by missing or ambiguous characters2267_16 and 2364_16 differ only by missing or ambiguous characters2267_16 and 2366_16 differ only by missing or ambiguous characters2267_16 and 2373_16 differ only by missing or ambiguous characters2267_16 and 2374_16 differ only by missing or ambiguous characters2267_16 and 2379_16 differ only by missing or ambiguous characters2267_16 and 2389_16 differ only by missing or ambiguous characters2267_16 and 2391_16 differ only by missing or ambiguous characters2267_16 and 2462_16 differ only by missing or ambiguous characters2268_16 and 2270_16 differ only by missing or ambiguous characters2268_16 and 2292_16 differ only by missing or ambiguous characters2268_16 and 2294_16 differ only by missing or ambiguous characters2268_16 and 2295_16 differ only by missing or ambiguous characters2268_16 and 2466_16 differ only by missing or ambiguous characters2268_16 and 2467_16 differ only by missing or ambiguous characters2268_16 and 2470_16 differ only by missing or ambiguous characters2268_16 and 2471_16 differ only by missing or ambiguous characters2268_16 and 2472_16 differ only by missing or ambiguous characters2268_16 and 2473_16 differ only by missing or ambiguous characters2268_16 and 2474_16 differ only by missing or ambiguous characters2267_16 and 2303_16 differ only by missing or ambiguous characters2313_18 and 2314_18 differ only by missing or ambiguous characters2302_20 and 2903_20 differ only by missing or ambiguous characters2302_20 and 2875_20 differ only by missing or ambiguous characters2302_20 and 2349_20 differ only by missing or ambiguous characters2302_20 and 2348_20 differ only by missing or ambiguous characters2302_20 and 2329_20 differ only by missing or ambiguous characters2302_20 and 2324_20 differ only by missing or ambiguous characters2342_21 and 2894_21 differ only by missing or ambiguous characters2866_24 and 2868_24 differ only by missing or ambiguous characters2866_24 and 2867_24 differ only by missing or ambiguous characters2801_25 and 2809_25 differ only by missing or ambiguous characters2801_25 and 2802_25 differ only by missing or ambiguous characters2805_26 and 2808_26 differ only by missing or ambiguous characters2805_26 and 2807_26 differ only by missing or ambiguous charactersHAPLOTYPESNumber of haplotypes = 103Haplotype list: - 2440_1 :  2841_1 828_1 - 1945_1 :  1947_1 1951_1 1952_1 2241_1 TB29_1 2451_1 2797_1 1322_1 1333_1 1335_1 1331_1 856_1 858_1 1341_1 1334_1 1938_1 1940_1 1344_1 2221_1 2220_1 - 836_1 :  843_1 1324_1 1337_1 1342_1 2453_1 1326_1 1948_1 - 2219_1 :  830_1 2909_1 1941_1 1955_1 2862_1 T06_1 824_1 1954_1 - 1339_1 :  1328_1 2789_1 - 1939_1 :  1950_1 2441_1 2840_1 - 2794_1 :  2217_1 1949_1 - 825_1 :  - 2911_1 :  - 1327_1 :  - 1332_1 :  - 2788_1 :  - 2796_1 :  - 2445_1 :  - 2871_1 :  - 2787_1 :  - 2860_1 :  - 1953_1 :  - 835_1 :  - 1311_2 :  2181_2 2271_2 2328_2 2331_2 2338_2 2381_2 844_2 2387_2 - 2180_2 :  2216_2 2272_2 2322_2 2332_2 2352_2 2354_2 2367_2 2368_2 2369_2 2336_2 2376_2 2185_2 - 2187_2 :  - 2333_2 :  2370_2 - 2390_2 :  - 2382_2 :  - 2353_2 :  - 1207_3 :  1310_3 2380_3 2812_3 2872_3 2873_3 2874_3 2876_3 2877_3 2879_3 2880_3 2881_3 2882_3 2885_3 2886_3 2887_3 2888_3 2889_3 2890_3 2891_3 2901_3 2906_3 2907_3 2908_3 2910_3 2912_3 2915_3 2916_3 2917_3 2922_3 2923_3 2930_3 2931_3 2932_3 2933_3 2934_3 2935_3 2902_3 2924_3 2784_3 - 2275_3 :  2285_3 2464_3 2301_3 - 2287_3 :  2913_3 2480_3 2291_3 2781_3 - 2463_3 :  2465_3 - 2878_3 :  - 2883_3 :  - 2286_3 :  - 2814_3 :  - 2226_4 :  2045_4 2227_4 2228_4 2229_4 2230_4 2231_4 2232_4 2233_4 2234_4 2235_4 2236_4 TB28_4 - 840_5 :  2777_5 2779_5 2790_5 2792_5 2904_5 2918_5 2927_5 2928_5 T02_5 - 2778_5 :  2780_5 2791_5 2793_5 2926_5 T04_5 - 842_5 :  - 2900_5 :  - 1874_6 :  838_6 - 846_6 :  847_6 849_6 1313_6 1321_6 1872_6 1320_6 - 1943_6 :  1316_6 1319_6 - 1318_6 :  1875_6 1873_6 1870_6 - 1869_6 :  1942_6 - 845_6 :  - 860_6 :  - 1317_6 :  - 1871_6 :  - 2170_6 :  - 2169_6 :  - 2173_6 :  - 1309_7 :  2859_7 2443_7 - 2448_7 :  - 2442_7 :  - 2449_7 :  - 1202_8 :  1958_8 2013_8 2039_8 2478_8 2798_8 2178_8 1197_8 1561_8 2014_8 2015_8 - 1203_8 :  2799_8 - 1946_8 :  2925_8 - 1198_8 :  1200_8 2036_8 2037_8 2214_8 2457_8 2775_8 1989_8 2896_8 1199_8 1988_8 - 1957_8 :  - 2920_8 :  - 2476_8 :  - 862_9 :  861_9 - 2031_10 :  2034_10 2024_10 2029_10 2033_10 TB25_10 - 2026_10 :  - 2786_11 :  1560_11 - 2323_11 :  - 2196_12 :  2200_12 2222_12 2199_12 - 2806_12 :  2826_12 2223_12 - 2194_12 :  2818_12 2829_12 - 2201_12 :  - 2202_12 :  - 2171_12 :  - 2225_12 :  - 2224_12 :  - 2198_12 :  - 1312_12 :  - 1923_13 :  1959_13 1960_13 2184_13 2776_13 2027_13 2175_13 2215_13 1986_13 2183_13 2317_13 2337_13 2458_13 2813_13 2921_13 2038_13 T01_13 T03_13 1201_13 2035_13 2475_13 1205_13 1999_13 - 1956_13 :  - 2028_13 :  - 2044_14 :  2842_14 2845_14 2846_14 2852_14 2855_14 2479_14 2477_14 - 2042_14 :  2843_14 2844_14 2811_14 - 2850_14 :  2851_14 2854_14 2847_14 - 2848_14 :  - 2040_14 :  - 2853_14 :  - 2004_15 :  2003_15 2005_15 2008_15 2009_15 2010_15 2011_15 2012_15 2017_15 2018_15 2019_15 2020_15 2025_15 2030_15 2043_15 2849_15 - 2267_16 :  2838_16 2289_16 2290_16 2296_16 2297_16 2298_16 2299_16 2300_16 2305_16 2306_16 2307_16 2309_16 2310_16 2316_16 2325_16 2330_16 2339_16 2341_16 2355_16 2356_16 2357_16 2358_16 2359_16 2361_16 2362_16 2363_16 2364_16 2366_16 2373_16 2374_16 2379_16 2389_16 2391_16 2462_16 2303_16 - 2268_16 :  2270_16 2292_16 2294_16 2295_16 2466_16 2467_16 2470_16 2471_16 2472_16 2473_16 2474_16 - 2469_16 :  - 2269_16 :  - 2274_17 :  - 2313_18 :  2314_18 - 2278_19 :  - 2302_20 :  2903_20 2875_20 2349_20 2348_20 2329_20 2324_20 - 2342_21 :  2894_21 - 2277_22 :  - 2281_23 :  - 2866_24 :  2868_24 2867_24 - 2865_24 :  - 2801_25 :  2809_25 2802_25 - 2805_26 :  2808_26 2807_26 - 2800_27 : CONNECTIONSadding two min taxa 42[1318_6] , 40[846_6]In clusters 44 , 50 distance 1adding two min taxa 4[1339_1] , 9[1327_1]In clusters 4 , 18 distance 2adding two min taxa 3[2219_1] , 1[1945_1]In clusters 4 , 18 distance 2adding two min taxa 5[1939_1] , 2[836_1]In clusters 4 , 18 distance 2adding two min taxa 6[2794_1] , 8[2911_1]In clusters 4 , 18 distance 2adding two min taxa 0[2440_1] , 12[2796_1]In clusters 4 , 11 distance 2adding two min taxa 1[1945_1] , 11[2788_1]In clusters 4 , 11 distance 2adding two min taxa 2[836_1] , 11[2788_1]In clusters 4 , 11 distance 2adding two min taxa 38[2900_5] , 87[2267_16]In clusters 38 , 90 distance 7REAL DISTANCE MATRIXpos:   			  1  2  3  4  5  6  7  8  9  10  11  12  13  14  15  16  17  18  19  20  21  22  23  24  25  26  27  28  29  30  31  32  33  34  35  36  37  38  39  40  41  42  43  44  45  46  47  48  49  50  51  52  53  54  55  56  57  58  59  60  61  62  63  64  65  66  67  68  69  70  71  72  73  74  75  76  77  78  79  80  81  82  83  84  85  86  87  88  89  90  91  92  93  94  95  96  97  98  99  100  101  102  103 [2440_1]1 	: --  1  2  3  5  4  4  2  2  3  3  3  2  5  1  1  2  3  3  ##  ##  ##  ##  ##  ##  ##  ##  ##  ##  ##  ##  ##  ##  ##  ##  ##  ##  ##  ##  ##  ##  ##  ##  ##  ##  ##  ##  ##  ##  ##  ##  ##  ##  ##  ##  ##  ##  ##  ##  ##  ##  ##  ##  ##  ##  ##  ##  ##  ##  ##  ##  ##  ##  ##  ##  ##  ##  ##  ##  ##  ##  ##  ##  ##  ##  ##  ##  ##  ##  ##  ##  ##  ##  ##  ##  ##  ##  ##  ##  ##  ##  ##  ## [1945_1]2 	: 1  --  1  2  4  3  3  1  1  2  2  2  3  4  2  2  3  2  2  ##  ##  ##  ##  ##  ##  ##  ##  ##  ##  ##  ##  ##  ##  ##  ##  ##  ##  ##  ##  ##  ##  ##  ##  ##  ##  ##  ##  ##  ##  ##  ##  ##  ##  ##  ##  ##  ##  ##  ##  ##  ##  ##  ##  ##  ##  ##  ##  ##  ##  ##  ##  ##  ##  ##  ##  ##  ##  ##  ##  ##  ##  ##  ##  ##  ##  ##  ##  ##  ##  ##  ##  ##  ##  ##  ##  ##  ##  ##  ##  ##  ##  ##  ## [836_1]3 	: 2  1  --  3  3  2  4  2  2  1  1  2  3  3  3  3  4  1  1  ##  ##  ##  ##  ##  ##  ##  ##  ##  ##  ##  ##  ##  ##  ##  ##  ##  ##  ##  ##  ##  ##  ##  ##  ##  ##  ##  ##  ##  ##  ##  ##  ##  ##  ##  ##  ##  ##  ##  ##  ##  ##  ##  ##  ##  ##  ##  ##  ##  ##  ##  ##  ##  ##  ##  ##  ##  ##  ##  ##  ##  ##  ##  ##  ##  ##  ##  ##  ##  ##  ##  ##  ##  ##  ##  ##  ##  ##  ##  ##  ##  ##  ##  ## [2219_1]4 	: 3  2  3  --  2  1  1  3  3  4  4  4  5  2  4  4  5  4  4  ##  ##  ##  ##  ##  ##  ##  ##  ##  ##  ##  ##  ##  ##  ##  ##  ##  ##  ##  ##  ##  ##  ##  ##  ##  ##  ##  ##  ##  ##  ##  ##  ##  ##  ##  ##  ##  ##  ##  ##  ##  ##  ##  ##  ##  ##  ##  ##  ##  ##  ##  ##  ##  ##  ##  ##  ##  ##  ##  ##  ##  ##  ##  ##  ##  ##  ##  ##  ##  ##  ##  ##  ##  ##  ##  ##  ##  ##  ##  ##  ##  ##  ##  ## [1339_1]5 	: 5  4  3  2  --  1  1  5  3  2  4  5  6  2  6  6  7  4  4  ##  ##  ##  ##  ##  ##  ##  ##  ##  ##  ##  ##  ##  ##  ##  ##  ##  ##  ##  ##  ##  ##  ##  ##  ##  ##  ##  ##  ##  ##  ##  ##  ##  ##  ##  ##  ##  ##  ##  ##  ##  ##  ##  ##  ##  ##  ##  ##  ##  ##  ##  ##  ##  ##  ##  ##  ##  ##  ##  ##  ##  ##  ##  ##  ##  ##  ##  ##  ##  ##  ##  ##  ##  ##  ##  ##  ##  ##  ##  ##  ##  ##  ##  ## [1939_1]6 	: 4  3  2  1  1  --  2  4  4  3  3  4  5  1  5  5  6  3  3  ##  ##  ##  ##  ##  ##  ##  ##  ##  ##  ##  ##  ##  ##  ##  ##  ##  ##  ##  ##  ##  ##  ##  ##  ##  ##  ##  ##  ##  ##  ##  ##  ##  ##  ##  ##  ##  ##  ##  ##  ##  ##  ##  ##  ##  ##  ##  ##  ##  ##  ##  ##  ##  ##  ##  ##  ##  ##  ##  ##  ##  ##  ##  ##  ##  ##  ##  ##  ##  ##  ##  ##  ##  ##  ##  ##  ##  ##  ##  ##  ##  ##  ##  ## [2794_1]7 	: 4  3  4  1  1  2  --  4  2  3  5  5  6  3  5  5  6  5  5  ##  ##  ##  ##  ##  ##  ##  ##  ##  ##  ##  ##  ##  ##  ##  ##  ##  ##  ##  ##  ##  ##  ##  ##  ##  ##  ##  ##  ##  ##  ##  ##  ##  ##  ##  ##  ##  ##  ##  ##  ##  ##  ##  ##  ##  ##  ##  ##  ##  ##  ##  ##  ##  ##  ##  ##  ##  ##  ##  ##  ##  ##  ##  ##  ##  ##  ##  ##  ##  ##  ##  ##  ##  ##  ##  ##  ##  ##  ##  ##  ##  ##  ##  ## [825_1]8 	: 2  1  2  3  5  4  4  --  2  3  3  3  4  5  3  3  4  3  3  ##  ##  ##  ##  ##  ##  ##  ##  ##  ##  ##  ##  ##  ##  ##  ##  ##  ##  ##  ##  ##  ##  ##  ##  ##  ##  ##  ##  ##  ##  ##  ##  ##  ##  ##  ##  ##  ##  ##  ##  ##  ##  ##  ##  ##  ##  ##  ##  ##  ##  ##  ##  ##  ##  ##  ##  ##  ##  ##  ##  ##  ##  ##  ##  ##  ##  ##  ##  ##  ##  ##  ##  ##  ##  ##  ##  ##  ##  ##  ##  ##  ##  ##  ## [2911_1]9 	: 2  1  2  3  3  4  2  2  --  1  3  3  4  5  3  3  4  3  3  ##  ##  ##  ##  ##  ##  ##  ##  ##  ##  ##  ##  ##  ##  ##  ##  ##  ##  ##  ##  ##  ##  ##  ##  ##  ##  ##  ##  ##  ##  ##  ##  ##  ##  ##  ##  ##  ##  ##  ##  ##  ##  ##  ##  ##  ##  ##  ##  ##  ##  ##  ##  ##  ##  ##  ##  ##  ##  ##  ##  ##  ##  ##  ##  ##  ##  ##  ##  ##  ##  ##  ##  ##  ##  ##  ##  ##  ##  ##  ##  ##  ##  ##  ## [1327_1]10	: 3  2  1  4  2  3  3  3  1  --  2  3  4  4  4  4  5  2  2  ##  ##  ##  ##  ##  ##  ##  ##  ##  ##  ##  ##  ##  ##  ##  ##  ##  ##  ##  ##  ##  ##  ##  ##  ##  ##  ##  ##  ##  ##  ##  ##  ##  ##  ##  ##  ##  ##  ##  ##  ##  ##  ##  ##  ##  ##  ##  ##  ##  ##  ##  ##  ##  ##  ##  ##  ##  ##  ##  ##  ##  ##  ##  ##  ##  ##  ##  ##  ##  ##  ##  ##  ##  ##  ##  ##  ##  ##  ##  ##  ##  ##  ##  ## [1332_1]11	: 3  2  1  4  4  3  5  3  3  2  --  3  4  4  4  4  5  2  2  ##  ##  ##  ##  ##  ##  ##  ##  ##  ##  ##  ##  ##  ##  ##  ##  ##  ##  ##  ##  ##  ##  ##  ##  ##  ##  ##  ##  ##  ##  ##  ##  ##  ##  ##  ##  ##  ##  ##  ##  ##  ##  ##  ##  ##  ##  ##  ##  ##  ##  ##  ##  ##  ##  ##  ##  ##  ##  ##  ##  ##  ##  ##  ##  ##  ##  ##  ##  ##  ##  ##  ##  ##  ##  ##  ##  ##  ##  ##  ##  ##  ##  ##  ## [2788_1]12	: 3  2  2  4  5  4  5  3  3  3  3  --  1  5  4  5  4  3  3  ##  ##  ##  ##  ##  ##  ##  ##  ##  ##  ##  ##  ##  ##  ##  ##  ##  ##  ##  ##  ##  ##  ##  ##  ##  ##  ##  ##  ##  ##  ##  ##  ##  ##  ##  ##  ##  ##  ##  ##  ##  ##  ##  ##  ##  ##  ##  ##  ##  ##  ##  ##  ##  ##  ##  ##  ##  ##  ##  ##  ##  ##  ##  ##  ##  ##  ##  ##  ##  ##  ##  ##  ##  ##  ##  ##  ##  ##  ##  ##  ##  ##  ##  ## [2796_1]13	: 2  3  3  5  6  5  6  4  4  4  4  1  --  6  3  4  3  4  4  ##  ##  ##  ##  ##  ##  ##  ##  ##  ##  ##  ##  ##  ##  ##  ##  ##  ##  ##  ##  ##  ##  ##  ##  ##  ##  ##  ##  ##  ##  ##  ##  ##  ##  ##  ##  ##  ##  ##  ##  ##  ##  ##  ##  ##  ##  ##  ##  ##  ##  ##  ##  ##  ##  ##  ##  ##  ##  ##  ##  ##  ##  ##  ##  ##  ##  ##  ##  ##  ##  ##  ##  ##  ##  ##  ##  ##  ##  ##  ##  ##  ##  ##  ## [2445_1]14	: 5  4  3  2  2  1  3  5  5  4  4  5  6  --  6  6  7  4  4  ##  ##  ##  ##  ##  ##  ##  ##  ##  ##  ##  ##  ##  ##  ##  ##  ##  ##  ##  ##  ##  ##  ##  ##  ##  ##  ##  ##  ##  ##  ##  ##  ##  ##  ##  ##  ##  ##  ##  ##  ##  ##  ##  ##  ##  ##  ##  ##  ##  ##  ##  ##  ##  ##  ##  ##  ##  ##  ##  ##  ##  ##  ##  ##  ##  ##  ##  ##  ##  ##  ##  ##  ##  ##  ##  ##  ##  ##  ##  ##  ##  ##  ##  ## [2871_1]15	: 1  2  3  4  6  5  5  3  3  4  4  4  3  6  --  2  1  4  4  ##  ##  ##  ##  ##  ##  ##  ##  ##  ##  ##  ##  ##  ##  ##  ##  ##  ##  ##  ##  ##  ##  ##  ##  ##  ##  ##  ##  ##  ##  ##  ##  ##  ##  ##  ##  ##  ##  ##  ##  ##  ##  ##  ##  ##  ##  ##  ##  ##  ##  ##  ##  ##  ##  ##  ##  ##  ##  ##  ##  ##  ##  ##  ##  ##  ##  ##  ##  ##  ##  ##  ##  ##  ##  ##  ##  ##  ##  ##  ##  ##  ##  ##  ## [2787_1]16	: 1  2  3  4  6  5  5  3  3  4  4  5  4  6  2  --  3  4  4  ##  ##  ##  ##  ##  ##  ##  ##  ##  ##  ##  ##  ##  ##  ##  ##  ##  ##  ##  ##  ##  ##  ##  ##  ##  ##  ##  ##  ##  ##  ##  ##  ##  ##  ##  ##  ##  ##  ##  ##  ##  ##  ##  ##  ##  ##  ##  ##  ##  ##  ##  ##  ##  ##  ##  ##  ##  ##  ##  ##  ##  ##  ##  ##  ##  ##  ##  ##  ##  ##  ##  ##  ##  ##  ##  ##  ##  ##  ##  ##  ##  ##  ##  ## [2860_1]17	: 2  3  4  5  7  6  6  4  4  5  5  4  3  7  1  3  --  5  5  ##  ##  ##  ##  ##  ##  ##  ##  ##  ##  ##  ##  ##  ##  ##  ##  ##  ##  ##  ##  ##  ##  ##  ##  ##  ##  ##  ##  ##  ##  ##  ##  ##  ##  ##  ##  ##  ##  ##  ##  ##  ##  ##  ##  ##  ##  ##  ##  ##  ##  ##  ##  ##  ##  ##  ##  ##  ##  ##  ##  ##  ##  ##  ##  ##  ##  ##  ##  ##  ##  ##  ##  ##  ##  ##  ##  ##  ##  ##  ##  ##  ##  ##  ## [1953_1]18	: 3  2  1  4  4  3  5  3  3  2  2  3  4  4  4  4  5  --  2  ##  ##  ##  ##  ##  ##  ##  ##  ##  ##  ##  ##  ##  ##  ##  ##  ##  ##  ##  ##  ##  ##  ##  ##  ##  ##  ##  ##  ##  ##  ##  ##  ##  ##  ##  ##  ##  ##  ##  ##  ##  ##  ##  ##  ##  ##  ##  ##  ##  ##  ##  ##  ##  ##  ##  ##  ##  ##  ##  ##  ##  ##  ##  ##  ##  ##  ##  ##  ##  ##  ##  ##  ##  ##  ##  ##  ##  ##  ##  ##  ##  ##  ##  ## [835_1]19	: 3  2  1  4  4  3  5  3  3  2  2  3  4  4  4  4  5  2  --  ##  ##  ##  ##  ##  ##  ##  ##  ##  ##  ##  ##  ##  ##  ##  ##  ##  ##  ##  ##  ##  ##  ##  ##  ##  ##  ##  ##  ##  ##  ##  ##  ##  ##  ##  ##  ##  ##  ##  ##  ##  ##  ##  ##  ##  ##  ##  ##  ##  ##  ##  ##  ##  ##  ##  ##  ##  ##  ##  ##  ##  ##  ##  ##  ##  ##  ##  ##  ##  ##  ##  ##  ##  ##  ##  ##  ##  ##  ##  ##  ##  ##  ##  ## [1311_2]20	: ##  ##  ##  ##  ##  ##  ##  ##  ##  ##  ##  ##  ##  ##  ##  ##  ##  ##  ##  --  1  1  1  2  4  2  ##  ##  ##  ##  ##  ##  ##  ##  ##  ##  ##  ##  ##  ##  ##  ##  ##  ##  ##  ##  ##  ##  ##  ##  ##  ##  ##  ##  ##  ##  ##  ##  ##  ##  ##  ##  ##  ##  ##  ##  ##  ##  ##  ##  ##  ##  ##  ##  ##  ##  ##  ##  ##  ##  ##  ##  ##  ##  ##  ##  ##  ##  ##  ##  ##  ##  ##  ##  ##  ##  ##  ##  ##  ##  ##  ##  ## [2180_2]21	: ##  ##  ##  ##  ##  ##  ##  ##  ##  ##  ##  ##  ##  ##  ##  ##  ##  ##  ##  1  --  2  2  3  3  1  ##  ##  ##  ##  ##  ##  ##  ##  ##  ##  ##  ##  ##  ##  ##  ##  ##  ##  ##  ##  ##  ##  ##  ##  ##  ##  ##  ##  ##  ##  ##  ##  ##  ##  ##  ##  ##  ##  ##  ##  ##  ##  ##  ##  ##  ##  ##  ##  ##  ##  ##  ##  ##  ##  ##  ##  ##  ##  ##  ##  ##  ##  ##  ##  ##  ##  ##  ##  ##  ##  ##  ##  ##  ##  ##  ##  ## [2187_2]22	: ##  ##  ##  ##  ##  ##  ##  ##  ##  ##  ##  ##  ##  ##  ##  ##  ##  ##  ##  1  2  --  2  3  5  3  ##  ##  ##  ##  ##  ##  ##  ##  ##  ##  ##  ##  ##  ##  ##  ##  ##  ##  ##  ##  ##  ##  ##  ##  ##  ##  ##  ##  ##  ##  ##  ##  ##  ##  ##  ##  ##  ##  ##  ##  ##  ##  ##  ##  ##  ##  ##  ##  ##  ##  ##  ##  ##  ##  ##  ##  ##  ##  ##  ##  ##  ##  ##  ##  ##  ##  ##  ##  ##  ##  ##  ##  ##  ##  ##  ##  ## [2333_2]23	: ##  ##  ##  ##  ##  ##  ##  ##  ##  ##  ##  ##  ##  ##  ##  ##  ##  ##  ##  1  2  2  --  1  3  1  ##  ##  ##  ##  ##  ##  ##  ##  ##  ##  ##  ##  ##  ##  ##  ##  ##  ##  ##  ##  ##  ##  ##  ##  ##  ##  ##  ##  ##  ##  ##  ##  ##  ##  ##  ##  ##  ##  ##  ##  ##  ##  ##  ##  ##  ##  ##  ##  ##  ##  ##  ##  ##  ##  ##  ##  ##  ##  ##  ##  ##  ##  ##  ##  ##  ##  ##  ##  ##  ##  ##  ##  ##  ##  ##  ##  ## [2390_2]24	: ##  ##  ##  ##  ##  ##  ##  ##  ##  ##  ##  ##  ##  ##  ##  ##  ##  ##  ##  2  3  3  1  --  2  2  ##  ##  ##  ##  ##  ##  ##  ##  ##  ##  ##  ##  ##  ##  ##  ##  ##  ##  ##  ##  ##  ##  ##  ##  ##  ##  ##  ##  ##  ##  ##  ##  ##  ##  ##  ##  ##  ##  ##  ##  ##  ##  ##  ##  ##  ##  ##  ##  ##  ##  ##  ##  ##  ##  ##  ##  ##  ##  ##  ##  ##  ##  ##  ##  ##  ##  ##  ##  ##  ##  ##  ##  ##  ##  ##  ##  ## [2382_2]25	: ##  ##  ##  ##  ##  ##  ##  ##  ##  ##  ##  ##  ##  ##  ##  ##  ##  ##  ##  4  3  5  3  2  --  2  ##  ##  ##  ##  ##  ##  ##  ##  ##  ##  ##  ##  ##  ##  ##  ##  ##  ##  ##  ##  ##  ##  ##  ##  ##  ##  ##  ##  ##  ##  ##  ##  ##  ##  ##  ##  ##  ##  ##  ##  ##  ##  ##  ##  ##  ##  ##  ##  ##  ##  ##  ##  ##  ##  ##  ##  ##  ##  ##  ##  ##  ##  ##  ##  ##  ##  ##  ##  ##  ##  ##  ##  ##  ##  ##  ##  ## [2353_2]26	: ##  ##  ##  ##  ##  ##  ##  ##  ##  ##  ##  ##  ##  ##  ##  ##  ##  ##  ##  2  1  3  1  2  2  --  ##  ##  ##  ##  ##  ##  ##  ##  ##  ##  ##  ##  ##  ##  ##  ##  ##  ##  ##  ##  ##  ##  ##  ##  ##  ##  ##  ##  ##  ##  ##  ##  ##  ##  ##  ##  ##  ##  ##  ##  ##  ##  ##  ##  ##  ##  ##  ##  ##  ##  ##  ##  ##  ##  ##  ##  ##  ##  ##  ##  ##  ##  ##  ##  ##  ##  ##  ##  ##  ##  ##  ##  ##  ##  ##  ##  ## [1207_3]27	: ##  ##  ##  ##  ##  ##  ##  ##  ##  ##  ##  ##  ##  ##  ##  ##  ##  ##  ##  ##  ##  ##  ##  ##  ##  ##  --  2  1  2  1  1  3  9  ##  ##  ##  ##  ##  ##  ##  ##  ##  ##  ##  ##  ##  ##  ##  ##  ##  ##  ##  ##  ##  ##  ##  ##  ##  ##  ##  ##  ##  ##  ##  ##  ##  ##  ##  ##  ##  ##  ##  ##  ##  ##  ##  ##  ##  ##  ##  ##  ##  ##  ##  ##  ##  ##  ##  ##  ##  ##  ##  ##  ##  ##  ##  ##  ##  ##  ##  ##  ## [2275_3]28	: ##  ##  ##  ##  ##  ##  ##  ##  ##  ##  ##  ##  ##  ##  ##  ##  ##  ##  ##  ##  ##  ##  ##  ##  ##  ##  2  --  1  2  2  3  1  9  ##  ##  ##  ##  ##  ##  ##  ##  ##  ##  ##  ##  ##  ##  ##  ##  ##  ##  ##  ##  ##  ##  ##  ##  ##  ##  ##  ##  ##  ##  ##  ##  ##  ##  ##  ##  ##  ##  ##  ##  ##  ##  ##  ##  ##  ##  ##  ##  ##  ##  ##  ##  ##  ##  ##  ##  ##  ##  ##  ##  ##  ##  ##  ##  ##  ##  ##  ##  ## [2287_3]29	: ##  ##  ##  ##  ##  ##  ##  ##  ##  ##  ##  ##  ##  ##  ##  ##  ##  ##  ##  ##  ##  ##  ##  ##  ##  ##  1  1  --  1  1  2  2  8  ##  ##  ##  ##  ##  ##  ##  ##  ##  ##  ##  ##  ##  ##  ##  ##  ##  ##  ##  ##  ##  ##  ##  ##  ##  ##  ##  ##  ##  ##  ##  ##  ##  ##  ##  ##  ##  ##  ##  ##  ##  ##  ##  ##  ##  ##  ##  ##  ##  ##  ##  ##  ##  ##  ##  ##  ##  ##  ##  ##  ##  ##  ##  ##  ##  ##  ##  ##  ## [2463_3]30	: ##  ##  ##  ##  ##  ##  ##  ##  ##  ##  ##  ##  ##  ##  ##  ##  ##  ##  ##  ##  ##  ##  ##  ##  ##  ##  2  2  1  --  2  3  3  9  ##  ##  ##  ##  ##  ##  ##  ##  ##  ##  ##  ##  ##  ##  ##  ##  ##  ##  ##  ##  ##  ##  ##  ##  ##  ##  ##  ##  ##  ##  ##  ##  ##  ##  ##  ##  ##  ##  ##  ##  ##  ##  ##  ##  ##  ##  ##  ##  ##  ##  ##  ##  ##  ##  ##  ##  ##  ##  ##  ##  ##  ##  ##  ##  ##  ##  ##  ##  ## [2878_3]31	: ##  ##  ##  ##  ##  ##  ##  ##  ##  ##  ##  ##  ##  ##  ##  ##  ##  ##  ##  ##  ##  ##  ##  ##  ##  ##  1  2  1  2  --  2  3  9  ##  ##  ##  ##  ##  ##  ##  ##  ##  ##  ##  ##  ##  ##  ##  ##  ##  ##  ##  ##  ##  ##  ##  ##  ##  ##  ##  ##  ##  ##  ##  ##  ##  ##  ##  ##  ##  ##  ##  ##  ##  ##  ##  ##  ##  ##  ##  ##  ##  ##  ##  ##  ##  ##  ##  ##  ##  ##  ##  ##  ##  ##  ##  ##  ##  ##  ##  ##  ## [2883_3]32	: ##  ##  ##  ##  ##  ##  ##  ##  ##  ##  ##  ##  ##  ##  ##  ##  ##  ##  ##  ##  ##  ##  ##  ##  ##  ##  1  3  2  3  2  --  4  10  ##  ##  ##  ##  ##  ##  ##  ##  ##  ##  ##  ##  ##  ##  ##  ##  ##  ##  ##  ##  ##  ##  ##  ##  ##  ##  ##  ##  ##  ##  ##  ##  ##  ##  ##  ##  ##  ##  ##  ##  ##  ##  ##  ##  ##  ##  ##  ##  ##  ##  ##  ##  ##  ##  ##  ##  ##  ##  ##  ##  ##  ##  ##  ##  ##  ##  ##  ##  ## [2286_3]33	: ##  ##  ##  ##  ##  ##  ##  ##  ##  ##  ##  ##  ##  ##  ##  ##  ##  ##  ##  ##  ##  ##  ##  ##  ##  ##  3  1  2  3  3  4  --  8  ##  ##  ##  ##  ##  ##  ##  ##  ##  ##  ##  ##  ##  ##  ##  ##  ##  ##  ##  ##  ##  ##  ##  ##  ##  ##  ##  ##  ##  ##  ##  ##  ##  ##  ##  ##  ##  ##  ##  ##  ##  ##  ##  ##  ##  ##  ##  ##  ##  ##  ##  ##  ##  ##  ##  ##  ##  ##  ##  ##  ##  ##  ##  ##  ##  ##  ##  ##  ## [2814_3]34	: ##  ##  ##  ##  ##  ##  ##  ##  ##  ##  ##  ##  ##  ##  ##  ##  ##  ##  ##  ##  ##  ##  ##  ##  ##  ##  9  9  8  9  9  10  8  --  ##  ##  ##  ##  ##  ##  ##  ##  ##  ##  ##  ##  ##  ##  ##  ##  ##  ##  ##  ##  ##  ##  ##  ##  ##  ##  ##  ##  ##  ##  ##  ##  ##  ##  ##  ##  ##  ##  ##  ##  ##  ##  ##  ##  ##  ##  ##  ##  ##  ##  ##  ##  ##  ##  ##  ##  ##  ##  ##  ##  ##  ##  ##  ##  ##  ##  ##  ##  ## [2226_4]35	: ##  ##  ##  ##  ##  ##  ##  ##  ##  ##  ##  ##  ##  ##  ##  ##  ##  ##  ##  ##  ##  ##  ##  ##  ##  ##  ##  ##  ##  ##  ##  ##  ##  ##  --  ##  ##  ##  ##  ##  ##  ##  ##  ##  ##  ##  ##  ##  ##  ##  ##  ##  ##  ##  ##  ##  ##  ##  ##  ##  ##  ##  ##  ##  ##  ##  ##  ##  ##  ##  ##  ##  ##  ##  ##  ##  ##  ##  ##  ##  ##  ##  ##  ##  ##  ##  ##  ##  ##  ##  ##  ##  ##  ##  ##  ##  ##  ##  ##  ##  ##  3  7 [840_5]36	: ##  ##  ##  ##  ##  ##  ##  ##  ##  ##  ##  ##  ##  ##  ##  ##  ##  ##  ##  ##  ##  ##  ##  ##  ##  ##  ##  ##  ##  ##  ##  ##  ##  ##  ##  --  1  1  4  ##  ##  ##  ##  ##  ##  ##  ##  ##  ##  ##  ##  ##  ##  ##  ##  ##  ##  ##  ##  ##  ##  ##  ##  ##  ##  ##  ##  ##  ##  ##  ##  ##  ##  ##  ##  ##  ##  ##  ##  ##  ##  ##  ##  ##  ##  ##  ##  9  10  11  11  ##  ##  ##  ##  ##  ##  ##  ##  ##  ##  ##  ## [2778_5]37	: ##  ##  ##  ##  ##  ##  ##  ##  ##  ##  ##  ##  ##  ##  ##  ##  ##  ##  ##  ##  ##  ##  ##  ##  ##  ##  ##  ##  ##  ##  ##  ##  ##  ##  ##  1  --  2  3  ##  ##  ##  ##  ##  ##  ##  ##  ##  ##  ##  ##  ##  ##  ##  ##  ##  ##  ##  ##  ##  ##  ##  ##  ##  ##  ##  ##  ##  ##  ##  ##  ##  ##  ##  ##  ##  ##  ##  ##  ##  ##  ##  ##  ##  ##  ##  ##  8  9  10  10  ##  ##  ##  ##  ##  ##  ##  ##  ##  ##  ##  ## [842_5]38	: ##  ##  ##  ##  ##  ##  ##  ##  ##  ##  ##  ##  ##  ##  ##  ##  ##  ##  ##  ##  ##  ##  ##  ##  ##  ##  ##  ##  ##  ##  ##  ##  ##  ##  ##  1  2  --  5  ##  ##  ##  ##  ##  ##  ##  ##  ##  ##  ##  ##  ##  ##  ##  ##  ##  ##  ##  ##  ##  ##  ##  ##  ##  ##  ##  ##  ##  ##  ##  ##  ##  ##  ##  ##  ##  ##  ##  ##  ##  ##  ##  ##  ##  ##  ##  ##  10  11  12  12  ##  ##  ##  ##  ##  ##  ##  ##  ##  ##  ##  ## [2900_5]39	: ##  ##  ##  ##  ##  ##  ##  ##  ##  ##  ##  ##  ##  ##  ##  ##  ##  ##  ##  ##  ##  ##  ##  ##  ##  ##  ##  ##  ##  ##  ##  ##  ##  ##  ##  4  3  5  --  ##  ##  ##  ##  ##  ##  ##  ##  ##  ##  ##  ##  ##  ##  ##  ##  ##  ##  ##  ##  ##  ##  ##  ##  ##  ##  ##  ##  ##  ##  ##  ##  ##  ##  ##  ##  ##  ##  ##  ##  ##  ##  ##  ##  ##  ##  ##  ##  7  9  10  10  ##  ##  ##  ##  ##  ##  ##  ##  ##  ##  ##  ## [1874_6]40	: ##  ##  ##  ##  ##  ##  ##  ##  ##  ##  ##  ##  ##  ##  ##  ##  ##  ##  ##  ##  ##  ##  ##  ##  ##  ##  ##  ##  ##  ##  ##  ##  ##  ##  ##  ##  ##  ##  ##  --  3  4  2  1  2  11  1  1  1  4  4  ##  ##  ##  ##  ##  ##  ##  ##  ##  ##  ##  ##  ##  ##  ##  ##  ##  ##  ##  ##  ##  ##  ##  ##  ##  ##  ##  ##  ##  ##  ##  ##  ##  ##  ##  ##  ##  ##  ##  ##  ##  ##  ##  ##  ##  ##  ##  ##  ##  ##  ##  ## [846_6]41	: ##  ##  ##  ##  ##  ##  ##  ##  ##  ##  ##  ##  ##  ##  ##  ##  ##  ##  ##  ##  ##  ##  ##  ##  ##  ##  ##  ##  ##  ##  ##  ##  ##  ##  ##  ##  ##  ##  ##  3  --  1  1  2  5  8  2  2  3  1  1  ##  ##  ##  ##  ##  ##  ##  ##  ##  ##  ##  ##  ##  ##  ##  ##  ##  ##  ##  ##  ##  ##  ##  ##  ##  ##  ##  ##  ##  ##  ##  ##  ##  ##  ##  ##  ##  ##  ##  ##  ##  ##  ##  ##  ##  ##  ##  ##  ##  ##  ##  ## [1943_6]42	: ##  ##  ##  ##  ##  ##  ##  ##  ##  ##  ##  ##  ##  ##  ##  ##  ##  ##  ##  ##  ##  ##  ##  ##  ##  ##  ##  ##  ##  ##  ##  ##  ##  ##  ##  ##  ##  ##  ##  4  1  --  2  4  8  9  3  3  4  2  2  ##  ##  ##  ##  ##  ##  ##  ##  ##  ##  ##  ##  ##  ##  ##  ##  ##  ##  ##  ##  ##  ##  ##  ##  ##  ##  ##  ##  ##  ##  ##  ##  ##  ##  ##  ##  ##  ##  ##  ##  ##  ##  ##  ##  ##  ##  ##  ##  ##  ##  ##  ## [1318_6]43	: ##  ##  ##  ##  ##  ##  ##  ##  ##  ##  ##  ##  ##  ##  ##  ##  ##  ##  ##  ##  ##  ##  ##  ##  ##  ##  ##  ##  ##  ##  ##  ##  ##  ##  ##  ##  ##  ##  ##  2  1  2  --  2  6  9  1  1  2  2  2  ##  ##  ##  ##  ##  ##  ##  ##  ##  ##  ##  ##  ##  ##  ##  ##  ##  ##  ##  ##  ##  ##  ##  ##  ##  ##  ##  ##  ##  ##  ##  ##  ##  ##  ##  ##  ##  ##  ##  ##  ##  ##  ##  ##  ##  ##  ##  ##  ##  ##  ##  ## [1869_6]44	: ##  ##  ##  ##  ##  ##  ##  ##  ##  ##  ##  ##  ##  ##  ##  ##  ##  ##  ##  ##  ##  ##  ##  ##  ##  ##  ##  ##  ##  ##  ##  ##  ##  ##  ##  ##  ##  ##  ##  1  2  4  2  --  4  10  3  1  2  3  3  ##  ##  ##  ##  ##  ##  ##  ##  ##  ##  ##  ##  ##  ##  ##  ##  ##  ##  ##  ##  ##  ##  ##  ##  ##  ##  ##  ##  ##  ##  ##  ##  ##  ##  ##  ##  ##  ##  ##  ##  ##  ##  ##  ##  ##  ##  ##  ##  ##  ##  ##  ## [845_6]45	: ##  ##  ##  ##  ##  ##  ##  ##  ##  ##  ##  ##  ##  ##  ##  ##  ##  ##  ##  ##  ##  ##  ##  ##  ##  ##  ##  ##  ##  ##  ##  ##  ##  ##  ##  ##  ##  ##  ##  2  5  8  6  4  --  13  4  4  3  6  5  ##  ##  ##  ##  ##  ##  ##  ##  ##  ##  ##  ##  ##  ##  ##  ##  ##  ##  ##  ##  ##  ##  ##  ##  ##  ##  ##  ##  ##  ##  ##  ##  ##  ##  ##  ##  ##  ##  ##  ##  ##  ##  ##  ##  ##  ##  ##  ##  ##  ##  ##  ## [860_6]46	: ##  ##  ##  ##  ##  ##  ##  ##  ##  ##  ##  ##  ##  ##  ##  ##  ##  ##  ##  ##  ##  ##  ##  ##  ##  ##  ##  ##  ##  ##  ##  ##  ##  ##  ##  ##  ##  ##  ##  11  8  9  9  10  13  --  10  10  10  9  9  ##  ##  ##  ##  ##  ##  ##  ##  ##  ##  ##  ##  ##  ##  ##  ##  ##  ##  ##  ##  ##  ##  ##  ##  ##  ##  ##  ##  ##  ##  ##  ##  ##  ##  ##  ##  ##  ##  ##  ##  ##  ##  ##  ##  ##  ##  ##  ##  ##  ##  ##  ## [1317_6]47	: ##  ##  ##  ##  ##  ##  ##  ##  ##  ##  ##  ##  ##  ##  ##  ##  ##  ##  ##  ##  ##  ##  ##  ##  ##  ##  ##  ##  ##  ##  ##  ##  ##  ##  ##  ##  ##  ##  ##  1  2  3  1  3  4  10  --  2  1  3  3  ##  ##  ##  ##  ##  ##  ##  ##  ##  ##  ##  ##  ##  ##  ##  ##  ##  ##  ##  ##  ##  ##  ##  ##  ##  ##  ##  ##  ##  ##  ##  ##  ##  ##  ##  ##  ##  ##  ##  ##  ##  ##  ##  ##  ##  ##  ##  ##  ##  ##  ##  ## [1871_6]48	: ##  ##  ##  ##  ##  ##  ##  ##  ##  ##  ##  ##  ##  ##  ##  ##  ##  ##  ##  ##  ##  ##  ##  ##  ##  ##  ##  ##  ##  ##  ##  ##  ##  ##  ##  ##  ##  ##  ##  1  2  3  1  1  4  10  2  --  2  3  3  ##  ##  ##  ##  ##  ##  ##  ##  ##  ##  ##  ##  ##  ##  ##  ##  ##  ##  ##  ##  ##  ##  ##  ##  ##  ##  ##  ##  ##  ##  ##  ##  ##  ##  ##  ##  ##  ##  ##  ##  ##  ##  ##  ##  ##  ##  ##  ##  ##  ##  ##  ## [2170_6]49	: ##  ##  ##  ##  ##  ##  ##  ##  ##  ##  ##  ##  ##  ##  ##  ##  ##  ##  ##  ##  ##  ##  ##  ##  ##  ##  ##  ##  ##  ##  ##  ##  ##  ##  ##  ##  ##  ##  ##  1  3  4  2  2  3  10  1  2  --  4  4  ##  ##  ##  ##  ##  ##  ##  ##  ##  ##  ##  ##  ##  ##  ##  ##  ##  ##  ##  ##  ##  ##  ##  ##  ##  ##  ##  ##  ##  ##  ##  ##  ##  ##  ##  ##  ##  ##  ##  ##  ##  ##  ##  ##  ##  ##  ##  ##  ##  ##  ##  ## [2169_6]50	: ##  ##  ##  ##  ##  ##  ##  ##  ##  ##  ##  ##  ##  ##  ##  ##  ##  ##  ##  ##  ##  ##  ##  ##  ##  ##  ##  ##  ##  ##  ##  ##  ##  ##  ##  ##  ##  ##  ##  4  1  2  2  3  6  9  3  3  4  --  2  ##  ##  ##  ##  ##  ##  ##  ##  ##  ##  ##  ##  ##  ##  ##  ##  ##  ##  ##  ##  ##  ##  ##  ##  ##  ##  ##  ##  ##  ##  ##  ##  ##  ##  ##  ##  ##  ##  ##  ##  ##  ##  ##  ##  ##  ##  ##  ##  ##  ##  ##  ## [2173_6]51	: ##  ##  ##  ##  ##  ##  ##  ##  ##  ##  ##  ##  ##  ##  ##  ##  ##  ##  ##  ##  ##  ##  ##  ##  ##  ##  ##  ##  ##  ##  ##  ##  ##  ##  ##  ##  ##  ##  ##  4  1  2  2  3  5  9  3  3  4  2  --  ##  ##  ##  ##  ##  ##  ##  ##  ##  ##  ##  ##  ##  ##  ##  ##  ##  ##  ##  ##  ##  ##  ##  ##  ##  ##  ##  ##  ##  ##  ##  ##  ##  ##  ##  ##  ##  ##  ##  ##  ##  ##  ##  ##  ##  ##  ##  ##  ##  ##  ##  ## [1309_7]52	: ##  ##  ##  ##  ##  ##  ##  ##  ##  ##  ##  ##  ##  ##  ##  ##  ##  ##  ##  ##  ##  ##  ##  ##  ##  ##  ##  ##  ##  ##  ##  ##  ##  ##  ##  ##  ##  ##  ##  ##  ##  ##  ##  ##  ##  ##  ##  ##  ##  ##  ##  --  1  6  2  ##  ##  ##  ##  ##  ##  ##  ##  ##  ##  ##  ##  ##  ##  ##  ##  ##  ##  ##  ##  ##  ##  ##  ##  ##  ##  ##  ##  ##  ##  ##  ##  ##  ##  ##  ##  ##  ##  ##  ##  ##  ##  ##  ##  ##  ##  ##  ## [2448_7]53	: ##  ##  ##  ##  ##  ##  ##  ##  ##  ##  ##  ##  ##  ##  ##  ##  ##  ##  ##  ##  ##  ##  ##  ##  ##  ##  ##  ##  ##  ##  ##  ##  ##  ##  ##  ##  ##  ##  ##  ##  ##  ##  ##  ##  ##  ##  ##  ##  ##  ##  ##  1  --  7  1  ##  ##  ##  ##  ##  ##  ##  ##  ##  ##  ##  ##  ##  ##  ##  ##  ##  ##  ##  ##  ##  ##  ##  ##  ##  ##  ##  ##  ##  ##  ##  ##  ##  ##  ##  ##  ##  ##  ##  ##  ##  ##  ##  ##  ##  ##  ##  ## [2442_7]54	: ##  ##  ##  ##  ##  ##  ##  ##  ##  ##  ##  ##  ##  ##  ##  ##  ##  ##  ##  ##  ##  ##  ##  ##  ##  ##  ##  ##  ##  ##  ##  ##  ##  ##  ##  ##  ##  ##  ##  ##  ##  ##  ##  ##  ##  ##  ##  ##  ##  ##  ##  6  7  --  8  ##  ##  ##  ##  ##  ##  ##  ##  ##  ##  ##  ##  ##  ##  ##  ##  ##  ##  ##  ##  ##  ##  ##  ##  ##  ##  ##  ##  ##  ##  ##  ##  ##  ##  ##  ##  ##  ##  ##  ##  ##  ##  ##  ##  ##  ##  ##  ## [2449_7]55	: ##  ##  ##  ##  ##  ##  ##  ##  ##  ##  ##  ##  ##  ##  ##  ##  ##  ##  ##  ##  ##  ##  ##  ##  ##  ##  ##  ##  ##  ##  ##  ##  ##  ##  ##  ##  ##  ##  ##  ##  ##  ##  ##  ##  ##  ##  ##  ##  ##  ##  ##  2  1  8  --  ##  ##  ##  ##  ##  ##  ##  ##  ##  ##  ##  ##  ##  ##  ##  ##  ##  ##  ##  ##  ##  ##  ##  ##  ##  ##  ##  ##  ##  ##  ##  ##  ##  ##  ##  ##  ##  ##  ##  ##  ##  ##  ##  ##  ##  ##  ##  ## [1202_8]56	: ##  ##  ##  ##  ##  ##  ##  ##  ##  ##  ##  ##  ##  ##  ##  ##  ##  ##  ##  ##  ##  ##  ##  ##  ##  ##  ##  ##  ##  ##  ##  ##  ##  ##  ##  ##  ##  ##  ##  ##  ##  ##  ##  ##  ##  ##  ##  ##  ##  ##  ##  ##  ##  ##  ##  --  2  1  2  1  1  2  ##  ##  ##  ##  ##  ##  ##  ##  ##  ##  ##  ##  ##  ##  ##  ##  ##  ##  ##  ##  ##  ##  ##  ##  ##  ##  ##  ##  ##  ##  ##  ##  ##  ##  ##  ##  ##  ##  ##  ##  ## [1203_8]57	: ##  ##  ##  ##  ##  ##  ##  ##  ##  ##  ##  ##  ##  ##  ##  ##  ##  ##  ##  ##  ##  ##  ##  ##  ##  ##  ##  ##  ##  ##  ##  ##  ##  ##  ##  ##  ##  ##  ##  ##  ##  ##  ##  ##  ##  ##  ##  ##  ##  ##  ##  ##  ##  ##  ##  2  --  1  2  3  3  2  ##  ##  ##  ##  ##  ##  ##  ##  ##  ##  ##  ##  ##  ##  ##  ##  ##  ##  ##  ##  ##  ##  ##  ##  ##  ##  ##  ##  ##  ##  ##  ##  ##  ##  ##  ##  ##  ##  ##  ##  ## [1946_8]58	: ##  ##  ##  ##  ##  ##  ##  ##  ##  ##  ##  ##  ##  ##  ##  ##  ##  ##  ##  ##  ##  ##  ##  ##  ##  ##  ##  ##  ##  ##  ##  ##  ##  ##  ##  ##  ##  ##  ##  ##  ##  ##  ##  ##  ##  ##  ##  ##  ##  ##  ##  ##  ##  ##  ##  1  1  --  1  2  2  1  ##  ##  ##  ##  ##  ##  ##  ##  ##  ##  ##  ##  ##  ##  ##  ##  ##  ##  ##  ##  ##  ##  ##  ##  ##  ##  ##  ##  ##  ##  ##  ##  ##  ##  ##  ##  ##  ##  ##  ##  ## [1198_8]59	: ##  ##  ##  ##  ##  ##  ##  ##  ##  ##  ##  ##  ##  ##  ##  ##  ##  ##  ##  ##  ##  ##  ##  ##  ##  ##  ##  ##  ##  ##  ##  ##  ##  ##  ##  ##  ##  ##  ##  ##  ##  ##  ##  ##  ##  ##  ##  ##  ##  ##  ##  ##  ##  ##  ##  2  2  1  --  3  3  2  ##  ##  ##  ##  ##  ##  ##  ##  ##  ##  ##  ##  ##  ##  ##  ##  ##  ##  ##  ##  ##  ##  ##  ##  ##  ##  ##  ##  ##  ##  ##  ##  ##  ##  ##  ##  ##  ##  ##  ##  ## [1957_8]60	: ##  ##  ##  ##  ##  ##  ##  ##  ##  ##  ##  ##  ##  ##  ##  ##  ##  ##  ##  ##  ##  ##  ##  ##  ##  ##  ##  ##  ##  ##  ##  ##  ##  ##  ##  ##  ##  ##  ##  ##  ##  ##  ##  ##  ##  ##  ##  ##  ##  ##  ##  ##  ##  ##  ##  1  3  2  3  --  2  3  ##  ##  ##  ##  ##  ##  ##  ##  ##  ##  ##  ##  ##  ##  ##  ##  ##  ##  ##  ##  ##  ##  ##  ##  ##  ##  ##  ##  ##  ##  ##  ##  ##  ##  ##  ##  ##  ##  ##  ##  ## [2920_8]61	: ##  ##  ##  ##  ##  ##  ##  ##  ##  ##  ##  ##  ##  ##  ##  ##  ##  ##  ##  ##  ##  ##  ##  ##  ##  ##  ##  ##  ##  ##  ##  ##  ##  ##  ##  ##  ##  ##  ##  ##  ##  ##  ##  ##  ##  ##  ##  ##  ##  ##  ##  ##  ##  ##  ##  1  3  2  3  2  --  2  ##  ##  ##  ##  ##  ##  ##  ##  ##  ##  ##  ##  ##  ##  ##  ##  ##  ##  ##  ##  ##  ##  ##  ##  ##  ##  ##  ##  ##  ##  ##  ##  ##  ##  ##  ##  ##  ##  ##  ##  ## [2476_8]62	: ##  ##  ##  ##  ##  ##  ##  ##  ##  ##  ##  ##  ##  ##  ##  ##  ##  ##  ##  ##  ##  ##  ##  ##  ##  ##  ##  ##  ##  ##  ##  ##  ##  ##  ##  ##  ##  ##  ##  ##  ##  ##  ##  ##  ##  ##  ##  ##  ##  ##  ##  ##  ##  ##  ##  2  2  1  2  3  2  --  ##  ##  ##  ##  ##  ##  ##  ##  ##  ##  ##  ##  ##  ##  ##  ##  ##  ##  ##  ##  ##  ##  ##  ##  ##  ##  ##  ##  ##  ##  ##  ##  ##  ##  ##  ##  ##  ##  ##  ##  ## [862_9]63	: ##  ##  ##  ##  ##  ##  ##  ##  ##  ##  ##  ##  ##  ##  ##  ##  ##  ##  ##  ##  ##  ##  ##  ##  ##  ##  ##  ##  ##  ##  ##  ##  ##  ##  ##  ##  ##  ##  ##  ##  ##  ##  ##  ##  ##  ##  ##  ##  ##  ##  ##  ##  ##  ##  ##  ##  ##  ##  ##  ##  ##  ##  --  ##  ##  ##  ##  ##  ##  ##  ##  ##  ##  ##  ##  ##  ##  ##  ##  ##  ##  ##  ##  ##  ##  ##  ##  ##  ##  ##  ##  ##  ##  ##  ##  ##  ##  ##  ##  ##  ##  ##  ## [2031_10]64	: ##  ##  ##  ##  ##  ##  ##  ##  ##  ##  ##  ##  ##  ##  ##  ##  ##  ##  ##  ##  ##  ##  ##  ##  ##  ##  ##  ##  ##  ##  ##  ##  ##  ##  ##  ##  ##  ##  ##  ##  ##  ##  ##  ##  ##  ##  ##  ##  ##  ##  ##  ##  ##  ##  ##  ##  ##  ##  ##  ##  ##  ##  ##  --  1  ##  ##  ##  ##  ##  ##  ##  ##  ##  ##  ##  ##  ##  ##  ##  ##  ##  ##  ##  ##  ##  ##  ##  ##  ##  ##  ##  ##  ##  ##  ##  ##  ##  ##  ##  ##  ##  ## [2026_10]65	: ##  ##  ##  ##  ##  ##  ##  ##  ##  ##  ##  ##  ##  ##  ##  ##  ##  ##  ##  ##  ##  ##  ##  ##  ##  ##  ##  ##  ##  ##  ##  ##  ##  ##  ##  ##  ##  ##  ##  ##  ##  ##  ##  ##  ##  ##  ##  ##  ##  ##  ##  ##  ##  ##  ##  ##  ##  ##  ##  ##  ##  ##  ##  1  --  ##  ##  ##  ##  ##  ##  ##  ##  ##  ##  ##  ##  ##  ##  ##  ##  ##  ##  ##  ##  ##  ##  ##  ##  ##  ##  ##  ##  ##  ##  ##  ##  ##  ##  ##  ##  ##  ## [2786_11]66	: ##  ##  ##  ##  ##  ##  ##  ##  ##  ##  ##  ##  ##  ##  ##  ##  ##  ##  ##  ##  ##  ##  ##  ##  ##  ##  ##  ##  ##  ##  ##  ##  ##  ##  ##  ##  ##  ##  ##  ##  ##  ##  ##  ##  ##  ##  ##  ##  ##  ##  ##  ##  ##  ##  ##  ##  ##  ##  ##  ##  ##  ##  ##  ##  ##  --  1  ##  ##  ##  ##  ##  ##  ##  ##  ##  ##  ##  ##  ##  ##  ##  ##  ##  ##  ##  ##  ##  ##  ##  ##  ##  ##  ##  ##  ##  ##  ##  ##  ##  ##  ##  ## [2323_11]67	: ##  ##  ##  ##  ##  ##  ##  ##  ##  ##  ##  ##  ##  ##  ##  ##  ##  ##  ##  ##  ##  ##  ##  ##  ##  ##  ##  ##  ##  ##  ##  ##  ##  ##  ##  ##  ##  ##  ##  ##  ##  ##  ##  ##  ##  ##  ##  ##  ##  ##  ##  ##  ##  ##  ##  ##  ##  ##  ##  ##  ##  ##  ##  ##  ##  1  --  ##  ##  ##  ##  ##  ##  ##  ##  ##  ##  ##  ##  ##  ##  ##  ##  ##  ##  ##  ##  ##  ##  ##  ##  ##  ##  ##  ##  ##  ##  ##  ##  ##  ##  ##  ## [2196_12]68	: ##  ##  ##  ##  ##  ##  ##  ##  ##  ##  ##  ##  ##  ##  ##  ##  ##  ##  ##  ##  ##  ##  ##  ##  ##  ##  ##  ##  ##  ##  ##  ##  ##  ##  ##  ##  ##  ##  ##  ##  ##  ##  ##  ##  ##  ##  ##  ##  ##  ##  ##  ##  ##  ##  ##  ##  ##  ##  ##  ##  ##  ##  ##  ##  ##  ##  ##  --  1  1  1  1  2  2  2  2  1  ##  ##  ##  ##  ##  ##  ##  ##  ##  ##  ##  ##  ##  ##  ##  ##  ##  ##  ##  ##  ##  ##  ##  ##  ##  ## [2806_12]69	: ##  ##  ##  ##  ##  ##  ##  ##  ##  ##  ##  ##  ##  ##  ##  ##  ##  ##  ##  ##  ##  ##  ##  ##  ##  ##  ##  ##  ##  ##  ##  ##  ##  ##  ##  ##  ##  ##  ##  ##  ##  ##  ##  ##  ##  ##  ##  ##  ##  ##  ##  ##  ##  ##  ##  ##  ##  ##  ##  ##  ##  ##  ##  ##  ##  ##  ##  1  --  2  2  2  1  5  3  3  2  ##  ##  ##  ##  ##  ##  ##  ##  ##  ##  ##  ##  ##  ##  ##  ##  ##  ##  ##  ##  ##  ##  ##  ##  ##  ## [2194_12]70	: ##  ##  ##  ##  ##  ##  ##  ##  ##  ##  ##  ##  ##  ##  ##  ##  ##  ##  ##  ##  ##  ##  ##  ##  ##  ##  ##  ##  ##  ##  ##  ##  ##  ##  ##  ##  ##  ##  ##  ##  ##  ##  ##  ##  ##  ##  ##  ##  ##  ##  ##  ##  ##  ##  ##  ##  ##  ##  ##  ##  ##  ##  ##  ##  ##  ##  ##  1  2  --  2  2  3  3  1  3  2  ##  ##  ##  ##  ##  ##  ##  ##  ##  ##  ##  ##  ##  ##  ##  ##  ##  ##  ##  ##  ##  ##  ##  ##  ##  ## [2201_12]71	: ##  ##  ##  ##  ##  ##  ##  ##  ##  ##  ##  ##  ##  ##  ##  ##  ##  ##  ##  ##  ##  ##  ##  ##  ##  ##  ##  ##  ##  ##  ##  ##  ##  ##  ##  ##  ##  ##  ##  ##  ##  ##  ##  ##  ##  ##  ##  ##  ##  ##  ##  ##  ##  ##  ##  ##  ##  ##  ##  ##  ##  ##  ##  ##  ##  ##  ##  1  2  2  --  2  3  3  3  1  2  ##  ##  ##  ##  ##  ##  ##  ##  ##  ##  ##  ##  ##  ##  ##  ##  ##  ##  ##  ##  ##  ##  ##  ##  ##  ## [2202_12]72	: ##  ##  ##  ##  ##  ##  ##  ##  ##  ##  ##  ##  ##  ##  ##  ##  ##  ##  ##  ##  ##  ##  ##  ##  ##  ##  ##  ##  ##  ##  ##  ##  ##  ##  ##  ##  ##  ##  ##  ##  ##  ##  ##  ##  ##  ##  ##  ##  ##  ##  ##  ##  ##  ##  ##  ##  ##  ##  ##  ##  ##  ##  ##  ##  ##  ##  ##  1  2  2  2  --  3  3  3  3  2  ##  ##  ##  ##  ##  ##  ##  ##  ##  ##  ##  ##  ##  ##  ##  ##  ##  ##  ##  ##  ##  ##  ##  ##  ##  ## [2171_12]73	: ##  ##  ##  ##  ##  ##  ##  ##  ##  ##  ##  ##  ##  ##  ##  ##  ##  ##  ##  ##  ##  ##  ##  ##  ##  ##  ##  ##  ##  ##  ##  ##  ##  ##  ##  ##  ##  ##  ##  ##  ##  ##  ##  ##  ##  ##  ##  ##  ##  ##  ##  ##  ##  ##  ##  ##  ##  ##  ##  ##  ##  ##  ##  ##  ##  ##  ##  2  1  3  3  3  --  6  4  4  3  ##  ##  ##  ##  ##  ##  ##  ##  ##  ##  ##  ##  ##  ##  ##  ##  ##  ##  ##  ##  ##  ##  ##  ##  ##  ## [2225_12]74	: ##  ##  ##  ##  ##  ##  ##  ##  ##  ##  ##  ##  ##  ##  ##  ##  ##  ##  ##  ##  ##  ##  ##  ##  ##  ##  ##  ##  ##  ##  ##  ##  ##  ##  ##  ##  ##  ##  ##  ##  ##  ##  ##  ##  ##  ##  ##  ##  ##  ##  ##  ##  ##  ##  ##  ##  ##  ##  ##  ##  ##  ##  ##  ##  ##  ##  ##  2  5  3  3  3  6  --  2  4  2  ##  ##  ##  ##  ##  ##  ##  ##  ##  ##  ##  ##  ##  ##  ##  ##  ##  ##  ##  ##  ##  ##  ##  ##  ##  ## [2224_12]75	: ##  ##  ##  ##  ##  ##  ##  ##  ##  ##  ##  ##  ##  ##  ##  ##  ##  ##  ##  ##  ##  ##  ##  ##  ##  ##  ##  ##  ##  ##  ##  ##  ##  ##  ##  ##  ##  ##  ##  ##  ##  ##  ##  ##  ##  ##  ##  ##  ##  ##  ##  ##  ##  ##  ##  ##  ##  ##  ##  ##  ##  ##  ##  ##  ##  ##  ##  2  3  1  3  3  4  2  --  4  3  ##  ##  ##  ##  ##  ##  ##  ##  ##  ##  ##  ##  ##  ##  ##  ##  ##  ##  ##  ##  ##  ##  ##  ##  ##  ## [2198_12]76	: ##  ##  ##  ##  ##  ##  ##  ##  ##  ##  ##  ##  ##  ##  ##  ##  ##  ##  ##  ##  ##  ##  ##  ##  ##  ##  ##  ##  ##  ##  ##  ##  ##  ##  ##  ##  ##  ##  ##  ##  ##  ##  ##  ##  ##  ##  ##  ##  ##  ##  ##  ##  ##  ##  ##  ##  ##  ##  ##  ##  ##  ##  ##  ##  ##  ##  ##  2  3  3  1  3  4  4  4  --  3  ##  ##  ##  ##  ##  ##  ##  ##  ##  ##  ##  ##  ##  ##  ##  ##  ##  ##  ##  ##  ##  ##  ##  ##  ##  ## [1312_12]77	: ##  ##  ##  ##  ##  ##  ##  ##  ##  ##  ##  ##  ##  ##  ##  ##  ##  ##  ##  ##  ##  ##  ##  ##  ##  ##  ##  ##  ##  ##  ##  ##  ##  ##  ##  ##  ##  ##  ##  ##  ##  ##  ##  ##  ##  ##  ##  ##  ##  ##  ##  ##  ##  ##  ##  ##  ##  ##  ##  ##  ##  ##  ##  ##  ##  ##  ##  1  2  2  2  2  3  2  3  3  --  ##  ##  ##  ##  ##  ##  ##  ##  ##  ##  ##  ##  ##  ##  ##  ##  ##  ##  ##  ##  ##  ##  ##  ##  ##  ## [1923_13]78	: ##  ##  ##  ##  ##  ##  ##  ##  ##  ##  ##  ##  ##  ##  ##  ##  ##  ##  ##  ##  ##  ##  ##  ##  ##  ##  ##  ##  ##  ##  ##  ##  ##  ##  ##  ##  ##  ##  ##  ##  ##  ##  ##  ##  ##  ##  ##  ##  ##  ##  ##  ##  ##  ##  ##  ##  ##  ##  ##  ##  ##  ##  ##  ##  ##  ##  ##  ##  ##  ##  ##  ##  ##  ##  ##  ##  ##  --  1  1  ##  ##  ##  ##  ##  ##  ##  ##  ##  ##  ##  ##  ##  ##  ##  ##  ##  ##  ##  ##  ##  ##  ## [1956_13]79	: ##  ##  ##  ##  ##  ##  ##  ##  ##  ##  ##  ##  ##  ##  ##  ##  ##  ##  ##  ##  ##  ##  ##  ##  ##  ##  ##  ##  ##  ##  ##  ##  ##  ##  ##  ##  ##  ##  ##  ##  ##  ##  ##  ##  ##  ##  ##  ##  ##  ##  ##  ##  ##  ##  ##  ##  ##  ##  ##  ##  ##  ##  ##  ##  ##  ##  ##  ##  ##  ##  ##  ##  ##  ##  ##  ##  ##  1  --  2  ##  ##  ##  ##  ##  ##  ##  ##  ##  ##  ##  ##  ##  ##  ##  ##  ##  ##  ##  ##  ##  ##  ## [2028_13]80	: ##  ##  ##  ##  ##  ##  ##  ##  ##  ##  ##  ##  ##  ##  ##  ##  ##  ##  ##  ##  ##  ##  ##  ##  ##  ##  ##  ##  ##  ##  ##  ##  ##  ##  ##  ##  ##  ##  ##  ##  ##  ##  ##  ##  ##  ##  ##  ##  ##  ##  ##  ##  ##  ##  ##  ##  ##  ##  ##  ##  ##  ##  ##  ##  ##  ##  ##  ##  ##  ##  ##  ##  ##  ##  ##  ##  ##  1  2  --  ##  ##  ##  ##  ##  ##  ##  ##  ##  ##  ##  ##  ##  ##  ##  ##  ##  ##  ##  ##  ##  ##  ## [2044_14]81	: ##  ##  ##  ##  ##  ##  ##  ##  ##  ##  ##  ##  ##  ##  ##  ##  ##  ##  ##  ##  ##  ##  ##  ##  ##  ##  ##  ##  ##  ##  ##  ##  ##  ##  ##  ##  ##  ##  ##  ##  ##  ##  ##  ##  ##  ##  ##  ##  ##  ##  ##  ##  ##  ##  ##  ##  ##  ##  ##  ##  ##  ##  ##  ##  ##  ##  ##  ##  ##  ##  ##  ##  ##  ##  ##  ##  ##  ##  ##  ##  --  3  4  4  4  5  ##  ##  ##  ##  ##  ##  ##  ##  ##  ##  ##  ##  ##  ##  ##  ##  ## [2042_14]82	: ##  ##  ##  ##  ##  ##  ##  ##  ##  ##  ##  ##  ##  ##  ##  ##  ##  ##  ##  ##  ##  ##  ##  ##  ##  ##  ##  ##  ##  ##  ##  ##  ##  ##  ##  ##  ##  ##  ##  ##  ##  ##  ##  ##  ##  ##  ##  ##  ##  ##  ##  ##  ##  ##  ##  ##  ##  ##  ##  ##  ##  ##  ##  ##  ##  ##  ##  ##  ##  ##  ##  ##  ##  ##  ##  ##  ##  ##  ##  ##  3  --  1  2  1  2  ##  ##  ##  ##  ##  ##  ##  ##  ##  ##  ##  ##  ##  ##  ##  ##  ## [2850_14]83	: ##  ##  ##  ##  ##  ##  ##  ##  ##  ##  ##  ##  ##  ##  ##  ##  ##  ##  ##  ##  ##  ##  ##  ##  ##  ##  ##  ##  ##  ##  ##  ##  ##  ##  ##  ##  ##  ##  ##  ##  ##  ##  ##  ##  ##  ##  ##  ##  ##  ##  ##  ##  ##  ##  ##  ##  ##  ##  ##  ##  ##  ##  ##  ##  ##  ##  ##  ##  ##  ##  ##  ##  ##  ##  ##  ##  ##  ##  ##  ##  4  1  --  3  2  1  ##  ##  ##  ##  ##  ##  ##  ##  ##  ##  ##  ##  ##  ##  ##  ##  ## [2848_14]84	: ##  ##  ##  ##  ##  ##  ##  ##  ##  ##  ##  ##  ##  ##  ##  ##  ##  ##  ##  ##  ##  ##  ##  ##  ##  ##  ##  ##  ##  ##  ##  ##  ##  ##  ##  ##  ##  ##  ##  ##  ##  ##  ##  ##  ##  ##  ##  ##  ##  ##  ##  ##  ##  ##  ##  ##  ##  ##  ##  ##  ##  ##  ##  ##  ##  ##  ##  ##  ##  ##  ##  ##  ##  ##  ##  ##  ##  ##  ##  ##  4  2  3  --  3  4  ##  ##  ##  ##  ##  ##  ##  ##  ##  ##  ##  ##  ##  ##  ##  ##  ## [2040_14]85	: ##  ##  ##  ##  ##  ##  ##  ##  ##  ##  ##  ##  ##  ##  ##  ##  ##  ##  ##  ##  ##  ##  ##  ##  ##  ##  ##  ##  ##  ##  ##  ##  ##  ##  ##  ##  ##  ##  ##  ##  ##  ##  ##  ##  ##  ##  ##  ##  ##  ##  ##  ##  ##  ##  ##  ##  ##  ##  ##  ##  ##  ##  ##  ##  ##  ##  ##  ##  ##  ##  ##  ##  ##  ##  ##  ##  ##  ##  ##  ##  4  1  2  3  --  1  ##  ##  ##  ##  ##  ##  ##  ##  ##  ##  ##  ##  ##  ##  ##  ##  ## [2853_14]86	: ##  ##  ##  ##  ##  ##  ##  ##  ##  ##  ##  ##  ##  ##  ##  ##  ##  ##  ##  ##  ##  ##  ##  ##  ##  ##  ##  ##  ##  ##  ##  ##  ##  ##  ##  ##  ##  ##  ##  ##  ##  ##  ##  ##  ##  ##  ##  ##  ##  ##  ##  ##  ##  ##  ##  ##  ##  ##  ##  ##  ##  ##  ##  ##  ##  ##  ##  ##  ##  ##  ##  ##  ##  ##  ##  ##  ##  ##  ##  ##  5  2  1  4  1  --  ##  ##  ##  ##  ##  ##  ##  ##  ##  ##  ##  ##  ##  ##  ##  ##  ## [2004_15]87	: ##  ##  ##  ##  ##  ##  ##  ##  ##  ##  ##  ##  ##  ##  ##  ##  ##  ##  ##  ##  ##  ##  ##  ##  ##  ##  ##  ##  ##  ##  ##  ##  ##  ##  ##  ##  ##  ##  ##  ##  ##  ##  ##  ##  ##  ##  ##  ##  ##  ##  ##  ##  ##  ##  ##  ##  ##  ##  ##  ##  ##  ##  ##  ##  ##  ##  ##  ##  ##  ##  ##  ##  ##  ##  ##  ##  ##  ##  ##  ##  ##  ##  ##  ##  ##  ##  --  ##  ##  ##  ##  ##  ##  ##  ##  ##  ##  ##  ##  ##  ##  ##  ## [2267_16]88	: ##  ##  ##  ##  ##  ##  ##  ##  ##  ##  ##  ##  ##  ##  ##  ##  ##  ##  ##  ##  ##  ##  ##  ##  ##  ##  ##  ##  ##  ##  ##  ##  ##  ##  ##  9  8  10  7  ##  ##  ##  ##  ##  ##  ##  ##  ##  ##  ##  ##  ##  ##  ##  ##  ##  ##  ##  ##  ##  ##  ##  ##  ##  ##  ##  ##  ##  ##  ##  ##  ##  ##  ##  ##  ##  ##  ##  ##  ##  ##  ##  ##  ##  ##  ##  ##  --  2  3  3  ##  ##  ##  ##  ##  ##  ##  ##  ##  ##  ##  ## [2268_16]89	: ##  ##  ##  ##  ##  ##  ##  ##  ##  ##  ##  ##  ##  ##  ##  ##  ##  ##  ##  ##  ##  ##  ##  ##  ##  ##  ##  ##  ##  ##  ##  ##  ##  ##  ##  10  9  11  9  ##  ##  ##  ##  ##  ##  ##  ##  ##  ##  ##  ##  ##  ##  ##  ##  ##  ##  ##  ##  ##  ##  ##  ##  ##  ##  ##  ##  ##  ##  ##  ##  ##  ##  ##  ##  ##  ##  ##  ##  ##  ##  ##  ##  ##  ##  ##  ##  2  --  1  1  ##  ##  ##  ##  ##  ##  ##  ##  ##  ##  ##  ## [2469_16]90	: ##  ##  ##  ##  ##  ##  ##  ##  ##  ##  ##  ##  ##  ##  ##  ##  ##  ##  ##  ##  ##  ##  ##  ##  ##  ##  ##  ##  ##  ##  ##  ##  ##  ##  ##  11  10  12  10  ##  ##  ##  ##  ##  ##  ##  ##  ##  ##  ##  ##  ##  ##  ##  ##  ##  ##  ##  ##  ##  ##  ##  ##  ##  ##  ##  ##  ##  ##  ##  ##  ##  ##  ##  ##  ##  ##  ##  ##  ##  ##  ##  ##  ##  ##  ##  ##  3  1  --  2  ##  ##  ##  ##  ##  ##  ##  ##  ##  ##  ##  ## [2269_16]91	: ##  ##  ##  ##  ##  ##  ##  ##  ##  ##  ##  ##  ##  ##  ##  ##  ##  ##  ##  ##  ##  ##  ##  ##  ##  ##  ##  ##  ##  ##  ##  ##  ##  ##  ##  11  10  12  10  ##  ##  ##  ##  ##  ##  ##  ##  ##  ##  ##  ##  ##  ##  ##  ##  ##  ##  ##  ##  ##  ##  ##  ##  ##  ##  ##  ##  ##  ##  ##  ##  ##  ##  ##  ##  ##  ##  ##  ##  ##  ##  ##  ##  ##  ##  ##  ##  3  1  2  --  ##  ##  ##  ##  ##  ##  ##  ##  ##  ##  ##  ## [2274_17]92	: ##  ##  ##  ##  ##  ##  ##  ##  ##  ##  ##  ##  ##  ##  ##  ##  ##  ##  ##  ##  ##  ##  ##  ##  ##  ##  ##  ##  ##  ##  ##  ##  ##  ##  ##  ##  ##  ##  ##  ##  ##  ##  ##  ##  ##  ##  ##  ##  ##  ##  ##  ##  ##  ##  ##  ##  ##  ##  ##  ##  ##  ##  ##  ##  ##  ##  ##  ##  ##  ##  ##  ##  ##  ##  ##  ##  ##  ##  ##  ##  ##  ##  ##  ##  ##  ##  ##  ##  ##  ##  ##  --  ##  ##  ##  ##  ##  ##  ##  ##  ##  ##  ## [2313_18]93	: ##  ##  ##  ##  ##  ##  ##  ##  ##  ##  ##  ##  ##  ##  ##  ##  ##  ##  ##  ##  ##  ##  ##  ##  ##  ##  ##  ##  ##  ##  ##  ##  ##  ##  ##  ##  ##  ##  ##  ##  ##  ##  ##  ##  ##  ##  ##  ##  ##  ##  ##  ##  ##  ##  ##  ##  ##  ##  ##  ##  ##  ##  ##  ##  ##  ##  ##  ##  ##  ##  ##  ##  ##  ##  ##  ##  ##  ##  ##  ##  ##  ##  ##  ##  ##  ##  ##  ##  ##  ##  ##  ##  --  ##  ##  ##  ##  ##  ##  ##  ##  ##  ## [2278_19]94	: ##  ##  ##  ##  ##  ##  ##  ##  ##  ##  ##  ##  ##  ##  ##  ##  ##  ##  ##  ##  ##  ##  ##  ##  ##  ##  ##  ##  ##  ##  ##  ##  ##  ##  ##  ##  ##  ##  ##  ##  ##  ##  ##  ##  ##  ##  ##  ##  ##  ##  ##  ##  ##  ##  ##  ##  ##  ##  ##  ##  ##  ##  ##  ##  ##  ##  ##  ##  ##  ##  ##  ##  ##  ##  ##  ##  ##  ##  ##  ##  ##  ##  ##  ##  ##  ##  ##  ##  ##  ##  ##  ##  ##  --  ##  ##  ##  ##  ##  ##  ##  ##  ## [2302_20]95	: ##  ##  ##  ##  ##  ##  ##  ##  ##  ##  ##  ##  ##  ##  ##  ##  ##  ##  ##  ##  ##  ##  ##  ##  ##  ##  ##  ##  ##  ##  ##  ##  ##  ##  ##  ##  ##  ##  ##  ##  ##  ##  ##  ##  ##  ##  ##  ##  ##  ##  ##  ##  ##  ##  ##  ##  ##  ##  ##  ##  ##  ##  ##  ##  ##  ##  ##  ##  ##  ##  ##  ##  ##  ##  ##  ##  ##  ##  ##  ##  ##  ##  ##  ##  ##  ##  ##  ##  ##  ##  ##  ##  ##  ##  --  ##  ##  ##  ##  ##  ##  ##  ## [2342_21]96	: ##  ##  ##  ##  ##  ##  ##  ##  ##  ##  ##  ##  ##  ##  ##  ##  ##  ##  ##  ##  ##  ##  ##  ##  ##  ##  ##  ##  ##  ##  ##  ##  ##  ##  ##  ##  ##  ##  ##  ##  ##  ##  ##  ##  ##  ##  ##  ##  ##  ##  ##  ##  ##  ##  ##  ##  ##  ##  ##  ##  ##  ##  ##  ##  ##  ##  ##  ##  ##  ##  ##  ##  ##  ##  ##  ##  ##  ##  ##  ##  ##  ##  ##  ##  ##  ##  ##  ##  ##  ##  ##  ##  ##  ##  ##  --  ##  ##  ##  ##  ##  ##  ## [2277_22]97	: ##  ##  ##  ##  ##  ##  ##  ##  ##  ##  ##  ##  ##  ##  ##  ##  ##  ##  ##  ##  ##  ##  ##  ##  ##  ##  ##  ##  ##  ##  ##  ##  ##  ##  ##  ##  ##  ##  ##  ##  ##  ##  ##  ##  ##  ##  ##  ##  ##  ##  ##  ##  ##  ##  ##  ##  ##  ##  ##  ##  ##  ##  ##  ##  ##  ##  ##  ##  ##  ##  ##  ##  ##  ##  ##  ##  ##  ##  ##  ##  ##  ##  ##  ##  ##  ##  ##  ##  ##  ##  ##  ##  ##  ##  ##  ##  --  ##  ##  ##  ##  ##  ## [2281_23]98	: ##  ##  ##  ##  ##  ##  ##  ##  ##  ##  ##  ##  ##  ##  ##  ##  ##  ##  ##  ##  ##  ##  ##  ##  ##  ##  ##  ##  ##  ##  ##  ##  ##  ##  ##  ##  ##  ##  ##  ##  ##  ##  ##  ##  ##  ##  ##  ##  ##  ##  ##  ##  ##  ##  ##  ##  ##  ##  ##  ##  ##  ##  ##  ##  ##  ##  ##  ##  ##  ##  ##  ##  ##  ##  ##  ##  ##  ##  ##  ##  ##  ##  ##  ##  ##  ##  ##  ##  ##  ##  ##  ##  ##  ##  ##  ##  ##  --  ##  ##  ##  ##  ## [2866_24]99	: ##  ##  ##  ##  ##  ##  ##  ##  ##  ##  ##  ##  ##  ##  ##  ##  ##  ##  ##  ##  ##  ##  ##  ##  ##  ##  ##  ##  ##  ##  ##  ##  ##  ##  ##  ##  ##  ##  ##  ##  ##  ##  ##  ##  ##  ##  ##  ##  ##  ##  ##  ##  ##  ##  ##  ##  ##  ##  ##  ##  ##  ##  ##  ##  ##  ##  ##  ##  ##  ##  ##  ##  ##  ##  ##  ##  ##  ##  ##  ##  ##  ##  ##  ##  ##  ##  ##  ##  ##  ##  ##  ##  ##  ##  ##  ##  ##  ##  --  1  ##  ##  ## [2865_24]100	: ##  ##  ##  ##  ##  ##  ##  ##  ##  ##  ##  ##  ##  ##  ##  ##  ##  ##  ##  ##  ##  ##  ##  ##  ##  ##  ##  ##  ##  ##  ##  ##  ##  ##  ##  ##  ##  ##  ##  ##  ##  ##  ##  ##  ##  ##  ##  ##  ##  ##  ##  ##  ##  ##  ##  ##  ##  ##  ##  ##  ##  ##  ##  ##  ##  ##  ##  ##  ##  ##  ##  ##  ##  ##  ##  ##  ##  ##  ##  ##  ##  ##  ##  ##  ##  ##  ##  ##  ##  ##  ##  ##  ##  ##  ##  ##  ##  ##  1  --  ##  ##  ## [2801_25]101	: ##  ##  ##  ##  ##  ##  ##  ##  ##  ##  ##  ##  ##  ##  ##  ##  ##  ##  ##  ##  ##  ##  ##  ##  ##  ##  ##  ##  ##  ##  ##  ##  ##  ##  ##  ##  ##  ##  ##  ##  ##  ##  ##  ##  ##  ##  ##  ##  ##  ##  ##  ##  ##  ##  ##  ##  ##  ##  ##  ##  ##  ##  ##  ##  ##  ##  ##  ##  ##  ##  ##  ##  ##  ##  ##  ##  ##  ##  ##  ##  ##  ##  ##  ##  ##  ##  ##  ##  ##  ##  ##  ##  ##  ##  ##  ##  ##  ##  ##  ##  --  ##  ## [2805_26]102	: ##  ##  ##  ##  ##  ##  ##  ##  ##  ##  ##  ##  ##  ##  ##  ##  ##  ##  ##  ##  ##  ##  ##  ##  ##  ##  ##  ##  ##  ##  ##  ##  ##  ##  3  ##  ##  ##  ##  ##  ##  ##  ##  ##  ##  ##  ##  ##  ##  ##  ##  ##  ##  ##  ##  ##  ##  ##  ##  ##  ##  ##  ##  ##  ##  ##  ##  ##  ##  ##  ##  ##  ##  ##  ##  ##  ##  ##  ##  ##  ##  ##  ##  ##  ##  ##  ##  ##  ##  ##  ##  ##  ##  ##  ##  ##  ##  ##  ##  ##  ##  --  8 [2800_27]103	: ##  ##  ##  ##  ##  ##  ##  ##  ##  ##  ##  ##  ##  ##  ##  ##  ##  ##  ##  ##  ##  ##  ##  ##  ##  ##  ##  ##  ##  ##  ##  ##  ##  ##  7  ##  ##  ##  ##  ##  ##  ##  ##  ##  ##  ##  ##  ##  ##  ##  ##  ##  ##  ##  ##  ##  ##  ##  ##  ##  ##  ##  ##  ##  ##  ##  ##  ##  ##  ##  ##  ##  ##  ##  ##  ##  ##  ##  ##  ##  ##  ##  ##  ##  ##  ##  ##  ##  ##  ##  ##  ##  ##  ##  ##  ##  ##  ##  ##  ##  ##  8  -- COMPUTED FROM THE NETWORK DISTANCE MATRIXpos:   			  1  2  3  4  5  6  7  8  9  10  11  12  13  14  15  16  17  18  19  20  21  22  23  24  25  26  27  28  29  30  31  32  33  34  35  36  37  38  39  40  41  42  43  44  45  46  47  48  49  50  51  52  53  54  55  56  57  58  59  60  61  62  63  64  65  66  67  68  69  70  71  72  73  74  75  76  77  78  79  80  81  82  83  84  85  86  87  88  89  90  91  92  93  94  95  96  97  98  99  100  101  102  103 [2440_1]1 	: --  1  2  3  5  4  4  2  2  3  3  3  2  5  1  1  2  3  3  ##  ##  ##  ##  ##  ##  ##  ##  ##  ##  ##  ##  ##  ##  ##  ##  ##  ##  ##  ##  ##  ##  ##  ##  ##  ##  ##  ##  ##  ##  ##  ##  ##  ##  ##  ##  ##  ##  ##  ##  ##  ##  ##  ##  ##  ##  ##  ##  ##  ##  ##  ##  ##  ##  ##  ##  ##  ##  ##  ##  ##  ##  ##  ##  ##  ##  ##  ##  ##  ##  ##  ##  ##  ##  ##  ##  ##  ##  ##  ##  ##  ##  ##  ## [1945_1]2 	: 1  --  1  2  4  3  3  1  1  2  2  2  3  4  2  2  3  2  2  ##  ##  ##  ##  ##  ##  ##  ##  ##  ##  ##  ##  ##  ##  ##  ##  ##  ##  ##  ##  ##  ##  ##  ##  ##  ##  ##  ##  ##  ##  ##  ##  ##  ##  ##  ##  ##  ##  ##  ##  ##  ##  ##  ##  ##  ##  ##  ##  ##  ##  ##  ##  ##  ##  ##  ##  ##  ##  ##  ##  ##  ##  ##  ##  ##  ##  ##  ##  ##  ##  ##  ##  ##  ##  ##  ##  ##  ##  ##  ##  ##  ##  ##  ## [836_1]3 	: 2  1  --  3  3  2  4  2  2  1  1  2  3  3  3  3  4  1  1  ##  ##  ##  ##  ##  ##  ##  ##  ##  ##  ##  ##  ##  ##  ##  ##  ##  ##  ##  ##  ##  ##  ##  ##  ##  ##  ##  ##  ##  ##  ##  ##  ##  ##  ##  ##  ##  ##  ##  ##  ##  ##  ##  ##  ##  ##  ##  ##  ##  ##  ##  ##  ##  ##  ##  ##  ##  ##  ##  ##  ##  ##  ##  ##  ##  ##  ##  ##  ##  ##  ##  ##  ##  ##  ##  ##  ##  ##  ##  ##  ##  ##  ##  ## [2219_1]4 	: 3  2  3  --  2  1  1  3  3  4  4  4  5  2  4  4  5  4  4  ##  ##  ##  ##  ##  ##  ##  ##  ##  ##  ##  ##  ##  ##  ##  ##  ##  ##  ##  ##  ##  ##  ##  ##  ##  ##  ##  ##  ##  ##  ##  ##  ##  ##  ##  ##  ##  ##  ##  ##  ##  ##  ##  ##  ##  ##  ##  ##  ##  ##  ##  ##  ##  ##  ##  ##  ##  ##  ##  ##  ##  ##  ##  ##  ##  ##  ##  ##  ##  ##  ##  ##  ##  ##  ##  ##  ##  ##  ##  ##  ##  ##  ##  ## [1339_1]5 	: 5  4  3  2  --  1  1  5  3  2  4  5  6  2  6  6  7  4  4  ##  ##  ##  ##  ##  ##  ##  ##  ##  ##  ##  ##  ##  ##  ##  ##  ##  ##  ##  ##  ##  ##  ##  ##  ##  ##  ##  ##  ##  ##  ##  ##  ##  ##  ##  ##  ##  ##  ##  ##  ##  ##  ##  ##  ##  ##  ##  ##  ##  ##  ##  ##  ##  ##  ##  ##  ##  ##  ##  ##  ##  ##  ##  ##  ##  ##  ##  ##  ##  ##  ##  ##  ##  ##  ##  ##  ##  ##  ##  ##  ##  ##  ##  ## [1939_1]6 	: 4  3  2  1  1  --  2  4  4  3  3  4  5  1  5  5  6  3  3  ##  ##  ##  ##  ##  ##  ##  ##  ##  ##  ##  ##  ##  ##  ##  ##  ##  ##  ##  ##  ##  ##  ##  ##  ##  ##  ##  ##  ##  ##  ##  ##  ##  ##  ##  ##  ##  ##  ##  ##  ##  ##  ##  ##  ##  ##  ##  ##  ##  ##  ##  ##  ##  ##  ##  ##  ##  ##  ##  ##  ##  ##  ##  ##  ##  ##  ##  ##  ##  ##  ##  ##  ##  ##  ##  ##  ##  ##  ##  ##  ##  ##  ##  ## [2794_1]7 	: 4  3  4  1  1  2  --  4  2  3  5  5  6  3  5  5  6  5  5  ##  ##  ##  ##  ##  ##  ##  ##  ##  ##  ##  ##  ##  ##  ##  ##  ##  ##  ##  ##  ##  ##  ##  ##  ##  ##  ##  ##  ##  ##  ##  ##  ##  ##  ##  ##  ##  ##  ##  ##  ##  ##  ##  ##  ##  ##  ##  ##  ##  ##  ##  ##  ##  ##  ##  ##  ##  ##  ##  ##  ##  ##  ##  ##  ##  ##  ##  ##  ##  ##  ##  ##  ##  ##  ##  ##  ##  ##  ##  ##  ##  ##  ##  ## [825_1]8 	: 2  1  2  3  5  4  4  --  2  3  3  3  4  5  3  3  4  3  3  ##  ##  ##  ##  ##  ##  ##  ##  ##  ##  ##  ##  ##  ##  ##  ##  ##  ##  ##  ##  ##  ##  ##  ##  ##  ##  ##  ##  ##  ##  ##  ##  ##  ##  ##  ##  ##  ##  ##  ##  ##  ##  ##  ##  ##  ##  ##  ##  ##  ##  ##  ##  ##  ##  ##  ##  ##  ##  ##  ##  ##  ##  ##  ##  ##  ##  ##  ##  ##  ##  ##  ##  ##  ##  ##  ##  ##  ##  ##  ##  ##  ##  ##  ## [2911_1]9 	: 2  1  2  3  3  4  2  2  --  1  3  3  4  5  3  3  4  3  3  ##  ##  ##  ##  ##  ##  ##  ##  ##  ##  ##  ##  ##  ##  ##  ##  ##  ##  ##  ##  ##  ##  ##  ##  ##  ##  ##  ##  ##  ##  ##  ##  ##  ##  ##  ##  ##  ##  ##  ##  ##  ##  ##  ##  ##  ##  ##  ##  ##  ##  ##  ##  ##  ##  ##  ##  ##  ##  ##  ##  ##  ##  ##  ##  ##  ##  ##  ##  ##  ##  ##  ##  ##  ##  ##  ##  ##  ##  ##  ##  ##  ##  ##  ## [1327_1]10	: 3  2  1  4  2  3  3  3  1  --  2  3  4  4  4  4  5  2  2  ##  ##  ##  ##  ##  ##  ##  ##  ##  ##  ##  ##  ##  ##  ##  ##  ##  ##  ##  ##  ##  ##  ##  ##  ##  ##  ##  ##  ##  ##  ##  ##  ##  ##  ##  ##  ##  ##  ##  ##  ##  ##  ##  ##  ##  ##  ##  ##  ##  ##  ##  ##  ##  ##  ##  ##  ##  ##  ##  ##  ##  ##  ##  ##  ##  ##  ##  ##  ##  ##  ##  ##  ##  ##  ##  ##  ##  ##  ##  ##  ##  ##  ##  ## [1332_1]11	: 3  2  1  4  4  3  5  3  3  2  --  3  4  4  4  4  5  2  2  ##  ##  ##  ##  ##  ##  ##  ##  ##  ##  ##  ##  ##  ##  ##  ##  ##  ##  ##  ##  ##  ##  ##  ##  ##  ##  ##  ##  ##  ##  ##  ##  ##  ##  ##  ##  ##  ##  ##  ##  ##  ##  ##  ##  ##  ##  ##  ##  ##  ##  ##  ##  ##  ##  ##  ##  ##  ##  ##  ##  ##  ##  ##  ##  ##  ##  ##  ##  ##  ##  ##  ##  ##  ##  ##  ##  ##  ##  ##  ##  ##  ##  ##  ## [2788_1]12	: 3  2  2  4  5  4  5  3  3  3  3  --  1  5  4  4  5  3  3  ##  ##  ##  ##  ##  ##  ##  ##  ##  ##  ##  ##  ##  ##  ##  ##  ##  ##  ##  ##  ##  ##  ##  ##  ##  ##  ##  ##  ##  ##  ##  ##  ##  ##  ##  ##  ##  ##  ##  ##  ##  ##  ##  ##  ##  ##  ##  ##  ##  ##  ##  ##  ##  ##  ##  ##  ##  ##  ##  ##  ##  ##  ##  ##  ##  ##  ##  ##  ##  ##  ##  ##  ##  ##  ##  ##  ##  ##  ##  ##  ##  ##  ##  ## [2796_1]13	: 2  3  3  5  6  5  6  4  4  4  4  1  --  6  3  3  4  4  4  ##  ##  ##  ##  ##  ##  ##  ##  ##  ##  ##  ##  ##  ##  ##  ##  ##  ##  ##  ##  ##  ##  ##  ##  ##  ##  ##  ##  ##  ##  ##  ##  ##  ##  ##  ##  ##  ##  ##  ##  ##  ##  ##  ##  ##  ##  ##  ##  ##  ##  ##  ##  ##  ##  ##  ##  ##  ##  ##  ##  ##  ##  ##  ##  ##  ##  ##  ##  ##  ##  ##  ##  ##  ##  ##  ##  ##  ##  ##  ##  ##  ##  ##  ## [2445_1]14	: 5  4  3  2  2  1  3  5  5  4  4  5  6  --  6  6  7  4  4  ##  ##  ##  ##  ##  ##  ##  ##  ##  ##  ##  ##  ##  ##  ##  ##  ##  ##  ##  ##  ##  ##  ##  ##  ##  ##  ##  ##  ##  ##  ##  ##  ##  ##  ##  ##  ##  ##  ##  ##  ##  ##  ##  ##  ##  ##  ##  ##  ##  ##  ##  ##  ##  ##  ##  ##  ##  ##  ##  ##  ##  ##  ##  ##  ##  ##  ##  ##  ##  ##  ##  ##  ##  ##  ##  ##  ##  ##  ##  ##  ##  ##  ##  ## [2871_1]15	: 1  2  3  4  6  5  5  3  3  4  4  4  3  6  --  2  1  4  4  ##  ##  ##  ##  ##  ##  ##  ##  ##  ##  ##  ##  ##  ##  ##  ##  ##  ##  ##  ##  ##  ##  ##  ##  ##  ##  ##  ##  ##  ##  ##  ##  ##  ##  ##  ##  ##  ##  ##  ##  ##  ##  ##  ##  ##  ##  ##  ##  ##  ##  ##  ##  ##  ##  ##  ##  ##  ##  ##  ##  ##  ##  ##  ##  ##  ##  ##  ##  ##  ##  ##  ##  ##  ##  ##  ##  ##  ##  ##  ##  ##  ##  ##  ## [2787_1]16	: 1  2  3  4  6  5  5  3  3  4  4  4  3  6  2  --  3  4  4  ##  ##  ##  ##  ##  ##  ##  ##  ##  ##  ##  ##  ##  ##  ##  ##  ##  ##  ##  ##  ##  ##  ##  ##  ##  ##  ##  ##  ##  ##  ##  ##  ##  ##  ##  ##  ##  ##  ##  ##  ##  ##  ##  ##  ##  ##  ##  ##  ##  ##  ##  ##  ##  ##  ##  ##  ##  ##  ##  ##  ##  ##  ##  ##  ##  ##  ##  ##  ##  ##  ##  ##  ##  ##  ##  ##  ##  ##  ##  ##  ##  ##  ##  ## [2860_1]17	: 2  3  4  5  7  6  6  4  4  5  5  5  4  7  1  3  --  5  5  ##  ##  ##  ##  ##  ##  ##  ##  ##  ##  ##  ##  ##  ##  ##  ##  ##  ##  ##  ##  ##  ##  ##  ##  ##  ##  ##  ##  ##  ##  ##  ##  ##  ##  ##  ##  ##  ##  ##  ##  ##  ##  ##  ##  ##  ##  ##  ##  ##  ##  ##  ##  ##  ##  ##  ##  ##  ##  ##  ##  ##  ##  ##  ##  ##  ##  ##  ##  ##  ##  ##  ##  ##  ##  ##  ##  ##  ##  ##  ##  ##  ##  ##  ## [1953_1]18	: 3  2  1  4  4  3  5  3  3  2  2  3  4  4  4  4  5  --  2  ##  ##  ##  ##  ##  ##  ##  ##  ##  ##  ##  ##  ##  ##  ##  ##  ##  ##  ##  ##  ##  ##  ##  ##  ##  ##  ##  ##  ##  ##  ##  ##  ##  ##  ##  ##  ##  ##  ##  ##  ##  ##  ##  ##  ##  ##  ##  ##  ##  ##  ##  ##  ##  ##  ##  ##  ##  ##  ##  ##  ##  ##  ##  ##  ##  ##  ##  ##  ##  ##  ##  ##  ##  ##  ##  ##  ##  ##  ##  ##  ##  ##  ##  ## [835_1]19	: 3  2  1  4  4  3  5  3  3  2  2  3  4  4  4  4  5  2  --  ##  ##  ##  ##  ##  ##  ##  ##  ##  ##  ##  ##  ##  ##  ##  ##  ##  ##  ##  ##  ##  ##  ##  ##  ##  ##  ##  ##  ##  ##  ##  ##  ##  ##  ##  ##  ##  ##  ##  ##  ##  ##  ##  ##  ##  ##  ##  ##  ##  ##  ##  ##  ##  ##  ##  ##  ##  ##  ##  ##  ##  ##  ##  ##  ##  ##  ##  ##  ##  ##  ##  ##  ##  ##  ##  ##  ##  ##  ##  ##  ##  ##  ##  ## [1311_2]20	: ##  ##  ##  ##  ##  ##  ##  ##  ##  ##  ##  ##  ##  ##  ##  ##  ##  ##  ##  --  1  1  1  2  4  2  ##  ##  ##  ##  ##  ##  ##  ##  ##  ##  ##  ##  ##  ##  ##  ##  ##  ##  ##  ##  ##  ##  ##  ##  ##  ##  ##  ##  ##  ##  ##  ##  ##  ##  ##  ##  ##  ##  ##  ##  ##  ##  ##  ##  ##  ##  ##  ##  ##  ##  ##  ##  ##  ##  ##  ##  ##  ##  ##  ##  ##  ##  ##  ##  ##  ##  ##  ##  ##  ##  ##  ##  ##  ##  ##  ##  ## [2180_2]21	: ##  ##  ##  ##  ##  ##  ##  ##  ##  ##  ##  ##  ##  ##  ##  ##  ##  ##  ##  1  --  2  2  3  3  1  ##  ##  ##  ##  ##  ##  ##  ##  ##  ##  ##  ##  ##  ##  ##  ##  ##  ##  ##  ##  ##  ##  ##  ##  ##  ##  ##  ##  ##  ##  ##  ##  ##  ##  ##  ##  ##  ##  ##  ##  ##  ##  ##  ##  ##  ##  ##  ##  ##  ##  ##  ##  ##  ##  ##  ##  ##  ##  ##  ##  ##  ##  ##  ##  ##  ##  ##  ##  ##  ##  ##  ##  ##  ##  ##  ##  ## [2187_2]22	: ##  ##  ##  ##  ##  ##  ##  ##  ##  ##  ##  ##  ##  ##  ##  ##  ##  ##  ##  1  2  --  2  3  5  3  ##  ##  ##  ##  ##  ##  ##  ##  ##  ##  ##  ##  ##  ##  ##  ##  ##  ##  ##  ##  ##  ##  ##  ##  ##  ##  ##  ##  ##  ##  ##  ##  ##  ##  ##  ##  ##  ##  ##  ##  ##  ##  ##  ##  ##  ##  ##  ##  ##  ##  ##  ##  ##  ##  ##  ##  ##  ##  ##  ##  ##  ##  ##  ##  ##  ##  ##  ##  ##  ##  ##  ##  ##  ##  ##  ##  ## [2333_2]23	: ##  ##  ##  ##  ##  ##  ##  ##  ##  ##  ##  ##  ##  ##  ##  ##  ##  ##  ##  1  2  2  --  1  3  1  ##  ##  ##  ##  ##  ##  ##  ##  ##  ##  ##  ##  ##  ##  ##  ##  ##  ##  ##  ##  ##  ##  ##  ##  ##  ##  ##  ##  ##  ##  ##  ##  ##  ##  ##  ##  ##  ##  ##  ##  ##  ##  ##  ##  ##  ##  ##  ##  ##  ##  ##  ##  ##  ##  ##  ##  ##  ##  ##  ##  ##  ##  ##  ##  ##  ##  ##  ##  ##  ##  ##  ##  ##  ##  ##  ##  ## [2390_2]24	: ##  ##  ##  ##  ##  ##  ##  ##  ##  ##  ##  ##  ##  ##  ##  ##  ##  ##  ##  2  3  3  1  --  2  2  ##  ##  ##  ##  ##  ##  ##  ##  ##  ##  ##  ##  ##  ##  ##  ##  ##  ##  ##  ##  ##  ##  ##  ##  ##  ##  ##  ##  ##  ##  ##  ##  ##  ##  ##  ##  ##  ##  ##  ##  ##  ##  ##  ##  ##  ##  ##  ##  ##  ##  ##  ##  ##  ##  ##  ##  ##  ##  ##  ##  ##  ##  ##  ##  ##  ##  ##  ##  ##  ##  ##  ##  ##  ##  ##  ##  ## [2382_2]25	: ##  ##  ##  ##  ##  ##  ##  ##  ##  ##  ##  ##  ##  ##  ##  ##  ##  ##  ##  4  3  5  3  2  --  2  ##  ##  ##  ##  ##  ##  ##  ##  ##  ##  ##  ##  ##  ##  ##  ##  ##  ##  ##  ##  ##  ##  ##  ##  ##  ##  ##  ##  ##  ##  ##  ##  ##  ##  ##  ##  ##  ##  ##  ##  ##  ##  ##  ##  ##  ##  ##  ##  ##  ##  ##  ##  ##  ##  ##  ##  ##  ##  ##  ##  ##  ##  ##  ##  ##  ##  ##  ##  ##  ##  ##  ##  ##  ##  ##  ##  ## [2353_2]26	: ##  ##  ##  ##  ##  ##  ##  ##  ##  ##  ##  ##  ##  ##  ##  ##  ##  ##  ##  2  1  3  1  2  2  --  ##  ##  ##  ##  ##  ##  ##  ##  ##  ##  ##  ##  ##  ##  ##  ##  ##  ##  ##  ##  ##  ##  ##  ##  ##  ##  ##  ##  ##  ##  ##  ##  ##  ##  ##  ##  ##  ##  ##  ##  ##  ##  ##  ##  ##  ##  ##  ##  ##  ##  ##  ##  ##  ##  ##  ##  ##  ##  ##  ##  ##  ##  ##  ##  ##  ##  ##  ##  ##  ##  ##  ##  ##  ##  ##  ##  ## [1207_3]27	: ##  ##  ##  ##  ##  ##  ##  ##  ##  ##  ##  ##  ##  ##  ##  ##  ##  ##  ##  ##  ##  ##  ##  ##  ##  ##  --  2  1  2  1  1  3  9  ##  ##  ##  ##  ##  ##  ##  ##  ##  ##  ##  ##  ##  ##  ##  ##  ##  ##  ##  ##  ##  ##  ##  ##  ##  ##  ##  ##  ##  ##  ##  ##  ##  ##  ##  ##  ##  ##  ##  ##  ##  ##  ##  ##  ##  ##  ##  ##  ##  ##  ##  ##  ##  ##  ##  ##  ##  ##  ##  ##  ##  ##  ##  ##  ##  ##  ##  ##  ## [2275_3]28	: ##  ##  ##  ##  ##  ##  ##  ##  ##  ##  ##  ##  ##  ##  ##  ##  ##  ##  ##  ##  ##  ##  ##  ##  ##  ##  2  --  1  2  2  3  1  9  ##  ##  ##  ##  ##  ##  ##  ##  ##  ##  ##  ##  ##  ##  ##  ##  ##  ##  ##  ##  ##  ##  ##  ##  ##  ##  ##  ##  ##  ##  ##  ##  ##  ##  ##  ##  ##  ##  ##  ##  ##  ##  ##  ##  ##  ##  ##  ##  ##  ##  ##  ##  ##  ##  ##  ##  ##  ##  ##  ##  ##  ##  ##  ##  ##  ##  ##  ##  ## [2287_3]29	: ##  ##  ##  ##  ##  ##  ##  ##  ##  ##  ##  ##  ##  ##  ##  ##  ##  ##  ##  ##  ##  ##  ##  ##  ##  ##  1  1  --  1  1  2  2  8  ##  ##  ##  ##  ##  ##  ##  ##  ##  ##  ##  ##  ##  ##  ##  ##  ##  ##  ##  ##  ##  ##  ##  ##  ##  ##  ##  ##  ##  ##  ##  ##  ##  ##  ##  ##  ##  ##  ##  ##  ##  ##  ##  ##  ##  ##  ##  ##  ##  ##  ##  ##  ##  ##  ##  ##  ##  ##  ##  ##  ##  ##  ##  ##  ##  ##  ##  ##  ## [2463_3]30	: ##  ##  ##  ##  ##  ##  ##  ##  ##  ##  ##  ##  ##  ##  ##  ##  ##  ##  ##  ##  ##  ##  ##  ##  ##  ##  2  2  1  --  2  3  3  9  ##  ##  ##  ##  ##  ##  ##  ##  ##  ##  ##  ##  ##  ##  ##  ##  ##  ##  ##  ##  ##  ##  ##  ##  ##  ##  ##  ##  ##  ##  ##  ##  ##  ##  ##  ##  ##  ##  ##  ##  ##  ##  ##  ##  ##  ##  ##  ##  ##  ##  ##  ##  ##  ##  ##  ##  ##  ##  ##  ##  ##  ##  ##  ##  ##  ##  ##  ##  ## [2878_3]31	: ##  ##  ##  ##  ##  ##  ##  ##  ##  ##  ##  ##  ##  ##  ##  ##  ##  ##  ##  ##  ##  ##  ##  ##  ##  ##  1  2  1  2  --  2  3  9  ##  ##  ##  ##  ##  ##  ##  ##  ##  ##  ##  ##  ##  ##  ##  ##  ##  ##  ##  ##  ##  ##  ##  ##  ##  ##  ##  ##  ##  ##  ##  ##  ##  ##  ##  ##  ##  ##  ##  ##  ##  ##  ##  ##  ##  ##  ##  ##  ##  ##  ##  ##  ##  ##  ##  ##  ##  ##  ##  ##  ##  ##  ##  ##  ##  ##  ##  ##  ## [2883_3]32	: ##  ##  ##  ##  ##  ##  ##  ##  ##  ##  ##  ##  ##  ##  ##  ##  ##  ##  ##  ##  ##  ##  ##  ##  ##  ##  1  3  2  3  2  --  4  10  ##  ##  ##  ##  ##  ##  ##  ##  ##  ##  ##  ##  ##  ##  ##  ##  ##  ##  ##  ##  ##  ##  ##  ##  ##  ##  ##  ##  ##  ##  ##  ##  ##  ##  ##  ##  ##  ##  ##  ##  ##  ##  ##  ##  ##  ##  ##  ##  ##  ##  ##  ##  ##  ##  ##  ##  ##  ##  ##  ##  ##  ##  ##  ##  ##  ##  ##  ##  ## [2286_3]33	: ##  ##  ##  ##  ##  ##  ##  ##  ##  ##  ##  ##  ##  ##  ##  ##  ##  ##  ##  ##  ##  ##  ##  ##  ##  ##  3  1  2  3  3  4  --  8  ##  ##  ##  ##  ##  ##  ##  ##  ##  ##  ##  ##  ##  ##  ##  ##  ##  ##  ##  ##  ##  ##  ##  ##  ##  ##  ##  ##  ##  ##  ##  ##  ##  ##  ##  ##  ##  ##  ##  ##  ##  ##  ##  ##  ##  ##  ##  ##  ##  ##  ##  ##  ##  ##  ##  ##  ##  ##  ##  ##  ##  ##  ##  ##  ##  ##  ##  ##  ## [2814_3]34	: ##  ##  ##  ##  ##  ##  ##  ##  ##  ##  ##  ##  ##  ##  ##  ##  ##  ##  ##  ##  ##  ##  ##  ##  ##  ##  9  9  8  9  9  10  8  --  ##  ##  ##  ##  ##  ##  ##  ##  ##  ##  ##  ##  ##  ##  ##  ##  ##  ##  ##  ##  ##  ##  ##  ##  ##  ##  ##  ##  ##  ##  ##  ##  ##  ##  ##  ##  ##  ##  ##  ##  ##  ##  ##  ##  ##  ##  ##  ##  ##  ##  ##  ##  ##  ##  ##  ##  ##  ##  ##  ##  ##  ##  ##  ##  ##  ##  ##  ##  ## [2226_4]35	: ##  ##  ##  ##  ##  ##  ##  ##  ##  ##  ##  ##  ##  ##  ##  ##  ##  ##  ##  ##  ##  ##  ##  ##  ##  ##  ##  ##  ##  ##  ##  ##  ##  ##  --  ##  ##  ##  ##  ##  ##  ##  ##  ##  ##  ##  ##  ##  ##  ##  ##  ##  ##  ##  ##  ##  ##  ##  ##  ##  ##  ##  ##  ##  ##  ##  ##  ##  ##  ##  ##  ##  ##  ##  ##  ##  ##  ##  ##  ##  ##  ##  ##  ##  ##  ##  ##  ##  ##  ##  ##  ##  ##  ##  ##  ##  ##  ##  ##  ##  ##  3  7 [840_5]36	: ##  ##  ##  ##  ##  ##  ##  ##  ##  ##  ##  ##  ##  ##  ##  ##  ##  ##  ##  ##  ##  ##  ##  ##  ##  ##  ##  ##  ##  ##  ##  ##  ##  ##  ##  --  1  1  4  ##  ##  ##  ##  ##  ##  ##  ##  ##  ##  ##  ##  ##  ##  ##  ##  ##  ##  ##  ##  ##  ##  ##  ##  ##  ##  ##  ##  ##  ##  ##  ##  ##  ##  ##  ##  ##  ##  ##  ##  ##  ##  ##  ##  ##  ##  ##  ##  9  11  12  12  ##  ##  ##  ##  ##  ##  ##  ##  ##  ##  ##  ## [2778_5]37	: ##  ##  ##  ##  ##  ##  ##  ##  ##  ##  ##  ##  ##  ##  ##  ##  ##  ##  ##  ##  ##  ##  ##  ##  ##  ##  ##  ##  ##  ##  ##  ##  ##  ##  ##  1  --  2  3  ##  ##  ##  ##  ##  ##  ##  ##  ##  ##  ##  ##  ##  ##  ##  ##  ##  ##  ##  ##  ##  ##  ##  ##  ##  ##  ##  ##  ##  ##  ##  ##  ##  ##  ##  ##  ##  ##  ##  ##  ##  ##  ##  ##  ##  ##  ##  ##  8  10  11  11  ##  ##  ##  ##  ##  ##  ##  ##  ##  ##  ##  ## [842_5]38	: ##  ##  ##  ##  ##  ##  ##  ##  ##  ##  ##  ##  ##  ##  ##  ##  ##  ##  ##  ##  ##  ##  ##  ##  ##  ##  ##  ##  ##  ##  ##  ##  ##  ##  ##  1  2  --  5  ##  ##  ##  ##  ##  ##  ##  ##  ##  ##  ##  ##  ##  ##  ##  ##  ##  ##  ##  ##  ##  ##  ##  ##  ##  ##  ##  ##  ##  ##  ##  ##  ##  ##  ##  ##  ##  ##  ##  ##  ##  ##  ##  ##  ##  ##  ##  ##  10  12  13  13  ##  ##  ##  ##  ##  ##  ##  ##  ##  ##  ##  ## [2900_5]39	: ##  ##  ##  ##  ##  ##  ##  ##  ##  ##  ##  ##  ##  ##  ##  ##  ##  ##  ##  ##  ##  ##  ##  ##  ##  ##  ##  ##  ##  ##  ##  ##  ##  ##  ##  4  3  5  --  ##  ##  ##  ##  ##  ##  ##  ##  ##  ##  ##  ##  ##  ##  ##  ##  ##  ##  ##  ##  ##  ##  ##  ##  ##  ##  ##  ##  ##  ##  ##  ##  ##  ##  ##  ##  ##  ##  ##  ##  ##  ##  ##  ##  ##  ##  ##  ##  7  9  10  10  ##  ##  ##  ##  ##  ##  ##  ##  ##  ##  ##  ## [1874_6]40	: ##  ##  ##  ##  ##  ##  ##  ##  ##  ##  ##  ##  ##  ##  ##  ##  ##  ##  ##  ##  ##  ##  ##  ##  ##  ##  ##  ##  ##  ##  ##  ##  ##  ##  ##  ##  ##  ##  ##  --  3  4  2  1  2  11  1  1  1  4  4  ##  ##  ##  ##  ##  ##  ##  ##  ##  ##  ##  ##  ##  ##  ##  ##  ##  ##  ##  ##  ##  ##  ##  ##  ##  ##  ##  ##  ##  ##  ##  ##  ##  ##  ##  ##  ##  ##  ##  ##  ##  ##  ##  ##  ##  ##  ##  ##  ##  ##  ##  ## [846_6]41	: ##  ##  ##  ##  ##  ##  ##  ##  ##  ##  ##  ##  ##  ##  ##  ##  ##  ##  ##  ##  ##  ##  ##  ##  ##  ##  ##  ##  ##  ##  ##  ##  ##  ##  ##  ##  ##  ##  ##  3  --  1  1  3  5  8  2  2  3  1  1  ##  ##  ##  ##  ##  ##  ##  ##  ##  ##  ##  ##  ##  ##  ##  ##  ##  ##  ##  ##  ##  ##  ##  ##  ##  ##  ##  ##  ##  ##  ##  ##  ##  ##  ##  ##  ##  ##  ##  ##  ##  ##  ##  ##  ##  ##  ##  ##  ##  ##  ##  ## [1943_6]42	: ##  ##  ##  ##  ##  ##  ##  ##  ##  ##  ##  ##  ##  ##  ##  ##  ##  ##  ##  ##  ##  ##  ##  ##  ##  ##  ##  ##  ##  ##  ##  ##  ##  ##  ##  ##  ##  ##  ##  4  1  --  2  4  6  9  3  3  4  2  2  ##  ##  ##  ##  ##  ##  ##  ##  ##  ##  ##  ##  ##  ##  ##  ##  ##  ##  ##  ##  ##  ##  ##  ##  ##  ##  ##  ##  ##  ##  ##  ##  ##  ##  ##  ##  ##  ##  ##  ##  ##  ##  ##  ##  ##  ##  ##  ##  ##  ##  ##  ## [1318_6]43	: ##  ##  ##  ##  ##  ##  ##  ##  ##  ##  ##  ##  ##  ##  ##  ##  ##  ##  ##  ##  ##  ##  ##  ##  ##  ##  ##  ##  ##  ##  ##  ##  ##  ##  ##  ##  ##  ##  ##  2  1  2  --  2  4  9  1  1  2  2  2  ##  ##  ##  ##  ##  ##  ##  ##  ##  ##  ##  ##  ##  ##  ##  ##  ##  ##  ##  ##  ##  ##  ##  ##  ##  ##  ##  ##  ##  ##  ##  ##  ##  ##  ##  ##  ##  ##  ##  ##  ##  ##  ##  ##  ##  ##  ##  ##  ##  ##  ##  ## [1869_6]44	: ##  ##  ##  ##  ##  ##  ##  ##  ##  ##  ##  ##  ##  ##  ##  ##  ##  ##  ##  ##  ##  ##  ##  ##  ##  ##  ##  ##  ##  ##  ##  ##  ##  ##  ##  ##  ##  ##  ##  1  3  4  2  --  3  11  2  1  2  4  4  ##  ##  ##  ##  ##  ##  ##  ##  ##  ##  ##  ##  ##  ##  ##  ##  ##  ##  ##  ##  ##  ##  ##  ##  ##  ##  ##  ##  ##  ##  ##  ##  ##  ##  ##  ##  ##  ##  ##  ##  ##  ##  ##  ##  ##  ##  ##  ##  ##  ##  ##  ## [845_6]45	: ##  ##  ##  ##  ##  ##  ##  ##  ##  ##  ##  ##  ##  ##  ##  ##  ##  ##  ##  ##  ##  ##  ##  ##  ##  ##  ##  ##  ##  ##  ##  ##  ##  ##  ##  ##  ##  ##  ##  2  5  6  4  3  --  13  3  3  3  6  6  ##  ##  ##  ##  ##  ##  ##  ##  ##  ##  ##  ##  ##  ##  ##  ##  ##  ##  ##  ##  ##  ##  ##  ##  ##  ##  ##  ##  ##  ##  ##  ##  ##  ##  ##  ##  ##  ##  ##  ##  ##  ##  ##  ##  ##  ##  ##  ##  ##  ##  ##  ## [860_6]46	: ##  ##  ##  ##  ##  ##  ##  ##  ##  ##  ##  ##  ##  ##  ##  ##  ##  ##  ##  ##  ##  ##  ##  ##  ##  ##  ##  ##  ##  ##  ##  ##  ##  ##  ##  ##  ##  ##  ##  11  8  9  9  11  13  --  10  10  11  9  9  ##  ##  ##  ##  ##  ##  ##  ##  ##  ##  ##  ##  ##  ##  ##  ##  ##  ##  ##  ##  ##  ##  ##  ##  ##  ##  ##  ##  ##  ##  ##  ##  ##  ##  ##  ##  ##  ##  ##  ##  ##  ##  ##  ##  ##  ##  ##  ##  ##  ##  ##  ## [1317_6]47	: ##  ##  ##  ##  ##  ##  ##  ##  ##  ##  ##  ##  ##  ##  ##  ##  ##  ##  ##  ##  ##  ##  ##  ##  ##  ##  ##  ##  ##  ##  ##  ##  ##  ##  ##  ##  ##  ##  ##  1  2  3  1  2  3  10  --  2  1  3  3  ##  ##  ##  ##  ##  ##  ##  ##  ##  ##  ##  ##  ##  ##  ##  ##  ##  ##  ##  ##  ##  ##  ##  ##  ##  ##  ##  ##  ##  ##  ##  ##  ##  ##  ##  ##  ##  ##  ##  ##  ##  ##  ##  ##  ##  ##  ##  ##  ##  ##  ##  ## [1871_6]48	: ##  ##  ##  ##  ##  ##  ##  ##  ##  ##  ##  ##  ##  ##  ##  ##  ##  ##  ##  ##  ##  ##  ##  ##  ##  ##  ##  ##  ##  ##  ##  ##  ##  ##  ##  ##  ##  ##  ##  1  2  3  1  1  3  10  2  --  2  3  3  ##  ##  ##  ##  ##  ##  ##  ##  ##  ##  ##  ##  ##  ##  ##  ##  ##  ##  ##  ##  ##  ##  ##  ##  ##  ##  ##  ##  ##  ##  ##  ##  ##  ##  ##  ##  ##  ##  ##  ##  ##  ##  ##  ##  ##  ##  ##  ##  ##  ##  ##  ## [2170_6]49	: ##  ##  ##  ##  ##  ##  ##  ##  ##  ##  ##  ##  ##  ##  ##  ##  ##  ##  ##  ##  ##  ##  ##  ##  ##  ##  ##  ##  ##  ##  ##  ##  ##  ##  ##  ##  ##  ##  ##  1  3  4  2  2  3  11  1  2  --  4  4  ##  ##  ##  ##  ##  ##  ##  ##  ##  ##  ##  ##  ##  ##  ##  ##  ##  ##  ##  ##  ##  ##  ##  ##  ##  ##  ##  ##  ##  ##  ##  ##  ##  ##  ##  ##  ##  ##  ##  ##  ##  ##  ##  ##  ##  ##  ##  ##  ##  ##  ##  ## [2169_6]50	: ##  ##  ##  ##  ##  ##  ##  ##  ##  ##  ##  ##  ##  ##  ##  ##  ##  ##  ##  ##  ##  ##  ##  ##  ##  ##  ##  ##  ##  ##  ##  ##  ##  ##  ##  ##  ##  ##  ##  4  1  2  2  4  6  9  3  3  4  --  2  ##  ##  ##  ##  ##  ##  ##  ##  ##  ##  ##  ##  ##  ##  ##  ##  ##  ##  ##  ##  ##  ##  ##  ##  ##  ##  ##  ##  ##  ##  ##  ##  ##  ##  ##  ##  ##  ##  ##  ##  ##  ##  ##  ##  ##  ##  ##  ##  ##  ##  ##  ## [2173_6]51	: ##  ##  ##  ##  ##  ##  ##  ##  ##  ##  ##  ##  ##  ##  ##  ##  ##  ##  ##  ##  ##  ##  ##  ##  ##  ##  ##  ##  ##  ##  ##  ##  ##  ##  ##  ##  ##  ##  ##  4  1  2  2  4  6  9  3  3  4  2  --  ##  ##  ##  ##  ##  ##  ##  ##  ##  ##  ##  ##  ##  ##  ##  ##  ##  ##  ##  ##  ##  ##  ##  ##  ##  ##  ##  ##  ##  ##  ##  ##  ##  ##  ##  ##  ##  ##  ##  ##  ##  ##  ##  ##  ##  ##  ##  ##  ##  ##  ##  ## [1309_7]52	: ##  ##  ##  ##  ##  ##  ##  ##  ##  ##  ##  ##  ##  ##  ##  ##  ##  ##  ##  ##  ##  ##  ##  ##  ##  ##  ##  ##  ##  ##  ##  ##  ##  ##  ##  ##  ##  ##  ##  ##  ##  ##  ##  ##  ##  ##  ##  ##  ##  ##  ##  --  1  6  2  ##  ##  ##  ##  ##  ##  ##  ##  ##  ##  ##  ##  ##  ##  ##  ##  ##  ##  ##  ##  ##  ##  ##  ##  ##  ##  ##  ##  ##  ##  ##  ##  ##  ##  ##  ##  ##  ##  ##  ##  ##  ##  ##  ##  ##  ##  ##  ## [2448_7]53	: ##  ##  ##  ##  ##  ##  ##  ##  ##  ##  ##  ##  ##  ##  ##  ##  ##  ##  ##  ##  ##  ##  ##  ##  ##  ##  ##  ##  ##  ##  ##  ##  ##  ##  ##  ##  ##  ##  ##  ##  ##  ##  ##  ##  ##  ##  ##  ##  ##  ##  ##  1  --  7  1  ##  ##  ##  ##  ##  ##  ##  ##  ##  ##  ##  ##  ##  ##  ##  ##  ##  ##  ##  ##  ##  ##  ##  ##  ##  ##  ##  ##  ##  ##  ##  ##  ##  ##  ##  ##  ##  ##  ##  ##  ##  ##  ##  ##  ##  ##  ##  ## [2442_7]54	: ##  ##  ##  ##  ##  ##  ##  ##  ##  ##  ##  ##  ##  ##  ##  ##  ##  ##  ##  ##  ##  ##  ##  ##  ##  ##  ##  ##  ##  ##  ##  ##  ##  ##  ##  ##  ##  ##  ##  ##  ##  ##  ##  ##  ##  ##  ##  ##  ##  ##  ##  6  7  --  8  ##  ##  ##  ##  ##  ##  ##  ##  ##  ##  ##  ##  ##  ##  ##  ##  ##  ##  ##  ##  ##  ##  ##  ##  ##  ##  ##  ##  ##  ##  ##  ##  ##  ##  ##  ##  ##  ##  ##  ##  ##  ##  ##  ##  ##  ##  ##  ## [2449_7]55	: ##  ##  ##  ##  ##  ##  ##  ##  ##  ##  ##  ##  ##  ##  ##  ##  ##  ##  ##  ##  ##  ##  ##  ##  ##  ##  ##  ##  ##  ##  ##  ##  ##  ##  ##  ##  ##  ##  ##  ##  ##  ##  ##  ##  ##  ##  ##  ##  ##  ##  ##  2  1  8  --  ##  ##  ##  ##  ##  ##  ##  ##  ##  ##  ##  ##  ##  ##  ##  ##  ##  ##  ##  ##  ##  ##  ##  ##  ##  ##  ##  ##  ##  ##  ##  ##  ##  ##  ##  ##  ##  ##  ##  ##  ##  ##  ##  ##  ##  ##  ##  ## [1202_8]56	: ##  ##  ##  ##  ##  ##  ##  ##  ##  ##  ##  ##  ##  ##  ##  ##  ##  ##  ##  ##  ##  ##  ##  ##  ##  ##  ##  ##  ##  ##  ##  ##  ##  ##  ##  ##  ##  ##  ##  ##  ##  ##  ##  ##  ##  ##  ##  ##  ##  ##  ##  ##  ##  ##  ##  --  2  1  2  1  1  2  ##  ##  ##  ##  ##  ##  ##  ##  ##  ##  ##  ##  ##  ##  ##  ##  ##  ##  ##  ##  ##  ##  ##  ##  ##  ##  ##  ##  ##  ##  ##  ##  ##  ##  ##  ##  ##  ##  ##  ##  ## [1203_8]57	: ##  ##  ##  ##  ##  ##  ##  ##  ##  ##  ##  ##  ##  ##  ##  ##  ##  ##  ##  ##  ##  ##  ##  ##  ##  ##  ##  ##  ##  ##  ##  ##  ##  ##  ##  ##  ##  ##  ##  ##  ##  ##  ##  ##  ##  ##  ##  ##  ##  ##  ##  ##  ##  ##  ##  2  --  1  2  3  3  2  ##  ##  ##  ##  ##  ##  ##  ##  ##  ##  ##  ##  ##  ##  ##  ##  ##  ##  ##  ##  ##  ##  ##  ##  ##  ##  ##  ##  ##  ##  ##  ##  ##  ##  ##  ##  ##  ##  ##  ##  ## [1946_8]58	: ##  ##  ##  ##  ##  ##  ##  ##  ##  ##  ##  ##  ##  ##  ##  ##  ##  ##  ##  ##  ##  ##  ##  ##  ##  ##  ##  ##  ##  ##  ##  ##  ##  ##  ##  ##  ##  ##  ##  ##  ##  ##  ##  ##  ##  ##  ##  ##  ##  ##  ##  ##  ##  ##  ##  1  1  --  1  2  2  1  ##  ##  ##  ##  ##  ##  ##  ##  ##  ##  ##  ##  ##  ##  ##  ##  ##  ##  ##  ##  ##  ##  ##  ##  ##  ##  ##  ##  ##  ##  ##  ##  ##  ##  ##  ##  ##  ##  ##  ##  ## [1198_8]59	: ##  ##  ##  ##  ##  ##  ##  ##  ##  ##  ##  ##  ##  ##  ##  ##  ##  ##  ##  ##  ##  ##  ##  ##  ##  ##  ##  ##  ##  ##  ##  ##  ##  ##  ##  ##  ##  ##  ##  ##  ##  ##  ##  ##  ##  ##  ##  ##  ##  ##  ##  ##  ##  ##  ##  2  2  1  --  3  3  2  ##  ##  ##  ##  ##  ##  ##  ##  ##  ##  ##  ##  ##  ##  ##  ##  ##  ##  ##  ##  ##  ##  ##  ##  ##  ##  ##  ##  ##  ##  ##  ##  ##  ##  ##  ##  ##  ##  ##  ##  ## [1957_8]60	: ##  ##  ##  ##  ##  ##  ##  ##  ##  ##  ##  ##  ##  ##  ##  ##  ##  ##  ##  ##  ##  ##  ##  ##  ##  ##  ##  ##  ##  ##  ##  ##  ##  ##  ##  ##  ##  ##  ##  ##  ##  ##  ##  ##  ##  ##  ##  ##  ##  ##  ##  ##  ##  ##  ##  1  3  2  3  --  2  3  ##  ##  ##  ##  ##  ##  ##  ##  ##  ##  ##  ##  ##  ##  ##  ##  ##  ##  ##  ##  ##  ##  ##  ##  ##  ##  ##  ##  ##  ##  ##  ##  ##  ##  ##  ##  ##  ##  ##  ##  ## [2920_8]61	: ##  ##  ##  ##  ##  ##  ##  ##  ##  ##  ##  ##  ##  ##  ##  ##  ##  ##  ##  ##  ##  ##  ##  ##  ##  ##  ##  ##  ##  ##  ##  ##  ##  ##  ##  ##  ##  ##  ##  ##  ##  ##  ##  ##  ##  ##  ##  ##  ##  ##  ##  ##  ##  ##  ##  1  3  2  3  2  --  3  ##  ##  ##  ##  ##  ##  ##  ##  ##  ##  ##  ##  ##  ##  ##  ##  ##  ##  ##  ##  ##  ##  ##  ##  ##  ##  ##  ##  ##  ##  ##  ##  ##  ##  ##  ##  ##  ##  ##  ##  ## [2476_8]62	: ##  ##  ##  ##  ##  ##  ##  ##  ##  ##  ##  ##  ##  ##  ##  ##  ##  ##  ##  ##  ##  ##  ##  ##  ##  ##  ##  ##  ##  ##  ##  ##  ##  ##  ##  ##  ##  ##  ##  ##  ##  ##  ##  ##  ##  ##  ##  ##  ##  ##  ##  ##  ##  ##  ##  2  2  1  2  3  3  --  ##  ##  ##  ##  ##  ##  ##  ##  ##  ##  ##  ##  ##  ##  ##  ##  ##  ##  ##  ##  ##  ##  ##  ##  ##  ##  ##  ##  ##  ##  ##  ##  ##  ##  ##  ##  ##  ##  ##  ##  ## [862_9]63	: ##  ##  ##  ##  ##  ##  ##  ##  ##  ##  ##  ##  ##  ##  ##  ##  ##  ##  ##  ##  ##  ##  ##  ##  ##  ##  ##  ##  ##  ##  ##  ##  ##  ##  ##  ##  ##  ##  ##  ##  ##  ##  ##  ##  ##  ##  ##  ##  ##  ##  ##  ##  ##  ##  ##  ##  ##  ##  ##  ##  ##  ##  --  ##  ##  ##  ##  ##  ##  ##  ##  ##  ##  ##  ##  ##  ##  ##  ##  ##  ##  ##  ##  ##  ##  ##  ##  ##  ##  ##  ##  ##  ##  ##  ##  ##  ##  ##  ##  ##  ##  ##  ## [2031_10]64	: ##  ##  ##  ##  ##  ##  ##  ##  ##  ##  ##  ##  ##  ##  ##  ##  ##  ##  ##  ##  ##  ##  ##  ##  ##  ##  ##  ##  ##  ##  ##  ##  ##  ##  ##  ##  ##  ##  ##  ##  ##  ##  ##  ##  ##  ##  ##  ##  ##  ##  ##  ##  ##  ##  ##  ##  ##  ##  ##  ##  ##  ##  ##  --  1  ##  ##  ##  ##  ##  ##  ##  ##  ##  ##  ##  ##  ##  ##  ##  ##  ##  ##  ##  ##  ##  ##  ##  ##  ##  ##  ##  ##  ##  ##  ##  ##  ##  ##  ##  ##  ##  ## [2026_10]65	: ##  ##  ##  ##  ##  ##  ##  ##  ##  ##  ##  ##  ##  ##  ##  ##  ##  ##  ##  ##  ##  ##  ##  ##  ##  ##  ##  ##  ##  ##  ##  ##  ##  ##  ##  ##  ##  ##  ##  ##  ##  ##  ##  ##  ##  ##  ##  ##  ##  ##  ##  ##  ##  ##  ##  ##  ##  ##  ##  ##  ##  ##  ##  1  --  ##  ##  ##  ##  ##  ##  ##  ##  ##  ##  ##  ##  ##  ##  ##  ##  ##  ##  ##  ##  ##  ##  ##  ##  ##  ##  ##  ##  ##  ##  ##  ##  ##  ##  ##  ##  ##  ## [2786_11]66	: ##  ##  ##  ##  ##  ##  ##  ##  ##  ##  ##  ##  ##  ##  ##  ##  ##  ##  ##  ##  ##  ##  ##  ##  ##  ##  ##  ##  ##  ##  ##  ##  ##  ##  ##  ##  ##  ##  ##  ##  ##  ##  ##  ##  ##  ##  ##  ##  ##  ##  ##  ##  ##  ##  ##  ##  ##  ##  ##  ##  ##  ##  ##  ##  ##  --  1  ##  ##  ##  ##  ##  ##  ##  ##  ##  ##  ##  ##  ##  ##  ##  ##  ##  ##  ##  ##  ##  ##  ##  ##  ##  ##  ##  ##  ##  ##  ##  ##  ##  ##  ##  ## [2323_11]67	: ##  ##  ##  ##  ##  ##  ##  ##  ##  ##  ##  ##  ##  ##  ##  ##  ##  ##  ##  ##  ##  ##  ##  ##  ##  ##  ##  ##  ##  ##  ##  ##  ##  ##  ##  ##  ##  ##  ##  ##  ##  ##  ##  ##  ##  ##  ##  ##  ##  ##  ##  ##  ##  ##  ##  ##  ##  ##  ##  ##  ##  ##  ##  ##  ##  1  --  ##  ##  ##  ##  ##  ##  ##  ##  ##  ##  ##  ##  ##  ##  ##  ##  ##  ##  ##  ##  ##  ##  ##  ##  ##  ##  ##  ##  ##  ##  ##  ##  ##  ##  ##  ## [2196_12]68	: ##  ##  ##  ##  ##  ##  ##  ##  ##  ##  ##  ##  ##  ##  ##  ##  ##  ##  ##  ##  ##  ##  ##  ##  ##  ##  ##  ##  ##  ##  ##  ##  ##  ##  ##  ##  ##  ##  ##  ##  ##  ##  ##  ##  ##  ##  ##  ##  ##  ##  ##  ##  ##  ##  ##  ##  ##  ##  ##  ##  ##  ##  ##  ##  ##  ##  ##  --  1  1  1  1  2  2  2  2  1  ##  ##  ##  ##  ##  ##  ##  ##  ##  ##  ##  ##  ##  ##  ##  ##  ##  ##  ##  ##  ##  ##  ##  ##  ##  ## [2806_12]69	: ##  ##  ##  ##  ##  ##  ##  ##  ##  ##  ##  ##  ##  ##  ##  ##  ##  ##  ##  ##  ##  ##  ##  ##  ##  ##  ##  ##  ##  ##  ##  ##  ##  ##  ##  ##  ##  ##  ##  ##  ##  ##  ##  ##  ##  ##  ##  ##  ##  ##  ##  ##  ##  ##  ##  ##  ##  ##  ##  ##  ##  ##  ##  ##  ##  ##  ##  1  --  2  2  2  1  3  3  3  2  ##  ##  ##  ##  ##  ##  ##  ##  ##  ##  ##  ##  ##  ##  ##  ##  ##  ##  ##  ##  ##  ##  ##  ##  ##  ## [2194_12]70	: ##  ##  ##  ##  ##  ##  ##  ##  ##  ##  ##  ##  ##  ##  ##  ##  ##  ##  ##  ##  ##  ##  ##  ##  ##  ##  ##  ##  ##  ##  ##  ##  ##  ##  ##  ##  ##  ##  ##  ##  ##  ##  ##  ##  ##  ##  ##  ##  ##  ##  ##  ##  ##  ##  ##  ##  ##  ##  ##  ##  ##  ##  ##  ##  ##  ##  ##  1  2  --  2  2  3  3  1  3  2  ##  ##  ##  ##  ##  ##  ##  ##  ##  ##  ##  ##  ##  ##  ##  ##  ##  ##  ##  ##  ##  ##  ##  ##  ##  ## [2201_12]71	: ##  ##  ##  ##  ##  ##  ##  ##  ##  ##  ##  ##  ##  ##  ##  ##  ##  ##  ##  ##  ##  ##  ##  ##  ##  ##  ##  ##  ##  ##  ##  ##  ##  ##  ##  ##  ##  ##  ##  ##  ##  ##  ##  ##  ##  ##  ##  ##  ##  ##  ##  ##  ##  ##  ##  ##  ##  ##  ##  ##  ##  ##  ##  ##  ##  ##  ##  1  2  2  --  2  3  3  3  1  2  ##  ##  ##  ##  ##  ##  ##  ##  ##  ##  ##  ##  ##  ##  ##  ##  ##  ##  ##  ##  ##  ##  ##  ##  ##  ## [2202_12]72	: ##  ##  ##  ##  ##  ##  ##  ##  ##  ##  ##  ##  ##  ##  ##  ##  ##  ##  ##  ##  ##  ##  ##  ##  ##  ##  ##  ##  ##  ##  ##  ##  ##  ##  ##  ##  ##  ##  ##  ##  ##  ##  ##  ##  ##  ##  ##  ##  ##  ##  ##  ##  ##  ##  ##  ##  ##  ##  ##  ##  ##  ##  ##  ##  ##  ##  ##  1  2  2  2  --  3  3  3  3  2  ##  ##  ##  ##  ##  ##  ##  ##  ##  ##  ##  ##  ##  ##  ##  ##  ##  ##  ##  ##  ##  ##  ##  ##  ##  ## [2171_12]73	: ##  ##  ##  ##  ##  ##  ##  ##  ##  ##  ##  ##  ##  ##  ##  ##  ##  ##  ##  ##  ##  ##  ##  ##  ##  ##  ##  ##  ##  ##  ##  ##  ##  ##  ##  ##  ##  ##  ##  ##  ##  ##  ##  ##  ##  ##  ##  ##  ##  ##  ##  ##  ##  ##  ##  ##  ##  ##  ##  ##  ##  ##  ##  ##  ##  ##  ##  2  1  3  3  3  --  4  4  4  3  ##  ##  ##  ##  ##  ##  ##  ##  ##  ##  ##  ##  ##  ##  ##  ##  ##  ##  ##  ##  ##  ##  ##  ##  ##  ## [2225_12]74	: ##  ##  ##  ##  ##  ##  ##  ##  ##  ##  ##  ##  ##  ##  ##  ##  ##  ##  ##  ##  ##  ##  ##  ##  ##  ##  ##  ##  ##  ##  ##  ##  ##  ##  ##  ##  ##  ##  ##  ##  ##  ##  ##  ##  ##  ##  ##  ##  ##  ##  ##  ##  ##  ##  ##  ##  ##  ##  ##  ##  ##  ##  ##  ##  ##  ##  ##  2  3  3  3  3  4  --  2  4  2  ##  ##  ##  ##  ##  ##  ##  ##  ##  ##  ##  ##  ##  ##  ##  ##  ##  ##  ##  ##  ##  ##  ##  ##  ##  ## [2224_12]75	: ##  ##  ##  ##  ##  ##  ##  ##  ##  ##  ##  ##  ##  ##  ##  ##  ##  ##  ##  ##  ##  ##  ##  ##  ##  ##  ##  ##  ##  ##  ##  ##  ##  ##  ##  ##  ##  ##  ##  ##  ##  ##  ##  ##  ##  ##  ##  ##  ##  ##  ##  ##  ##  ##  ##  ##  ##  ##  ##  ##  ##  ##  ##  ##  ##  ##  ##  2  3  1  3  3  4  2  --  4  3  ##  ##  ##  ##  ##  ##  ##  ##  ##  ##  ##  ##  ##  ##  ##  ##  ##  ##  ##  ##  ##  ##  ##  ##  ##  ## [2198_12]76	: ##  ##  ##  ##  ##  ##  ##  ##  ##  ##  ##  ##  ##  ##  ##  ##  ##  ##  ##  ##  ##  ##  ##  ##  ##  ##  ##  ##  ##  ##  ##  ##  ##  ##  ##  ##  ##  ##  ##  ##  ##  ##  ##  ##  ##  ##  ##  ##  ##  ##  ##  ##  ##  ##  ##  ##  ##  ##  ##  ##  ##  ##  ##  ##  ##  ##  ##  2  3  3  1  3  4  4  4  --  3  ##  ##  ##  ##  ##  ##  ##  ##  ##  ##  ##  ##  ##  ##  ##  ##  ##  ##  ##  ##  ##  ##  ##  ##  ##  ## [1312_12]77	: ##  ##  ##  ##  ##  ##  ##  ##  ##  ##  ##  ##  ##  ##  ##  ##  ##  ##  ##  ##  ##  ##  ##  ##  ##  ##  ##  ##  ##  ##  ##  ##  ##  ##  ##  ##  ##  ##  ##  ##  ##  ##  ##  ##  ##  ##  ##  ##  ##  ##  ##  ##  ##  ##  ##  ##  ##  ##  ##  ##  ##  ##  ##  ##  ##  ##  ##  1  2  2  2  2  3  2  3  3  --  ##  ##  ##  ##  ##  ##  ##  ##  ##  ##  ##  ##  ##  ##  ##  ##  ##  ##  ##  ##  ##  ##  ##  ##  ##  ## [1923_13]78	: ##  ##  ##  ##  ##  ##  ##  ##  ##  ##  ##  ##  ##  ##  ##  ##  ##  ##  ##  ##  ##  ##  ##  ##  ##  ##  ##  ##  ##  ##  ##  ##  ##  ##  ##  ##  ##  ##  ##  ##  ##  ##  ##  ##  ##  ##  ##  ##  ##  ##  ##  ##  ##  ##  ##  ##  ##  ##  ##  ##  ##  ##  ##  ##  ##  ##  ##  ##  ##  ##  ##  ##  ##  ##  ##  ##  ##  --  1  1  ##  ##  ##  ##  ##  ##  ##  ##  ##  ##  ##  ##  ##  ##  ##  ##  ##  ##  ##  ##  ##  ##  ## [1956_13]79	: ##  ##  ##  ##  ##  ##  ##  ##  ##  ##  ##  ##  ##  ##  ##  ##  ##  ##  ##  ##  ##  ##  ##  ##  ##  ##  ##  ##  ##  ##  ##  ##  ##  ##  ##  ##  ##  ##  ##  ##  ##  ##  ##  ##  ##  ##  ##  ##  ##  ##  ##  ##  ##  ##  ##  ##  ##  ##  ##  ##  ##  ##  ##  ##  ##  ##  ##  ##  ##  ##  ##  ##  ##  ##  ##  ##  ##  1  --  2  ##  ##  ##  ##  ##  ##  ##  ##  ##  ##  ##  ##  ##  ##  ##  ##  ##  ##  ##  ##  ##  ##  ## [2028_13]80	: ##  ##  ##  ##  ##  ##  ##  ##  ##  ##  ##  ##  ##  ##  ##  ##  ##  ##  ##  ##  ##  ##  ##  ##  ##  ##  ##  ##  ##  ##  ##  ##  ##  ##  ##  ##  ##  ##  ##  ##  ##  ##  ##  ##  ##  ##  ##  ##  ##  ##  ##  ##  ##  ##  ##  ##  ##  ##  ##  ##  ##  ##  ##  ##  ##  ##  ##  ##  ##  ##  ##  ##  ##  ##  ##  ##  ##  1  2  --  ##  ##  ##  ##  ##  ##  ##  ##  ##  ##  ##  ##  ##  ##  ##  ##  ##  ##  ##  ##  ##  ##  ## [2044_14]81	: ##  ##  ##  ##  ##  ##  ##  ##  ##  ##  ##  ##  ##  ##  ##  ##  ##  ##  ##  ##  ##  ##  ##  ##  ##  ##  ##  ##  ##  ##  ##  ##  ##  ##  ##  ##  ##  ##  ##  ##  ##  ##  ##  ##  ##  ##  ##  ##  ##  ##  ##  ##  ##  ##  ##  ##  ##  ##  ##  ##  ##  ##  ##  ##  ##  ##  ##  ##  ##  ##  ##  ##  ##  ##  ##  ##  ##  ##  ##  ##  --  3  4  5  4  5  ##  ##  ##  ##  ##  ##  ##  ##  ##  ##  ##  ##  ##  ##  ##  ##  ## [2042_14]82	: ##  ##  ##  ##  ##  ##  ##  ##  ##  ##  ##  ##  ##  ##  ##  ##  ##  ##  ##  ##  ##  ##  ##  ##  ##  ##  ##  ##  ##  ##  ##  ##  ##  ##  ##  ##  ##  ##  ##  ##  ##  ##  ##  ##  ##  ##  ##  ##  ##  ##  ##  ##  ##  ##  ##  ##  ##  ##  ##  ##  ##  ##  ##  ##  ##  ##  ##  ##  ##  ##  ##  ##  ##  ##  ##  ##  ##  ##  ##  ##  3  --  1  2  1  2  ##  ##  ##  ##  ##  ##  ##  ##  ##  ##  ##  ##  ##  ##  ##  ##  ## [2850_14]83	: ##  ##  ##  ##  ##  ##  ##  ##  ##  ##  ##  ##  ##  ##  ##  ##  ##  ##  ##  ##  ##  ##  ##  ##  ##  ##  ##  ##  ##  ##  ##  ##  ##  ##  ##  ##  ##  ##  ##  ##  ##  ##  ##  ##  ##  ##  ##  ##  ##  ##  ##  ##  ##  ##  ##  ##  ##  ##  ##  ##  ##  ##  ##  ##  ##  ##  ##  ##  ##  ##  ##  ##  ##  ##  ##  ##  ##  ##  ##  ##  4  1  --  3  2  1  ##  ##  ##  ##  ##  ##  ##  ##  ##  ##  ##  ##  ##  ##  ##  ##  ## [2848_14]84	: ##  ##  ##  ##  ##  ##  ##  ##  ##  ##  ##  ##  ##  ##  ##  ##  ##  ##  ##  ##  ##  ##  ##  ##  ##  ##  ##  ##  ##  ##  ##  ##  ##  ##  ##  ##  ##  ##  ##  ##  ##  ##  ##  ##  ##  ##  ##  ##  ##  ##  ##  ##  ##  ##  ##  ##  ##  ##  ##  ##  ##  ##  ##  ##  ##  ##  ##  ##  ##  ##  ##  ##  ##  ##  ##  ##  ##  ##  ##  ##  5  2  3  --  3  4  ##  ##  ##  ##  ##  ##  ##  ##  ##  ##  ##  ##  ##  ##  ##  ##  ## [2040_14]85	: ##  ##  ##  ##  ##  ##  ##  ##  ##  ##  ##  ##  ##  ##  ##  ##  ##  ##  ##  ##  ##  ##  ##  ##  ##  ##  ##  ##  ##  ##  ##  ##  ##  ##  ##  ##  ##  ##  ##  ##  ##  ##  ##  ##  ##  ##  ##  ##  ##  ##  ##  ##  ##  ##  ##  ##  ##  ##  ##  ##  ##  ##  ##  ##  ##  ##  ##  ##  ##  ##  ##  ##  ##  ##  ##  ##  ##  ##  ##  ##  4  1  2  3  --  1  ##  ##  ##  ##  ##  ##  ##  ##  ##  ##  ##  ##  ##  ##  ##  ##  ## [2853_14]86	: ##  ##  ##  ##  ##  ##  ##  ##  ##  ##  ##  ##  ##  ##  ##  ##  ##  ##  ##  ##  ##  ##  ##  ##  ##  ##  ##  ##  ##  ##  ##  ##  ##  ##  ##  ##  ##  ##  ##  ##  ##  ##  ##  ##  ##  ##  ##  ##  ##  ##  ##  ##  ##  ##  ##  ##  ##  ##  ##  ##  ##  ##  ##  ##  ##  ##  ##  ##  ##  ##  ##  ##  ##  ##  ##  ##  ##  ##  ##  ##  5  2  1  4  1  --  ##  ##  ##  ##  ##  ##  ##  ##  ##  ##  ##  ##  ##  ##  ##  ##  ## [2004_15]87	: ##  ##  ##  ##  ##  ##  ##  ##  ##  ##  ##  ##  ##  ##  ##  ##  ##  ##  ##  ##  ##  ##  ##  ##  ##  ##  ##  ##  ##  ##  ##  ##  ##  ##  ##  ##  ##  ##  ##  ##  ##  ##  ##  ##  ##  ##  ##  ##  ##  ##  ##  ##  ##  ##  ##  ##  ##  ##  ##  ##  ##  ##  ##  ##  ##  ##  ##  ##  ##  ##  ##  ##  ##  ##  ##  ##  ##  ##  ##  ##  ##  ##  ##  ##  ##  ##  --  ##  ##  ##  ##  ##  ##  ##  ##  ##  ##  ##  ##  ##  ##  ##  ## [2267_16]88	: ##  ##  ##  ##  ##  ##  ##  ##  ##  ##  ##  ##  ##  ##  ##  ##  ##  ##  ##  ##  ##  ##  ##  ##  ##  ##  ##  ##  ##  ##  ##  ##  ##  ##  ##  9  8  10  7  ##  ##  ##  ##  ##  ##  ##  ##  ##  ##  ##  ##  ##  ##  ##  ##  ##  ##  ##  ##  ##  ##  ##  ##  ##  ##  ##  ##  ##  ##  ##  ##  ##  ##  ##  ##  ##  ##  ##  ##  ##  ##  ##  ##  ##  ##  ##  ##  --  2  3  3  ##  ##  ##  ##  ##  ##  ##  ##  ##  ##  ##  ## [2268_16]89	: ##  ##  ##  ##  ##  ##  ##  ##  ##  ##  ##  ##  ##  ##  ##  ##  ##  ##  ##  ##  ##  ##  ##  ##  ##  ##  ##  ##  ##  ##  ##  ##  ##  ##  ##  11  10  12  9  ##  ##  ##  ##  ##  ##  ##  ##  ##  ##  ##  ##  ##  ##  ##  ##  ##  ##  ##  ##  ##  ##  ##  ##  ##  ##  ##  ##  ##  ##  ##  ##  ##  ##  ##  ##  ##  ##  ##  ##  ##  ##  ##  ##  ##  ##  ##  ##  2  --  1  1  ##  ##  ##  ##  ##  ##  ##  ##  ##  ##  ##  ## [2469_16]90	: ##  ##  ##  ##  ##  ##  ##  ##  ##  ##  ##  ##  ##  ##  ##  ##  ##  ##  ##  ##  ##  ##  ##  ##  ##  ##  ##  ##  ##  ##  ##  ##  ##  ##  ##  12  11  13  10  ##  ##  ##  ##  ##  ##  ##  ##  ##  ##  ##  ##  ##  ##  ##  ##  ##  ##  ##  ##  ##  ##  ##  ##  ##  ##  ##  ##  ##  ##  ##  ##  ##  ##  ##  ##  ##  ##  ##  ##  ##  ##  ##  ##  ##  ##  ##  ##  3  1  --  2  ##  ##  ##  ##  ##  ##  ##  ##  ##  ##  ##  ## [2269_16]91	: ##  ##  ##  ##  ##  ##  ##  ##  ##  ##  ##  ##  ##  ##  ##  ##  ##  ##  ##  ##  ##  ##  ##  ##  ##  ##  ##  ##  ##  ##  ##  ##  ##  ##  ##  12  11  13  10  ##  ##  ##  ##  ##  ##  ##  ##  ##  ##  ##  ##  ##  ##  ##  ##  ##  ##  ##  ##  ##  ##  ##  ##  ##  ##  ##  ##  ##  ##  ##  ##  ##  ##  ##  ##  ##  ##  ##  ##  ##  ##  ##  ##  ##  ##  ##  ##  3  1  2  --  ##  ##  ##  ##  ##  ##  ##  ##  ##  ##  ##  ## [2274_17]92	: ##  ##  ##  ##  ##  ##  ##  ##  ##  ##  ##  ##  ##  ##  ##  ##  ##  ##  ##  ##  ##  ##  ##  ##  ##  ##  ##  ##  ##  ##  ##  ##  ##  ##  ##  ##  ##  ##  ##  ##  ##  ##  ##  ##  ##  ##  ##  ##  ##  ##  ##  ##  ##  ##  ##  ##  ##  ##  ##  ##  ##  ##  ##  ##  ##  ##  ##  ##  ##  ##  ##  ##  ##  ##  ##  ##  ##  ##  ##  ##  ##  ##  ##  ##  ##  ##  ##  ##  ##  ##  ##  --  ##  ##  ##  ##  ##  ##  ##  ##  ##  ##  ## [2313_18]93	: ##  ##  ##  ##  ##  ##  ##  ##  ##  ##  ##  ##  ##  ##  ##  ##  ##  ##  ##  ##  ##  ##  ##  ##  ##  ##  ##  ##  ##  ##  ##  ##  ##  ##  ##  ##  ##  ##  ##  ##  ##  ##  ##  ##  ##  ##  ##  ##  ##  ##  ##  ##  ##  ##  ##  ##  ##  ##  ##  ##  ##  ##  ##  ##  ##  ##  ##  ##  ##  ##  ##  ##  ##  ##  ##  ##  ##  ##  ##  ##  ##  ##  ##  ##  ##  ##  ##  ##  ##  ##  ##  ##  --  ##  ##  ##  ##  ##  ##  ##  ##  ##  ## [2278_19]94	: ##  ##  ##  ##  ##  ##  ##  ##  ##  ##  ##  ##  ##  ##  ##  ##  ##  ##  ##  ##  ##  ##  ##  ##  ##  ##  ##  ##  ##  ##  ##  ##  ##  ##  ##  ##  ##  ##  ##  ##  ##  ##  ##  ##  ##  ##  ##  ##  ##  ##  ##  ##  ##  ##  ##  ##  ##  ##  ##  ##  ##  ##  ##  ##  ##  ##  ##  ##  ##  ##  ##  ##  ##  ##  ##  ##  ##  ##  ##  ##  ##  ##  ##  ##  ##  ##  ##  ##  ##  ##  ##  ##  ##  --  ##  ##  ##  ##  ##  ##  ##  ##  ## [2302_20]95	: ##  ##  ##  ##  ##  ##  ##  ##  ##  ##  ##  ##  ##  ##  ##  ##  ##  ##  ##  ##  ##  ##  ##  ##  ##  ##  ##  ##  ##  ##  ##  ##  ##  ##  ##  ##  ##  ##  ##  ##  ##  ##  ##  ##  ##  ##  ##  ##  ##  ##  ##  ##  ##  ##  ##  ##  ##  ##  ##  ##  ##  ##  ##  ##  ##  ##  ##  ##  ##  ##  ##  ##  ##  ##  ##  ##  ##  ##  ##  ##  ##  ##  ##  ##  ##  ##  ##  ##  ##  ##  ##  ##  ##  ##  --  ##  ##  ##  ##  ##  ##  ##  ## [2342_21]96	: ##  ##  ##  ##  ##  ##  ##  ##  ##  ##  ##  ##  ##  ##  ##  ##  ##  ##  ##  ##  ##  ##  ##  ##  ##  ##  ##  ##  ##  ##  ##  ##  ##  ##  ##  ##  ##  ##  ##  ##  ##  ##  ##  ##  ##  ##  ##  ##  ##  ##  ##  ##  ##  ##  ##  ##  ##  ##  ##  ##  ##  ##  ##  ##  ##  ##  ##  ##  ##  ##  ##  ##  ##  ##  ##  ##  ##  ##  ##  ##  ##  ##  ##  ##  ##  ##  ##  ##  ##  ##  ##  ##  ##  ##  ##  --  ##  ##  ##  ##  ##  ##  ## [2277_22]97	: ##  ##  ##  ##  ##  ##  ##  ##  ##  ##  ##  ##  ##  ##  ##  ##  ##  ##  ##  ##  ##  ##  ##  ##  ##  ##  ##  ##  ##  ##  ##  ##  ##  ##  ##  ##  ##  ##  ##  ##  ##  ##  ##  ##  ##  ##  ##  ##  ##  ##  ##  ##  ##  ##  ##  ##  ##  ##  ##  ##  ##  ##  ##  ##  ##  ##  ##  ##  ##  ##  ##  ##  ##  ##  ##  ##  ##  ##  ##  ##  ##  ##  ##  ##  ##  ##  ##  ##  ##  ##  ##  ##  ##  ##  ##  ##  --  ##  ##  ##  ##  ##  ## [2281_23]98	: ##  ##  ##  ##  ##  ##  ##  ##  ##  ##  ##  ##  ##  ##  ##  ##  ##  ##  ##  ##  ##  ##  ##  ##  ##  ##  ##  ##  ##  ##  ##  ##  ##  ##  ##  ##  ##  ##  ##  ##  ##  ##  ##  ##  ##  ##  ##  ##  ##  ##  ##  ##  ##  ##  ##  ##  ##  ##  ##  ##  ##  ##  ##  ##  ##  ##  ##  ##  ##  ##  ##  ##  ##  ##  ##  ##  ##  ##  ##  ##  ##  ##  ##  ##  ##  ##  ##  ##  ##  ##  ##  ##  ##  ##  ##  ##  ##  --  ##  ##  ##  ##  ## [2866_24]99	: ##  ##  ##  ##  ##  ##  ##  ##  ##  ##  ##  ##  ##  ##  ##  ##  ##  ##  ##  ##  ##  ##  ##  ##  ##  ##  ##  ##  ##  ##  ##  ##  ##  ##  ##  ##  ##  ##  ##  ##  ##  ##  ##  ##  ##  ##  ##  ##  ##  ##  ##  ##  ##  ##  ##  ##  ##  ##  ##  ##  ##  ##  ##  ##  ##  ##  ##  ##  ##  ##  ##  ##  ##  ##  ##  ##  ##  ##  ##  ##  ##  ##  ##  ##  ##  ##  ##  ##  ##  ##  ##  ##  ##  ##  ##  ##  ##  ##  --  1  ##  ##  ## [2865_24]100	: ##  ##  ##  ##  ##  ##  ##  ##  ##  ##  ##  ##  ##  ##  ##  ##  ##  ##  ##  ##  ##  ##  ##  ##  ##  ##  ##  ##  ##  ##  ##  ##  ##  ##  ##  ##  ##  ##  ##  ##  ##  ##  ##  ##  ##  ##  ##  ##  ##  ##  ##  ##  ##  ##  ##  ##  ##  ##  ##  ##  ##  ##  ##  ##  ##  ##  ##  ##  ##  ##  ##  ##  ##  ##  ##  ##  ##  ##  ##  ##  ##  ##  ##  ##  ##  ##  ##  ##  ##  ##  ##  ##  ##  ##  ##  ##  ##  ##  1  --  ##  ##  ## [2801_25]101	: ##  ##  ##  ##  ##  ##  ##  ##  ##  ##  ##  ##  ##  ##  ##  ##  ##  ##  ##  ##  ##  ##  ##  ##  ##  ##  ##  ##  ##  ##  ##  ##  ##  ##  ##  ##  ##  ##  ##  ##  ##  ##  ##  ##  ##  ##  ##  ##  ##  ##  ##  ##  ##  ##  ##  ##  ##  ##  ##  ##  ##  ##  ##  ##  ##  ##  ##  ##  ##  ##  ##  ##  ##  ##  ##  ##  ##  ##  ##  ##  ##  ##  ##  ##  ##  ##  ##  ##  ##  ##  ##  ##  ##  ##  ##  ##  ##  ##  ##  ##  --  ##  ## [2805_26]102	: ##  ##  ##  ##  ##  ##  ##  ##  ##  ##  ##  ##  ##  ##  ##  ##  ##  ##  ##  ##  ##  ##  ##  ##  ##  ##  ##  ##  ##  ##  ##  ##  ##  ##  3  ##  ##  ##  ##  ##  ##  ##  ##  ##  ##  ##  ##  ##  ##  ##  ##  ##  ##  ##  ##  ##  ##  ##  ##  ##  ##  ##  ##  ##  ##  ##  ##  ##  ##  ##  ##  ##  ##  ##  ##  ##  ##  ##  ##  ##  ##  ##  ##  ##  ##  ##  ##  ##  ##  ##  ##  ##  ##  ##  ##  ##  ##  ##  ##  ##  ##  --  8 [2800_27]103	: ##  ##  ##  ##  ##  ##  ##  ##  ##  ##  ##  ##  ##  ##  ##  ##  ##  ##  ##  ##  ##  ##  ##  ##  ##  ##  ##  ##  ##  ##  ##  ##  ##  ##  7  ##  ##  ##  ##  ##  ##  ##  ##  ##  ##  ##  ##  ##  ##  ##  ##  ##  ##  ##  ##  ##  ##  ##  ##  ##  ##  ##  ##  ##  ##  ##  ##  ##  ##  ##  ##  ##  ##  ##  ##  ##  ##  ##  ##  ##  ##  ##  ##  ##  ##  ##  ##  ##  ##  ##  ##  ##  ##  ##  ##  ##  ##  ##  ##  ##  ##  8  -- DIFFERENCE MATRIX pos:   			  1  2  3  4  5  6  7  8  9  10  11  12  13  14  15  16  17  18  19  20  21  22  23  24  25  26  27  28  29  30  31  32  33  34  35  36  37  38  39  40  41  42  43  44  45  46  47  48  49  50  51  52  53  54  55  56  57  58  59  60  61  62  63  64  65  66  67  68  69  70  71  72  73  74  75  76  77  78  79  80  81  82  83  84  85  86  87  88  89  90  91  92  93  94  95  96  97  98  99  100  101  102  103 [2440_1]1 	: --  0  0  0  0  0  0  0  0  0  0  0  0  0  0  0  0  0  0  ##  ##  ##  ##  ##  ##  ##  ##  ##  ##  ##  ##  ##  ##  ##  ##  ##  ##  ##  ##  ##  ##  ##  ##  ##  ##  ##  ##  ##  ##  ##  ##  ##  ##  ##  ##  ##  ##  ##  ##  ##  ##  ##  ##  ##  ##  ##  ##  ##  ##  ##  ##  ##  ##  ##  ##  ##  ##  ##  ##  ##  ##  ##  ##  ##  ##  ##  ##  ##  ##  ##  ##  ##  ##  ##  ##  ##  ##  ##  ##  ##  ##  ##  ## [1945_1]2 	: 0  --  0  0  0  0  0  0  0  0  0  0  0  0  0  0  0  0  0  ##  ##  ##  ##  ##  ##  ##  ##  ##  ##  ##  ##  ##  ##  ##  ##  ##  ##  ##  ##  ##  ##  ##  ##  ##  ##  ##  ##  ##  ##  ##  ##  ##  ##  ##  ##  ##  ##  ##  ##  ##  ##  ##  ##  ##  ##  ##  ##  ##  ##  ##  ##  ##  ##  ##  ##  ##  ##  ##  ##  ##  ##  ##  ##  ##  ##  ##  ##  ##  ##  ##  ##  ##  ##  ##  ##  ##  ##  ##  ##  ##  ##  ##  ## [836_1]3 	: 0  0  --  0  0  0  0  0  0  0  0  0  0  0  0  0  0  0  0  ##  ##  ##  ##  ##  ##  ##  ##  ##  ##  ##  ##  ##  ##  ##  ##  ##  ##  ##  ##  ##  ##  ##  ##  ##  ##  ##  ##  ##  ##  ##  ##  ##  ##  ##  ##  ##  ##  ##  ##  ##  ##  ##  ##  ##  ##  ##  ##  ##  ##  ##  ##  ##  ##  ##  ##  ##  ##  ##  ##  ##  ##  ##  ##  ##  ##  ##  ##  ##  ##  ##  ##  ##  ##  ##  ##  ##  ##  ##  ##  ##  ##  ##  ## [2219_1]4 	: 0  0  0  --  0  0  0  0  0  0  0  0  0  0  0  0  0  0  0  ##  ##  ##  ##  ##  ##  ##  ##  ##  ##  ##  ##  ##  ##  ##  ##  ##  ##  ##  ##  ##  ##  ##  ##  ##  ##  ##  ##  ##  ##  ##  ##  ##  ##  ##  ##  ##  ##  ##  ##  ##  ##  ##  ##  ##  ##  ##  ##  ##  ##  ##  ##  ##  ##  ##  ##  ##  ##  ##  ##  ##  ##  ##  ##  ##  ##  ##  ##  ##  ##  ##  ##  ##  ##  ##  ##  ##  ##  ##  ##  ##  ##  ##  ## [1339_1]5 	: 0  0  0  0  --  0  0  0  0  0  0  0  0  0  0  0  0  0  0  ##  ##  ##  ##  ##  ##  ##  ##  ##  ##  ##  ##  ##  ##  ##  ##  ##  ##  ##  ##  ##  ##  ##  ##  ##  ##  ##  ##  ##  ##  ##  ##  ##  ##  ##  ##  ##  ##  ##  ##  ##  ##  ##  ##  ##  ##  ##  ##  ##  ##  ##  ##  ##  ##  ##  ##  ##  ##  ##  ##  ##  ##  ##  ##  ##  ##  ##  ##  ##  ##  ##  ##  ##  ##  ##  ##  ##  ##  ##  ##  ##  ##  ##  ## [1939_1]6 	: 0  0  0  0  0  --  0  0  0  0  0  0  0  0  0  0  0  0  0  ##  ##  ##  ##  ##  ##  ##  ##  ##  ##  ##  ##  ##  ##  ##  ##  ##  ##  ##  ##  ##  ##  ##  ##  ##  ##  ##  ##  ##  ##  ##  ##  ##  ##  ##  ##  ##  ##  ##  ##  ##  ##  ##  ##  ##  ##  ##  ##  ##  ##  ##  ##  ##  ##  ##  ##  ##  ##  ##  ##  ##  ##  ##  ##  ##  ##  ##  ##  ##  ##  ##  ##  ##  ##  ##  ##  ##  ##  ##  ##  ##  ##  ##  ## [2794_1]7 	: 0  0  0  0  0  0  --  0  0  0  0  0  0  0  0  0  0  0  0  ##  ##  ##  ##  ##  ##  ##  ##  ##  ##  ##  ##  ##  ##  ##  ##  ##  ##  ##  ##  ##  ##  ##  ##  ##  ##  ##  ##  ##  ##  ##  ##  ##  ##  ##  ##  ##  ##  ##  ##  ##  ##  ##  ##  ##  ##  ##  ##  ##  ##  ##  ##  ##  ##  ##  ##  ##  ##  ##  ##  ##  ##  ##  ##  ##  ##  ##  ##  ##  ##  ##  ##  ##  ##  ##  ##  ##  ##  ##  ##  ##  ##  ##  ## [825_1]8 	: 0  0  0  0  0  0  0  --  0  0  0  0  0  0  0  0  0  0  0  ##  ##  ##  ##  ##  ##  ##  ##  ##  ##  ##  ##  ##  ##  ##  ##  ##  ##  ##  ##  ##  ##  ##  ##  ##  ##  ##  ##  ##  ##  ##  ##  ##  ##  ##  ##  ##  ##  ##  ##  ##  ##  ##  ##  ##  ##  ##  ##  ##  ##  ##  ##  ##  ##  ##  ##  ##  ##  ##  ##  ##  ##  ##  ##  ##  ##  ##  ##  ##  ##  ##  ##  ##  ##  ##  ##  ##  ##  ##  ##  ##  ##  ##  ## [2911_1]9 	: 0  0  0  0  0  0  0  0  --  0  0  0  0  0  0  0  0  0  0  ##  ##  ##  ##  ##  ##  ##  ##  ##  ##  ##  ##  ##  ##  ##  ##  ##  ##  ##  ##  ##  ##  ##  ##  ##  ##  ##  ##  ##  ##  ##  ##  ##  ##  ##  ##  ##  ##  ##  ##  ##  ##  ##  ##  ##  ##  ##  ##  ##  ##  ##  ##  ##  ##  ##  ##  ##  ##  ##  ##  ##  ##  ##  ##  ##  ##  ##  ##  ##  ##  ##  ##  ##  ##  ##  ##  ##  ##  ##  ##  ##  ##  ##  ## [1327_1]10	: 0  0  0  0  0  0  0  0  0  --  0  0  0  0  0  0  0  0  0  ##  ##  ##  ##  ##  ##  ##  ##  ##  ##  ##  ##  ##  ##  ##  ##  ##  ##  ##  ##  ##  ##  ##  ##  ##  ##  ##  ##  ##  ##  ##  ##  ##  ##  ##  ##  ##  ##  ##  ##  ##  ##  ##  ##  ##  ##  ##  ##  ##  ##  ##  ##  ##  ##  ##  ##  ##  ##  ##  ##  ##  ##  ##  ##  ##  ##  ##  ##  ##  ##  ##  ##  ##  ##  ##  ##  ##  ##  ##  ##  ##  ##  ##  ## [1332_1]11	: 0  0  0  0  0  0  0  0  0  0  --  0  0  0  0  0  0  0  0  ##  ##  ##  ##  ##  ##  ##  ##  ##  ##  ##  ##  ##  ##  ##  ##  ##  ##  ##  ##  ##  ##  ##  ##  ##  ##  ##  ##  ##  ##  ##  ##  ##  ##  ##  ##  ##  ##  ##  ##  ##  ##  ##  ##  ##  ##  ##  ##  ##  ##  ##  ##  ##  ##  ##  ##  ##  ##  ##  ##  ##  ##  ##  ##  ##  ##  ##  ##  ##  ##  ##  ##  ##  ##  ##  ##  ##  ##  ##  ##  ##  ##  ##  ## [2788_1]12	: 0  0  0  0  0  0  0  0  0  0  0  --  0  0  0  -1  1  0  0  ##  ##  ##  ##  ##  ##  ##  ##  ##  ##  ##  ##  ##  ##  ##  ##  ##  ##  ##  ##  ##  ##  ##  ##  ##  ##  ##  ##  ##  ##  ##  ##  ##  ##  ##  ##  ##  ##  ##  ##  ##  ##  ##  ##  ##  ##  ##  ##  ##  ##  ##  ##  ##  ##  ##  ##  ##  ##  ##  ##  ##  ##  ##  ##  ##  ##  ##  ##  ##  ##  ##  ##  ##  ##  ##  ##  ##  ##  ##  ##  ##  ##  ##  ## [2796_1]13	: 0  0  0  0  0  0  0  0  0  0  0  0  --  0  0  -1  1  0  0  ##  ##  ##  ##  ##  ##  ##  ##  ##  ##  ##  ##  ##  ##  ##  ##  ##  ##  ##  ##  ##  ##  ##  ##  ##  ##  ##  ##  ##  ##  ##  ##  ##  ##  ##  ##  ##  ##  ##  ##  ##  ##  ##  ##  ##  ##  ##  ##  ##  ##  ##  ##  ##  ##  ##  ##  ##  ##  ##  ##  ##  ##  ##  ##  ##  ##  ##  ##  ##  ##  ##  ##  ##  ##  ##  ##  ##  ##  ##  ##  ##  ##  ##  ## [2445_1]14	: 0  0  0  0  0  0  0  0  0  0  0  0  0  --  0  0  0  0  0  ##  ##  ##  ##  ##  ##  ##  ##  ##  ##  ##  ##  ##  ##  ##  ##  ##  ##  ##  ##  ##  ##  ##  ##  ##  ##  ##  ##  ##  ##  ##  ##  ##  ##  ##  ##  ##  ##  ##  ##  ##  ##  ##  ##  ##  ##  ##  ##  ##  ##  ##  ##  ##  ##  ##  ##  ##  ##  ##  ##  ##  ##  ##  ##  ##  ##  ##  ##  ##  ##  ##  ##  ##  ##  ##  ##  ##  ##  ##  ##  ##  ##  ##  ## [2871_1]15	: 0  0  0  0  0  0  0  0  0  0  0  0  0  0  --  0  0  0  0  ##  ##  ##  ##  ##  ##  ##  ##  ##  ##  ##  ##  ##  ##  ##  ##  ##  ##  ##  ##  ##  ##  ##  ##  ##  ##  ##  ##  ##  ##  ##  ##  ##  ##  ##  ##  ##  ##  ##  ##  ##  ##  ##  ##  ##  ##  ##  ##  ##  ##  ##  ##  ##  ##  ##  ##  ##  ##  ##  ##  ##  ##  ##  ##  ##  ##  ##  ##  ##  ##  ##  ##  ##  ##  ##  ##  ##  ##  ##  ##  ##  ##  ##  ## [2787_1]16	: 0  0  0  0  0  0  0  0  0  0  0  -1  -1  0  0  --  0  0  0  ##  ##  ##  ##  ##  ##  ##  ##  ##  ##  ##  ##  ##  ##  ##  ##  ##  ##  ##  ##  ##  ##  ##  ##  ##  ##  ##  ##  ##  ##  ##  ##  ##  ##  ##  ##  ##  ##  ##  ##  ##  ##  ##  ##  ##  ##  ##  ##  ##  ##  ##  ##  ##  ##  ##  ##  ##  ##  ##  ##  ##  ##  ##  ##  ##  ##  ##  ##  ##  ##  ##  ##  ##  ##  ##  ##  ##  ##  ##  ##  ##  ##  ##  ## [2860_1]17	: 0  0  0  0  0  0  0  0  0  0  0  1  1  0  0  0  --  0  0  ##  ##  ##  ##  ##  ##  ##  ##  ##  ##  ##  ##  ##  ##  ##  ##  ##  ##  ##  ##  ##  ##  ##  ##  ##  ##  ##  ##  ##  ##  ##  ##  ##  ##  ##  ##  ##  ##  ##  ##  ##  ##  ##  ##  ##  ##  ##  ##  ##  ##  ##  ##  ##  ##  ##  ##  ##  ##  ##  ##  ##  ##  ##  ##  ##  ##  ##  ##  ##  ##  ##  ##  ##  ##  ##  ##  ##  ##  ##  ##  ##  ##  ##  ## [1953_1]18	: 0  0  0  0  0  0  0  0  0  0  0  0  0  0  0  0  0  --  0  ##  ##  ##  ##  ##  ##  ##  ##  ##  ##  ##  ##  ##  ##  ##  ##  ##  ##  ##  ##  ##  ##  ##  ##  ##  ##  ##  ##  ##  ##  ##  ##  ##  ##  ##  ##  ##  ##  ##  ##  ##  ##  ##  ##  ##  ##  ##  ##  ##  ##  ##  ##  ##  ##  ##  ##  ##  ##  ##  ##  ##  ##  ##  ##  ##  ##  ##  ##  ##  ##  ##  ##  ##  ##  ##  ##  ##  ##  ##  ##  ##  ##  ##  ## [835_1]19	: 0  0  0  0  0  0  0  0  0  0  0  0  0  0  0  0  0  0  --  ##  ##  ##  ##  ##  ##  ##  ##  ##  ##  ##  ##  ##  ##  ##  ##  ##  ##  ##  ##  ##  ##  ##  ##  ##  ##  ##  ##  ##  ##  ##  ##  ##  ##  ##  ##  ##  ##  ##  ##  ##  ##  ##  ##  ##  ##  ##  ##  ##  ##  ##  ##  ##  ##  ##  ##  ##  ##  ##  ##  ##  ##  ##  ##  ##  ##  ##  ##  ##  ##  ##  ##  ##  ##  ##  ##  ##  ##  ##  ##  ##  ##  ##  ## [1311_2]20	: ##  ##  ##  ##  ##  ##  ##  ##  ##  ##  ##  ##  ##  ##  ##  ##  ##  ##  ##  --  0  0  0  0  0  0  ##  ##  ##  ##  ##  ##  ##  ##  ##  ##  ##  ##  ##  ##  ##  ##  ##  ##  ##  ##  ##  ##  ##  ##  ##  ##  ##  ##  ##  ##  ##  ##  ##  ##  ##  ##  ##  ##  ##  ##  ##  ##  ##  ##  ##  ##  ##  ##  ##  ##  ##  ##  ##  ##  ##  ##  ##  ##  ##  ##  ##  ##  ##  ##  ##  ##  ##  ##  ##  ##  ##  ##  ##  ##  ##  ##  ## [2180_2]21	: ##  ##  ##  ##  ##  ##  ##  ##  ##  ##  ##  ##  ##  ##  ##  ##  ##  ##  ##  0  --  0  0  0  0  0  ##  ##  ##  ##  ##  ##  ##  ##  ##  ##  ##  ##  ##  ##  ##  ##  ##  ##  ##  ##  ##  ##  ##  ##  ##  ##  ##  ##  ##  ##  ##  ##  ##  ##  ##  ##  ##  ##  ##  ##  ##  ##  ##  ##  ##  ##  ##  ##  ##  ##  ##  ##  ##  ##  ##  ##  ##  ##  ##  ##  ##  ##  ##  ##  ##  ##  ##  ##  ##  ##  ##  ##  ##  ##  ##  ##  ## [2187_2]22	: ##  ##  ##  ##  ##  ##  ##  ##  ##  ##  ##  ##  ##  ##  ##  ##  ##  ##  ##  0  0  --  0  0  0  0  ##  ##  ##  ##  ##  ##  ##  ##  ##  ##  ##  ##  ##  ##  ##  ##  ##  ##  ##  ##  ##  ##  ##  ##  ##  ##  ##  ##  ##  ##  ##  ##  ##  ##  ##  ##  ##  ##  ##  ##  ##  ##  ##  ##  ##  ##  ##  ##  ##  ##  ##  ##  ##  ##  ##  ##  ##  ##  ##  ##  ##  ##  ##  ##  ##  ##  ##  ##  ##  ##  ##  ##  ##  ##  ##  ##  ## [2333_2]23	: ##  ##  ##  ##  ##  ##  ##  ##  ##  ##  ##  ##  ##  ##  ##  ##  ##  ##  ##  0  0  0  --  0  0  0  ##  ##  ##  ##  ##  ##  ##  ##  ##  ##  ##  ##  ##  ##  ##  ##  ##  ##  ##  ##  ##  ##  ##  ##  ##  ##  ##  ##  ##  ##  ##  ##  ##  ##  ##  ##  ##  ##  ##  ##  ##  ##  ##  ##  ##  ##  ##  ##  ##  ##  ##  ##  ##  ##  ##  ##  ##  ##  ##  ##  ##  ##  ##  ##  ##  ##  ##  ##  ##  ##  ##  ##  ##  ##  ##  ##  ## [2390_2]24	: ##  ##  ##  ##  ##  ##  ##  ##  ##  ##  ##  ##  ##  ##  ##  ##  ##  ##  ##  0  0  0  0  --  0  0  ##  ##  ##  ##  ##  ##  ##  ##  ##  ##  ##  ##  ##  ##  ##  ##  ##  ##  ##  ##  ##  ##  ##  ##  ##  ##  ##  ##  ##  ##  ##  ##  ##  ##  ##  ##  ##  ##  ##  ##  ##  ##  ##  ##  ##  ##  ##  ##  ##  ##  ##  ##  ##  ##  ##  ##  ##  ##  ##  ##  ##  ##  ##  ##  ##  ##  ##  ##  ##  ##  ##  ##  ##  ##  ##  ##  ## [2382_2]25	: ##  ##  ##  ##  ##  ##  ##  ##  ##  ##  ##  ##  ##  ##  ##  ##  ##  ##  ##  0  0  0  0  0  --  0  ##  ##  ##  ##  ##  ##  ##  ##  ##  ##  ##  ##  ##  ##  ##  ##  ##  ##  ##  ##  ##  ##  ##  ##  ##  ##  ##  ##  ##  ##  ##  ##  ##  ##  ##  ##  ##  ##  ##  ##  ##  ##  ##  ##  ##  ##  ##  ##  ##  ##  ##  ##  ##  ##  ##  ##  ##  ##  ##  ##  ##  ##  ##  ##  ##  ##  ##  ##  ##  ##  ##  ##  ##  ##  ##  ##  ## [2353_2]26	: ##  ##  ##  ##  ##  ##  ##  ##  ##  ##  ##  ##  ##  ##  ##  ##  ##  ##  ##  0  0  0  0  0  0  --  ##  ##  ##  ##  ##  ##  ##  ##  ##  ##  ##  ##  ##  ##  ##  ##  ##  ##  ##  ##  ##  ##  ##  ##  ##  ##  ##  ##  ##  ##  ##  ##  ##  ##  ##  ##  ##  ##  ##  ##  ##  ##  ##  ##  ##  ##  ##  ##  ##  ##  ##  ##  ##  ##  ##  ##  ##  ##  ##  ##  ##  ##  ##  ##  ##  ##  ##  ##  ##  ##  ##  ##  ##  ##  ##  ##  ## [1207_3]27	: ##  ##  ##  ##  ##  ##  ##  ##  ##  ##  ##  ##  ##  ##  ##  ##  ##  ##  ##  ##  ##  ##  ##  ##  ##  ##  --  0  0  0  0  0  0  0  ##  ##  ##  ##  ##  ##  ##  ##  ##  ##  ##  ##  ##  ##  ##  ##  ##  ##  ##  ##  ##  ##  ##  ##  ##  ##  ##  ##  ##  ##  ##  ##  ##  ##  ##  ##  ##  ##  ##  ##  ##  ##  ##  ##  ##  ##  ##  ##  ##  ##  ##  ##  ##  ##  ##  ##  ##  ##  ##  ##  ##  ##  ##  ##  ##  ##  ##  ##  ## [2275_3]28	: ##  ##  ##  ##  ##  ##  ##  ##  ##  ##  ##  ##  ##  ##  ##  ##  ##  ##  ##  ##  ##  ##  ##  ##  ##  ##  0  --  0  0  0  0  0  0  ##  ##  ##  ##  ##  ##  ##  ##  ##  ##  ##  ##  ##  ##  ##  ##  ##  ##  ##  ##  ##  ##  ##  ##  ##  ##  ##  ##  ##  ##  ##  ##  ##  ##  ##  ##  ##  ##  ##  ##  ##  ##  ##  ##  ##  ##  ##  ##  ##  ##  ##  ##  ##  ##  ##  ##  ##  ##  ##  ##  ##  ##  ##  ##  ##  ##  ##  ##  ## [2287_3]29	: ##  ##  ##  ##  ##  ##  ##  ##  ##  ##  ##  ##  ##  ##  ##  ##  ##  ##  ##  ##  ##  ##  ##  ##  ##  ##  0  0  --  0  0  0  0  0  ##  ##  ##  ##  ##  ##  ##  ##  ##  ##  ##  ##  ##  ##  ##  ##  ##  ##  ##  ##  ##  ##  ##  ##  ##  ##  ##  ##  ##  ##  ##  ##  ##  ##  ##  ##  ##  ##  ##  ##  ##  ##  ##  ##  ##  ##  ##  ##  ##  ##  ##  ##  ##  ##  ##  ##  ##  ##  ##  ##  ##  ##  ##  ##  ##  ##  ##  ##  ## [2463_3]30	: ##  ##  ##  ##  ##  ##  ##  ##  ##  ##  ##  ##  ##  ##  ##  ##  ##  ##  ##  ##  ##  ##  ##  ##  ##  ##  0  0  0  --  0  0  0  0  ##  ##  ##  ##  ##  ##  ##  ##  ##  ##  ##  ##  ##  ##  ##  ##  ##  ##  ##  ##  ##  ##  ##  ##  ##  ##  ##  ##  ##  ##  ##  ##  ##  ##  ##  ##  ##  ##  ##  ##  ##  ##  ##  ##  ##  ##  ##  ##  ##  ##  ##  ##  ##  ##  ##  ##  ##  ##  ##  ##  ##  ##  ##  ##  ##  ##  ##  ##  ## [2878_3]31	: ##  ##  ##  ##  ##  ##  ##  ##  ##  ##  ##  ##  ##  ##  ##  ##  ##  ##  ##  ##  ##  ##  ##  ##  ##  ##  0  0  0  0  --  0  0  0  ##  ##  ##  ##  ##  ##  ##  ##  ##  ##  ##  ##  ##  ##  ##  ##  ##  ##  ##  ##  ##  ##  ##  ##  ##  ##  ##  ##  ##  ##  ##  ##  ##  ##  ##  ##  ##  ##  ##  ##  ##  ##  ##  ##  ##  ##  ##  ##  ##  ##  ##  ##  ##  ##  ##  ##  ##  ##  ##  ##  ##  ##  ##  ##  ##  ##  ##  ##  ## [2883_3]32	: ##  ##  ##  ##  ##  ##  ##  ##  ##  ##  ##  ##  ##  ##  ##  ##  ##  ##  ##  ##  ##  ##  ##  ##  ##  ##  0  0  0  0  0  --  0  0  ##  ##  ##  ##  ##  ##  ##  ##  ##  ##  ##  ##  ##  ##  ##  ##  ##  ##  ##  ##  ##  ##  ##  ##  ##  ##  ##  ##  ##  ##  ##  ##  ##  ##  ##  ##  ##  ##  ##  ##  ##  ##  ##  ##  ##  ##  ##  ##  ##  ##  ##  ##  ##  ##  ##  ##  ##  ##  ##  ##  ##  ##  ##  ##  ##  ##  ##  ##  ## [2286_3]33	: ##  ##  ##  ##  ##  ##  ##  ##  ##  ##  ##  ##  ##  ##  ##  ##  ##  ##  ##  ##  ##  ##  ##  ##  ##  ##  0  0  0  0  0  0  --  0  ##  ##  ##  ##  ##  ##  ##  ##  ##  ##  ##  ##  ##  ##  ##  ##  ##  ##  ##  ##  ##  ##  ##  ##  ##  ##  ##  ##  ##  ##  ##  ##  ##  ##  ##  ##  ##  ##  ##  ##  ##  ##  ##  ##  ##  ##  ##  ##  ##  ##  ##  ##  ##  ##  ##  ##  ##  ##  ##  ##  ##  ##  ##  ##  ##  ##  ##  ##  ## [2814_3]34	: ##  ##  ##  ##  ##  ##  ##  ##  ##  ##  ##  ##  ##  ##  ##  ##  ##  ##  ##  ##  ##  ##  ##  ##  ##  ##  0  0  0  0  0  0  0  --  ##  ##  ##  ##  ##  ##  ##  ##  ##  ##  ##  ##  ##  ##  ##  ##  ##  ##  ##  ##  ##  ##  ##  ##  ##  ##  ##  ##  ##  ##  ##  ##  ##  ##  ##  ##  ##  ##  ##  ##  ##  ##  ##  ##  ##  ##  ##  ##  ##  ##  ##  ##  ##  ##  ##  ##  ##  ##  ##  ##  ##  ##  ##  ##  ##  ##  ##  ##  ## [2226_4]35	: ##  ##  ##  ##  ##  ##  ##  ##  ##  ##  ##  ##  ##  ##  ##  ##  ##  ##  ##  ##  ##  ##  ##  ##  ##  ##  ##  ##  ##  ##  ##  ##  ##  ##  --  ##  ##  ##  ##  ##  ##  ##  ##  ##  ##  ##  ##  ##  ##  ##  ##  ##  ##  ##  ##  ##  ##  ##  ##  ##  ##  ##  ##  ##  ##  ##  ##  ##  ##  ##  ##  ##  ##  ##  ##  ##  ##  ##  ##  ##  ##  ##  ##  ##  ##  ##  ##  ##  ##  ##  ##  ##  ##  ##  ##  ##  ##  ##  ##  ##  ##  0  0 [840_5]36	: ##  ##  ##  ##  ##  ##  ##  ##  ##  ##  ##  ##  ##  ##  ##  ##  ##  ##  ##  ##  ##  ##  ##  ##  ##  ##  ##  ##  ##  ##  ##  ##  ##  ##  ##  --  0  0  0  ##  ##  ##  ##  ##  ##  ##  ##  ##  ##  ##  ##  ##  ##  ##  ##  ##  ##  ##  ##  ##  ##  ##  ##  ##  ##  ##  ##  ##  ##  ##  ##  ##  ##  ##  ##  ##  ##  ##  ##  ##  ##  ##  ##  ##  ##  ##  ##  0  1  1  1  ##  ##  ##  ##  ##  ##  ##  ##  ##  ##  ##  ## [2778_5]37	: ##  ##  ##  ##  ##  ##  ##  ##  ##  ##  ##  ##  ##  ##  ##  ##  ##  ##  ##  ##  ##  ##  ##  ##  ##  ##  ##  ##  ##  ##  ##  ##  ##  ##  ##  0  --  0  0  ##  ##  ##  ##  ##  ##  ##  ##  ##  ##  ##  ##  ##  ##  ##  ##  ##  ##  ##  ##  ##  ##  ##  ##  ##  ##  ##  ##  ##  ##  ##  ##  ##  ##  ##  ##  ##  ##  ##  ##  ##  ##  ##  ##  ##  ##  ##  ##  0  1  1  1  ##  ##  ##  ##  ##  ##  ##  ##  ##  ##  ##  ## [842_5]38	: ##  ##  ##  ##  ##  ##  ##  ##  ##  ##  ##  ##  ##  ##  ##  ##  ##  ##  ##  ##  ##  ##  ##  ##  ##  ##  ##  ##  ##  ##  ##  ##  ##  ##  ##  0  0  --  0  ##  ##  ##  ##  ##  ##  ##  ##  ##  ##  ##  ##  ##  ##  ##  ##  ##  ##  ##  ##  ##  ##  ##  ##  ##  ##  ##  ##  ##  ##  ##  ##  ##  ##  ##  ##  ##  ##  ##  ##  ##  ##  ##  ##  ##  ##  ##  ##  0  1  1  1  ##  ##  ##  ##  ##  ##  ##  ##  ##  ##  ##  ## [2900_5]39	: ##  ##  ##  ##  ##  ##  ##  ##  ##  ##  ##  ##  ##  ##  ##  ##  ##  ##  ##  ##  ##  ##  ##  ##  ##  ##  ##  ##  ##  ##  ##  ##  ##  ##  ##  0  0  0  --  ##  ##  ##  ##  ##  ##  ##  ##  ##  ##  ##  ##  ##  ##  ##  ##  ##  ##  ##  ##  ##  ##  ##  ##  ##  ##  ##  ##  ##  ##  ##  ##  ##  ##  ##  ##  ##  ##  ##  ##  ##  ##  ##  ##  ##  ##  ##  ##  0  0  0  0  ##  ##  ##  ##  ##  ##  ##  ##  ##  ##  ##  ## [1874_6]40	: ##  ##  ##  ##  ##  ##  ##  ##  ##  ##  ##  ##  ##  ##  ##  ##  ##  ##  ##  ##  ##  ##  ##  ##  ##  ##  ##  ##  ##  ##  ##  ##  ##  ##  ##  ##  ##  ##  ##  --  0  0  0  0  0  0  0  0  0  0  0  ##  ##  ##  ##  ##  ##  ##  ##  ##  ##  ##  ##  ##  ##  ##  ##  ##  ##  ##  ##  ##  ##  ##  ##  ##  ##  ##  ##  ##  ##  ##  ##  ##  ##  ##  ##  ##  ##  ##  ##  ##  ##  ##  ##  ##  ##  ##  ##  ##  ##  ##  ## [846_6]41	: ##  ##  ##  ##  ##  ##  ##  ##  ##  ##  ##  ##  ##  ##  ##  ##  ##  ##  ##  ##  ##  ##  ##  ##  ##  ##  ##  ##  ##  ##  ##  ##  ##  ##  ##  ##  ##  ##  ##  0  --  0  0  1  0  0  0  0  0  0  0  ##  ##  ##  ##  ##  ##  ##  ##  ##  ##  ##  ##  ##  ##  ##  ##  ##  ##  ##  ##  ##  ##  ##  ##  ##  ##  ##  ##  ##  ##  ##  ##  ##  ##  ##  ##  ##  ##  ##  ##  ##  ##  ##  ##  ##  ##  ##  ##  ##  ##  ##  ## [1943_6]42	: ##  ##  ##  ##  ##  ##  ##  ##  ##  ##  ##  ##  ##  ##  ##  ##  ##  ##  ##  ##  ##  ##  ##  ##  ##  ##  ##  ##  ##  ##  ##  ##  ##  ##  ##  ##  ##  ##  ##  0  0  --  0  0  -2  0  0  0  0  0  0  ##  ##  ##  ##  ##  ##  ##  ##  ##  ##  ##  ##  ##  ##  ##  ##  ##  ##  ##  ##  ##  ##  ##  ##  ##  ##  ##  ##  ##  ##  ##  ##  ##  ##  ##  ##  ##  ##  ##  ##  ##  ##  ##  ##  ##  ##  ##  ##  ##  ##  ##  ## [1318_6]43	: ##  ##  ##  ##  ##  ##  ##  ##  ##  ##  ##  ##  ##  ##  ##  ##  ##  ##  ##  ##  ##  ##  ##  ##  ##  ##  ##  ##  ##  ##  ##  ##  ##  ##  ##  ##  ##  ##  ##  0  0  0  --  0  -2  0  0  0  0  0  0  ##  ##  ##  ##  ##  ##  ##  ##  ##  ##  ##  ##  ##  ##  ##  ##  ##  ##  ##  ##  ##  ##  ##  ##  ##  ##  ##  ##  ##  ##  ##  ##  ##  ##  ##  ##  ##  ##  ##  ##  ##  ##  ##  ##  ##  ##  ##  ##  ##  ##  ##  ## [1869_6]44	: ##  ##  ##  ##  ##  ##  ##  ##  ##  ##  ##  ##  ##  ##  ##  ##  ##  ##  ##  ##  ##  ##  ##  ##  ##  ##  ##  ##  ##  ##  ##  ##  ##  ##  ##  ##  ##  ##  ##  0  1  0  0  --  -1  1  -1  0  0  1  1  ##  ##  ##  ##  ##  ##  ##  ##  ##  ##  ##  ##  ##  ##  ##  ##  ##  ##  ##  ##  ##  ##  ##  ##  ##  ##  ##  ##  ##  ##  ##  ##  ##  ##  ##  ##  ##  ##  ##  ##  ##  ##  ##  ##  ##  ##  ##  ##  ##  ##  ##  ## [845_6]45	: ##  ##  ##  ##  ##  ##  ##  ##  ##  ##  ##  ##  ##  ##  ##  ##  ##  ##  ##  ##  ##  ##  ##  ##  ##  ##  ##  ##  ##  ##  ##  ##  ##  ##  ##  ##  ##  ##  ##  0  0  -2  -2  -1  --  0  -1  -1  0  0  1  ##  ##  ##  ##  ##  ##  ##  ##  ##  ##  ##  ##  ##  ##  ##  ##  ##  ##  ##  ##  ##  ##  ##  ##  ##  ##  ##  ##  ##  ##  ##  ##  ##  ##  ##  ##  ##  ##  ##  ##  ##  ##  ##  ##  ##  ##  ##  ##  ##  ##  ##  ## [860_6]46	: ##  ##  ##  ##  ##  ##  ##  ##  ##  ##  ##  ##  ##  ##  ##  ##  ##  ##  ##  ##  ##  ##  ##  ##  ##  ##  ##  ##  ##  ##  ##  ##  ##  ##  ##  ##  ##  ##  ##  0  0  0  0  1  0  --  0  0  1  0  0  ##  ##  ##  ##  ##  ##  ##  ##  ##  ##  ##  ##  ##  ##  ##  ##  ##  ##  ##  ##  ##  ##  ##  ##  ##  ##  ##  ##  ##  ##  ##  ##  ##  ##  ##  ##  ##  ##  ##  ##  ##  ##  ##  ##  ##  ##  ##  ##  ##  ##  ##  ## [1317_6]47	: ##  ##  ##  ##  ##  ##  ##  ##  ##  ##  ##  ##  ##  ##  ##  ##  ##  ##  ##  ##  ##  ##  ##  ##  ##  ##  ##  ##  ##  ##  ##  ##  ##  ##  ##  ##  ##  ##  ##  0  0  0  0  -1  -1  0  --  0  0  0  0  ##  ##  ##  ##  ##  ##  ##  ##  ##  ##  ##  ##  ##  ##  ##  ##  ##  ##  ##  ##  ##  ##  ##  ##  ##  ##  ##  ##  ##  ##  ##  ##  ##  ##  ##  ##  ##  ##  ##  ##  ##  ##  ##  ##  ##  ##  ##  ##  ##  ##  ##  ## [1871_6]48	: ##  ##  ##  ##  ##  ##  ##  ##  ##  ##  ##  ##  ##  ##  ##  ##  ##  ##  ##  ##  ##  ##  ##  ##  ##  ##  ##  ##  ##  ##  ##  ##  ##  ##  ##  ##  ##  ##  ##  0  0  0  0  0  -1  0  0  --  0  0  0  ##  ##  ##  ##  ##  ##  ##  ##  ##  ##  ##  ##  ##  ##  ##  ##  ##  ##  ##  ##  ##  ##  ##  ##  ##  ##  ##  ##  ##  ##  ##  ##  ##  ##  ##  ##  ##  ##  ##  ##  ##  ##  ##  ##  ##  ##  ##  ##  ##  ##  ##  ## [2170_6]49	: ##  ##  ##  ##  ##  ##  ##  ##  ##  ##  ##  ##  ##  ##  ##  ##  ##  ##  ##  ##  ##  ##  ##  ##  ##  ##  ##  ##  ##  ##  ##  ##  ##  ##  ##  ##  ##  ##  ##  0  0  0  0  0  0  1  0  0  --  0  0  ##  ##  ##  ##  ##  ##  ##  ##  ##  ##  ##  ##  ##  ##  ##  ##  ##  ##  ##  ##  ##  ##  ##  ##  ##  ##  ##  ##  ##  ##  ##  ##  ##  ##  ##  ##  ##  ##  ##  ##  ##  ##  ##  ##  ##  ##  ##  ##  ##  ##  ##  ## [2169_6]50	: ##  ##  ##  ##  ##  ##  ##  ##  ##  ##  ##  ##  ##  ##  ##  ##  ##  ##  ##  ##  ##  ##  ##  ##  ##  ##  ##  ##  ##  ##  ##  ##  ##  ##  ##  ##  ##  ##  ##  0  0  0  0  1  0  0  0  0  0  --  0  ##  ##  ##  ##  ##  ##  ##  ##  ##  ##  ##  ##  ##  ##  ##  ##  ##  ##  ##  ##  ##  ##  ##  ##  ##  ##  ##  ##  ##  ##  ##  ##  ##  ##  ##  ##  ##  ##  ##  ##  ##  ##  ##  ##  ##  ##  ##  ##  ##  ##  ##  ## [2173_6]51	: ##  ##  ##  ##  ##  ##  ##  ##  ##  ##  ##  ##  ##  ##  ##  ##  ##  ##  ##  ##  ##  ##  ##  ##  ##  ##  ##  ##  ##  ##  ##  ##  ##  ##  ##  ##  ##  ##  ##  0  0  0  0  1  1  0  0  0  0  0  --  ##  ##  ##  ##  ##  ##  ##  ##  ##  ##  ##  ##  ##  ##  ##  ##  ##  ##  ##  ##  ##  ##  ##  ##  ##  ##  ##  ##  ##  ##  ##  ##  ##  ##  ##  ##  ##  ##  ##  ##  ##  ##  ##  ##  ##  ##  ##  ##  ##  ##  ##  ## [1309_7]52	: ##  ##  ##  ##  ##  ##  ##  ##  ##  ##  ##  ##  ##  ##  ##  ##  ##  ##  ##  ##  ##  ##  ##  ##  ##  ##  ##  ##  ##  ##  ##  ##  ##  ##  ##  ##  ##  ##  ##  ##  ##  ##  ##  ##  ##  ##  ##  ##  ##  ##  ##  --  0  0  0  ##  ##  ##  ##  ##  ##  ##  ##  ##  ##  ##  ##  ##  ##  ##  ##  ##  ##  ##  ##  ##  ##  ##  ##  ##  ##  ##  ##  ##  ##  ##  ##  ##  ##  ##  ##  ##  ##  ##  ##  ##  ##  ##  ##  ##  ##  ##  ## [2448_7]53	: ##  ##  ##  ##  ##  ##  ##  ##  ##  ##  ##  ##  ##  ##  ##  ##  ##  ##  ##  ##  ##  ##  ##  ##  ##  ##  ##  ##  ##  ##  ##  ##  ##  ##  ##  ##  ##  ##  ##  ##  ##  ##  ##  ##  ##  ##  ##  ##  ##  ##  ##  0  --  0  0  ##  ##  ##  ##  ##  ##  ##  ##  ##  ##  ##  ##  ##  ##  ##  ##  ##  ##  ##  ##  ##  ##  ##  ##  ##  ##  ##  ##  ##  ##  ##  ##  ##  ##  ##  ##  ##  ##  ##  ##  ##  ##  ##  ##  ##  ##  ##  ## [2442_7]54	: ##  ##  ##  ##  ##  ##  ##  ##  ##  ##  ##  ##  ##  ##  ##  ##  ##  ##  ##  ##  ##  ##  ##  ##  ##  ##  ##  ##  ##  ##  ##  ##  ##  ##  ##  ##  ##  ##  ##  ##  ##  ##  ##  ##  ##  ##  ##  ##  ##  ##  ##  0  0  --  0  ##  ##  ##  ##  ##  ##  ##  ##  ##  ##  ##  ##  ##  ##  ##  ##  ##  ##  ##  ##  ##  ##  ##  ##  ##  ##  ##  ##  ##  ##  ##  ##  ##  ##  ##  ##  ##  ##  ##  ##  ##  ##  ##  ##  ##  ##  ##  ## [2449_7]55	: ##  ##  ##  ##  ##  ##  ##  ##  ##  ##  ##  ##  ##  ##  ##  ##  ##  ##  ##  ##  ##  ##  ##  ##  ##  ##  ##  ##  ##  ##  ##  ##  ##  ##  ##  ##  ##  ##  ##  ##  ##  ##  ##  ##  ##  ##  ##  ##  ##  ##  ##  0  0  0  --  ##  ##  ##  ##  ##  ##  ##  ##  ##  ##  ##  ##  ##  ##  ##  ##  ##  ##  ##  ##  ##  ##  ##  ##  ##  ##  ##  ##  ##  ##  ##  ##  ##  ##  ##  ##  ##  ##  ##  ##  ##  ##  ##  ##  ##  ##  ##  ## [1202_8]56	: ##  ##  ##  ##  ##  ##  ##  ##  ##  ##  ##  ##  ##  ##  ##  ##  ##  ##  ##  ##  ##  ##  ##  ##  ##  ##  ##  ##  ##  ##  ##  ##  ##  ##  ##  ##  ##  ##  ##  ##  ##  ##  ##  ##  ##  ##  ##  ##  ##  ##  ##  ##  ##  ##  ##  --  0  0  0  0  0  0  ##  ##  ##  ##  ##  ##  ##  ##  ##  ##  ##  ##  ##  ##  ##  ##  ##  ##  ##  ##  ##  ##  ##  ##  ##  ##  ##  ##  ##  ##  ##  ##  ##  ##  ##  ##  ##  ##  ##  ##  ## [1203_8]57	: ##  ##  ##  ##  ##  ##  ##  ##  ##  ##  ##  ##  ##  ##  ##  ##  ##  ##  ##  ##  ##  ##  ##  ##  ##  ##  ##  ##  ##  ##  ##  ##  ##  ##  ##  ##  ##  ##  ##  ##  ##  ##  ##  ##  ##  ##  ##  ##  ##  ##  ##  ##  ##  ##  ##  0  --  0  0  0  0  0  ##  ##  ##  ##  ##  ##  ##  ##  ##  ##  ##  ##  ##  ##  ##  ##  ##  ##  ##  ##  ##  ##  ##  ##  ##  ##  ##  ##  ##  ##  ##  ##  ##  ##  ##  ##  ##  ##  ##  ##  ## [1946_8]58	: ##  ##  ##  ##  ##  ##  ##  ##  ##  ##  ##  ##  ##  ##  ##  ##  ##  ##  ##  ##  ##  ##  ##  ##  ##  ##  ##  ##  ##  ##  ##  ##  ##  ##  ##  ##  ##  ##  ##  ##  ##  ##  ##  ##  ##  ##  ##  ##  ##  ##  ##  ##  ##  ##  ##  0  0  --  0  0  0  0  ##  ##  ##  ##  ##  ##  ##  ##  ##  ##  ##  ##  ##  ##  ##  ##  ##  ##  ##  ##  ##  ##  ##  ##  ##  ##  ##  ##  ##  ##  ##  ##  ##  ##  ##  ##  ##  ##  ##  ##  ## [1198_8]59	: ##  ##  ##  ##  ##  ##  ##  ##  ##  ##  ##  ##  ##  ##  ##  ##  ##  ##  ##  ##  ##  ##  ##  ##  ##  ##  ##  ##  ##  ##  ##  ##  ##  ##  ##  ##  ##  ##  ##  ##  ##  ##  ##  ##  ##  ##  ##  ##  ##  ##  ##  ##  ##  ##  ##  0  0  0  --  0  0  0  ##  ##  ##  ##  ##  ##  ##  ##  ##  ##  ##  ##  ##  ##  ##  ##  ##  ##  ##  ##  ##  ##  ##  ##  ##  ##  ##  ##  ##  ##  ##  ##  ##  ##  ##  ##  ##  ##  ##  ##  ## [1957_8]60	: ##  ##  ##  ##  ##  ##  ##  ##  ##  ##  ##  ##  ##  ##  ##  ##  ##  ##  ##  ##  ##  ##  ##  ##  ##  ##  ##  ##  ##  ##  ##  ##  ##  ##  ##  ##  ##  ##  ##  ##  ##  ##  ##  ##  ##  ##  ##  ##  ##  ##  ##  ##  ##  ##  ##  0  0  0  0  --  0  0  ##  ##  ##  ##  ##  ##  ##  ##  ##  ##  ##  ##  ##  ##  ##  ##  ##  ##  ##  ##  ##  ##  ##  ##  ##  ##  ##  ##  ##  ##  ##  ##  ##  ##  ##  ##  ##  ##  ##  ##  ## [2920_8]61	: ##  ##  ##  ##  ##  ##  ##  ##  ##  ##  ##  ##  ##  ##  ##  ##  ##  ##  ##  ##  ##  ##  ##  ##  ##  ##  ##  ##  ##  ##  ##  ##  ##  ##  ##  ##  ##  ##  ##  ##  ##  ##  ##  ##  ##  ##  ##  ##  ##  ##  ##  ##  ##  ##  ##  0  0  0  0  0  --  1  ##  ##  ##  ##  ##  ##  ##  ##  ##  ##  ##  ##  ##  ##  ##  ##  ##  ##  ##  ##  ##  ##  ##  ##  ##  ##  ##  ##  ##  ##  ##  ##  ##  ##  ##  ##  ##  ##  ##  ##  ## [2476_8]62	: ##  ##  ##  ##  ##  ##  ##  ##  ##  ##  ##  ##  ##  ##  ##  ##  ##  ##  ##  ##  ##  ##  ##  ##  ##  ##  ##  ##  ##  ##  ##  ##  ##  ##  ##  ##  ##  ##  ##  ##  ##  ##  ##  ##  ##  ##  ##  ##  ##  ##  ##  ##  ##  ##  ##  0  0  0  0  0  1  --  ##  ##  ##  ##  ##  ##  ##  ##  ##  ##  ##  ##  ##  ##  ##  ##  ##  ##  ##  ##  ##  ##  ##  ##  ##  ##  ##  ##  ##  ##  ##  ##  ##  ##  ##  ##  ##  ##  ##  ##  ## [862_9]63	: ##  ##  ##  ##  ##  ##  ##  ##  ##  ##  ##  ##  ##  ##  ##  ##  ##  ##  ##  ##  ##  ##  ##  ##  ##  ##  ##  ##  ##  ##  ##  ##  ##  ##  ##  ##  ##  ##  ##  ##  ##  ##  ##  ##  ##  ##  ##  ##  ##  ##  ##  ##  ##  ##  ##  ##  ##  ##  ##  ##  ##  ##  --  ##  ##  ##  ##  ##  ##  ##  ##  ##  ##  ##  ##  ##  ##  ##  ##  ##  ##  ##  ##  ##  ##  ##  ##  ##  ##  ##  ##  ##  ##  ##  ##  ##  ##  ##  ##  ##  ##  ##  ## [2031_10]64	: ##  ##  ##  ##  ##  ##  ##  ##  ##  ##  ##  ##  ##  ##  ##  ##  ##  ##  ##  ##  ##  ##  ##  ##  ##  ##  ##  ##  ##  ##  ##  ##  ##  ##  ##  ##  ##  ##  ##  ##  ##  ##  ##  ##  ##  ##  ##  ##  ##  ##  ##  ##  ##  ##  ##  ##  ##  ##  ##  ##  ##  ##  ##  --  0  ##  ##  ##  ##  ##  ##  ##  ##  ##  ##  ##  ##  ##  ##  ##  ##  ##  ##  ##  ##  ##  ##  ##  ##  ##  ##  ##  ##  ##  ##  ##  ##  ##  ##  ##  ##  ##  ## [2026_10]65	: ##  ##  ##  ##  ##  ##  ##  ##  ##  ##  ##  ##  ##  ##  ##  ##  ##  ##  ##  ##  ##  ##  ##  ##  ##  ##  ##  ##  ##  ##  ##  ##  ##  ##  ##  ##  ##  ##  ##  ##  ##  ##  ##  ##  ##  ##  ##  ##  ##  ##  ##  ##  ##  ##  ##  ##  ##  ##  ##  ##  ##  ##  ##  0  --  ##  ##  ##  ##  ##  ##  ##  ##  ##  ##  ##  ##  ##  ##  ##  ##  ##  ##  ##  ##  ##  ##  ##  ##  ##  ##  ##  ##  ##  ##  ##  ##  ##  ##  ##  ##  ##  ## [2786_11]66	: ##  ##  ##  ##  ##  ##  ##  ##  ##  ##  ##  ##  ##  ##  ##  ##  ##  ##  ##  ##  ##  ##  ##  ##  ##  ##  ##  ##  ##  ##  ##  ##  ##  ##  ##  ##  ##  ##  ##  ##  ##  ##  ##  ##  ##  ##  ##  ##  ##  ##  ##  ##  ##  ##  ##  ##  ##  ##  ##  ##  ##  ##  ##  ##  ##  --  0  ##  ##  ##  ##  ##  ##  ##  ##  ##  ##  ##  ##  ##  ##  ##  ##  ##  ##  ##  ##  ##  ##  ##  ##  ##  ##  ##  ##  ##  ##  ##  ##  ##  ##  ##  ## [2323_11]67	: ##  ##  ##  ##  ##  ##  ##  ##  ##  ##  ##  ##  ##  ##  ##  ##  ##  ##  ##  ##  ##  ##  ##  ##  ##  ##  ##  ##  ##  ##  ##  ##  ##  ##  ##  ##  ##  ##  ##  ##  ##  ##  ##  ##  ##  ##  ##  ##  ##  ##  ##  ##  ##  ##  ##  ##  ##  ##  ##  ##  ##  ##  ##  ##  ##  0  --  ##  ##  ##  ##  ##  ##  ##  ##  ##  ##  ##  ##  ##  ##  ##  ##  ##  ##  ##  ##  ##  ##  ##  ##  ##  ##  ##  ##  ##  ##  ##  ##  ##  ##  ##  ## [2196_12]68	: ##  ##  ##  ##  ##  ##  ##  ##  ##  ##  ##  ##  ##  ##  ##  ##  ##  ##  ##  ##  ##  ##  ##  ##  ##  ##  ##  ##  ##  ##  ##  ##  ##  ##  ##  ##  ##  ##  ##  ##  ##  ##  ##  ##  ##  ##  ##  ##  ##  ##  ##  ##  ##  ##  ##  ##  ##  ##  ##  ##  ##  ##  ##  ##  ##  ##  ##  --  0  0  0  0  0  0  0  0  0  ##  ##  ##  ##  ##  ##  ##  ##  ##  ##  ##  ##  ##  ##  ##  ##  ##  ##  ##  ##  ##  ##  ##  ##  ##  ## [2806_12]69	: ##  ##  ##  ##  ##  ##  ##  ##  ##  ##  ##  ##  ##  ##  ##  ##  ##  ##  ##  ##  ##  ##  ##  ##  ##  ##  ##  ##  ##  ##  ##  ##  ##  ##  ##  ##  ##  ##  ##  ##  ##  ##  ##  ##  ##  ##  ##  ##  ##  ##  ##  ##  ##  ##  ##  ##  ##  ##  ##  ##  ##  ##  ##  ##  ##  ##  ##  0  --  0  0  0  0  -2  0  0  0  ##  ##  ##  ##  ##  ##  ##  ##  ##  ##  ##  ##  ##  ##  ##  ##  ##  ##  ##  ##  ##  ##  ##  ##  ##  ## [2194_12]70	: ##  ##  ##  ##  ##  ##  ##  ##  ##  ##  ##  ##  ##  ##  ##  ##  ##  ##  ##  ##  ##  ##  ##  ##  ##  ##  ##  ##  ##  ##  ##  ##  ##  ##  ##  ##  ##  ##  ##  ##  ##  ##  ##  ##  ##  ##  ##  ##  ##  ##  ##  ##  ##  ##  ##  ##  ##  ##  ##  ##  ##  ##  ##  ##  ##  ##  ##  0  0  --  0  0  0  0  0  0  0  ##  ##  ##  ##  ##  ##  ##  ##  ##  ##  ##  ##  ##  ##  ##  ##  ##  ##  ##  ##  ##  ##  ##  ##  ##  ## [2201_12]71	: ##  ##  ##  ##  ##  ##  ##  ##  ##  ##  ##  ##  ##  ##  ##  ##  ##  ##  ##  ##  ##  ##  ##  ##  ##  ##  ##  ##  ##  ##  ##  ##  ##  ##  ##  ##  ##  ##  ##  ##  ##  ##  ##  ##  ##  ##  ##  ##  ##  ##  ##  ##  ##  ##  ##  ##  ##  ##  ##  ##  ##  ##  ##  ##  ##  ##  ##  0  0  0  --  0  0  0  0  0  0  ##  ##  ##  ##  ##  ##  ##  ##  ##  ##  ##  ##  ##  ##  ##  ##  ##  ##  ##  ##  ##  ##  ##  ##  ##  ## [2202_12]72	: ##  ##  ##  ##  ##  ##  ##  ##  ##  ##  ##  ##  ##  ##  ##  ##  ##  ##  ##  ##  ##  ##  ##  ##  ##  ##  ##  ##  ##  ##  ##  ##  ##  ##  ##  ##  ##  ##  ##  ##  ##  ##  ##  ##  ##  ##  ##  ##  ##  ##  ##  ##  ##  ##  ##  ##  ##  ##  ##  ##  ##  ##  ##  ##  ##  ##  ##  0  0  0  0  --  0  0  0  0  0  ##  ##  ##  ##  ##  ##  ##  ##  ##  ##  ##  ##  ##  ##  ##  ##  ##  ##  ##  ##  ##  ##  ##  ##  ##  ## [2171_12]73	: ##  ##  ##  ##  ##  ##  ##  ##  ##  ##  ##  ##  ##  ##  ##  ##  ##  ##  ##  ##  ##  ##  ##  ##  ##  ##  ##  ##  ##  ##  ##  ##  ##  ##  ##  ##  ##  ##  ##  ##  ##  ##  ##  ##  ##  ##  ##  ##  ##  ##  ##  ##  ##  ##  ##  ##  ##  ##  ##  ##  ##  ##  ##  ##  ##  ##  ##  0  0  0  0  0  --  -2  0  0  0  ##  ##  ##  ##  ##  ##  ##  ##  ##  ##  ##  ##  ##  ##  ##  ##  ##  ##  ##  ##  ##  ##  ##  ##  ##  ## [2225_12]74	: ##  ##  ##  ##  ##  ##  ##  ##  ##  ##  ##  ##  ##  ##  ##  ##  ##  ##  ##  ##  ##  ##  ##  ##  ##  ##  ##  ##  ##  ##  ##  ##  ##  ##  ##  ##  ##  ##  ##  ##  ##  ##  ##  ##  ##  ##  ##  ##  ##  ##  ##  ##  ##  ##  ##  ##  ##  ##  ##  ##  ##  ##  ##  ##  ##  ##  ##  0  -2  0  0  0  -2  --  0  0  0  ##  ##  ##  ##  ##  ##  ##  ##  ##  ##  ##  ##  ##  ##  ##  ##  ##  ##  ##  ##  ##  ##  ##  ##  ##  ## [2224_12]75	: ##  ##  ##  ##  ##  ##  ##  ##  ##  ##  ##  ##  ##  ##  ##  ##  ##  ##  ##  ##  ##  ##  ##  ##  ##  ##  ##  ##  ##  ##  ##  ##  ##  ##  ##  ##  ##  ##  ##  ##  ##  ##  ##  ##  ##  ##  ##  ##  ##  ##  ##  ##  ##  ##  ##  ##  ##  ##  ##  ##  ##  ##  ##  ##  ##  ##  ##  0  0  0  0  0  0  0  --  0  0  ##  ##  ##  ##  ##  ##  ##  ##  ##  ##  ##  ##  ##  ##  ##  ##  ##  ##  ##  ##  ##  ##  ##  ##  ##  ## [2198_12]76	: ##  ##  ##  ##  ##  ##  ##  ##  ##  ##  ##  ##  ##  ##  ##  ##  ##  ##  ##  ##  ##  ##  ##  ##  ##  ##  ##  ##  ##  ##  ##  ##  ##  ##  ##  ##  ##  ##  ##  ##  ##  ##  ##  ##  ##  ##  ##  ##  ##  ##  ##  ##  ##  ##  ##  ##  ##  ##  ##  ##  ##  ##  ##  ##  ##  ##  ##  0  0  0  0  0  0  0  0  --  0  ##  ##  ##  ##  ##  ##  ##  ##  ##  ##  ##  ##  ##  ##  ##  ##  ##  ##  ##  ##  ##  ##  ##  ##  ##  ## [1312_12]77	: ##  ##  ##  ##  ##  ##  ##  ##  ##  ##  ##  ##  ##  ##  ##  ##  ##  ##  ##  ##  ##  ##  ##  ##  ##  ##  ##  ##  ##  ##  ##  ##  ##  ##  ##  ##  ##  ##  ##  ##  ##  ##  ##  ##  ##  ##  ##  ##  ##  ##  ##  ##  ##  ##  ##  ##  ##  ##  ##  ##  ##  ##  ##  ##  ##  ##  ##  0  0  0  0  0  0  0  0  0  --  ##  ##  ##  ##  ##  ##  ##  ##  ##  ##  ##  ##  ##  ##  ##  ##  ##  ##  ##  ##  ##  ##  ##  ##  ##  ## [1923_13]78	: ##  ##  ##  ##  ##  ##  ##  ##  ##  ##  ##  ##  ##  ##  ##  ##  ##  ##  ##  ##  ##  ##  ##  ##  ##  ##  ##  ##  ##  ##  ##  ##  ##  ##  ##  ##  ##  ##  ##  ##  ##  ##  ##  ##  ##  ##  ##  ##  ##  ##  ##  ##  ##  ##  ##  ##  ##  ##  ##  ##  ##  ##  ##  ##  ##  ##  ##  ##  ##  ##  ##  ##  ##  ##  ##  ##  ##  --  0  0  ##  ##  ##  ##  ##  ##  ##  ##  ##  ##  ##  ##  ##  ##  ##  ##  ##  ##  ##  ##  ##  ##  ## [1956_13]79	: ##  ##  ##  ##  ##  ##  ##  ##  ##  ##  ##  ##  ##  ##  ##  ##  ##  ##  ##  ##  ##  ##  ##  ##  ##  ##  ##  ##  ##  ##  ##  ##  ##  ##  ##  ##  ##  ##  ##  ##  ##  ##  ##  ##  ##  ##  ##  ##  ##  ##  ##  ##  ##  ##  ##  ##  ##  ##  ##  ##  ##  ##  ##  ##  ##  ##  ##  ##  ##  ##  ##  ##  ##  ##  ##  ##  ##  0  --  0  ##  ##  ##  ##  ##  ##  ##  ##  ##  ##  ##  ##  ##  ##  ##  ##  ##  ##  ##  ##  ##  ##  ## [2028_13]80	: ##  ##  ##  ##  ##  ##  ##  ##  ##  ##  ##  ##  ##  ##  ##  ##  ##  ##  ##  ##  ##  ##  ##  ##  ##  ##  ##  ##  ##  ##  ##  ##  ##  ##  ##  ##  ##  ##  ##  ##  ##  ##  ##  ##  ##  ##  ##  ##  ##  ##  ##  ##  ##  ##  ##  ##  ##  ##  ##  ##  ##  ##  ##  ##  ##  ##  ##  ##  ##  ##  ##  ##  ##  ##  ##  ##  ##  0  0  --  ##  ##  ##  ##  ##  ##  ##  ##  ##  ##  ##  ##  ##  ##  ##  ##  ##  ##  ##  ##  ##  ##  ## [2044_14]81	: ##  ##  ##  ##  ##  ##  ##  ##  ##  ##  ##  ##  ##  ##  ##  ##  ##  ##  ##  ##  ##  ##  ##  ##  ##  ##  ##  ##  ##  ##  ##  ##  ##  ##  ##  ##  ##  ##  ##  ##  ##  ##  ##  ##  ##  ##  ##  ##  ##  ##  ##  ##  ##  ##  ##  ##  ##  ##  ##  ##  ##  ##  ##  ##  ##  ##  ##  ##  ##  ##  ##  ##  ##  ##  ##  ##  ##  ##  ##  ##  --  0  0  1  0  0  ##  ##  ##  ##  ##  ##  ##  ##  ##  ##  ##  ##  ##  ##  ##  ##  ## [2042_14]82	: ##  ##  ##  ##  ##  ##  ##  ##  ##  ##  ##  ##  ##  ##  ##  ##  ##  ##  ##  ##  ##  ##  ##  ##  ##  ##  ##  ##  ##  ##  ##  ##  ##  ##  ##  ##  ##  ##  ##  ##  ##  ##  ##  ##  ##  ##  ##  ##  ##  ##  ##  ##  ##  ##  ##  ##  ##  ##  ##  ##  ##  ##  ##  ##  ##  ##  ##  ##  ##  ##  ##  ##  ##  ##  ##  ##  ##  ##  ##  ##  0  --  0  0  0  0  ##  ##  ##  ##  ##  ##  ##  ##  ##  ##  ##  ##  ##  ##  ##  ##  ## [2850_14]83	: ##  ##  ##  ##  ##  ##  ##  ##  ##  ##  ##  ##  ##  ##  ##  ##  ##  ##  ##  ##  ##  ##  ##  ##  ##  ##  ##  ##  ##  ##  ##  ##  ##  ##  ##  ##  ##  ##  ##  ##  ##  ##  ##  ##  ##  ##  ##  ##  ##  ##  ##  ##  ##  ##  ##  ##  ##  ##  ##  ##  ##  ##  ##  ##  ##  ##  ##  ##  ##  ##  ##  ##  ##  ##  ##  ##  ##  ##  ##  ##  0  0  --  0  0  0  ##  ##  ##  ##  ##  ##  ##  ##  ##  ##  ##  ##  ##  ##  ##  ##  ## [2848_14]84	: ##  ##  ##  ##  ##  ##  ##  ##  ##  ##  ##  ##  ##  ##  ##  ##  ##  ##  ##  ##  ##  ##  ##  ##  ##  ##  ##  ##  ##  ##  ##  ##  ##  ##  ##  ##  ##  ##  ##  ##  ##  ##  ##  ##  ##  ##  ##  ##  ##  ##  ##  ##  ##  ##  ##  ##  ##  ##  ##  ##  ##  ##  ##  ##  ##  ##  ##  ##  ##  ##  ##  ##  ##  ##  ##  ##  ##  ##  ##  ##  1  0  0  --  0  0  ##  ##  ##  ##  ##  ##  ##  ##  ##  ##  ##  ##  ##  ##  ##  ##  ## [2040_14]85	: ##  ##  ##  ##  ##  ##  ##  ##  ##  ##  ##  ##  ##  ##  ##  ##  ##  ##  ##  ##  ##  ##  ##  ##  ##  ##  ##  ##  ##  ##  ##  ##  ##  ##  ##  ##  ##  ##  ##  ##  ##  ##  ##  ##  ##  ##  ##  ##  ##  ##  ##  ##  ##  ##  ##  ##  ##  ##  ##  ##  ##  ##  ##  ##  ##  ##  ##  ##  ##  ##  ##  ##  ##  ##  ##  ##  ##  ##  ##  ##  0  0  0  0  --  0  ##  ##  ##  ##  ##  ##  ##  ##  ##  ##  ##  ##  ##  ##  ##  ##  ## [2853_14]86	: ##  ##  ##  ##  ##  ##  ##  ##  ##  ##  ##  ##  ##  ##  ##  ##  ##  ##  ##  ##  ##  ##  ##  ##  ##  ##  ##  ##  ##  ##  ##  ##  ##  ##  ##  ##  ##  ##  ##  ##  ##  ##  ##  ##  ##  ##  ##  ##  ##  ##  ##  ##  ##  ##  ##  ##  ##  ##  ##  ##  ##  ##  ##  ##  ##  ##  ##  ##  ##  ##  ##  ##  ##  ##  ##  ##  ##  ##  ##  ##  0  0  0  0  0  --  ##  ##  ##  ##  ##  ##  ##  ##  ##  ##  ##  ##  ##  ##  ##  ##  ## [2004_15]87	: ##  ##  ##  ##  ##  ##  ##  ##  ##  ##  ##  ##  ##  ##  ##  ##  ##  ##  ##  ##  ##  ##  ##  ##  ##  ##  ##  ##  ##  ##  ##  ##  ##  ##  ##  ##  ##  ##  ##  ##  ##  ##  ##  ##  ##  ##  ##  ##  ##  ##  ##  ##  ##  ##  ##  ##  ##  ##  ##  ##  ##  ##  ##  ##  ##  ##  ##  ##  ##  ##  ##  ##  ##  ##  ##  ##  ##  ##  ##  ##  ##  ##  ##  ##  ##  ##  --  ##  ##  ##  ##  ##  ##  ##  ##  ##  ##  ##  ##  ##  ##  ##  ## [2267_16]88	: ##  ##  ##  ##  ##  ##  ##  ##  ##  ##  ##  ##  ##  ##  ##  ##  ##  ##  ##  ##  ##  ##  ##  ##  ##  ##  ##  ##  ##  ##  ##  ##  ##  ##  ##  0  0  0  0  ##  ##  ##  ##  ##  ##  ##  ##  ##  ##  ##  ##  ##  ##  ##  ##  ##  ##  ##  ##  ##  ##  ##  ##  ##  ##  ##  ##  ##  ##  ##  ##  ##  ##  ##  ##  ##  ##  ##  ##  ##  ##  ##  ##  ##  ##  ##  ##  --  0  0  0  ##  ##  ##  ##  ##  ##  ##  ##  ##  ##  ##  ## [2268_16]89	: ##  ##  ##  ##  ##  ##  ##  ##  ##  ##  ##  ##  ##  ##  ##  ##  ##  ##  ##  ##  ##  ##  ##  ##  ##  ##  ##  ##  ##  ##  ##  ##  ##  ##  ##  1  1  1  0  ##  ##  ##  ##  ##  ##  ##  ##  ##  ##  ##  ##  ##  ##  ##  ##  ##  ##  ##  ##  ##  ##  ##  ##  ##  ##  ##  ##  ##  ##  ##  ##  ##  ##  ##  ##  ##  ##  ##  ##  ##  ##  ##  ##  ##  ##  ##  ##  0  --  0  0  ##  ##  ##  ##  ##  ##  ##  ##  ##  ##  ##  ## [2469_16]90	: ##  ##  ##  ##  ##  ##  ##  ##  ##  ##  ##  ##  ##  ##  ##  ##  ##  ##  ##  ##  ##  ##  ##  ##  ##  ##  ##  ##  ##  ##  ##  ##  ##  ##  ##  1  1  1  0  ##  ##  ##  ##  ##  ##  ##  ##  ##  ##  ##  ##  ##  ##  ##  ##  ##  ##  ##  ##  ##  ##  ##  ##  ##  ##  ##  ##  ##  ##  ##  ##  ##  ##  ##  ##  ##  ##  ##  ##  ##  ##  ##  ##  ##  ##  ##  ##  0  0  --  0  ##  ##  ##  ##  ##  ##  ##  ##  ##  ##  ##  ## [2269_16]91	: ##  ##  ##  ##  ##  ##  ##  ##  ##  ##  ##  ##  ##  ##  ##  ##  ##  ##  ##  ##  ##  ##  ##  ##  ##  ##  ##  ##  ##  ##  ##  ##  ##  ##  ##  1  1  1  0  ##  ##  ##  ##  ##  ##  ##  ##  ##  ##  ##  ##  ##  ##  ##  ##  ##  ##  ##  ##  ##  ##  ##  ##  ##  ##  ##  ##  ##  ##  ##  ##  ##  ##  ##  ##  ##  ##  ##  ##  ##  ##  ##  ##  ##  ##  ##  ##  0  0  0  --  ##  ##  ##  ##  ##  ##  ##  ##  ##  ##  ##  ## [2274_17]92	: ##  ##  ##  ##  ##  ##  ##  ##  ##  ##  ##  ##  ##  ##  ##  ##  ##  ##  ##  ##  ##  ##  ##  ##  ##  ##  ##  ##  ##  ##  ##  ##  ##  ##  ##  ##  ##  ##  ##  ##  ##  ##  ##  ##  ##  ##  ##  ##  ##  ##  ##  ##  ##  ##  ##  ##  ##  ##  ##  ##  ##  ##  ##  ##  ##  ##  ##  ##  ##  ##  ##  ##  ##  ##  ##  ##  ##  ##  ##  ##  ##  ##  ##  ##  ##  ##  ##  ##  ##  ##  ##  --  ##  ##  ##  ##  ##  ##  ##  ##  ##  ##  ## [2313_18]93	: ##  ##  ##  ##  ##  ##  ##  ##  ##  ##  ##  ##  ##  ##  ##  ##  ##  ##  ##  ##  ##  ##  ##  ##  ##  ##  ##  ##  ##  ##  ##  ##  ##  ##  ##  ##  ##  ##  ##  ##  ##  ##  ##  ##  ##  ##  ##  ##  ##  ##  ##  ##  ##  ##  ##  ##  ##  ##  ##  ##  ##  ##  ##  ##  ##  ##  ##  ##  ##  ##  ##  ##  ##  ##  ##  ##  ##  ##  ##  ##  ##  ##  ##  ##  ##  ##  ##  ##  ##  ##  ##  ##  --  ##  ##  ##  ##  ##  ##  ##  ##  ##  ## [2278_19]94	: ##  ##  ##  ##  ##  ##  ##  ##  ##  ##  ##  ##  ##  ##  ##  ##  ##  ##  ##  ##  ##  ##  ##  ##  ##  ##  ##  ##  ##  ##  ##  ##  ##  ##  ##  ##  ##  ##  ##  ##  ##  ##  ##  ##  ##  ##  ##  ##  ##  ##  ##  ##  ##  ##  ##  ##  ##  ##  ##  ##  ##  ##  ##  ##  ##  ##  ##  ##  ##  ##  ##  ##  ##  ##  ##  ##  ##  ##  ##  ##  ##  ##  ##  ##  ##  ##  ##  ##  ##  ##  ##  ##  ##  --  ##  ##  ##  ##  ##  ##  ##  ##  ## [2302_20]95	: ##  ##  ##  ##  ##  ##  ##  ##  ##  ##  ##  ##  ##  ##  ##  ##  ##  ##  ##  ##  ##  ##  ##  ##  ##  ##  ##  ##  ##  ##  ##  ##  ##  ##  ##  ##  ##  ##  ##  ##  ##  ##  ##  ##  ##  ##  ##  ##  ##  ##  ##  ##  ##  ##  ##  ##  ##  ##  ##  ##  ##  ##  ##  ##  ##  ##  ##  ##  ##  ##  ##  ##  ##  ##  ##  ##  ##  ##  ##  ##  ##  ##  ##  ##  ##  ##  ##  ##  ##  ##  ##  ##  ##  ##  --  ##  ##  ##  ##  ##  ##  ##  ## [2342_21]96	: ##  ##  ##  ##  ##  ##  ##  ##  ##  ##  ##  ##  ##  ##  ##  ##  ##  ##  ##  ##  ##  ##  ##  ##  ##  ##  ##  ##  ##  ##  ##  ##  ##  ##  ##  ##  ##  ##  ##  ##  ##  ##  ##  ##  ##  ##  ##  ##  ##  ##  ##  ##  ##  ##  ##  ##  ##  ##  ##  ##  ##  ##  ##  ##  ##  ##  ##  ##  ##  ##  ##  ##  ##  ##  ##  ##  ##  ##  ##  ##  ##  ##  ##  ##  ##  ##  ##  ##  ##  ##  ##  ##  ##  ##  ##  --  ##  ##  ##  ##  ##  ##  ## [2277_22]97	: ##  ##  ##  ##  ##  ##  ##  ##  ##  ##  ##  ##  ##  ##  ##  ##  ##  ##  ##  ##  ##  ##  ##  ##  ##  ##  ##  ##  ##  ##  ##  ##  ##  ##  ##  ##  ##  ##  ##  ##  ##  ##  ##  ##  ##  ##  ##  ##  ##  ##  ##  ##  ##  ##  ##  ##  ##  ##  ##  ##  ##  ##  ##  ##  ##  ##  ##  ##  ##  ##  ##  ##  ##  ##  ##  ##  ##  ##  ##  ##  ##  ##  ##  ##  ##  ##  ##  ##  ##  ##  ##  ##  ##  ##  ##  ##  --  ##  ##  ##  ##  ##  ## [2281_23]98	: ##  ##  ##  ##  ##  ##  ##  ##  ##  ##  ##  ##  ##  ##  ##  ##  ##  ##  ##  ##  ##  ##  ##  ##  ##  ##  ##  ##  ##  ##  ##  ##  ##  ##  ##  ##  ##  ##  ##  ##  ##  ##  ##  ##  ##  ##  ##  ##  ##  ##  ##  ##  ##  ##  ##  ##  ##  ##  ##  ##  ##  ##  ##  ##  ##  ##  ##  ##  ##  ##  ##  ##  ##  ##  ##  ##  ##  ##  ##  ##  ##  ##  ##  ##  ##  ##  ##  ##  ##  ##  ##  ##  ##  ##  ##  ##  ##  --  ##  ##  ##  ##  ## [2866_24]99	: ##  ##  ##  ##  ##  ##  ##  ##  ##  ##  ##  ##  ##  ##  ##  ##  ##  ##  ##  ##  ##  ##  ##  ##  ##  ##  ##  ##  ##  ##  ##  ##  ##  ##  ##  ##  ##  ##  ##  ##  ##  ##  ##  ##  ##  ##  ##  ##  ##  ##  ##  ##  ##  ##  ##  ##  ##  ##  ##  ##  ##  ##  ##  ##  ##  ##  ##  ##  ##  ##  ##  ##  ##  ##  ##  ##  ##  ##  ##  ##  ##  ##  ##  ##  ##  ##  ##  ##  ##  ##  ##  ##  ##  ##  ##  ##  ##  ##  --  0  ##  ##  ## [2865_24]100	: ##  ##  ##  ##  ##  ##  ##  ##  ##  ##  ##  ##  ##  ##  ##  ##  ##  ##  ##  ##  ##  ##  ##  ##  ##  ##  ##  ##  ##  ##  ##  ##  ##  ##  ##  ##  ##  ##  ##  ##  ##  ##  ##  ##  ##  ##  ##  ##  ##  ##  ##  ##  ##  ##  ##  ##  ##  ##  ##  ##  ##  ##  ##  ##  ##  ##  ##  ##  ##  ##  ##  ##  ##  ##  ##  ##  ##  ##  ##  ##  ##  ##  ##  ##  ##  ##  ##  ##  ##  ##  ##  ##  ##  ##  ##  ##  ##  ##  0  --  ##  ##  ## [2801_25]101	: ##  ##  ##  ##  ##  ##  ##  ##  ##  ##  ##  ##  ##  ##  ##  ##  ##  ##  ##  ##  ##  ##  ##  ##  ##  ##  ##  ##  ##  ##  ##  ##  ##  ##  ##  ##  ##  ##  ##  ##  ##  ##  ##  ##  ##  ##  ##  ##  ##  ##  ##  ##  ##  ##  ##  ##  ##  ##  ##  ##  ##  ##  ##  ##  ##  ##  ##  ##  ##  ##  ##  ##  ##  ##  ##  ##  ##  ##  ##  ##  ##  ##  ##  ##  ##  ##  ##  ##  ##  ##  ##  ##  ##  ##  ##  ##  ##  ##  ##  ##  --  ##  ## [2805_26]102	: ##  ##  ##  ##  ##  ##  ##  ##  ##  ##  ##  ##  ##  ##  ##  ##  ##  ##  ##  ##  ##  ##  ##  ##  ##  ##  ##  ##  ##  ##  ##  ##  ##  ##  0  ##  ##  ##  ##  ##  ##  ##  ##  ##  ##  ##  ##  ##  ##  ##  ##  ##  ##  ##  ##  ##  ##  ##  ##  ##  ##  ##  ##  ##  ##  ##  ##  ##  ##  ##  ##  ##  ##  ##  ##  ##  ##  ##  ##  ##  ##  ##  ##  ##  ##  ##  ##  ##  ##  ##  ##  ##  ##  ##  ##  ##  ##  ##  ##  ##  ##  --  0 [2800_27]103	: ##  ##  ##  ##  ##  ##  ##  ##  ##  ##  ##  ##  ##  ##  ##  ##  ##  ##  ##  ##  ##  ##  ##  ##  ##  ##  ##  ##  ##  ##  ##  ##  ##  ##  0  ##  ##  ##  ##  ##  ##  ##  ##  ##  ##  ##  ##  ##  ##  ##  ##  ##  ##  ##  ##  ##  ##  ##  ##  ##  ##  ##  ##  ##  ##  ##  ##  ##  ##  ##  ##  ##  ##  ##  ##  ##  ##  ##  ##  ##  ##  ##  ##  ##  ##  ##  ##  ##  ##  ##  ##  ##  ##  ##  ##  ##  ##  ##  ##  ##  ##  0  -- The total positive difference matrix is 19.0The total negative difference matrix is -14.0OUTGROUP WEIGHTS*** Network 11339_1 weigth = 0.050890585241730282219_1 weigth = 0.081424936386768451939_1 weigth = 0.086513994910941472794_1 weigth = 0.076335877862595422445_1 weigth = 0.002544529262086514835_1 weigth = 0.0025445292620865142787_1 weigth = 0.0025445292620865141332_1 weigth = 0.0025445292620865142911_1 weigth = 0.117048346055979652440_1 weigth = 0.132315521628498721945_1 weigth = 0.173027989821882942871_1 weigth = 0.02544529262086514836_1 weigth = 0.16793893129770993825_1 weigth = 0.0025445292620865141327_1 weigth = 0.050890585241730281953_1 weigth = 0.0025445292620865142860_1 weigth = 0.0025445292620865142788_1 weigth = 0.0101781170483460572796_1 weigth = 0.010178117048346057Total weight = 196.5Biggest outgroup probability is 1945_1 (0.17302798982188294)*** Network 22382_2 weigth = 0.0061728395061728392187_2 weigth = 0.0061728395061728391311_2 weigth = 0.308641975308641962180_2 weigth = 0.28395061728395062333_2 weigth = 0.160493827160493822353_2 weigth = 0.197530864197530852390_2 weigth = 0.037037037037037035Total weight = 81.0Biggest outgroup probability is 1311_2 (0.30864197530864196)*** Network 32814_3 weigth = 0.00308641975308641962883_3 weigth = 0.00308641975308641962275_3 weigth = 0.061728395061728391207_3 weigth = 0.290123456790123472287_3 weigth = 0.320987654320987642878_3 weigth = 0.28395061728395062286_3 weigth = 0.0308641975308641962463_3 weigth = 0.006172839506172839Total weight = 162.0Biggest outgroup probability is 2287_3 (0.32098765432098764)*** Network 42900_5 weigth = 0.0058823529411764705842_5 weigth = 0.0058823529411764705840_5 weigth = 0.22778_5 weigth = 0.188235294117647062269_16 weigth = 0.00588235294117647052267_16 weigth = 0.42352941176470592268_16 weigth = 0.164705882352941172469_16 weigth = 0.0058823529411764705Total weight = 85.0Biggest outgroup probability is 2267_16 (0.4235294117647059)*** Network 5845_6 weigth = 0.0076335877862595421318_6 weigth = 0.19847328244274811874_6 weigth = 0.106870229007633591869_6 weigth = 0.076335877862595421317_6 weigth = 0.122137404580152672170_6 weigth = 0.0610687022900763331871_6 weigth = 0.137404580152671762173_6 weigth = 0.007633587786259542860_6 weigth = 0.007633587786259542846_6 weigth = 0.244274809160305331943_6 weigth = 0.0229007633587786262169_6 weigth = 0.007633587786259542Total weight = 65.5Biggest outgroup probability is 846_6 (0.24427480916030533)*** Network 62442_7 weigth = 0.051309_7 weigth = 0.42448_7 weigth = 0.52449_7 weigth = 0.05Total weight = 10.0Biggest outgroup probability is 2448_7 (0.5)*** Network 72920_8 weigth = 0.011203_8 weigth = 0.021202_8 weigth = 0.31946_8 weigth = 0.541957_8 weigth = 0.011198_8 weigth = 0.112476_8 weigth = 0.01Total weight = 50.0Biggest outgroup probability is 1946_8 (0.54)*** Network 8862_9 weigth = 1.0Total weight = 2.0Biggest outgroup probability is 862_9 (1.0)*** Network 92031_10 weigth = 0.85714285714285712026_10 weigth = 0.14285714285714285Total weight = 3.5Biggest outgroup probability is 2031_10 (0.8571428571428571)*** Network 102786_11 weigth = 0.66666666666666662323_11 weigth = 0.3333333333333333Total weight = 1.5Biggest outgroup probability is 2786_11 (0.6666666666666666)*** Network 112225_12 weigth = 0.0215053763440860232202_12 weigth = 0.0107526881720430122194_12 weigth = 0.172043010752688192196_12 weigth = 0.279569892473118252806_12 weigth = 0.172043010752688192201_12 weigth = 0.129032258064516131312_12 weigth = 0.107526881720430112171_12 weigth = 0.0107526881720430122224_12 weigth = 0.086021505376344092198_12 weigth = 0.010752688172043012Total weight = 46.5Biggest outgroup probability is 2196_12 (0.27956989247311825)*** Network 122028_13 weigth = 0.0192307692307692321923_13 weigth = 0.96153846153846161956_13 weigth = 0.019230769230769232Total weight = 26.0Biggest outgroup probability is 1923_13 (0.9615384615384616)*** Network 132848_14 weigth = 0.0144927536231884062044_14 weigth = 0.115942028985507252042_14 weigth = 0.26086956521739132850_14 weigth = 0.26086956521739132040_14 weigth = 0.173913043478260862853_14 weigth = 0.17391304347826086Total weight = 34.5Biggest outgroup probability is 2042_14 (0.2608695652173913)*** Network 142004_15 weigth = 1.0Total weight = 16.0Biggest outgroup probability is 2004_15 (1.0)*** Network 152274_17 weigth = 1.0Total weight = 1.0Biggest outgroup probability is 2274_17 (1.0)*** Network 162313_18 weigth = 1.0Total weight = 2.0Biggest outgroup probability is 2313_18 (1.0)*** Network 172278_19 weigth = 1.0Total weight = 1.0Biggest outgroup probability is 2278_19 (1.0)*** Network 182302_20 weigth = 1.0Total weight = 7.0Biggest outgroup probability is 2302_20 (1.0)*** Network 192342_21 weigth = 1.0Total weight = 2.0Biggest outgroup probability is 2342_21 (1.0)*** Network 202277_22 weigth = 1.0Total weight = 1.0Biggest outgroup probability is 2277_22 (1.0)*** Network 212281_23 weigth = 1.0Total weight = 1.0Biggest outgroup probability is 2281_23 (1.0)*** Network 222866_24 weigth = 0.752865_24 weigth = 0.25Total weight = 2.0Biggest outgroup probability is 2866_24 (0.75)*** Network 232801_25 weigth = 1.0Total weight = 3.0Biggest outgroup probability is 2801_25 (1.0)*** Network 242800_27 weigth = 0.0588235294117647052226_4 weigth = 0.76470588235294112805_26 weigth = 0.17647058823529413Total weight = 8.5Biggest outgroup probability is 2226_4 (0.7647058823529411)Calculations are finished._________________________________________________________TCS v1.21Thu Nov 02 09:55:02 CET 2017Datafile = /Users/arnenygren/Desktop/namnlös mapp 3/its_xinsi_402.nexIt took 0.034216666666666666 minutes. 
