# Supplementary material for: A mega-cryptic species complex hidden among one of the most common annelids in the North East Atlantic
Source: PLoS One. 2018 Jun 20;13(6):e0198356. doi: 10.1371/journal.pone.0198356 (PMC6010226; doi:10.1371/journal.pone.0198356)
Supplement: S24 Appendix — Log-file from the GMYC-analysis on COI-unique. (RTF) [file pone.0198356.s024.rtf]

Result of GMYC species delimitation	method:	single	likelihood of null model:	839.0492	maximum likelihood of GMYC model:	914.4762	likelihood ratio:	150.854	result of LR test:	0***	number of ML clusters:	25	confidence interval:	21-27	number of ML entities:	31	confidence interval:	27-33	threshold time:	-2.32205---------------------------------------------------------------------------SPECIES LIST> spec.list(test1)sample    GMYC_spec      sample_name1           1          2875_202           1          2329_203           2          2349_20 (should be _28)4           2          2302_20 (should be _28)5           3          2834_216           3          2894_217           4          2864_248           4          2869_249           5          2809_2510          5          2801_2511          6          2312_1812          6          2313_1813          6          2314_1814          7          1201_1315          7          2776_1316          7           T03_1317          7          2038_1318          7          2475_1319          7          2028_1320          7          2458_1321          7          2337_1322          7          2184_1323          7          1923_1324          7          2215_1325          7          1986_1326          7          1998_1327          7          2183_1328          7          2921_1329          8          2200_1230          8          2818_1231          8          2197_1232          8          2222_1233          8          2196_1234          8          2199_1235          8          2827_1236          8          2195_1237          8          2198_1238          8          1312_1239          8          2202_1240          8          2194_1241          8          2193_1242          8          2171_1243          8          2826_1244          8          2201_1245          9          2304_1046          9          2031_1047          9          2034_1048          9          2033_1049         10          2899_1150         10          2347_1151         10          2323_1152         10          2786_1153         11           2476_854         11           2798_855         11           2037_856         11           2036_857         11           1561_858         11           2457_859         11           1922_860         11           2478_861         11           1202_862         11           1957_863         11           2896_864         11           2925_865         11           1946_866         11           2456_867         11           1988_868         11           2775_869         11           1958_870         11           1985_871         11           1984_872         11           1203_873         11           2920_874         11           1199_875         11           1200_876         11           2002_877         11           1994_878         11           2039_879         11           1996_880         11           2001_881         12           1198_882         12           2214_883         12           1197_884         12           1995_885         12           2014_886         12           2013_887         13            859_988         13            862_989         14           2449_790         14           2914_791         14            829_792         14           2448_793         14           2447_794         14           1309_795         14           2450_796         14           2859_797         15           1873_698         15            850_699         15           1870_6100        15           1319_6101        15           2167_6102        15            838_6103        15           1313_6104        15           1871_6105        15            839_6106        15           1872_6107        15           1875_6108        16          2030_15109        16          2004_15110        16          2005_15111        16          2006_15112        17           2932_3113        17           2912_3114        17           2888_3115        17           2906_3116        17           2907_3117        17           2879_3118        17           2884_3119        17           2882_3120        17           2915_3121        17           1207_3122        17           2814_3123        17           2935_3124        17           2908_3125        17           2465_3126        17           2380_3127        17           2464_3128        17           2463_3129        17           2872_3130        17           2891_3131        17           2812_3132        17           2934_3133        17           2878_3134        17           2886_3135        17           2883_3136        17           2930_3137        17           2887_3138        17           2923_3139        17           2876_3140        17           2910_3141        17           2933_3142        17           2916_3143        17           2929_3144        17           2880_3145        17           2885_3146        17           2889_3147        17           1310_3148        17           2913_3149        17           2881_3150        18           2901_3151        18           2480_3152        18           2877_3153        18           2922_3154        18           2874_3155        18           2931_3156        19           2370_2157        19           2369_2158        19           2271_2159        19           2332_2160        19  POLYNOR088_13_2161        19           1987_2162        19           2331_2163        19            844_2164        19           2180_2165        19           2326_2166        19           2185_2167        19           2381_2168        19           1311_2169        19           2182_2170        19           2328_2171        19           2354_2172        19           2216_2173        19           2333_2174        19           2187_2175        19           2181_2176        19           2272_2177        19           2378_2178        19           2352_2179        19           2367_2180        19           2338_2181        20          2850_14182        20          2843_14183        20          2479_14184        20 POLYNOR086_13_14185        20          2852_14186        21           2045_4187        21           2227_4188        21           2234_4189        21           2229_4190        21           2226_4191        21           TB28_4192        21           2228_4193        22           2904_5194        22           2927_5195        22            840_5196        22           2777_5197        22           2918_5198        22    PONOR087_13_5199        22            842_5200        22           2928_5201        22           2791_5202        22            T02_5203        23          2306_16204        23          2468_16205        23          2279_16206        23          2300_16207        23          2466_16208        23          2470_16209        23          2294_16210        23          2269_16211        23          2268_16212        23          2270_16213        23          2469_16214        23          2467_16215        23          2474_16216        23          2471_16217        23          2295_16218        23          2303_16219        23          2288_16220        23          2280_16221        23          2330_16222        23          2364_16223        23          2389_16224        24          2357_16225        24          2339_16226        24          2361_16227        24          2325_16228        24          2309_16229        24          2311_16230        24          2366_16231        24          2344_16232        24          2374_16233        24          2365_16234        24          2284_16235        24          2315_16236        24          2359_16237        24          2358_16238        24          2307_16239        24          2345_16240        24          2299_16241        24          2298_16242        24          2289_16243        24          2290_16244        24          2356_16245        24          2373_16246        24          2316_16247        24          2362_16248        24          2363_16249        24          2308_16250        24          2267_16251        24          2305_16252        24          2297_16253        25           2444_1254        25           1342_1255        25            T06_1256        25            T05_1257        25           2445_1258        25           1324_1259        25            833_1260        25           2871_1261        25           TB29_1262        25           1327_1263        25           2440_1264        25           2796_1265        25           2453_1266        26          2277_22267        27          2278_19268        28          2274_17269        29          2805_26270        30          2800_27271        31          2281_23 
