# Supplementary figures and images for: A mega-cryptic species complex hidden among one of the most common annelids in the North East Atlantic
Source: PLoS One. 2018 Jun 20;13(6):e0198356. doi: 10.1371/journal.pone.0198356 (PMC6010226; doi:10.1371/journal.pone.0198356)

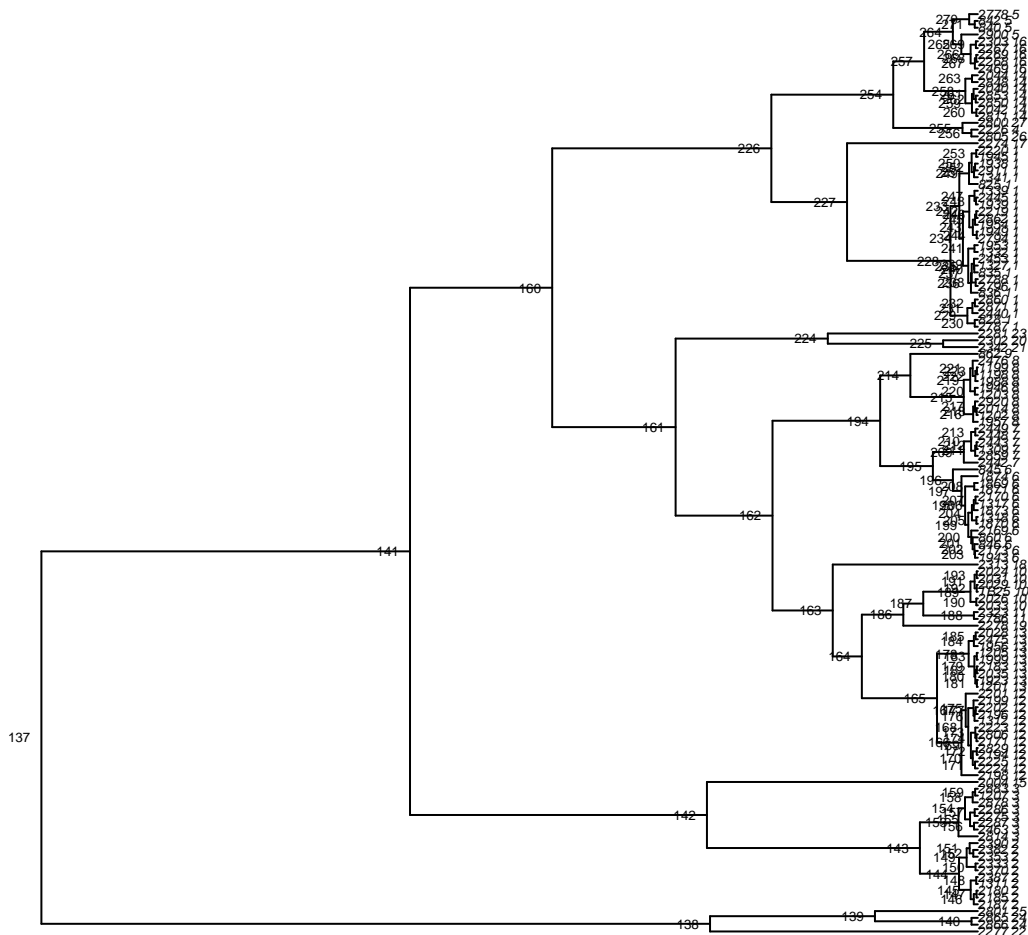

Supplement: S26 Appendix — Topology from the GMYC-analysis on ITS2s with node numbers. (PDF) [file pone.0198356.s026.pdf]

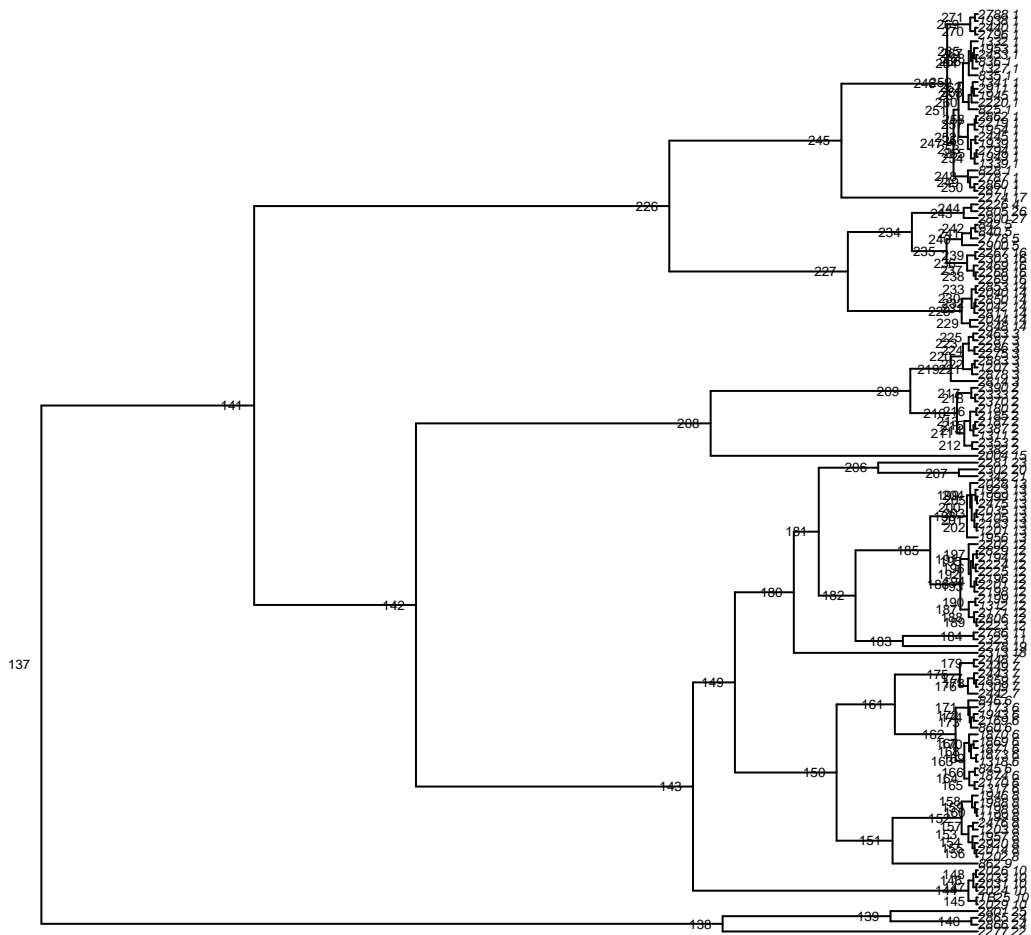

Supplement: S29 Appendix — Topology from the GMYC-analysis on ITS2s with node numbers. (PDF) [file pone.0198356.s029.pdf]
