# Supplementary material for: A mega-cryptic species complex hidden among one of the most common annelids in the North East Atlantic
Source: PLoS One. 2018 Jun 20;13(6):e0198356. doi: 10.1371/journal.pone.0198356 (PMC6010226; doi:10.1371/journal.pone.0198356)
Supplement: S27 Appendix — Log-file from the GMYC-analysis on ITS2s. (RTF) [file pone.0198356.s027.rtf]

((2277_22:30.186752384845715,((2866_24:0.7156040330841863,2865_24:0.7156040330841863):10.882272165426105,2801_25:11.597876198510292):18.588876186335426):75.37314768690075,((((((2187_2:0.45775000788770187,(2185_2:0.14661342901917915,2180_2:0.14661342901917912):0.3111365788685228):0.34943685063458013,(1311_2:0.18234801889387076,2387_2:0.18234801889387078):0.6248388396284112):1.3125785362937092,((2370_2:0.26014138202645254,2333_2:0.26014138202645254):1.0011701014943621,((2353_2:0.5131826925399744,2382_2:0.5131826925399744):0.4091596076985887,2390_2:0.9223423002385631):0.3389691832822517):0.8584539112951766):4.409961314347507,(2814_3:2.1827331349952286,(((2463_3:0.4343492911439278,2287_3:0.4343492911439278):0.44585725147271593,(2275_3:0.35760379886798394,2286_3:0.35760379886798394):0.5226027437486598):0.5665163330577242,(2878_3:0.5834142710546656,(1207_3:0.30469603193382044,2883_3:0.30469603193382044):0.27871823912084515):0.8633086046197023):0.7360102593208606):4.34699357416827):24.006887073613353,2004_15:30.53661378277685):33.47267573893486,(((((((2198_12:1.847213926554931,(((((2224_12:0.3177949612092484,2225_12:0.31779496120924833):0.2535476205236237,(2194_12:0.12240857166635156,2829_12:0.12240857166635158):0.44893401006652045):0.21118797054125793,((2171_12:0.1513853390505589,2806_12:0.15138533905055884):0.13705189708551985,2223_12:0.28843723613607875):0.49409331613805124):0.29921127517698065,((1312_12:0.3745501533256612,(2196_12:0.23967497784512948,2202_12:0.23967497784512945):0.13487517548053177):0.11066534416508034,2199_12:0.48521549749074155):0.596526329960369):0.2853015125693885,2201_12:1.367043340020499):0.4801705865344319):2.752996589784645,((((1201_13:0.1084978064571483,1923_13:0.10849780645714845):0.17756484900463948,(2035_13:0.11234762647012424,2183_13:0.11234762647012424):0.17371502899166363):0.10267627657985329,(1999_13:0.1188989958083064,1205_13:0.11889899580830646):0.2698399362333347):0.6478147826748375,(1956_13:0.4463454336016725,(2475_13:0.24404920893940285,2028_13:0.24404920893940285):0.20229622466226962):0.5902082811148062):3.5636568016230976):8.474410151316977,(2278_19:8.400178974334853,((2786_11:0.44357661263958964,2323_11:0.44357661263958964):5.708331271450648,((2033_10:0.1725813674588105,2026_10:0.1725813674588105):0.6632804427354092,((TB25_10:0.12671711997163684,2029_10:0.12671711997163687):0.23842461374058954,(2031_10:0.12581563655475606,2024_10:0.12581563655475606):0.2393260971574703):0.4707200764819933):5.316046073896018):2.2482710902446152):4.6744416933217):3.2793461941429047,2313_18:16.353966861799456):6.791921977612869,((((((((((1943_6:0.3162296085021055,2173_6:0.3162296085021055):0.0746530206021449,846_6:0.39088262910425037):0.195258712251557,860_6:0.5861413413558073):0.15722380764392385,2169_6:0.7433651489997313):0.33529357462076614,((1870_6:0.16317040478199507,1318_6:0.16317040478199504):0.5029166944864925,(1873_6:0.4574805851025195,(1317_6:0.2648818977232394,2170_6:0.2648818977232394):0.1925986873792801):0.20860651416596798):0.4125716243520099):0.3539645841435479,(1871_6:0.4068597381328388,1869_6:0.4068597381328388):1.0257635696312066):0.4135320561148135,1874_6:1.8461553638788588):0.964048385429612,845_6:2.810203749308471):2.2572626113681586,(2442_7:1.5788795269133118,((2859_7:0.301031270390222,(1309_7:0.14223919873748272,2443_7:0.14223919873748275):0.15879207165273923):0.45914837019106625,(2448_7:0.29104798211284,2449_7:0.29104798211284):0.4691316584684482):0.8186998863320235):3.4885868337633177):5.9439516449061784,(((1957_8:0.5265952812885976,((1202_8:0.1364146466305417,2014_8:0.13641464663054167):0.18412249463273986,2920_8:0.32053714126328153):0.20605814002531603):1.0617686967841031,((1203_8:0.33551731269171015,1946_8:0.33551731269171015):0.5264770848666048,((1988_8:0.27872078588071547,(1198_8:0.1063791586186898,1199_8:0.1063791586186898):0.1723416272620257):0.300154061163365,2476_8:0.5788748470440805):0.28311955051423443):0.7263695805143856):6.018400114413031,862_9:7.606764092485731):3.4046539130970768):12.13447083382952):10.923268615287782,((2342_21:3.9127049603775057,2302_20:3.9127049603775057):13.00211193495998,2281_23:16.914816895337484):17.154340559362623):13.912152941160485,(((((2787_1:0.34963271862444156,828_1:0.34963271862444156):0.8307778775656341,(2440_1:0.6123202135368752,(2871_1:0.33152139518729,2860_1:0.33152139518729):0.28079881834958514):0.5680903826532006):1.9050856370573364,((((836_1:0.7760733534020909,((2796_1:0.24564301166574928,2788_1:0.24564301166574928):0.44404319330798064,((835_1:0.4014988962295823,1327_1:0.40149889622958235):0.03645908983512125,2453_1:0.4379579860647036):0.2517282189090263):0.08638714842836104):0.30889447311783447,(1332_1:0.38222514729810514,1953_1:0.38222514729810514):0.7027426792218203):0.5978221875011502,(((2794_1:0.13076260418638289,1949_1:0.13076260418638289):0.43444248145563286,(1954_1:0.3142057084800602,(2862_1:0.17753173340205325,2219_1:0.17753173340205322):0.136673975078007):0.2509993771619556):0.402150209612824,((1939_1:0.21619450423064232,2445_1:0.21619450423064232):0.16558440231382707,1339_1:0.3817789065444694):0.5855763887103704):0.7154347187662359):0.40411869409854617,(825_1:0.9938655797121776,((1341_1:0.4289761318201143,(2911_1:0.3120921786267325,1938_1:0.3120921786267325):0.11688395319338174):0.2712606974650609,(1945_1:0.18132986298761275,2220_1:0.18132986298761275):0.5189069662975624):0.2936287504270024):1.0930431284074442):0.9985875251277903):11.657580626675902,2274_17:14.743076859923313):8.534548493687529,(((2805_26:0.7284733288022813,2226_4:0.7284733288022813):1.0015228709568156,2800_27:1.729996199759097):7.805459946572417,((((2811_14:0.1663740749375123,2042_14:0.16637407493751225):0.4981358889200215,((2850_14:0.23574659491039635,2853_14:0.23574659491039632):0.24517263244740858,2040_14:0.4809192273578049):0.1835907364997289):0.7442434983341149,(2848_14:0.6579977287131797,2044_14:0.6579977287131797):0.7507557334784689):4.64817321659432,((((2469_16:0.3362670592696724,(2268_16:0.17491868718808173,2269_16:0.1749186871880817):0.1613483720815906):0.4767571595926498,(2267_16:0.22236744651197182,2303_16:0.22236744651197182):0.5906567723503502):1.0490690240453309,2900_5:1.8620932429076529):0.9519363790313364,((840_5:0.34673965489656033,842_5:0.34673965489656033):0.6294718570762882,2778_5:0.9762115119728485):1.837818109966141):3.2428970568469793):3.4785294675455454):13.742169207279328):24.703685042249752):16.027979125851118):41.55061055003476);----------------------------------------------------> summary(test1)Result of GMYC species delimitation	method:	single	likelihood of null model:	298.7035	maximum likelihood of GMYC model:	312.2801	likelihood ratio:	27.1531	result of LR test:	1.269926e-06***	number of ML clusters:	16	confidence interval:	14-29	number of ML entities:	27	confidence interval:	23-48	threshold time:	-2.182733-----------------------------------------------------------> spec.list(test1)    GMYC_spec sample_name1           1     2866_242           1     2865_243           2      2187_24           2      2185_25           2      2180_26           2      1311_27           2      2387_28           2      2370_29           2      2333_210          2      2353_211          2      2382_212          2      2390_213          3      2814_314          3      2463_315          3      2287_316          3      2275_317          3      2286_318          3      2878_319          3      1207_320          3      2883_321          4     2198_1222          4     2224_1223          4     2225_1224          4     2194_1225          4     2829_1226          4     2171_1227          4     2806_1228          4     2223_1229          4     1312_1230          4     2196_1231          4     2202_1232          4     2199_1233          4     2201_1234          5     1201_1335          5     1923_1336          5     2035_1337          5     2183_1338          5     1999_1339          5     1205_1340          5     1956_1341          5     2475_1342          5     2028_1343          6     2786_1144          6     2323_1145          7     2033_1046          7     2026_1047          7     TB25_1048          7     2029_1049          7     2031_1050          7     2024_1051          8      1943_652          8      2173_653          8       846_654          8       860_655          8      2169_656          8      1870_657          8      1318_658          8      1873_659          8      1317_660          8      2170_661          8      1871_662          8      1869_663          8      1874_664          9      2442_765          9      2859_766          9      1309_767          9      2443_768          9      2448_769          9      2449_770         10      1957_871         10      1202_872         10      2014_873         10      2920_874         10      1203_875         10      1946_876         10      1988_877         10      1198_878         10      1199_879         10      2476_880         11      2787_181         11       828_182         11      2440_183         11      2871_184         11      2860_185         12       836_186         12      2796_187         12      2788_188         12       835_189         12      1327_190         12      2453_191         12      1332_192         12      1953_193         12      2794_194         12      1949_195         12      1954_196         12      2862_197         12      2219_198         12      1939_199         12      2445_1100        12      1339_1101        12       825_1102        12      1341_1103        12      2911_1104        12      1938_1105        12      1945_1106        12      2220_1107        13     2805_26108        13      2226_4109        13     2800_27110        14     2811_14111        14     2042_14112        14     2850_14113        14     2853_14114        14     2040_14115        14     2848_14116        14     2044_14117        15     2469_16118        15     2268_16119        15     2269_16120        15     2267_16121        15     2303_16122        15      2900_5123        16       840_5124        16       842_5125        16      2778_5126        17     2277_22127        18     2801_25128        19     2004_15129        20     2278_19130        21     2313_18131        22       845_6132        23       862_9133        24     2342_21134        25     2302_20135        26     2281_23136        27     2274_17
