# Supplementary material for: A mega-cryptic species complex hidden among one of the most common annelids in the North East Atlantic
Source: PLoS One. 2018 Jun 20;13(6):e0198356. doi: 10.1371/journal.pone.0198356 (PMC6010226; doi:10.1371/journal.pone.0198356)
Supplement: S30 Appendix — Log-file from the GMYC-analysis on ITS2s. (RTF) [file pone.0198356.s030.rtf]

((2277_22:28.76823026652041,((2866_24:0.7320190347313952,2865_24:0.7320190347313952):9.111541615360316,2801_25:9.84356065009171):18.924669616428694):76.79206826683044,(((((2029_10:0.15568387961751862,TB25_10:0.15568387961751856):0.9094373516366495,((2024_10:0.16454911633187017,2031_10:0.1645491163318701):0.34203079721526686,(2033_10:0.20697756366963696,2026_10:0.20697756366963693):0.2996023498775):0.5585413177070311):31.043409771004686,(((862_9:9.61196823360192,(((((1202_8:0.15889593406696953,2014_8:0.15889593406696953):0.2086969688190835,2920_8:0.36759290288605306):0.23196652414699276,1957_8:0.5995594270330458):0.4621528361731302,(1203_8:0.5486812279969072,2476_8:0.5486812279969071):0.5130310352092688):0.7636766106805364,(((1199_8:0.12762751709884587,1198_8:0.12762751709884587):0.2063517965944653,1988_8:0.33397931369331113):0.3169082652197641,1946_8:0.6508875789130754):1.174501294973637):7.7865793597152075):6.310982344556779,(((((1317_6:0.37563905047906004,2170_6:0.37563905047906004):0.5924590965770951,(1874_6:0.33372865668306995,845_6:0.33372865668306995):0.6343694903730852):0.557843517281234,(((1318_6:0.19617256101666422,1873_6:0.19617256101666425):0.575108861700745,(1871_6:0.47442292673393477,1869_6:0.47442292673393477):0.2968584959834746):0.21540595785885186,1870_6:0.9866873805762612):0.5392542837611279):0.9833957356544198,(((860_6:0.7692111360143997,(2169_6:0.4614864780960275,1943_6:0.4614864780960275):0.30772465791837217):0.1471688041005207,2173_6:0.9163799401149204):0.15975261019026377,846_6:1.0761325503051842):1.4332048496866248):6.843440905293162,((2442_7:1.1198409626088752,((1309_7:0.19151445452157306,2859_7:0.19151445452157306):0.24154608693853255,2443_7:0.43306054146010564):0.6867804211487696):0.9141301828093495,(2449_7:0.4571249291334788,2448_7:0.4571249291334788):1.5768462162847459):7.318807159866747):6.570172272873727):11.461537469842193,(2313_18:20.741781280528457,(((2278_19:8.45027216076261,(2323_11:0.48027650457205845,2786_11:0.48027650457205845):7.9699956561905525):5.336714726589985,(((((2223_12:0.15100210081405654,2806_12:0.15100210081405652):0.2754960774933394,2171_12:0.4264981783073959):0.5981901413275584,(1312_12:0.27758249549470265,2199_12:0.27758249549470265):0.7471058241402517):0.9484485130777591,(((2198_12:0.3841631616833595,(2201_12:0.16926507953278566,2196_12:0.16926507953278566):0.21489808215057393):0.4128189637425605,((2225_12:0.24482423834794195,2224_12:0.24482423834794206):0.30182368519082625,(2194_12:0.13131307855408791,2829_12:0.13131307855408791):0.4153348449846804):0.25033420188715183):0.2371430718388169,2202_12:1.034125197264737):0.9390116354479765):3.3949133738150774,(1956_13:1.2288708177163425,((((1201_13:0.14376689781699653,2183_13:0.14376689781699659):0.24134682594974705,(1205_13:0.15068105784955282,2035_13:0.15068105784955282):0.23443266591719084):0.3090558850905088,((2475_13:0.10249204401797307,1999_13:0.10249204401797304):0.1662296789285293,1923_13:0.2687217229465024):0.42544788591075006):0.16843287947984575,2028_13:0.8626024883370982):0.36626832937924436):4.139179388811448):8.418936680824805):4.146462673608998,((2342_21:2.1374384077676494,2302_20:2.1374384077676494):9.094483974305115,2281_23:11.231922382072764):6.7015271788888295):2.808331719566862):6.642706767472436):4.724042954257964):31.22657198733407,(2004_15:30.119171724316317,((((2382_2:0.6379579743413273,2353_2:0.6379579743413273):0.8871040157783183,((1311_2:0.49040303540972296,(2387_2:0.3067159438571316,2187_2:0.3067159438571316):0.18368709155259139):0.3729421191768042,(2185_2:0.16601813228914838,2180_2:0.16601813228914838):0.6973270222973788):0.6617168355331184):0.8303338544970169,((2370_2:0.29943968604163296,2333_2:0.29943968604163296):0.4351078332263432,2390_2:0.7345475192679762):1.6208483253486863):5.258403685034764,(2814_3:3.053259766721714,((2878_3:0.6448718916536831,(1207_3:0.35534241072502415,2883_3:0.35534241072502415):0.2895294809286589):1.031075388622483,((2275_3:0.38355436258849857,2286_3:0.38355436258849857):0.6446032136014423,(2287_3:0.5175648095076446,2463_3:0.5175648095076446):0.5105927666822963):0.6477897040862253):1.3773124864455477):4.560539762929713):22.50537219466489):33.21593126527661):18.242120336793796,((((2848_14:0.8925097003758128,2044_14:0.8925097003758128):0.9284292250580679,((2811_14:0.39057851191703946,(2042_14:0.27355174977052554,2850_14:0.2735517497705255):0.11702676214651392):0.31497494482941857,(2040_14:0.24327594412159761,2853_14:0.24327594412159756):0.4622775126248604):1.1153854686874227):12.831308729175705,(((((2269_16:0.24873987656596908,2268_16:0.24873987656596905):0.21966633549515094,2469_16:0.46840621206112):0.739895900053865,(2303_16:0.2878791538637706,2267_16:0.2878791538637706):0.9204229582512143):2.31624467095145,(2900_5:1.7899738243897358,(2778_5:0.7678200150274628,(840_5:0.2801981164350464,842_5:0.2801981164350464):0.48762189859241634):1.022153809362273):1.734572958676699):3.908379475295239,(2800_27:1.5962546604428922,(2805_26:0.7375553153710348,2226_4:0.7375553153710349):0.8586993450718574):5.836671597918782):7.219321396247912):20.130438507152938,(2274_17:15.376520684284321,(((((2871_1:0.3876568453318752,2860_1:0.3876568453318752):0.47734256913829304,2787_1:0.8649994144701683):0.25577304762858544,828_1:1.1207724620987536):1.62000077198081,((((1339_1:0.36322476472696724,(1949_1:0.14927962324300162,2794_1:0.14927962324300162):0.21394514148396554):0.42068835905502494,(1939_1:0.27226499682431476,2445_1:0.27226499682431476):0.5116481269576774):0.34278722631634984,(1954_1:0.3599160480129656,(2219_1:0.21247776703567853,2862_1:0.21247776703567853):0.147438280977287):0.7667843020853764):1.0524412127892768,((825_1:1.037195764927767,(2220_1:0.7121136522253699,((1945_1:0.43180210810516045,2911_1:0.43180210810516045):0.1435421671993583,1341_1:0.5753442753045187):0.13676937692085112):0.3250821127023972):0.6423023503713019,(835_1:1.0227190381404172,((1327_1:0.6015746099481876,((836_1:0.17719732846180944,2453_1:0.1771973284618095):0.24418393849646677,1953_1:0.42138126695827627):0.18019334298991135):0.18086917440140526,1332_1:0.7824437843495928):0.24027525379082437):0.6567790771586517):0.49964344758854984):0.5616316711919449):0.7171754561309709,((2796_1:0.2989814560735733,2440_1:0.2989814560735733):0.5586842693420193,(1938_1:0.3331491196798375,2788_1:0.33314911967983746):0.5245166057357551):2.6002829647949417):11.918571994073787):19.406165477478204):46.794537164624195):23.98307520696413);----------------------------------------------> summary(test1)Result of GMYC species delimitation	method:	single	likelihood of null model:	277.7269	maximum likelihood of GMYC model:	295.1348	likelihood ratio:	34.81566	result of LR test:	2.753443e-08***	number of ML clusters:	18	confidence interval:	15-28	number of ML entities:	27	confidence interval:	23-41	threshold time:	-2.509337-----------------------------------------------------------------> spec.list(test1)    GMYC_spec sample_name1           1     2866_242           1     2865_243           2     2029_104           2     TB25_105           2     2024_106           2     2031_107           2     2033_108           2     2026_109           3      1202_810          3      2014_811          3      2920_812          3      1957_813          3      1203_814          3      2476_815          3      1199_816          3      1198_817          3      1988_818          3      1946_819          4      1317_620          4      2170_621          4      1874_622          4       845_623          4      1318_624          4      1873_625          4      1871_626          4      1869_627          4      1870_628          4       860_629          4      2169_630          4      1943_631          4      2173_632          4       846_633          5      2442_734          5      1309_735          5      2859_736          5      2443_737          5      2449_738          5      2448_739          6     2323_1140          6     2786_1141          7     2223_1242          7     2806_1243          7     2171_1244          7     1312_1245          7     2199_1246          7     2198_1247          7     2201_1248          7     2196_1249          7     2225_1250          7     2224_1251          7     2194_1252          7     2829_1253          7     2202_1254          8     1956_1355          8     1201_1356          8     2183_1357          8     1205_1358          8     2035_1359          8     2475_1360          8     1999_1361          8     1923_1362          8     2028_1363          9     2342_2164          9     2302_2065         10      2382_266         10      2353_267         10      1311_268         10      2387_269         10      2187_270         10      2185_271         10      2180_272         10      2370_273         10      2333_274         10      2390_275         11      2878_376         11      1207_377         11      2883_378         11      2275_379         11      2286_380         11      2287_381         11      2463_382         12     2848_1483         12     2044_1484         12     2811_1485         12     2042_1486         12     2850_1487         12     2040_1488         12     2853_1489         13     2269_1690         13     2268_1691         13     2469_1692         13     2303_1693         13     2267_1694         14      2900_595         14      2778_596         14       840_597         14       842_598         15     2800_2799         15     2805_26100        15      2226_4101        16      2871_1102        16      2860_1103        16      2787_1104        16       828_1105        17      1339_1106        17      1949_1107        17      2794_1108        17      1939_1109        17      2445_1110        17      1954_1111        17      2219_1112        17      2862_1113        17       825_1114        17      2220_1115        17      1945_1116        17      2911_1117        17      1341_1118        17       835_1119        17      1327_1120        17       836_1121        17      2453_1122        17      1953_1123        17      1332_1124        18      2796_1125        18      2440_1126        18      1938_1127        18      2788_1128        19     2277_22129        20     2801_25130        21       862_9131        22     2313_18132        23     2278_19133        24     2281_23134        25     2004_15135        26      2814_3136        27     2274_17
