# Supplementary material for: A mega-cryptic species complex hidden among one of the most common annelids in the North East Atlantic
Source: PLoS One. 2018 Jun 20;13(6):e0198356. doi: 10.1371/journal.pone.0198356 (PMC6010226; doi:10.1371/journal.pone.0198356)
Supplement: S36 Appendix — List of sequenced specimens with voucher specification, site ID (see Table 1), sequence ID, and GenBank accession numbers. (DOCX) [file pone.0198356.s036.docx]

S36 Appendix. List of sequenced specimens with voucher specification, site ID (see Table1), seuqence ID, and GenBank accession numbers. Abbreviations as follows: ZMBN= Department of Natural History, University Museum of Bergen, GNM=The Gothenburg Museum of Natural History, NTNU-VM=Norwegian University of Science and Technology, NTNU University Museum, Trondheim, SMF=Senckenberg Museum Frankfurt, KA=Kattegat, SK=Skagerrak, NS=North Sea, ISCS=Irish Sea, Celtic Sea, NC=Norwegian coast and shelf, NWS=Norwegian Sea, BS=Barents Sea, AO=Arctic Ocean, GS=Greenland Sea, SI=South of Iceland.

| **Specimen voucher** | **Site ID** | **Sequence ID** | **COI** | **16S** | **ITS2** | **28S** |
| --- | --- | --- | --- | --- | --- | --- |
| ZMBN 116171 | SK25 | 824_1 |  |  | MG024492 |  |
| ZMBN 116172 | SK25 | 825_1 | MG024894 |  | MG024493 |  |
| ZMBN 116173 | SK25 | 826_1 | MG024895 |  |  |  |
| ZMBN 116174 | SK25 | 828_1 | MG024896 |  | MG024494 |  |
| ZMBN 116175 | SK25 | 830_1 | MG024897 |  | MG024495 |  |
| ZMBN 116176 | SK25 | 831_1 | MG024898 |  |  |  |
| ZMBN 116177 | SK25 | 833_1 | MG024899 |  |  |  |
| ZMBN 116178 | SK25 | 834_1 | MG024900 |  |  |  |
| ZMBN 116179 | SK25 | 835_1 | MG024901 |  | MG024496 |  |
| ZMBN 116180 | SK25 | 836_1 | MG024902 |  | MG024497 |  |
| ZMBN 116181 | SK25 | 837_1 | MG024903 |  |  |  |
| GNM 15106 | SK4 | 843_1 | MG024904 |  | MG024498 |  |
| ZMBN 116182 | NS2 | 856_1 | MG024905 |  | MG024499 |  |
| ZMBN 116183 | NS2 | 857_1 | MG024906 |  |  |  |
| ZMBN 116184 | NS2 | 858_1 | MG024907 |  | MG024500 | MG025356 |
| ZMBN 116185 | SK24 | 1322_1 | MG024908 |  | MG024501 |  |
| ZMBN 116186 | SK24 | 1323_1 | MG024909 |  |  |  |
| ZMBN 116187 | SK24 | 1324_1 | MG024910 |  | MG024502 |  |
| ZMBN 116188 | SK24 | 1325_1 | MG024911 |  |  |  |
| ZMBN 116189 | SK24 | 1326_1 | MG024912 |  | MG024503 |  |
| ZMBN 116190 | SK24 | 1327_1 | MG024913 |  | MG024504 |  |
| ZMBN 116191 | SK24 | 1328_1 | MG024914 |  | MG024505 |  |
| ZMBN 116192 | SK24 | 1329_1 | MG024915 |  |  |  |
| ZMBN 116193 | SK24 | 1330_1 | MG024916 |  |  |  |
| ZMBN 116194 | SK24 | 1331_1 |  |  | MG024506 |  |
| ZMBN 116195 | SK24 | 1332_1 |  |  | MG024507 |  |
| ZMBN 116196 | SK24 | 1333_1 | MG024917 |  | MG024508 |  |
| ZMBN 116197 | SK24 | 1334_1 |  |  | MG024509 |  |
| ZMBN 116198 | SK24 | 1335_1 | MG024918 |  | MG024510 |  |
| ZMBN 116199 | SK24 | 1336_1 | MG024919 |  |  |  |
| ZMBN 116200 | SK24 | 1337_1 | MG024920 |  | MG024511 |  |
| ZMBN 116201 | SK24 | 1338_1 | MG024921 |  |  |  |
| ZMBN 116202 | SK24 | 1339_1 | MG024922 |  | MG024512 |  |
| ZMBN 116203 | SK24 | 1340_1 | MG024923 |  |  |  |
| ZMBN 116204 | SK24 | 1341_1 |  |  | MG024513 |  |
| ZMBN 116205 | SK24 | 1342_1 | MG024924 |  | MG024514 |  |
| ZMBN 116206 | SK24 | 1344_1 | MG024925 |  | MG024515 |  |
| ZMBN 116207 | SK15 | 1938_1 | MG024926 |  | MG024516 |  |
| ZMBN 116208 | SK15 | 1939_1 | MG024927 |  | MG024517 |  |
| ZMBN 116209 | SK15 | 1940_1 | MG024928 |  | MG024518 |  |
| ZMBN 116210 | SK15 | 1941_1 |  |  | MG024519 |  |
| ZMBN 116211 | SK13 | 1945_1 | MG024929 |  | MG024520 |  |
| ZMBN 116212 | SK13 | 1947_1 | MG024930 |  | MG024521 |  |
| ZMBN 116213 | SK13 | 1948_1 | MG024931 | MG025443 | MG024522 | MG025357 |
| ZMBN 116214 | SK26 | 1949_1 | MG024932 |  | MG024523 |  |
| ZMBN 116215 | SK26 | 1950_1 | MG024933 |  | MG024524 |  |
| ZMBN 116216 | SK26 | 1951_1 | MG024934 |  | MG024525 |  |
| ZMBN 116217 | SK26 | 1952_1 | MG024935 |  | MG024526 |  |
| ZMBN 116218 | SK26 | 1953_1 |  |  | MG024527 |  |
| ZMBN 116219 | SK26 | 1954_1 |  |  | MG024528 |  |
| ZMBN 116220 | SK26 | 1955_1 |  |  | MG024529 |  |
| GNM 14640 | SK3 | 2217_1 |  |  | MG024530 |  |
| GNM 14642 | KA4 | 2219_1 |  |  | MG024531 |  |
| GNM 14643 | KA5 | 2220_1 |  |  | MG024532 |  |
| GNM 14643_1 | KA5 | 2221_1 |  |  | MG024533 |  |
| GNM 14649 | SK21 | 2241_1 | MG024936 |  | MG024534 |  |
| ZMBN 116221 | NCS4 | 2439_1 | MG024937 |  |  |  |
| ZMBN 116222 | NCS4 | 2440_1 | MG024938 |  | MG024535 |  |
| ZMBN 116223 | NCS4 | 2441_1 | MG024939 |  | MG024536 | MG025358 |
| ZMBN 116224 | NCS2 | 2444_1 | MG024940 |  |  |  |
| ZMBN 116225 | NCS2 | 2445_1 | MG024941 | MG025444 | MG024537 | MG025359 |
| ZMBN 116226 | NCS1 | 2446_1 | MG024942 |  |  |  |
| ZMBN 116227 | NCS2 | 2451_1 |  |  | MG024538 |  |
| ZMBN 116228 | NCS2 | 2453_1 | MG024943 | MG025445 | MG024539 | MG025360 |
| ZMBN 116229 | NCS10 | 2787_1 |  |  | MG024540 |  |
| ZMBN 116230 | NCS10 | 2788_1 |  |  | MG024541 |  |
| ZMBN 116231 | NCS10 | 2789_1 |  |  | MG024542 |  |
| ZMBN 116232 | NS7 | 2794_1 | MG024944 |  | MG024543 |  |
| ZMBN 116233 | NS7 | 2796_1 | MG024945 |  | MG024544 |  |
| ZMBN 116234 | NS7 | 2797_1 |  |  | MG024545 |  |
| ZMBN 116235 | NCS9 | 2839_1 | MG024946 |  |  |  |
| ZMBN 116236 | NCS9 | 2840_1 |  |  | MG024546 |  |
| ZMBN 116237 | NCS9 | 2841_1 | MG024947 |  | MG024547 |  |
| ZMBN 116238 | NCS7 | 2860_1 | MG024948 |  | MG024548 |  |
| ZMBN 116239 | NCS7 | 2861_1 | MG024949 |  |  |  |
| ZMBN 116240 | NCS7 | 2862_1 | MG024950 |  | MG024549 |  |
| ZMBN 116241 | NCS8 | 2871_1 | MG024951 |  | MG024550 |  |
| ZMBN 116242 | NCS19 | 2909_1 | MG024952 |  | MG024551 |  |
| ZMBN 116243 | NCS19 | 2911_1 | MG024953 |  | MG024552 |  |
| NTNU-VM-61384 | NCS23 | T05_1 | MG024954 |  |  |  |
| NTNU-VM-61385 | NCS23 | T06_1 | MG024955 |  | MG024553 |  |
| NTNU-VM-59990 | NS1 | TB29_1 | MG024956 |  | MG024554 |  |
| GNM 15107 | SK4 | 841_2 | MG024957 |  |  |  |
| GNM 15108 | SK18 | 844_2 | MG024958 |  | MG024555 |  |
| GNM 15109 | SK5 | 1311_2 | MG024959 | MG025446 | MG024556 | MG025361 |
| ZMBN 116272 | BS6 | 1987_2 | MG024960 |  |  |  |
| ZMBN 116244 | BS1 | 2180_2 | MG024961 |  | MG024557 |  |
| ZMBN 116245 | BS1 | 2181_2 | MG024962 |  | MG024558 |  |
| ZMBN 116246 | BS1 | 2182_2 | MG024963 |  |  |  |
| ZMBN 116247 | BS9 | 2185_2 | MG024964 |  | MG024559 |  |
| ZMBN 116248 | BS9 | 2187_2 | MG024965 |  | MG024560 |  |
| GNM 14639 | SK17 | 2216_2 | MG024966 |  | MG024561 |  |
| SMF 24679 | GS2 | 2271_2 | MG024967 | MG025447 | MG024562 | MG025362 |
| SMF 24673 | GS2 | 2272_2 | MG024968 |  | MG024563 |  |
| ZMBN 116249 | BS7 | 2322_2 | MG024969 |  | MG024564 |  |
| ZMBN 116250 | BS13 | 2326_2 | MG024970 |  |  |  |
| ZMBN 116251 | NCS32 | 2328_2 | MG024971 |  | MG024565 |  |
| ZMBN 116252 | BS14 | 2331_2 | MG024972 |  | MG024566 |  |
| ZMBN 116253 | BS14 | 2332_2 | MG024973 |  | MG024567 |  |
| ZMBN 116254 | BS14 | 2333_2 | MG024974 |  | MG024568 |  |
| ZMBN 116255 | BS4 | 2336_2 |  |  | MG024569 |  |
| ZMBN 116256 | BS11 | 2338_2 | MG024975 |  | MG024570 |  |
| ZMBN 116257 | BS14 | 2351_2 | MG024976 |  |  |  |
| ZMBN 116258 | BS14 | 2352_2 | MG024977 | MG025448 | MG024571 | MG025363 |
| ZMBN 116259 | BS14 | 2353_2 | MG024978 |  | MG024572 | MG025364 |
| ZMBN 116260 | BS14 | 2354_2 | MG024979 |  | MG024573 |  |
| ZMBN 116261 | BS14 | 2360_2 | MG024980 |  |  |  |
| ZMBN 116262 | BS14 | 2367_2 | MG024981 |  | MG024574 |  |
| ZMBN 116263 | BS14 | 2368_2 | MG024982 |  | MG024575 |  |
| ZMBN 116264 | BS14 | 2369_2 | MG024983 |  | MG024576 |  |
| ZMBN 116265 | BS14 | 2370_2 | MG024984 |  | MG024577 |  |
| ZMBN 116266 | BS12 | 2376_2 |  |  | MG024578 |  |
| ZMBN 116267 | BS12 | 2378_2 | MG024985 |  |  |  |
| ZMBN 116268 | NWS2 | 2381_2 | MG024986 |  | MG024579 |  |
| ZMBN 116269 | NWS2 | 2382_2 | MG024987 |  | MG024580 |  |
| ZMBN 116270 | BS10 | 2387_2 |  |  | MG024581 |  |
| ZMBN 116271 | BS10 | 2390_2 |  |  | MG024582 |  |
| NTNU-VM-68253 | NCS24 | POLYNOR088_2 | MG024988 |  |  |  |
| GNM 15110 | SK11 | 1207_3 | MG024989 |  | MG024583 | MG025365 |
| GNM 15111 | SK5 | 1310_3 | MG024990 | MG025449 | MG024584 | MG025366 |
| SMF 24669 | SI9 | 2275_3 | MG024991 |  | MG024585 |  |
| SMF 24683 | SI11 | 2285_3 | MG024992 |  | MG024586 |  |
| SMF 24666 | SI11 | 2286_3 |  |  | MG024587 |  |
| SMF 24667 | SI11 | 2287_3 | MG024993 |  | MG024588 |  |
| SMF 24684 | SI11 | 2291_3 |  |  | MG024589 |  |
| SMF 24670 | SI8 | 2301_3 |  |  | MG024590 |  |
| ZMBN 116273 | NWS2 | 2380_3 | MG024994 |  | MG024591 |  |
| SMF 24692 | SI10 | 2463_3 | MG024995 | MG025450 | MG024592 | MG025367 |
| SMF 24665 | SI10 | 2464_3 | MG024996 |  | MG024593 |  |
| SMF 24668 | SI10 | 2465_3 | MG024997 |  | MG024594 |  |
| NTNU-VM-68196 | NCS24 | 2480_3 | MG024998 | MG025452 | MG024595 | MG025368 |
| ZMBN 116274 | NCS17 | 2781_3 |  |  | MG024596 |  |
| ZMBN 116275 | NCS17 | 2784_3 |  |  | MG024597 |  |
| ZMBN 116276 | NCS20 | 2812_3 | MG024999 |  | MG024598 |  |
| ZMBN 116277 | NCS15 | 2814_3 | MG025000 |  | MG024599 |  |
| ZMBN 116278 | NCS18 | 2872_3 | MG025001 |  | MG024600 |  |
| ZMBN 116279 | NCS18 | 2873_3 | MG025002 |  | MG024601 |  |
| ZMBN 116280 | NCS18 | 2874_3 | MG025003 |  | MG024602 |  |
| ZMBN 116281 | NCS18 | 2876_3 | MG025004 |  | MG024603 |  |
| ZMBN 116282 | NCS18 | 2877_3 | MG025005 |  | MG024604 |  |
| ZMBN 116283 | NCS18 | 2878_3 | MG025006 |  | MG024605 |  |
| ZMBN 116284 | NCS18 | 2879_3 | MG025007 |  | MG024606 |  |
| ZMBN 116285 | NCS18 | 2880_3 | MG025008 |  | MG024607 |  |
| ZMBN 116286 | NCS18 | 2881_3 | MG025009 |  | MG024608 |  |
| ZMBN 116287 | NCS18 | 2882_3 | MG025010 |  | MG024609 |  |
| ZMBN 116288 | NCS18 | 2883_3 | MG025011 |  | MG024610 |  |
| ZMBN 116289 | NCS18 | 2884_3 | MG025012 |  |  |  |
| ZMBN 116290 | NCS18 | 2885_3 | MG025013 |  | MG024611 |  |
| ZMBN 116291 | NCS18 | 2886_3 | MG025014 |  | MG024612 |  |
| ZMBN 116292 | NCS18 | 2887_3 | MG025015 | MG025451 | MG024613 | MG025369 |
| ZMBN 116293 | NCS18 | 2888_3 | MG025016 |  | MG024614 |  |
| ZMBN 116294 | NCS18 | 2889_3 | MG025017 |  | MG024615 |  |
| ZMBN 116295 | NCS18 | 2890_3 | MG025018 |  | MG024616 |  |
| ZMBN 116296 | NCS18 | 2891_3 | MG025019 |  | MG024617 |  |
| ZMBN 116297 | NCS16 | 2901_3 | MG025020 |  | MG024618 |  |
| ZMBN 116298 | NCS16 | 2902_3 |  |  | MG024619 |  |
| ZMBN 116299 | NCS19 | 2906_3 | MG025021 |  | MG024620 |  |
| ZMBN 116300 | NCS19 | 2907_3 | MG025022 |  | MG024621 |  |
| ZMBN 116301 | NCS19 | 2908_3 | MG025023 |  | MG024622 |  |
| ZMBN 116302 | NCS19 | 2910_3 | MG025024 |  | MG024623 |  |
| ZMBN 116303 | NCS16 | 2912_3 | MG025025 |  | MG024624 |  |
| ZMBN 116304 | NCS16 | 2913_3 | MG025026 |  | MG024625 |  |
| ZMBN 116305 | NCS19 | 2915_3 | MG025027 |  | MG024626 |  |
| ZMBN 116306 | NCS19 | 2916_3 | MG025028 |  | MG024627 |  |
| ZMBN 116307 | NCS19 | 2917_3 | MG025029 |  | MG024628 |  |
| ZMBN 116308 | NCS5 | 2922_3 | MG025030 |  | MG024629 |  |
| ZMBN 116309 | NCS5 | 2923_3 | MG025031 |  | MG024630 |  |
| ZMBN 116310 | NCS5 | 2924_3 |  |  | MG024631 |  |
| ZMBN 116311 | NCS17 | 2929_3 | MG025032 |  |  |  |
| ZMBN 116312 | NCS17 | 2930_3 | MG025033 |  | MG024632 |  |
| ZMBN 116313 | NCS17 | 2931_3 | MG025034 |  | MG024633 |  |
| ZMBN 116314 | NCS17 | 2932_3 | MG025035 |  | MG024634 |  |
| ZMBN 116315 | NCS17 | 2933_3 | MG025036 |  | MG024635 |  |
| ZMBN 116316 | NCS17 | 2934_3 | MG025037 |  | MG024636 |  |
| ZMBN 116317 | NCS17 | 2935_3 | MG025038 |  | MG024637 |  |
| ZMBN 116318 | SK10 | 2045_4 | MG025039 | MG025456 | MG024638 | MG025370 |
| GNM 14645 | KA1 | 2226_4 | MG025040 |  | MG024639 |  |
| GNM 14645_1 | KA1 | 2227_4 | MG025041 |  | MG024640 |  |
| GNM 14645_2 | KA1 | 2228_4 | MG025042 |  | MG024641 |  |
| GNM 14645_3 | KA1 | 2229_4 | MG025043 |  | MG024642 |  |
| GNM 14645_4 | KA1 | 2230_4 | MG025044 |  | MG024643 |  |
| GNM 14645_5 | KA1 | 2231_4 | MG025045 |  | MG024644 |  |
| GNM 14645_6 | KA1 | 2232_4 | MG025046 |  | MG024645 |  |
| GNM 14645_7 | KA1 | 2233_4 | MG025047 | MG025453 | MG024646 | MG025371 |
| GNM 14645_8 | KA1 | 2234_4 | MG025048 | MG025454 | MG024647 | MG025372 |
| GNM 14645_9 | KA1 | 2235_4 | MG025049 | MG025455 | MG024648 | MG025373 |
| GNM 14645_10 | KA1 | 2236_4 | MG025050 |  | MG024649 |  |
| NTNU-VM-65049 | KA2 | TB27_4 | MG025051 |  |  |  |
| NTNU-VM-65048 | KA2 | TB28_4 | MG025052 |  | MG024650 |  |
| GNM 15112 | SK4 | 840_5 | MG025053 | MG025457 | MG024651 | MG025374 |
| GNM 15113 | SK4 | 842_5 | MG025054 | MG025458 | MG024652 | MG025375 |
| ZMBN 116319 | NCS6 | 2777_5 | MG025055 |  | MG024653 |  |
| ZMBN 116320 | NCS6 | 2778_5 | MG025056 |  | MG024654 |  |
| ZMBN 116321 | NCS6 | 2779_5 | MG025057 |  | MG024655 |  |
| ZMBN 116322 | NCS6 | 2780_5 | MG025058 |  | MG024656 |  |
| ZMBN 116323 | NCS6 | 2790_5 | MG025059 |  | MG024657 |  |
| ZMBN 116324 | NCS6 | 2791_5 | MG025060 |  | MG024658 |  |
| ZMBN 116325 | NCS6 | 2792_5 | MG025061 |  | MG024659 |  |
| ZMBN 116326 | NCS6 | 2793_5 | MG025062 | MG025459 | MG024660 | MG025376 |
| ZMBN 116327 | NCS14 | 2900_5 | MG025063 |  | MG024661 |  |
| ZMBN 116328 | NCS11 | 2904_5 | MG025064 | MG025460 | MG024662 | MG025377 |
| ZMBN 116329 | NCS19 | 2918_5 | MG025065 |  | MG024663 |  |
| ZMBN 116330 | NCS5 | 2926_5 | MG025066 |  | MG024664 |  |
| ZMBN 116331 | NCS5 | 2927_5 | MG025067 |  | MG024665 |  |
| ZMBN 116332 | NCS5 | 2928_5 | MG025068 |  | MG024666 |  |
| NTNU-VM-68252 | NCS25 | POLYNOR087_5 | MG025069 |  |  |  |
| NTNU-VM-61386 | NCS24 | T02_5 | MG025070 |  | MG024667 |  |
| NTNU-VM-61387 | NCS25 | T04_5 | MG025071 |  | MG024668 |  |
| GNM 15114 | SK2 | 838_6 | MG025072 |  | MG024669 |  |
| GNM 15115 | SK1 | 839_6 | MG025073 |  |  |  |
| GNM 15116 | SK22 | 845_6 | MG025074 |  | MG024670 |  |
| GNM 15117 | SK22 | 846_6 | MG025075 |  | MG024671 |  |
| GNM 15118 | SK20 | 847_6 | MG025076 |  | MG024672 |  |
| GNM 15119 | SK20 | 848_6 | MG025077 |  |  |  |
| GNM 15120 | SK20 | 849_6 | MG025078 |  | MG024673 | MG025378 |
| GNM 15121 | SK20 | 850_6 | MG025079 |  |  |  |
| ZMBN 116333 | NS4 | 860_6 | MG025080 |  | MG024674 |  |
| GNM 15122 | SK23 | 1313_6 | MG025081 |  | MG024675 |  |
| GNM 15123 | SK23 | 1314_6 | MG025082 |  |  |  |
| GNM 15124 | SK23 | 1315_6 | MG025083 |  |  |  |
| GNM 15125 | SK23 | 1316_6 | MG025084 |  | MG024676 |  |
| GNM 15126 | SK23 | 1317_6 | MG025085 |  | MG024677 |  |
| GNM 15127 | SK23 | 1318_6 | MG025086 |  | MG024678 |  |
| GNM 15128 | SK23 | 1319_6 | MG025087 |  | MG024679 |  |
| ZMBN 116334 | SK24 | 1320_6 |  |  | MG024680 |  |
| ZMBN 116335 | SK24 | 1321_6 | MG025088 |  | MG024681 |  |
| ZMBN 116336 | ISCS4 | 1869_6 | MG025089 |  | MG024682 |  |
| ZMBN 116337 | ISCS1 | 1870_6 | MG025090 | MG025461 | MG024683 | MG025379 |
| ZMBN 116338 | ISCS2 | 1871_6 | MG025091 |  | MG024684 |  |
| ZMBN 116339 | ISCS2 | 1872_6 | MG025092 |  | MG024685 |  |
| ZMBN 116340 | ISCS5 | 1873_6 | MG025093 |  | MG024686 |  |
| ZMBN 116341 | ISCS3 | 1874_6 | MG025094 |  | MG024687 |  |
| ZMBN 116342 | ISCS3 | 1875_6 | MG025095 |  | MG024688 |  |
| ZMBN 116343 | SK16 | 1942_6 | MG025096 |  | MG024689 |  |
| ZMBN 116344 | SK16 | 1943_6 | MG025097 | MG025462 | MG024690 | MG025380 |
| ZMBN 116345 | SK16 | 1944_6 | MG025098 |  |  |  |
| ZMBN 116346 | SK24 | 2046_6 | MG025099 |  |  |  |
| GNM 14625_d | KA6 | 2167_6 | MG025100 |  |  |  |
| GNM 14625_2 | KA6 | 2169_6 |  |  | MG024691 |  |
| GNM 14625_3 | KA6 | 2170_6 |  |  | MG024692 |  |
| GNM 14628_1 | SK6 | 2172_6 | MG025101 |  |  |  |
| GNM 14628 | SK6 | 2173_6 | MG025102 |  | MG024693 | MG025381 |
| GNM 14625 | KA6 | 2190_6 | MG025103 |  |  |  |
| ZMBN 116347 | NCS13 | 2774_6 | MG025104 |  |  |  |
| ZMBN 116348 | SK25 | 829_7 | MG025105 |  |  |  |
| ZMBN 116349 | SK25 | 1309_7 | MG025106 |  | MG024694 | MG025382 |
| ZMBN 116350 | NCS2 | 2442_7 | MG025107 | MG025463 | MG024695 | MG025383 |
| ZMBN 116351 | NCS2 | 2443_7 | MG025108 |  | MG024696 |  |
| ZMBN 116352 | NCS2 | 2447_7 | MG025109 |  |  |  |
| ZMBN 116353 | NCS2 | 2448_7 | MG025110 | MG025464 | MG024697 | MG025384 |
| ZMBN 116354 | NCS2 | 2449_7 | MG025111 | MG025465 | MG024698 | MG025385 |
| ZMBN 116355 | NCS2 | 2450_7 | MG025112 |  |  |  |
| ZMBN 116356 | NCS2 | 2452_7 | MG025113 |  |  |  |
| ZMBN 116357 | NCS7 | 2859_7 | MG025114 | MG025466 | MG024699 | MG025386 |
| ZMBN 116358 | NCS7 | 2863_7 | MG025115 |  |  |  |
| ZMBN 116359 | NCS21 | 2914_7 | MG025116 |  |  |  |
| GNM 15129 | SK7 | 1197_8 | MG025117 |  | MG024700 |  |
| GNM 15130 | SK7 | 1198_8 | MG025118 |  | MG024701 |  |
| GNM 15131 | SK7 | 1199_8 | MG025119 |  | MG024702 |  |
| GNM 15132 | SK7 | 1200_8 | MG025120 |  | MG024703 |  |
| GNM 15133 | SK7 | 1202_8 | MG025121 |  | MG024704 |  |
| GNM 15134 | SK7 | 1203_8 | MG025122 |  | MG024705 |  |
| ZMBN 116360 | NCS26 | 1561_8 | MG025123 |  | MG024706 |  |
| NTNU-VM-66569 | NCS29 | 1922_8 | MG025124 |  |  |  |
| ZMBN 116361 | SK13 | 1946_8 | MG025125 |  | MG024707 |  |
| NTNU-VM-66574 | NCS29 | 1957_8 | MG025126 |  | MG024708 |  |
| NTNU-VM-66573 | NCS29 | 1958_8 | MG025127 |  | MG024709 |  |
| ZMBN 116362 | NCS38 | 1984_8 | MG025128 |  |  |  |
| ZMBN 116363 | NCS37 | 1985_8 | MG025129 |  |  |  |
| ZMBN 116364 | BS2 | 1988_8 | MG025130 |  | MG024710 |  |
| ZMBN 116365 | BS2 | 1989_8 | MG025131 |  | MG024711 |  |
| ZMBN 116366 | BS3 | 1991_8 | MG025132 |  |  |  |
| ZMBN 116367 | BS3 | 1992_8 | MG025133 |  |  |  |
| ZMBN 116368 | NCS36 | 1994_8 | MG025134 |  |  |  |
| ZMBN 116369 | NCS35 | 1995_8 | MG025135 |  |  |  |
| ZMBN 116370 | NCS35 | 1996_8 | MG025136 |  |  |  |
| ZMBN 116371 | NCS28 | 2000_8 | MG025137 |  |  |  |
| ZMBN 116372 | NCS28 | 2001_8 | MG025138 |  |  |  |
| ZMBN 116373 | NCS27 | 2002_8 | MG025139 |  |  |  |
| ZMBN 116374 | NCS45 | 2013_8 | MG025140 |  | MG024712 |  |
| ZMBN 116375 | NCS45 | 2014_8 | MG025141 |  | MG024713 |  |
| ZMBN 116376 | NCS45 | 2015_8 | MG025142 |  | MG024714 |  |
| NTNU-VM-61388 | NCS24 | 2036_8 | MG025143 |  | MG024715 |  |
| NTNU-VM-61389 | NCS24 | 2037_8 | MG025144 |  | MG024716 |  |
| NTNU-VM-61390 | NCS25 | 2039_8 | MG025145 |  | MG024717 |  |
| GNM 14632_3 | SK12 | 2178_8 |  |  | MG024718 |  |
| GNM 14637 | SK19 | 2214_8 | MG025146 |  | MG024719 |  |
| ZMBN 116377 | NCS3 | 2456_8 | MG025147 |  |  |  |
| ZMBN 116378 | NCS3 | 2457_8 | MG025148 | MG025467 | MG024720 | MG025387 |
| NTNU-VM-68197 | NCS24 | 2476_8 | MG025149 | MG025468 | MG024721 | MG025388 |
| NTNU-VM-68198 | NCS24 | 2478_8 | MG025150 | MG025469 | MG024722 | MG025389 |
| ZMBN 116379 | NCS20 | 2775_8 | MG025151 |  | MG024723 |  |
| ZMBN 116380 | NCS19 | 2798_8 | MG025152 |  | MG024724 |  |
| ZMBN 116381 | NCS19 | 2799_8 | MG025153 |  | MG024725 |  |
| ZMBN 116382 | NCS17 | 2896_8 | MG025154 |  | MG024726 |  |
| ZMBN 116383 | NCS20 | 2920_8 | MG025155 |  | MG024727 |  |
| ZMBN 116384 | NCS5 | 2925_8 | MG025156 |  | MG024728 |  |
| ZMBN 116385 | NS3 | 859_9 | MG025157 |  |  |  |
| ZMBN 116386 | NS5 | 861_9 |  | MG025471 | MG024729 | MG025390 |
| ZMBN 116387 | NS6 | 862_9 | MG025158 | MG025470 | MG024730 | MG025391 |
| ZMBN 116389 | BS2 | 1990_10 | MG025159 |  |  |  |
| ZMBN 116390 | NCS41 | 2024_10 | MG025160 |  | MG024731 |  |
| ZMBN 116391 | NCS42 | 2026_10 | MG025161 |  | MG024732 |  |
| ZMBN 116392 | NCS46 | 2029_10 | MG025162 |  | MG024733 |  |
| ZMBN 116393 | NCS47 | 2031_10 | MG025163 | MG025472 | MG024734 | MG025392 |
| ZMBN 116394 | NCS47 | 2032_10 | MG025164 |  |  |  |
| ZMBN 116395 | NCS47 | 2033_10 | MG025165 | MG025473 | MG024735 | MG025393 |
| ZMBN 116396 | NCS47 | 2034_10 | MG025166 | MG025474 | MG024736 | MG025394 |
| SMF 24688 | GS7 | 2304_10 | MG025167 |  |  |  |
| ZMBN 116388 | BS7 | 2321_10 | MG025168 |  |  |  |
| NTNU-VM-61376 | NCS30 | TB25_10 | MG025169 |  | MG024737 |  |
| NTNU-VM-61377 | NCS30 | TB26_10 | MG025170 |  |  |  |
| ZMBN 116397 | NCS26 | 1560_11 | MG025171 | MG025475 | MG024738 | MG025395 |
| ZMBN 116398 | NCS31 | 2323_11 | MG025172 | MG025476 | MG024739 | MG025396 |
| ZMBN 116399 | NCS33 | 2347_11 | MG025173 |  |  |  |
| ZMBN 116401 | NCS12 | 2786_11 | MG025174 | MG025477 | MG024740 | MG025397 |
| ZMBN 116400 | NCS14 | 2899_11 | MG025175 |  |  |  |
| GNM 15135 | SK23 | 1312_12 | MG025176 | MG025478 | MG024741 | MG025398 |
| GNM 14644_1 | KA3 | 2171_12 | MG025177 |  | MG024742 |  |
| GNM 14630 | SK9 | 2193_12 | MG025178 |  |  |  |
| GNM 14630_1 | SK9 | 2194_12 | MG025179 |  | MG024743 | MG025399 |
| GNM 14630_2 | SK9 | 2195_12 | MG025180 |  |  |  |
| GNM 14630_3 | SK9 | 2196_12 | MG025181 |  | MG024744 |  |
| GNM 14630_4 | SK9 | 2197_12 | MG025182 |  |  |  |
| GNM 14630_5 | SK9 | 2198_12 | MG025183 |  | MG024745 |  |
| GNM 14630_6 | SK9 | 2199_12 | MG025184 |  | MG024746 |  |
| GNM 14630_7 | SK9 | 2200_12 | MG025185 |  | MG024747 |  |
| GNM 14630_8 | SK9 | 2201_12 | MG025186 |  | MG024748 |  |
| GNM 14631 | SK8 | 2202_12 | MG025187 |  | MG024749 |  |
| GNM 14644 | KA3 | 2222_12 | MG025188 | MG025479 | MG024750 | MG025400 |
| GNM 14644_2 | KA3 | 2223_12 | MG025189 |  | MG024751 |  |
| GNM 14644_3 | KA3 | 2224_12 | MG025190 |  | MG024752 |  |
| GNM 14644_4 | KA3 | 2225_12 | MG025191 |  | MG024753 |  |
| ZMBN 116404 | BS15 | 2806_12 | MG025192 | MG025480 | MG024754 | MG025401 |
| ZMBN 116408 | BS16 | 2818_12 | MG025193 |  | MG024755 |  |
| ZMBN 116406 | BS16 | 2824_12 | MG025194 |  |  |  |
| ZMBN 116407 | BS16 | 2826_12 | MG025195 |  | MG024756 | MG025402 |
| ZMBN 116402 | BS16 | 2827_12 | MG025196 |  |  |  |
| ZMBN 116405 | BS16 | 2829_12 | MG025197 |  | MG024757 | MG025403 |
| ZMBN 116403 | BS16 | 2832_12 | MG025198 |  |  |  |
| GNM 15136 | SK7 | 1201_13 | MG025199 |  | MG024758 |  |
| GNM 15137 | SK11 | 1205_13 | MG025200 |  | MG024759 |  |
| NTNU-VM-66572 | NCS29 | 1923_13 | MG025201 |  | MG024760 |  |
| NTNU-VM-66571 | NCS29 | 1956_13 | MG025202 |  | MG024761 |  |
| NTNU-VM-66568 | NCS29 | 1959_13 | MG025203 |  | MG024762 |  |
| NTNU-VM-66570 | NCS29 | 1960_13 | MG025204 |  | MG024763 |  |
| ZMBN 116414 | BS6 | 1986_13 | MG025205 |  | MG024764 |  |
| ZMBN 116415 | NCS34 | 1998_13 | MG025206 |  |  |  |
| ZMBN 116416 | NCS22 | 1999_13 | MG025207 |  | MG024765 |  |
| ZMBN 116417 | NCS46 | 2027_13 | MG025208 |  | MG024766 |  |
| ZMBN 116418 | NCS46 | 2028_13 | MG025209 | MG025483 | MG024767 | MG025404 |
| NTNU-VM-72560 | NCS39 | 2035_13 | MG025210 |  | MG024768 |  |
| NTNU-VM-72561 | NCS25 | 2038_13 | MG025211 |  | MG024769 |  |
| GNM 14632 | SK12 | 2175_13 |  |  | MG024770 |  |
| ZMBN 116409 | BS8 | 2183_13 | MG025212 |  | MG024771 |  |
| ZMBN 116410 | BS9 | 2184_13 | MG025213 |  | MG024772 |  |
| GNM 14638 | SK14 | 2215_13 | MG025214 |  | MG024773 |  |
| SMF 24659 | GS3 | 2317_13 | MG025215 |  | MG024774 |  |
| ZMBN 116411 | BS11 | 2337_13 | MG025216 |  | MG024775 | MG025405 |
| ZMBN 116412 | NCS3 | 2454_13 | MG025217 |  |  |  |
| ZMBN 116413 | NCS3 | 2458_13 | MG025218 |  | MG024776 | MG025406 |
| NTNU-VM-68195 | NCS25 | 2475_13 | MG025219 | MG025481 | MG024777 | MG025407 |
| ZMBN 116419 | NCS20 | 2776_13 | MG025220 |  | MG024778 |  |
| ZMBN 116420 | NCS15 | 2813_13 | MG025221 |  | MG024779 |  |
| ZMBN 116421 | NCS20 | 2921_13 | MG025222 | MG025482 | MG024780 | MG025408 |
| NTNU-VM-72562 | NCS24 | T01_13 | MG025223 |  | MG024781 |  |
| NTNU-VM-72563 | NCS25 | T03_13 | MG025224 |  | MG024782 |  |
| NTNU-VM-72564 | BS5 | 2040_14 |  |  | MG024783 |  |
| NTNU-VM-72565 | BS5 | 2042_14 | MG025225 | MG025486 | MG024784 | MG025409 |
| NTNU-VM-72566 | BS5 | 2044_14 | MG025226 |  | MG024785 | MG025410 |
| NTNU-VM 68200 | BS5 | 2477_14 | MG025227 |  | MG024786 |  |
| NTNU-VM 68199 | BS5 | 2479_14 | MG025228 |  | MG024787 |  |
| ZMBN 116435 | BS15 | 2811_14 |  |  | MG024788 |  |
| ZMBN 116422 | NCS48 | 2842_14 | MG025229 |  | MG024789 |  |
| ZMBN 116423 | NCS48 | 2843_14 | MG025230 |  | MG024790 |  |
| ZMBN 116424 | NCS48 | 2844_14 | MG025231 |  | MG024791 |  |
| ZMBN 116425 | NCS48 | 2845_14 | MG025232 |  | MG024792 |  |
| ZMBN 116426 | NCS48 | 2846_14 | MG025233 |  | MG024793 |  |
| ZMBN 116427 | NCS48 | 2847_14 | MG025234 |  | MG024794 |  |
| ZMBN 116428 | NCS48 | 2848_14 | MG025235 |  | MG024795 |  |
| ZMBN 116429 | NCS48 | 2850_14 | MG025236 |  | MG024796 |  |
| ZMBN 116430 | NCS48 | 2851_14 | MG025237 |  | MG024797 |  |
| ZMBN 116431 | NCS48 | 2852_14 | MG025238 |  | MG024798 |  |
| ZMBN 116432 | NCS48 | 2853_14 | MG025239 |  | MG024799 |  |
| ZMBN 116433 | NCS48 | 2854_14 | MG025240 | MG025484 | MG024800 | MG025411 |
| ZMBN 116434 | NCS48 | 2855_14 | MG025241 | MG025485 | MG024801 | MG025412 |
| NTNU-VM 68251 | BS5 | POLNOR086_14 | MG025242 |  |  |  |
| ZMBN 116436 | NCS40 | 2003_15 |  |  | MG024802 |  |
| ZMBN 116437 | NCS43 | 2004_15 | MG025243 |  | MG024803 |  |
| ZMBN 116438 | NCS43 | 2005_15 | MG025244 |  | MG024804 |  |
| ZMBN 116439 | NCS43 | 2006_15 | MG025245 |  |  |  |
| ZMBN 116440 | NCS44 | 2007_15 | MG025246 |  |  |  |
| ZMBN 116441 | NCS44 | 2008_15 | MG025247 |  | MG024805 |  |
| ZMBN 116442 | NCS44 | 2009_15 | MG025248 |  | MG024806 | MG025413 |
| ZMBN 116443 | NCS43 | 2010_15 | MG025249 | MG025487 | MG024807 | MG025414 |
| ZMBN 116444 | NCS43 | 2011_15 | MG025250 |  | MG024808 |  |
| ZMBN 116445 | NCS43 | 2012_15 | MG025251 |  | MG024809 |  |
| ZMBN 116446 | NCS45 | 2017_15 | MG025252 |  | MG024810 |  |
| ZMBN 116447 | NCS45 | 2018_15 | MG025253 |  | MG024811 |  |
| ZMBN 116448 | NCS45 | 2019_15 | MG025254 |  | MG024812 |  |
| ZMBN 116449 | NCS45 | 2020_15 |  |  | MG024813 |  |
| ZMBN 116450 | NCS42 | 2025_15 |  |  | MG024814 |  |
| ZMBN 116451 | NCS46 | 2030_15 | MG025255 | MG025489 | MG024815 | MG025415 |
| NTNU-VM-72567 | BS5 | 2043_15 | MG025256 | MG025488 | MG024816 | MG025416 |
| ZMBN 116452 | NCS48 | 2849_15 | MG025257 |  | MG024817 |  |
| SMF 24677 | GS7 | 2267_16 | MG025258 | MG025490 | MG024818 | MG025417 |
| SMF 24678 | SI3 | 2268_16 | MG025259 | MG025491 | MG024819 | MG025418 |
| SMF 24663 | SI3 | 2269_16 | MG025260 | MG025492 | MG024820 |  |
| SMF 24671 | SI1 | 2270_16 | MG025261 |  | MG024821 |  |
| SMF 24646 | SI6 | 2279_16 | MG025262 |  |  |  |
| SMF 24681 | SI6 | 2280_16 | MG025263 |  |  |  |
| SMF 24682 | GS6 | 2284_16 | MG025264 |  |  |  |
| SMF 24674 | GS6 | 2288_16 | MG025265 |  |  |  |
| SMF 24643 | GS6 | 2289_16 | MG025266 |  | MG024822 |  |
| SMF 24645 | GS6 | 2290_16 | MG025267 |  | MG024823 |  |
| SMF 24685 | SI4 | 2292_16 |  |  | MG024824 |  |
| SMF 24652 | SI4 | 2294_16 | MG025268 |  | MG024825 |  |
| SMF 24664 | SI4 | 2295_16 | MG025269 |  | MG024826 |  |
| SMF 24686 | GS4 | 2296_16 | MG025270 |  | MG024827 |  |
| SMF 24655 | GS4 | 2297_16 | MG025271 |  | MG024828 |  |
| SMF 24649 | GS4 | 2298_16 | MG025272 |  | MG024829 |  |
| SMF 24687 | GS4 | 2299_16 | MG025273 |  | MG024830 |  |
| SMF 24642 | GS4 | 2300_16 | MG025274 |  | MG024831 |  |
| SMF 24662 | SI8 | 2303_16 | MG025275 |  | MG024832 |  |
| SMF 24660 | GS7 | 2305_16 | MG025276 |  | MG024833 |  |
| SMF 24653 | GS7 | 2306_16 | MG025277 |  | MG024834 |  |
| SMF 24641 | GS7 | 2307_16 | MG025278 |  | MG024835 |  |
| SMF 24651 | GS7 | 2308_16 | MG025279 |  |  |  |
| SMF 24656 | GS7 | 2309_16 | MG025280 |  | MG024836 |  |
| SMF 24639 | GS7 | 2310_16 | MG025281 |  | MG024837 |  |
| SMF 24658 | GS7 | 2311_16 | MG025282 |  |  |  |
| SMF 24691 | GS1 | 2315_16 | MG025283 |  |  |  |
| SMF 24661 | GS1 | 2316_16 | MG025284 |  | MG024838 |  |
| ZMBN 116453 | BS13 | 2325_16 | MG025285 | MG025493 | MG024839 |  |
| ZMBN 116454 | BS14 | 2330_16 | MG025286 |  | MG024840 |  |
| ZMBN 116455 | BS11 | 2339_16 | MG025287 |  | MG024841 |  |
| ZMBN 116456 | BS11 | 2340_16 | MG025288 |  |  |  |
| ZMBN 116457 | BS11 | 2341_16 |  |  | MG024842 |  |
| ZMBN 116458 | BS11 | 2344_16 | MG025289 |  |  |  |
| ZMBN 116459 | BS11 | 2345_16 | MG025290 |  |  |  |
| ZMBN 116460 | BS14 | 2355_16 | MG025291 |  | MG024843 |  |
| ZMBN 116461 | BS14 | 2356_16 | MG025292 |  | MG024844 |  |
| ZMBN 116462 | BS14 | 2357_16 | MG025293 |  | MG024845 |  |
| ZMBN 116463 | BS14 | 2358_16 | MG025294 |  | MG024846 |  |
| ZMBN 116464 | BS14 | 2359_16 | MG025295 |  | MG024847 |  |
| ZMBN 116465 | BS14 | 2361_16 | MG025296 |  | MG024848 |  |
| ZMBN 116466 | BS14 | 2362_16 | MG025297 |  | MG024849 |  |
| ZMBN 116467 | BS14 | 2363_16 | MG025298 | MG025494 | MG024850 | MG025419 |
| ZMBN 116468 | BS14 | 2364_16 | MG025299 |  | MG024851 |  |
| ZMBN 116469 | BS14 | 2365_16 | MG025300 |  |  |  |
| ZMBN 116470 | BS14 | 2366_16 | MG025301 |  | MG024852 |  |
| ZMBN 116471 | BS14 | 2373_16 | MG025302 |  | MG024853 |  |
| ZMBN 116472 | NWS1 | 2374_16 | MG025303 |  | MG024854 |  |
| ZMBN 116473 | BS12 | 2379_16 |  |  | MG024855 |  |
| ZMBN 116474 | BS10 | 2389_16 | MG025304 | MG025495 | MG024856 | MG025420 |
| ZMBN 116475 | BS10 | 2391_16 |  |  | MG024857 |  |
| SMF 24675 | GS5 | 2462_16 |  |  | MG024858 |  |
| SMF 24693 | SI2 | 2466_16 | MG025305 |  | MG024859 |  |
| SMF 24644 | SI2 | 2467_16 | MG025306 |  | MG024860 | MG025421 |
|  | SI2 | 2468_16 | MG025307 |  |  |  |
| SMF 24650 | SI2 | 2469_16 | MG025308 |  | MG024861 | MG025422 |
| SMF 24654 | SI2 | 2470_16 | MG025309 |  | MG024862 |  |
| SMF 24640 | SI2 | 2471_16 | MG025310 |  | MG024863 |  |
| SMF 24648 | SI2 | 2472_16 | MG025311 |  | MG024864 |  |
| SMF 24647 | SI2 | 2473_16 |  |  | MG024865 |  |
| SMF 24657 | SI2 | 2474_16 | MG025312 |  | MG024866 |  |
| ZMBN 116476 | GS8 | 2838_16 |  |  | MG024867 |  |
| SMF 24676 | SI9 | 2274_17 | MG025313 | MG025496 | MG024868 | MG025423 |
| SMF 24689 | SI5 | 2312_18 | MG025314 |  |  |  |
| SMF 24690 | SI5 | 2313_18 | MG025315 | MG025497 | MG024869 | MG025424 |
| SMF 24368 | SI5 | 2314_18 | MG025316 | MG025498 | MG024870 | MG025425 |
| SMF 24680 | SI6 | 2278_19 | MG025317 | MG025499 | MG024871 | MG025426 |
| SMF 24636 | SI8 | 2302_20 | MG025318 | MG025500 | MG024872 | MG025427 |
| ZMBN 116477 | NCS33 | 2349_20 | MG025319 | MG025501 | MG024873 | MG025428 |
| ZMBN 116478 | BS11 | 2342_21 | MG025320 | MG025502 | MG024874 | MG025429 |
| ZMBN 116479 | BS12 | 2377_21 | MG025321 |  |  |  |
| ZMBN 116480 | BS10 | 2384_21 | MG025322 |  |  |  |
| ZMBN 116481 | BS10 | 2385_21 | MG025323 |  |  |  |
| ZMBN 116484 | BS16 | 2815_21 | MG025324 |  |  |  |
| ZMBN 116482 | BS16 | 2816_21 | MG025325 |  |  |  |
| ZMBN 116483 | BS16 | 2817_21 | MG025326 |  |  |  |
| ZMBN 116493 | BS16 | 2819_21 | MG025327 |  |  |  |
| ZMBN 116494 | BS16 | 2820_21 | MG025328 |  |  |  |
| ZMBN 116485 | BS16 | 2823_21 | MG025329 |  |  |  |
| ZMBN 116486 | BS16 | 2825_21 | MG025330 |  |  |  |
| ZMBN 116495 | BS16 | 2830_21 | MG025331 |  |  |  |
| ZMBN 116487 | BS16 | 2831_21 | MG025332 |  |  |  |
| ZMBN 116488 | BS16 | 2833_21 | MG025333 |  |  |  |
| ZMBN 116489 | BS16 | 2834_21 | MG025334 |  |  |  |
| ZMBN 116490 | BS16 | 2836_21 | MG025335 |  |  |  |
| ZMBN 116491 | BS18 | 2894_21 | MG025336 |  | MG024875 |  |
| ZMBN 116492 | BS17 | 2895_21 | MG025337 |  |  |  |
| SMF 24672 | SI9 | 2277_22 | MG025338 | MG025503 | MG024876 | MG025430 |
| SMF 24637 | SI7 | 2281_23 | MG025339 | MG025504 | MG024877 | MG025431 |
| ZMBN 116496 | AO2 | 2864_24 | MG025340 |  |  |  |
| ZMBN 116497 | AO3 | 2865_24 | MG025341 | MG025505 | MG024878 |  |
| ZMBN 116500 | AO3 | 2866_24 | MG025342 | MG025506 | MG024879 | MG025432 |
| ZMBN 116498 | AO1 | 2867_24 | MG025343 | MG025507 | MG024880 | MG025433 |
| ZMBN 116501 | AO1 | 2868_24 |  | MG025508 | MG024881 | MG025434 |
| ZMBN 116499 | AO3 | 2869_24 | MG025344 |  |  |  |
| ZMBN 116503 | BS15 | 2801_25 | MG025345 | MG025509 | MG024882 | MG025435 |
| ZMBN 116502 | BS15 | 2802_25 | MG025346 |  | MG024883 |  |
| ZMBN 116504 | BS15 | 2809_25 | MG025347 | MG025510 | MG024884 | MG025436 |
| ZMBN 116505 | BS15 | 2810_25 | MG025348 |  |  |  |
| ZMBN 116506 | BS15 | 2805_26 | MG025349 | MG025511 | MG024885 | MG025437 |
| ZMBN 116508 | BS15 | 2807_26 |  | MG025512 | MG024886 | MG025438 |
| ZMBN 116507 | BS15 | 2808_26 |  |  | MG024887 |  |
| ZMBN 116509 | BS15 | 2800_27 | MG025350 | MG025513 | MG024888 | MG025439 |
| ZMBN 116510 | NCS31 | 2324_28 | MG025351 | MG025514 | MG024889 | MG025440 |
| ZMBN 116511 | BS14 | 2329_28 | MG025352 |  | MG024890 |  |
| ZMBN 116512 | NCS33 | 2348_28 | MG025353 | MG025515 | MG024891 |  |
| ZMBN 116513 | NCS18 | 2875_28 | MG025354 | MG025516 | MG024892 | MG025441 |
| ZMBN 116514 | NCS11 | 2903_28 | MG025355 | MG025517 | MG024893 |  |
